# A Novel Class of “Super‐Strained” Spiro Heterocycles: Gateway to 1‐Azaspiro[3.3]heptane Derivatives, and Biological Validation

**DOI:** 10.1002/anie.3488479

**Published:** 2026-06-30

**Authors:** Philipp Natho, Annarita Vicenti, Marco Colella, Francesco Pasca, Giuseppe Romanazzi, Mauro Niso, Carmen Abate, Ernesto Mesto, Emanuela Schingaro, Renzo Luisi

**Affiliations:** ^1^ Department of Pharmacy‐Drug Sciences University of Bari Aldo Moro Bari Italy; ^2^ DICATECh Politecnico di Bari Bari Italy; ^3^ Department of Earth and Geoenvironmental Sciences University of Bari Aldo Moro Bari Italy

**Keywords:** azabicyclo[1.1.0]butane, azetidine, oxetane, predictive analytics, spirocycle, spiro[3.3]heptanes

## Abstract

Strained spiro heterocycles have gained popularity in medicinal chemistry due to their potential to act as conformationally rigid non‐classical three‐dimensional bioisosteres. Recently, 1‐azaspiro[3.3]heptane has been validated as a piperidine bioisostere, although synthetic methods available for functionalized or heteroatom‐containing derivatives are scarce. We address this shortcoming by accessing spirocyclic 1‐azabicyclo[1.1.0]butanes—a novel class of “super‐strained” spirocycles—through a Johnson‐Corey‐Chaykovsky reaction between cyclobutane‐, oxetane‐, and azetidine‐substituted sulfonium salts, and azirines. We demonstrate that such spirocycles are suitable precursors for highly functionalized 1‐azaspiro[3.3]heptane derivatives. In addition, such super‐strained spirocycles have been validated in vitro as potential sigma‐1 receptor agonists, a target identified through an artificial intelligence‐supported target fishing approach.

## Graphical abstract

A new class of “super‐strained” spiro heterocycles—spirocyclic 1‐azabicyclo[1.1.0]butanes—was synthesized via insertion of cyclobutane‐, oxetane‐, and azetidine‐containing sulfonium reagents into substituted azirines. The stability of this new class of compounds was studied. In addition, their value is demonstrated by two applications: first, by allowing access to highly functionalized 1‐azaspiro[3.3]heptanes, which are validated piperidine bioisosteres, and second, as biologically active molecules. Their high in vitro affinity for the human sigma‐1 receptor is demonstrated; a target which was identified by a predictive analytics‐supported target fishing approach.

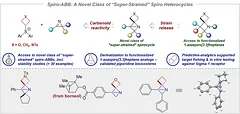

## 1 Introduction

Strained spiro heterocycles have become valuable frameworks in modern drug design as their properties align with important features related to *escape from flatland* [1, 2] and *conformational restriction* concepts [3]. Following the design transition from replacing two‐dimensional aromatic scaffolds with saturated non‐strained heterocycles to introduce three‐dimensionality in drug candidates, the community is now witnessing a drive to replace such non‐strained heterocycles with strained spiro heterocycles [4, 5, 6, 7]. This follows the observation that, unlike their non‐strained heterocyclic counterparts, the structurally induced key characteristics of strained spiro heterocycles (high *sp^3^*‐fraction, good balance between rigidity and flexibility) offer a series of desirable attributes for modern drug design, such as well‐defined positioning of exit vectors in space, in addition to improved physicochemical properties, including reduced lipophilicity, improved metabolic stability, and enhanced target selectivity [7].

**FIGURE 1:**
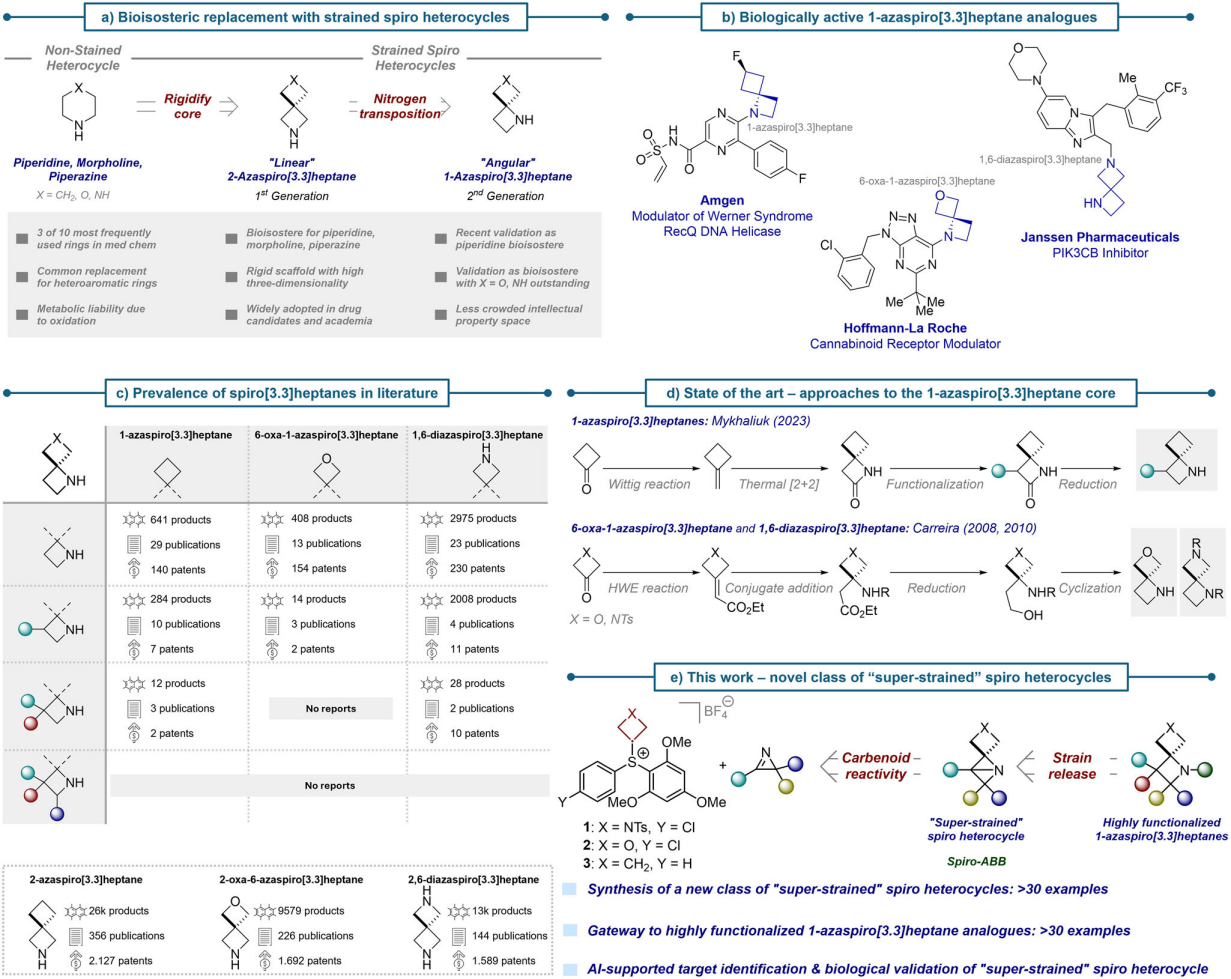
(a) Strained spiro heterocycles as bioisosteres for non‐strained saturated heterocycles. (b) Biologically active 1‐azaspiro[3.3]heptane analogs. (c) Prevalence of spiro[3.3]heptanes in literature. Data obtained from CAS SciFinder through sub‐structure search and further filtering for “available as product/preparation available.” Reviews were excluded. Accessed: February 27, 2026. (d) State of the art approaches to 1‐azaspiro[3.3]heptane analogs. (e) This work—sulfonium salts as precursors to “super‐strained” spiro heterocycles and functionalized 1‐azaspiro[3.3]heptane analogs.

It is thus unsurprising that a series of strained spiro heterocycles have been deliberately designed to mimic the geometries and properties of non‐strained heterocycles (Figure 1a, [8, 9, 10, 11, 12, 13, 14, 15, 16, 17, 18, 19, 20, 21, 22]). Heteroatom‐containing spiro[3.3]heptane derivatives were found particularly suitable for this purpose and various bioisosteric replacements have been validated, leading to the incorporation of these motifs into lead compounds or drug candidates currently undergoing clinical trials. For example, “linear” 2‐azaspiro[3.3]heptane was developed in 2010 as a bioisostere for piperidine [10], one of the top three most frequently used rings in medicinal chemistry present in over 30 marketed drugs [23]. In the last 15 years, replacement of the piperidine ring with 2‐azaspiro[3.3]heptane was widely adopted, showcased by its inclusion in over 2000 patents and over 26 000 products [24]. In addition, its oxygen‐ and nitrogen‐containing analogs were also validated as morpholine‐ and piperazine‐bioisosteres, respectively [4].

In 2023, its “angular” analog—1‐azaspiro[3.3]heptane—bearing the nitrogen atom adjacent to the spiro carbon, has also been validated as a piperidine bioisostere (Figure 1a, [25]). In comparison to its “linear” analog, important physicochemical properties (incl. basicity, solubility, logD) are nearly identical, although, pleasingly, it can induce higher metabolic stability. In addition to the beneficial physicochemical properties, it occupies a less crowded intellectual property space, offering additional incentives for drug discovery programs. Indeed, recently patented lead compounds incorporating 1‐azaspiro[3.3]heptane and its analogs bearing additional heteroatoms demonstrate a remarkable versatility across multiple therapeutic areas (Figure 1b) [26, 27, 28].

Despite the proven benefits of incorporating 1‐azaspiro[3.3]heptane analogs in drug candidates, it becomes apparent that their preparation and accessible substitution pattern is currently rather limited. Indeed, a survey on CAS SciFinder reveals three key limitations (Figure 1c) [24]. First, “angular” 1‐azaspiro[3.3]heptane derivatives are significantly underrepresented in comparison to their linear 2‐azaspiro[3.3]heptane analogs. For example, whereas ca. 13 000 and 10 000 products are described for 2,6‐diazaspiro[3.3]heptane and 2‐oxa‐6‐azaspiro[3.3]heptane, respectively, only ca. 3000 and 400 products are reported for their angular analogs. Second, in comparison to 1,6‐diazaspiro[3.3]heptane, for which ca. 3000 analogs are reported, the cyclobutane‐ and oxetane‐bearing analogs are severely underrepresented (approx. 600 and 400 compounds, respectively). And third, the number of reported substrates with available synthetic procedures drops significantly when the azetidine ring is decorated with substituents. In fact, only 14 6‐oxa‐1‐azaspiro[3.3]heptane derivatives bearing a substituent in the 3‐position are reported, and only 12 derivatives of 1‐azaspiro[3.3]heptane bearing a 3,3‐bis‐functionalization substitution pattern, and those are mostly limited to dimethyl‐ or difluoro‐substituted analogs [17].

The aforementioned three key observations are a consequence of a lack of general synthetic methods available for efficiently incorporating such motifs (Figure 1d). For 1‐azaspiro[3.3]heptane derivatives, although other procedures have been reported [29, 30], the most general route consists of a multistep sequence starting from cyclobutanone (derivatives), followed by a Wittig‐reaction and thermal [2+2]‐cycloaddition with the Graf isocyanate, followed by reduction of the β‐lactam ring [25]. Substitution on the 3‐position can be introduced prior to reduction of the carbonyl group. Notably, however, this method precludes the synthesis of 6‐oxa‐1‐azaspiro[3.3]heptane or 1,6‐diazaspiro[3.3]heptane derivatives. These analogs can be prepared through a multistep sequence starting from oxetan‐3‐one or azetidin‐3‐one, respectively, although this route does not permit the efficient installation of substituents [31, 32]. An alternative sequence for the preparation of 3,3‐*gem*‐difluoro‐ and 3,3‐*gem*‐dimethyl‐1,6‐diazaspiro[3.3]heptanes from a suitably substituted azetidin‐3‐one has been reported, yet also, in this case, substitution with alternative functionality is not explored [17]. The development of a general, modular approach to all three classes of derivatives with potential for variable substitution around the azetidine ring is thus imperative.

In continuation with our research interest in the development and biological validation of unprecedented strained spiro heterocycles, we pursued an approach that would address current limitations allowing modular access to all three 1‐azaspiro[3.3]heptane analogs, while enabling versatile substitution pattern around the azetidine ring. Drawing from our expertise, and the recent resurgence of methods for the synthesis of highly functionalized azetidines by strain‐release of 1‐azabicyclo[1.1.0]butanes [21, 33, 34, 35], we questioned whether a new class of “super‐strained” spiro heterocycles—spirocyclic 1‐azabicyclo[1.1.0]butanes (spiro‐ABB)—could offer a promising gateway to accessing 1‐azaspiro[3.3]heptane derivatives with a more versatile substitution pattern (Figure 1e). The inclusion of additional ring strain would allow subsequent intermolecular functionalization of the “spring‐loaded” bridgehead bond to deliver functionalized 1‐azaspiro[3.3]heptane derivatives. As the required spirocyclic 1‐azabicyclo[1.1.0]butanes, to the best of our knowledge, are hitherto unknown, we hypothesized that a Johnson‐Corey‐Chaykovsky reaction between azirines and our recently developed four‐membered ring‐bearing sulfonium salts **1** – **3** [36] could furnish the desired spirocyclic 1‐azabicyclo[1.1.0]butanes (Figure 1e). Herein, we report the successful implementation of this methodology securing access to new chemical space for strained and “super‐strained” spiro heterocycles. Given the proven utility of such heterocycles in biologically active drug candidates, we also questioned if the new class of “super‐strained” spiro heterocycles could be equally effective. Thus, we also report preliminary results on the AI‐supported target identification and in vitro validation of spirocyclic 1‐azabicyclo[1.1.0]butane derivatives as potent sigma‐1 receptor agonists.

## 2 Results and Discussion

### 2.1 Optimization

**SCHEME 1:**
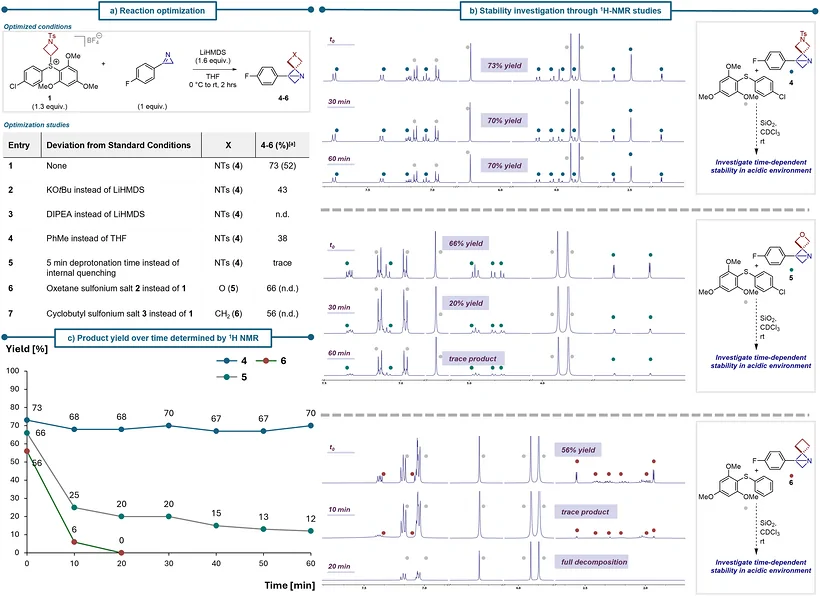
(a) Reaction optimization. ^[a]^ Yields reported are quantitative ^1^H‐NMR yields. Yields in brackets are isolated yields. (b) Stability investigation through ^1^H‐NMR studies. (c) Development of product yield determined by ^1^H‐NMR over time in the presence of silica gel.

At the outset, we tested the proof of concept for the synthesis of “super‐strained” spiro heterocycles required as key intermediates for the synthesis of highly functionalized 1‐azaspiro[3.3]heptane analogs. According to our design plan, we questioned if such spiro‐ABBs could be accessible from a Johnson‐Corey‐Chaykovsky reaction between suitably substituted sulfonium salts and azirines. Thus, we selected 3‐(4‐fluorophenyl)‐2*H*‐azirine as a model compound in combination with azetidinyl sulfonium salt **1** (Scheme 1a). Upon optimization of the reaction conditions (see Supporting Information), we found that treatment of a mixture of the azirine and 1.3 equivalents of the sulfonium salt with LiHMDS (1.6 equiv.) in THF at 0°C afforded the desired spirocyclic 1‐azabicyclo[1.1.0]butane **4** in 73% yield within 2 h (Entry 1). Replacement of LiHMDS with other organic or inorganic bases reduced the efficiency of the process (Entries 2–3), as did substitution of THF with toluene (Entry 4). Drawing from our previous results on the reactivity of the corresponding ylids, it was unsurprising that conducting the reaction under internal quenching conditions delivered superior results compared to external quenching conditions (Entry 5). With the aim to further expand the methodology to other spiro‐ABBs, we tested the optimized reaction conditions for the preparation of the corresponding oxetane‐ and cyclobutyl‐ analogs **5** and **6**. Treatment of sulfonium salts **2** and **3** with 3‐(4‐fluorophenyl)‐2*H*‐azirine under standard conditions cleanly delivered the corresponding spirocycles **5** and **6** in 66% and 56% yield, respectively (entries 6–7). To our surprise, however, in contrast to azetidine analog **4**, any attempts to isolate the desired products by flash column chromatography failed even when the silica gel was pre‐treated with triethylamine, and even on alumina, which indicated to us very strong acid sensitivity of the oxetane‐ and cyclobutyl analogs **5** and **6**. Intrigued by this observation, we undertook ^1^H‐NMR studies to test the stability of the three products in the presence of silica gel (Scheme 1b). Whereas the azetidine analog **4** showed little to no sign of decomposition even after one hour of contact time with silica gel, in line with our expectation, the oxetane‐ and cyclobutyl‐analogs **5** and **6** underwent rapid acid‐mediated decomposition. Specifically, more than 60% of the oxetane‐analog **5** decomposed within the first ten minutes of contact with silica gel, and even more drastically the cyclobutyl analog **6** decomposed almost completely within ten minutes (Scheme 1c). Although 1‐azabicyclo[1.1.0]butanes are generally considered sufficiently stable for isolation by flash column chromatography, it is plausible that the presence of a spiro‐center increases strain and thus weakens the sensitive bridgehead bond towards acid‐mediated opening.

### 2.2 Scope Evaluation

**SCHEME 2:**
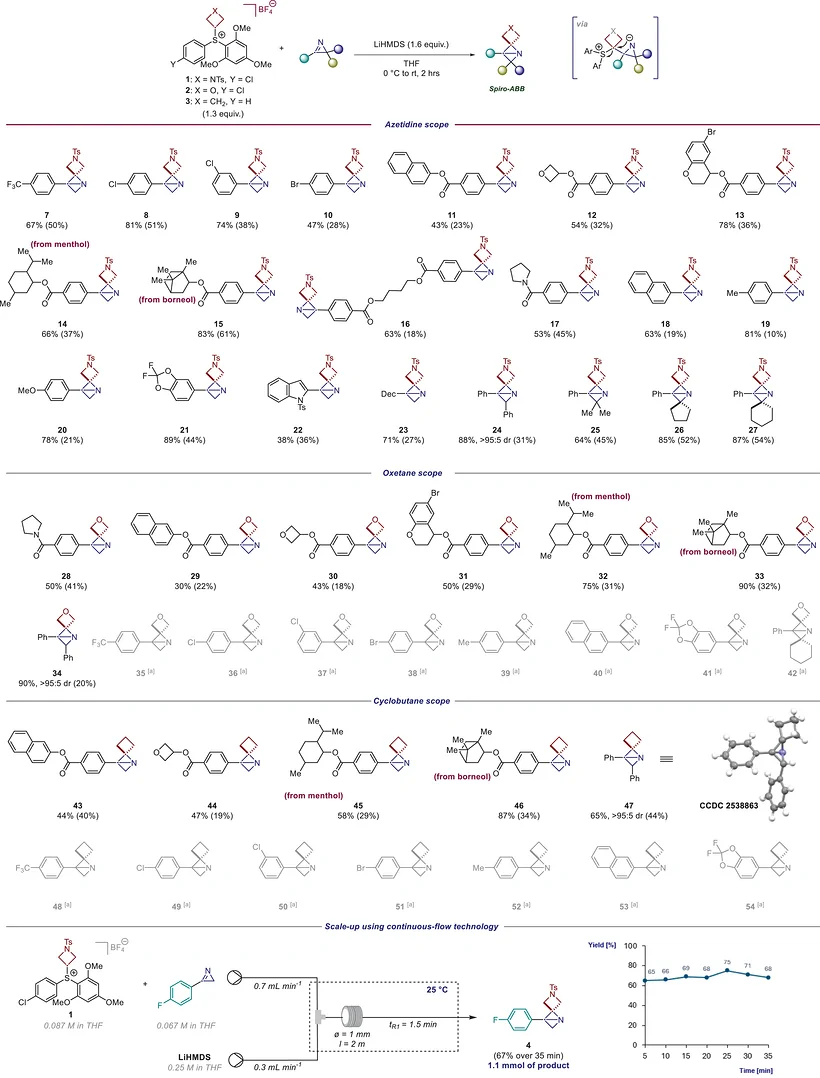
Scope exploration for the synthesis of spiro‐ABBs using sulfonium salts **1**–**3**. Yields reported are quantitative ^1^H‐NMR yields with isolated yields reported in brackets. Isolated yields differ from ^1^H‐NMR yields due to the observed sensitivity of most compounds to long exposure to acidic environments. ^[a]^ Not isolated. Engaged in a subsequent strain‐release reaction, see Scheme 3.

**SCHEME 3:**
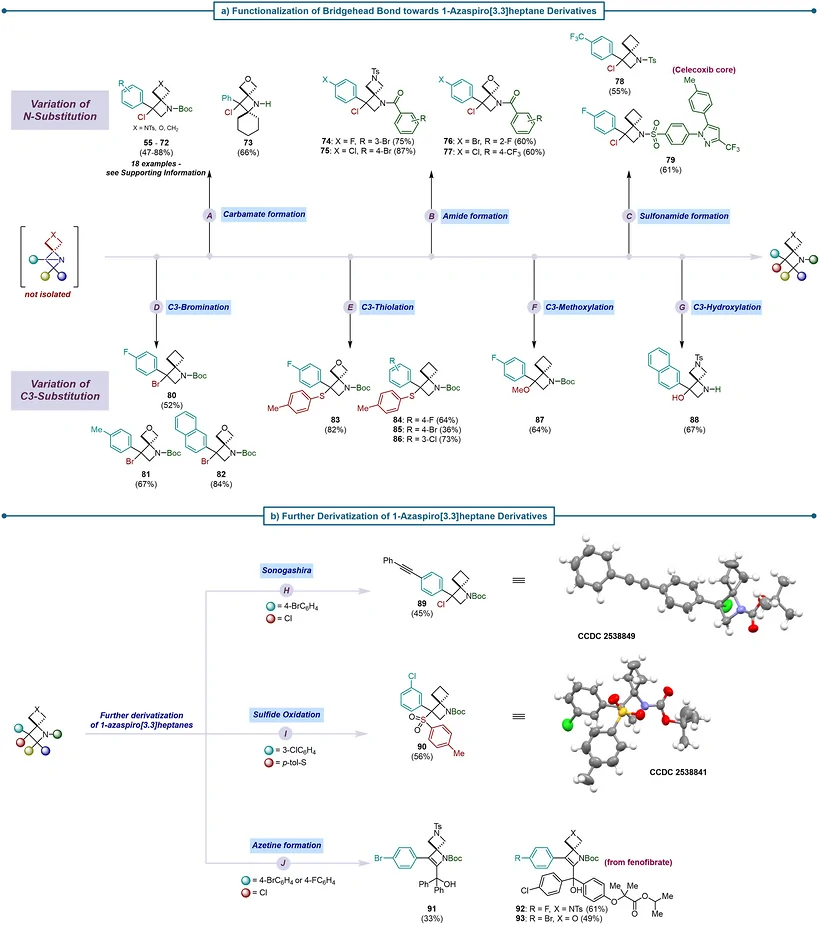
(a) Functionalization of bridgehead bonds for the synthesis of 1‐azaspiro[3.3]heptane derivatives. (b) Further derivatization of 1‐azaspiro[3.3]heptane derivatives. (A) Boc_2_O (2 equiv.), LiCl (3 equiv.), MeCN, rt, overnight; (B) acyl chloride (2 equiv.), Amberlyst, MeCN, rt, overnight; (C) sulfonyl chloride (2 equiv.), Amberlyst, MeCN, rt, overnight; (D) Boc_2_O (2 equiv.), LiBr (3 equiv.), MeCN, rt, overnight; (E) thiol (2 equiv.), THF, 0°C to rt, overnight; then K_2_CO_3_ (2 equiv.), Boc_2_O (2 equiv.), 0°C to rt, 3 hrs; (F) HBr (aq. 48%, 1 equiv.), MeOH, 0°C to rt, overnight; then Boc_2_O (2 equiv.), K_2_CO_3_ (2 equiv.), 0°C to rt, 3 hrs; (G) TFAA (1.2 equiv.), CH_2_Cl_2_, 0°C, 6 hrs; then NaOH (aq. 10%)/MeOH, rt, overnight; (H) phenylacetylene (1.1 equiv.), Pd(PPh_3_)_4_ (5 mol%), CuI (5 mol%), Et_3_N, THF, 65°C, overnight; (I) *m*CPBA (2.5 equiv.), CH_2_Cl_2_, rt, 3 hrs; (J) LDA (2.2 equiv.), ketone (1.2 equiv.), THF, −70°C, 2 hrs.

With a protocol for the synthesis of spiro‐ABBs in hand, we sought to evaluate the scope of the present transformation (Scheme 2). Following our findings on the relative stability imposed by the azetidine ring, we began our exploration with those analogs. In addition to 3‐(4‐fluorophenyl)‐2*H*‐azirine, also azirines bearing alternatively substituted aromatic rings gave the corresponding spiro‐ABBs in generally good yields. We tested a series of azirines bearing electronically diverse aromatic groups. For example, azirines bearing electron‐withdrawing groups, including trifluoromethyl‐, chloro‐, or bromo‐groups, afforded the desired products **7**–**10** in 47%–81% yield. Especially the introduction of bromo‐substituted aromatic groups was of importance for further derivatizations (see Scheme 3). In addition, other electron‐withdrawing groups such as amides and esters were tolerated, providing the products **11**–**17** in up to 83% yield. Particularly esters were well‐suited for the introduction of medicinally relevant or biologically active motifs, including oxetane, chromanol or terpenes, such as borneol and menthol. Remarkably, using 1,4‐butanediol as the azirines’ linker, two spiro‐ABBs can be installed obtaining derivative **16** in 63% yield.

We then turned our attention to azirines bearing more electron‐rich aromatic rings. The reaction of naphthyl‐substituted azirine afforded the desired spirocycle **18** in 63% yield. Similarly, azirines substituted with phenyl rings bearing methyl‐ or methoxy‐groups in the *para*‐position facilitated the formation of spiro‐ABBs **19**–**20** in up to 81% yield. Representative pharmaceutically relevant building blocks such as 2,2‐difluoro‐1,3‐benzodioxole (**21**) and indole (**22**) were successfully incorporated. Extension to other heterocycles is primarily limited by the lack of suitable methods available for the preparation of the corresponding azirines. Also, an aliphatic chain (**23** in 71% yield) is tolerated, highlighting that the presence of an aromatic group is not essential.

One of our design objectives was to enable access to 1‐azaspiro[3.3]heptane derivatives with multiple substituents on the azetidine ring. Meeting this target demands the synthesis of spiro‐ABBs with additional substitution on the 2‐position. Thus, we extended the protocol to poly‐substituted azirines. For example, reaction with 2,3‐diphenyl‐2*H*‐azirine afforded the bis‐phenyl‐substituted spirocycle **24** in 88% yield as a single diastereomer. To our delight, unprecedented fully substituted 1‐azabicyclo[1.1.0]butanes were also accessible. Analog **25** bearing two methyl groups in the 2‐position was successfully afforded in 64% yield, as were synthetically challenging bis‐spirocyclic products **26** and **27** in up to 87% yield. It is worth noting that attempts to remove the tosyl protecting group under single electron reduction conditions (Na/THF) resulted in substrate decomposition.

After assessing the scope for the azetidine‐analogs, we continued our exploration of the substrate scope with respect to the corresponding oxetane‐ and cyclobutane‐derivatives. We mainly focused on substrates bearing ester‐ and amide‐functionality, as we discovered that these substrates were sufficiently stable to flash column chromatography. To our delight, reaction with azirines bearing aromatic groups substituted with esters or amides afforded isolable spirocycles **28**–**33** and **43**–**46** in up to 90% yield, including biorelevant examples such as borneol and menthol. Notably, also reaction with 2,3‐diphenyl‐2*H*‐azirine afforded the desired spirocycles **34** & **47** in 90% and 65% yield, respectively. Also, these analogs showed relative high stability, and the structure of cyclobutyl‐derivative **47** was confirmed by single crystal x‐ray crystallography as the (3R*,4S*)‐isomer [37]. It is important to note that the presence of the spirocycle induces high diastereoselectivity (>95:5 dr), with the phenyl ring on C2 placed in the exo‐position to minimize steric repulsion with the cyclobutyl ring. Last, we tested azirines substituted with other electronically diverse aromatic rings (**35**‐**42** and **48**–**54**). Although these could successfully be employed affording the desired spiro‐ABBs, any attempt of isolation failed due to their acid‐sensitivity. Nonetheless, these substrates could be directly engaged in the subsequent strain‐release protocol to afford the corresponding 1,3‐functionalized azetidines (see Scheme 3).

To conclude, for our subsequent studies on the application of these spiro‐ABBs, we required a scalable procedure that would allow rapid synthesis of the desired products. We thus turned to flow technology [38, 39] and found that this reaction was indeed amenable to continuous flow synthesis. Remarkably, in a PTFE microtubing reactor, full conversion was achieved with a residence time of only 1.5 min at room temperature (vs. 2 h in batch), thus not only eliminating the requirement for cooling, but also drastically reducing reaction duration. A long run experiment allowed the processing of 1.6 mmol of azirine within 35 min, affording the desired spiro‐ABB **4** in 67% yield—comparable to that observed in batch processing, which reduces the processing time for over 1 mmol of product by over 70% (2 h in batch vs. 35 min in continuous flow). It is worth noting that the yield was found to be stable over this timeframe, indicating that this transformation is potentially suitable also for longer processing times.

### 2.3 Synthetic Transformations and Derivatization

Having demonstrated scalability and range of spiro‐ABBs that could be accessed using this methodology, we were interested in leveraging these compounds for two applications: (i) access “angular” 1‐azaspiro[3.3]heptane derivatives, which are validated bioisosteres, and (ii) to assess their potential as bioactive molecules, as these motifs have so far been unexplored.

We began our study with synthetic transformations to expand the accessible chemical space of (heteroatom‐containing) 1‐azaspiro[3.3]heptanes, and thus studied transformations for the 1,3‐functionalization of the “spring‐loaded” bridgehead bond with various electrophilic activators and nucleophiles (Scheme 3a). For example, treatment of spiro‐ABBs with lithium chloride and di‐*tert*‐butyl dicarbonate affords the *N*‐Boc protected 3‐chlorinated azetidines **55**–**72** in up to 88% yield over two steps. Notably, this reaction proceeds well without the necessity for prior isolation of the spiro‐ABBs, and thus permits access to 1‐azaspiro[3.3]heptanes derivatives even from non‐isolable super‐strained precursors (e.g., **35**–**42** and **48**–**54**). The reaction proceeded well regardless of the steric or electronic properties of the aryl group, or the nature of the four‐membered ring connected to the 1‐azabicyclo[1.1.0]butane. It is worth noting that under these reaction conditions bis‐spirocyclic 1‐azabicyclo[1.1.0]butane **42** underwent strain‐release through 3‐chlorination, yet without concomitant *N*‐Boc protection (**73**), presumably due to steric factors considering the double spiro‐substitution on the central azetidine ring.

Next, we studied alternative electrophilic activators to affect the desired 1,3‐functionalization. In addition to di‐*tert*‐butyl dicarbonate, also acyl chlorides were found competent electrophilic activators, affording amides **74**–**77** in 60%–87% yield. Last, we tested sulfonyl chlorides as electrophilic activators of the bridgehead bond. Sulfonamides **78** and **79** were thus obtained in 55%–61% yield over two steps. We were delighted to find that this protocol was amenable to complex settings as it enabled the installation of the celecoxib core, a marketed COX‐2 inhibitor.

Having explored a range of electrophilic activators, we turned our attention to variation of the substituent on C3 position. Effective nucleophiles beyond lithium chloride included lithium bromide to obtain 3‐brominated 1‐azaspiro[3.3]heptane derivatives **80**–**82**, as well as aromatic thiols delivering sulfides **83**–**86** in high yields over two steps. Unfortunately, extension to *N*‐(*tert*‐butoxycarbonyl)‐L‐cysteine methyl ester as an alternative thiol‐containing nucleophile failed to deliver the desired product. A two‐step protocol of acid‐mediated strain‐release in methanolic hydrogen bromide, followed by *N*‐protection affords 3‐methoxylated *N*‐Boc protected 1‐azaspiro[3.3]heptane **87** in 64% yield over two steps. Interestingly, also *N*‐protecting group‐free azetidin‐3‐ols are accessible by a two‐step procedure consisting of anhydride‐mediated strain‐release, followed by substitution and deprotection in basic media, affording spirocycle **88** in 67% yield.

To further extend the diversity of accessible products, we engaged the obtained 1‐azaspiro[3.3]heptane analogs in further derivatization (Scheme 3b). For example, 4‐bromophenyl‐substituted 1‐azaspiro[3.3]heptane was a suitable substrate for palladium‐catalyzed cross‐coupling and could be engaged in a Sonogashira coupling to afford alkyne derivative **89** in 45% yield. The structure of **89** was confirmed by single crystal x‐ray crystallography [37]. Beyond palladium‐catalyzed cross‐coupling reactions, the motif is tolerant to strongly oxidative conditions, and sulfide **86** could be converted to the corresponding sulfone **90** with an excess of *meta*‐chloroperbenzoic acid. Also, this structure was furthermore confirmed by single crystal x‐ray crystallography [37]. Last, we hypothesized that the 3‐chloro‐substituted derivatives were amenable to an elimination/deprotonation/functionalization protocol to afford functionalized spirocyclic azetines—a spirocyclic class of compound currently inaccessible. To our delight, treatment with 2.5 equivalents of lithium diisopropylamide at −78°C and a benzophenone derivative under internal quenching conditions, affords the desired spirocyclic azetines **91–93** in up to 61% yield. Also this transformation was applicable to structurally complex settings which is showcased by the installation of fenofibrate (**92** & **93**), a marketed drug for the treatment of abnormal blood lipid levels.

### 2.4 Validation of the Bioactivity of Spiro‐ABBs

To conclude our study and in line with our objectives, we turned our attention to testing the potential suitability of spirocyclic 1‐azabicyclo[1.1.0]butanes in bioactive molecules. This hypothesis was guided by the fact that strained spiro heterocycles have already been well‐adopted in drug discovery programs with several derivatives currently undergoing clinical trials.

**SCHEME 4:**
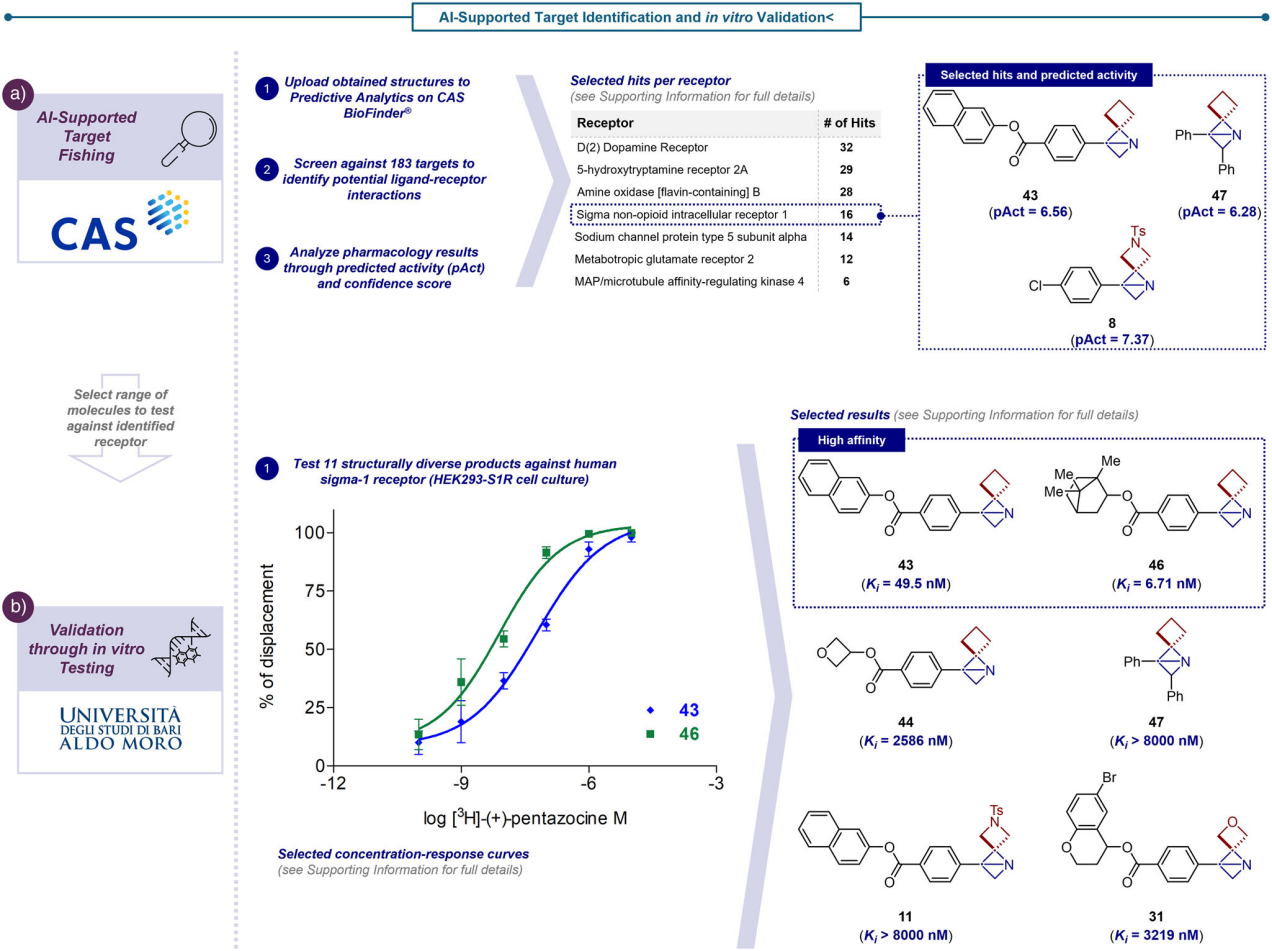
AI‐Supported target identification and in vitro validation. (a) AI‐supported target fishing using CAS BioFinder. See Supporting Information for full details. (b) Validation of target fishing results through in vitro testing using human embryonic kidney cells stably transfected with human sigma‐1 receptor (HEK293‐S1R cell culture).

Given that this class of compounds is hitherto unexplored, we first sought potential targets. To identify potential receptor targets and streamline our in vitro efforts, we initially turned to AI‐enhanced predictive target identification using CAS BioFinder (Scheme 4a). Using the predictive analytics functionality, we screened all our obtained spiro‐ABBs in silico against a panel of 183 protein targets (e.g., 5‐hydroxytryptamine receptor 1A, alpha‐1A adrenergic receptor, D(2) dopamine receptor, opioid receptors, etc.). Potential ligand‐to‐target interactions were evaluated based on a cluster of five different predictive models (e.g., similarity‐based method, or cross‐pharmacology index), which are combined to predict a consensus of how likely a ligand is to interact with a specific target (pAct) (see Supporting Information for further details). Evaluation of the results obtained from the in silico target fishing approach, revealed that a series of analogs had a high likelihood of predicted activity for interaction. The top 10% of targets with the highest predicted number of interactions with the ligand set include the D(2) dopamine receptor (32 predicted hits), carbonic anhydrase 2 (23 hits) or the sigma non‐opioid intracellular receptor 1 (sigma‐1 receptor) (16 hits). We focused our interest on the last target, defined as a pluripotent chaperone, endowed with a plethora of therapeutic potentials [40], that span from oncology to neurodegenerative diseases, such as Huntington diseases, amyotrophic lateral sclerosis [41] and Alzheimer's disease [42, 43].

Based on these in silico predictions, we decided to test a range of analogs in the sigma‐1 receptor radioligand competition binding assay (Scheme 4b). Specifically, we used [^3^H]‐(+)‐pentazocine as the radioligand and membranes of human embryonic kidney cells stably transfected with human sigma‐1 receptor (HEK293‐S1R cell culture) as source of sigma‐1 receptor. We were delighted to find that two analogs displayed affinity in the nM‐range. Specifically, **43** and **46** displayed inhibition constants (*K_i_* values) of 49.5 and 6.71 nM, respectively, indicating strong interaction with the sigma‐1 receptor. Despite the presence of a reactive carbon‐nitrogen bridgehead bond, at this stage neither the competition binding curves (Hill slopes 0.8–1.1) nor extended incubation experiments (no meaningful changes after an additional 60 min incubation) provided evidence for irreversible or covalent receptor engagement under the assay conditions employed. Cross‐study comparisons with reported literature values suggest that the observed inhibition constants are comparable in magnitude to those of known sigma‐1 receptor ligands [44], including clinically investigated compounds such as pridopidine and blarcamesine [41, 42].

It is further worth noting that ligand **43** was predicted to have likely interaction with the target by CAS BioFinder (pAct = 6.56), showcasing the power of artificial intelligence‐supported approaches to identify potential targets for compounds of new chemical space, which likely would not have been conceptualized through rational drug design. Interestingly, both compounds displaying high affinity (**43** and **46**) share common structural attributes—cyclobutane‐derivatives bearing aryl‐groups substituted with a non‐polar ester side chain, indicating that this particular sub‐structure might be a potential starting point for further investigation for development of potent sigma‐1 receptor ligands carrying structural diversity. This can be supported by the observation that the azetidine analog **11** showed no activity, and compound **44**, in which the ester bears a polar substituent (i.e., oxetane) has drastically reduced potency. This is in line with the sigma‐1 receptor pharmacophore known to rely on a basic *N*‐atom flanked by two hydrophobic portions [45]. Interestingly and in contrast to the results obtained from the AI‐supported target fishing results, cyclobutyl analog **47** does not show any affinity for the sigma‐1 receptor. In addition, also no in vitro activity for product **8** was observed, despite a high predicted activity.

## 3 Conclusion

We developed a synthetic strategy towards a new class of “super‐strained” spiro heterocycles—spirocyclic azabicyclo[1.1.0]butanes through a Johnson‐Corey‐Chaykovsky reaction between sulfonium salts and azirines. The wide applicability and modularity of this approach was showcased by the synthesis of over 30 derivatives. We studied the stability of this new compound class, and further demonstrate its synthetic value in accessing highly functionalized 1‐azaspiro[3.3]heptanes and their heteroatom‐containing analogs through a range of strain‐release functionalization protocols. In addition, we used an in silico AI‐supported target fishing approach to investigate the potential of this new compound class in medicinal chemistry. We discovered through in vitro experiments that certain derivatives show promising affinity for the sigma‐1 receptor, and further studies into leveraging this discovery are currently underway.

## Author Contributions

**Philipp Natho**: writing – original draft, investigation, methodology, data curation, conceptualization. **Annarita Vicenti**: investigation, methodology. **Marco Colella**: writing – review and editing, methodology, investigation. **Francesco Pasca**: investigation. **Giuseppe Romanazzi**: investigation, writing – review and editing. **Mauro Niso**: investigation, validation. **Carmen Abate**: investigation, validation. **Ernesto Mesto**: investigation, validation. **Emanuela Schingaro**: investigation, validation. **Renzo Luisi**: conceptualization, writing – original draft, writing – review and editing, supervision, funding acquisition, investigation.

## Conflicts of Interest

The authors declare no conflicts of interest.

## Supporting information




**Supporting File 1**: anie73352‐sup‐0001‐SuppMat.pdf.

**Supporting File 2**: anie73352‐sup‐0002‐Data.zip.

**Supporting File 3**: anie73352‐sup‐0003‐Data.zip.

## Data Availability

The data that support the findings of this study are available in the Supporting Information of this article.
