# Supplementary material for: A Novel Class of “Super‐Strained” Spiro Heterocycles: Gateway to 1‐Azaspiro[3.3]heptane Derivatives, and Biological Validation
Source: Angew Chem Int Ed Engl. 2026 Jun 30;65(36):e3488479. doi: 10.1002/anie.3488479 (PMC13528538; doi:10.1002/anie.3488479)

# A Novel Class of “Super-Strained” Spiro Heterocycles: Gateway to 1-Azaspiro[3.3]heptane Derivatives, and Biological Validation

## *Supplementary Information*


Philipp Natho,[ł,a] Annarita Vicenti,[ł,a] Marco Colella,[a] Francesco Pasca,[a] Giuseppe Romanazzi,[b] Mauro Niso,[a] Carmen Abate,[a] Ernesto Mesto,[c] Emanuela Schingaro,[c] Renzo Luisi*[a]

[ł] These authors contributed equally to this work.

[a] Dr. Philipp Natho, Annarita Vicenti, Dr. Marco Colella, Dr. Francesco Pasca, Prof. Mauro Niso, Prof. Carmen Abate, Prof. Renzo Luisi
Department of Pharmacy-Drug Sciences
University of Bari “A. Moro”
Via E. Orabona 4, 70125 Bari, Italy
Renzo.Luisi@uniba.it

[b] Prof. Giuseppe Romanazzi
DICATECh
Politecnico di Bari
Via E. Orabona 4, 70125 Bari, Italy

[c] Prof. Ernesto Mesto, Prof. Emanuela Schingaro
Department of Earth and Geoenvironmental Sciences
University of Bari “A. Moro”
Via E. Orabona 4, 70125 Bari, Italy

## Table of Contents

1. General Information ........................................................................................ S3

2. Optimization Studies ........................................................................................ S5

3. Synthesis of Sulfonium Salts ........................................................................................ S6

4. Azirine collection ........................................................................................ S7

5. General Procedures ........................................................................................ S8

*5.1 General Procedure 1: Synthesis of Azirines from 4-Vinylbenzoic Acid ........................................ S8*

*5.2 General Procedure 2: Synthesis of Azirines by Bromination ........................................ S10*

*5.3 General Procedure 3: Synthesis of Azirines via β-Iodo Azide Intermediate ........................................ S11*

*5.4 General Procedure 4: Synthesis of Azirines through Photocatalysis ........................................ S12*

*5.5 General Procedure 5: Synthesis of Spirocyclic 1-Azaspirobicyclo[1.1.0]butane Derivatives . S14*

*5.6 General Procedure 6: Telescoped 1-Azabicyclo[1.1.0]butane Synthesis/Ring Opening to 1-Azaspiro[3.3]heptane derivatives ........................................ S15*

6. Synthesis & characterization of compounds ........................................ S16

7. Continuous-flow synthesis of 3'-(4-fluorophenyl)-1-tosyl-1'-azaspiro[azetidine-3,2'-bicyclo[1.1.0]butane], **4** ........................................ S116

8. X-Ray Crystallography ........................................ S117

9. Predictive Analytics-Supported Target Fishing ........................................ S120

10. *In vitro* studies ........................................ S122

11. Note on $^{13}$C-Spectra of 1-Azaspiro[3.3]heptane Analogues ........................................ S126

12. References ........................................ S128

13. NMR spectra ........................................ S130

# 1. General Information

### Solvents, Reagents & Reactions

Chemical symbols are employed according to their conventional meaning. All measurements are expressed using SI units and standard abbreviations. Solvent evaporation was performed under reduced pressure using a Büchi R-300 rotary evaporator operating between 0 and 1000 mbar, with the water bath temperature maintained in the range of 35–40 °C. Commercially available reagents obtained from commercial suppliers (Merck, BLD, ThermoFisher Scientific, Fluorochem) were used without additional purification unless otherwise indicated. Lithium bis(trimethylsilyl)amide (1.0 M solution in THF) was obtained from Fluorochem, and anhydrous tetrahydrofuran was obtained from Sigma-Aldrich and used as received.

### Chromatography

Thin-layer chromatography (TLC) was performed on aluminum-backed silica gel plates (0.25 mm, pre-coated with fluorescent indicator 60 F254, Merck) and visualized under UV light ($\lambda$ = 254 nm).
Purification of crude products was carried out by flash column chromatography on silica gel (40–63 µm, Macherey-Nagel), applying pressure through head bellows. In some cases, purification was performed by preparative TLC (0.5 mm, pre-coated with fluorescent indicator 60F254, Sigma-Aldrich). All chromatography solvents were purchased from commercial suppliers and used without further purification.

### Analysis, Spectroscopy and Spectrometry of compounds

Nuclear magnetic resonance (NMR) spectra were acquired on a Bruker Ascend 400 spectrometer operating at frequencies of 400 MHz for $^{1}$H, 101 MHz for $^{13}$C, and 377 MHz for $^{19}$F. Spectra were recorded at room temperature, and chemical shifts were referenced to the residual non-deuterated solvent signal. Chemical shift values (δ) are reported in parts per million (ppm), with precision to two decimal places for $^{1}$H and $^{19}$F spectra and to one decimal place for $^{13}$C spectra. Signal multiplicities are denoted as singlet (s), doublet (d), triplet (t), quartet (q), pentet (p), multiplet (m), broad (br), or appropriate combinations. Coupling constants (J) are provided in Hertz (Hz), rounded to the nearest 0.1 Hz.
Signal assignments for $^{1}$H and $^{13}$C{$^{1}$H} NMR spectra were established through combined evaluation of chemical shift and coupling constant data, with additional support from HSQC and HMBC experiments when necessary.

Infrared spectra were collected using a ThermoScientific Nicolet Summit Pro FTIR instrument. Samples were analysed without solvent, and absorption maxima ($\nu_{max}$) are reported in units of $cm^{-1}$. High-resolution mass spectra were acquired by electrospray ionization (ESI).

**Naming of compounds**

Systematic compound names were generated using ChemDraw Professional 23.1 (PerkinElmer) following IUPAC naming conventions.

## 2. Optimization Studies

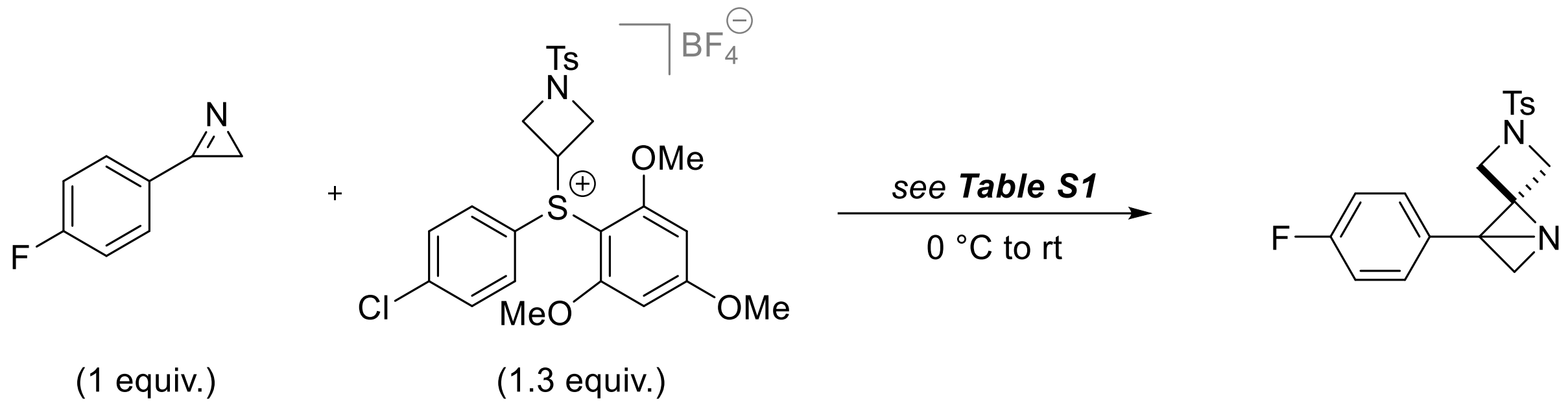


| entry | solvent | base | equiv. base | time of deprotonation [min] | quant. $^1$H-NMR yield (%) |
|---|---|---|---|---|---|
| **1** | THF | LiHMDS | 1.6 | 20 | 0 |
| **2** | THF | LiHMDS | 1.6 | 5 | 28 |
| **3** | $CH_2Cl_2$ | DIPEA | 1.6 | 5 | 0 |
| **4** | THF | DIPEA | 1.6 | internal quenching | 0 |
| **5** | THF | LiHMDS | 1.6 | internal quenching | 73 |
| **6** | THF | KO*t*-BU | 1.6 | internal quenching | 43 |
| **7** | PhMe | LiHMDS | 1.6 | internal quenching | 39 |
| **8** | $Et_2O$ | LiHMDS | 1.6 | internal quenching | 54 |

***Table S1***: Optimization of reaction conditions

## 3. Synthesis of Sulfonium Salts

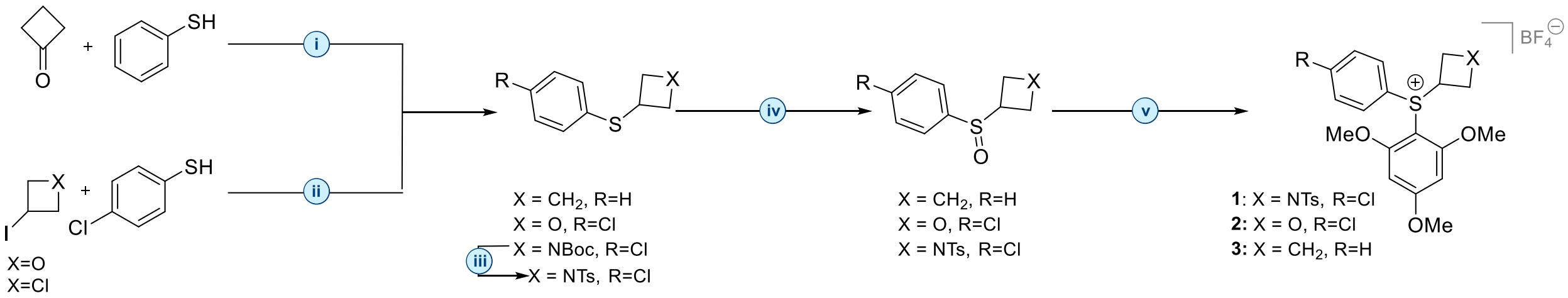


(i) $Et_3SiH$, TFA, $Cl_2Cl_2$, rt, 10 h

(ii) $K_2CO_3$, DMF, 50 °C, 6 h

(iii) TFA, $Cl_2Cl_2$, rt, 1 h, then DMAP, $Et_3N$, TsCl, rt, 12 h

(iv) NBS, MeOH, $H_2O$, 0 °C to rt, 2 h

(v) 1,3,5-Trimethoxybenzene, $Tf_2O$, $Et_2O$, 0 °C, 2 h then 1M $NaBF_{4\ (aq)}$, $CH_2Cl_2$, rt, 1h

The sulfonium salts were prepared according to our previously reported procedure. [1]

# 4. Azirine collection

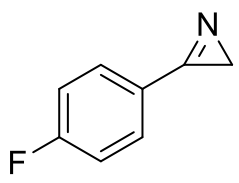
**J**

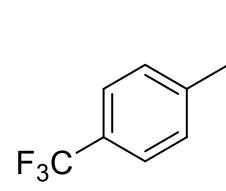
**K**

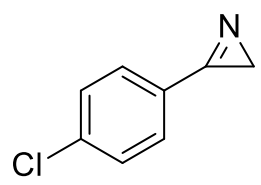
**L**

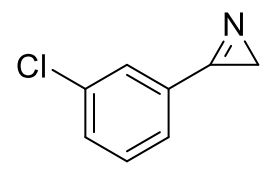
**M**

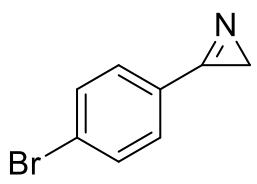
**N**

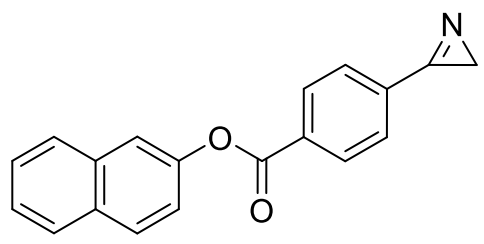
**O**

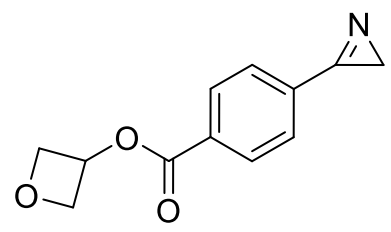
**P**

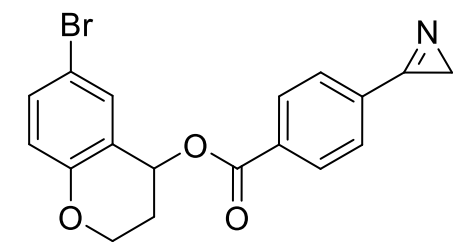
**Q**

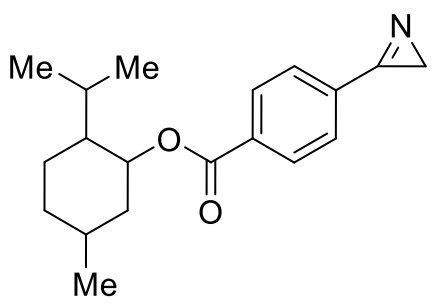
**R**

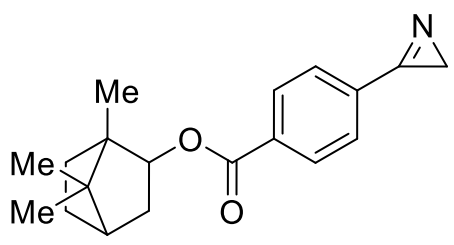
**S**

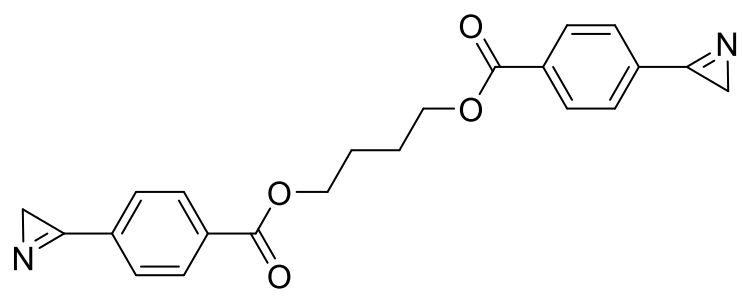
**T**

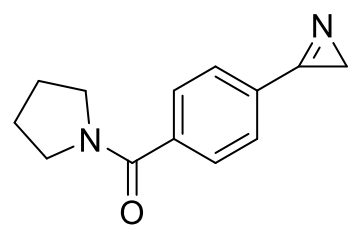
**U**

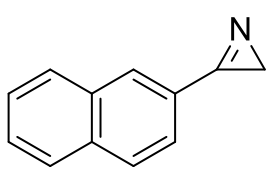
**V**

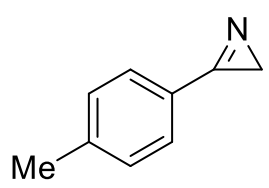
**W**

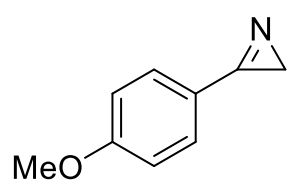
**X**

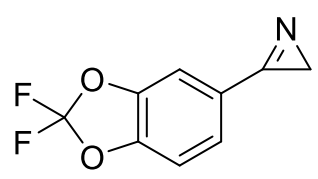
**Y**

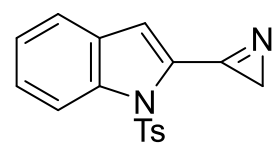
**Z**

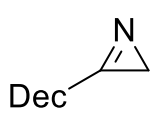
**AA**

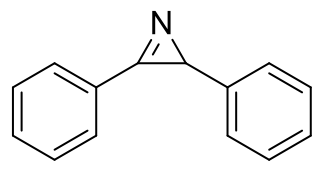
**AB**

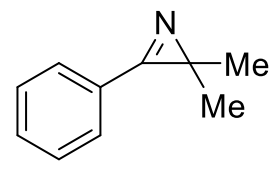
**AC**

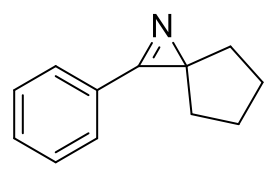
**AD**

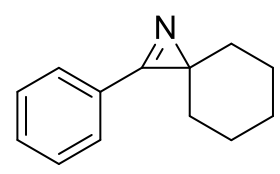
**AE**

See **Synthesis and Characterization of Compounds** for details on azirine synthesis.

Prepared according to reported literature procedures.[2-6, 9]

# 5. General Procedures

## 5.1 General Procedure 1: Synthesis of Azirines from 4-Vinylbenzoic Acid

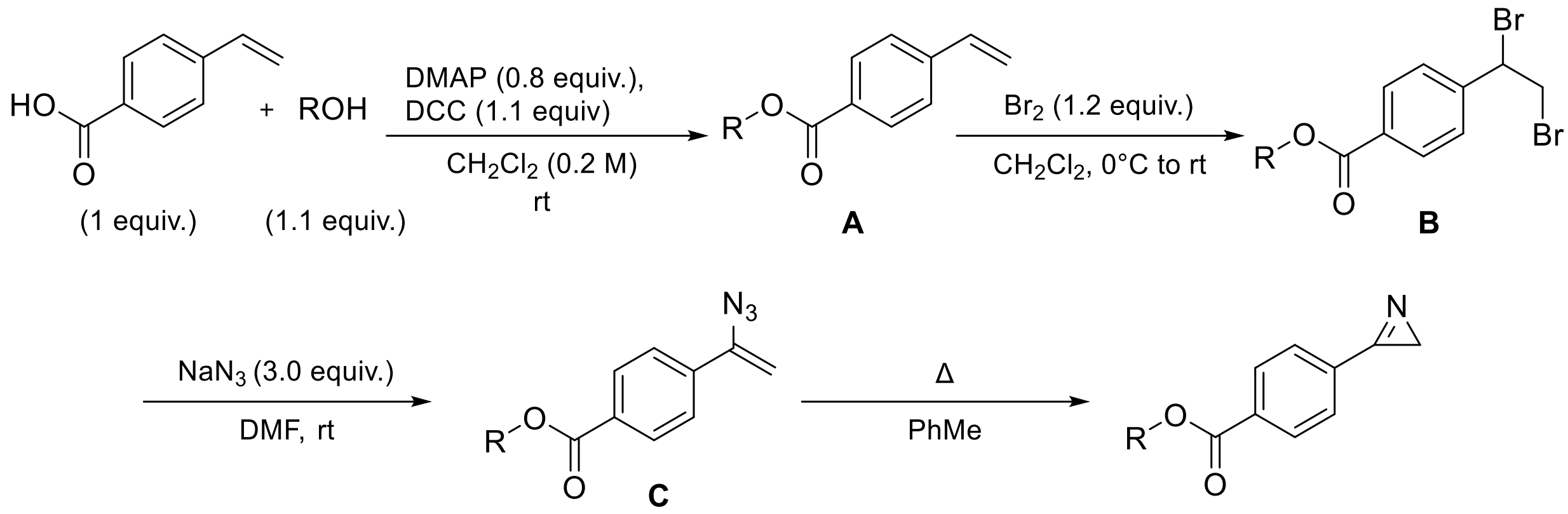


Prepared according to a modified literature procedure. [2]

To a solution of 4-vinylbenzoic acid (1.0 equiv.) and the corresponding alcohol (1.1 equiv.) in dichloromethane (0.2 M) were added 4-dimethylaminopyridine (0.8 equiv.) and *N,N'*-dicyclohexylcarbodiimide (1.1 equiv.). The resulting mixture was stirred at room temperature overnight. Upon completion of the reaction, the mixture was diluted with dichloromethane and the resulting precipitate was removed by filtration. The organic layer was washed successively with saturated aqueous ammonium chloride solution (2 x 20 mL) and saturated aqueous sodium chloride solution (2 x 20 mL). The organic phase was then dried over anhydrous sodium sulfate, filtered, and concentrated under reduced pressure to afford the crude product, which was purified by flash column chromatography, if necessary.

To a solution of the alkene **A** (1.0 equiv.) in dichloromethane (0.2 M) was added bromine (1.2 equiv.) at 0 °C, and the resulting mixture was stirred at room temperature for 1 hour. Upon completion of the reaction, the mixture was diluted with $CH_2Cl_2$ and quenched with aqueous sodium thiosulfate. The organic layer was washed successively with saturated aqueous sodium chloride solution (2 x 20 mL), dried over anhydrous sodium sulfate, filtered, and concentrated under reduced pressure to afford product **B**, which was used in the next step without further purification.

To a solution of product **B** (1.0 equiv.) in DMF (0.2 M) was added sodium azide (3.0 equiv.), and the mixture was stirred at room temperature overnight. The reaction mixture was then poured into water (70 mL per mmol) and extracted with diethyl ether (3 x 50 mL). The combined organic layers were washed

successively with saturated aqueous sodium chloride solution (2 x 20 mL), dried over anhydrous sodium sulfate, filtered, and carefully concentrated under reduced pressure to afford the vinyl azide **C**, which was used immediately in the next step without further purification.

A solution of the vinyl azide **C** in toluene (0.1 M) was heated at reflux for 2 hours. Upon completion of the reaction, the mixture was cooled to room temperature and concentrated under reduced pressure to afford the crude material, which was purified by flash column chromatography.

## 5.2 General Procedure 2: Synthesis of Azirines by Bromination

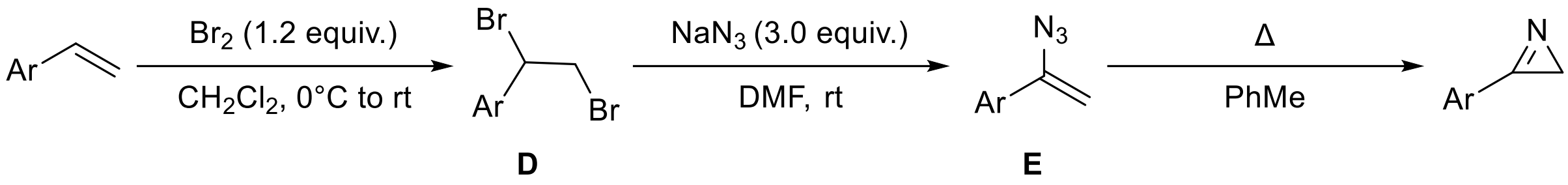

Prepared according to a modified literature procedure. [2]

To a solution of the alkene (1.0 equiv.) in dichloromethane (0.2 M) was added bromine (1.2 equiv.) at 0 °C, and the resulting mixture was stirred at room temperature for 1 hour. Upon completion of the reaction, the mixture was diluted with $CH_2Cl_2$ and quenched with aqueous sodium thiosulfate. The organic layer was washed successively with saturated aqueous sodium chloride solution (2 x 20 mL), dried over anhydrous sodium sulfate, filtered, and concentrated under reduced pressure to afford product **D**, which was used in the next step without further purification.

To a solution of product **D** (1.0 equiv.) in DMF (0.2 M) was added sodium azide (3.0 equiv.), and the mixture was stirred at room temperature overnight. The reaction mixture was poured into water (70 mL) and extracted with diethyl ether (3 x 50 mL). The combined organic layers were washed successively with saturated aqueous sodium chloride solution (2 x 20 mL), dried over anhydrous sodium sulfate, filtered, and concentrated under reduced pressure to afford the vinyl azide **E**, which was used immediately in the next step without further purification.

A solution of the vinyl azide **E** in toluene (0.1 M) was heated at reflux for 2 hours. Upon completion of the reaction, the mixture was cooled to room temperature and concentrated under reduced pressure to afford the crude material, which was purified by flash column chromatography.

## 5.3 General Procedure 3: Synthesis of Azirines via β-Iodo Azide Intermediate

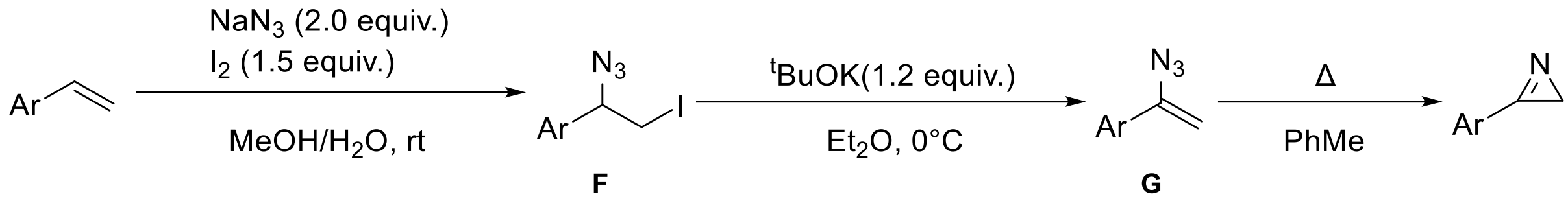


Prepared according to a modified literature procedure.[3]

To a solution of the alkene (1.0 equiv.) in MeOH/$H_2O$ (5:1, 0.2 M), sodium azide (2.0 equiv.) was added in one portion at 25 °C, followed by iodine (1.5 equiv.). The resulting mixture was stirred at room temperature for 3 h. After completion, $CH_2Cl_2$ (90 mL) and $H_2O$ (50 mL) were added, and the organic layer was separated and washed with an aqueous solution of sodium thiosulfate. The organic layer was then washed twice with water (2 x 35 mL), dried over anhydrous sodium sulfate and the solvent removed under reduced pressure. The resulting product **F** was used directly in the next step without further purification.

A solution of the β-iodo azide **F** in $Et_2O$ (0.2 M) was treated with potassium *tert*-butoxide (1.2 equiv.) at 0 °C, and the mixture was stirred at the same temperature for 1.5 h. The reaction mixture was then filtered through a pad of Celite, and the solvent removed under reduced pressure to afford the vinyl azide, which was used immediately in the subsequent step without purification.

A solution of the vinyl azide **G** in toluene (0.1 M) was heated at reflux for 2 h. Upon completion, the reaction mixture was cooled to room temperature, concentrated under reduced pressure, and the crude product purified by flash column chromatography.

## 5.4 General Procedure 4: Synthesis of Azirines through Photocatalysis

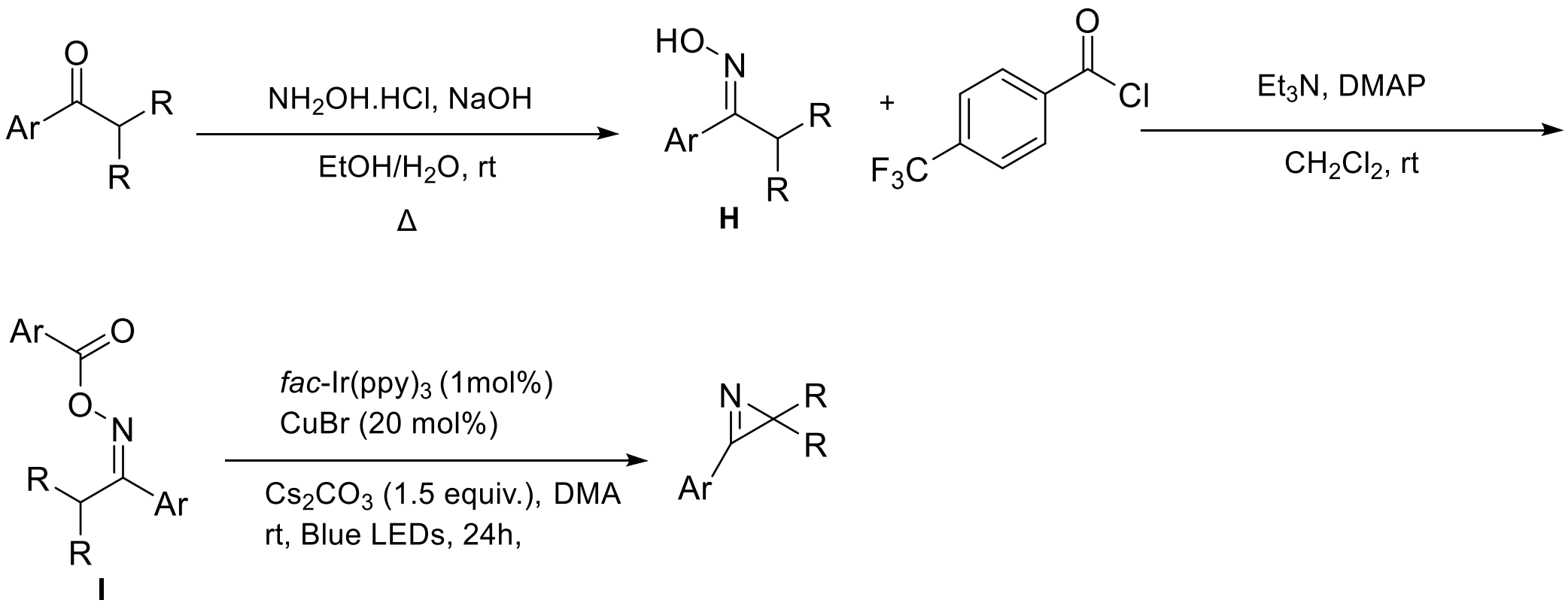


Prepared according to a modified literature procedure.[4,5]

To a stirred solution of the ketone (1.0 equiv.) in ethanol/$H_2O$ (1:2 v/v, 0.2 M) were added hydroxylamine hydrochloride (1.5 equiv.) and sodium hydroxide (5 equiv.). The resulting reaction mixture was heated at 95 °C for 6 h. After completion of the reaction, the reaction mixture was cooled to room temperature, and added conc. hydrochloric acid (0.55 mL/mmol), and deionized water (4 mL/mmol). The precipitate was filtered off, and recrystallized from EtOH affording the corresponding oxime **H**, which was used in the next step.

To a stirred solution of the oxime (1.0 equiv.), triethylamine (1.5 equiv.), and 4-dimethylaminopyridine (0.1 equiv.) in dichloromethane (0.3 M) was added 4-(trifluoromethyl)benzoyl chloride (1.2 equiv.). The reaction mixture was stirred at room temperature for 5.5 h. After completion of the reaction, the mixture was diluted with dichloromethane and washed successively with water, saturated aqueous ammonium chloride solution (2 x 30 mL), and saturated aqueous sodium chloride solution (2 x 30 mL). The organic layer was dried over anhydrous sodium sulfate, filtered, and concentrated under reduced pressure to afford the product **I**, which was used without further purification.

In an oven-dried reaction tube equipped with a magnetic stir bar were placed urea substrate **I** (1.0 equiv.), caesium carbonate (1.5 equiv.), copper(I) bromide (20 mol%), and *fac*-Ir(ppy)$_3$ (1 mol%). The tube evacuated, and refilled with argon (three cycles). *N,N*-Dimethylacetamide (30 mL) was subsequently introduced under an inert atmosphere. The reaction mixture was stirred at room temperature and exposed to 5 W blue LED irradiation for 12 h. Upon completion, the mixture was poured into cold water and diluted with ethyl acetate. The suspension was filtered through a Celite pad to remove inorganic residues. The organic layer was washed with saturated aqueous sodium chloride solution and

dried over anhydrous sodium sulfate, filtered, and concentrated under reduced pressure to afford the crude product, which was purified by flash column chromatography.

## 5.5 General Procedure 5: Synthesis of Spirocyclic 1-Azaspirobicyclo[1.1.0]butane Derivatives

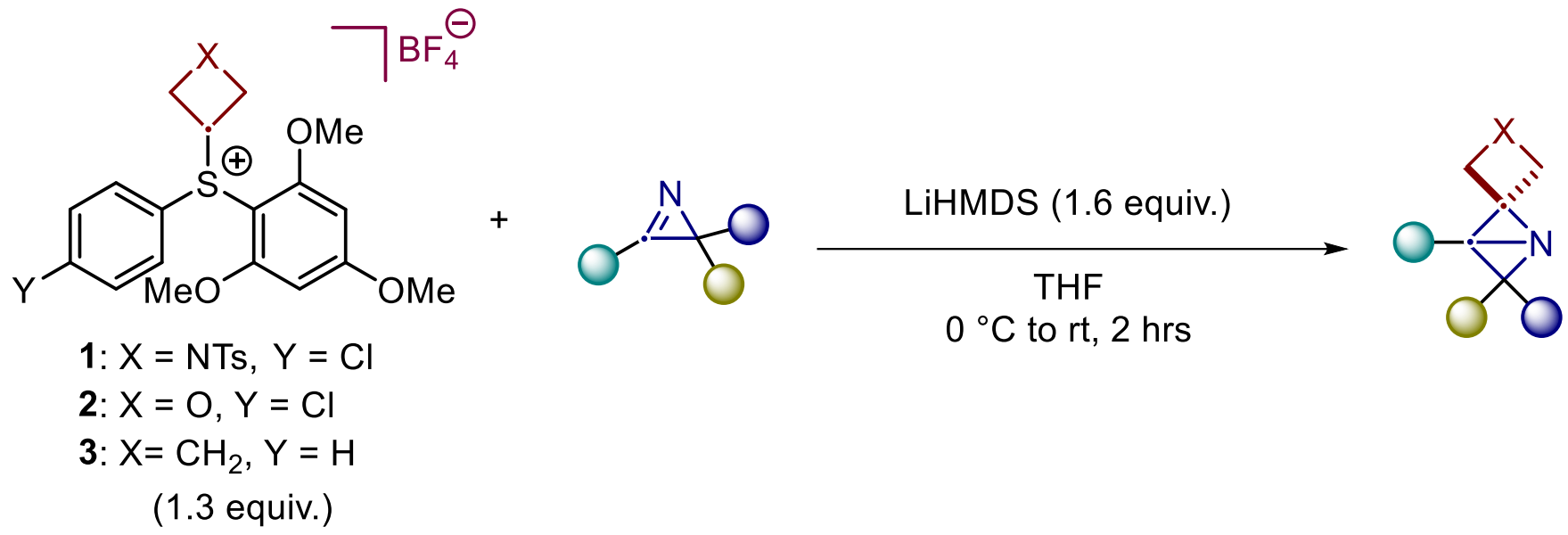


To a solution of azirine (1 equiv.) and the sulfonium salt (**1** or **2** or **3**) (1.3 equiv.) in THF (0.03 M) at 0 °C was added a solution of LiHMDS (1.0 M in THF, 1.6 equiv.) dropwise. The reaction mixture was stirred at 0 °C for 10 minutes, then allowed to warm to the room temperature and stirred for 2 hours. The reaction was quenched by the addition of methanol (1 mL per 1 mmol) and concentrated under reduced pressure to afford the crude material, which was purified by flash column chromatography as indicated in each entry.

*Note: Particular caution is advised during the isolation of the 1-azaspirobicyclo[1.1.0]butane derivatives by column chromatography, since:*

*a) Many products cannot or can only poorly be visualized by UV light or $KMnO_4$ solution or iodine stain; however, some can be detected using an ethanolic phosphomolybdic acid solution;*

*b) Many 1-azaspirobicyclo[1.1.0]butane derivatives have been found to have similar polarity compared to the sulfide by-product;*

*c) Some decomposition (especially for cyclobutane- and oxetane-derivatives) was observed during purification on silica gel and could only occasionally be suppressed by pre-treatment pre-treatment with triethylamine.*

## 5.6 General Procedure 6: Telescoped 1-Azabicyclo[1.1.0]butane Synthesis/Ring Opening to 1-Azaspiro[3.3]heptane derivatives

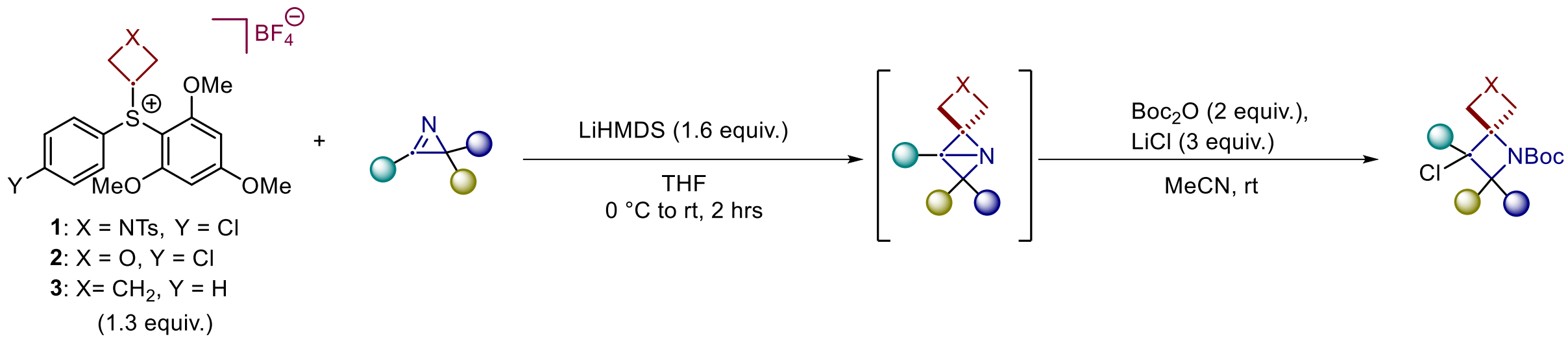


To a solution of azirine (1.0 equiv.) and the sulfonium salt (**1** or **2** or **3**) (1.3 equiv.) in THF (0.03 M) at 0 °C was added dropwise a solution of LiHMDS (1.0 M in THF, 1.6 equiv.). The reaction mixture was stirred at 0 °C for 10 min, then allowed to warm to room temperature and stirred for 2 h. The reaction was quenched by the addition of methanol (1 mL per 1 mmol) and concentrated under reduced pressure. The resulting crude material was dissolved in acetonitrile (0.03 M), di-*tert*-butyl dicarbonate (2.0 equiv.) and lithium chloride (3.0 equiv.) were added, and the resulting mixture was stirred at room temperature for 12 h. Upon completion, the reaction mixture was concentrated under reduced pressure to afford the crude product, which was purified by flash column chromatography.

## 6. Synthesis & characterization of compounds

### 3-(4-Fluorophenyl)-2*H*-azirine, J

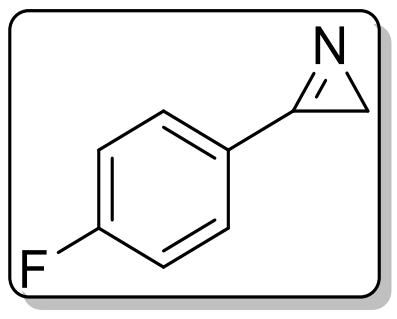

Prepared following **General Procedure 2** from 4-fluorostyrene (610 mg, 5.00 mmol). Purification by flash column chromatography (5% $Et_2O$/hexane) affords the title compound **J** (351 mg, 2.6 mmol, 52% over 3 steps) as a yellow oil.

**$^1$H-NMR** (400 MHz, $CDCl_3$): $\delta_H$ 7.95 – 7.89 (2H, m), 7.27 – 7.24 (2H, m), 1.80 (2H, s).

**$^{19}$F-NMR** (377 MHz, $CDCl_3$): $\delta_F$ -104.77 (m).

**$^{13}$C{$^1$H}-NMR** (101 MHz, $CDCl_3$): $\delta_C$ 165.5 (d, *J* = 254.7 Hz), 164.7, 132.0 (d, *J* = 9.5 Hz), 122.0 (d, *J* = 3.0 Hz), 116.5 (d, *J* = 22.3 Hz), 19.8.

*The spectroscopic data are in agreement with those previously reported.*[6]

**3-(4-(Trifluoromethyl)phenyl)-2*H*-azirine, K**

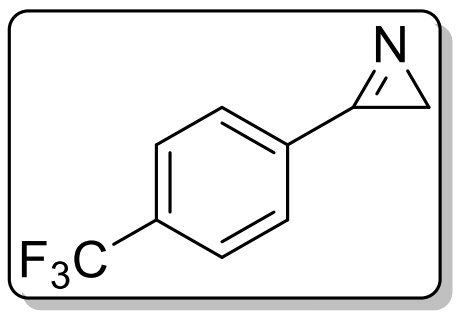


Prepared following **General Procedure 2** from 1-(trifluoromethyl)-4-vinylbenzene (864 mg, 5.00 mmol). Purification by flash column chromatography (5% $Et_2O$/hexane) affords the title compound **K** (350 mg, 1.89 mmol, 38% over 3 steps) as a yellow oil.

**$^1$H-NMR** (400 MHz, $CDCl_3$): $\delta_H$ 8.12 – 7.95 (2H, m), 7.90 – 7.71 (2H, m), 1.87 (2H, s).

**$^{19}$F-NMR** (377 MHz, $CDCl_3$): $\delta_F$ -63.15 (s).

**$^{13}$C{$^1$H}-NMR** (101 MHz, $CDCl_3$): $\delta_C$ 165.8, 134.6 (d, *J* = 33.0 Hz), 129.9, 128.9, 126.3 (q, *J* = 3.8 Hz), 123.7 (d, *J* = 272.7 Hz), 20.4.

*The spectroscopic data are in agreement with those previously reported.*[2]

**3-(4-Chlorophenyl)-2*H*-azirine, L**

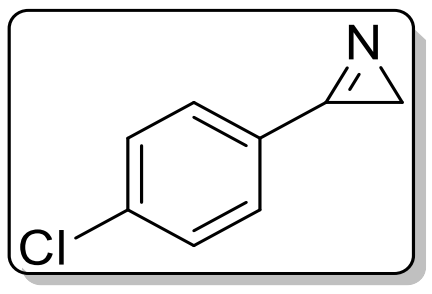


Prepared following **General Procedure 2** from 1-chloro-4-vinylbenzene (693 mg, 5.00 mmol). Purification by flash column chromatography (10% $Et_2O$/hexane) affords the title compound **L** (400 mg, 2.65 mmol, 53% over 3 steps) as a yellow solid.

**$^1$H-NMR** (400 MHz, $CDCl_3$): $\delta_H$ 7.85 (2H, d, *J* = 8.5 Hz), 7.55 (2H, d, *J* = 8.5 Hz), 1.81 (2H, s).

**$^{13}$C{$^1$H}-NMR** (101 MHz, $CDCl_3$): $\delta_C$ 165.3, 139.4, 130.9, 129.7, 124.2, 20.1.

*The spectroscopic data are in agreement with those previously reported.[3]*

**3-(4-Bromophenyl)-2*H*-azirine, N**

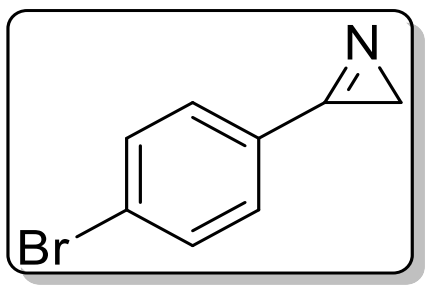


Prepared following **General Procedure 2** from 1-bromo-4-vinylbenzene (2.00 g, 10.9 mmol). Purification by flash column chromatography (8% $Et_2O$/hexane) affords the title compound **N** (750 mg, 3.83 mmol, 35% over 3 steps) as an orange solid.

**$^1$H-NMR** (400 MHz, $CDCl_3$): $\delta_H$ 7.76 (2H, d, *J* = 8.5 Hz), 7.70 (2H, d, *J* = 8.5 Hz), 1.79 (2H, s).

**$^{13}$C{$^1$H}-NMR** (101 MHz, $CDCl_3$): $\delta_C$ 165.4, 132.6, 131.0, 127.9, 124.6, 20.1.

*The spectroscopic data are in agreement with those previously reported.*[3]

**Naphthalen-2-yl 4-(2*H*-azirin-3-yl)benzoate, O**

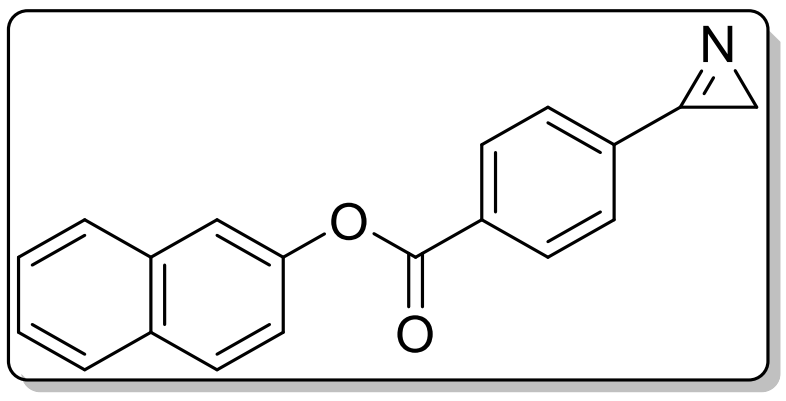

Prepared following **General Procedure 1** from 4-vinylbenzoic acid (1.50 g, 10.1 mmol) and naphthalen-2-ol (1.60 g, 11.1 mmol). Purification by flash column chromatography (5-15% EtOAc/hexane) affords the title compound **O** (250 mg, 0.87 mmol, 9% over 4 steps) as a yellow solid.

**$^{1}$H-NMR** (400 MHz, $CDCl_3$): $\delta_H$ 8.50 – 8.39 (2H, m), 8.12 – 8.02 (2H, m), 7.93 (1H, d, *J* = 8.8 Hz), 7.90 – 7.81 (2H, m), 7.73 (1H, d, *J* = 2.4 Hz), 7.56 – 7.50 (2H, m), 7.39 (1H, dd, *J* = 8.9, 2.4 Hz), 1.91 (2H, s).

**$^{13}$C{$^{1}$H}-NMR** (101 MHz, $CDCl_3$): $\delta_C$ 166.1, 164.5, 148.5, 133.9, 133.5, 131.8, 131.0, 130.0, 129.8, 129.7, 128.0, 127.9, 126.9, 126.1, 121.1, 118.8, 20.5.

**IR** (neat, *v* cm$^{-1}$): 2984, 1731, 1237, 1158, 1063.

**HRMS** (ESI+): *m/z* calcd for $C_{19}H_{13}NO_2Na$ $[M+Na]^+$ 310.0838, found 310.0835

**Oxetan-3-yl 4-(2*H*-azirin-3-yl)benzoate, P**

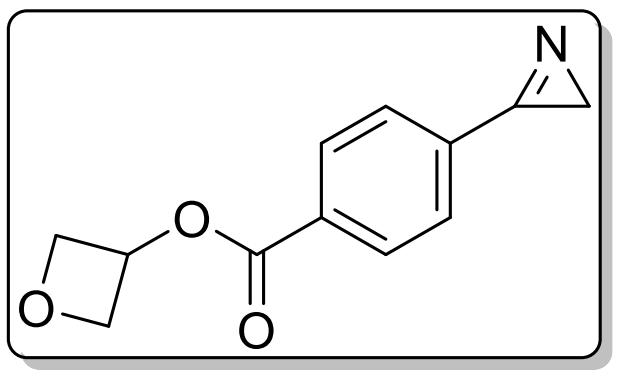

Prepared following **General Procedure 1** from 4-vinylbenzoic acid (1.50 g, 10.1 mmol) and oxetan-3-ol (820 mg, 11.1 mmol). Purification by flash column chromatography (5-20% EtOAc/hexane) affords the title compound **P** (940 mg, 4.33 mmol, 43% over 4 steps) as an orange solid.

**$^1$H-NMR** (400 MHz, $CDCl_3$): $\delta_H$ 8.25 (2H, d, *J* = 8.3 Hz), 8.00 (2H, d, *J* = 8.3 Hz), 5.82 – 5.60 (1H, m), 5.02 (2H, m), 4.81 (2H, m), 1.87 (2H, s).

**$^{13}$C{$^1$H}-NMR** (101 MHz, $CDCl_3$): $\delta_C$ 166.0, 164.9, 133.1, 130.5, 129.9, 129.6, 77.6, 69.0, 20.5.

**IR** (neat, *v* cm$^{-1}$): 2953, 2880, 1719, 1269, 1126.

**HRMS** (ESI+): *m/z* calcd for $C_{12}H_{11}NO_3Na$ $[M+Na]^+$ 240.0631, found 240.0624.

**6-Bromochroman-4-yl 4-(2*H*-azirin-3-yl)benzoate, Q**

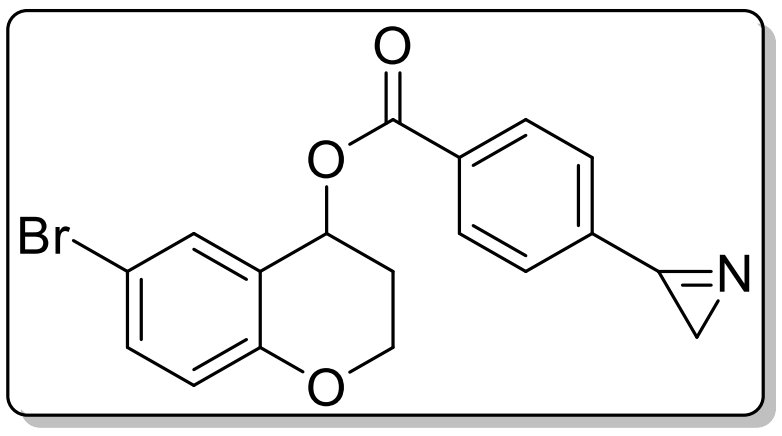


Prepared following **General Procedure 1** from 4-vinylbenzoic acid (1.5 g, 10.1 mmol) and chroman-4-ol (1.66 g, 11.1 mmol). Purification by flash column chromatography (5-10% EtOAc/hexane) affords the title compound **Q** (200 mg, 0.54 mmol, 5.5% over 4 steps) as a yellow solid.

*Note: Concomitant bromination of the aromatic ring was observed during the bromination of the styrene.*

**$^1$H-NMR** (400 MHz, $CDCl_3$): $\delta_H$ 8.25 – 8.18 (2H, m), 8.05 – 7.94 (2H, m), 7.51 (1H, d, *J* = 2.8 Hz), 7.35 (1H, dd, *J* = 8.8, 2.5 Hz), 6.80 (1H, d, *J* = 8.8 Hz), 6.18 (1H, t, *J* = 3.9 Hz), 4.47 – 4.22 (2H, m), 2.34 – 2.26 (2H, m), 1.86 (2H, s).

**$^{13}$C{$^1$H}-NMR** (101 MHz, $CDCl_3$): $\delta_C$ 165.7, 165.0, 156.6, 154.7, 133.8, 133.5, 133.3, 130.5, 129.6, 121.8, 119.3, 112.7, 66.1, 62.5, 28.3, 20.5.

**IR** (neat, *v* $cm^{-1}$): 2971, 1713, 1483, 1263, 1039.

**HRMS** (ESI+): *m/z* calcd for $C_{18}H_{14}{}^{79}BrNO_3Na$ $[M+Na]^+$ 394.0049, found 394.0036.

**2-Isopropyl-5-methylcyclohexyl 4-(2*H*-azirin-3-yl)benzoate, R**

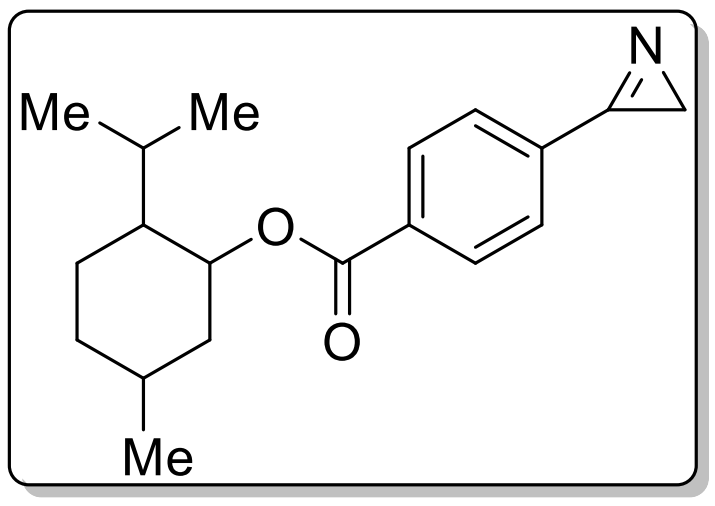


Prepared following **General Procedure 1** from 4-vinylbenzoic acid (330 mg, 2.25 mmol) and menthol (385 mg, 2.47 mmol). Purification by flash column chromatography (8% EtOAc/hexane) affords the title compound **R** (130 mg, 0.43 mmol, 17% over 4 steps) as a colorless oil.

**$^1$H-NMR** (400 MHz, $CDCl_3$): $\delta_H$ 8.22 (2H, d, *J* = 8.3 Hz), 8.00 – 7.95 (2H, m), 4.97 (1H, td, *J* = 10.9, 4.4 Hz), 2.19 – 2.10 (1H, m), 1.95 (1H, td, *J* = 7.0, 2.8 Hz), 1.85 (2H, s), 1.75 (2H, dt, *J* = 14.9, 4.0 Hz), 1.62 – 1.53 (3H, m), 1.19 – 1.08 (2H, m), 0.94 (6H, dd, *J* = 6.8, 5.2 Hz), 0.81 (3H, d, *J* = 6.9 Hz).

**$^{13}$C{$^1$H}-NMR** (101 MHz, $CDCl_3$): $\delta_C$ 165.9, 165.1, 134.7, 130.2, 129.4, 129.1, 75.7, 47.3, 41.0, 34.4, 31.5, 26.7, 23.7, 22.1, 20.8, 20.3, 16.6.

**IR** (neat, *v* $cm^{-1}$): 2954, 2868, 1713, 1285, 1266

**HRMS** (ESI+): *m/z* calcd for $C_{19}H_{25}NO_2Na$ $[M+Na]^+$ 322.1778, found 322.1795.

**1,7,7-Trimethylbicyclo[2.2.1]heptan-2-yl 4-(2*H*-azirin-3-yl)benzoate, S**

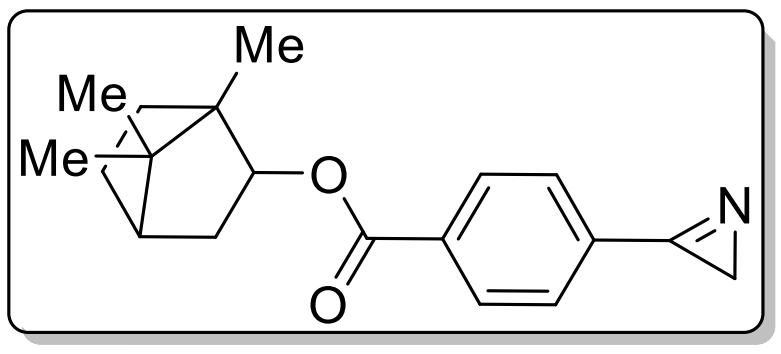


Prepared following **General Procedure 1** from 4-vinylbenzoic acid (629 mg, 4.25 mmol) and borneol (721 mg, 4.68 mmol). Purification by flash column chromatography (5-7.5% $Et_2O$/hexane) affords the title compound **S** (227 mg, 0.76 mmol, 18% over 4 steps) as a white solid.

**$^1$H-NMR** (400 MHz, $CDCl_3$): $\delta_H$ 8.23 (2H, d, *J* = 8.4 Hz), 7.99 (2H, d, *J* = 8.4 Hz), 5.16 (1H, ddd, *J* = 9.9, 3.5, 2.2 Hz), 2.59 – 2.38 (1H, m), 2.11 (1H, m), 1.86 (2H, s), 1.84 – 1.72 (2H, m), 1.53 – 1.39 (1H, m), 1.38 – 1.29 (1H, m), 1.15 (1H, dd, *J* = 13.8, 3.5 Hz), 0.98 (3H, s), 0.93 (6H, s).

**$^{13}$C{$^1$H}-NMR** (101 MHz, $CDCl_3$): $\delta_C$ 166.0, 166.0, 134.8, 130.3, 129.6, 129.2, 81.5, 49.3, 48.1, 45.1, 37.0, 28.2, 27.6, 20.4, 19.9, 19.1, 13.8.

**IR** (neat, *v* cm$^{-1}$): 2955, 1711, 1307, 1270, 1104.

**HRMS** (ESI+): *m/z* calcd for $C_{19}H_{23}NO_2Na$ $[M+Na]^+$ 320.1621, found 320.1631.

**Butane-1,4-diyl bis(4-(2*H*-azirin-3-yl)benzoate), T**

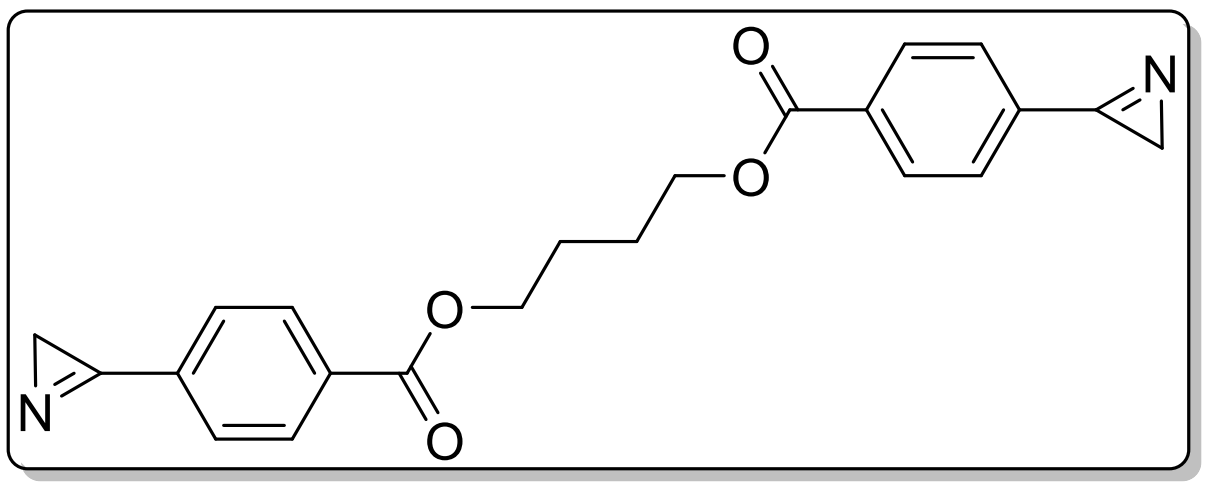

Prepared following **General Procedure 1** from 4-vinylbenzoic acid (3.00 g, 20.2 mmol) and butane-1,4-diol (912 mg, 10.1 mmol). Purification by flash column chromatography (10-40% EtOAc/hexane) affords the title compound **T** (750 mg, 1.99 mmol, 10% over 4 steps) as a yellow solid.

**$^{1}$H-NMR** (400 MHz, $CDCl_3$): $\delta_H$ 8.25 – 8.19 (4H, m), 7.99 – 7.95 (4H, m), 4.55 – 4.34 (4H, m), 2.02 – 1.97 (4H, m), 1.85 (4H, d, *J* = 0.9 Hz).

**$^{13}$C{$^{1}$H}-NMR** (101 MHz, $CDCl_3$): $\delta_C$ 166.0, 165.7, 134.1, 130.3, 129.6, 127.4, 65.1, 25.64, 20.4.

**IR** (neat, $v$ $cm^{-1}$): 2931, 2851, 1717, 1647, 1275.

**HRMS** (ESI+): *m/z* calcd for $C_{22}H_{20}N_2O_4Na$ $[M+Na]^+$ 399.1315, found 399.1320.

**(4-(2*H*-Azirin-3-yl)phenyl)(pyrrolidin-1-yl)methanone, U**

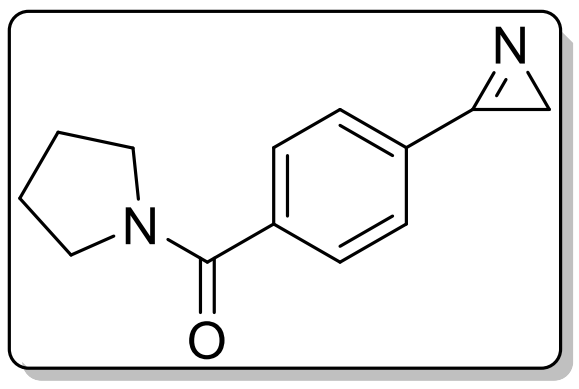

Prepared following **General Procedure 2** from pyrrolidin-1-yl(4-vinylphenyl)methanone (3.30 g, 16.00 mmol). Purification by flash column chromatography (10% EtOAc/hexane) affords the title compound **U** (320 mg, 1.49 mmol, 9% over 3 steps) as a yellow oil.

**$^1$H-NMR** (400 MHz, $CDCl_3$): $\delta_H$ 7.94 (2H, d, *J* = 8.1 Hz), 7.71 – 7.67 (2H, m), 3.67 (2H, t, *J* = 7.1 Hz), 3.42 (2H, t, *J* = 6.6 Hz), 2.01 – 1.96 (2H, m), 1.91 (2H, m), 1.82 (2H, s).

**$^{13}$C{$^1$H}-NMR** (101 MHz, $CDCl_3$): $\delta_C$ 168.6, 165.8, 141.6, 129.7, 127.9, 126.7, 49.6, 46.4, 26.5, 24.6, 20.1.

**IR** (neat, $\nu$ cm$^{-1}$): 3332, 1624, 1585, 854, 792

**HRMS** (ESI+): *m/z* calcd for $C_{13}H_{14}N_2ONa$ $[M+Na]^+$ 237.0998, found 237.1008.

**3-(Naphthalen-2-yl)-2*H*-azirine, V**

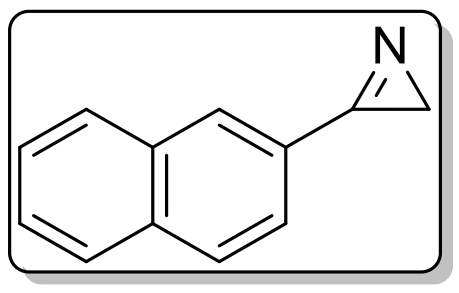


Prepared following **General Procedure 2** from 2-vinylnaphthalene (770 mg, 5.00 mmol). Purification by flash column chromatography (5% $Et_2O$/hexane) affords the title compound **V** (490 mg, 2.93 mmol, 59% over 3 steps) as a yellow oil.

**$^1$H-NMR** (400 MHz, $CDCl_3$): $\delta_H$ 8.35 (1H, d, *J* = 1.2 Hz), 8.01 – 7.96 (3H, m), 7.96 – 7.89 (1H, m), 7.69 – 7.53 (2H, m), 1.89 (2H, s).

**$^{13}$C{$^1$H}-NMR** (101 MHz, $CDCl_3$): $\delta_C$ 166.0, 135.6, 133.0, 132.0, 129.2, 128.6, 128.2, 127.2, 124.5, 123.1, 20.1.

*The spectroscopic data are in agreement with those previously reported.*[3]

**3-(*p*-tolyl)-2*H*-azirine, W**

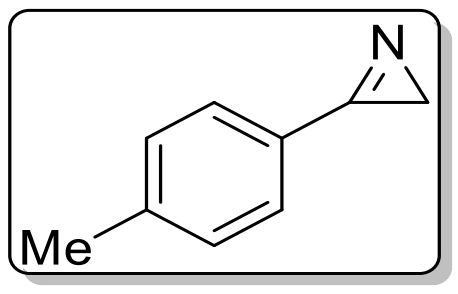


Prepared following **General Procedure 2** from 1-methyl-4-vinylbenzene (591 mg, 5.00 mmol). Purification by flash column chromatography (8% $Et_2O$/hexane) affords the title compound **W** (160 mg, 1.22 mmol, 25% over 3 steps) as a yellow oil.

**$^1$H-NMR** (400 MHz, $CDCl_3$): $\delta_H$ 7.80 (2H, d, *J* = 8.1 Hz), 7.36 (2H, m), 2.46 (3H, s), 1.76 (2H, s).

**$^{13}$C{$^1$H}-NMR** (101 MHz, $CDCl_3$): $\delta_C$ 165.4, 143.9, 129.9, 129.8, 123.0, 22.0, 19.6.

*The spectroscopic data are in agreement with those previously reported.*[3]

**3-(2,2-Difluorobenzo[*d*][1,3]dioxol-5-yl)-2*H*-azirine, Y**

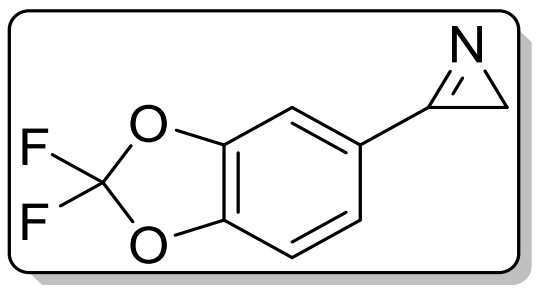


Prepared following **General Procedure 2** from 2,2-difluoro-5-vinylbenzo[*d*][1,3]dioxole (980 mg, 5.32 mmol). Purification by flash column chromatography (8% $Et_2O$/hexane) affords the title compound **Y** (370 mg, 1.87 mmol, 35% over 3 steps) as a white solid.

**$^1$H-NMR** (400 MHz, $CDCl_3$): $\delta_H$ 7.68 (1H, dd, *J* = 8.2, 1.5 Hz), 7.63 (1H, d, *J* = 1.5 Hz), 7.31 – 7.13 (1H, d, *J* = 8.2 Hz), 1.83 (2H, s).

**$^{19}$F-NMR** (377 MHz, $CDCl_3$): $\delta_F$ -49.74 (s).

**$^{13}$C{$^1$H}-NMR** (101 MHz, $CDCl_3$): $\delta_C$ 165.2, 147.0, 144.5, 131.8 (t, *J* = 258.0 Hz), 127.0, 122.1, 110.3, 110.0, 20.6.

**IR** (neat, *v* $cm^{-1}$): 3052, 1747, 1497, 1217, 1149.

**HRMS** (ESI+): *m/z* calcd for $C_9H_6F_2NO_2$ $[M+H]^+$ 198.0361, found 198.0344.

**2-(2*H*-Azirin-3-yl)-1-tosyl-1*H*-indole, Z**

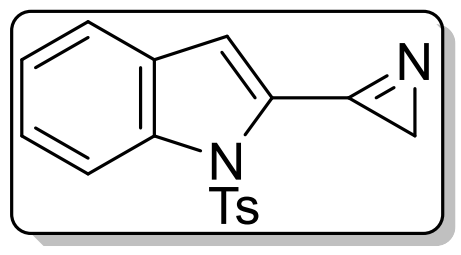


To a solution of sodium azide (520 mg, 8.00 mmol, 3 equiv.) in acetonitrile (1 mL) was added a solution of iodine monochloride (648 mg, 4.00 mmol, 1.5 equiv.) in $CH_2Cl_2$ (15 mL) at -20 °C. The reaction mixture was stirred at the same temperature for 30 min, then a solution of 1-tosyl-2-vinyl-1*H*-indole (790 mg, 2.70 mmol, 1.0 equiv.) in $CH_2Cl_2$ (5 mL) was added dropwise, and the resulting mixture was stirred for 1 h. The reaction was quenched with aqueous sodium thiosulfate, and the aqueous phase was extracted with diethyl ether (3 x 10 mL). The organic phase was then dried over anhydrous sodium sulfate, filtered, and concentrated under reduced pressure to afford the crude product which was used in the next step without further purification. To a solution of the crude material in diethyl ether (30 mL) was added potassium *tert*-butoxide (360 mg, 3.20 mmol, 1.2 equiv.) at 0 °C, and the reaction was stirred for 1.5 h at the same temperature. The reaction was quenched with saturated aqueous ammonium chloride solution, and the organic materials was extracted with diethyl ether (2 x 10 mL) and dried over anhydrous sodium sulfate, filtered, and then concentrated under reduced pressure to afford the crude product which was used in the next step without further purification. A solution of 2-(1-azidovinyl)-1-tosyl-1*H*-indole in toluene (0.1 M) was heated at reflux for 2 h. Upon completion of the reaction, the mixture was cooled to room temperature and concentrated under reduced pressure to afford the crude material, which was purified by flash column chromatography (5-10% EtOAc/hexane) affords the title compound **Z** (120 mg, 0.39 mmol, 15% over 3 steps) as a white solid.

**$^1$H-NMR** (400 MHz, $CDCl_3$): $\delta_H$ 8.36 (1H m), 7.85 (2H, d, *J* = 8.4 Hz), 7.66 (1H, d, *J* = 1.1 Hz), 7.57 – 7.50 (1H, m), 7.43 – 7.31 (2H, m), 7.21 (2H, d, *J* = 8.2 Hz), 2.34 (3H, s), 1.89, (2H, s).

**$^{13}$C{$^1$H}-NMR** (101 MHz, $CDCl_3$): $\delta_C$ 158.1, 145.6, 139.4, 135.5, 130.0, 128.4, 128.2, 127.2, 126.3, 124.4, 122.9, 120.9, 115.1, 21.7, 21.7.

**IR** (neat, *v* cm$^{-1}$): 2976, 1743, 1374, 1307, 1172

**HRMS** (ESI+): *m/z* calcd for $C_{17}H_{14}N_2O_2SNa$ [M+Na]$^+$ 333.0668, found 333.0686.

**3-Decyl-2*H*-azirine, AA**

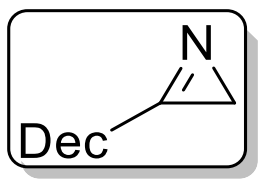


Prepared following **General Procedure 3** from dodec-1-ene (2.36 g, 14.0 mmol). Purification by flash column chromatography (5% $Et_2O$/hexane) affords the title compound **AA** (580 mg, 3.20 mmol, 23% over 3 steps) as a colourless oil.

**$^1$H-NMR** (400 MHz, $CDCl_3$): $\delta_H$ 2.78 (2H, t, $J$ = 7.3 Hz), 1.74 (2H, p, $J$ = 7.3 Hz), 1.45 – 1.20 (16H, m), 0.92 – 0.85 (3H, m).

**$^{13}$C{$^1$H}-NMR** (101 MHz, $CDCl_3$): $\delta_C$ 169.9, 32.0, 29.7, 29.6, 29.4, 29.4, 28.6, 24.3, 22.8, 18.9, 14.2.

*The spectroscopic data are in agreement with those previously reported.*[3]

**2,3-Diphenyl-2*H*-azirine, AB**

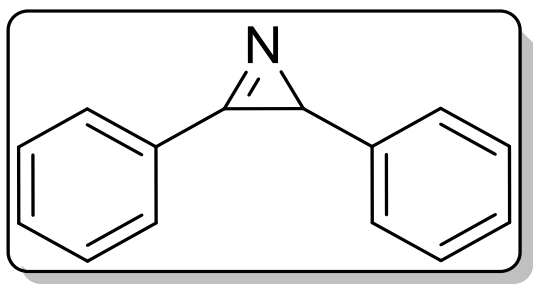


Prepared following **General Procedure 3** from 1,2-diphenylethene (2.52 g, 14.0 mmol). Purification by flash column chromatography (5% $Et_2O$/hexane) affords the title compound **AB** (720 mg, 3.73 mmol, 26% over 3 steps) as a white solid.

**$^1$H-NMR** (400 MHz, $CDCl_3$): $\delta_H$ 7.95 – 7.88 (2H, m), 7.63 – 7.54 (3H, m), 7.31 – 7.24 (3H, m), 7.21 – 7.15 (2H, m), 3.34 (1H, s).

**$^{13}$C{$^1$H}-NMR** (101 MHz, $CDCl_3$): $\delta_C$ 163.6, 141.0, 133.3, 130.0, 129.4, 128.4, 127.2, 126.2, 124.3, 34.6.

*The spectroscopic data are in agreement with those previously reported.*[3]

**2,2-Dimethyl-3-phenyl-2*H*-azirine, AC**

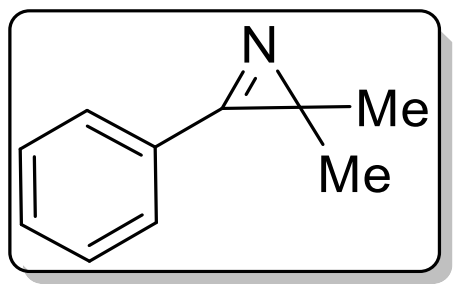


Prepared following **General Procedure 4** from 2-methyl-1-phenylpropan-1-one (1.48 g, 10.0 mmol). Purification by flash column chromatography (2% EtOAc /hexane) affords the title compound **AC** (320 mg, 2.20 mmol, 22% over 3 steps) as a colourless oil.

**$^1$H-NMR** (400 MHz, $CDCl_3$): $\delta_H$ 7.96 – 7.74 (2H, m), 7.64 – 7.44 (3H, m), 1.42 (6H, s).

**$^{13}$C{$^1$H}-NMR** (101 MHz, $CDCl_3$): $\delta_C$ 177.9, 132.7, 129.2, 129.1, 126.1, 34.0, 24.8.

*The spectroscopic data are in agreement with those previously reported.*[4]

**2-Phenyl-1-azaspiro[2.4]hept-1-ene, AD**

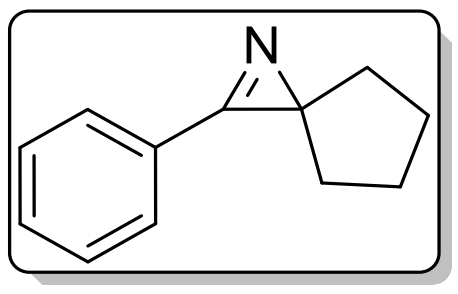


Prepared following **General Procedure 4** from cyclopentyl(phenyl)methanone (583 mg, 3.35 mmol). Purification by flash column chromatography (2% EtOAc /hexane) affords the title compound **AD** (0.39 g, 2.3 mmol, 69% over 3 steps) as a colourless oil.

**$^1$H-NMR** (400 MHz, $CDCl_3$): $\delta_H$ 7.88 – 7.78 (2H, m), 7.59 – 7.50 (3H, m), 2.02 – 1.93 (2H, m), 1.93 – 1.85 (2H, m), 1.85 – 1.74 (2H, m), 1.60 – 1.41 (2H, m).

**$^{13}$C{$^1$H}-NMR** (101 MHz, $CDCl_3$): $\delta_C$ 172.2, 132.6, 129.2, 129.1, 125.9, 44.4, 34.3, 26.4.

*The spectroscopic data are in agreement with those previously reported.*[4]

**2-Phenyl-1-azaspiro[2.5]oct-1-ene, AE**

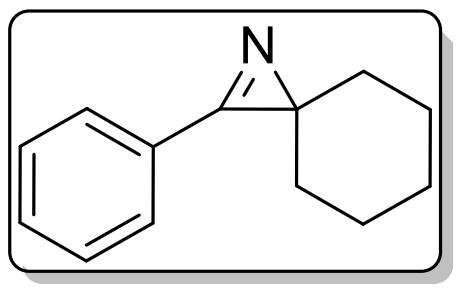

Prepared following **General Procedure 4** from cyclohexyl(phenyl)methanone (2.10 g, 11.2 mmol). Purification by flash column chromatography (2% EtOAc /hexane) affords the title compound **AE** (913 mg, 4.93 mmol, 44% over 3 steps) as a colourless oil.

**$^1$H-NMR** (400 MHz, $CDCl_3$): $\delta_H$ 7.84 (2H, m), 7.55 (3H, m), 1.91 (2H, m), 1.75 – 1.55 (8H, m).

**$^{13}$C{$^1$H}-NMR** (101 MHz, $CDCl_3$): $\delta_C$ 179.3, 132.7, 129.2, 129.2, 126.3, 40.6, 35.7, 26.7, 26.2.

*The spectroscopic data are in agreement with those previously reported.*[4]

**3'-(4-Fluorophenyl)-1-tosyl-1'-azaspiro[azetidine-3,2'-bicyclo[1.1.0]butane], 4**

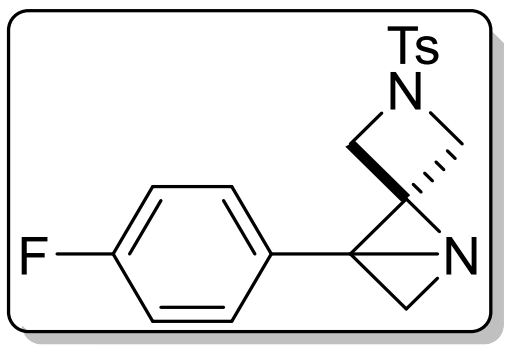


Prepared following **General Procedure 5** from 3-(4-fluorophenyl)-2*H*-azirine **J** (38 mg, 0.28 mmol) and (4-chlorophenyl)(1-tosylazetidin-3-yl)(2,4,6-trimethoxyphenyl)sulfonium tetrafluoroborate **1** (225 mg, 0.364 mmol). Work-up affords the crude material with a quantitative $^{1}$H-NMR yield of 73%. Purification by flash column chromatography (10-40% EtOAc/hexane) affords the title compound **4** (50 mg, 0.15 mmol, 52%) as a yellow oil.

**$^{1}$H-NMR** (400 MHz, $CDCl_3$): $\delta_H$ 7.73 (2H, d, *J* = 8.3 Hz), 7.36 (2H, d, *J* = 8.1 Hz), 7.20 – 7.10 (2H, m), 7.06 – 6.97 (2H, m), 4.12 (1H, dd, *J* = 9.3, 1.2 Hz), 4.01 (1H, dd, *J* = 10.1, 1.2 Hz), 3.94 (1H, dd, *J* = 10.0, 1.2 Hz), 3.84 (1H, dd, *J* = 9.3, 1.2 Hz), 2.59 (1H, d, *J* = 2.0 Hz), 2.46 (3H, s), 1.77 (1H, d, *J* = 2.0 Hz).

**$^{19}$F-NMR** (377 MHz, $CDCl_3$): $\delta_F$ -111.98 (s).

**$^{13}$C{$^{1}$H}-NMR** (101 MHz, $CDCl_3$): $\delta_C$ 163.1 (d, *J* = 248.5 Hz), 144.6, 131.7, 130.3 (d, *J* = 8.3 Hz), 130.0, 128.5, 127.1 (d, *J* = 3.3 Hz), 115.9 (d, *J* = 21.8 Hz), 65.2, 55.9, 50.8, 38.2, 21.7.

**IR** (neat, *v* cm$^{-1}$): 2925, 1528, 1343, 1157, 1092.

**HRMS** (ESI+): *m/z* calcd for $C_{18}H_{17}FN_2O_2SNa$ $[M+Na]^+$ 367.0887, found 367.0869

**1-Tosyl-3'-(4-(trifluoromethyl)phenyl)-1'-azaspiro[azetidine-3,2'-bicyclo[1.1.0]butane], 7**

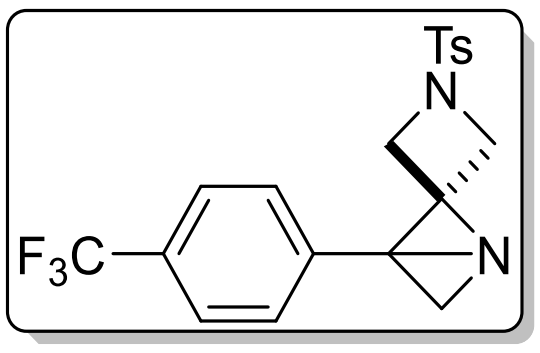


Prepared following **General Procedure 5** from 3-(4-(trifluoromethyl)phenyl)-2*H*-azirine **K** (52 mg, 0.28 mmol) and (4-chlorophenyl)(1-tosylazetidin-3-yl)(2,4,6-trimethoxyphenyl)sulfonium tetrafluoroborate **1** (225 mg, 0.364 mmol). Work-up affords the crude material with a quantitative $^1$H-NMR yield of 67%. Purification by flash column chromatography (10-40% EtOAc/hexane) affords the title compound **7** (55 mg, 0.14 mmol, 50%) as a colourless oil.

**$^1$H-NMR** (400 MHz, $CDCl_3$): $\delta_H$ 7.73 (2H, d, *J* = 8.2 Hz), 7.58 (2H, d, *J* = 8.1 Hz), 7.36 (2H, d, *J* = 8.1 Hz), 7.31 (2H, d, *J* = 8.1 Hz), 4.14 (1H, dd, *J* = 9.3, 1.2 Hz), 4.04 (1H, dd, *J* = 10.1, 1.2 Hz), 3.95 (1H, dd, *J* = 10.1, 1.2 Hz), 3.87 (1H, dd, *J* = 9.3, 1.1 Hz), 2.66 (1H, d, *J* = 1.9 Hz), 2.46 (3H, s), 1.83 (1H, d, *J* = 1.8 Hz).

**$^{19}$F-NMR** (377 MHz, $CDCl_3$): $\delta_F$ -62.77 (s).

**$^{13}$C{$^1$H}-NMR** (101 MHz, $CDCl_3$): $\delta_C$ 144.7, 135.7, 131.7, 131.1 (q, *J* = 32.8 Hz), 130.0, 128.9, 128.5, 125.7 (q, *J* = 3.8 Hz), 123.9 (q, *J* = 272.3 Hz), 65.5, 55.8, 50.8, 38.0, 21.7.

**IR** (neat, *v* cm$^{-1}$): 2979, 1621, 1345, 1321, 1159.

**HRMS** (ESI+): *m/z* calcd for $C_{19}H_{17}F_3N_2O_2SNa$ $[M+Na]^+$ 417.0855, found 417.0840

**3'-(4-Chlorophenyl)-1-tosyl-1'-azaspiro[azetidine-3,2'-bicyclo[1.1.0]butane], 8**

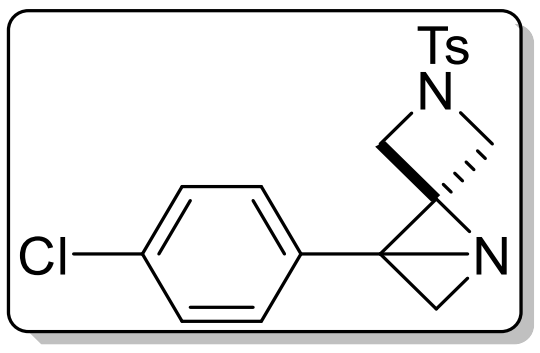


Prepared following **General Procedure 5** from 3-(4-chlorophenyl)-2*H*-azirine **L** (42 mg, 0.28 mmol) and (4-chlorophenyl)(1-tosylazetidin-3-yl)(2,4,6-trimethoxyphenyl)sulfonium tetrafluoroborate **1** (225 mg, 0.364 mmol). Work-up affords the crude material with a quantitative $^{1}$H-NMR yield of 81%. Purification by flash column chromatography (10-40% EtOAc/hexane) affords the title compound **8** (51 mg, 0.14 mmol, 51%) as a colourless oil.

**$^{1}$H-NMR** (400 MHz, $CDCl_3$): $\delta_H$ 7.73 (2H, d, *J* = 8.3 Hz), 7.36 (2H, d, *J* = 8.0 Hz), 7.32 – 7.27 (2H, m), 7.11 (2H, d, *J* = 8.4 Hz), 4.11 (1H, dd, *J* = 9.2, 1.2 Hz), 4.01 (1H, dd, *J* = 10.1, 1.1 Hz), 3.94 (1H, dd, *J* = 10.1, 1.2 Hz), 3.85 (1H, dd, *J* = 9.3, 1.1 Hz), 2.60 (1H, d, *J* = 1.9 Hz), 2.46 (3H, s), 1.78 (1H, d, *J* = 1.9 Hz).

**$^{13}$C{$^{1}$H}-NMR** (101 MHz, $CDCl_3$): $\delta_C$ 144.6, 135.0, 131.7, 130.0, 129.9, 129.8, 129.0, 128.5, 65.3, 55.8, 50.7, 38.1, 21.7.

**IR** (neat, *v* $cm^{-1}$): 2942, 1596, 1343, 1158, 1090.

**HRMS** (ESI+): *m/z* calcd for $C_{18}H_{17}{}^{35}ClN_2O_2SNa$ $[M+Na]^+$ 383.0591, found 383.0556

**3'-(3-Chlorophenyl)-1-tosyl-1'-azaspiro[azetidine-3,2'-bicyclo[1.1.0]butane], 9**

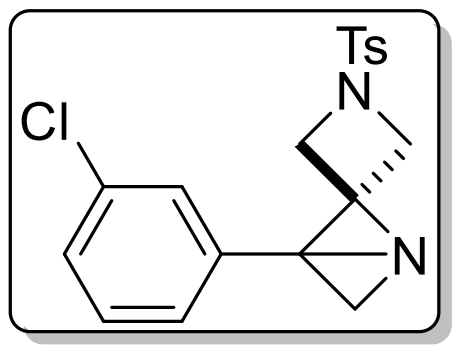


Prepared following **General Procedure 5** from 3-(3-chlorophenyl)-2*H*-azirine **M** (42 mg, 0.28 mmol) and (4-chlorophenyl)(1-tosylazetidin-3-yl)(2,4,6-trimethoxyphenyl)sulfonium tetrafluoroborate **1** (225 mg, 0.364 mmol). Work-up affords the crude material with a quantitative $^{1}$H-NMR yield of 74%. Purification by flash column chromatography (10-40% EtOAc/hexane) affords the title compound **9** (38 mg, 0.11 mmol, 38%) as a colourless oil.

**$^{1}$H-NMR** (400 MHz, $CDCl_3$): $\delta_H$ 7.75 – 7.72 (2H, m), 7.38 (2H, d, *J* = 8.0 Hz), 7.33 – 7.30 (1H, m), 7.26 (1H, app. t, *J* = 7.8 Hz), 7.12 (1H, app. t, *J* = 1.9 Hz), 7.06 (1H, app. dt, *J* = 7.5, 1.5 Hz), 4.11 (1H, dd, *J* = 9.3, 1.2 Hz), 4.03 (1H, dd, *J* = 10.1, 1.2 Hz), 3.95 (1H, dd, *J* = 10.1, 1.3 Hz), 3.87 (1H, dd, *J* = 9.4, 1.1 Hz), 2.62 (1H, d, *J* = 1.9 Hz), 2.47 (2H, s), 1.78 (1H, d, *J* = 1.9 Hz).

**$^{13}$C{$^{1}$H}-NMR** (101 MHz, $CDCl_3$): $\delta_C$ 144.6, 134.7, 133.5, 131.6, 130.1, 130.1, 129.2, 128.6, 128.5, 126.6, 65.4, 55.9, 55.8, 50.8, 38.0, 21.8.

**IR** (neat, *v* $cm^{-1}$): 2940, 1598, 1345, 1160, 1093.

**HRMS** (ESI+): *m/z* calcd for $C_{18}H_{17}{}^{35}ClN_2O_2SNa$ $[M+Na]^+$ 383.0591, found 383.0577

**3'-(4-Bromophenyl)-1-tosyl-1'-azaspiro[azetidine-3,2'-bicyclo[1.1.0]butane], 10**

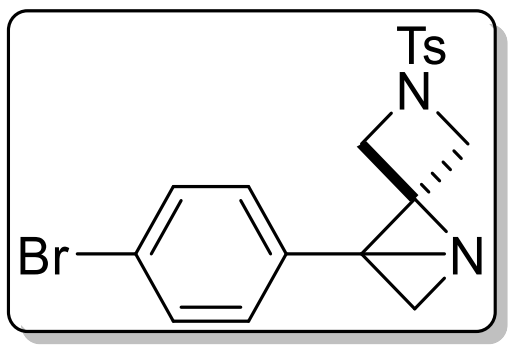


Prepared following **General Procedure 5** from 3-(4-bromophenyl)-2*H*-azirine **N** (35 mg, 0.18 mmol) and (4-chlorophenyl)(1-tosylazetidin-3-yl)(2,4,6-trimethoxyphenyl)sulfonium tetrafluoroborate **1** (150 mg, 0.25 mmol). Work-up affords the crude material with a quantitative $^1$H-NMR yield of 47%. Purification by flash column chromatography (10-40% EtOAc/hexane) affords the title compound **10** (20 mg, 0.050 mmol, 28%) as a yellow oil.

**$^1$H-NMR** (400 MHz, $CDCl_3$): $\delta_H$ 7.73 (2H, d, *J* = 7.9 Hz), 7.45 (2H, d, *J* = 8.2 Hz), 7.36 (2H, d, *J* = 7.9 Hz), 7.05 (2H, d, *J* = 8.2 Hz), 4.11 (1H, d, *J* = 9.2 Hz), 4.01 (1H, d, *J* = 10.1 Hz), 3.94 (1H, d, *J* = 10.0 Hz), 3.85 (1H, d, *J* = 9.2 Hz), 2.60 (1H, d, *J* = 1.9 Hz), 2.46 (3H, s), 1.78 (1H, d, *J* = 1.8 Hz).

**$^{13}$C{$^1$H}-NMR** (101 MHz, $CDCl_3$): $\delta_C$ 144.6, 131.9, 131.7, 130.5, 130.1, 130.0, 128.5, 123.1, 65.3, 55.8, 55.8, 50.7, 38.2, 21.7.

**IR** (neat, *v* $cm^{-1}$): 2937, 1586, 1344, 1159, 1093.

**HRMS** (ESI+): *m/z* calcd for $C_{18}H_{17}{}^{79}BrN_2O_2SNa$ $[M+Na]^+$ 427.0086, found 427.0102

**Naphthalen-2-yl 4-(1-tosyl-1'-azaspiro[azetidine-3,2'-bicyclo[1.1.0]butan]-3'-yl)benzoate, 11**

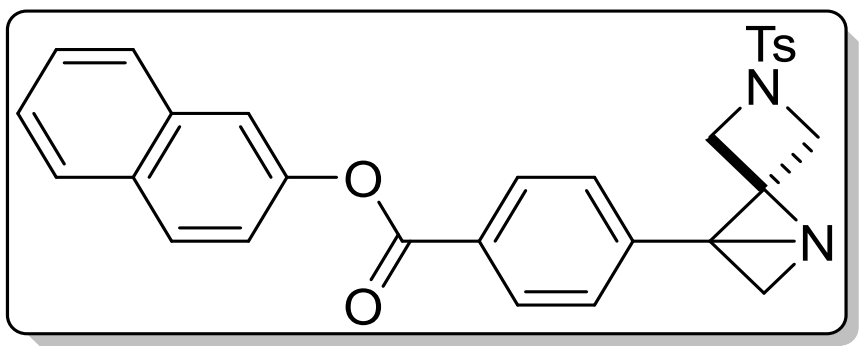

Prepared following a modification of **General Procedure 5** from naphthalen-2-yl 4-(2*H*-azirin-3-yl)benzoate **O** (38 mg, 0.13 mmol) and (4-chlorophenyl)(1-tosylazetidin-3-yl)(2,4,6-trimethoxyphenyl)sulfonium tetrafluoroborate **1** (104 mg, 0.17 mmol). The reaction mixture was concentrated under reduced pressure to afford the crude material with a quantitative $^1$H-NMR yield of 43%. Purification by flash column chromatography (50% dichloromethane/hexane to 20% EtOAc/hexane) affords the title compound **11** (15 mg, 0.030 mmol, 23%) as a white solid.

*Note: in this case, addition of MeOH to quench the reaction can lead to LiHMDS-mediated transesterification to the corresponding methyl ester and is therefore avoided.*[7] *Upon completion of the reaction, the mixture is concentrated under reduced pressure.*

**$^1$H-NMR** (400 MHz, $CDCl_3$): $\delta_H$ 8.21 (2H, d, *J* = 8.3 Hz), 7.94 – 7.81 (3H, m), 7.77 (2H, d, *J* = 8.3 Hz), 7.70 (1H, d, *J* = 2.3 Hz), 7.54 – 7.45 (2H, m), 7.43 – 7.30 (5H, m), 4.18 (1H, dd, *J* = 9.3, 1.1 Hz), 4.12 – 4.01 (2H, m), 3.90 (1H, dd, *J* = 9.2, 1.1 Hz), 2.73 (1H, d, *J* = 1.9 Hz), 2.48 (3H, s), 1.87 (1H, d, *J* = 1.9 Hz).

**$^{13}$C{$^1$H}-NMR** (101 MHz, $CDCl_3$): $\delta_C$ 164.8, 148.6, 144.7, 137.6, 133.9, 131.7, 131.7, 130.6, 130.1, 130.1, 129.7, 128.7, 128.5, 128.0, 127.8, 126.8, 126.0, 121.2, 118.8, 65.6, 55.9, 55.8, 50.8, 38.2, 21.8.

**IR** (neat, *v* cm$^{-1}$): 2943, 1725, 1346, 1269, 1162.

**HRMS** (ESI+): *m/z* calcd for $C_{29}H_{24}N_2O_4SNa$ [M+Na]$^+$ 519.1349, found 519.1329

**Oxetan-3-yl 4-(1-tosyl-1'-azaspiro[azetidine-3,2'-bicyclo[1.1.0]butan]-3'-yl)benzoate, 12**

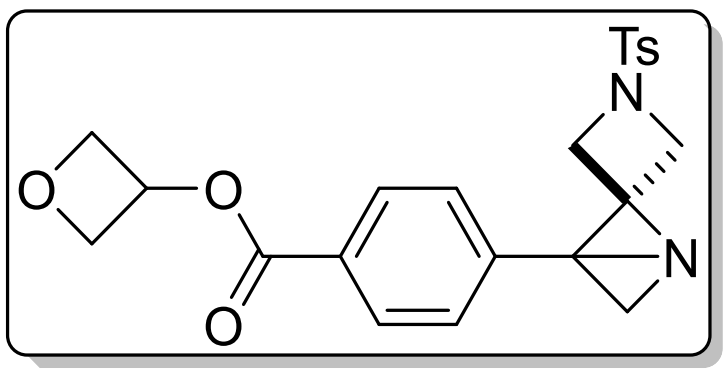


Prepared following a modification of **General Procedure 5** from oxetan-3-yl 4-(2*H*-azirin-3-yl)benzoate, **P** (43 mg, 0.20 mmol) and (4-chlorophenyl)(1-tosylazetidin-3-yl)(2,4,6-trimethoxyphenyl)sulfonium tetrafluoroborate **1** (156 mg, 0.26 mmol). The reaction mixture was concentrated under reduced pressure to afford the crude material with a quantitative $^1$H-NMR yield of 54%. Purification by flash column chromatography (50% dichloromethane/hexane to 70% EtOAc/hexane) affords the title compound **12** (27 mg, 0.063 mmol, 32%) as a white solid.

*Note: in this case, addition of MeOH to quench the reaction can lead to LiHMDS-mediated transesterification to the corresponding methyl ester and is therefore avoided.[7] Upon completion of the reaction, the mixture is concentrated under reduced pressure.*

**$^1$H-NMR** (400 MHz, $CDCl_3$): $\delta_H$ 8.07 – 8.00 (2H, m), 7.77 – 7.71 (2H, m), 7.41 – 7.34 (2H, m), 7.31 – 7.27 (2H, m), 5.67 (1H, m), 5.00 (2H, m), 4.78 (2H, m), 4.15 (1H, dd, *J* = 9.2, 1.2 Hz), 4.07 – 3.96 (2H, m), 3.87 (1H, dd, *J* = 9.2, 1.1 Hz), 2.69 (1H, d, *J* = 1.9 Hz), 2.47 (3H, s), 1.84 (1H, d, *J* = 1.9 Hz).

**$^{13}$C{$^1$H}-NMR** (101 MHz, $CDCl_3$): $\delta_C$ 165.3, 144.7, 137.4, 131.7, 130.1, 129.8, 128.7, 128.5, 77.7, 68.8, 65.6, 55.8, 55.8, 50.8, 38.2, 21.8.

**IR** (neat, *v* $cm^{-1}$): 2950, 1718, 1346, 1272, 1162.

**HRMS** (ESI+): *m/z* calcd for $C_{22}H_{22}N_2O_5SNa$ $[M+Na]^+$ 449.1142, found 449.1136

**6-Bromochroman-4-yl 4-(1-tosyl-1'-azaspiro[azetidine-3,2'-bicyclo[1.1.0]butan]-3'-yl)benzoate, 13**

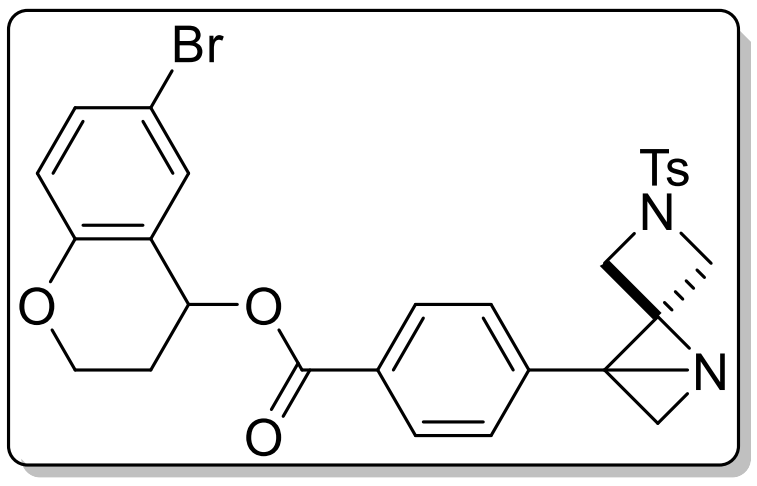

Prepared following a modification of **General Procedure 5** from 6-bromochroman-4-yl 4-(2*H*-azirin-3-yl)benzoate, **Q** (45 mg, 0.12 mmol) and (4-chlorophenyl)(1-tosylazetidin-3-yl)(2,4,6-trimethoxyphenyl)sulfonium tetrafluoroborate **1** (95 mg, 0.16 mmol). The reaction mixture was concentrated under reduced pressure to afford the crude material with a quantitative $^{1}$H-NMR yield of 78%. Purification by flash column chromatography (5-40% EtOAc/hexane) affords the title compound **13** (25 mg, 0.043 mmol, 36%) as a white solid.

*Note: in this case, addition of MeOH to quench the reaction can lead to LiHMDS-mediated transesterification to the corresponding methyl ester and is therefore avoided.*[7] *Upon completion of the reaction, the mixture is concentrated under reduced pressure.*

**$^{1}$H-NMR** (400 MHz, $CDCl_3$): $\delta_H$ 8.00 (2H, d, *J* = 8.4 Hz), 7.73 (2H, d, *J* = 8.3 Hz), 7.47 (1H, d, *J* = 2.5 Hz), 7.37 (2H, d, *J* = 8.0 Hz), 7.33 (1H, dd, *J* = 8.8, 2.5 Hz), 7.28 – 7.22 (2H, m), 6.78 (1H, d, *J* = 8.8 Hz), 6.13 (1H, t, *J* = 3.9 Hz), 4.39 – 4.29 (2H, m), 4.14 (1H, dd, *J* = 9.3, 1.2 Hz), 4.03 (1H, d, *J* = 10.1 Hz), 3.96 (1H, dd, *J* = 10.1, 1.1 Hz), 3.87 (1H, dt, *J* = 9.2, 1.3 Hz), 2.67 (1H, d, *J* = 1.9 Hz), 2.46 (3H, s), 2.35 – 2.26 (1H, m), 2.23 (1H, m), 1.83 (1H, d, *J* = 1.9 Hz).

**$^{13}$C{$^{1}$H}-NMR** (101 MHz, $CDCl_3$): $\delta_C$ 165.3, 154.6, 144.6, 137.2, 133.4, 133.2, 131.7, 130.5, 130.1, 130.1, 128.6, 128.5, 122.0, 119.2, 112.6, 65.7, 65.5, 62.5, 55.8, 55.8, 50.8, 38.2, 28.3, 21.8.

**IR** (neat, $\nu$ cm$^{-1}$): 2976, 1715, 1346, 1261, 1159.

**HRMS** (ESI+): *m/z* calcd for $C_{28}H_{25}{}^{81}BrN_2O_5SNa$ $[M+Na]^+$ 605.0543, found 605.0523

**2-Isopropyl-5-methylcyclohexyl 4-(1-tosyl-1'-azaspiro[azetidine-3,2'-bicyclo[1.1.0]butan]-3'-yl)benzoate, 14**

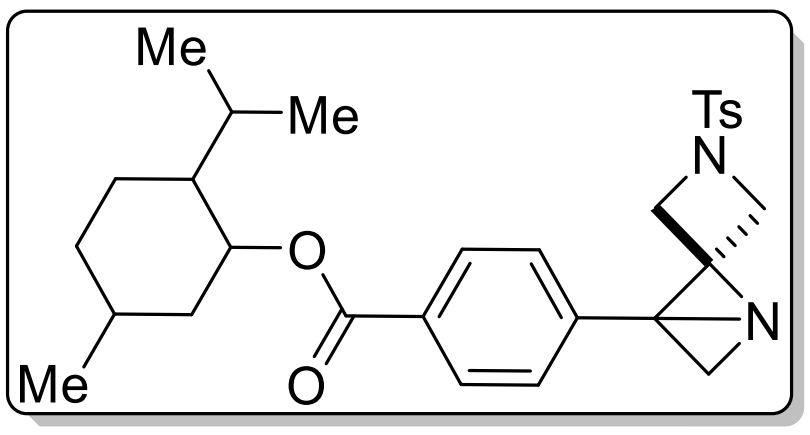


Prepared following **General Procedure 5** from 2-isopropyl-5-methylcyclohexyl 4-(2*H*-azirin-3-yl)benzoate **R** (40 mg, 0.13 mmol) and (4-chlorophenyl)(1-tosylazetidin-3-yl)(2,4,6- trimethoxyphenyl)sulfonium tetrafluoroborate **1** (105 mg, 0.17 mmol). Work-up affords the crude material with a quantitative $^1$H-NMR yield of 66%. Purification by flash column chromatography (10-50% EtOAc/hexane) affords the title compound **14** (25 mg, 0.050 mmol, 37%) as a yellow oil.

**$^1$H-NMR** (400 MHz, $CDCl_3$): $\delta_H$ 7.99 (2H, d, *J* = 8.1 Hz), 7.74 (2H, d, *J* = 8.0 Hz), 7.43 – 7.33 (2H, m), 7.24 (2H, dd, *J* = 8.4, 2.5 Hz), 4.93 (1H, td, *J* = 10.9, 4.4 Hz), 4.14 (1H, dd, *J* = 9.3, 1.1 Hz), 4.04 (1H,m), 4.01 – 3.93 (1H, m), 3.88 (1H, d, *J* = 9.2 Hz), 2.66 (1H, d, *J* = 1.9 Hz), 2.46 (3H, s), 2.15 – 2.07 (1H, m), 1.93 (1H, m ), 1.82 (1H, d, *J* = 1.9 Hz), 1.81 – 1.68 (2H, m), 1.64 – 1.49 (2H, m), 1.12 (2H, m), 0.92 (7H, dd, *J* = 6.8, 5.6 Hz), 0.79 (3H, d, *J* = 6.9 Hz).

**$^{13}$C{$^1$H}-NMR** (101 MHz, $CDCl_3$): $\delta_C$ 165.5, 144.6, 136.4, 131.7, 131.4, 130.1, 129.9, 128.5, 128.4, 75.3, 65.5, 55.9, 55.9, 50.8, 50.7, 47.4, 41.1, 38.3, 34.4, 31.6, 26.7, 23.8, 22.2, 21.7, 20.9, 16.7.

*Some signals are doubled due to the presence of diastereomers.*

**IR** (neat, *v* cm$^{-1}$): 2954, 1710, 1348, 1273, 1162.

**HRMS** (ESI+): *m/z* calcd for $C_{29}H_{36}N_2O_4SNa$ $[M+Na]^+$ 531.2288, found 531.2270

**1,7,7-Trimethylbicyclo[2.2.1]heptan-2-yl 4-(1-tosyl-1'-azaspiro[azetidine-3,2'-bicyclo[1.1.0]butan]-3'-yl)benzoate, 15**

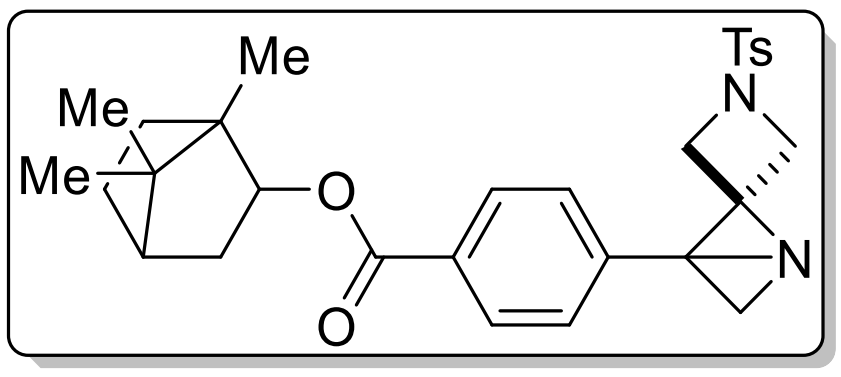


Prepared following **General Procedure 5** from 1,7,7-trimethylbicyclo[2.2.1]heptan-2-yl 4-(2*H*-azirin-3-yl)benzoate **S** (42 mg, 0.14 mmol) and (4-chlorophenyl)(1-tosylazetidin-3-yl)(2,4,6-trimethoxyphenyl)sulfonium tetrafluoroborate **1** (111 mg, 0.18 mmol). Work-up affords the crude material with a quantitative $^{1}$H-NMR yield of 83%. Purification by flash column chromatography (5-40% EtOAc/hexane) affords the title compound **15** (44 mg, 0.087 mmol, 61%) as a yellow oil.

**$^{1}$H-NMR** (400 MHz, $CDCl_3$): $\delta_H$ 8.00 (2H, d, *J* = 8.3 Hz), 7.74 (2H, d, *J* = 8.3 Hz), 7.37 (2H, d, *J* = 8.0 Hz), 7.24 (2H, d, *J* = 8.4 Hz), 5.11 (1H, ddd, *J* = 9.9, 3.5, 2.1 Hz), 4.15 (1H, dd, *J* = 9.3, 1.2 Hz), 4.05 (1H, dd, *J* = 10.1, 1.1 Hz), 3.99 – 3.95 (1H , m), 3.88 (1H, dd, *J* = 9.3, 1.1 Hz), 2.67 (1H, d, *J* = 1.9 Hz), 2.47 (4H, m), 2.10 (1H, m), 1.83 (1H, d, *J* = 1.9 Hz), 1.81 – 1.77 (1H, m), 1.74 (1H, t, *J* = 4.5 Hz), 1.42 (1H, m), 1.33 – 1.25 (1H, m), 1.11 (1H, dd, *J* = 13.8, 3.5 Hz), 0.96 (3H, s), 0.91 (6H, d, *J* = 3.9 Hz).

**$^{13}$C{$^{1}$H}-NMR** (101 MHz, $CDCl_3$): $\delta_C$ 166.3, 144.6, 136.5, 131.7, 131.5, 130.1, 129.8, 128.5, 128.5, 81.0, 65.5, 55.9, 55.9, 50.7, 49.3, 48.0, 45.1, 38.3, 37.0, 28.2, 27.5, 21.8, 19.8, 19.0, 13.7.

**IR** (neat, *v* $cm^{-1}$): 2953, 1712, 1271, 1160, 1016.

**HRMS** (ESI+): *m/z* calcd for $C_{29}H_{34}N_2O_4SNa$ $[M+Na]^+$ 529.2131, found 529.2086

**Butane-1,4-diyl bis(4-(1-tosyl-1'-azaspiro[azetidine-3,2'-bicyclo[1.1.0]butan]-3'-yl)benzoate), 16**

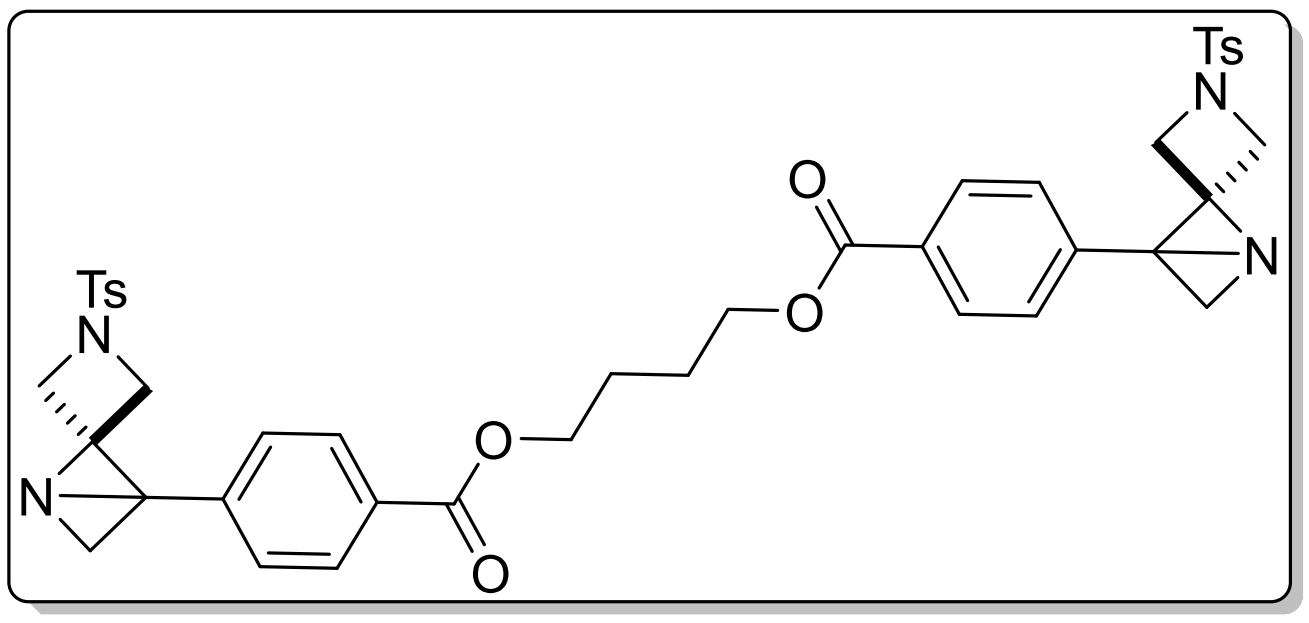


Prepared following a modification of **General Procedure 5** from butane-1,4-diyl bis(4-(2*H*-azirin-3-yl)benzoate) **T** (40 mg, 0.11 mmol) and (4-chlorophenyl)(1-tosylazetidin-3-yl)(2,4,6- trimethoxyphenyl)sulfonium tetrafluoroborate **1** (173 mg, 0.29 mmol, 2.6 equiv.), and LiHMDS (1M in THF, 3.2 equiv.). The reaction mixture was concentrated under reduced pressure to afford the crude material with a quantitative $^1$H-NMR yield of 63%. Purification by flash column chromatography (20% dichloromethane/hexane to 60% EtOAc/hexane) affords the title compound **16** (15 mg, 0.019 mmol, 18%) as a white solid.

*Note: in this case, addition of MeOH to quench the reaction can lead to LiHMDS-mediated transesterification to the corresponding methyl ester and is therefore avoided.*[7] *Upon completion of the reaction, the mixture is concentrated under reduced pressure.*

**$^1$H-NMR** (400 MHz, $CDCl_3$): $\delta_H$ 8.11 – 7.94 (4H, m), 7.74 (4H, d, *J* = 8.2 Hz), 7.37 (4H, d, *J* = 7.9 Hz), 7.32 – 7.19 (4H, m), 4.53 – 4.33 (4H, m), 4.15 (2H, d, *J* = 9.2 Hz), 4.01 (4H, m), 3.86 (2H, d, *J* = 9.2 Hz), 2.68 (2H, d, *J* = 1.9 Hz), 2.46 (6H, s), 2.03 – 1.93 (4H, m), 1.83 (2H, d, *J* = 2.0 Hz).

**$^{13}$C{$^1$H}-NMR** (101 MHz, $CDCl_3$): $\delta_C$ 166.0, 144.7, 136.8, 131.7, 130.8, 130.1, 129.9, 128.5, 128.5, 65.5, 64.8, 55.9, 55.8, 50.7, 38.2, 25.7, 21.8.

**IR** (neat, *v* cm$^{-1}$): 2922, 1713, 1344, 1269, 1159.

**HRMS** (ESI+): *m/z* calcd for $C_{42}H_{42}N_4O_8S_2Na$ $[M+Na]^+$ 817.2342, found 817.2286

**Pyrrolidin-1-yl(4-(1-tosyl-1'-azaspiro[azetidine-3,2'-bicyclo[1.1.0]butan]-3'-yl)phenyl)methanone, 17**

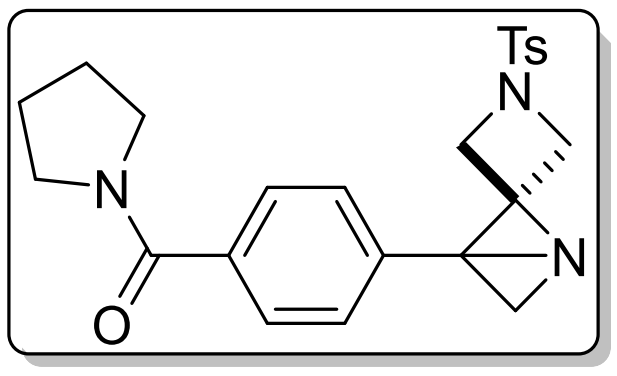


Prepared following **General Procedure 5** from (4-(2*H*-azirin-3-yl)phenyl)(pyrrolidin-1-yl)methanone **U** (39 mg, 0.18 mmol) and (4-chlorophenyl)(1-tosylazetidin-3-yl)(2,4,6-trimethoxyphenyl)sulfonium tetrafluoroborate **1** (145 mg, 0.24 mmol). Work-up affords the crude material with a quantitative $^{1}$H-NMR yield of 53%. Purification by flash column chromatography (50% EtOAc/hexane to MeOH) affords the title compound **17** (35 mg, 0.083 mmol, 45%) as a yellow oil.

**$^{1}$H-NMR** (400 MHz, $CDCl_3$): $\delta_H$ 7.73 (2H, d, *J* = 8.2 Hz), 7.47 (2H, d, *J* = 8.1 Hz), 7.37 (2H, d, *J* = 7.9 Hz), 7.20 (2H, d, *J* = 8.0 Hz), 4.12 (1H, d, *J* = 9.2 Hz), 4.05 – 3.94 (3H, m), 3.63 (2H, t, *J* = 7.0 Hz), 3.38 (2H, t, *J* = 6.6 Hz), 2.64 (1H, d, *J* = 1.9 Hz), 2.45 (3H, d, *J* = 3.6 Hz), 1.98 – 1.93 (2H, m), 1.90 – 1.85 (2H, m), 1.80 (1H, d, *J* = 1.9 Hz).

**$^{13}$C{$^{1}$H}-NMR** (101 MHz, $CDCl_3$): $\delta_C$ 169.0, 144.6, 137.8, 133.2, 131.7, 130.1, 128.5, 128.4, 127.6, 65.3, 55.9, 55.9, 50.7, 49.7, 46.4, 38.3, 26.5, 24.5, 21.8.

**IR** (neat, *v* $cm^{-1}$): 2971, 2875, 1610, 1432, 1159.

**HRMS** (ESI+): *m/z* calcd for $C_{23}H_{25}N_3O_3SNa$ $[M+Na]^+$ 446.1509, found 446.1514

**3'-(Naphthalen-2-yl)-1-tosyl-1'-azaspiro[azetidine-3,2'-bicyclo[1.1.0]butane], 18**

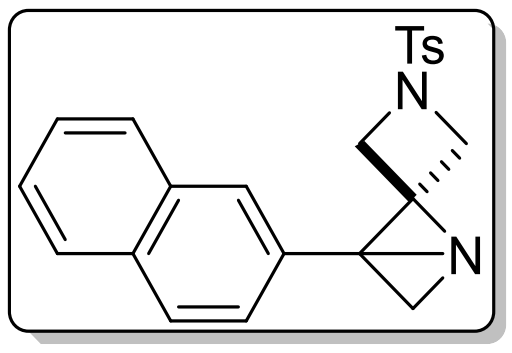

Prepared following **General Procedure 5** from 3-(naphthalen-2-yl)-2*H*-azirine **V** (48 mg, 0.29 mmol) and (4-chlorophenyl)(1-tosylazetidin-3-yl)(2,4,6-trimethoxyphenyl)sulfonium tetrafluoroborate **1** (226 mg, 0.37 mmol). Work-up affords the crude material with a quantitative $^{1}$H-NMR yield of 63%. Purification by flash column chromatography (5-40% EtOAc/hexane) affords the title compound **18** (20 mg, 0.053 mmol, 19%) as a yellow oil.

**$^{1}$H-NMR** (400 MHz, $CDCl_3$): $\delta_H$ 7.85 – 7.70 (6H, m), 7.54 – 7.48 (2H, m), 7.37 (2H, d, *J* = 7.9 Hz), 7.21 (1H, dd, *J* = 8.4, 1.8 Hz), 4.19 (1H, d, *J* = 9.2 Hz), 4.08 (2H, s), 3.91 (1H, d, *J* = 9.2 Hz), 2.74 (1H, d, *J* = 2.0 Hz), 2.45 (3H, s), 1.86 (1H, d, *J* = 1.9 Hz).

**$^{13}$C{$^{1}$H}-NMR** (101 MHz, $CDCl_3$): $\delta_C$ 144.5, 133.4, 133.1, 131.7, 130.1, 128.7, 128.6, 128.5, 128.3, 128.0, 127.8, 126.8, 126.8, 125.0, 65.3, 56.1, 56.1, 50.8, 39.0, 21.7.

**IR** (neat, *v* cm$^{-1}$): 3054, 1597, 1344, 1161, 1093.

**HRMS:** *exact mass not found in ESI+ or ESI- mode*

**3'-(*p*-Tolyl)-1-tosyl-1'-azaspiro[azetidine-3,2'-bicyclo[1.1.0]butane], 19**

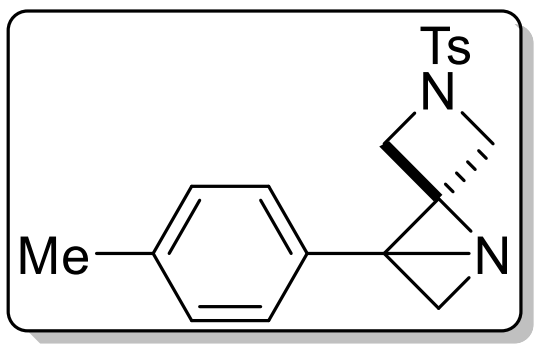


Prepared following **General Procedure 5** from 3-(*p*-tolyl)-2*H*-azirine **W** (37 mg, 0.28 mmol) and (4-chlorophenyl)(1-tosylazetidin-3-yl)(2,4,6-trimethoxyphenyl)sulfonium tetrafluoroborate **1** (225 mg, 0.37 mmol). Work-up affords the crude material with a quantitative $^{1}$H-NMR yield of 81%. Purification by flash column chromatography (5-40% EtOAc/hexane) affords the title compound **19** (10 mg, 0.029 mmol, 10%) as a yellow oil.

**$^{1}$H-NMR** (400 MHz, $CDCl_3$): $\delta_H$ 7.78 – 7.68 (2H, m), 7.37 (2H, d, *J* = 7.9 Hz), 7.14 (2H, d, *J* = 7.9 Hz), 7.06 (2H, d, *J* = 8.1 Hz), 4.12 (1H, d, *J* = 9.2 Hz), 4.01 (1H, s), 3.84 (1H, d, *J* = 9.1 Hz), 3.79 – 3.75 (1H, m), 2.61 (1H, d, *J* = 2.0 Hz), 2.47 (3H, s), 2.35 (3H, s), 1.75 (1H, d, *J* = 2.1 Hz).

**$^{13}$C{$^{1}$H}-NMR** (101 MHz, $CDCl_3$): $\delta_C$ 144.4, 138.8, 131.7, 129.9, 129.3, 128.4, 128.2, 127.8, 64.9, 56.0, 55.9, 50.4, 38.6, 21.6, 21.3.

**IR** (neat, *v* $cm^{-1}$): 2925, 1345, 1160, 1094, 1018.

**HRMS** (ESI+): *m/z* calcd for $C_{19}H_{20}N_2O_2SNa$ $[M+Na]^+$ 363.1138, found 363.1122

**3'-(4-Methoxyphenyl)-1-tosyl-1'-azaspiro[azetidine-3,2'-bicyclo[1.1.0]butane], 20**

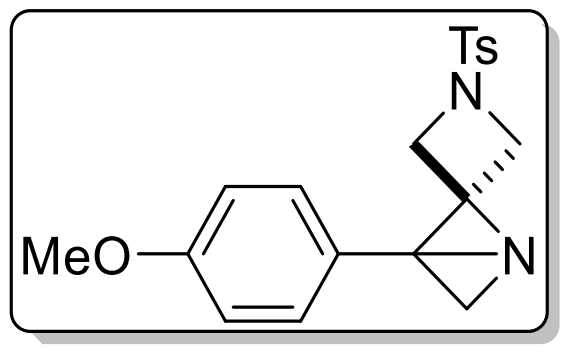


Prepared following **General Procedure 5** from 3-(4-methoxyphenyl)-2*H*-azirine **X** (41 mg, 0.28 mmol) and (4-chlorophenyl)(1-tosylazetidin-3-yl)(2,4,6-trimethoxyphenyl)sulfonium tetrafluoroborate **1** (225 mg, 0.37 mmol). Work-up affords the crude material with a quantitative $^{1}$H-NMR yield of 78%. Purification by flash column chromatography (5-40% EtOAc/hexane) affords the title compound **20** (21 mg, 0.059 mmol, 21%) as a colourless oil.

**$^{1}$H-NMR** (400 MHz, $CDCl_3$): $\delta_H$ 7.73 (2H, d, *J* = 8.3 Hz), 7.40-7.33 (2H, m), 7.09 (2H, d, *J* = 8.7 Hz), 6.85 (2H, d, *J* = 8.8 Hz), 4.11 (1H, dd, *J* = 9.1, 1.0 Hz), 4.04 – 3.96 (2H, m), 3.80 (4H, m), 2.58 (1H, d, *J* = 2.0 Hz), 2.46 (3H, s), 1.74 (1H, d, *J* = 2.0 Hz).

**$^{13}$C{$^{1}$H}-NMR** (101 MHz, $CDCl_3$): $\delta_C$ 160.2, 144.5, 131.7, 130.0, 129.7, 128.5, 123.0, 114.3, 65.0, 56.1, 56.0, 55.5, 50.6, 38.7, 21.7.

**IR** (neat, *v* cm$^{-1}$): 2937, 1598, 1512, 1340, 1159.

**HRMS** *exact mass not found in ESI+ or ESI- mode.*

**3'-(2,2-Difluorobenzo[*d*][1,3]dioxol-5-yl)-1-tosyl-1'-azaspiro[azetidine-3,2'-bicyclo[1.1.0]butane], 21**

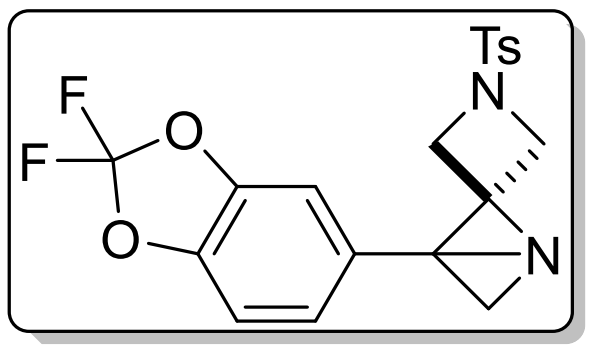

Prepared following **General Procedure 5** from 3-(2,2-difluorobenzo[*d*][1,3]dioxol-5-yl)-2*H*-azirine **Y** (35 mg, 0.18 mmol) and (4-chlorophenyl)(1-tosylazetidin-3-yl)(2,4,6-trimethoxyphenyl)sulfonium tetrafluoroborate **1** (140 mg, 0.23 mmol). Work-up affords the crude material with a quantitative $^{1}$H-NMR yield of 89%. Purification by flash column chromatography (10-40% EtOAc/hexane) affords the title compound **21** (32 mg, 0.079 mmol, 44%) as a colourless oil.

**$^{1}$H-NMR** (400 MHz, $CDCl_3$): $\delta_H$ 7.74 (2H, d, *J* = 8.3 Hz), 7.38 (2H, d, *J* = 7.9 Hz), 7.02 (1H, d, *J* = 8.2 Hz), 6.94 (1H, m), 6.81 (1H, d, *J* = 1.7 Hz), 4.12 (1H, dd, *J* = 9.4, 1.2 Hz), 4.01 (1H, dd, *J* = 10.0, 1.2 Hz), 3.91 – 3.84 (2H, m), 2.59 (1H, d, *J* = 1.9 Hz), 2.48 (3H, s), 1.80 (1H, d, *J* = 1.9 Hz).

**$^{19}$F-NMR** (377 MHz, $CDCl_3$): $\delta_F$ -49.93 (s).

**$^{13}$C{$^{1}$H}-NMR** (101 MHz, $CDCl_3$): $\delta_C$ 144.7, 144.2, 144.0, 131.7 (t, *J* = 256.4 Hz), 130.1, 129.2, 128.5, 127.4, 124.2, 109.8, 109.7, 65.6, 55.8, 55.7, 51.3, 38.3, 21.7.

**IR** (neat, *v* $cm^{-1}$): 2927, 1486, 1233, 1157, 1093.

**HRMS** (ESI+): *m/z* calcd for $C_{19}H_{16}F_2N_2O_4SNa$ $[M+Na]^+$ 429.0691, found 429.0663

**1-Tosyl-3'-(1-tosyl-1*H*-indol-2-yl)-1'-azaspiro[azetidine-3,2'-bicyclo[1.1.0]butane], 22**

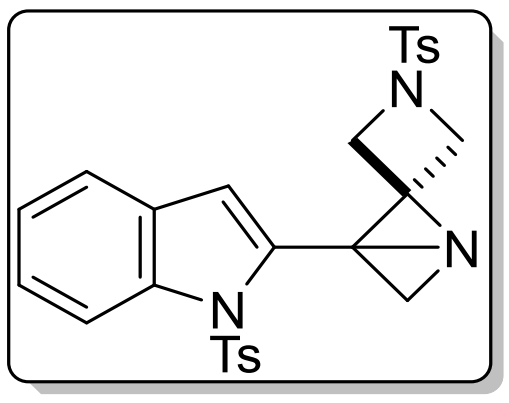


Prepared following **General Procedure 5** from 2-(2*H*-azirin-3-yl)-1-tosyl-1*H*-indole **Z** (43 mg, 0.14 mmol) and (4-chlorophenyl)(1-tosylazetidin-3-yl)(2,4,6-trimethoxyphenyl)sulfonium tetrafluoroborate **1** (140 mg, 0.23 mmol). Work-up affords the crude material with a quantitative $^{1}$H-NMR yield of 38%. Purification by flash column chromatography (10-50% EtOAc/hexane) affords the title compound **22** (26 mg, 0.050 mmol, 36%) as a colourless oil.

**$^{1}$H-NMR** (400 MHz, $CDCl_3$): $\delta_H$ 8.20 (1H, dd, *J* = 8.5, 1.0 Hz), 7.81 – 7.77 (2H, m), 7.77 – 7.72 (2H, m), 7.45 (1H,m), 7.39 (3H,m), 7.29 – 7.23 (3H, m), 6.46 (1H, d, *J* = 0.8 Hz), 4.29 (1H, dd, *J* = 9.0, 1.2 Hz), 3.89 (2H, ddd, *J* = 14.0, 9.5, 1.0 Hz), 3.56 (1H, dd, *J* = 9.8, 1.2 Hz), 2.54 (1H, d, *J* = 2.1 Hz), 2.49 (3H, s), 2.37 (3H, s), 1.98 (1H, d, *J* = 2.3 Hz).

**$^{13}$C{$^{1}$H}-NMR** (101 MHz, $CDCl_3$): $\delta_C$ 145.7, 144.7, 137.6, 135.6, 131.6, 131.5, 130.3, 130.1, 128.7, 128.4, 126.6, 125.5, 124.0, 121.6, 114.5, 111.4, 67.3, 55.6, 55.3, 53.3, 33.5, 21.7, 21.7.

**IR** (neat, *v* $cm^{-1}$): 2926, 1596, 1345, 1160, 1090.

**HRMS**: *exact mass not found in ESI+ or ESI- mode*

**3'-Decyl-1-tosyl-1'-azaspiro[azetidine-3,2'-bicyclo[1.1.0]butane], 23**

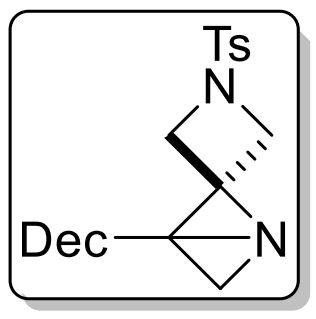

Prepared following **General Procedure 5** from 3-decyl-2*H*-azirine **AA** (42 mg, 0.23 mmol) and (4-chlorophenyl)(1-tosylazetidin-3-yl)(2,4,6-trimethoxyphenyl)sulfonium tetrafluoroborate **1** (182 mg, 0.30 mmol). Work-up affords the crude material with a quantitative $^1$H-NMR yield of 71%. Purification by flash column chromatography (50% dichloromethane/hexane to 20% EtOAc/hexane) affords the title compound **23** (24 mg, 0.061 mmol, 27%) as a colourless oil.

**$^1$H-NMR** (400 MHz, $CDCl_3$): $\delta_H$ 7.73 (2H, d, *J* = 8.3 Hz,), 7.36 (2H, d, *J* = 8.0 Hz), 4.01 – 3.91 (2H, m), 3.94 – 3.84 (2H, m), 3.70 (1H, d, *J* = 8.9 Hz), 2.45 (3H, s), 2.21 (1H, d, *J* = 1.8 Hz), 1.90 – 1.75 (1H, m), 1.69 – 1.55 (1H, m), 1.32 (1H, d, *J* = 1.9 Hz), 1.24 (16H, s), 0.87 (3H, t, *J* = 6.8 Hz).

**$^{13}$C{$^1$H}-NMR** (101 MHz, $CDCl_3$): $\delta_C$ 144.4, 131.7, 130.0, 128.5, 63.5, 56.1, 55.6, 48.3, 37.9, 32.0, 29.7, 29.6, 29.5, 29.5, 29.4, 26.2, 25.3, 22.8, 21.7, 14.2.

**IR** (neat, *v* cm$^{-1}$): 2923, 2853, 1347, 1160, 1092.

**HRMS** (ESI+): *m/z* calcd for $C_{22}H_{34}N_2O_2SNa$ [M+Na]$^+$ 413.2233, found 413.2234

**3',4'-Diphenyl-1-tosyl-1'-azaspiro[azetidine-3,2'-bicyclo[1.1.0]butane], 24**

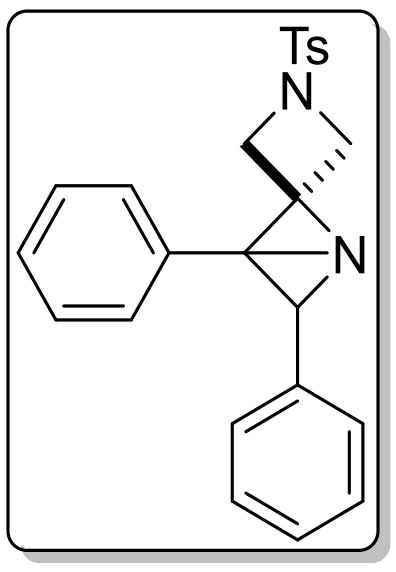

Prepared following **General Procedure 5** from 2,3-diphenyl-2*H*-azirine **AB** (39 mg, 0.20 mmol) and (4-chlorophenyl)(1-tosylazetidin-3-yl)(2,4,6-trimethoxyphenyl)sulfonium tetrafluoroborate **1** (160 mg, 0.26 mmol). Work-up affords the crude material with a quantitative $^1$H-NMR yield of 88%. Purification by flash column chromatography (50% dichloromethane/hexane to 40% EtOAc/hexane) affords the title compound **24** (25 mg, 0.062 mmol, 31%) as a white solid.

**$^1$H-NMR** (400 MHz, $CDCl_3$): $\delta_H$ 7.78 – 7.72 (2H, m), 7.33 (2H, d, *J* = 8.1 Hz), 7.29 – 7.21 (3H, m), 7.17 – 7.09 (3H, m), 7.02 (4H, m), 4.21 (2H, m), 4.08 – 3.92 (2H, m), 2.86 (1H, s), 2.44 (3H, s).

**$^{13}$C{$^1$H}-NMR** (101 MHz, $CDCl_3$): $\delta_C$ 144.5, 132.7, 131.7, 130.0, 129.6, 128.8, 128.5, 128.4, 128.3, 128.2, 128.2, 127.5, 62.1, 61.7, 56.1, 55.8, 45.1, 21.7.

**IR** (neat, *v* cm$^{-1}$): 3032, 1597, 1344, 1159, 1106.

**HRMS** (ESI+): *m/z* calcd for $C_{24}H_{22}N_2O_2SNa$ $[M+Na]^+$ 425.1294, found 425.1289.

**4',4'-Dimethyl-3'-phenyl-1-tosyl-1'-azaspiro[azetidine-3,2'-bicyclo[1.1.0]butane], 25**

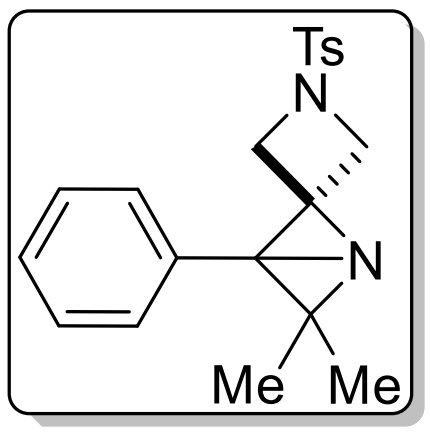


Prepared following **General Procedure 5** from 2,2-dimethyl-3-phenyl-2*H*-azirine **AC** (29 mg, 0.20 mmol) and (4-chlorophenyl)(1-tosylazetidin-3-yl)(2,4,6-trimethoxyphenyl)sulfonium tetrafluoroborate **1** (160 mg, 0.26 mmol). Work-up affords the crude material with a quantitative $^1$H-NMR yield of 64%. Purification by flash column chromatography (50% dichloromethane/hexane to 40% EtOAc/hexane) affords the title compound **25** (32 mg, 0.090 mmol, 45%) as a colourless oil, which co-eluted with an unidentified impurity (ca. 10%).

**$^1$H-NMR** (400 MHz, $CDCl_3$): $\delta_H$ 7.69 (3H, dd, *J* = 7.8, 6.1 Hz), 7.37 – 7.28 (4H, m), 7.14 – 7.07 (2H, m), 4.34 (1H, dd, *J* = 9.4, 1.3 Hz), 4.17 – 4.11 (1H, m), 4.01 – 3.96 (1H, m), 3.71 (1H, dd, *J* = 10.0, 1.2 Hz), 2.45 (3H, s), 1.11 (3H, s), 0.97 (3H, s).

**$^{13}$C{$^1$H}-NMR** (101 MHz, $CDCl_3$): $\delta_C$ 144.3, 131.8, 130.4, 130.0, 129.3, 129.1, 128.4, 128.3, 70.2, 68.8, 60.3, 57.6, 50.2, 23.1, 21.7, 18.3.

**HRMS** *exact mass not found in ESI+ or ESI- mode*

**3'-Phenyl-1-tosyl-1'-azadispiro[azetidine-3,2'-bicyclo[1.1.0]butane-4',1''-cyclopentane], 26**

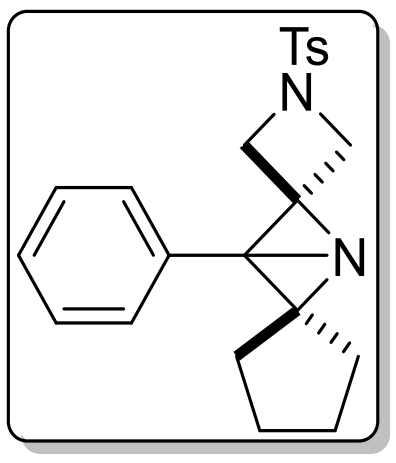

Prepared following **General Procedure 5** from 2-phenyl-1-azaspiro[2.4]hept-1-ene **AD** (36 mg, 0.21 mmol) and (4-chlorophenyl)(1-tosylazetidin-3-yl)(2,4,6-trimethoxyphenyl)sulfonium tetrafluoroborate **1** (166 mg, 0.27 mmol). Work-up affords the crude material with a quantitative $^{1}$H-NMR yield of 85%. Purification by flash column chromatography (50% dichloromethane/hexane to 25% EtOAc/hexane) affords the title compound **26** (40 mg, 0.104 mmol, 52%) as a colourless oil.

**$^{1}$H-NMR** (400 MHz, $CDCl_3$): $\delta_H$ 7.78 – 7.64 (2H, m), 7.40 – 7.30 (5H, m), 7.15 – 7.05 (2H, m), 4.20 (1H, dd, *J* = 9.4, 1.2 Hz), 4.06 (1H, d, *J* = 9.4 Hz), 3.92 (1H, d, *J* = 9.8 Hz), 3.71 (1H, dd, *J* = 9.7, 1.1 Hz), 2.47 (3H, s), 1.71 – 1.46 (8H, m).

**$^{13}$C{$^{1}$H}-NMR** (101 MHz, $CDCl_3$): $\delta_C$ 144.3, 131.7, 130.3, 130.0, 129.4, 128.7, 128.5, 128.5, 79.9, 67.0, 60.1, 56.9, 48.3, 31.8, 29.3, 26.5, 24.5, 21.8.

**IR** (neat, *v* $cm^{-1}$): 2953, 1345, 1271, 1158, 1101.

**HRMS** (ESI+): *m/z* calcd for $C_{22}H_{24}N_2O_2SNa$ $[M+Na]^+$ 403.1451, found 403.1436

**3'-Phenyl-1-tosyl-1'-azadispiro[azetidine-3,2'-bicyclo[1.1.0]butane-4',1''-cyclohexane], 27**

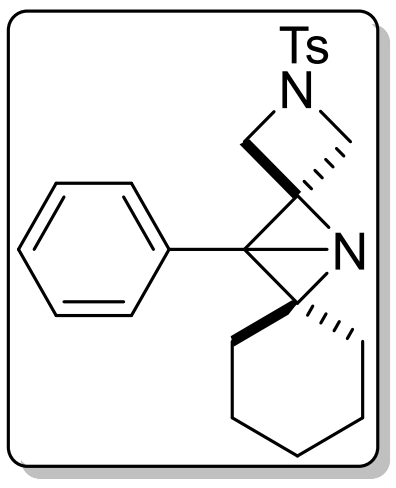


Prepared following **General Procedure 5** from 2-phenyl-1-azaspiro[2.5]oct-1-ene **AE** (39 mg, 0.21 mmol) and (4-chlorophenyl)(1-tosylazetidin-3-yl)(2,4,6-trimethoxyphenyl)sulfonium tetrafluoroborate **1** (166 mg, 0.27 mmol). Work-up affords the crude material with a quantitative $^{1}$H-NMR yield of 87%. Purification by flash column chromatography (50% dichloromethane/hexane to 20% EtOAc/hexane) affords the title compound **27** (46 mg, 0.11 mmol, 54%) as a colourless oil.

**$^{1}$H-NMR** (400 MHz, $CDCl_3$): $\delta_H$ 7.68 (2H, d, *J* = 8.2 Hz), 7.43 – 7.25 (5H, m), 7.19 – 7.10 (2H, m), 4.36 (1H, dd, *J* = 9.4, 1.2 Hz), 4.13 (1H, d, *J* = 9.4 Hz), 3.99 (1H, dd, *J* = 9.9, 1.1 Hz), 3.68 (1H, dd, *J* = 9.9, 1.2 Hz), 2.45 (3H, s), 1.55 – 1.23 (10 H, m).

**$^{13}$C{$^{1}$H}-NMR** (101 MHz, $CDCl_3$): $\delta_C$ 144.3, 132.0, 130.3, 130.0, 129.5, 128.6, 128.4, 128.3, 77.0, 69.2, 60.4, 57.7, 50.7, 32.9, 28.5, 25.9, 25.5, 25.1, 21.7.

**IR** (neat, *v* cm$^{-1}$): 2929, 2855, 1345, 1159, 1092.

**HRMS** (ESI+): *m/z* calcd for $C_{23}H_{26}N_2O_2SNa$ $[M+Na]^+$ 417.1607, found 417.1587

**(4-(1-Azaspiro[bicyclo[1.1.0]butane-2,3'-oxetan]-3-yl)phenyl)(pyrrolidin-1-yl)methanone, 28**

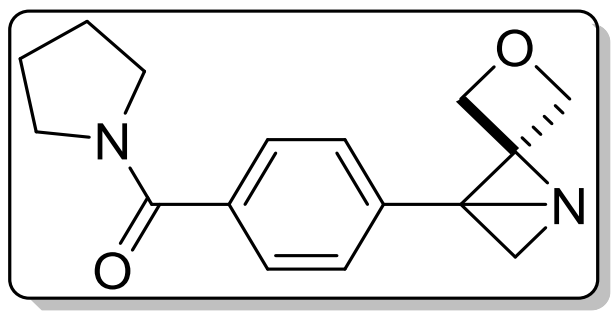

Prepared following **General Procedure 5** from (4-(2*H*-azirin-3-yl)phenyl)(pyrrolidin-1-yl)methanone **U** (39 mg, 0.18 mmol) and (4-chlorophenyl)(oxetan-3-yl)(2,4,6-trimethoxyphenyl)sulfonium tetrafluoroborate **2** (108 mg, 0.24 mmol). Work-up affords the crude material with a quantitative $^{1}$H-NMR yield of 50%. Purification by flash column chromatography (90% EtOAc/hexane to 5% MeOH/dichloromethane) affords the title compound **28** (20 mg, 0.074 mmol, 41%) as a blue oil.

**$^{1}$H-NMR** (400 MHz, $CDCl_3$): $\delta_H$ 7.52 – 7.49 (2H, m), 7.38 – 7.32 (2H, m), 4.97 – 4.89 (2H, m), 4.84 (1H, dd, *J* = 8.0, 1.0 Hz), 4.78 – 4.68 (1H, m), 3.62 (2H, t, *J* = 6.9 Hz), 3.39 (2H, t, *J* = 6.6 Hz), 2.69 (1H, d, *J* = 2.1 Hz), 1.97 – 1.90 (2H, m), 1.87 (2H, q, *J* = 6.4 Hz), 1.81 (1H, d, *J* = 2.1 Hz).

**$^{13}$C{$^{1}$H}-NMR** (101 MHz, $CDCl_3$): $\delta_C$ 169.1, 137.6, 134.3, 128.3, 127.5, 77.2, 76.5, 70.4, 49.9, 49.7, 46.3, 38.4, 26.5, 24.5.

**IR** (neat, *v* $cm^{-1}$): 3450, 2948, 2872, 1608, 1421.

**HRMS** (ESI+): *m/z* calcd for $C_{16}H_{18}N_2O_2Na$ $[M+Na]^+$ 293.1260, found 293.1245

**Naphthalen-2-yl 4-(1-azaspiro[bicyclo[1.1.0]butane-2,3'-oxetan]-3-yl)benzoate, 29**

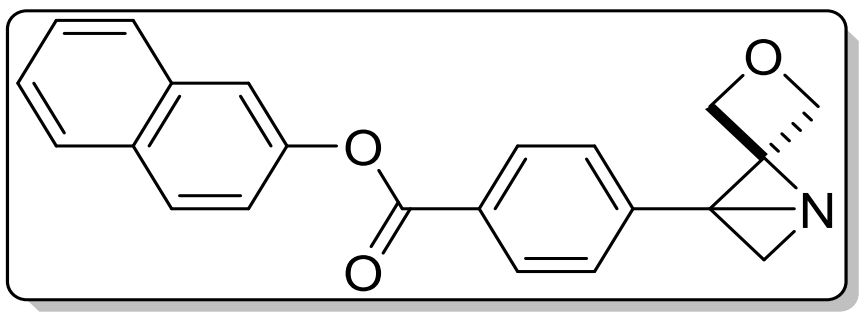


Prepared following a modification of **General Procedure 5** from naphthalen-2-yl 4-(2*H*-azirin-3-yl)benzoate **O** (38 mg, 0.13 mmol) and (4-chlorophenyl)(oxetan-3-yl)(2,4,6-trimethoxyphenyl)sulfonium tetrafluoroborate **2** (78 mg, 0.17 mmol). The reaction mixture was concentrated under reduced pressure to afford the crude material with a quantitative $^1$H-NMR yield of 30%. Purification by flash column chromatography (50% dichloromethane/hexane to 40% EtOAc/hexane, 1% $Et_3N$) affords the title compound **29** (27 mg, 0.029 mmol, 22%) as a yellow oil.

*Note: in this case, addition of MeOH to quench the reaction can lead to LiHMDS-mediated transesterification to the corresponding methyl ester and is therefore avoided.[7] Upon completion of the reaction, the mixture is concentrated under reduced pressure.*

**$^1$H-NMR** (400 MHz, $CDCl_3$): $\delta_H$ 8.26 (2H, d, *J* = 8.4 Hz), 7.94 – 7.81 (3H, m), 7.70 (1H, d, *J* = 2.3 Hz), 7.54 – 7.45 (4H, m), 7.36 (1H, dd, *J* = 8.9, 2.4 Hz), 5.03 – 4.97 (2H, m), 4.93 – 4.88 (1H, m), 4.81 (1H, d, *J* = 1.2 Hz), 2.79 (1H, d, *J* = 2.1 Hz), 1.90 (1H, d, *J* = 2.0 Hz).

**$^{13}$C{$^1$H}-NMR** (101 MHz, $CDCl_3$): $\delta_C$ 164.9, 148.6, 138.8, 134.0, 131.7, 130.6, 129.9, 129.7, 128.7, 128.0, 127.8, 126.8, 126.0, 121.2, 118.8, 77.2, 76.5, 70.7, 50.1, 38.4.

**IR** (neat, *v* cm$^{-1}$): 2934, 1716, 1479, 1264, 1044.

**HRMS**: *exact mass not found in ESI+ or ESI- mode*

**Oxetan-3-yl 4-(1-azaspiro[bicyclo[1.1.0]butane-2,3'-oxetan]-3-yl)benzoate, 30**

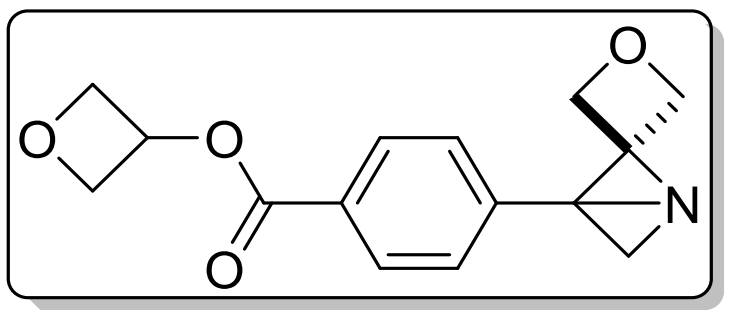

Prepared following a modification of **General Procedure 5** from oxetan-3-yl 4-(2*H*-azirin-3-yl)benzoate **P** (43 mg, 0.20 mmol) and (4-chlorophenyl)(oxetan-3-yl)(2,4,6-trimethoxyphenyl)sulfonium tetrafluoroborate **2** (120 mg, 0.26 mmol). The reaction mixture was concentrated under reduced pressure to afford the crude material with a quantitative $^1$H-NMR yield of 43%. Purification by flash column chromatography (5-40% EtOAc/hexane, 1% $Et_3N$) affords the title compound **30** (10 mg, 0.037 mmol, 18%) as a yellow oil.

*Note: in this case, addition of MeOH to quench the reaction can lead to LiHMDS-mediated transesterification to the corresponding methyl ester and is therefore avoided.*[7] *Upon completion of the reaction, the mixture is concentrated under reduced pressure.*

**$^1$H-NMR** (400 MHz, $CDCl_3$): $\delta_H$ 8.08 – 8.04 (2H, m), 7.47 – 7.40 (2H, m), 5.68 (1H, tt, *J* = 6.4, 5.2 Hz), 4.98 (4H, m), 4.86 (1H, dd, *J* = 8.2, 1.1 Hz), 4.81 – 4.76 (3H, m), 2.75 (1H, d, *J* = 2.1 Hz), 1.87 (1H, d, *J* = 2.0 Hz).

**$^{13}$C{$^1$H}-NMR** (101 MHz, $CDCl_3$): $\delta_C$ 165.4, 138.6, 130.1, 129.6, 128.6, 77.7, 77.4, 76.5, 70.7, 68.7, 50.1, 38.3.

**IR** (neat, *v* cm$^{-1}$): 2954, 2874, 1717, 1269, 1121.

**HRMS** *exact mass not found in ESI+ or ESI- mode*

**6-Bromochroman-4-yl 4-(1-azaspiro[bicyclo[1.1.0]butane-2,3'-oxetan]-3-yl)benzoate, 31**

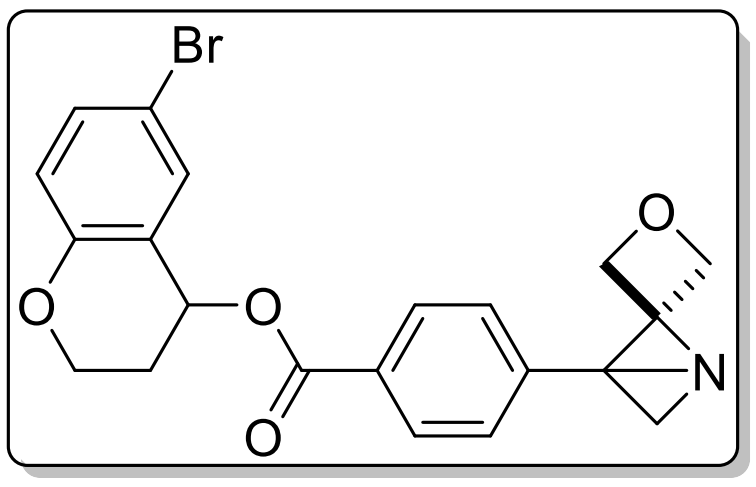

Prepared following a modification of **General Procedure 5** from 6-bromochroman-4-yl 4-(2*H*-azirin-3-yl)benzoate **Q** (45 mg, 0.12 mmol) and (4-chlorophenyl)(oxetan-3-yl)(2,4,6-trimethoxyphenyl)sulfonium tetrafluoroborate **2** (71 mg, 0.16 mmol). The reaction mixture was concentrated under reduced pressure to afford the crude material with a quantitative $^1$H-NMR yield of 50%. Purification by flash column chromatography (5-40% EtOAc/hexane, 1% $Et_3N$) affords the title compound **31** (15 mg, 0.035 mmol, 29%) as a yellow oil.

*Note: in this case, addition of MeOH to quench the reaction can lead to LiHMDS-mediated transesterification to the corresponding methyl ester and is therefore avoided.[7] Upon completion of the reaction, the mixture is concentrated under reduced pressure.*

**$^1$H-NMR** (400 MHz, $CDCl_3$): $\delta_H$ 8.04 (2H, d, *J* = 8.4 Hz), 7.48 (1H, d, *J* = 2.4 Hz), 7.43 – 7.36 (2H, m), 7.33 (1H, dd, *J* = 8.8, 2.5 Hz), 6.78 (1H, d, *J* = 8.8 Hz), 6.14 (1H, t, *J* = 3.9 Hz), 5.01 – 4.90 (2H, m), 4.84 (1H, m, *J* = 8.0, 2.4, 1.0 Hz), 4.79 – 4.75 (1H, m), 4.38 – 4.31 (1H, m), 4.25 (1H, d, *J* = 3.9 Hz), 2.73 – 2.71 (1H, m), 2.33 – 2.19 (2H, m), 1.85 (1H, d, *J* = 2.1 Hz).

**$^{13}$C{$^1$H}-NMR** (101 MHz, $CDCl_3$): $\delta_C$ 165.4, 154.6, 133.4, 133.2, 130.3, 130.1, 128.6, 128.5, 122.1, 119.2, 112.6, 76.4, 70.7, 65.6, 63.1, 62.5, 50.1, 38.4, 28.3.

**IR** (neat, *v* cm$^{-1}$): 2925, 1712, 1483, 1260, 1046.

**HRMS** (ESI+): *m/z* calcd for $C_{21}H_{18}{}^{81}BrNO_4Na$ $[M+Na]^+$ 452.0291, found 452.0278

**2-Isopropyl-5-methylcyclohexyl 4-(1-azaspiro[bicyclo[1.1.0]butane-2,3'-oxetan]-3-yl)benzoate, 32**

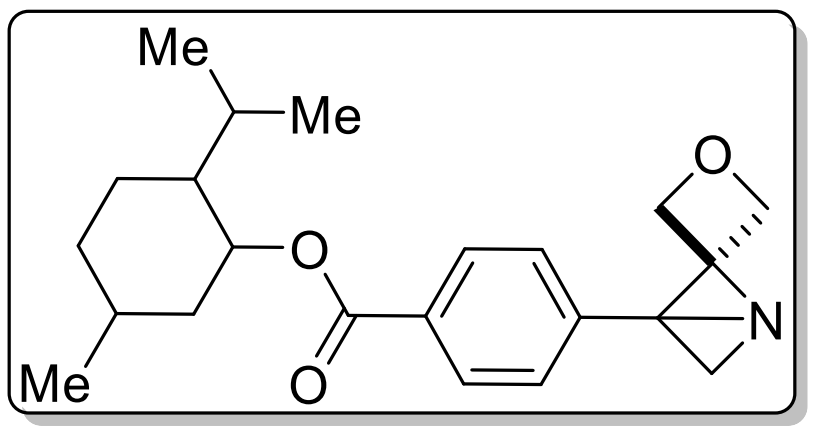


Prepared following **General Procedure 5** from 2-isopropyl-5-methylcyclohexyl 4-(2*H*-azirin-3-yl)benzoate **R** (41 mg, 0.14 mmol) and (4-chlorophenyl)(oxetan-3-yl)(2,4,6-trimethoxyphenyl)sulfonium tetrafluoroborate **2** (81 mg, 0.18 mmol). Work-up affords the crude material with a quantitative $^{1}$H-NMR yield of 75%. Purification by flash column chromatography (30% EtOAc/hexane, 1% $Et_3N$) affords the title compound **32** (15 mg, 0.042 mmol, 31%) as a colourless oil.

**$^{1}$H-NMR** (400 MHz, $CDCl_3$): $\delta_H$ 8.04 (2H, d, *J* = 8.1 Hz), 7.46 – 7.35 (2H, m), 5.02 – 4.93 (2H, m), 4.86 (1H, d, *J* = 8.0 Hz), 4.77 (1H, d, *J* = 7.0 Hz), 2.73 (1H, t, *J* = 1.8 Hz), 2.12 (1H,m), 1.94 (1H,m), 1.85 (1H, d, *J* = 2.1 Hz), 1.73 (2H,m), 1.56 (3H, m), 1.32 – 1.23 (1H, m), 1.19 – 1.04 (2H, m), 0.92 (6H, t, *J* = 6.5 Hz), 0.79 (3H, d, *J* = 6.9 Hz).

**$^{13}$C{$^{1}$H}-NMR** (101 MHz, $CDCl_3$): $\delta_C$ 165.7, 137.6, 129.9, 128.4, 128.4, 76.5, 75.3, 70.6, 50.1, 50.0, 47.4, 41.1, 38.5, 34.5, 31.6, 26.7, 23.8, 22.2, 20.9, 16.7.

**IR** (neat, *v* $cm^{-1}$): 2952, 2869, 1712, 1285, 1272.

**HRMS** (ESI+): *m/z* calcd for $C_{22}H_{29}NO_3Na$ $[M+Na]^+$ 378.2040, found 378.2015

**1,7,7-Trimethylbicyclo[2.2.1]heptan-2-yl 4-(1-azaspiro[bicyclo[1.1.0]butane-2,3'-oxetan]-3-yl)benzoate, 33**

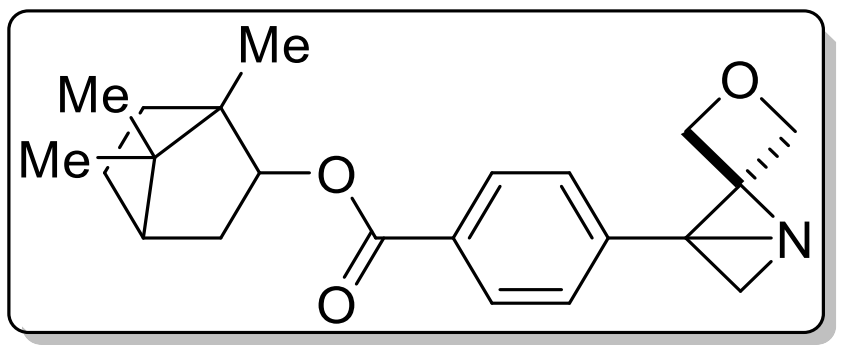


Prepared following **General Procedure 5** from 1,7,7-trimethylbicyclo[2.2.1]heptan-2-yl 4-(2*H*-azirin-3-yl)benzoate **S** (42 mg, 0.14 mmol) and (4-chlorophenyl)(oxetan-3-yl)(2,4,6-trimethoxyphenyl)sulfonium tetrafluoroborate **2** (83 mg, 0.18 mmol). Work-up affords the crude material with a quantitative $^{1}$H-NMR yield of 90%. Purification by preparative TLC (50% EtOAc/hexane, 1% $Et_3N$) affords the title compound **33** (16 mg, 0.045 mmol, 32%) as a colourless oil.

**$^{1}$H-NMR** (400 MHz, $CDCl_3$): $\delta_H$ 8.09 – 8.00 (2H,m), 7.44 – 7.36 (2H,m), 5.12 (1H, dt, *J* = 9.8, 2.9 Hz), 5.00 – 4.91 (2H, m), 4.86 (1H, dd, *J* = 8.0, 1.1 Hz), 4.81 – 4.74 (1H, m), 2.73 (1H, d, *J* = 2.1 Hz), 2.53 – 2.39 (1H, m), 2.11 (1H, m), 1.85 (1H, d, *J* = 2.1 Hz), 1.82 – 1.74 (2H, m), 1.41 (1H,m), 1.36 – 1.22 (1H, m), 1.12 (1H, dd, *J* = 13.8, 3.5 Hz), 0.97 (3H, s), 0.91 (6H, d, *J* = 3.2 Hz).

**$^{13}$C{$^{1}$H}-NMR** (101 MHz, $CDCl_3$): $\delta_C$ 166.4, 137.7, 131.3, 129.9, 128.5, 81.0, 76.5, 70.6, 50.0, 49.3, 48.1, 45.1, 38.4, 37.0, 28.2, 27.6, 19.9, 19.1, 13.8.

**IR** (neat, *v* cm$^{-1}$): 2951, 2872, 1714, 1272, 1116.

**HRMS** *exact mass not found in ESI+ or ESI- mode*

**3,4-Diphenyl-1-azaspiro[bicyclo[1.1.0]butane-2,3'-oxetane], 34**

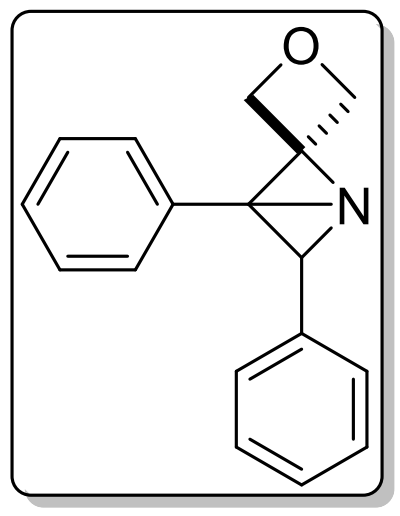

Prepared following **General Procedure 5** from 2,3-diphenyl-2*H*-azirine **AB** (39 mg, 0.20 mmol) and (4-chlorophenyl)(oxetan-3-yl)(2,4,6-trimethoxyphenyl)sulfonium tetrafluoroborate **2** (119 mg, 0.26 mmol). Work-up affords the crude material with a quantitative $^1$H-NMR yield of 90%. Purification by preparative TLC (70% dichloromethane/hexane, 1% $Et_3N$) affords the title compound **34** (10 mg, 0.040 mmol, 20%) as a colourless oil.

**$^1$H-NMR** (400 MHz, $CDCl_3$): $\delta_H$ 7.30 – 7.24 (3H, m), 7.18 (3H, m), 7.13 – 7.05 (4H, m), 5.09 – 5.03 (2H, m), 4.92 (1H, dd, *J* = 6.8, 1.1 Hz), 4.81 (1H, dd, *J* = 7.8, 1.1 Hz), 2.90 (1H, s).

**$^{13}$C{$^1$H}-NMR** (101 MHz, $CDCl_3$): $\delta_C$ 133.2, 129.5, 129.3, 128.6, 128.2, 127.6, 77.4, 76.5, 66.8, 61.6, 45.4.

**IR** (neat, *v* cm$^{-1}$): 2924, 2874, 2849, 1447, 971.

**HRMS** (ESI+): *m/z* calcd for $C_{17}H_{16}NO$ [M+H]$^+$ 250.1226, found 250.1225

**Naphthalen-2-yl 4-(1-azaspiro[bicyclo[1.1.0]butane-2,1'-cyclobutan]-3-yl)benzoate, 43**

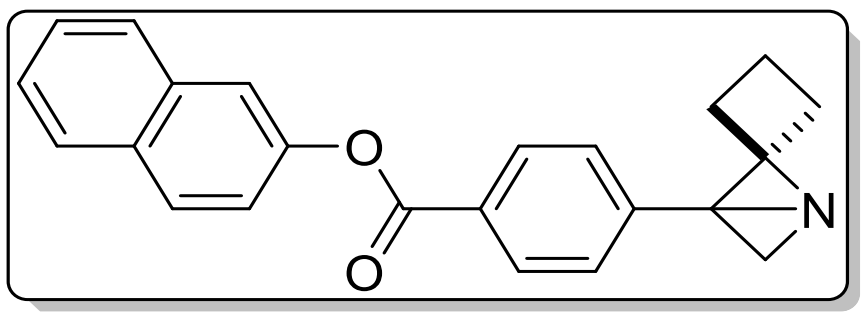

Prepared following a modification of **General Procedure 5** from naphthalen-2-yl 4-(2*H*-azirin-3-yl)benzoate **O** (57 mg, 0.20 mmol) and (cyclobutyl(phenyl)(2,4,6-trimethoxyphenyl)sulfonium tetrafluoroborate **3** (108 mg, 0.26 mmol). The reaction mixture was concentrated under reduced pressure to afford the crude material with a quantitative $^1$H-NMR yield of 44%. Purification by flash column chromatography (50% dichloromethane/hexane to 30% EtOAc/hexane) affords the title compound **43** (27 mg, 0.080 mmol, 40%) as a yellow oil.

*Note: in this case, addition of MeOH to quench the reaction can lead to LiHMDS-mediated transesterification to the corresponding methyl ester and is therefore avoided.[7] Upon completion of the reaction, the reaction mixture is concentrated under reduced pressure.*

**$^1$H-NMR** (400 MHz, $CDCl_3$): $\delta_H$ 8.23 (2H, d, *J* = 8.3 Hz), 7.92 – 7.80 (3H, m), 7.70 (1H, d, *J* = 2.2 Hz), 7.58 (1H, d, *J* = 8.5 Hz), 7.54 (2H, d, *J* = 8.3 Hz), 7.50 (1H, m), 7.36 (1H, m), 2.68 (1H, d, *J* = 2.4 Hz), 2.50 – 2.34 (3H, m), 2.25 – 2.15 (1H, m), 2.07 (1H, m), 2.01 (1H, d, *J* = 2.4 Hz), 1.93 – 1.79 (1H, m).

**$^{13}$C{$^1$H}-NMR** (101 MHz, $CDCl_3$): $\delta_C$ 165.2, 148.7, 144.5, 141.1, 134.0, 131.7, 130.3, 129.6, 129.0, 127.9, 127.8, 126.7, 125.9, 121.3, 118.8, 72.8, 49.5, 39.6, 28.5, 26.1, 14.4.

**IR** (neat, *v* cm$^{-1}$): 2949, 2871, 1715, 1263, 1172.

**HRMS** (ESI+): *m/z* calcd for $C_{23}H_{19}NO_2Na$ [M+Na]$^+$ 364.1308, found 364.1299

**Oxetan-3-yl 4-(1-azaspiro[bicyclo[1.1.0]butane-2,1'-cyclobutan]-3-yl)benzoate, 44**

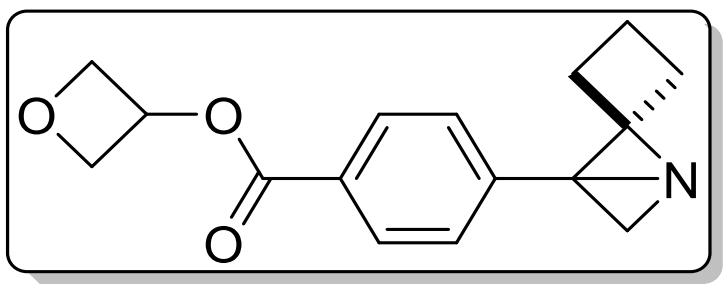

Prepared following a modification of **General Procedure 5** from oxetan-3-yl 4-(2*H*-azirin-3-yl)benzoate **P** (43 mg, 0.20 mmol) and (cyclobutyl(phenyl)(2,4,6-trimethoxyphenyl)sulfonium tetrafluoroborate **3** (108 mg, 0.26 mmol). The reaction mixture was concentrated under reduced pressure to afford the crude material with a quantitative $^{1}$H-NMR yield of 47%. Purification by flash column chromatography (50% dichloromethane/hexane to 40% EtOAc/hexane) affords the title compound **44** (8 mg, 0.037 mmol, 19%) as a yellow oil.

*Note: in this case, addition of MeOH to quench the reaction can lead to LiHMDS-mediated transesterification to the corresponding methyl ester and is therefore avoided.[7] Upon completion of the reaction, the reaction mixture is concentrated under reduced pressure.*

**$^{1}$H-NMR** (400 MHz, $CDCl_3$): $\delta_H$ 8.04 (2H, d, *J* = 8.4 Hz), 7.47 (2H, d, *J* = 8.4 Hz), 5.67 (1H, m), 5.00 (2H, m), 4.83 – 4.72 (2H, m), 2.64 (1H, d, *J* = 2.4 Hz), 2.49 – 2.29 (3H, m), 2.25 – 2.12 (1H, m), 2.09 – 1.90 (2H, m), 1.90 – 1.76 (1H, m).

**$^{13}$C{$^{1}$H}-NMR** (101 MHz, $CDCl_3$): $\delta_C$ 165.6, 140.9, 129.8, 128.9, 128.9, 77.8, 72.8, 68.6, 49.5, 39.5, 28.5, 26.1, 14.4.

**IR** (neat, *v* cm$^{-1}$): 2952, 2880, 1717, 1269, 1123.

**HRMS** *exact mass not found in ESI+ or ESI- mode*

**2-Isopropyl-5-methylcyclohexyl 4-(1-azaspiro[bicyclo[1.1.0]butane-2,1'-cyclobutan]-3-yl)benzoate, 45**

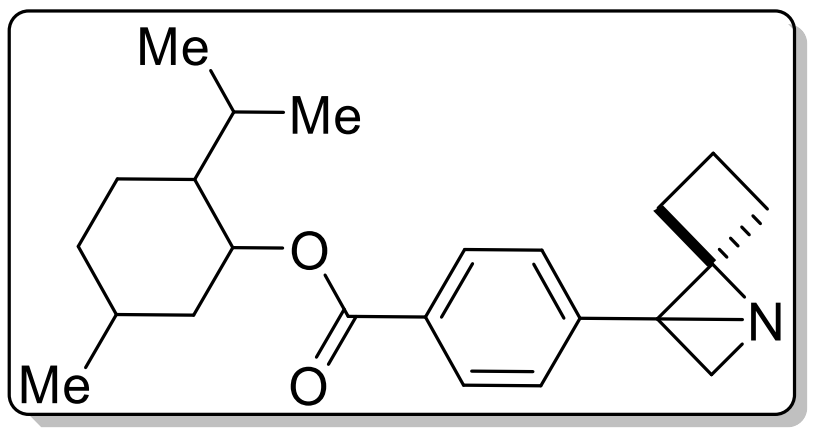


Prepared following **General Procedure 5** from 2-isopropyl-5-methylcyclohexyl 4-(2*H*-azirin-3-yl)benzoate **R** (41 mg, 0.14 mmol) and (cyclobutyl(phenyl)(2,4,6-trimethoxyphenyl)sulfonium tetrafluoroborate **3** (75 mg, 0.18 mmol). Work-up affords the crude material with a quantitative $^{1}$H-NMR yield of 58%. Purification by flash column chromatography (13% EtOAc/hexane) affords the title compound **45** (14 mg, 0.040 mmol, 29%) as a yellow oil.

**$^{1}$H-NMR** (400 MHz, $CDCl_3$): $\delta_H$ 8.01 (2H, d, *J* = 8.4 Hz), 7.43 (2H, d, *J* = 8.3 Hz), 4.93 (1H, td, *J* = 10.9, 4.4 Hz), 2.61 (1H, dd, *J* = 2.5, 1.2 Hz), 2.35 (3H,m), 2.21 – 2.07 (2H, m), 2.05 – 1.89 (3H, m), 1.87 – 1.79 (1H, m), 1.77 – 1.70 (2H, m), 1.62 – 1.51 (2H, m), 1.17 – 1.02 (2H, m), 0.92 (7H, t, *J* = 6.4 Hz), 0.79 (3H, d, *J* = 6.9 Hz).

**$^{13}$C{$^{1}$H}-NMR** (101 MHz, $CDCl_3$): $\delta_C$ 165.8, 139.3, 130.4, 129.5, 128.6, 74.9, 74.7, 72.5, 49.4, 49.3, 47.3, 41.0, 39.5, 34.3, 31.5, 28.3, 26.6, 25.9, 23.7, 22.0, 20.8, 16.6, 14.2.

**IR** (neat, *v* cm$^{-1}$): 2952, 2868, 1710, 1267, 1174.

**HRMS** (ESI+): *m/z* calcd for $C_{23}H_{31}NO_2Na$ $[M+Na]^+$ 376.2247, found 376.2245

**1,7,7-Trimethylbicyclo[2.2.1]heptan-2-yl 4-(1-azaspiro[bicyclo[1.1.0]butane-2,1'-cyclobutan]-3-yl)benzoate, 46**

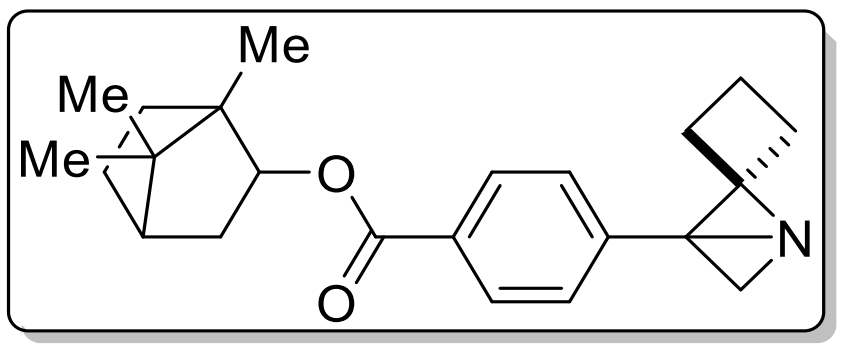


Prepared following **General Procedure 5** from 1,7,7-trimethylbicyclo[2.2.1]heptan-2-yl 4-(2*H*-azirin-3-yl)benzoate **S** (42 mg, 0.14 mmol) and (cyclobutyl(phenyl)(2,4,6-trimethoxyphenyl)sulfonium tetrafluoroborate **3** (77 mg, 0.18 mmol). Work-up affords the crude material with a quantitative $^1$H-NMR yield of 87%. Purification by flash column chromatography (12% EtOAc/hexane) affords the title compound **46** (14 mg, 0.048 mmol, 34%) as a colourless oil.

**$^1$H-NMR** (400 MHz, $CDCl_3$): $\delta_H$ 8.02 (2H, m), 7.47 – 7.42 (2H, m), 5.11 (1H, ddd, *J* = 9.9, 3.5, 2.2 Hz), 2.62 (1H, d, *J* = 2.5 Hz), 2.51 – 2.27 (4H, m), 2.22 – 2.07 (2H, m), 2.05 – 1.92 (2H, m), 1.88 – 1.76 (2H, m), 1.75 – 1.70 (1H, m), 1.45 – 1.36 (1H, m), 1.30 (1H, ddd, *J* = 12.0, 9.5, 4.5 Hz), 1.16 – 1.08 (1H, m), 0.96 (3H. s), 0.91 (6H, d, *J* = 2.6 Hz).

**$^{13}$C{$^1$H}-NMR** (101 MHz, $CDCl_3$): $\delta_C$ 166.6, 139.9, 130.5, 129.6, 128.7, 80.7, 72.7, 49.5, 49.2, 48.0, 45.1, 39.6, 37.0, 28.4, 28.2, 27.5, 26.1, 19.9, 19.0, 14.3, 13.7.

**IR** (neat, $\nu$ cm$^{-1}$): 2951, 1712, 1300, 1269, 1114.

**HRMS** (ESI+): *m/z* calcd for $C_{23}H_{29}NO_2Na$ $[M+Na]^+$ 374.2091, found 374.2081

**3,4-Diphenyl-1-azaspiro[bicyclo[1.1.0]butane-2,1'-cyclobutane], 47**

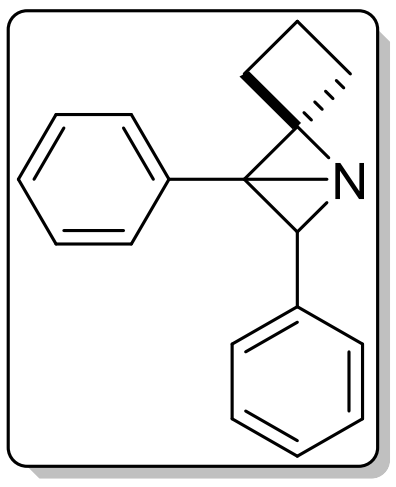

Prepared following **General Procedure 5** from 2,3-diphenyl-2*H*-azirine **AB** (39 mg, 0.20 mmol) and (cyclobutyl(phenyl)(2,4,6- trimethoxyphenyl)sulfonium tetrafluoroborate **3** (110 mg, 0.26 mmol). Work-up affords the crude material with a quantitative $^1$H-NMR yield of 65%. Purification by flash column chromatography (5-15% EtOAc/hexane, 1% $Et_3N$) affords the title compound **47** (22 mg, 0.089 mmol, 44%) as a white solid.

A sample suitable for x-ray crystallography was obtained by slow evaporation from ethyl acetate.

**$^1$H-NMR** (400 MHz, $CDCl_3$): $\delta_H$ 7.42 – 7.19 (5H, m), 7.19 – 7.01 (5H, m), 3.02 (1H, s), 2.59 – 2.43 (2H, m), 2.38 – 2.21 (2H, m), 2.09 – 1.98 (1H, m), 1.89 – 1.73 (1H, m).

**$^{13}$C{$^1$H}-NMR** (101 MHz, $CDCl_3$): $\delta_C$ 134.4, 131.1, 129.9, 128.1, 127.9, 127.8, 127.6, 127.6, 68.5, 60.7, 47.1, 27.9, 26.4, 14.2.

**IR** (neat, *v* cm$^{-1}$): 2952, 1604, 1305, 748, 694.

**HRMS** (ESI+): *m/z* calcd for $C_{18}H_{17}NNa$ [M+Na]$^+$ 270.1253, found 270.1228

**Melting Point**: decomposition observed from ca. 122 °C

**tert-Butyl 3-chloro-3-(4-fluorophenyl)-6-tosyl-1,6-diazaspiro[3.3]heptane-1-carboxylate, 55**

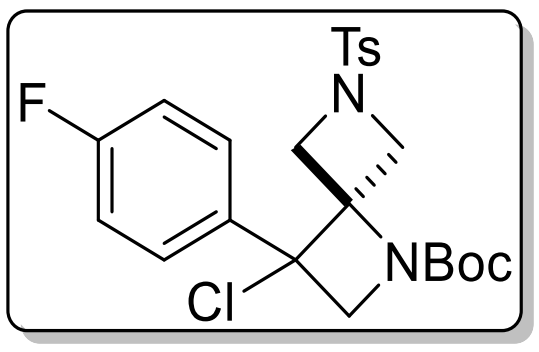


Prepared following **General Procedure 6** from 3-(4-fluorophenyl)-2*H*-azirine **J** (38 mg, 0.28 mmol) and (4-chlorophenyl)(1-tosylazetidin-3-yl)(2,4,6-trimethoxyphenyl)sulfonium tetrafluoroborate **1** (222 mg, 0.37 mmol). Work-up affords the crude material with a quantitative $^{1}$H-NMR yield of 51% over two steps. Purification by flash column chromatography (50% dichloromethane/hexane to 20% EtOAc/hexane) affords the title compound **55** (22 mg, 0.050 mmol, 17%) as a yellow oil.

**$^{1}$H-NMR** (400 MHz, $CDCl_3$): $\delta_H$ 7.65 (2H, d, *J* = 8.0 Hz), 7.34 (2H, d, *J* = 8.0 Hz), 7.18 – 7.11 (2H, m), 7.01 (2H, t, *J* = 8.2 Hz), 4.54 (1H, d, *J* = 9.5 Hz), 4.43 (1H, d, *J* = 9.6 Hz), 4.35 (1H, d, *J* = 9.8 Hz), 4.17 (1H, d, *J* = 9.5 Hz), 3.96 (1H, d, *J* = 9.4 Hz), 3.21 (1H, d, *J* = 9.0 Hz), 2.49 (3H, s), 1.46 (9H, m).

*Note: Signal of the Boc-group is split due to the presence of rotamers.*

**$^{19}$F-NMR** (377 MHz, $CDCl_3$): $\delta_F$ -111.87 (s).

**$^{13}$C{$^{1}$H}-NMR** (101 MHz, $CDCl_3$): $\delta_C$ 162.8 (d, *J* = 250.1 Hz), 153.9, 144.3, 133.0 (br. s), 129.9, 128.4 (d, *J* = 9.2 Hz), 128.3, 127.2, 116.0 (d, *J* = 21.8 Hz), 82.2, 71.8, 68.1, 61.2, 58.3, 57.6, 28.5, 21.7.

**IR** (neat, *v* cm$^{-1}$): 2982, 1705, 1391, 1159, 1092.

**HRMS** (ESI+): *m/z* calcd for $C_{23}H_{26}{}^{35}ClFN_2O_4SNa$ $[M+Na]^+$ 503.1178, found 503.1145

**_tert_-Butyl 3-(4-bromophenyl)-3-chloro-6-tosyl-1,6-diazaspiro[3.3]heptane-1-carboxylate, 56**

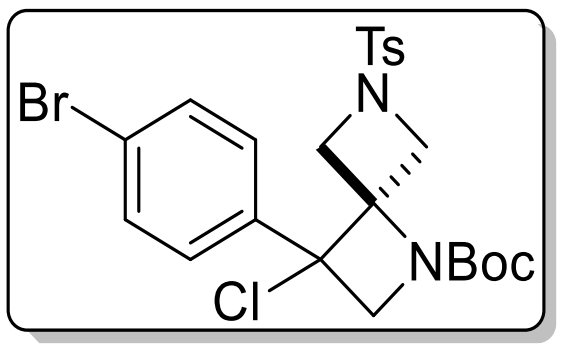


Prepared following **General Procedure 6** from 3-(4-bromophenyl)-2*H*-azirine **N** (55 mg, 0.28 mmol) and (4-chlorophenyl)(1-tosylazetidin-3-yl)(2,4,6-trimethoxyphenyl)sulfonium tetrafluoroborate **1** (221 mg, 0.36 mmol). Work-up affords the crude material with a quantitative $^{1}$H-NMR yield of 61% over two steps. Purification by flash column chromatography (50% dichloromethane/hexane to 20% EtOAc/hexane) affords the title compound **56** (40 mg, 0.074 mmol, 26%) as a yellow oil.

**$^{1}$H-NMR** (400 MHz, $CDCl_3$): $\delta_H$ 7.64 (2H, d, *J* = 7.9 Hz), 7.46 (2H, d, *J* = 8.1 Hz), 7.34 (2H, d, *J* = 8.0 Hz), 7.03 (2H, s), 4.51 (1H, d, *J* = 9.5 Hz), 4.41 (1H, d, *J* = 9.9 Hz), 4.36 (1H, s), 4.16 (1H, d, *J* = 9.5 Hz), 3.96 (1H, d, *J* = 9.3 Hz), 3.24 – 3.15 (1H, m), 2.49 (3H, s), 1.51 – 1.41 (9H, m).

**$^{13}$C{$^{1}$H}-NMR** (101 MHz, $CDCl_3$): $\delta_C$ 153.8, 144.3, 136.1, 132.2, 129.9, 128.5, 128.4, 128.0, 123.4, 82.3, 71.7, 68.2, 61.1, 58.2, 57.3, 28.5, 21.8.

**IR** (neat, *v* $cm^{-1}$): 2975, 1705, 1394, 1157, 1091.

**HRMS** (ESI+): *m/z* calcd for $C_{23}H_{26}{}^{79}Br^{35}ClN_2O_4SNa$ $[M+Na]^+$ 563.0377, found 563.0332

**tert-Butyl 3-chloro-3-(4-fluorophenyl)-6-oxa-1-azaspiro[3.3]heptane-1-carboxylate, 57**

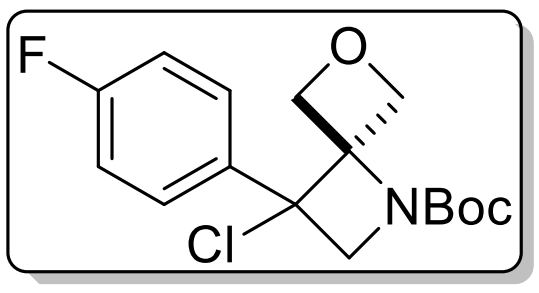


Prepared following **General Procedure 6** from 3-(4-fluorophenyl)-2*H*-azirine **J** (38 mg, 0.28 mmol) and (4-chlorophenyl)(oxetan-3-yl)(2,4,6-trimethoxyphenyl)sulfonium tetrafluoroborate **2** (166 mg, 0.36 mmol). Work-up affords the crude material with a quantitative $^{1}$H-NMR yield of 88% over two steps. Purification by flash column chromatography (50% dichloromethane/hexane to 20% EtOAc/hexane) affords the title compound **57** (80 mg, 0.19 mmol, 70%) as a yellow oil.

*Note: The $^{1}$H-NMR spectrum of the crude material of the 1-azabicyclo[1.1.0]butane intermediate, with the key product signals integrated, is included in the section:* ***13. NMR spectra***

**$^{1}$H-NMR** (400 MHz, $CDCl_3$): $\delta_H$ 7.54 – 7.41 (2H, m), 7.16 – 7.06 (2H, m), 5.19 (2H, d, *J* = 6.6 Hz), 4.81 (1H, s), 4.56 (1H, d, *J* = 9.4 Hz), 4.22 (1H, d, *J* = 9.5 Hz), 4.08 (1H, d, *J* = 7.5 Hz), 1.54 (9H, s).

**$^{19}$F-NMR** (377 MHz, $CDCl_3$): $\delta_F$ -112.21 (s).

**$^{13}$C{$^{1}$H}-NMR** (101 MHz, $CDCl_3$): $\delta_C$ 162.8 (d, *J* = 249.6 Hz), 154.4, 133.8 (d, *J* = 3.5 Hz), 128.5 (d, *J* = 8.3 Hz), 116.1 (d, *J* = 21.9 Hz), 81.7, 78.3, 77.4, 68.5, 61.1, 28.6.

**IR** (neat, *v* cm$^{-1}$): 2975, 1701, 1511, 1389, 1150.

**HRMS** (ESI+): *m/z* calcd for $C_{16}H_{19}{}^{35}ClFNO_3Na$ $[M+Na]^+$ 350.0930, found 350.0938

***tert*-Butyl 3-chloro-3-(4-(trifluoromethyl)phenyl)-6-oxa-1-azaspiro[3.3]heptane-1-carboxylate, 58**

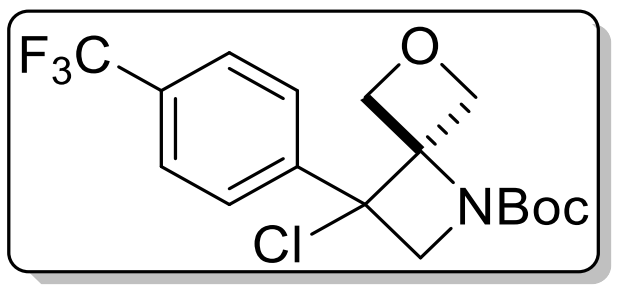


Prepared following **General Procedure 6** from 3-(4-(trifluoromethyl)phenyl)-2*H*-azirine **K** (39 mg, 0.21 mmol) and (4-chlorophenyl)(oxetan-3-yl)(2,4,6-trimethoxyphenyl)sulfonium tetrafluoroborate **2** (125 mg, 0.27 mmol). Work-up affords the crude material with a quantitative $^{1}$H-NMR yield of 85% over two steps. Purification by flash column chromatography (50% dichloromethane/hexane to 20% EtOAc/hexane) affords the title compound **58** (52 mg, 0.14 mmol, 65%) as a yellow oil.

*Note: The $^{1}$H-NMR spectrum of the crude material of the 1-azabicyclo[1.1.0]butane intermediate, with the key product signals integrated, is included in the section:* ***13. NMR spectra***

**$^{1}$H-NMR** (400 MHz, $CDCl_3$): $\delta_H$ 7.72 (2H, d, *J* = 8.5 Hz,), 7.64 (2H, d, *J* = 8.3 Hz), 5.20 (2H, d, *J* = 6.7 Hz), 4.82 (1H, s), 4.58 (1H, d, *J* = 9.5 Hz), 4.26 (1H, d, *J* = 9.5 Hz), 4.03 (1H, d, *J* = 7.7 Hz), 1.54 (9H, m).

*Note: Signal of the Boc-group is split due to the presence of rotamers.*

**$^{19}$F-NMR** (377 MHz, $CDCl_3$): $\delta_F$ -62.84 (s).

**$^{13}$C{$^{1}$H}-NMR** (101 MHz, $CDCl_3$): $\delta_C$ 154.4, 141.7, 131.3 (q, *J* = 33.0 Hz), 126.9, 126.2 (q, *J* = 3.7 Hz), 123.8 (q, *J* = 272.4 Hz), 81.8, 78.2, 77.3, 68.0, 61.2, 28.6.

**IR** (neat, *v* cm$^{-1}$): 2977, 1702, 1323, 1153, 1120.

**HRMS** (ESI+): *m/z* calcd for $C_{17}H_{19}{}^{35}ClF_3NO_3Na$ $[M+Na]^+$ 400.0898, found 400.0904

**_tert_-Butyl 3-chloro-3-(4-chlorophenyl)-6-oxa-1-azaspiro[3.3]heptane-1-carboxylate, 59**

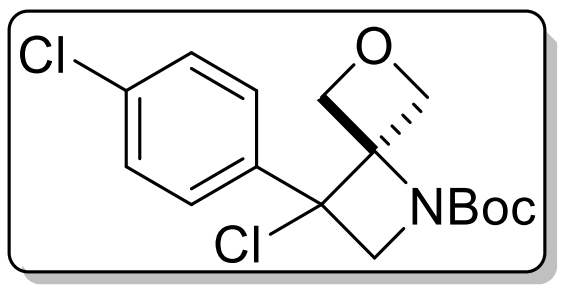


Prepared following **General Procedure 6** from 3-(4-chlorophenyl)-2*H*-azirine **L** (42 mg, 0.28 mmol) and (4-chlorophenyl)(oxetan-3-yl)(2,4,6-trimethoxyphenyl)sulfonium tetrafluoroborate **2** (164 mg, 0.36 mmol). Work-up affords the crude material with a quantitative $^1$H-NMR yield of 84% over two steps. Purification by flash column chromatography (18% EtOAc/hexane) affords the title compound **59** (60 mg, 0.17 mmol, 62%) as a colourless oil.

*Note: The $^1$H-NMR spectrum of the crude material of the 1-azabicyclo[1.1.0]butane intermediate, with the key product signals integrated, is included in the section:* ***13. NMR spectra***

**$^1$H-NMR** (400 MHz, $CDCl_3$): $\delta_H$ 7.53 – 7.38 (4H, m), 5.18 (2H, d, *J* = 6.8 Hz), 4.80 (1H, s), 4.54 (1H, d, *J* = 9.5 Hz), 4.22 (1H, d, *J* = 9.5 Hz), 4.07 (1H, d, *J* = 7.6 Hz), 1.54 (9H, s).

**$^{13}$C{$^1$H}-NMR** (101 MHz, $CDCl_3$): $\delta_C$ 154.7, 136.4, 135.1, 129.3, 127.9, 81.6, 78.2, 77.3, 68.3, 61.1, 28.6.

**IR** (neat, *v* cm$^{-1}$): 2975, 1700, 1388, 1151, 1093.

**HRMS** (ESI+): *m/z* calcd for $C_{16}H_{19}{}^{35}Cl_2NO_3Na$ [M+Na]$^+$ 366.0634, found 366.0641

**_tert_-Butyl 3-chloro-3-(3-chlorophenyl)-6-oxa-1-azaspiro[3.3]heptane-1-carboxylate, 60**

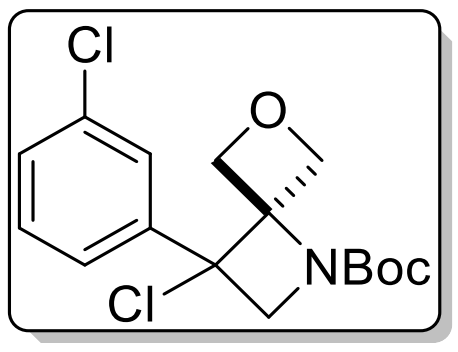


Prepared following **General Procedure 6** from 3-(3-chlorophenyl)-2*H*-azirine **M** (42 mg, 0.28 mmol) and (4-chlorophenyl)(oxetan-3-yl)(2,4,6-trimethoxyphenyl)sulfonium tetrafluoroborate **2** (164 mg, 0.36 mmol). Work-up affords the crude material with a quantitative $^1$H-NMR yield of 81% over two steps. Purification by flash column chromatography (18% EtOAc/hexane) affords the title compound **60** (48 mg, 0.14 mmol, 50%) as a colourless oil.

*Note**:** The $^1$H-NMR spectrum of the crude material of the 1-azabicyclo[1.1.0]butane intermediate, with the key product signals integrated, is included in the section: **13. NMR spectra***

**$^1$H-NMR** (400 MHz, $CDCl_3$): $\delta_H$ 7.49 (1H, m), 7.38 (3H, m), 5.18 (2H, d, *J* = 7.0 Hz), 4.81 (1H, s), 4.55 (1H, d, *J* = 9.5 Hz), 4.22 (1H, d, *J* = 9.5 Hz), 4.07 (1H, d, *J* = 7.7 Hz), 1.53 (9H, m).

*Note: Signal of the Boc-group is split due to the presence of rotamers.*

**$^{13}$C{$^1$H}-NMR** (101 MHz, $CDCl_3$): $\delta_C$ 154.4, 139.8, 135.3, 130.4, 129.3, 126.7, 124.6, 81.7, 78.3, 77.0, 68.1, 61.2, 28.6.

**IR** (neat, *v* cm$^{-1}$): 2974, 1704, 1391, 1367, 1154.

**HRMS** (ESI+): *m/z* calcd for $C_{16}H_{19}{}^{35}Cl_2NO_3Na$ $[M+Na]^+$ 366.0634, found 366.0636.

***tert*-Butyl 3-(4-bromophenyl)-3-chloro-6-oxa-1-azaspiro[3.3]heptane-1-carboxylate, 61**

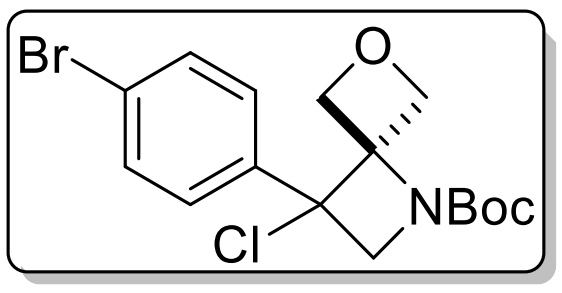

Prepared following **General Procedure 6** from 3-(4-bromophenyl)-2*H*-azirine **N** (41 mg, 0.21 mmol) and (4-chlorophenyl)(oxetan-3-yl)(2,4,6-trimethoxyphenyl)sulfonium tetrafluoroborate **2** (124 mg, 0.27 mmol). Work-up affords the crude material with a quantitative $^{1}$H-NMR yield of 72% over two steps. Purification by flash column chromatography (18% EtOAc/hexane) affords the title compound **61** (38 mg, 0.098 mmol, 46%) as a colourless oil.

*Note**:** The $^{1}$H-NMR spectrum of the crude material of the 1-azabicyclo[1.1.0]butane intermediate, with the key product signals integrated, is included in the section:* ***13. NMR spectra***

**$^{1}$H-NMR** (400 MHz, $CDCl_3$): $\delta_H$ 7.62 – 7.55 (2H, m), 7.39 – 7.33 (2H, m), 5.17 (2H, m), 4.90 – 4.70 (1H, m), 4.53 (1H, d, *J* = 9.5 Hz), 4.21 (1H, d, *J* = 9.5 Hz), 4.07 (1H, d, *J* = 7.6 Hz), 1.54 (9H, s).

**$^{13}$C{$^{1}$H}-NMR** (101 MHz, $CDCl_3$): $\delta_C$ 154.4, 136.9, 132.3, 128.1, 123.3, 81.7, 78.2, 77.3, 68.4, 66.0, 60.9, 28.6.

**IR** (neat, *v* cm$^{-1}$): 2974, 1701, 1389, 1367, 1152.

**HRMS** (ESI+): *m/z* calcd for $C_{16}H_{19}{}^{79}Br^{35}ClNO_3Na$ [M+Na]$^+$ 410.0129, found 410.0139

**tert-Butyl 3-chloro-3-(p-tolyl)-6-oxa-1-azaspiro[3.3]heptane-1-carboxylate, 62**

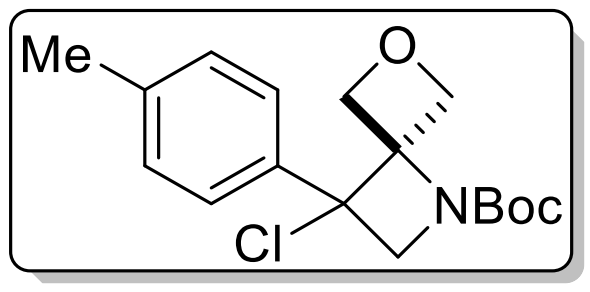


Prepared following **General Procedure 6** from 3-(p-tolyl)-2*H*-azirine **W** (37 mg, 0.28 mmol) and (4-chlorophenyl)(oxetan-3-yl)(2,4,6-trimethoxyphenyl)sulfonium tetrafluoroborate **2** (166 mg, 0.37 mmol). Work-up affords the crude material with a quantitative $^1$H-NMR yield of 64% over two steps. Purification by flash column chromatography (18% EtOAc/hexane) affords the title compound **62** (36 mg, 0.11 mmol, 40%) as a colourless oil.

*Note: The $^1$H-NMR spectrum of the crude material of the 1-azabicyclo[1.1.0]butane intermediate, with the key product signals integrated, is included in the section:* ***13. NMR spectra***

**$^1$H-NMR** (400 MHz, $CDCl_3$) $\delta_H$: 7.42 – 7.31 (2H, m), 7.24 (2H, d, *J* = 8.1 Hz), 5.18 (2H, m), 4.84 – 4.73 (1H, m), 4.59 (1H, d, *J* = 9.4 Hz), 4.21 (1H, d, *J* = 9.4 H), 4.12 (1H, d, *J* = 7.5 Hz), 2.37 (3H, s), 1.54 (9H, s).

**$^{13}$C{$^1$H}-NMR** (101 MHz, $CDCl_3$): $\delta_C$ 154.6, 139.0, 134.8, 129.7, 126.3, 81.5, 78.3, 77.4, 69.1, 64.9, 28.6, 21.2.

**IR** (neat, *v* cm$^{-1}$): 2974, 1701, 1388, 1150, 1064.

**HRMS** (ESI+): *m/z* calcd for $C_{17}H_{22}{}^{35}ClNO_3Na$ [M+Na]$^+$ 346.1180, found 346.1183.

**_tert_-Butyl 3-chloro-3-(4-fluorophenyl)-1-azaspiro[3.3]heptane-1-carboxylate, 63**

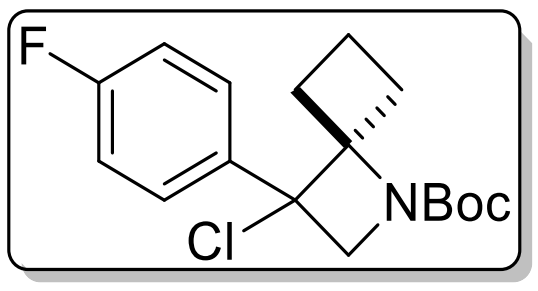

Prepared following **General Procedure 6** from 3-(4-fluorophenyl)-2*H*-azirine **J** (26 mg, 0.19 mmol) and (cyclobutyl(phenyl)(2,4,6- trimethoxyphenyl)sulfonium tetrafluoroborate **3** (105 mg, 0.25 mmol). Work-up affords the crude material with a quantitative $^1$H-NMR yield of 55% over two steps. Purification by flash column chromatography (5-10% $Et_2O$/hexane) affords the title compound **63** (23 mg, 0.070 mmol, 37%) as a yellow oil.

*Note: The $^1$H-NMR spectrum of the crude material of the 1-azabicyclo[1.1.0]butane intermediate, with the key product signals integrated, is included in the section:* ***13. NMR spectra***

**$^1$H-NMR** (400 MHz, $CDCl_3$): $\delta_H$ 7.46 (2H, m), 7.09 (2H, m), 4.55 (1H, d, *J* = 9.6 Hz), 4.18 (1H, d, *J* = 9.6 Hz), 2.75 (2H, m), 2.50 – 2.19 (1H, m), 1.88 (1H, m), 1.61 (2H, m), 1.51 (9H, s).

**$^{19}$F-NMR** (377 MHz, $CDCl_3$): $\delta_F$ -113.63 (s).

**$^{13}$C{$^1$H}-NMR** (101 MHz, $CDCl_3$): $\delta_C$ 162.5 (d, *J* = 248.3 Hz), 155.4, 135.1 (d, *J* = 3.3 Hz), 128.5 (d, *J* = 8.3 Hz), 115.6 (d, *J* = 21.8 Hz), 80.6, 78.8, 70.7, 60.4, 31.1, 30.4, 28.7, 12.3.

**IR** (neat, *v* $cm^{-1}$): 2975, 1697, 1511, 1387, 1154.

**HRMS** (ESI+): *m/z* calcd for $C_{17}H_{21}{}^{35}ClFNO_2Na$ $[M+Na]^+$ 348.1137, found 348.1276

**tert-Butyl 3-chloro-3-(4-(trifluoromethyl)phenyl)-1-azaspiro[3.3]heptane-1-carboxylate, 64**

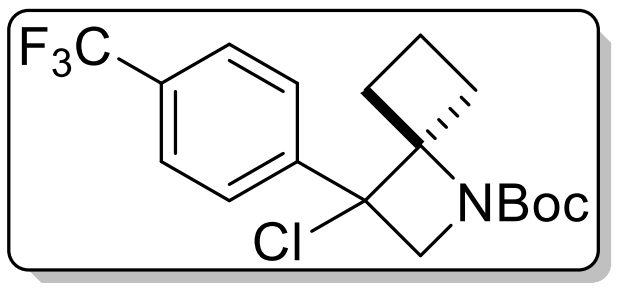


Prepared following **General Procedure 6** from 3-(4-(trifluoromethyl)phenyl)-2*H*-azirine **K** (35 mg, 0.19 mmol) and (cyclobutyl(phenyl)(2,4,6-trimethoxyphenyl)sulfonium tetrafluoroborate **3** (105 mg, 0.25 mmol). Work-up affords the crude material with a quantitative $^1$H-NMR yield of 53% over two steps. Purification by flash column chromatography (5-10% $Et_2O$/hexane) affords the title compound **64** (26 mg, 0.070 mmol, 37%) as a yellow oil.

*Note: The $^1$H-NMR spectrum of the crude material of the 1-azabicyclo[1.1.0]butane intermediate, with the key product signals integrated, is included in the section:* ***13. NMR spectra***

**$^1$H-NMR** (400 MHz, $CDCl_3$): $\delta_H$ 7.65 (4H, m), 4.56 (1H, d, *J* = 9.7 Hz), 4.22 (1H, d, *J* = 9.7 Hz), 2.77 (2H, m), 2.50 – 2.28 (1H, m), 1.97 – 1.80 (1H, m), 1.69 – 1.31 (11H, m).

**$^{19}$F-NMR** (377 MHz, $CDCl_3$): $\delta_F$ -62.72 (s).

**$^{13}$C{$^1$H}-NMR** (101 MHz, $CDCl_3$): $\delta_C$ 155.2, 143.1, 130.6 (q, *J* = 32.8 Hz), 127.1, 125.7 (q, *J* = 3.7 Hz), 124.0 (q, *J* = 272.3 Hz), 80.8, 78.8, 70.2, 60.6, 31.1, 30.5, 28.6, 12.4.

**IR** (neat, *v* $cm^{-1}$): 2976, 1698, 1325, 1157, 1127.

**HRMS** (ESI+): *m/z* calcd for $C_{18}H_{21}{}^{35}ClF_3NO_2Na$ $[M+Na]^+$ 398.1105, found 398.1109

**_tert_-Butyl 3-chloro-3-(3-chlorophenyl)-1-azaspiro[3.3]heptane-1-carboxylate, 65**

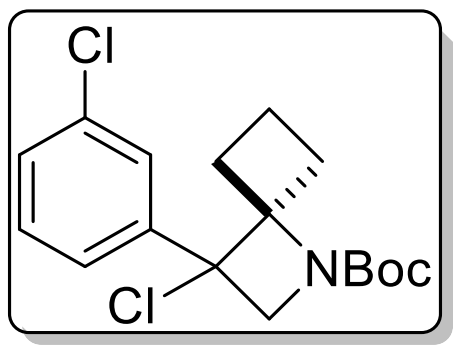


Prepared following **General Procedure 6** from 3-(3-chlorophenyl)-2*H*-azirine **M** (29 mg, 0.19 mmol) and (cyclobutyl(phenyl)(2,4,6- trimethoxyphenyl)sulfonium tetrafluoroborate **3** (105 mg, 0.25 mmol). Work-up affords the crude material with a quantitative $^1$H-NMR yield of 60% over two steps. Purification by flash column chromatography (5-10% $Et_2O$/hexane) affords the title compound **65** (19 mg, 0.056 mmol, 29%) as a colourless oil.

*Note**:** The $^1$H-NMR spectrum of the crude material of the 1-azabicyclo[1.1.0]butane intermediate, with the key product signals integrated, is included in the section: **13. NMR spectra***

**$^1$H-NMR** (400 MHz, $CDCl_3$): $\delta_H$ 7.49 (1H, d, *J* = 2.0 Hz), 7.40 – 7.31 (3H, m), 4.54 (1H, d, *J* = 9.7 Hz), 4.18 (1H, d, *J* = 9.7 Hz), 2.75 (2H, m), 2.56 – 2.18 (1H, m), 1.85 (1H, m), 1.60 (2H, m), 1.52 (9H, s).

**$^{13}$C{$^1$H}-NMR** (101 MHz, $CDCl_3$): $\delta_C$ 155.3, 141.2, 134.8, 129.9, 128.6, 126.9, 124.8, 80.7, 78.8, 70.3, 60.4, 31.1, 30.5, 28.7, 12.4.

**IR** (neat, *v* cm$^{-1}$): 2975, 1697, 1255, 1553, 778.

**HRMS** (ESI+): *m/z* calcd for $C_{17}H_{21}{}^{35}Cl_2NO_2Na$ $[M+Na]^+$ 364.0842, found 364.0843

***tert*-Butyl 3-(4-bromophenyl)-3-chloro-1-azaspiro[3.3]heptane-1-carboxylate, 66**

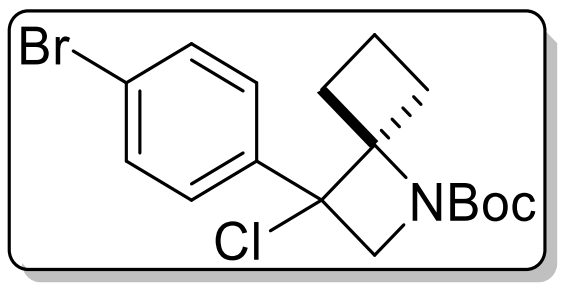

Prepared following **General Procedure 6** from 3-(4-bromophenyl)-2*H*-azirine **N** (36 mg, 0.18 mmol) and (cyclobutyl(phenyl)(2,4,6- trimethoxyphenyl)sulfonium tetrafluoroborate **3** (100 mg, 0.24 mmol). Work-up affords the crude material with a quantitative $^1$H-NMR yield of 71% over two steps. Purification by flash column chromatography (5-10% $Et_2O$/hexane) affords the title compound **66** (39 mg, 0.10 mmol, 55%) as a yellow oil.

*Note: The $^1$H-NMR spectrum of the crude material of the 1-azabicyclo[1.1.0]butane intermediate, with the key product signals integrated, is included in the section:* ***13. NMR spectra***

**$^1$H-NMR** (400 MHz, $CDCl_3$): $\delta_H$ 7.54 (2H, d, *J* = 8.7 Hz), 7.36 (2H, d, *J* = 8.6 Hz), 4.52 (1H, d, *J* = 9.7 Hz), 4.18 (1H, d, *J* = 9.7 Hz), 2.75 (2H, m), 2.55 – 2.20 (1H, m), 1.88 (1H, m), 1.60 (2H, m), 1.51 (9H, s).

**$^{13}$C{$^1$H}-NMR** (101 MHz, $CDCl_3$): $\delta_C$ 155.3, 138.3, 131.8, 128.4, 122.6, 80.6, 78.7, 70.6, 60.4, 31.1, 30.5, 28.7, 12.4.

**IR** (neat, *v* cm$^{-1}$): 2974, 1698, 1389, 1366, 1156.

**HRMS** (ESI+): *m/z* calcd for $C_{17}H_{21}{}^{79}Br^{35}ClNO_2Na$ [M+Na]$^+$ 408.0336, found 408.0340

**_tert_-Butyl 3-chloro-3-(_p_-tolyl)-1-azaspiro[3.3]heptane-1-carboxylate, 67**

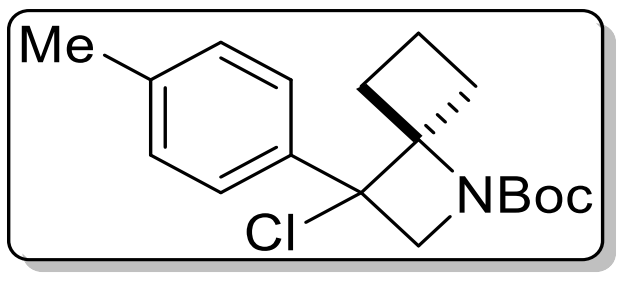


Prepared following **General Procedure 6** from 3-(*p*-tolyl)-2*H*-azirine **W** (37 mg, 0.28 mmol) and (cyclobutyl(phenyl)(2,4,6- trimethoxyphenyl)sulfonium tetrafluoroborate **3** (153 mg, 0.37 mmol). Work-up affords the crude material with a quantitative $^{1}$H-NMR yield of 78% over two steps. Purification by flash column chromatography (5-10% $Et_2O$/hexane) affords the title compound **67** (48 mg, 0.15 mmol, 56%) as a colourless oil.

*Note: The $^{1}$H-NMR spectrum of the crude material of the 1-azabicyclo[1.1.0]butane intermediate, with the key product signals integrated, is included in the section:* ***13. NMR spectra***

**$^{1}$H-NMR** (400 MHz, $CDCl_3$): $\delta_H$ 7.35 (2H, d, *J* = 8.3 Hz), 7.21 (2H, d, *J* = 8.2 Hz), 4.60 (1H, d, *J* = 9.6 Hz), 4.16 (1H, d, *J* = 9.6 Hz), 2.85 (1H, m), 2.67 (1H, m), 2.55 – 2.40 (1H, m), 2.36 (3H, s), 2.33 – 2.21 (1H, m), 1.96 – 1.79 (1H, m), 1.66 (1H, m), 1.52 (9H, m).

*Note: Signal of the Boc-group is split due to the presence of rotamers.*

**$^{13}$C{$^{1}$H}-NMR** (101 MHz, $CDCl_3$): $\delta_C$ 155.5, 138.2, 136.2, 129.3, 126.5, 80.4, 78.8, 71.6, 60.0, 31.1, 30.4, 28.7, 21.2, 12.3.

**IR** (neat, *v* $cm^{-1}$): 2974, 1697, 1385, 1153, 1056.

**HRMS** (ESI+): *m/z* calcd for $C_{18}H_{24}{}^{35}ClNO_2Na$ $[M+Na]^+$ 344.1388, found 344.1394

**_tert_-Butyl 3-chloro-3-(naphthalen-2-yl)-1-azaspiro[3.3]heptane-1-carboxylate, 68**

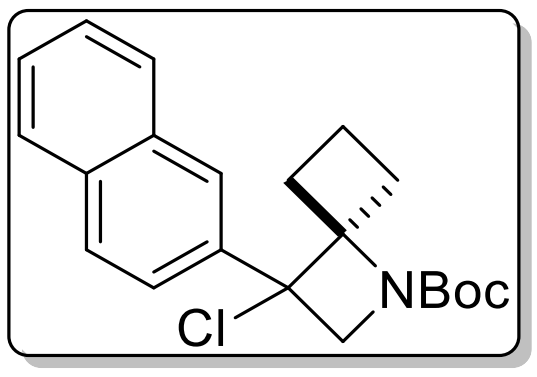


Prepared following **General Procedure 6** from 3-(naphthalen-2-yl)-2*H*-azirine **O** (32 mg, 0.19 mmol) and (cyclobutyl(phenyl)(2,4,6- trimethoxyphenyl)sulfonium tetrafluoroborate **3** (105 mg, 0.25 mmol). Work-up affords the crude material with a quantitative $^{1}$H-NMR yield of 66% over two steps. Purification by flash column chromatography (5-10% $Et_2O$/hexane) affords the title compound **68** (35 mg, 0.067 mmol, 51%) as a colourless oil.

*Note: The $^{1}$H-NMR spectrum of the crude material of the 1-azabicyclo[1.1.0]butane intermediate, with the key product signals integrated, is included in the section: **13. NMR spectra***

**$^{1}$H-NMR** (400 MHz, $CD_3OD$): $\delta_H$ 7.98 – 7.87 (3H, m), 7.87 – 7.84 (1H, m), 7.59 (1H, dd, *J* = 8.7, 2.1 Hz), 7.52 (2H, m), 4.87 – 4.85 (1H, m), 4.16 (1H, d, *J* = 10.0 Hz), 2.85 (2H, d, *J* = 85.3 Hz), 2.50 – 2.24 (1H, m), 1.88 (1H, s), 1.71 – 1.56 (2H, m), 1.52 (9H, s).

**$^{13}$C{$^{1}$H}-NMR** (101 MHz, $CD_3OD$): $\delta_C$ 157.1, 137.2, 134.3, 134.3, 129.6, 129.5, 128.6, 128.0, 127.8, 126.5, 125.6, 82.0, 79.9, 73.0, 60.4, 31.9, 31.2, 28.8, 13.1.

**IR** (neat, *v* $cm^{-1}$): 2972, 1696, 1389, 1365, 1154.

**HRMS** (ESI+): *m/z* calcd for $C_{21}H_{24}{}^{35}ClNO_2Na$ $[M+Na]^+$ 380.1388, found 380.1384

**_tert_-Butyl 3-chloro-3-(naphthalen-2-yl)-6-oxa-1-azaspiro[3.3]heptane-1-carboxylate, 69**

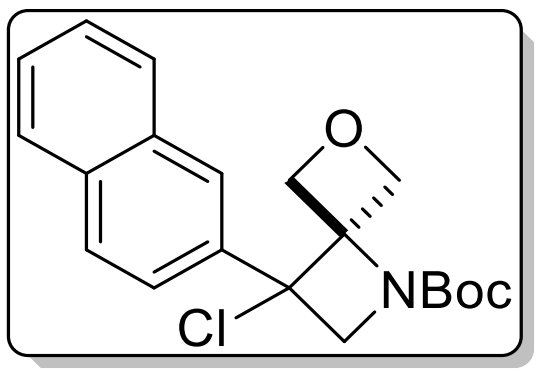


Prepared following **General Procedure 6** from 3-(naphthalen-2-yl)-2*H*-azirine **V** (47 mg, 0.28 mmol) and (4-chlorophenyl)(oxetan-3-yl)(2,4,6-trimethoxyphenyl)sulfonium tetrafluoroborate **2** (166 mg, 0.37 mmol). Work-up affords the crude material with a quantitative $^{1}$H-NMR yield of 83% over two steps. Purification by flash column chromatography (18% EtOAc /hexane) affords the title compound **69** (60 mg, 0.16 mmol, 60%) as a yellow oil.

*Note**:** The $^{1}$H-NMR spectrum of the crude material of the 1-azabicyclo[1.1.0]butane intermediate, with the key product signals integrated, is included in the section:* ***13. NMR spectra***

**$^{1}$H-NMR** (400 MHz, $CDCl_3$): $\delta_H$ 7.93 (1H, d, *J* = 8.7 Hz), 7.91 – 7.85 (3H, m), 7.61 (1H, dd, *J* = 8.6, 2.0 Hz), 7.58 – 7.52 (2H, m), 5.29 (1H, d, *J* = 7.5 Hz), 5.20 (1H, s), 4.78 (2H, d, *J* = 9.5 Hz), 4.31 (1H, d, *J* = 9.5 Hz), 4.12 (1H, d, *J* = 7.6 Hz), 1.56 (9H, s).

**$^{13}$C{$^{1}$H}-NMR** (101 MHz, $CDCl_3$): $\delta_C$ 133.2, 133.0, 129.3, 128.5, 127.8, 127.3, 127.1, 125.6, 124.0, 78.2, 77.1, 60.7, 28.7.

*Note: The signals for the carbonyl group and some quaternary carbons were not observed in the $^{13}$C{$^{1}$H}-NMR spectrum.*

**IR** (neat, *v* $cm^{-1}$): 2975, 1704, 1392, 1367, 1153.

**HRMS** (ESI+): *m/z* calcd for $C_{20}H_{22}{}^{35}ClNO_3Na$ $[M+Na]^+$ 382.1180, found 382.1157

**tert-Butyl 3-chloro-3-(naphthalen-2-yl)-6-tosyl-1,6-diazaspiro[3.3]heptane-1-carboxylate, 70**

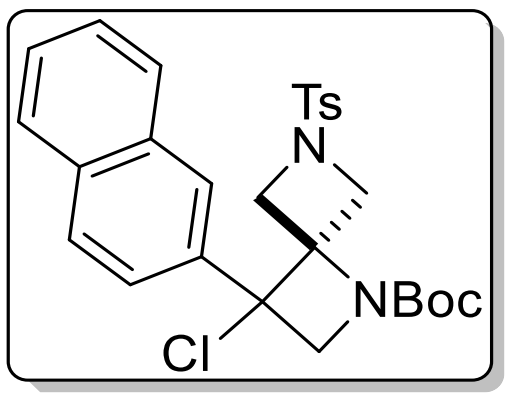


Prepared following **General Procedure 6** from 3-(naphthalen-2-yl)-2*H*-azirine **V** (47 mg, 0.28 mmol) and (4-chlorophenyl)(1-tosylazetidin-3-yl)(2,4,6-trimethoxyphenyl)sulfonium tetrafluoroborate **1** (222 mg, 0.37 mmol). Work-up affords the crude material with a quantitative $^{1}$H-NMR yield of 52% over two steps. Purification by flash column chromatography (50% dichloromethane/hexane to 20% EtOAc/hexane) affords the title compound **70** (48 mg, 0.094 mmol, 33%) as a yellow oil.

**$^{1}$H-NMR** (400 MHz, $CDCl_3$): $\delta_H$ 7.89 – 7.76 (3H, m), 7.68 (1H, s), 7.62 (2H, d, *J* = 8.0 Hz), 7.58 – 7.54 (2H, m), 7.29 (2H, d, *J* = 8.1 Hz),7.17 (1H, m) 4.74 (1H, d, *J* = 9.5 Hz), 4.50 (1H, d, *J* = 9.4 Hz), 4.42 (1H, d, *J* = 9.7 Hz), 4.26 (1H, d, *J* = 9.5 Hz), 3.99 (1H, d, *J* = 9.6 Hz), 3.26 (1H, s), 2.50 (3H, s), 1.51 (9H, s).

**$^{13}$C{$^{1}$H}-NMR** (101 MHz, $CDCl_3$): $\delta_C$ 154.1, 144.1, 133.1, 132.8, 129.9, 129.2, 128.5, 128.4, 127.8, 127.4, 127.1, 125.6, 123.7, 82.1, 71.9, 69.0, 60.5, 58.5, 57.5, 28.5, 21.8.

**IR** (neat, *v* cm$^{-1}$): 2982, 1795, 1704, 1347, 1157.

**HRMS** (ESI+): *m/z* calcd for $C_{27}H_{29}{}^{35}ClN_2O_4SNa$ $[M+Na]^+$ 535.1429, found 535.1440

**_tert_-Butyl 3-chloro-3-(2,2-difluorobenzo[_d_][1,3]dioxol-5-yl)-1-azaspiro[3.3]heptane-1-carboxylate, 71**

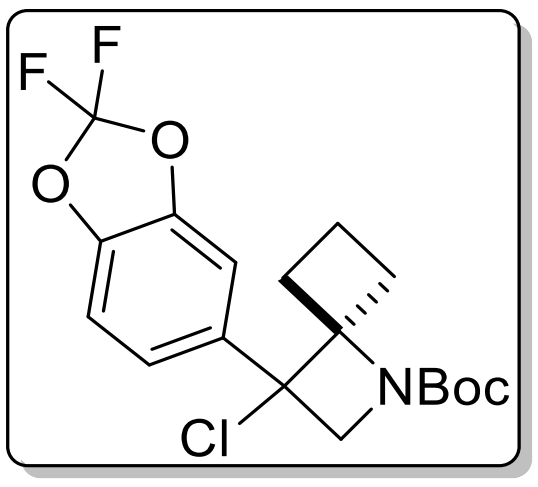


Prepared following **General Procedure 6** from 3-(2,2-difluorobenzo[_d_][1,3]dioxol-5-yl)-2_H_-azirine **Y** (35 mg, 0.18 mmol) and (cyclobutyl(phenyl)(2,4,6- trimethoxyphenyl)sulfonium tetrafluoroborate **3** (97 mg, 0.23 mmol). Work-up affords the crude material with a quantitative $^1$H-NMR yield of 75% over two steps. Purification by flash column chromatography (5-10% $Et_2O$/hexane) affords the title compound **71** (23 mg, 0.059 mmol, 33%) as a colourless oil.

_Note**:** The $^1$H-NMR spectrum of the crude material of the 1-azabicyclo[1.1.0]butane intermediate, with the key product signals integrated, is included in the section: **13. NMR spectra**_

**$^1$H-NMR** (400 MHz, $CDCl_3$): $\delta_H$ 7.26 (1H, d, _J_ = 2.0 Hz), 7.20 (1H, dd, _J_ = 8.4, 2.0 Hz), 7.08 (1H, d, _J_ = 8.4 Hz), 4.50 (1H, d, _J_ = 9.6 Hz), 4.19 (1H, d, _J_ = 9.7 Hz), 2.75 (2H, m), 2.51 – 2.27 (1H, m), 1.89 (1H, m), 1.63 (1H, m), 1.52 (10H, m).

_Note: Signal of the Boc-group is split due to the presence of rotamers._

**$^{19}$F-NMR** (377 MHz, $CDCl_3$): $\delta_F$ -49.82 (s).

**$^{13}$C{$^1$H}-NMR** (101 MHz, DMSO-$d_6$): $\delta_C$ 153.9, 142.9, 142.4, 135.0, 131.1 (t, _J_ = 256.4 Hz), 123.2, 109.9, 108.9, 79.5, 77.7, 71.8, 59.1, 30.1, 27.9, 11.6.

_Note: The signal for the carbonyl group was observed upon recording the $^{13}$C-spectrum at 50 °C with increased relaxation delay of 6 seconds._

**IR** (neat, _v_ cm$^{-1}$): 2976, 1697, 1236, 1149, 1034.

**HRMS** (ESI+): _m/z_ calcd for $C_{18}H_{20}{}^{35}ClF_2NO_4Na$ $[M+Na]^+$ 410.0941, found 410.0928

***tert*-Butyl 3-chloro-3-(2,2-difluorobenzo[*d*][1,3]dioxol-5-yl)-6-oxa-1-azaspiro[3.3]heptane-1-carboxylate, 72**

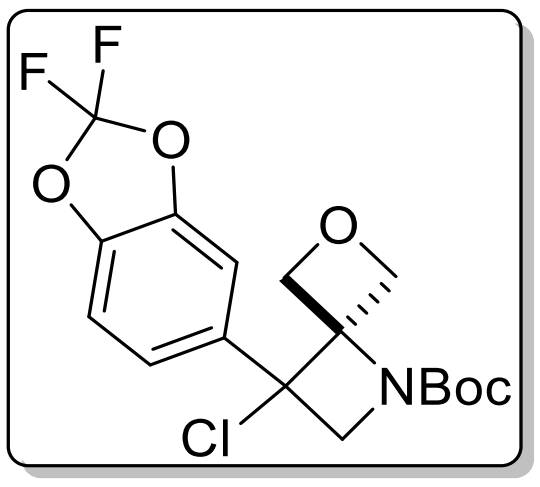


Prepared following **General Procedure 6** from 3-(2,2-difluorobenzo[*d*][1,3]dioxol-5-yl)-2*H*-azirine **Y** (37 mg, 0.178 mmol) and (4-chlorophenyl)(oxetan-3-yl)(2,4,6-trimethoxyphenyl)sulfonium tetrafluoroborate **2** (105 mg, 0.23 mmol). Work-up affords the crude material with a quantitative $^{1}$H-NMR yield of 81% over two steps. Purification by flash column chromatography (18% EtOAc/hexane) affords the title compound **72** (23 mg, 0.059 mmol, 33%) as a colourless oil.

**$^{1}$H-NMR** (400 MHz, $CDCl_3$): $\delta_H$ 7.28 (1H, d, *J* = 1.9 Hz), 7.21 (1H, dd, *J* = 8.4, 2.0 Hz), 7.12 (1H, d, *J* = 8.4 Hz), 5.17 (2H, d, *J* = 5.7 Hz), 4.83 (1H, s), 4.53 (1H, d, *J* = 9.4 Hz), 4.23 (1H, d, *J* = 9.5 Hz), 4.09 (1H, d, *J* = 7.6 Hz), 1.54 (9H, s).

**$^{19}$F-NMR** (377 MHz, $CDCl_3$): $\delta_F$ -49.77 (s)

**$^{13}$C{$^{1}$H}-NMR** (101 MHz, $CDCl_3$): $\delta_C$ 144.4, 144.0, 134.3, 131.9 (t, *J* = 254.4 Hz), 122.0, 109.8, 108.4, 81.8, 78.2, 77.4, 68.6, 61.0, 28.6.

*Note: The signals for the carbonyl group and some quaternary carbons were not observed in the $^{13}$C{$^{1}$H}-NMR spectrum.*

**IR** (neat, *v* cm$^{-1}$): 2976, 1702, 1391, 1238, 1149.

**HRMS** (ESI+): *m/z* calcd for $C_{17}H_{18}{}^{35}ClF_2NO_5Na$ $[M+Na]^+$ 412.0734, found 412.0748

**12-Chloro-12-phenyl-2-oxa-5-azadispiro[3.1.5$^{6}$.1$^{4}$]dodecane, 73**

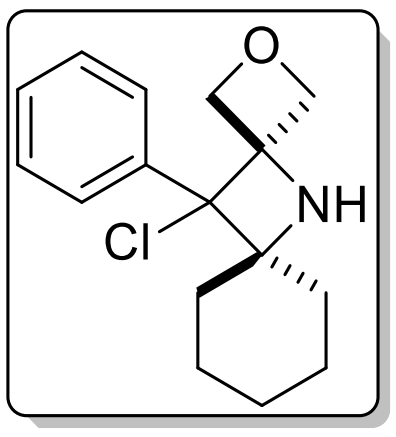


Prepared following **General Procedure 6** from 2-phenyl-1-azaspiro[2.5]oct-1-ene **AE** (30 mg, 0.16 mmol) and (4-chlorophenyl)(oxetan-3-yl)(2,4,6-trimethoxyphenyl)sulfonium tetrafluoroborate **2** (96 mg, 0.21 mmol). Work-up affords the crude material with a quantitative $^{1}$H-NMR yield of 66% over two steps. Purification by flash column chromatography (5-60% EtOAc/hexane) affords the title compound **73** (25 mg, 0.090 mmol, 56%) as a yellow oil.

*Note**:** The $^{1}$H-NMR spectrum of the crude material of the 1-azabicyclo[1.1.0]butane intermediate, with the key product signals integrated, is included in the section: **13. NMR spectra***

**$^{1}$H-NMR** (400 MHz, CDCl$_3$): $\delta_H$ 7.54 – 7.47 (2H, m), 7.43 – 7.37 (2H, m), 7.36 – 7.28 (1H, m), 5.42 (1H, dd, *J* = 7.9, 1.2 Hz), 4.93 (1H, dd, *J* = 7.8, 1.1 Hz), 4.74 (1H, dd, *J* = 7.9, 1.1 Hz), 4.68 (1H, dd, *J* = 7.8, 1.1 Hz), 2.30 (1H, m), 2.08 – 1.98 (1H, m), 1.82 – 1.75 (1H, m), 1.69 (1H, m), 1.54 (2H, m), 1.44 – 1.35 (1H, m), 1.30 – 1.21 (2H, m), 1.16 (1H, m), 1.05 – 0.90 (1H, m).

**$^{13}$C{$^{1}$H}-NMR** (101 MHz, CDCl$_3$): $\delta_C$ 138.2, 128.5, 128.2, 127.6, 83.7, 82.3, 79.8, 70.5, 68.5, 36.6, 35.0, 25.4, 22.5, 22.4.

**IR** (neat, *v* cm$^{-1}$): 2932, 2855, 1445, 1351, 980.

**HRMS** (ESI+): *m/z* calcd for $C_{16}H_{21}{}^{35}ClNO$ [M+H]$^+$ 278.1306, found 278.1299

**(3-Bromophenyl)(3-chloro-3-(4-fluorophenyl)-6-tosyl-1,6-diazaspiro[3.3]heptan-1-yl)methanone, 74**

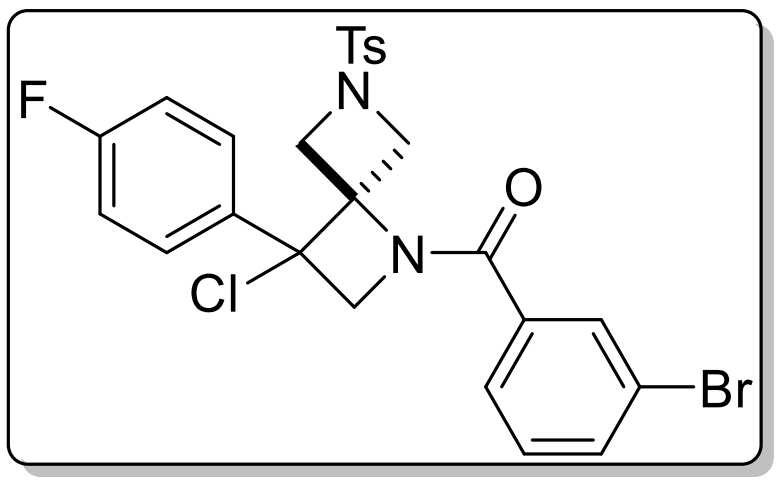


To a solution of 3-(4-fluorophenyl)-2*H*-azirine **J** (38 mg, 0.28 mmol, 1.0 equiv.) and (4-chlorophenyl)(1-tosylazetidin-3-yl)(2,4,6- trimethoxyphenyl)sulfonium tetrafluoroborate **1** (222 mg, 0.37 mmol, 1.3 equiv.) in THF (3 mL) at 0 °C was added dropwise a solution of LiHMDS (0.45 mL, 1.0 M in THF, 1.6 equiv.). The mixture was stirred at 0 °C for 10 min, then allowed to warm to room temperature and stirred for 2 h. The reaction was quenched by the addition of methanol (0.2 mL) and concentrated under reduced pressure. The resulting crude material was dissolved in acetonitrile (3 mL). 3-Bromobenzoyl chloride (123 mg, 0.56 mmol, 2.0 equiv.) and Amberlyst (45 mg) were added, and the mixture was stirred at room temperature for 12 h. The reaction mixture was then diluted with EtOAc, filtered, and concentrated under reduced pressure to afford the crude material with a quantitative $^{1}$H-NMR yield of 75% over two steps. Purification by flash column chromatography (20-40% EtOAc/hexane) affords the title compound **74** (68 mg, 0.12 mmol, 43%) as a white solid.

**$^{1}$H-NMR** (400 MHz, $CDCl_3$): $\delta_H$ 7.77 – 7.65 (3H, m), 7.66 – 7.56 (1H, m), 7.52 – 7.43 (1H, m), 7.38 – 7.27 (5H, m), 7.17 – 7.02 (2H, m), 4.87 (1H, d, *J* = 9.8 Hz), 4.58 (2H, d, *J* = 8.7 Hz), 4.53 (1H, d, *J* = 9.8 Hz), 4.27 (1H, s), 3.40 (1H, d, *J* = 9.1 Hz), 2.48 (3H, s).

**$^{19}$F-NMR** (377 MHz, $CDCl_3$): $\delta_F$ -111.11 (s).

**$^{13}$C{$^{1}$H}-NMR** (101 MHz, $CDCl_3$): $\delta_C$ 163.0 (d, *J* =250.7 Hz), 144.4, 135.2, 134.7, 132.6 (d, *J* = 3.5 Hz), 131.5, 130.8, 130.4, 130.0, 128.6, 128.5 (d, *J* = 8.5 Hz), 126.1, 123.0, 116.4 (d, *J* = 22.0 Hz), 72.8, 69.3, 65.0, 57.4, 55.9, 21.8.

*Note: The signal for the carbonyl group was not observed in the $^{13}$C{$^{1}$H}-NMR spectrum.*

**IR** (neat, *v* $cm^{-1}$): 2927, 1640, 1508, 1428, 1157.

**HRMS** (ESI+): *m/z* calcd for $C_{25}H_{21}{}^{79}Br^{35}ClFN_2O_3SNa$ $[M+Na]^+$ 585.0021, found 584.9882.

**(4-Bromophenyl)(3-chloro-3-(4-chlorophenyl)-6-tosyl-1,6-diazaspiro[3.3]heptan-1-yl)methanone, 75**

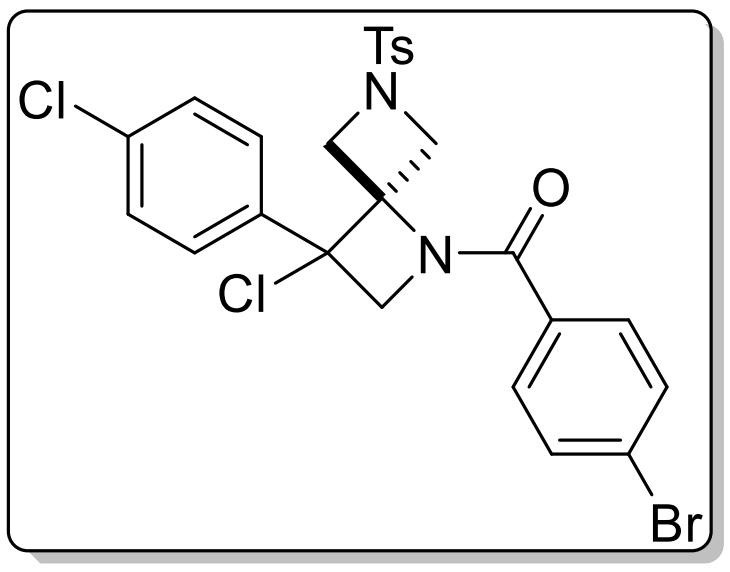


To a solution of 3-(4-chlorophenyl)-2*H*-azirine **L** (42 mg, 0.28 mmol, 1.0 equiv.) and (4-chlorophenyl)(1-tosylazetidin-3-yl)(2,4,6- trimethoxyphenyl)sulfonium tetrafluoroborate **1** (222 mg, 0.37 mmol, 1.3 equiv.) in THF (3 mL) at 0 °C was added dropwise a solution of LiHMDS (0.45 mL, 1.0 M in THF, 1.6 equiv.). The reaction mixture was stirred at 0 °C for 10 min, then allowed to warm to room temperature and stirred for 2 h. The reaction was quenched by the addition of methanol (0.2 mL) and concentrated under reduced pressure. The resulting crude material was dissolved in acetonitrile (0.03 M). 4-Bromobenzoyl chloride (123 mg, 0.56 mmol, 2.0 equiv.) and Amberlyst (45 mg) were added, and the resulting mixture was stirred at room temperature for 12 h. Upon completion, the reaction mixture was diluted with ethyl acetate, filtered, and concentrated under reduced pressure to afford the crude material with a quantitative $^1$H-NMR yield of 87% over two steps. Purification by flash column chromatography (15-30% EtOAc/hexane) affords the title compound **75** (80 mg, 0.14 mmol, 50%) as a white solid.

**$^1$H-NMR** (400 MHz, $CDCl_3$): $\delta_H$ 7.72 – 7.67 (2H, m), 7.58 – 7.50 (2H, m), 7.45 – 7.33 (6H, m), 7.29 – 7.18 (2H, m), 4.85 (1H, d, *J* = 9.7 Hz), 4.56 (2H, d, *J* = 8.8 Hz), 4.52 (1H, d, *J* = 9.7 Hz), 4.28 (1H, s), 3.38 (1H, d, *J* = 9.1 Hz), 2.48 (3H, s).

**$^{13}$C{$^1$H}-NMR** (101 MHz, $CDCl_3$): $\delta_C$ 144.4, 135.6, 135.1, 132.1, 131.7, 130.0, 129.5, 129.3, 128.6, 127.8, 126.5, 72.8, 69.2, 65.0, 57.4, 55.9, 21.8.

*Note: The signal for the carbonyl group was not observed in the $^{13}$C{$^1$H}-NMR spectrum.*

**IR** (neat, *v* cm$^{-1}$): 2930, 1641, 1405, 1160, 1090.

**HRMS** (ESI+): *m/z* calcd for $C_{25}H_{21}{}^{79}Br{}^{37}Cl_2N_2O_3SNa$ $[M+Na]^+$ 604.9681, found 604.9702

**(3-(4-Bromophenyl)-3-chloro-6-oxa-1-azaspiro[3.3]heptan-1-yl)(2-fluorophenyl)methanone, 76**

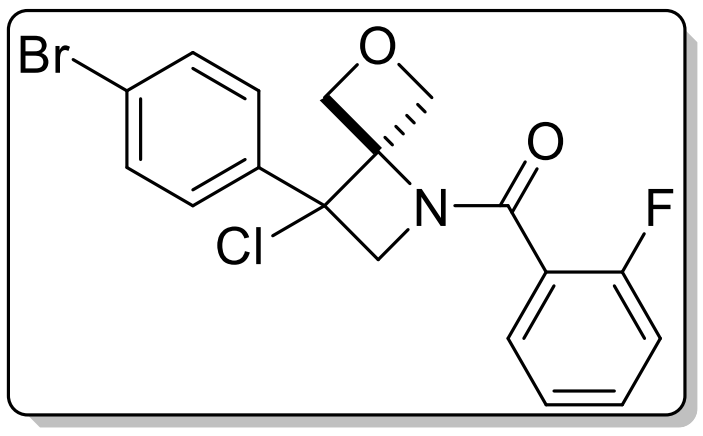


To a solution of 3-(4-bromophenyl)-2*H*-azirine **N** (55 mg, 0.28 mmol, 1.0 equiv.) and (4-chlorophenyl)(oxetan-3-yl)(2,4,6-trimethoxyphenyl)sulfonium tetrafluoroborate **2** (166 mg, 0.37 mmol, 1.3 equiv.) in THF (3 mL) at 0 °C was added dropwise a solution of LiHMDS (0.45 mL, 1.0 M in THF, 1.6 equiv.). The reaction mixture was stirred at 0 °C for 10 min, then allowed to warm to room temperature and stirred for 2 h. The reaction was quenched by the addition of methanol (0.2 mL) and concentrated under reduced pressure. The resulting crude material was dissolved in acetonitrile (3 mL). 2-Fluorobenzoyl chloride (89 mg, 0.56 mmol, 2.0 equiv.) and Amberlyst (45 mg) were added, and the resulting mixture was stirred at room temperature for 12 h. Upon completion, the reaction mixture was diluted with ethyl acetate, filtered, and concentrated under reduced pressure to afford the crude material with a quantitative $^{1}$H-NMR yield of 60% over two steps. Purification by flash column chromatography (10-50% EtOAc/hexane) affords the title compound **76** (48 mg, 0.12 mmol, 42%) as a yellow oil.

**$^{1}$H-NMR** (400 MHz, $CDCl_3$): $\delta_H$ 7.72 – 7.39 (4H, m), 7.39 – 7.23 (3H, m), 7.22 – 7.08 (1H, m), 5.08 (1H, dd, *J* = 10.2, 2.1 Hz), 4.67 (1H, dd, *J* = 12.6, 2.1 Hz), 4.44 – 4.15 (3H, m), 3.84 (1H, d, *J* = 12.5 Hz).

**$^{19}$F-NMR** (377 MHz, $CDCl_3$): $\delta_F$ -113.08 (m).

**$^{13}$C{$^{1}$H}-NMR** (101 MHz, $CDCl_3$): $\delta_C$ 168.0, 158.7 (d, *J* = 250.6 Hz), 136.5, 133.3 (d, *J* = 8.3 Hz), 132.0, 130.2 (d, *J* = 3.1 Hz), 128.9, 125.1 (d, *J* = 3.6 Hz), 123.5, 121.4 (d, *J* = 16.4 Hz), 116.4 (d, *J* = 21.7 Hz), 80.3, 69.5, 65.4, 63.1, 43.4.

**IR** (neat, *v* cm$^{-1}$): 3397, 1613, 1456, 1420, 1010.

**HRMS:** *exact mass not found in ESI+ or ESI- mode*

**(3-Chloro-3-(4-chlorophenyl)-6-oxa-1-azaspiro[3.3]heptan-1-yl)(4-(trifluoromethyl)phenyl)methanone, 77**

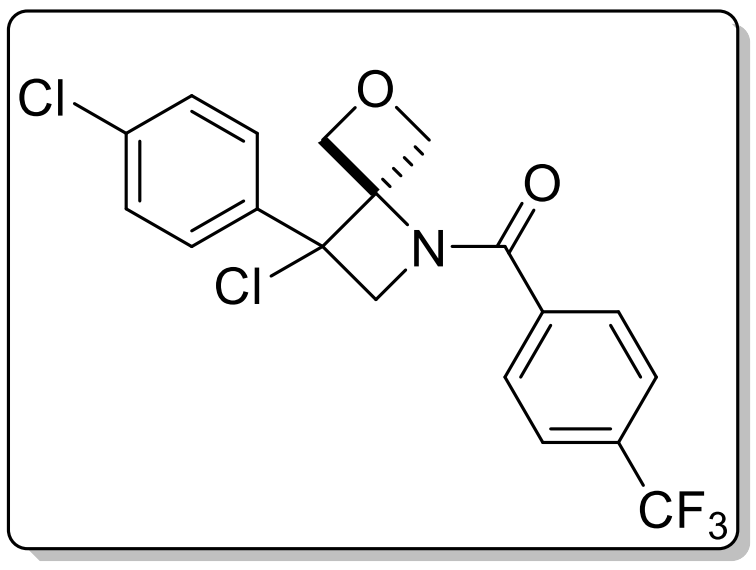


To a solution of 3-(4-chlorophenyl)-2*H*-azirine **L** (55 mg, 0.28 mmol, 1.0 equiv.) and (4-chlorophenyl)(oxetan-3-yl)(2,4,6-trimethoxyphenyl)sulfonium tetrafluoroborate **2** (166 mg, 0.37 mmol, 1.3 equiv.) in THF (3 mL) at 0 °C was added dropwise a solution of LiHMDS (0.45 mL, 1.0 M in THF, 1.6 equiv.). The reaction mixture was stirred at 0 °C for 10 min, then allowed to warm to room temperature and stirred for 2 h. The reaction was quenched by the addition of methanol (0.2 mL) and concentrated under reduced pressure. The resulting crude material was dissolved in acetonitrile (3 mL). 4-(Trifluoromethyl)benzoyl chloride (117 mg, 0.56 mmol, 2.0 equiv.) and Amberlyst (45 mg) were added, and the resulting mixture was stirred at room temperature for 12 h. Upon completion, the reaction mixture was diluted with ethyl acetate, filtered, and concentrated under reduced pressure to afford the crude material with a quantitative $^1$H-NMR yield of 60% over two steps. Purification by flash column chromatography (10-25% EtOAc/hexane) affords the title compound **77** (40 mg, 0.096 mmol, 35%) as a colourless oil.

**$^1$H-NMR** (400 MHz, $CDCl_3$): $\delta_H$ 7.75 (4H, s), 7.40 (2H, d, *J* = 8.7 Hz), 7.32 – 7.26 (2H, m), 5.25 (1H, d, *J* = 9.9 Hz), 4.69 (1H, d, *J* = 12.5 Hz), 4.47 (2H, d, *J* = 9.9 Hz), 4.27 (1H, d, *J* = 12.6 Hz), 3.77 (1H, d, *J* = 12.6 Hz).

**$^{19}$F-NMR** (377 MHz, $CDCl_3$): $\delta_F$ -63.07 (s).

**$^{13}$C{$^1$H}-NMR** (101 MHz, $CDCl_3$): $\delta_C$ 170.1, 135.9, 135.3, 133.5 (q, *J* = 33.0 Hz), 130.2, 129.2, 128.3, 128.1, 126.0 (q, *J* = 3.8 Hz), 123.6 (q, *J* = 272.4 Hz), 80.5, 70.0, 65.6, 64.8, 44.2.

**IR** (neat, *v* cm$^{-1}$): 3370, 1630, 1418, 1322, 1128.

**HRMS** (ESI+): *m/z* calcd for $C_{19}H_{14}{}^{35}Cl_2F_3NO_2Na$ $[M+Na]^+$ 438.0246, found 438.0237

**3-Chloro-1-tosyl-3-(4-(trifluoromethyl)phenyl)-1-azaspiro[3.3]heptane, 78**

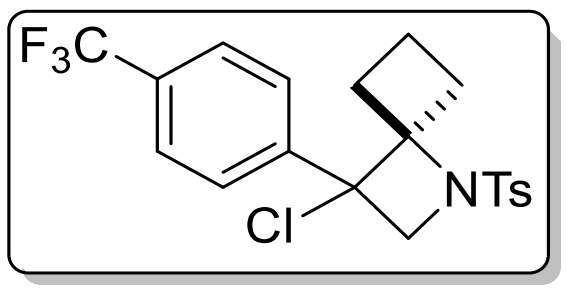

To a solution of 3-(4-(trifluoromethyl)phenyl)-2*H*-azirine **K** (52 mg, 0.28 mmol, 1.0 equiv.) and (cyclobutyl(phenyl)(2,4,6- trimethoxyphenyl)sulfonium tetrafluoroborate **3** (222 mg, 0.37 mmol, 1.3 equiv.) in THF (3 mL) at 0 °C was added dropwise a solution of LiHMDS (0.45 mL, 1.0 M in THF, 1.6 equiv.). The reaction mixture was stirred at 0 °C for 10 min, then allowed to warm to room temperature and stirred for 2 h. The reaction was quenched by the addition of methanol (0.2 mL) and concentrated under reduced pressure. The resulting crude material was dissolved in acetonitrile (3 mL). 4-Toluenesulfonyl chloride (107 mg, 0.56 mmol, 2.0 equiv.) and Amberlyst (45 mg) were added, and the resulting mixture was stirred at room temperature for 12 h. Upon completion, the reaction mixture was diluted with ethyl acetate, filtered, and concentrated under reduced pressure to afford the crude material with a quantitative $^{1}$H-NMR yield of 55% over two steps. Purification by flash column chromatography (8% EtOAc/hexane) affords the title compound **78** (33 mg, 0.077 mmol, 27%) as a colourless oil.

**$^{1}$H-NMR** (400 MHz, $CDCl_3$): $\delta_H$ 7.82 (2H, d, *J* = 8.3 Hz), 7.63 (4H, q, *J* = 8.6 Hz), 7.36 (2H, d, *J* = 8.2 Hz), 4.51 (1H, d, *J* = 8.4 Hz), 4.08 (1H, d, *J* = 8.4 Hz), 2.84 – 2.77 (2H, m), 2.64 – 2.52 (1H, m), 2.46 (3H, s), 1.90 – 1.80 (1H, m), 1.61 – 1.53 (1H, m), 1.41 (1H, m).

**$^{19}$F-NMR** (377 MHz, $CDCl_3$): $\delta_F$ -62.77 (s).

**$^{13}$C{$^{1}$H}-NMR** (101 MHz, $CDCl_3$): $\delta_C$ 144.4, 142.4, 136.0, 130.9 (q, *J* = 32.8 Hz), 130.0, 127.9, 127.1, 125.7 (q, *J* = 3.8 Hz) 123.9 (q, *J* = 272.3 Hz), 81.9, 70.0, 61.8, 31.7, 31.6, 21.8, 13.0.

**IR** (neat, *v* $cm^{-1}$): 2953, 1322, 1156, 1120, 1069.

**HRMS** (ESI+)**:** *m/z* calcd for $C_{20}H_{19}{}^{35}ClF_3NO_2SNa$ $[M+Na]^+$ 452.0669, found 452.0657

**3-Chloro-3-(4-fluorophenyl)-1-((4-(5-(*p*-tolyl)-3-(trifluoromethyl)-1*H*-pyrazol-1-yl)phenyl)sulfonyl)-1-azaspiro[3.3]heptane, 79**

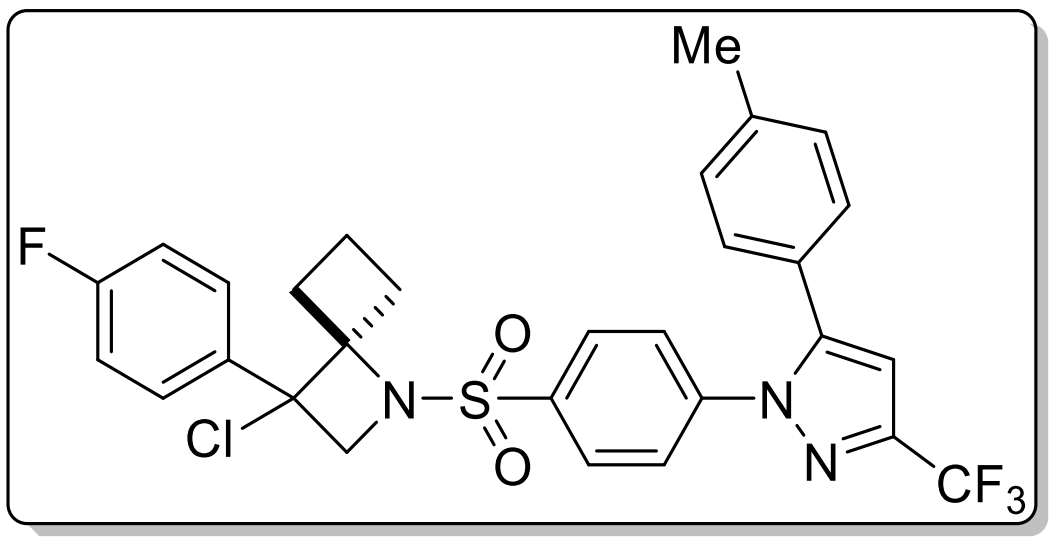


To a solution of 3-(4-fluorophenyl)-2*H*-azirine **J** (27 mg, 0.20 mmol, 1.0 equiv.) and (cyclobutyl(phenyl)(2,4,6-trimethoxyphenyl)sulfonium tetrafluoroborate **3** (109 mg, 0.26 mmol, 1.3 equiv.) in THF (3 mL) at 0 °C was added dropwise a solution of LiHMDS (0.32 mL, 1.0 M in THF, 1.6 equiv.). The reaction mixture was stirred at 0 °C for 10 min, then allowed to warm to room temperature and stirred for 2 h. The reaction was quenched by the addition of methanol (0.2 mL) and concentrated under reduced pressure. The resulting crude material was dissolved in acetonitrile (3 mL). 4-(5-(*p*-Tolyl)-3-(trifluoromethyl)-1*H*-pyrazol-1-yl)benzenesulfonyl chloride (160 mg, 0.40 mmol, 2.0 equiv.) and Amberlyst (32 mg) were added, and the resulting mixture was stirred at room temperature for 12 h. Upon completion, the reaction mixture was diluted with ethyl acetate, filtered, and concentrated under reduced pressure to afford the crude material with a quantitative $^1$H-NMR yield of 61% over two steps. Purification by flash column chromatography (50% dichloromethane/hexane to 12% EtOAc/hexane) affords the title compound **79** (37 mg, 0.063 mmol, 33%) as a white solid.

**$^1$H-NMR** (400 MHz, $CDCl_3$): $\delta_H$ 7.99 – 7.85 (2H, m), 7.58 – 7.49 (2H, m), 7.48 – 7.41 (2H, m), 7.18 (2H, d, *J* = 8.1 Hz), 7.13 – 7.01 (4H, m), 6.76 (1H, s), 4.56 (1H, d, *J* = 8.5 Hz), 4.03 (1H, d, *J* = 8.5 Hz), 2.85 – 2.71 (2H, m), 2.57 – 2.46 (1H, m), 2.38 (3H, s), 1.88 – 1.80 (1H, m), 1.66 (1H,m), 1.45 – 1.32 (1H, m).

**$^{19}$F-NMR** (377 MHz, $CDCl_3$): $\delta_F$ -62.46 (3F, s), -108.05 – -117.52 (1F, m).

**$^{13}$C{$^1$H}-NMR** (101 MHz, $CDCl_3$): $\delta_C$ 162.7 (d, *J* = 249.4 Hz), 145.5, 144.4 (q, *J* = 38.6 Hz), 143.0, 140.0, 138.7, 134.1 (d, *J* = 3.3 Hz), 129.9, 128.9, 128.8, 128.5 (d, *J* = 8.3 Hz), 125.8, 125.8, 121.2 (q, *J* = 269.1 Hz), 115.8 (d, *J* = 21.7 Hz), 106.5 (br. s), 82.5, 70.6, 61.9, 31.7, 31.5, 21.5, 12.9.

**IR** (neat, *v* cm$^{-1}$): 2956, 1597, 1471, 1234, 1156.

**HRMS** (ESI-): *m/z* calcd for $C_{29}H_{23}{}^{35}ClF_4N_3O_2S$ $[M-H]^-$ 588.1141, found 588.1134

**_tert_-Butyl 3-bromo-3-(4-fluorophenyl)-1-azaspiro[3.3]heptane-1-carboxylate, 80**

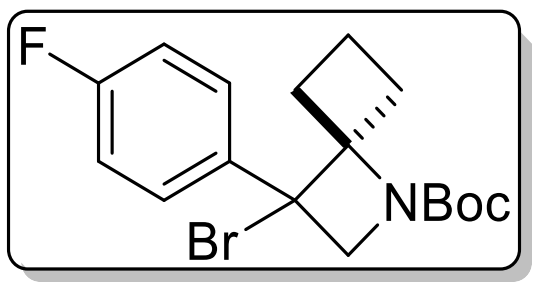


To a solution of 3-(4-fluorophenyl)-2*H*-azirine **J** (54 mg, 0.40 mmol, 1.0 equiv.) and (cyclobutyl(phenyl)(2,4,6-trimethoxyphenyl)sulfonium tetrafluoroborate **3** (217 mg, 0.52 mmol, 1.3 equiv.) in THF (3 mL) at 0 °C was added dropwise a solution of LiHMDS (0.64 mL, 1.0 M in THF, 1.6 equiv.). The reaction mixture was stirred at 0 °C for 10 min, then allowed to warm to room temperature and stirred for 2 h. The reaction was quenched by the addition of methanol (0.2 mL) and concentrated under reduced pressure. The resulting crude material was dissolved in acetonitrile (3 mL), di-*tert*-butyl dicarbonate (175 mg, 0.80 mmol, 2.0 equiv.) and lithium bromide (104 mg, 1,2 mmol, 3.0 equiv.) were added, and the resulting mixture was stirred at room temperature for 12 h. Upon completion, the reaction mixture concentrated under reduced pressure to afford the crude material with a quantitative $^{1}$H-NMR yield of 52% over two steps. Purification by flash column chromatography (5% to 10% $Et_2O$/hexane) affords the title compound **80** (35 mg, 0.095 mmol, 24%) as a white solid.

**$^{1}$H-NMR** (400 MHz, $CDCl_3$): $\delta_H$ 7.45 – 7.34 (2H, m), 7.13 – 7.03 (2H, m), 4.67 (1H, d, *J* = 9.9 Hz), 4.28 (1H, d, *J* = 9.9 Hz), 2.85 (2H, m, *J* = 22.7 Hz), 2.50 – 2.22 (1H, m), 2.05 – 1.76 (2H, m), 1.68 – 1.55 (1H, m), 1.51 (9H, s).

**$^{19}$F-NMR** (377 MHz, $CDCl_3$): $\delta_F$ -112.21 (s).

**$^{13}$C{$^{1}$H}-NMR** (101 MHz, $CDCl_3$): $\delta_C$ 162.4 (d, *J* = 249.0 Hz), 155.8, 136.2 (d, *J* = 3.5 Hz), 129.0 (d, *J* = 8.2 Hz), 115.7 (d, *J* = 21.8 Hz), 80.6, 79.1, 66.5, 59.9, 33.9, 29.7, 28.6, 12.1.

**IR** (neat, $\nu$ $cm^{-1}$): 2974, 1694, 1511, 1363, 1153.

**HRMS** (ESI+): *m/z* calcd for $C_{17}H_{21}{}^{79}BrFNO_2Na$ $[M+Na]^+$ 392.0632, found 392.0583

***tert*-Butyl 3-bromo-3-(*p*-tolyl)-6-oxa-1-azaspiro[3.3]heptane-1-carboxylate, 81**

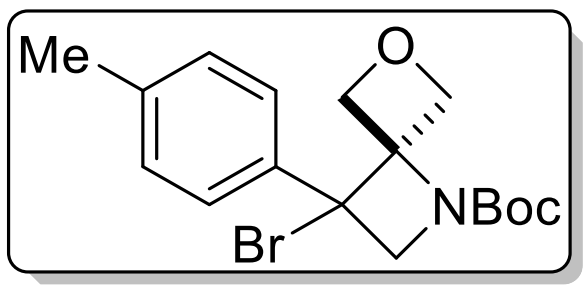


To a solution of 3-(*p*-tolyl)-2*H*-azirine **W** (74 mg, 0.56 mmol, 1.0 equiv.) and (4-chlorophenyl)(oxetan-3-yl)(2,4,6-trimethoxyphenyl)sulfonium tetrafluoroborate **2** (333 mg, 0.73 mmol, 1.3 equiv.) in THF (3 mL) at 0 °C was added dropwise a solution of LiHMDS (0.9 mL, 1.0 M in THF, 1.6 equiv.). The reaction mixture was stirred at 0 °C for 10 min, then allowed to warm to room temperature and stirred for 2 h The reaction was quenched by the addition of methanol (0.2 mL) and concentrated under reduced pressure. The resulting crude material was dissolved in acetonitrile (3 mL), di-*tert*-butyl dicarbonate (246 mg, 1.13 mmol, 2.0 equiv.) and lithium bromide (146 mg, 1.69 mmol, 3.0 equiv.) were added, and the resulting mixture was stirred at room temperature for 12 h. Upon completion, the reaction mixture concentrated under reduced pressure to afford the crude material with a quantitative $^{1}$H-NMR yield of 67% over two steps. Purification by flash column chromatography (18% EtOAc/hexane) affords the title compound **81** (104 mg, 0.30 mmol, 54%) as a yellow oil.

**$^{1}$H-NMR** (400 MHz, $CDCl_3$): $\delta_H$ 7.37 – 7.27 (2H, m), 7.23 (2H, d, *J* = 8.0 Hz), 5.22 (2H, H, d, *J* = 5.1 Hz), 4.77 (1H, s), 4.68 (1H, d, *J* = 9.7 Hz), 4.39 – 4.18 (2H, m), 2.36 (3H, s), 1.53 (9H, s).

**$^{13}$C{$^{1}$H}-NMR** (101 MHz, $CDCl_3$): $\delta_C$ 155.0, 139.1, 136.1, 129.8, 126.8, 81.2, 81.2, 77.7, 76.4, 63.7, 60.6, 28.6, 21.3.

**IR** (neat, *v* cm$^{-1}$): 2974, 2876, 1702, 1388, 1150.

**HRMS** (ESI+)**:** *m/z* calcd for $C_{17}H_{22}{}^{79}BrNO_3Na$ $[M+Na]^+$ 390.0675, found 390.0672

**_tert_-Butyl 3-bromo-3-(naphthalen-2-yl)-6-oxa-1-azaspiro[3.3]heptane-1-carboxylate, 82**

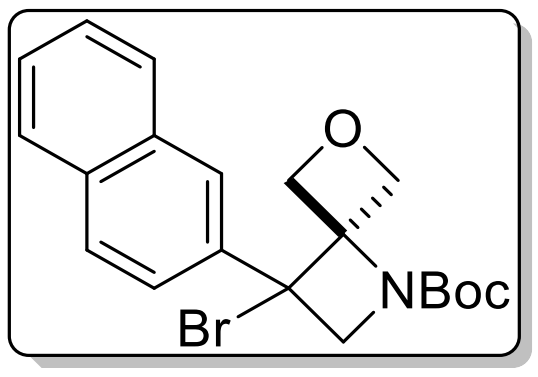

To a solution of 3-(naphthalen-2-yl)-2*H*-azirine **V** (63 mg, 0.37 mmol, 1.0 equiv.) and (4-chlorophenyl)(oxetan-3-yl)(2,4,6-trimethoxyphenyl)sulfonium tetrafluoroborate **2** (222 mg, 0.49 mmol, 1.3 equiv.) in THF (3 mL) at 0 °C was added dropwise a solution of LiHMDS (0.6 mL, 1.0 M in THF, 1.6 equiv.). The reaction mixture was stirred at 0 °C for 10 min, then allowed to warm to room temperature and stirred for 2 h. The reaction was quenched by the addition of methanol (0.2 mL) and concentrated under reduced pressure. The resulting crude material was dissolved in acetonitrile (3 mL), di-*tert*-butyl dicarbonate (164 mg, 0.75 mmol, 2.0 equiv.) and lithium bromide (98 mg, 1.13 mmol, 3.0 equiv.) were added, and the resulting mixture was stirred at room temperature for 12 h. Upon completion, the reaction mixture concentrated under reduced pressure to afford the crude material with a quantitative $^{1}$H-NMR yield of 84% over two steps. Purification by flash column chromatography (18% EtOAc/hexane) affords the title compound **82** (63 mg, 0.16 mmol, 52%) as a yellow oil.

**$^{1}$H-NMR** (400 MHz, $CDCl_3$): $\delta_H$ 7.92 (1H, d, *J* = 8.6 Hz), 7.89 – 7.83 (2H, m), 7.80 (1H, d, *J* = 2.0 Hz), 7.65 (1H, dd, *J* = 8.6, 2.0 Hz), 7.59 – 7.51 (2H, m), 5.31 (2H, d, *J* = 6.6 Hz), 4.89 (1H, d, *J* = 9.7 Hz), 4.81 – 4.75 (1H, m), 4.44 (1H, d, *J* = 9.7 Hz), 4.29 (1H, d, *J* = 7.4 Hz), 1.55 (9H, s).

**$^{13}$C{$^{1}$H}-NMR** (101 MHz, $CDCl_3$): $\delta_C$ 136.3, 133.2, 133.0, 129.3, 128.5, 127.9, 127.4, 127.1, 125.5, 124.9, 81.2, 77.6, 76.4, 63.8, 60.5, 28.6.

*Note: The signals for some quaternary carbons were not observed in the $^{13}$C{$^{1}$H}-NMR spectrum.*

**IR** (neat, *v* $cm^{-1}$): 2975, 2877, 1701, 1390, 1150.

**HRMS** (ESI+): *m/z* calcd for $C_{20}H_{22}{}^{79}BrNO_3Na$ $[M+Na]^+$ 426.0675, found 426.0674

**_tert_-Butyl 3-(4-fluorophenyl)-3-(_p_-tolylthio)-6-oxa-1-azaspiro[3.3]heptane-1-carboxylate, 83**

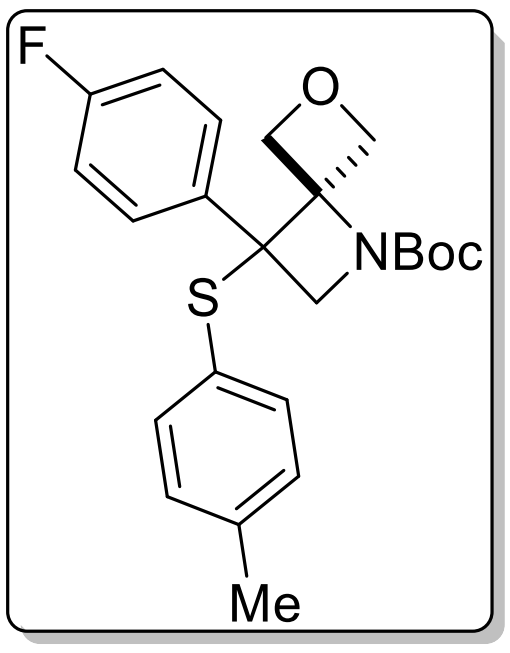


*Prepared according to a modified literature procedure.*[8]

To a solution of 3-(4-fluorophenyl)-2*H*-azirine **J** (38 mg, 0.28 mmol, 1.0 equiv.) and (4-chlorophenyl)(oxetan-3-yl)(2,4,6-trimethoxyphenyl)sulfonium tetrafluoroborate **2** (166 mg, 0.37 mmol, 1.3 equiv.) in THF (3 mL) at 0 °C was added dropwise a solution of LiHMDS (0.45 mL, 1.0 M in THF, 1.6 equiv.). The reaction mixture was stirred at 0 °C for 10 min, then allowed to warm to room temperature and stirred for 2 h. The reaction was quenched by the addition of methanol (0.2 mL) and concentrated under reduced pressure. The resulting crude material was dissolved in THF (3 mL), and 4-methylbenzenethiol (70 mg, 0.56 mmol, 2 equiv.) was added at 0°C. The resulting mixture was stirred at room temperature for 12 h. Then di-*tert*-butyl dicarbonate (123 mg, 0.56 mmol, 2.0 equiv.) and potassium carbonate (77 mg, 0.56 mmol, 2.0 equiv.) were added at 0 °C and the mixture was stirred for a further 3 hours at 25 °C. Upon completion, the reaction mixture concentrated under reduced pressure and the residue was solubilized in dichloromethane. The organic layer was washed successively with water (2 x 20 mL). The organic phase was then dried over anhydrous sodium sulfate, filtered, and concentrated under reduced pressure to afford the crude product with a quantitative $^1$H-NMR yield of 82% over two steps. Purification by flash column chromatography (18% EtOAc/hexane) affords the title compound **83** (37 mg, 0.089 mmol, 32%) as a yellow oil.

**$^1$H-NMR** (400 MHz, $CDCl_3$): $\delta_H$ 7.24 (2H, m), 7.06 – 7.00 (2H, m), 6.97 (2H, d, *J* = 8.0 Hz), 6.92 – 6.86 (2H, m), 5.27 (2H, d, *J* = 7.2 Hz), 4.90 – 4.83 (1H, m), 4.33 (1H, d, *J* = 7.4 Hz), 4.24 (1H, d, *J* = 8.6 Hz), 4.00 (1H, d, *J* = 8.6 Hz), 2.28 (3H, s), 1.52 (9H, d, *J* = 17.7 Hz).

*Note: Signal of the Boc-group is split due to the presence of rotamers.*

**$^{19}$F-NMR** (377 MHz, $CDCl_3$): $\delta_F$ -113.96 (s).

**$^{13}$C{$^1$H}-NMR** (101 MHz, $CDCl_3$): $\delta_C$ 162.1 (d, *J* = 247.8 Hz), 155.1, 139.4, 135.4, 135.1 (d, *J* = 3.1 Hz), 129.8, 129.8 (br. s), 115.5 (d, *J* = 21.6 Hz), 81.3, 78.0, 77.8, 75.0, 56.7, 56.0, 28.7, 21.3.

**IR** (neat, *v* $cm^{-1}$): 2974, 2875, 1699, 1390, 1150.

**HRMS** (ESI+): *m/z* calcd for $C_{23}H_{26}FNO_3SNa$ $[M+Na]^+$ 438.1510, found 438.1530

**_tert_-Butyl 3-(4-fluorophenyl)-3-(_p_-tolylthio)-1-azaspiro[3.3]heptane-1-carboxylate, 84**

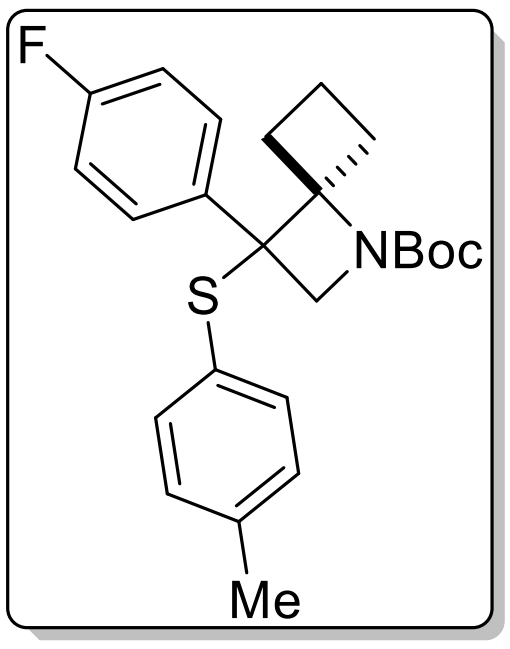


*Prepared according to a modified literature procedure.*[8]

To a solution of 3-(4-fluorophenyl)-2*H*-azirine **J** (38 mg, 0.28 mmol, 1.0 equiv.) and (cyclobutyl(phenyl)(2,4,6-trimethoxyphenyl)sulfonium tetrafluoroborate **3** (153 mg, 0.37 mmol, 1.3 equiv.) in THF (3 mL) at 0 °C was added dropwise a solution of LiHMDS (0.45 mL, 1.0 M in THF, 1.6 equiv.). The reaction mixture was stirred at 0 °C for 10 min, then allowed to warm to room temperature and stirred for 2 h. The reaction was quenched by the addition of methanol (0.2 mL) and concentrated under reduced pressure. The resulting crude material was dissolved in THF (3 mL), and 4-methylbenzenethiol (70 mg, 0.56 mmol, 2 equiv.) was added at 0°C. The resulting mixture was stirred at room temperature for 12 h. Then di-*tert*-butyl dicarbonate (123 mg, 0.56 mmol, 2.0 equiv.) and potassium carbonate (77 mg, 0.56 mmol, 2.0 equiv.) were added at 0 °C and the mixture was stirred for a further 3 hours at 25 °C. Upon completion, the reaction mixture concentrated under reduced pressure and the residue was solubilized in dichloromethane. The organic layer was washed successively with water (2 x 20 mL). The organic phase was then dried over anhydrous sodium sulfate, filtered, and concentrated under reduced pressure to afford the crude product with a quantitative $^{1}$H-NMR yield of 64% over two steps. Purification by flash column chromatography (5-7% EtOAc/hexane) affords the title compound **84** (34 mg, 0.082 mmol, 29%) as a yellow oil.

**$^{1}$H-NMR** (400 MHz, $CDCl_3$): $\delta_H$ 7.25 – 7.19 (2H, m), 7.00 (2H, app. t, *J* = 8.7 Hz), 6.94 (2H, d, *J* = 8.0 Hz), 6.88 (2H, d, *J* = 8.2 Hz), 4.29 (1H, m), 3.98 (1H, d, *J* = 8.8 Hz), 2.97 – 2.75 (2H, m), 2.32 (1H, m), 2.25 (3H, s), 1.97 – 1.77 (2H, m), 1.66 (1H, m), 1.57 – 1.42 (9H, m).

$^{19}$**F-NMR** (377 MHz, $CDCl_3$): $\delta_F$ -115.04 (s).

$^{13}$**C{$^1$H}-NMR** (101 MHz, $CDCl_3$): $\delta_C$ 161.8 (d, *J* = 247.0 Hz), 156.0, 136.5, 134.9, 134.2, 129.9 (d, *J* = 8.9 Hz), 129.6, 128.0, 115.2 (d, *J* = 21.3 Hz), 80.3, 76.3, 58.1, 55.4, 30.6, 30.0, 28.7, 21.2, 13.4.

**IR** (neat, *v* $cm^{-1}$): 2973, 1696, 1389, 1365, 1155.

**HRMS** (ESI+): *m/z* calcd for $C_{24}H_{28}FNO_2SNa$ $[M+Na]^+$ 436.1717, found 436.1716

**_tert_-Butyl 3-(4-bromophenyl)-3-(_p_-tolylthio)-1-azaspiro[3.3]heptane-1-carboxylate, 85**

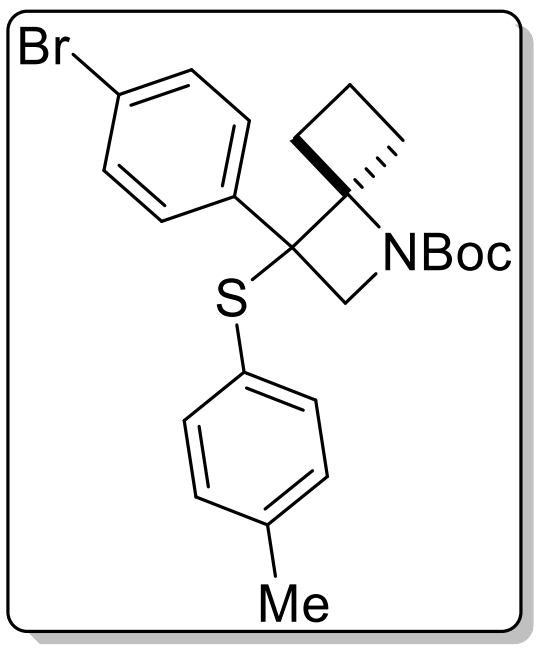


*Prepared according to a modified literature procedure.*[8]

To a solution of 3-(4-bromophenyl)-2*H*-azirine **N** (55mg, 0.28 mmol, 1.0 equiv.) and (cyclobutyl(phenyl)(2,4,6-trimethoxyphenyl)sulfonium tetrafluoroborate **3** (153 mg, 0.37 mmol, 1.3 equiv.) in THF (3 mL) at 0 °C was added dropwise a solution of LiHMDS (0.45 mL, 1.0 M in THF, 1.6 equiv.). The reaction mixture was stirred at 0 °C for 10 min, then allowed to warm to room temperature and stirred for 2 h. The reaction was quenched by the addition of methanol (0.2 mL) and concentrated under reduced pressure. The resulting crude material was dissolved in THF (3 mL), and 4-methylbenzenethiol (70 mg, 0.56 mmol, 2 equiv.) was added at 0°C. The resulting mixture was stirred at room temperature for 12 h. Then di-*tert*-butyl dicarbonate (123 mg, 0.56 mmol, 2.0 equiv.) and potassium carbonate (77 mg, 0.56 mmol, 2.0 equiv.) were added at 0 °C and the mixture was stirred for a further 3 hours at 25 °C. Upon completion, the reaction mixture concentrated under reduced pressure and the residue was solubilized in dichloromethane. The organic layer was washed successively with water (2 x 20 mL). The organic phase was then dried over anhydrous sodium sulfate, filtered, and concentrated under reduced pressure to afford the crude product with a quantitative $^{1}$H-NMR yield of 36% over two steps. Purification by flash column chromatography (5% EtOAc/hexane) affords the title compound **85** (38 mg, 0.080 mmol, 29%) as a yellow oil.

**$^{1}$H-NMR** (400 MHz, $CDCl_3$): $\delta_H$ 7.50 – 7.42 (2H, m), 7.15 (2H, d, *J* = 8.2 Hz), 6.95 (2H, d, *J* = 7.9 Hz), 6.89 (2H, d, *J* = 8.2 Hz), 4.36 – 4.20 (1H, m), 3.97 (1H, d, *J* = 8.8 Hz), 2.93 – 2.73 (2H, m), 2.30 (1H, m), 2.26 (3H, s), 1.97 – 1.74 (2H, m), 1.64 (1H, m), 1.50 (9H, m).

*Note: Signal of the Boc-group is split due to the presence of rotamers.*

**$^{13}$C{$^{1}$H}-NMR** (101 MHz, $CDCl_3$): $\delta_C$ 155.9, 138.7, 134.7, 131.4, 129.9, 129.9, 129.7, 127.9, 121.3, 80.3, 76.2, 58.1, 55.1, 30.7, 30.0, 28.7, 21.2, 13.4.

*Note: Some signals are doubled due to the presence of rotamers.*

**IR** (neat, *v* $cm^{-1}$):2972, 1695, 1390, 1385, 1153.

**HRMS** (ESI+): *m/z* calcd for $C_{24}H_{28}{}^{81}BrNO_2SNa$ $[M+Na]^+$ 498.0898, found 498.0903

**_tert_-Butyl 3-(3-chlorophenyl)-3-(_p_-tolylthio)-1-azaspiro[3.3]heptane-1-carboxylate, 86**

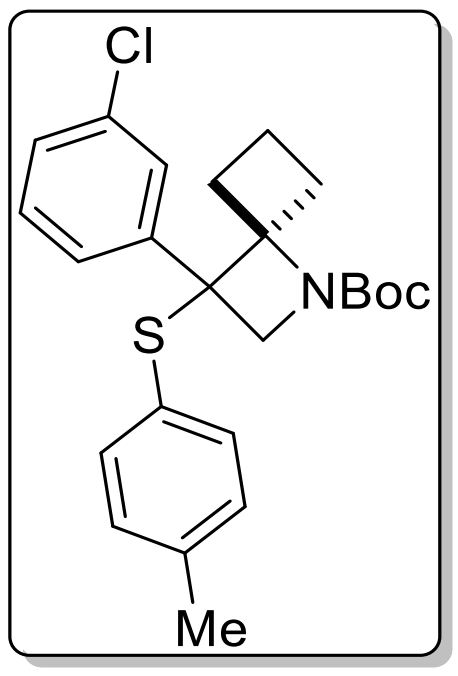


*Prepared according to a modified literature procedure.[8]*

To a solution of 3-(3-chlorophenyl)-2*H*-azirine **M** (42 mg, 0.28 mmol, 1.0 equiv.) and (cyclobutyl(phenyl)(2,4,6-trimethoxyphenyl)sulfonium tetrafluoroborate **3** (153 mg, 0.37 mmol, 1.3 equiv.) in THF (3 mL) at 0 °C was added dropwise a solution of LiHMDS (0.45 mL, 1.0 M in THF, 1.6 equiv.). The reaction mixture was stirred at 0 °C for 10 min, then allowed to warm to room temperature and stirred for 2 h. The reaction was quenched by the addition of methanol (0.2 mL) and concentrated under reduced pressure. The resulting crude material was dissolved in THF (3 mL), and 4-methylbenzenethiol (70 mg, 0.56 mmol, 2 equiv.) was added at 0°C. The resulting mixture was stirred at room temperature for 12 h. Then di-*tert*-butyl dicarbonate (123 mg, 0.56 mmol, 2.0 equiv.) and potassium carbonate (77 mg, 0.56 mmol, 2.0 equiv.) were added at 0 °C and the mixture was stirred for a further 3 hours at 25 °C. Upon completion, the reaction mixture concentrated under reduced pressure and the residue was solubilized in dichloromethane. The organic layer was washed successively with water (2 x 20 mL). The organic phase was then dried over anhydrous sodium sulfate, filtered, and concentrated under reduced pressure to afford the crude product with a quantitative $^1$H-NMR yield of 73% over two steps. Purification by flash column chromatography (5-7% EtOAc/hexane) affords the title compound **86** (34 mg, 0.079 mmol, 29%) as a yellow oil.

**$^1$H-NMR** (400 MHz, $CDCl_3$): $\delta_H$ 7.29 – 7.19 (3H, m), 7.15 (1H, m), 6.95 (2H, d, *J* = 8.0 Hz), 6.92 – 6.87 (2H, m), 4.29 (1H, m), 3.97 (1H, d, *J* = 8.8 Hz), 2.83 (2H, d, *J* = 49.6 Hz), 2.39 – 2.30 (1H, m), 2.26 (3H, s), 2.16 – 1.79 (2H, m), 1.67 (1H, m), 1.61 – 1.42 (9H, m).

*Note: Signal of the Boc-group is split due to the presence of rotamers.*

**$^{13}C\{^1H\}$-NMR** (101 MHz, $CDCl_3$): $\delta_C$ 155.9, 142.9, 138.8, 134.8, 134.4, 129.9, 129.6, 129.6, 128.3, 127.4, 126.4, 80.3, 76.3, 58.1, 55.1, 30.7, 30.1, 28.7, 21.2, 13.4.

**IR** (neat, *v* $cm^{-1}$): 2974, 1696, 1388, 1365, 1154.

**HRMS** (ESI+): *m/z* calcd for $C_{24}H_{28}{}^{35}ClNO_2SNa$ $[M+Na]^+$ 452.1421, found 452.1440

**tert-Butyl 3-(4-fluorophenyl)-3-methoxy-1-azaspiro[3.3]heptane-1-carboxylate, 87**

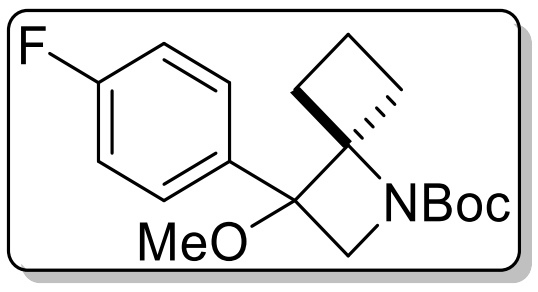


*Prepared according to a modified literature procedure.[8]*

To a solution of 3-(4-fluorophenyl)-2*H*-azirine **J** (38 mg, 0.28 mmol, 1.0 equiv.) and the (cyclobutyl(phenyl)(2,4,6-trimethoxyphenyl)sulfonium tetrafluoroborate **3** (153 mg, 0.37 mmol, 1.3 equiv.) in THF (3 mL) at 0 °C was added dropwise a solution of LiHMDS (0.45 mL, 1.0 M in THF, 1.6 equiv.). The reaction mixture was stirred at 0 °C for 10 min, then allowed to warm to room temperature and stirred for 2 h. The reaction was quenched by the addition of methanol (0.2 mL) and concentrated under reduced pressure to afford the crude material. The crude material was dissolved in methanol (3 mL) and HBr (aq. 48%, 47 mg, 0.28 mmol, 1.0 equiv.) was added at 0°C. The resulting mixture was stirred at room temperature for 12 h. Then di-*tert*-butyl dicarbonate (123 mg, 0.56 mmol, 2.0 equiv.) and potassium carbonate (77 mg, 0.56 mmol, 2.0 equiv.) were added at 0 °C and the mixture was stirred for a further 3 hours at 25 °C. Upon completion, the reaction mixture concentrated under reduced pressure and the residue was solubilized in dichloromethane. The organic layer was washed successively with water (2 x 20 mL). The organic phase was then dried over anhydrous sodium sulfate, filtered, and concentrated under reduced pressure to afford the crude product with a quantitative $^1$H-NMR yield of 64% over two steps. Purification by flash column chromatography (20% EtOAc/hexane) affords the title compound **87** (30 mg, 0.093 mmol, 33%) as a colourless oil.

**$^1$H-NMR** (400 MHz, $CDCl_3$): $\delta_H$ 7.56 – 7.31 (2H, m), 7.16 – 7.05 (2H, m), 4.14 (1H, d, *J* = 9.2 Hz), 3.93 (1H, d, *J* = 9.3 Hz), 3.13 (3H, s), 2.75 (1H, m), 2.44 – 2.35 (1H, m), 2.18 (1H, m), 1.67 (1H, m), 1.50 (9H, s), 1.35 (2H, m).

**$^{19}$F-NMR** (377 MHz, $CDCl_3$): $\delta_F$ -114.74 (s).

**$^{13}C\{^1H\}$-NMR** (101 MHz, $CDCl_3$): $\delta_C$ 162.4 (d, *J* = 246.7 Hz), 156.0, 134.1 (d, *J* = 3.2 Hz), 128.4 (d, *J* = 8.1 Hz), 115.7 (d, *J* = 21.4 Hz), 80.0, 76.3, 58.6, 52.6, 52.2, 30.1, 28.7, 27.4, 13.2.

**IR** (neat, *v* $cm^{-1}$): 2975, 1697, 1509, 1390, 1155.

**HRMS** (ESI+): *m/z* calcd for $C_{18}H_{24}FNO_3Na$ $[M+Na]^+$ 344.1632, found 344.1644

**3-(Naphthalen-2-yl)-6-tosyl-1,6-diazaspiro[3.3]heptan-3-ol, 88**

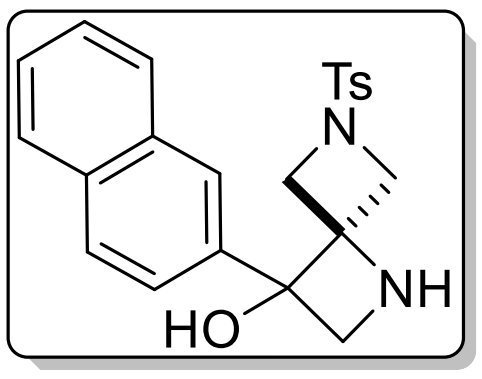


*Prepared according to a modified literature procedure.*[9]

To a solution of 3'-(naphthalen-2-yl)-1-tosyl-1'-azaspiro[azetidine-3,2'-bicyclo[1.1.0]butane] **V** (20 mg, 0.053 mmol, 1.0 equiv.) was added trifluoroacetic anhydride (10 µL, 0.64 mmol, 1.2 equiv.) at 0°C The resulting mixture was stirred at room temperature for 6 h and then concentrated under reduced pressure. The residue was dissolved in methanol (0.5 mL), and an aqueous solution of NaOH (10% w/w, 0.5 mL) was added. The mixture was stirred at room temperature for 12 h. Upon completion, the reaction was diluted with ethyl acetate. The organic layer was washed successively with saturated aqueous sodium chloride solution. The organic phase was then dried over anhydrous sodium sulfate, filtered, and concentrated under reduced pressure to afford the crude product. Purification by flash column chromatography (50% $Et_2O$/hexane to MeOH) affords the title compound **88** (14 mg, 0.035 mmol, 67%) as a colourless oil.

**$^1$H-NMR** (400 MHz, $CD_3OD$): $\delta_H$ 7.90 – 7.79 (4H, m), 7.57 – 7.51 (4H, m), 7.34 – 7.25 (3H, m), 4.42 (1H, dd, *J* = 10.2, 1.3 Hz), 4.32 (1H, d, *J* = 9.9 Hz), 3.97 (1H, dd, *J* = 10.1, 1.3 Hz), 3.77 (1H, d, *J* = 9.9 Hz), 3.69 (1H, dd, *J* = 10.1, 1.3 Hz), 3.18 (1H, dd, *J* = 10.1, 1.3 Hz), 2.46 (3H, s).

**$^{13}$C{$^1$H}-NMR** (101 MHz, $CD_3OD$): $\delta_C$ 146.1, 137.9, 134.4, 134.4, 131.4, 131.0, 130.9, 129.6, 129.5, 129.4, 128.6, 127.6, 127.6, 125.4, 77.3, 70.0, 59.5, 58.2, 57.3, 21.7.

**IR** (neat, *v* $cm^{-1}$): 3400, 2925, 1597, 1158, 1088.

**HRMS** (ESI+): *m/z* calcd for $C_{22}H_{23}N_2O_3S$ $[M+H]^+$ 395.1424, found 395.1436.

**_tert_-Butyl 3-chloro-3-(4-(phenylethynyl)phenyl)-1-azaspiro[3.3]heptane-1-carboxylate, 89**

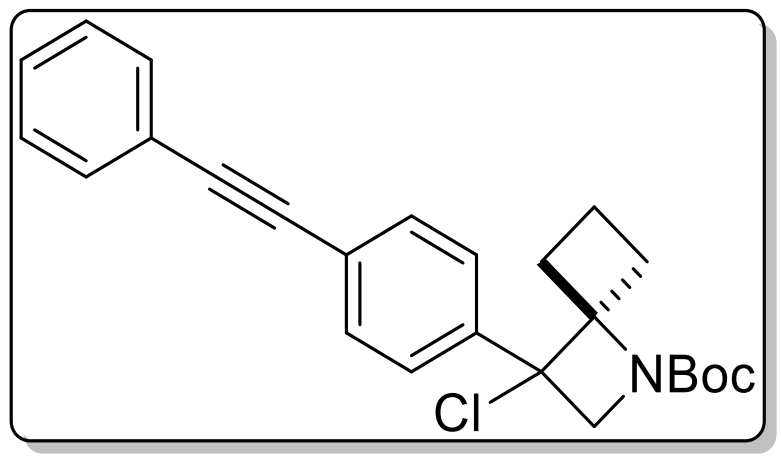


To a solution of *tert*-butyl 3-(4-bromophenyl)-3-chloro-1-azaspiro[3.3]heptane-1-carboxylate **66** (56 mg, 0.14 mmol, 1 equiv.) and phenylacetylene (16 mg, 0.15 mmol, 1.1 equiv.) in THF (1 mL) at room temperature was added Tetrakis(triphenylphosphine)-palladium(0) (8 mg, 0.007 mmol, 5 mol%), copper iodide (1.3 mg, 0.007 mmol, 5 mol%) and triethylamine (0.23 mL) consecutively. The reaction mixture was allowed to stir at 65 °C for 12 h. Upon completion of the reaction, the mixture was allowed to cool to ambient temperature, diluted with ethyl acetate, filtered through a short pad of celite, and concentrated under reduced pressure to afford crude product. Purification by flash column chromatography (7% $Et_2O$/hexane) affords the title compound **89** (26 mg, 0.064 mmol, 45%) as a yellow oil.

A sample suitable for x-ray crystallography was obtained by slow evaporation from ethyl acetate.

**$^1$H-NMR** (400 MHz, $CDCl_3$): $\delta_H$ 7.60 – 7.52 (4H, m), 7.48 (2H, d, *J* = 8.5 Hz), 7.36 (3H, dd, *J* = 5.0, 2.0 Hz), 4.59 (1H, d, *J* = 9.7 Hz), 4.19 (1H, d, *J* = 9.6 Hz), 2.85 (1H, m), 2.68 (1H, m), 2.47 (1H, m), 2.30 (1H, m), 1.89 (1H, m, *J* = 36.4 Hz), 1.70 – 1.60 (1H, m), 1.52 (9H, s).

**$^{13}$C{$^1$H}-NMR** (101 MHz, $CDCl_3$): $\delta_C$ 155.3, 139.1, 131.8, 128.6, 128.5, 126.7, 123.5, 123.1, 90.6, 88.7, 80.6, 78.9, 71.0, 60.3, 31.1, 30.5, 28.7, 12.4.

**IR** (neat, *v* cm$^{-1}$): 2974, 1697, 1387, 1365, 1154.

**HRMS** (ESI+): *m/z* calcd for $C_{25}H_{26}{}^{35}ClNO_2Na$ $[M+Na]^+$ 430.1544, found 430.1549

**Melting point:** 109.3-113.0°C

**_tert_-Butyl 3-(3-chlorophenyl)-3-tosyl-1-azaspiro[3.3]heptane-1-carboxylate, 90**

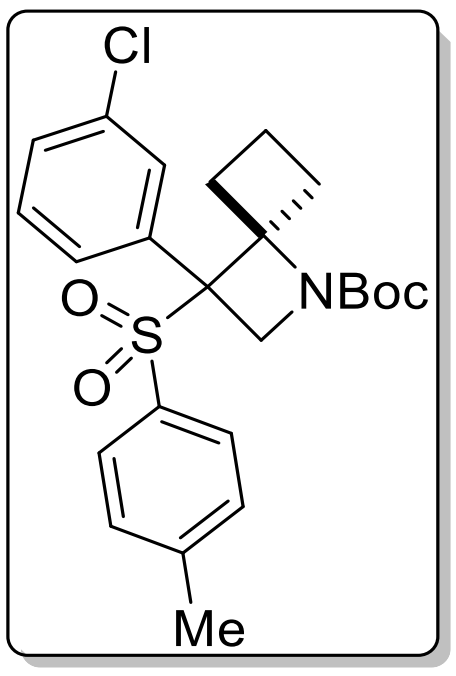


To a solution of *tert*-butyl 3-(3-chlorophenyl)-3-(*p*-tolylthio)-1-azaspiro[3.3]heptane-1-carboxylate **86** (35 mg, 0.081 mmol, 1 equiv.) in dichloromethane (3 mL) was added *meta*-chloroperoxybenzoic acid (35 mg, 0.20 mmol, 2.5 equiv.). The reaction mixture was allowed to stir at room temperature for 3 hours, before it was diluted with dichloromethane (10 mL), and quenched by the addition of saturated aqueous sodium bicarbonate solution (5 mL). The organic phase was separated, washed twice with saturated aqueous sodium bicarbonate solution (2 x 5 mL), dried over sodium sulfate, and concentrated under reduced pressure. The crude material was purified by flash column chromatography (20% EtOAc/hexane) to afford the title compound **90** (20 mg, 0.045 mmol, 56%) as a colourless oil.

A sample suitable for x-ray crystallography was obtained by slow evaporation from ethyl acetate.

**$^1$H-NMR** (400 MHz, $CDCl_3$): $\delta_H$ 7.43 – 7.32 (1H, m), 7.28 (2H, m), 7.12 (4H, m), 7.04 – 6.89 (1H, m), 4.30 (1H, m), 4.12 (1H, m), 3.48 (1H, m) 3.22 – 2.95 (1H, m), 2.36 (3H, s), 2.32 – 2.18 (2H, m), 2.02 (2H, m), 1.50 (9H, m).

*Note: Signal of the Boc-group is split due to the presence of rotamers.*

**$^{13}$C{$^1$H}-NMR** (101 MHz, $CDCl_3$): $\delta_C$ 154.5, 145.3, 134.4, 134.3, 132.7, 130.2, 129.9, 129.4, 129.3, 129.1, 128.5, 80.8, 75.6, 71.2, 50.5, 32.0, 31.8, 28.7, 21.7, 14.1.

**IR** (neat, *v* cm$^{-1}$): 2972, 1693, 1390, 1145, 1094.

**HRMS** (ESI+): *m/z* calcd for $C_{24}H_{28}{}^{35}ClNO_4SNa$ $[M+Na]^+$ 484.1320, found 484.1323

**Melting point:** 177.5-182.1°C

**_tert_-Butyl 3-(4-bromophenyl)-2-(hydroxydiphenylmethyl)-6-tosyl-1,6-diazaspiro[3.3]hept-2-ene-1-carboxylate, 91**

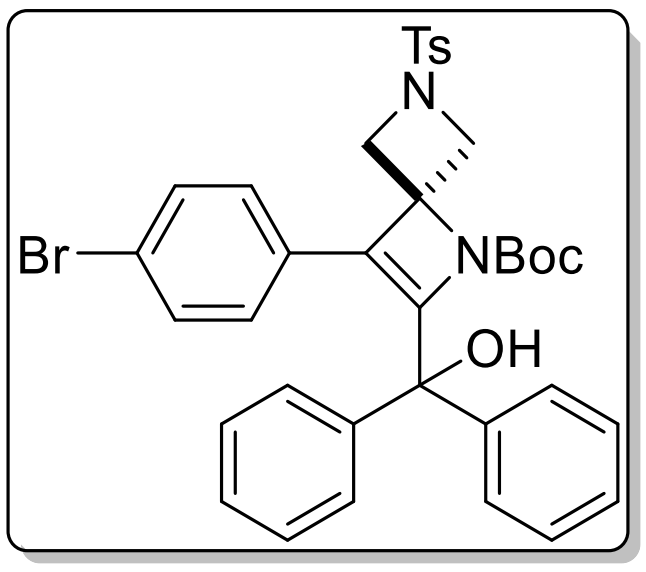


To a solution of *tert*-butyl 3-(4-bromophenyl)-3-chloro-6-tosyl-1,6-diazaspiro[3.3]heptane-1-carboxylate **56** (40 mg, 0.083 mmol, 1 equiv.) and benzophenone (18 mg, 0.099 mmol, 1.2 equiv.) in THF (3 mL) at -70 °C was added lithium diisopropylamide (0.09 mL, 0.18 mmol, 2.2 equiv.). The reaction mixture was stirred at the same temperature 2 hours, before it was diluted with ethyl acetate (20 mL), and quenched by water. The aqueous phase was extracted with ethyl acetate (3 x 10 mL), before the combined organic phase was washed with saturated aqueous sodium chloride solution (2 x 10 mL) before being dried over sodium sulfate, filtered and concentrated under reduced pressure. The resulting crude material was purified by flash column chromatography (30-40% $Et_2O$/hexane, 1% $Et_3N$) to afford the title compound **91** (19 mg, 0.027 mmol, 33%) as a colourless oil.

**$^1$H-NMR** (400 MHz, $CDCl_3$): $\delta_H$ 7.73 (2H, d, *J* = 8.3 Hz), 7.38 – 7.28 (6H, m), 7.24 (6H, m), 6.89 (2H, d, *J* = 8.6 Hz), 6.13 (2H, d, *J* = 8.6 Hz), 4.65 (2H, d, *J* = 9.0 Hz), 4.18 (2H, d, *J* = 9.0 Hz), 2.48 (3H, s), 1.45 (9H, s).

**$^{13}$C{$^1$H}-NMR** (101 MHz, $CDCl_3$): $\delta_C$ 144.5, 142.5, 131.2, 130.0, 128.5, 128.2, 128.1, 128.1, 127.7, 127.4, 121.2, 83.7, 77.4, 67.6, 56.0, 28.5, 21.8.

*Note: the signal for the carbonyl group was not observed.*

**IR** (neat, *v* $cm^{-1}$): 3304, 2977, 1672, 1412, 1162.

**HRMS** (ESI+): *m/z* calcd for $C_{36}H_{35}{}^{79}BrN_2O_5SNa$ $[M+Na]^+$ 709.1342, found 709.1328

**_tert_-Butyl 2-((4-chlorophenyl)(hydroxy)(4-((1-isopropoxy-2-methyl-1-oxopropan-2-yl)oxy)phenyl)methyl)-3-(4-fluorophenyl)-6-tosyl-1,6-diazaspiro[3.3]hept-2-ene-1-carboxylate, 92**

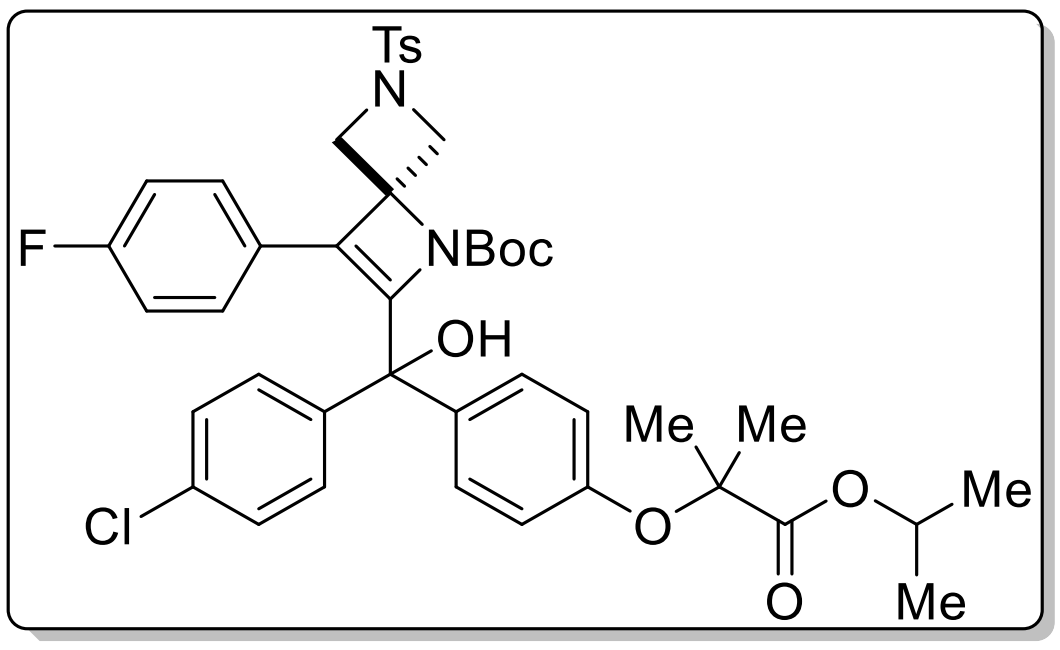


To a solution of *tert*-butyl 3-(4-fluorophenyl)-3-chloro-6-tosyl-1,6-diazaspiro[3.3]heptane-1-carboxylate **55** (39 mg, 0.081 mmol, 1 equiv.) and fenofibrate (35 mg, 0.097 mmol, 1.2 equiv.) in THF (3 mL) at -70 °C was added lithium diisopropylamide (0.09 mL, 0.18 mmol, 2.2 equiv.). The reaction mixture was stirred at the same temperature 2 hours, before it was diluted with ethyl acetate (20 mL), and quenched by water. The aqueous phase was extracted with ethyl acetate (3 x 10 mL), before the combined organic phase was washed with saturated aqueous sodium chloride solution (2 x 10 mL) before being dried over sodium sulfate, filtered and concentrated under reduced pressure. The resulting crude material was purified by flash column chromatography (10-20% EtOAc/hexane, 1% $Et_3N$) to afford the title compound **92** (40 mg, 0.049 mmol, 61%) as a white solid.

**$^1$H-NMR** (400 MHz, $CDCl_3$): $\delta_H$ 7.70 (2H, d, *J* = 8.0 Hz), 7.30 – 7.24 (4H, m), 7.19 (2H, d , *J* = 8.6 Hz), 7.09 (2H, d, *J* = 8.7 Hz), 6.65 (2H, d, *J* = 8.7 Hz), 6.55 (2H, t, *J* = 8.6 Hz), 6.33 (2H, m), 5.03 (1H, p, *J* = 6.2 Hz), 4.61 (2H, dd, *J* = 14.2, 9.0 Hz), 4.18 (1H, d, *J* = 8.7 Hz), 4.11 (1H, d, *J* = 9.1 Hz), 2.44 (3H, s), 1.52 (6H, d, *J* = 5.3 Hz), 1.45 (9H, s), 1.16 (6H, app. dd, *J* = 9.1, 6.3 Hz).

**$^{19}$F-NMR** (377 MHz, $CDCl_3$): $\delta_F$ -112.85 (s).

**$^{13}$C{$^1$H}-NMR** (101 MHz, $CDCl_3$): $\delta_C$ 173.6, 161.8 (d, *J* = 249.2 Hz), 155.6, 144.4, 141.3, 135.4, 134.0, 129.9, 128.8, 128.7, 128.5, 128.4, 128.1, 124.7 (d, *J* = 3.5 Hz), 118.5, 115.2 (d, *J* = 21.7 Hz), 83.6, 79.1, 76.5, 69.1, 67.9, 55.8, 55.7, 28.5, 25.4, 25.4, 21.7, 21.7, 21.6.

**IR** (neat, *v* cm$^{-1}$): 2981, 1669, 1416, 1155, 1090.

**HRMS** (ESI-): *m/z* calcd for $C_{43}H_{45}ClFN_2O_8S$ $[M-H]^-$ 803.2575, found 803.2603.

***tert*-Butyl 3-(4-bromophenyl)-2-((4-chlorophenyl)(hydroxy)(4-((1-isopropoxy-2-methyl-1-oxopropan-2-yl)oxy)phenyl)methyl)-6-oxa-1-azaspiro[3.3]hept-2-ene-1-carboxylate, 93**

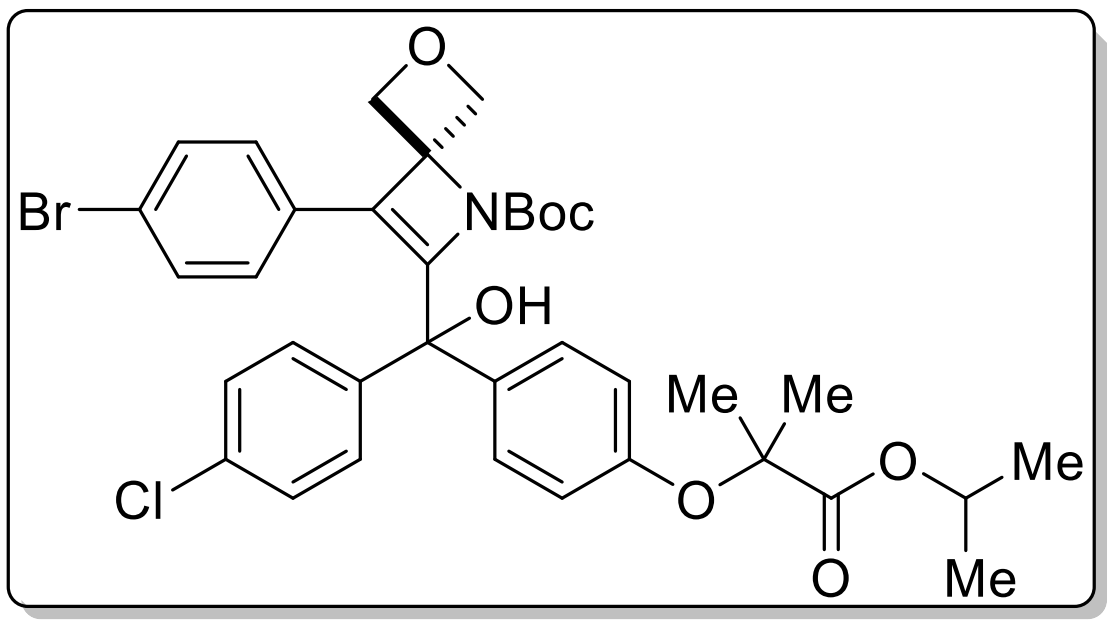


To a solution of *tert*-butyl 3-(4-bromophenyl)-3-chloro-6-oxa-1-azaspiro[3.3]heptane-1-carboxylate **61** (32 mg, 0.082 mmol, 1 equiv.) and fenofibrate (36 mg, 0.098 mmol, 1.2 equiv.) in THF (3 mL) at -70 °C was added lithium diisopropylamide (0.1 mL, 0.19 mmol, 2.2 equiv.). The reaction mixture was stirred at the same temperature 2 hours, before it was diluted with ethyl acetate (20 mL), and quenched by water. The aqueous phase was extracted with ethyl acetate (3 x 10 mL), before the combined organic phase was washed with saturated aqueous sodium chloride solution (2 x 10 mL) before being dried over sodium sulfate, filtered and concentrated under reduced pressure. The resulting crude material was purified by flash column chromatography (10-15% EtOAc/hexane, 1% $Et_3N$) to afford the title compound **93** (28 mg, 0.040 mmol, 49%) as a colourless oil.

**$^1$H-NMR** (400 MHz, $CDCl_3$): $\delta_H$ 7.38 – 7.32 (2H, m), 7.31 – 7.24 (2H, m), 7.20 – 7.13 (4H, m), 6.73 – 6.67 (2H, m), 6.57 – 6.48 (2H, m), 5.39 (2H, dd, *J* = 15.8, 7.3 Hz), 5.02 (2H, d, *J* = 7.5 Hz), 4.95 (1H, d, *J* = 7.3 Hz), 1.56 (6H, d, *J* = 7.2 Hz), 1.51 (9H, s), 1.18 (6H, dd, *J* = 12.1, 6.2 Hz).

**$^{13}$C{$^1$H}-NMR** (101 MHz, $CDCl_3$): $\delta_C$ 173.7, 155.8, 150.5, 141.4, 135.6, 134.2, 131.7, 128.8, 128.7, 128.7, 128.1, 128.0, 121.7, 118.7, 83.2, 79.2, 76.9, 76.0, 75.8, 73.3, 69.2, 28.6, 25.4, 25.4, 21.7, 21.7.

**IR** (neat, *v* cm$^{-1}$): 2978, 1669, 1411, 1153, 1099.

**HRMS** (ESI+): *m/z* calcd for $C_{36}H_{39}{}^{79}Br^{35}ClNO_7Na$ $[M+Na]^+$ 734.1491, found 734.1500.

## 7. Continuous-flow synthesis of 3'-(4-fluorophenyl)-1-tosyl-1'-azaspiro[azetidine-3,2'-bicyclo[1.1.0]butane], 4

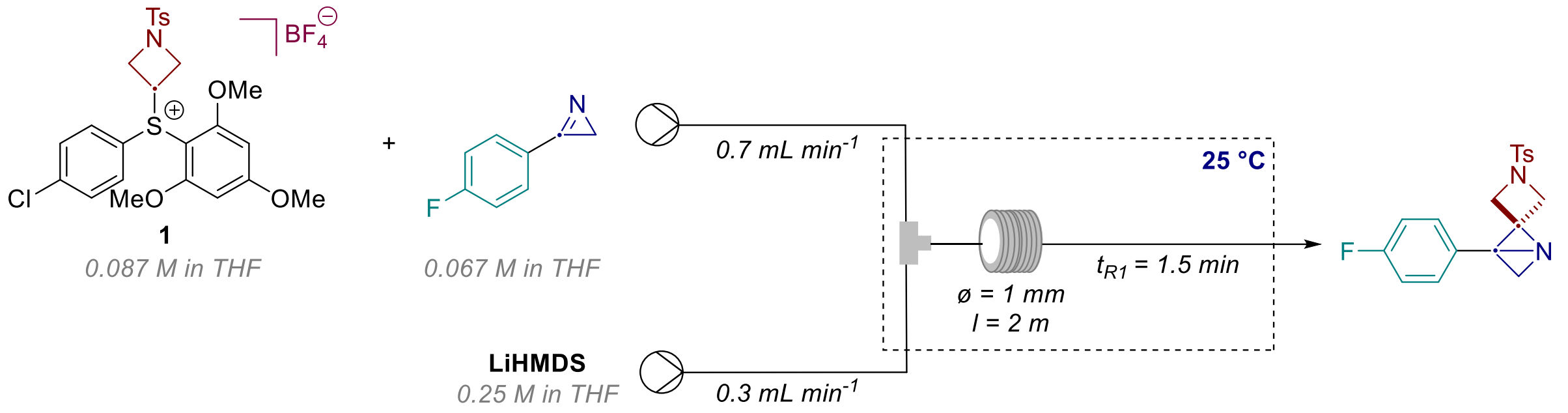


A solution of 3-(4-fluorophenyl)-2*H*-azirine **J** (317 mg, 2.35 mmol) and (4-chlorophenyl)(1-tosylazetidin-3-yl)(2,4,6-trimethoxyphenyl)sulfonium tetrafluoroborate **1** (1.85 g, 3.05 mmol) in THF (35 mL) (flow rate: 0.7 mL/min by Harvard Apparatus PHD ULTRA syringe pump), and a freshly prepared solution of LiHMDS (0.25 M in THF) (flow rate: 0.3 mL/min by Harvard Apparatus PHD ULTRA syringe pump), were introduced to M1 (inner diameter = 0.5 mm) by syringe pumps. The resulting solution was passed through R1 [ϕ1 = 1.0 mm, L1 = 200 cm ($t_{R1}$ = 1.5 min)]. After reaching the steady state (collection for two minutes), the solution was collected continuously for 35 minutes (in 5 min intervals to determine the $^1$H-NMR yield in these intervals) in separate vials containing an excess of methanol as the quench. The averaged $^1$H-NMR yield was 67%. The combined solutions were concentrated under reduced pressure to afford the crude material which was purified by flash column chromatography to afford the title compound **4** as a colourless oil (303 mg, 0.88 mmol, 54%).

Stainless steel (SUS304) T-shaped micromixers with inner diameter of 500 µm were manufactured by Sanko Seiki Co., Inc. PTFA Tubes (1.0 mm inner diameter) were used as reactor and connectors between syringe and T-piece. SGE Gas Tight Syringes, Luer Lock (50 mL) syringes were used for reagent delivery.

# 8. X-Ray Crystallography

The X-ray data were acquired at room temperature with a Bruker AXS X8 APEXII automated diffractometer, equipped with a CCD detector and graphite-monochromatized Mo Kα radiation (λ = 0.71073 Å) operating at 50 kV, 30 mA and 40 mm crystal-to-detector distance. Relevant crystallographic data of the investigated crystals is reported in **Table S2**. The whole Ewald sphere (±h,±k,±l) was recorded up to θ~30° with a scan ranging from 0.5 to 0.8°/frame and an exposure time ranging from 180 to 240 s/frame. The APEX program suite allowed to optimize the collection strategy;[10] the SAINT package [11] was used for the integration of the intensities of reflections and the correction of the Lorentz and polarization effects; the SADABS software[12] was employed for the empirical absorption correction; XPREP[13] was used for the subsequent analysis of the intensity data and the assignment of the space group. Structure was solved by charge flipping method[14] and refined in $P2_1/c$ space group by full-matrix least-square analysis using the program CRYSTALS.[15] All reflections are included in the refinement. The overall scale factor, atomic positions and anisotropic atomic displacement parameters for non-hydrogen atoms were refined. All hydrogen atoms were located and refined isotropically through ride restraints conditions.[16]

***Table S2.*** X-ray crystal data of the studied crystals.

| | Compound **90** | Compound **89** | Compound **47** |
|---|---|---|---|
| Crystal size (mm) | 0.47 × 0.42 × 0.12 | 0.61 × 0.45 × 0.09 | 0.74 × 0.22 × 0.14 |
| Chemical formula | $C_{24}H_{28}ClNO_4S$ | $C_{25}H_{26}ClNO_2$ | $C_{18}H_{17}N$ |
| $M_r$ | 462.01 | 407.92 | 247.34 |
| Crystal system, space group | Orthorhombic, $Pna2_1$ | Triclinic, $P\bar{1}$ | Monoclinic, $P2_1/c$ |
| *a*, *b*, *c* (Å) | 10.3148 (5), 16.6537 (8), 14.0343 (7) | 6.4542 (16), 10.423 (3), 17.010 (5) | 14.3846 (7), 5.6190 (3), 18.3509 (10) |
| a, b, g (°) | 90 90 90 | 93.364 (9), 92.517 (9), 99.939 (8) | 90, 112.832 (2), 90 |
| $V$ (Å$^3$) | 2410.8 (2) | 1123.5 (5) | 1367.03 (13) |
| $R[F^2 > 2s(F^2)]$, $wR(F^2)$, $S$ | 0.066, 0.201, 0.98 | 0.063, 0.061, 0.98 | 0.048, 0.103, 1.00 |
| $Dr_{max}$, $Dr_{min}$ (e Å$^{-3}$) | 0,68, -0,69 | 0.27, -0.36 | 0.25, -0.25 |

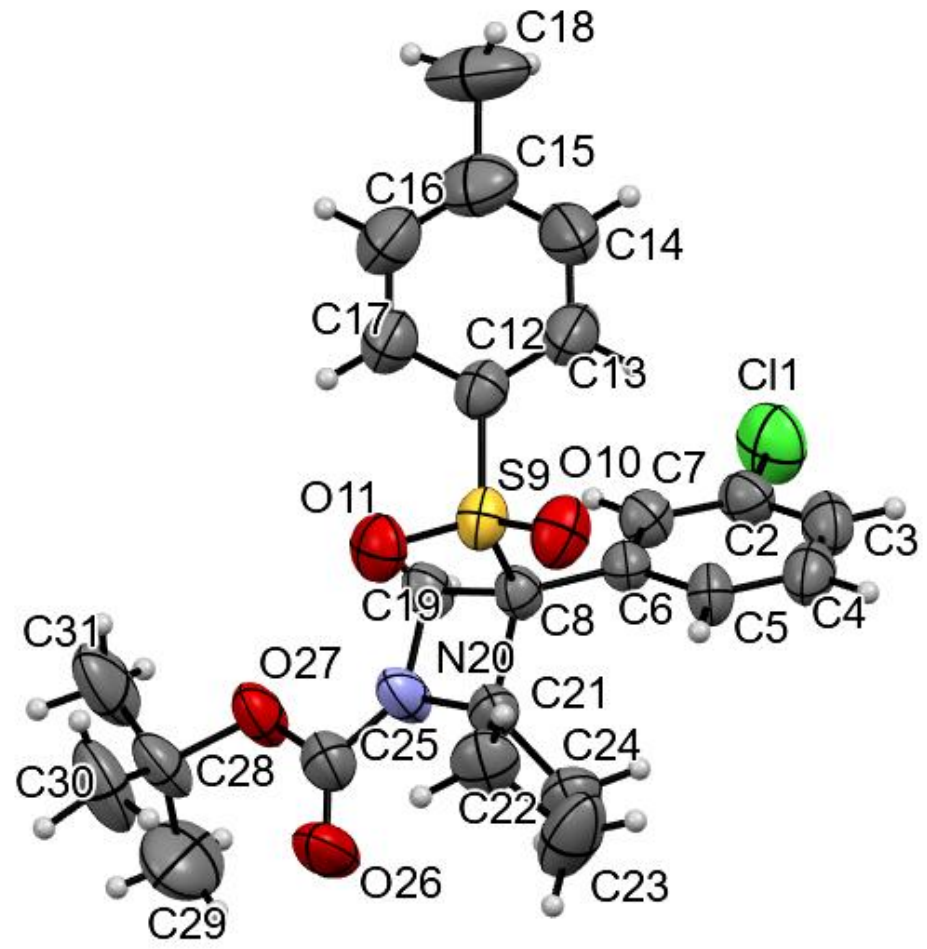


**Figure S1.** ORTEP drawing of compound **90**. The displacement ellipsoids are drawn at the 50% probability level. Hydrogen atoms labels are omitted for clarity.

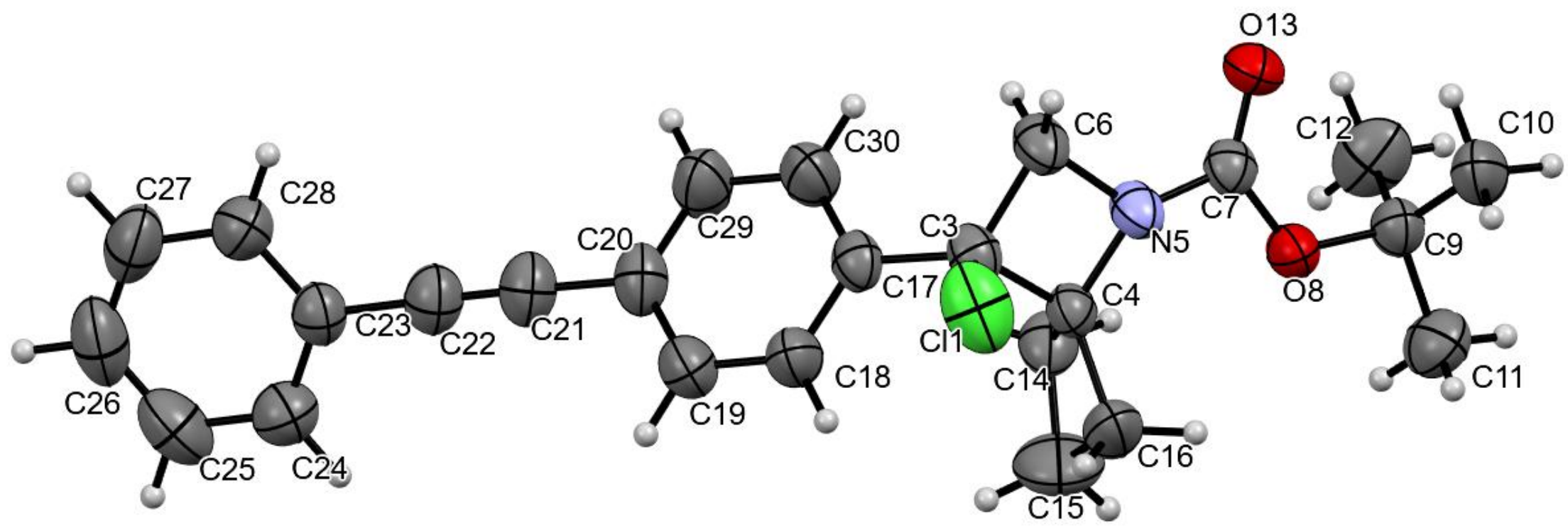


**Figure S2.** ORTEP drawing of compound **89**. The displacement ellipsoids are drawn at the 50% probability level. Hydrogen atoms labels are omitted for clarity.

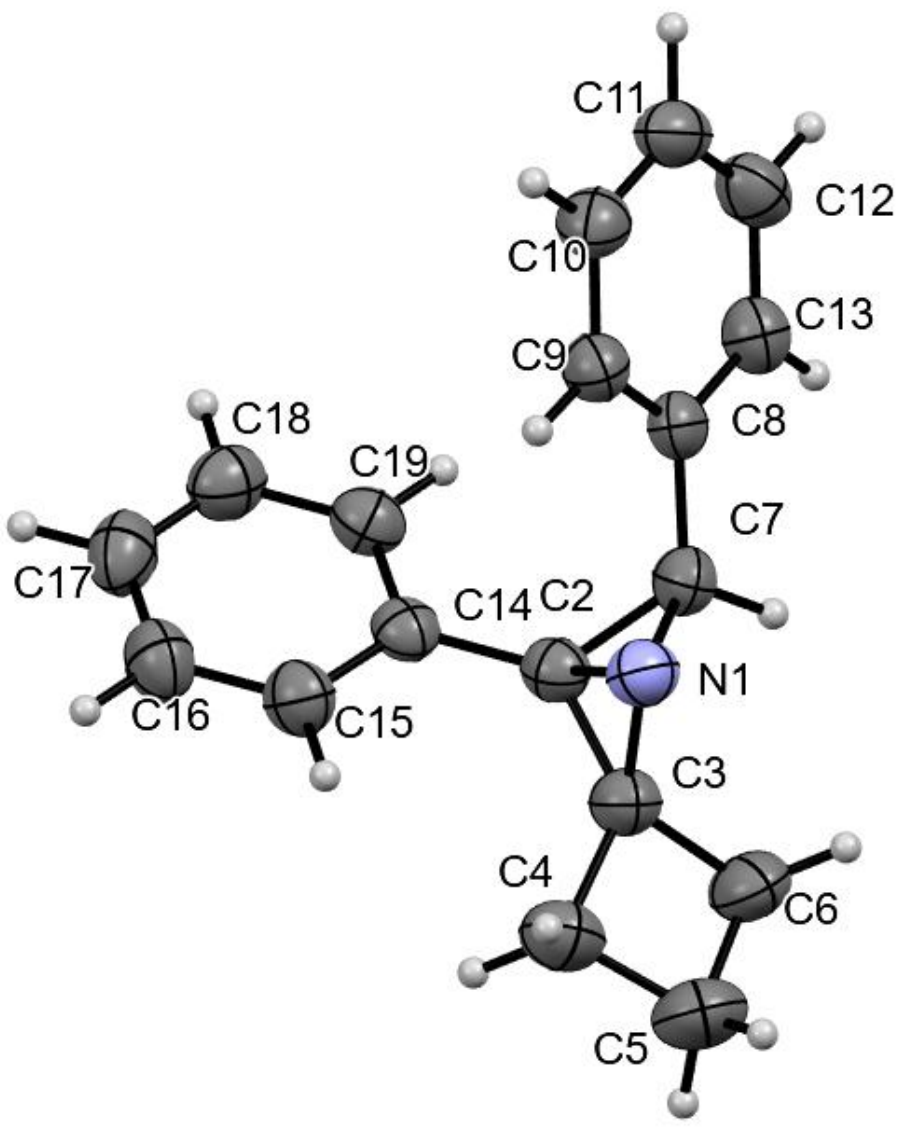


**Figure S3.** ORTEP drawing of compound **47**. The displacement ellipsoids are drawn at the 50% probability level. Hydrogen atoms labels are omitted for clarity.

# 9. Predictive Analytics-Supported Target Fishing

Using the *Predictive Analytics* tool on CAS BioFinder® (https://www.cas.org/solutions/biofinder-discovery-platform) all isolated "super-stained" spiro heterocycles (spirocyclic 1-azabicyclo[1.1.0]butanes) were screened *in silico* for their predicted interaction (pAct). The calculation of pAct is based on the combination of an ensemble of multiple models based on different methodologies that are used together to generate the final prediction. These methodologies are:

- **Similarity-based method (SIM)**: A kNN (k nearest neighbors) method based on the weighted average of the affinities of molecules in the training set similar to the input molecule.
- **Similarity ensemble approach (SEA)**: Based on the distribution of similarities of the input molecule to all the training molecules that are active to a particular target. If this distribution is similar to the average distributions of all those molecules when compared against each other then the input molecule is considered active.
- **Simplest Active Substructure (SAS)**: Given a biological target, a SAS is defined as the simplest active subgraph containing the minimum pharmacophoric features to achieve activity. These subgraphs are detected using MMPs and multiple SAS can be defined for any given target reflecting the diversity of chemical series in active molecules. If the input molecule presents one or more SAS an affinity will be derived based on the affinities of the molecules in the training set presenting that same substructure.
- **Machine Learning Method (MLM)**: This methodology uses molecular descriptors to train machine learning models, with methods such as XGBoost and gNN, and produces estimated affinity values for the input molecule. For each target there is a classifier model that separates molecules into active and inactive and then for the active ones a secondary regressor model produces the predicted affinity value.
- **Cross-Pharmacology Index (XPI)**: This methodology is based on the activity profile of the input molecule including known data, if any, as well as the results produced by the other methods. Known and predicted annotations are used to impute missing target activities for targets related via cross-pharmacology.

All these methods are trained once with each of two different orthogonal molecular descriptors including ECFP4 and FPD, an inhouse implementation similar in concept to MACCS fingerprints. This means that in the end there are 12 models involved in a single prediction.

The final consensus takes into account the affinities predicted by the different methods together with their respective model metrics to aggregate them into a single affinity value. The confidence score is produced by an additional ML model that learns to project the native confidence scores of the different methodologies into the error space so that for a confidence score of 0.5 the expected error is of 0.5 log units or lower.

Before training the different models, input data is standardized and thoroughly deduplicated at the molecular fingerprint level. For model building a ratio of 10 to 1 between negatives and positives is forced on every model, adding random molecules as negatives if the original data does not have enough. Finally, a five-fold validation is used to generate the metrics of all the individual models as well as the consensus.

Using this model, the molecular structures were screened against a standard set ("Full Screen") available on CAS BioFinder® containing 183 different receptors.

The results were subsequently ranked according to the number of hits by receptor. The top 10% of receptors with the highest number of hits were then evaluated according to

- Confidence scores
- pAct
- Expertise/feasibility for *in vitro* testing

The full data obtained from the predictive analytics screen is available as an Excel file as a separate Supporting Information file.

## 10. In vitro studies

**Materials:** All cell culture reagents were purchased from S.I.A.L. S.r.l. (Rome, Italy), [$^3$H]-(+)-Pentazocine (25 Ci/mmol) was purchased from Revvity (Milan, Italy), haloperidol and all binding reagent obtained from Sigma-Aldrich (Milan, Italy).

**Cell Culture:** HEK293-S1R, human embryonic kidney cells stably transfected with human sigma-1 receptor (S1R), were obtained from Olivier Soriani, Inserm/CNRS, Institut de Biologie Valrose, Université de Nice, Nice, France. HEK293-S1R cells were cultured in RPMI1640 supplemented with 10% fetal bovine serum, 2 mM L-glutamine, 100 U/mL penicillin, and 100 µg/mL streptomycin and maintained at 37 °C in a humidified 5% $CO_2$ atmosphere.

**Competition Binding Assay:** Sigma-1 competition binding assay was carried out as previously reported with minor modification.[17] The experiments were performed in MultiScreen plates (Merck Millipore) with glass fiber filters (GF/C), presoaked in 0.3% poly(ethylenimine) for 20 min. After this time, 150 µg of HEK293-S1R membranes, ~2 nM [$^3$H]-(+)-Pentazocine, and reference or test compounds were suspended in 0.25 mL of incubation buffer (50 mM Tris-HCl pH 8.0). The samples were incubated for 120 min at 25 °C. The incubation was stopped by rapid filtration, and the filters were washed with 2 x 0.25 mL of ice-cold buffer (50 mM Tris-HCl, pH 8.0). Nonspecific binding was determined in the presence of 10 µM haloperidol. Approximately 90% of specific binding was determined under these conditions. Concentrations required to inhibit 50% of radioligand specific binding ($IC_{50}$) were determined using six to nine different concentrations of the compound studied in two or three experiments with samples in duplicate. Apparent inhibition constants ($K_i$) were determined by nonlinear curve fitting, using the Prism, version 5.0, GraphPad software.

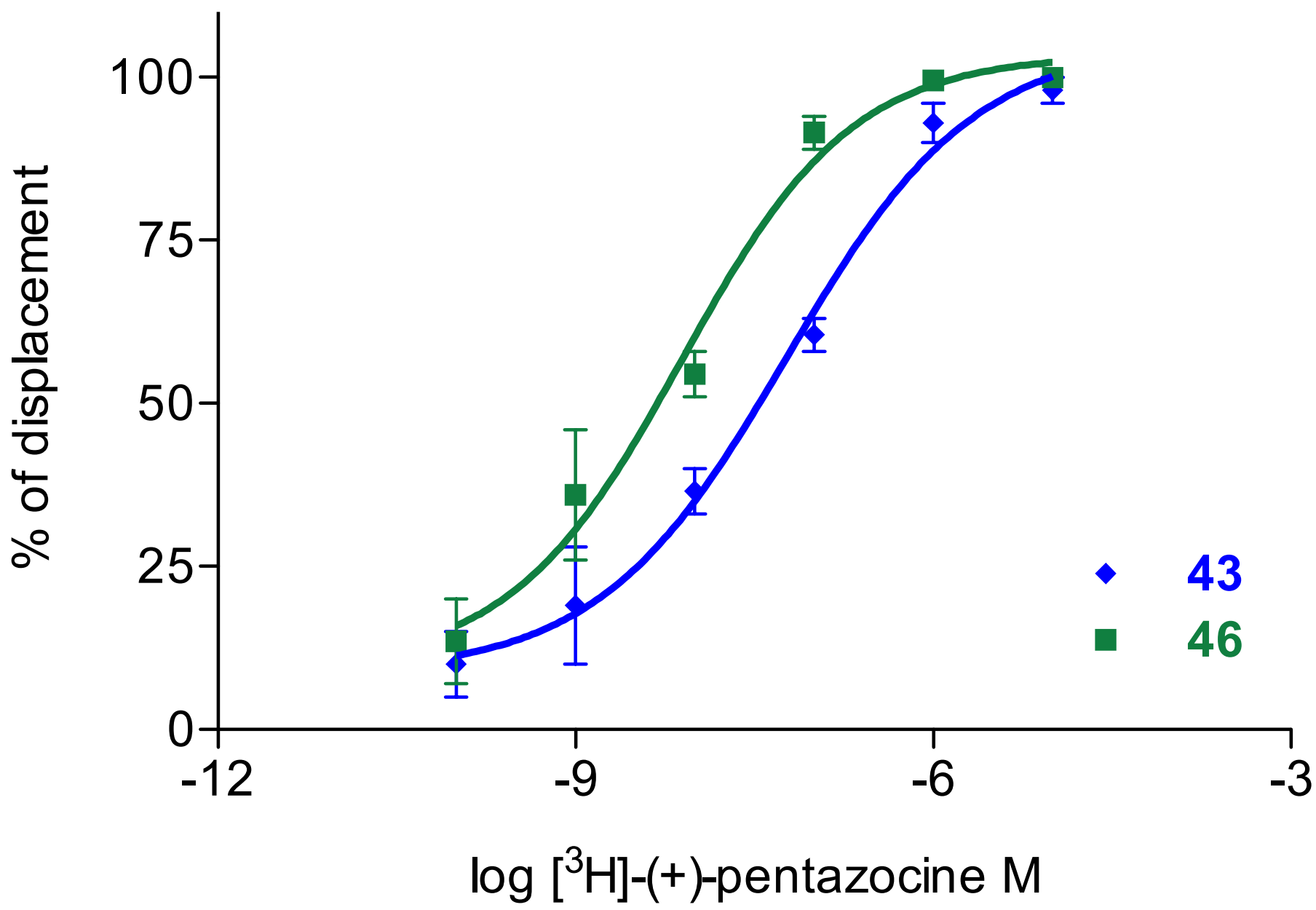


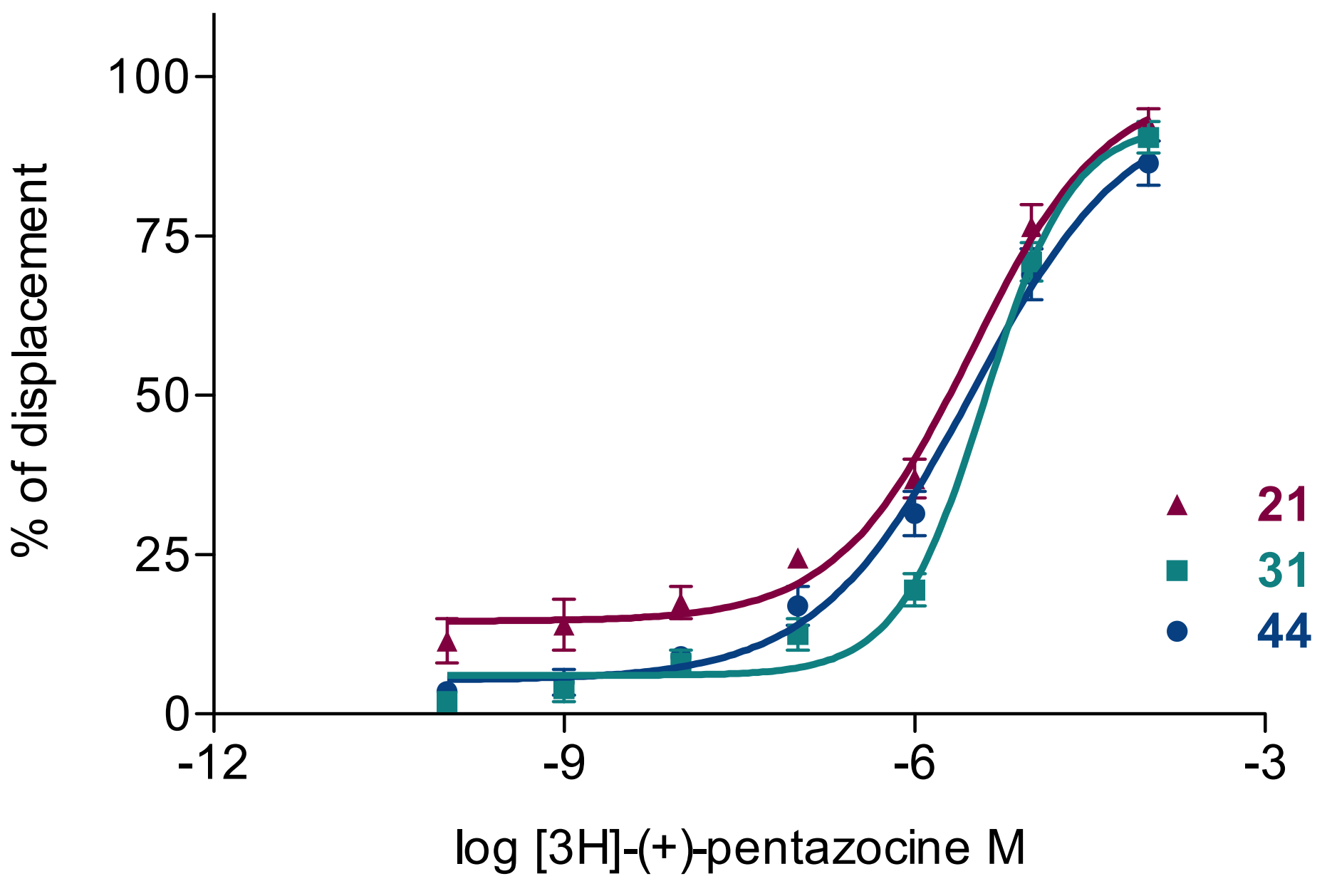


*Concentration-Response curves for compounds **43**, **46**, **21**, **31**, **44***

| Entry | Structure | σ1 Receptor Ki (nM) |
|---|---|---|
| **1** | 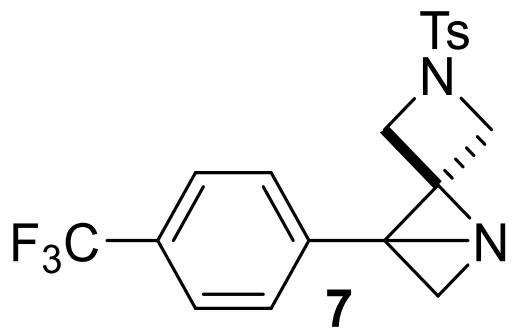 **7** | >8000 (19%) |
| **2** | 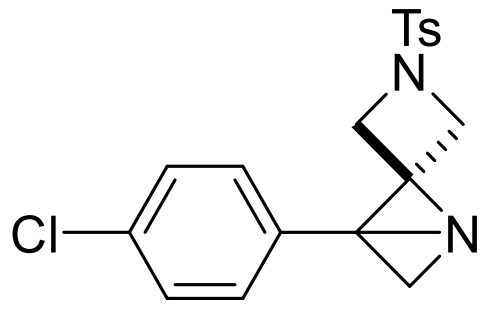 **8** | >8000 (38%) |
| **3** | 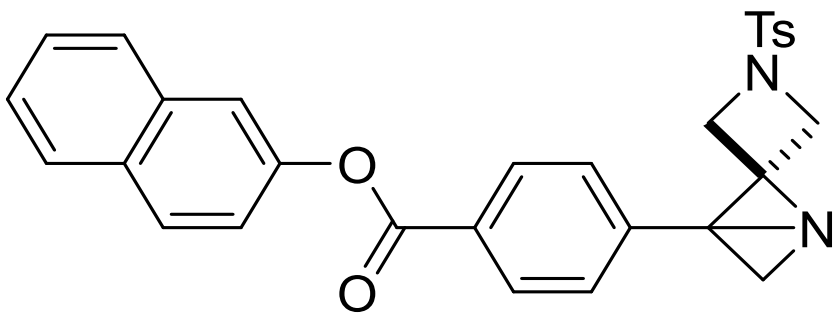 **11** | >8000 (33%) |
| **4** | 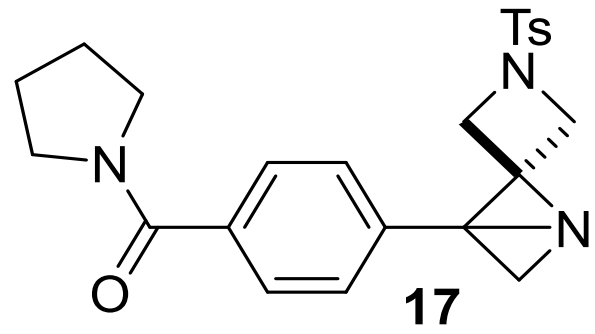 **17** | >8000 (17%) |
| **5** | 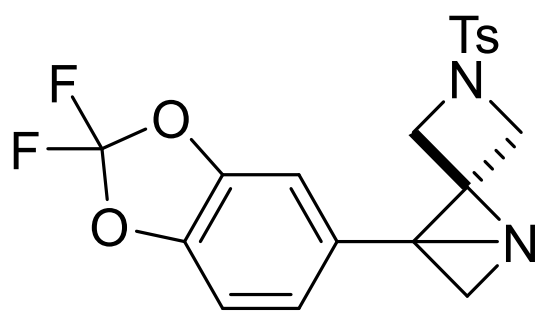 **21** | 2532 ± 125 |
| **6** | 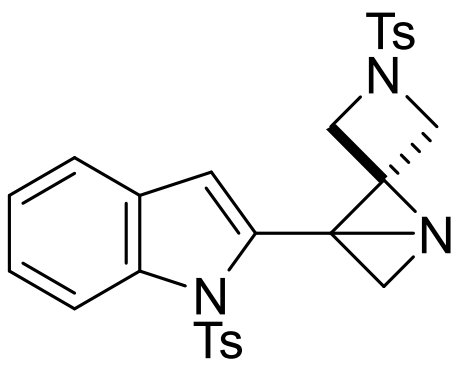 **22** | >8000 (27%) |
| **7** | 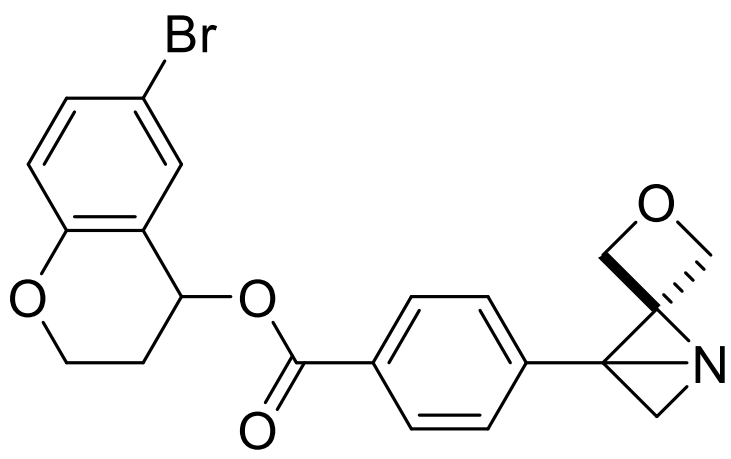 **31** | 3219 ± 210 |

| | | |
|---|---|---|
| **8** | 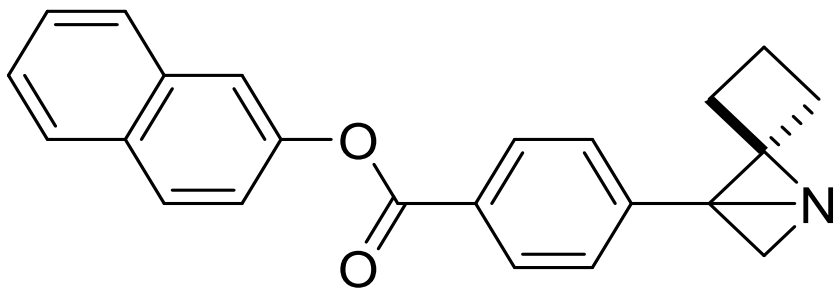 **43** | 64.3 ± 14.8 |
| **9** | 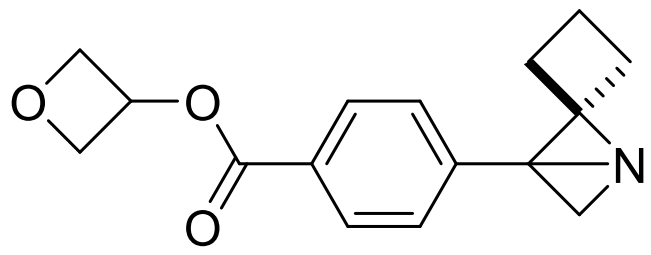 **44** | 2586 ± 110 |
| **10** | 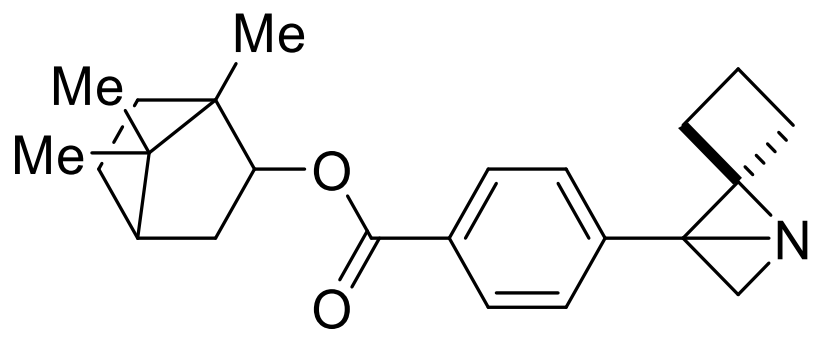 **46** | 6.71 ± 0.52 |
| **11** | 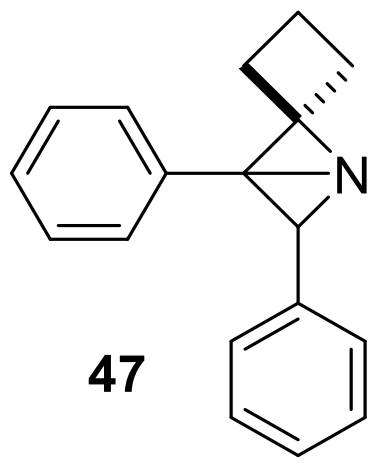 **47** | >8000 (5%) |

***Table S3***: *In vitro* binding results for selected ligands

## 11. Note on $^{13}$C-Spectra of 1-Azaspiro[3.3]heptane Analogues

For several reported 1-azaspiro[3.3]heptane analogues in which the nitrogen-atom is protected with a carbonyl-bearing protecting group (e.g., *N*-Boc, or amides), the $^{13}$C signal for the carbonyl group is only weak and broad, or not visible at all. An increase in acquisition time (i.e., higher number of scans) or concentration of the sample does not lead to a significant improvement. An increase in the relaxation delay (d1, 8 sec.) leads to a small improvement. The reason for the carbonyl signal not or being only weakly observed is a known consequence of loss of transversal magnetization. This is likely due to the slowly rotating carbonyl group due to efficient transverse relaxation driven by significant chemical shift anisotropy and nearby dipolar interactions. When a carbonyl group rotates slowly (high rotational correlation time, ($\tau_c$)), the large anisotropy of its electron cloud results in a very fast dephasing of spin coherence, leading to broad, weak signals, or complete loss of the FID signal, as observed also in our cases.[18]

To address this issue, we run another experiment on the same sample at higher temperature (50 °C) in order to decrease the correlation time. Despite the increased bond rotation and hence decreased correlation time, the signal remained extremely weak and broad, however became somewhat visible. Readers and practitioners should be aware of this observation when reproducing this work.

A sample case study visualizing the described observation is shown below for compound **71**.

**$^{13}$C{$^1$H}-NMR** (101 MHz, $CDCl_3$): 25 °C, 2000 scans, 2s relaxation delay

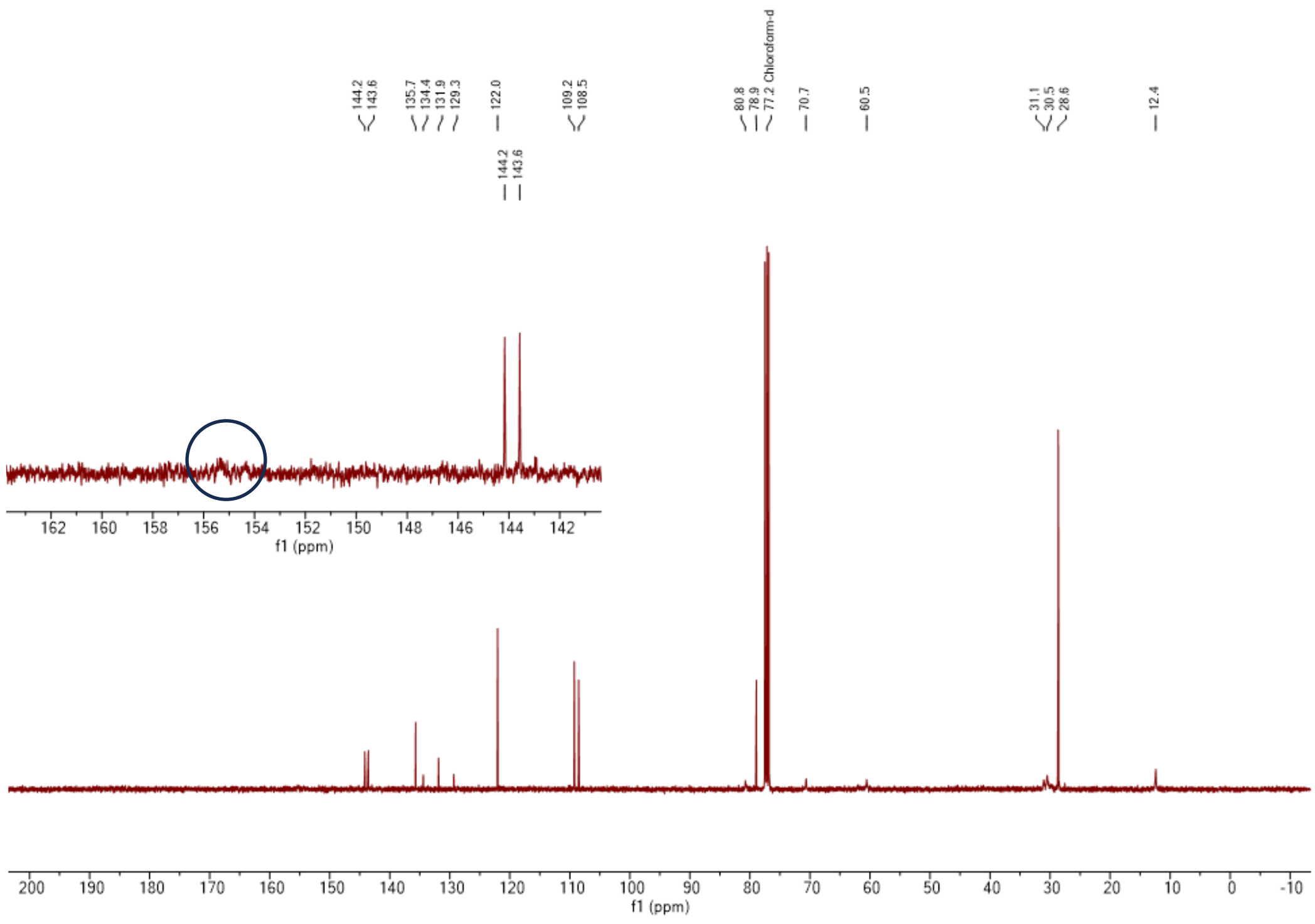

**$^{13}C\{^1H\}$-NMR** (101 MHz, $CDCl_3$): 25 °C, 2000 scans, 6s relaxation delay

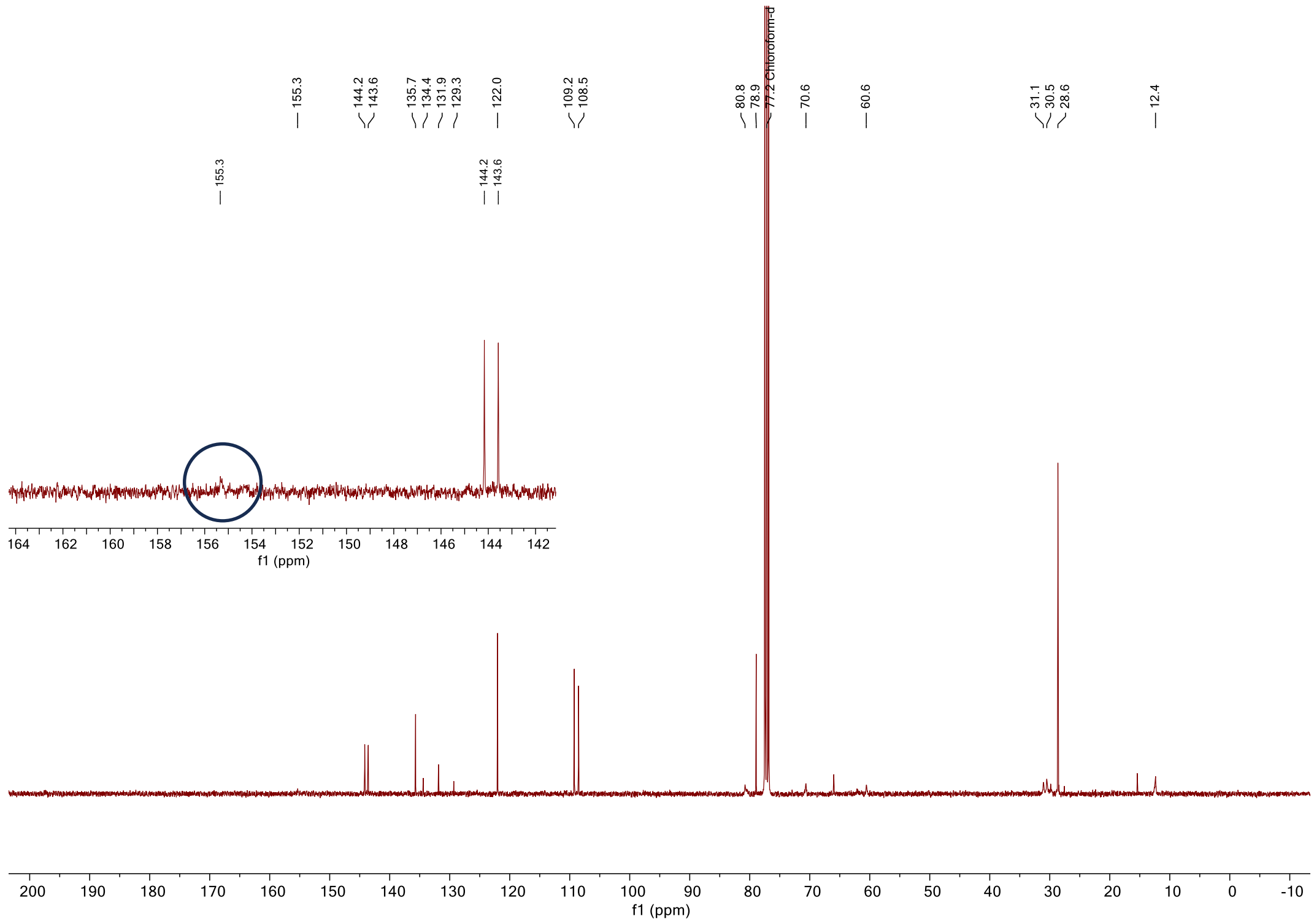


**$^{13}C\{^1H\}$-NMR** (101 MHz, DMSO-$d_6$): 50 °C, 2000 scans, 8 s relaxation delay

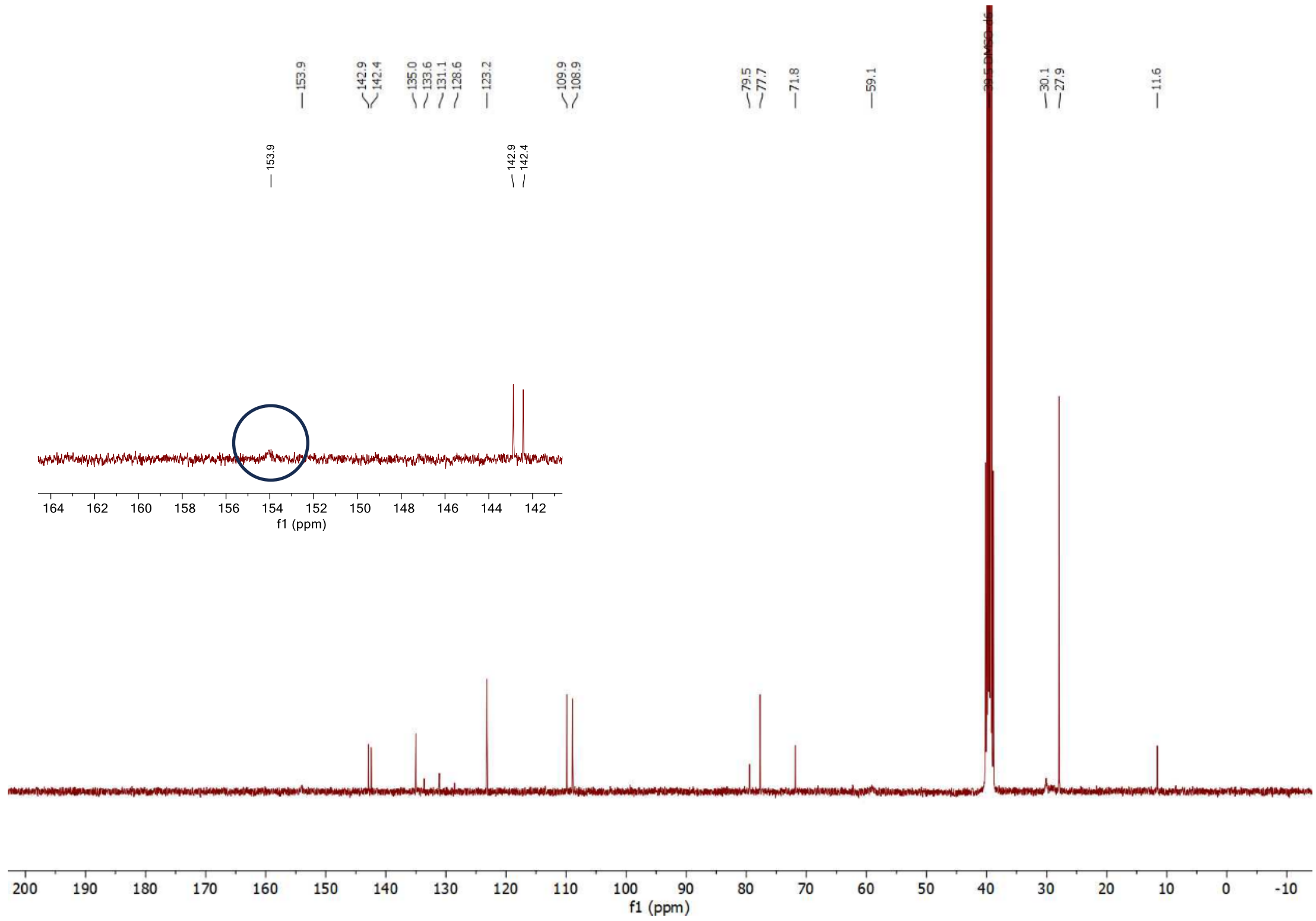

## 12. References


[1] P. Natho, A. Vicenti, F. Mastrolorito, F. De Franco, L. Walsh-Benn, M. Colella, E. Mesto, E. Schingaro, O. Nicolotti, A. Gioiello, R. Luisi, “Novel Sulfonium Reagents for the Modular Synthesis of Spiro[2.3]Hexanes and Heteroatom-Containing Analogues: Synthesis, Application, and Evaluation as Bioisosteres” *Angew. Chem. Int. Ed.* **2026**, *65*, e21633.

[2] N. S. Y. Loy, S. Kim, C.-M. Park, “Synthesis of Unsymmetrical Pyrazines Based on α-Diazo Oxime Ethers” *Org. Lett.* **2015**, *17*, 395–397.

[3] M. Andresini, L. Degennaro, R. Luisi, “A sustainable strategy for the straightforward preparation of 2H-azirines and highly functionalized NH-aziridines from vinyl azides using a single solvent flow-batch approach” *Beilstein J. Org. Chem.* **2021**, *17*, 203–209.

[4] H. Hu, X. Cai, Z. Xu, X. Yan, S. Zhao, “Beckmann Rearrangement of Ketoximine Catalyzed by N-methyl-imidazolium Hydrosulfate” *Molecules*, **2018**, *23,* 1764.

[5] L. Feng, C. Yang, W. Xia, “Visible-Light Promoted Selective Imination of Unactivated C–H Bonds via Copper-nitrene Intermediates for the Synthesis of 2H-Azirines” *Org. Lett.* **2019**, *21*, 8323–8327.

[6] L. Janecký, A. Madabeni, E. Jelínková, B. Klepetářová, L. Rulíšek, P. Beier, “Reactivity of 2H-Azirines in Copper-Catalyzed Azide–Alkyne Cycloaddition Reactions” *Org. Lett.* **2025**, *27*, 10077–10081.

[7] L. L. Mittapelli, K. R. Gore, “LiHMDS: Facile, highly efficient and metal-free transesterification under solvent-free condition” *Catal. Commun.* **2021**, *149*, 106194.

[8] Y. Yang, H. Jiang, Y. Dai, K. Tang, B. Pan, H. Jin, X. Chen, **2025**, Research Square preprint, Modular Assembly of Bioisosteric Bridged Aza-frameworks via Strained Ring Release, DOI: 10.21203/rs.3.rs-7615919/v1.

[9] A. Scuiller, A. Dupas, G. Lefebvre, N. Bouriche, H. Chédotal, G. Guillamot, J. Cossy, C. Meyer, “Synthesis of 2-(Trifluoromethyl)Azetidines by Strain-Release Reactions of 2-(Trifluoromethyl)-1-Azabicyclo[1.1.0]Butanes” *Chem. Eur. J.* **2025**, *31*, e202500590.

[10] APEX 2, version 2010.3-0, Bruker (2010), Bruker AXS Inc., Madison, Wisconsin, USA

[11] SAINT, version V7.60A, Bruker (2009), Bruker AXS Inc., Madison, Wisconsin, USA

[12] SADABS, version 2008/1, Bruker (2008), Bruker AXS Inc., Madison, Wisconsin, USA

[13] Sheldrick, G.M. (2008) XPREP Version 2008/2. Bruker AXS Inc., Madison

[14] L. Palatinus, G. Chapuis *J. Appl. Cryst.* **2007**, 40, 786-790

[15] R.I. Cooper, A. L. Thompson, D. J. Watkin *J. Appl. Crystallogr.* **2010**, 43,1100-1107

[16] P. W. Betteridge, J. R. Carruthers, R. I. Cooper, K. Prout, & D. J. Watkin, *J. Appl. Cryst.* (**2003**), 36, 1487

[17] F. Mastropasqua, A. T. Lisi, L. Crouzier, G. R. Musillo, F. S. Abatematteo, M. Niso, B. Delprat, N. A. Colabufo, V. Nanna, G. F. Mangiatordi, P. Delre, T. Spalholz, M. Colella, W. Deuther-Conrad, F. Loiodice, T. Maurice, A. Laghezza, C. Abate, "Development of Second-Generation Phenoxyethylpiperidines as Potent Sigma-1 Receptor Agonists with Neuroprotective Potential for Alzheimer's Disease" *J. Med. Chem.* **2026**, *69*, 4613–4628.

[18] Friebolin H. Basic One- and Two-Dimensional NMR Spectroscopy. 5th edition ed. Wiley-VCH; Weinheim, Germany: 2010. p. 418.

# 13. NMR spectra

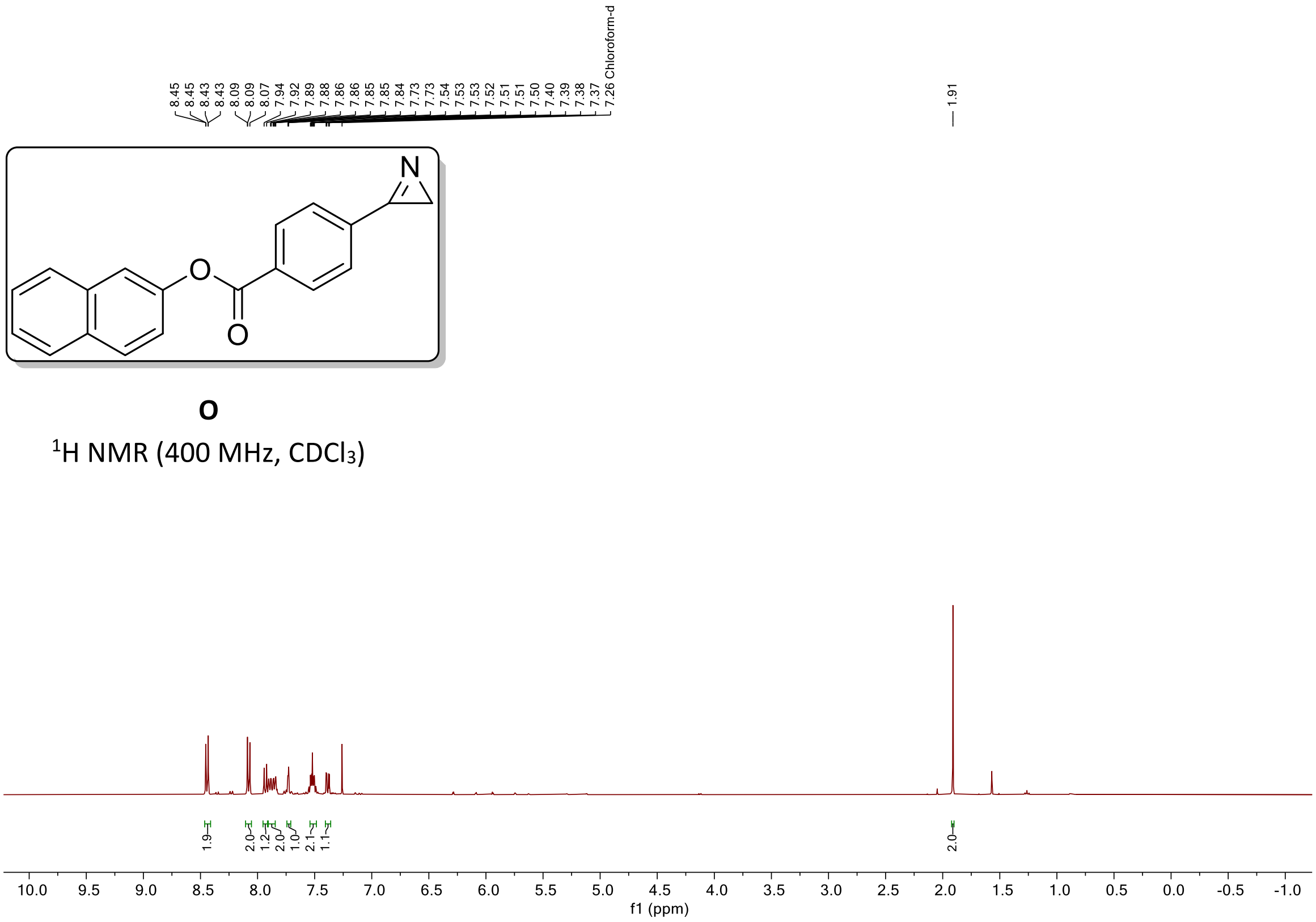


**O**

$^{1}H$ NMR (400 MHz, $CDCl_3$)

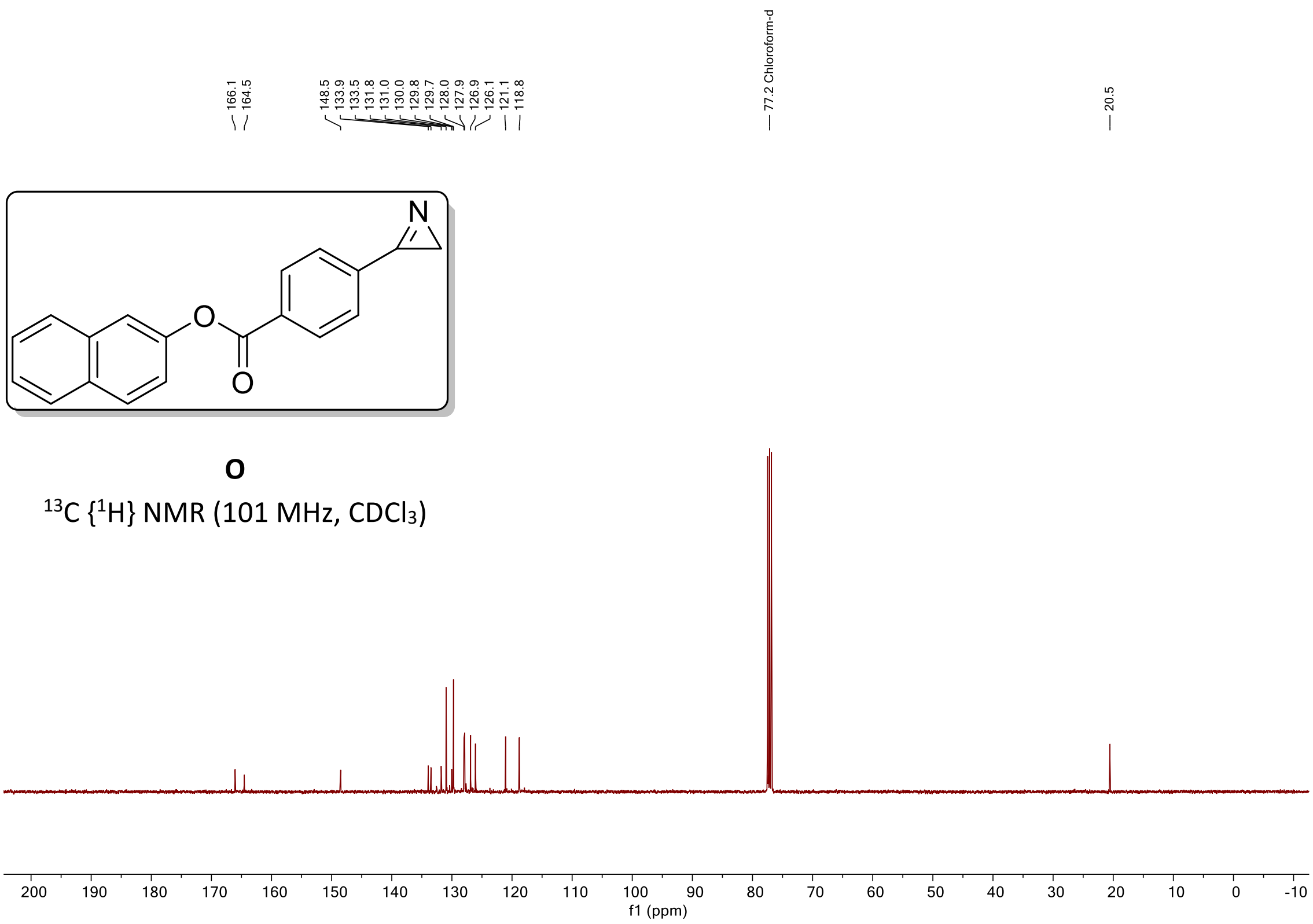


**O**

$^{13}C$ {$^{1}H$} NMR (101 MHz, $CDCl_3$)

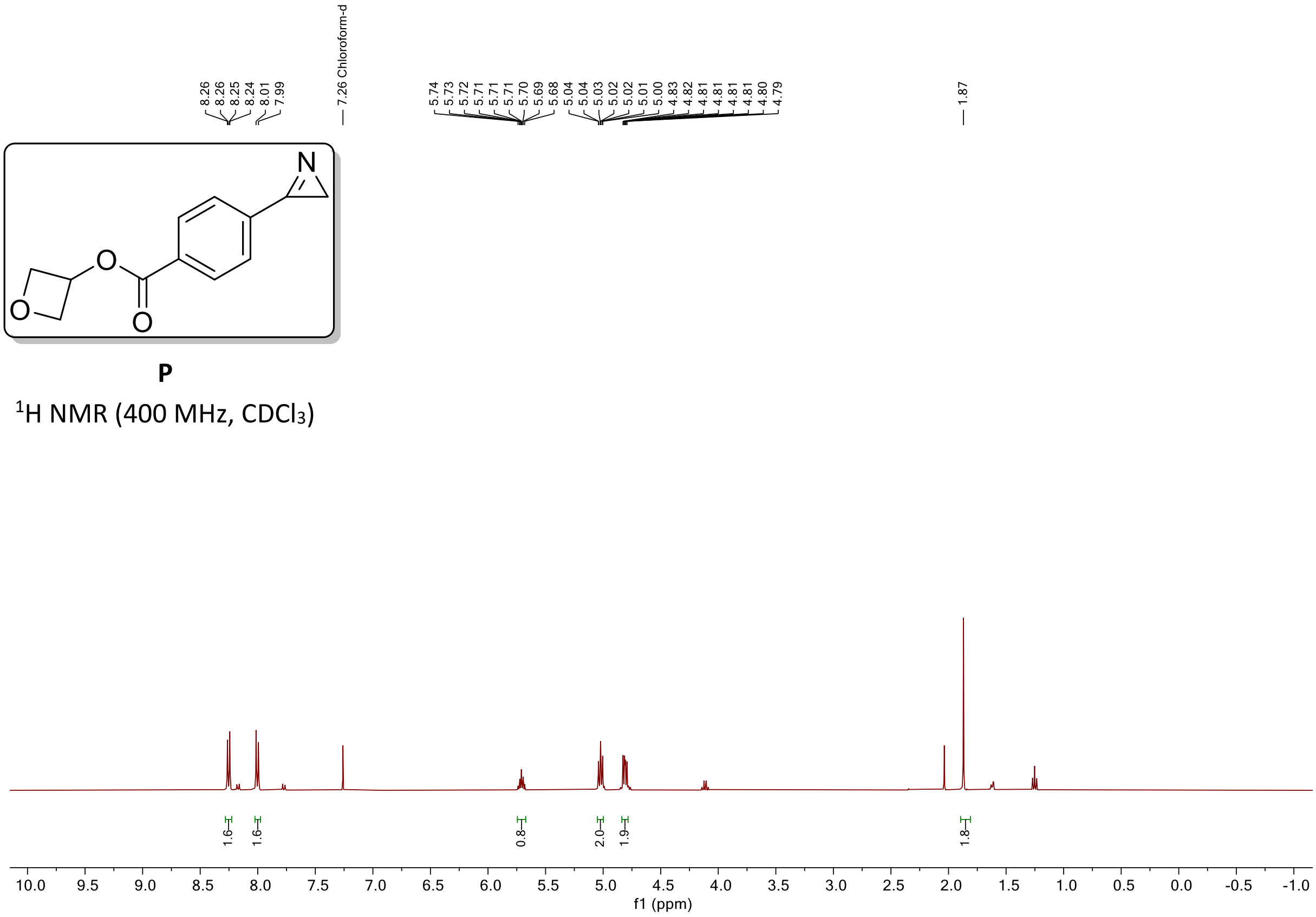


**P**

$^{1}H$ NMR (400 MHz, $CDCl_3$)

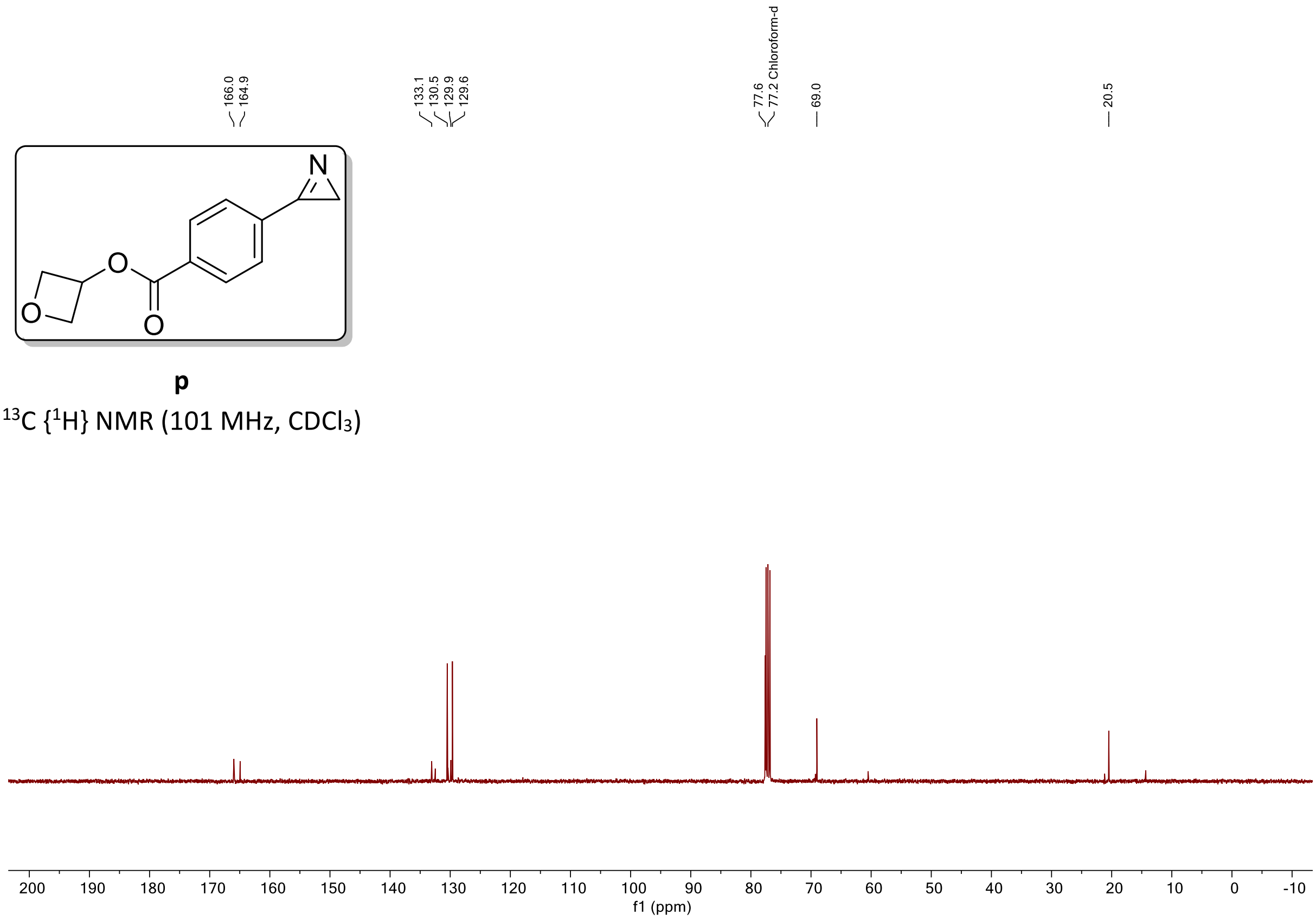


**p**

$^{13}C$ {$^{1}H$} NMR (101 MHz, $CDCl_3$)

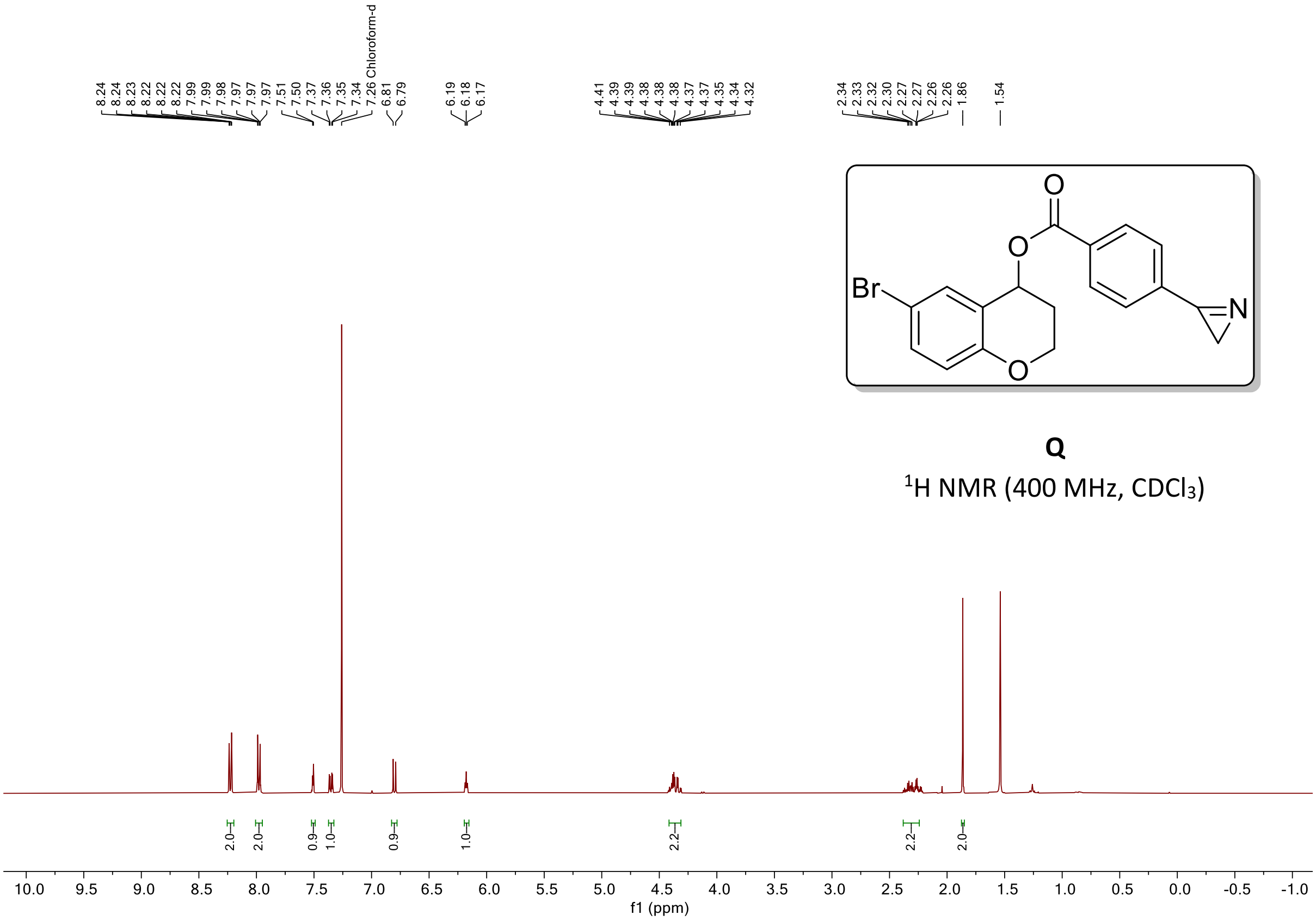

**Q**

$^1H$ NMR (400 MHz, $CDCl_3$)

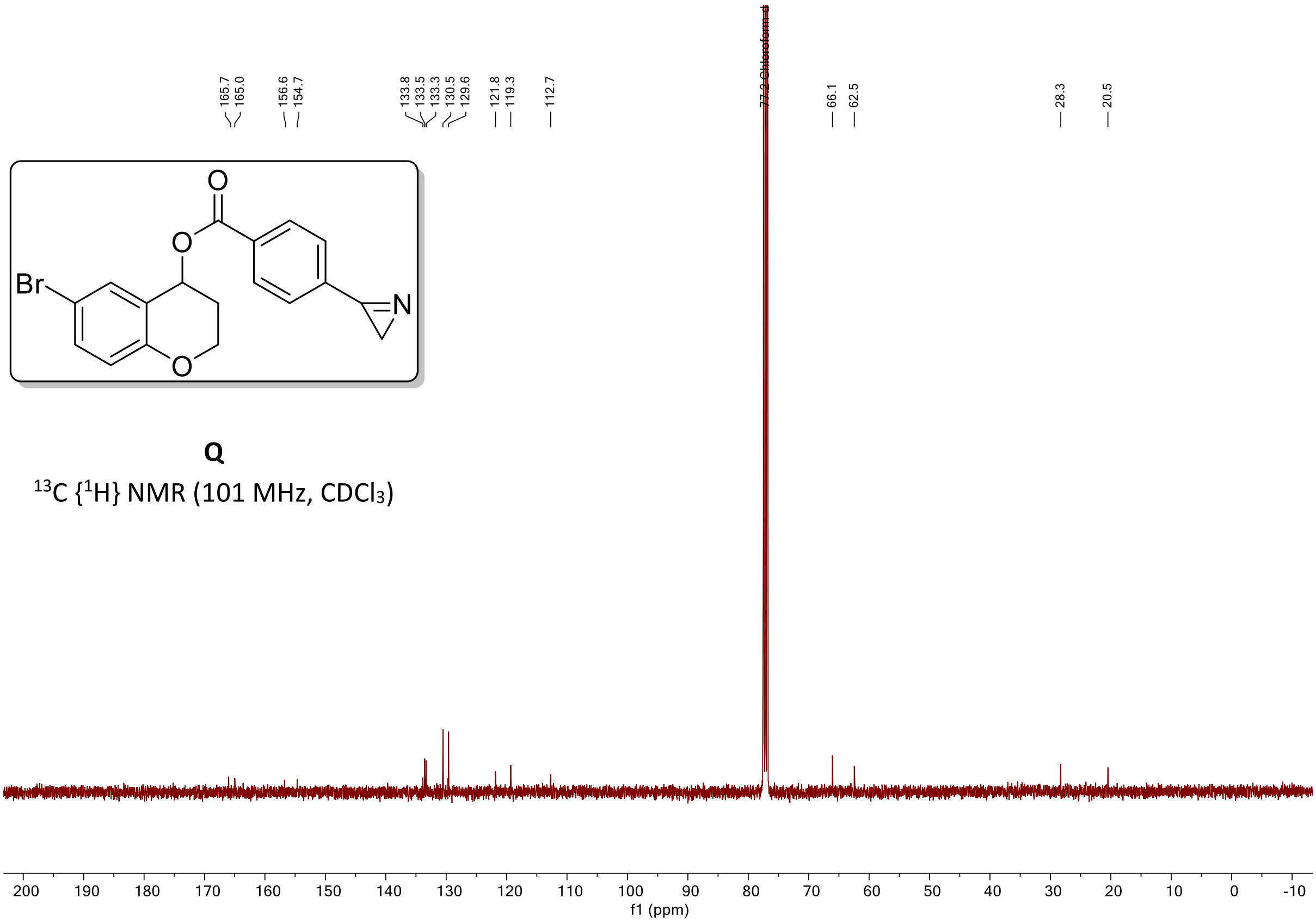

**Q**

$^{13}C$ {$^1H$} NMR (101 MHz, $CDCl_3$)

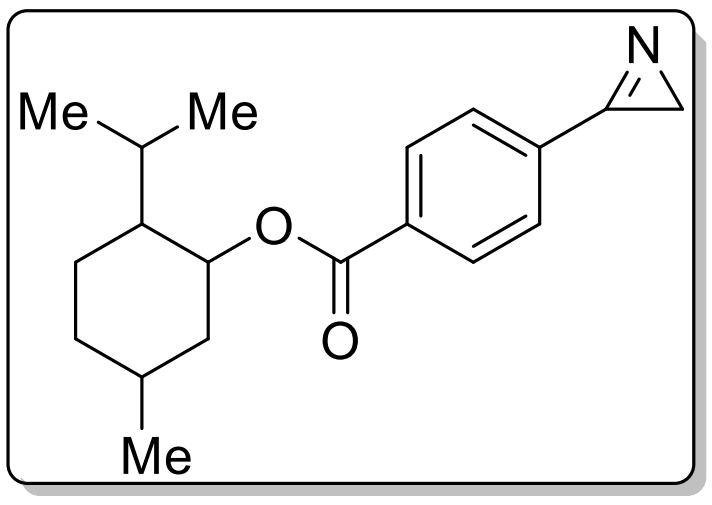

**R**

$^{1}H$ NMR (400 MHz, $CDCl_3$)

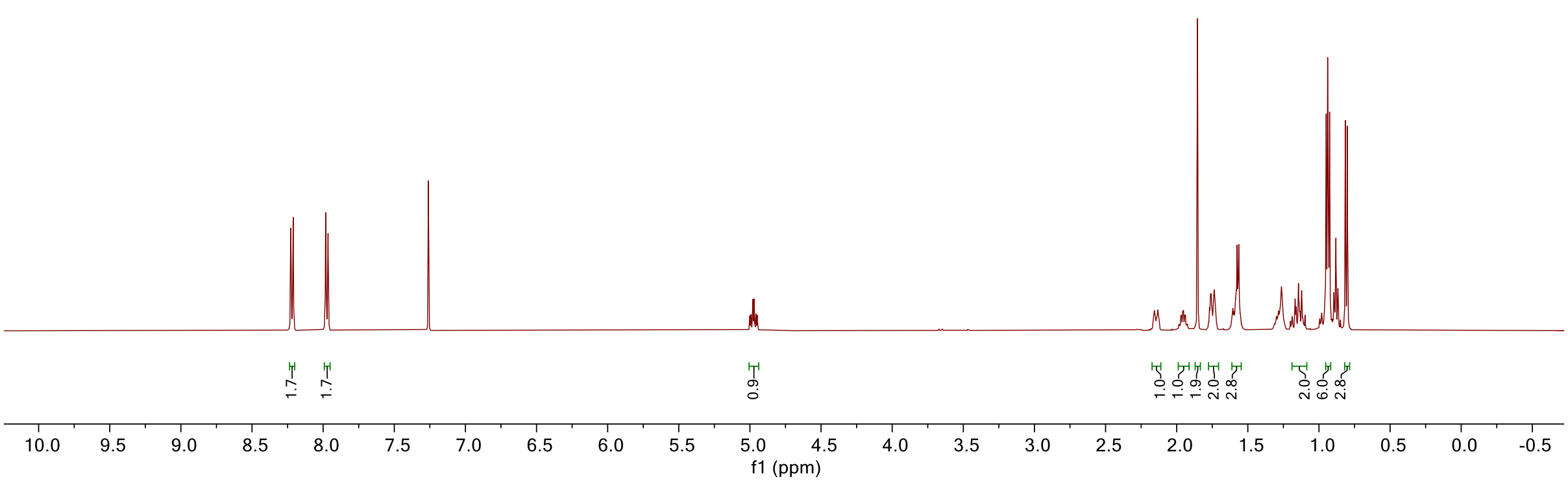

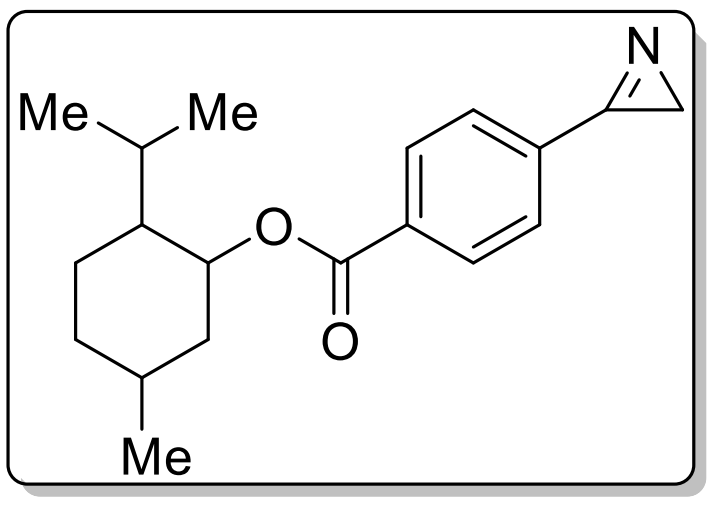

**R**

$^{13}C$ {$^{1}H$} NMR (101 MHz, $CDCl_3$)

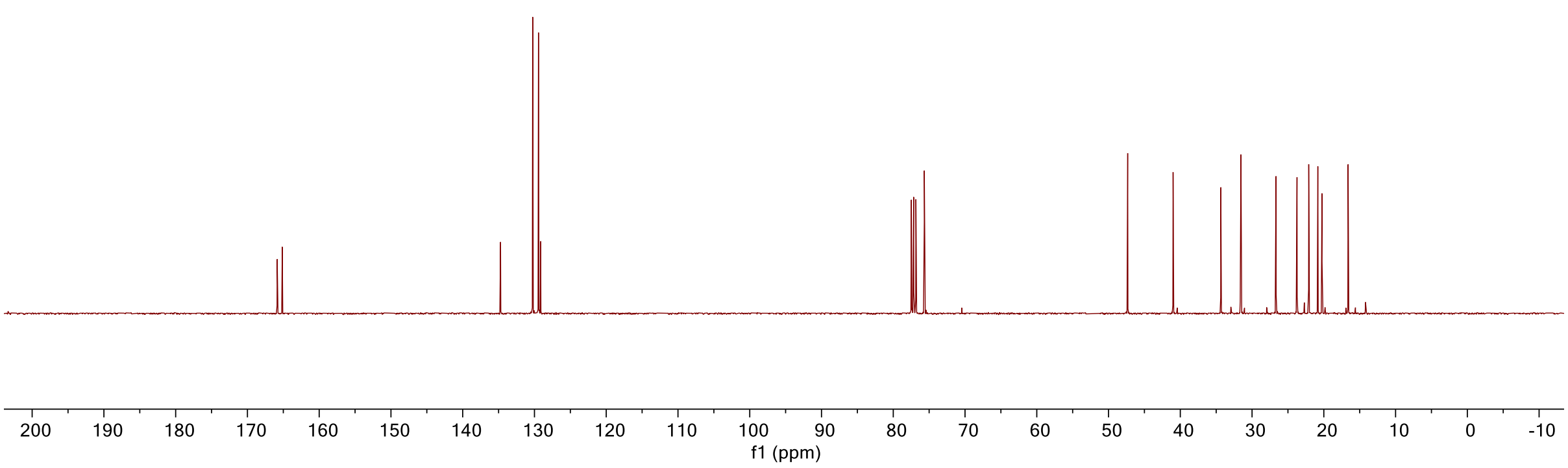

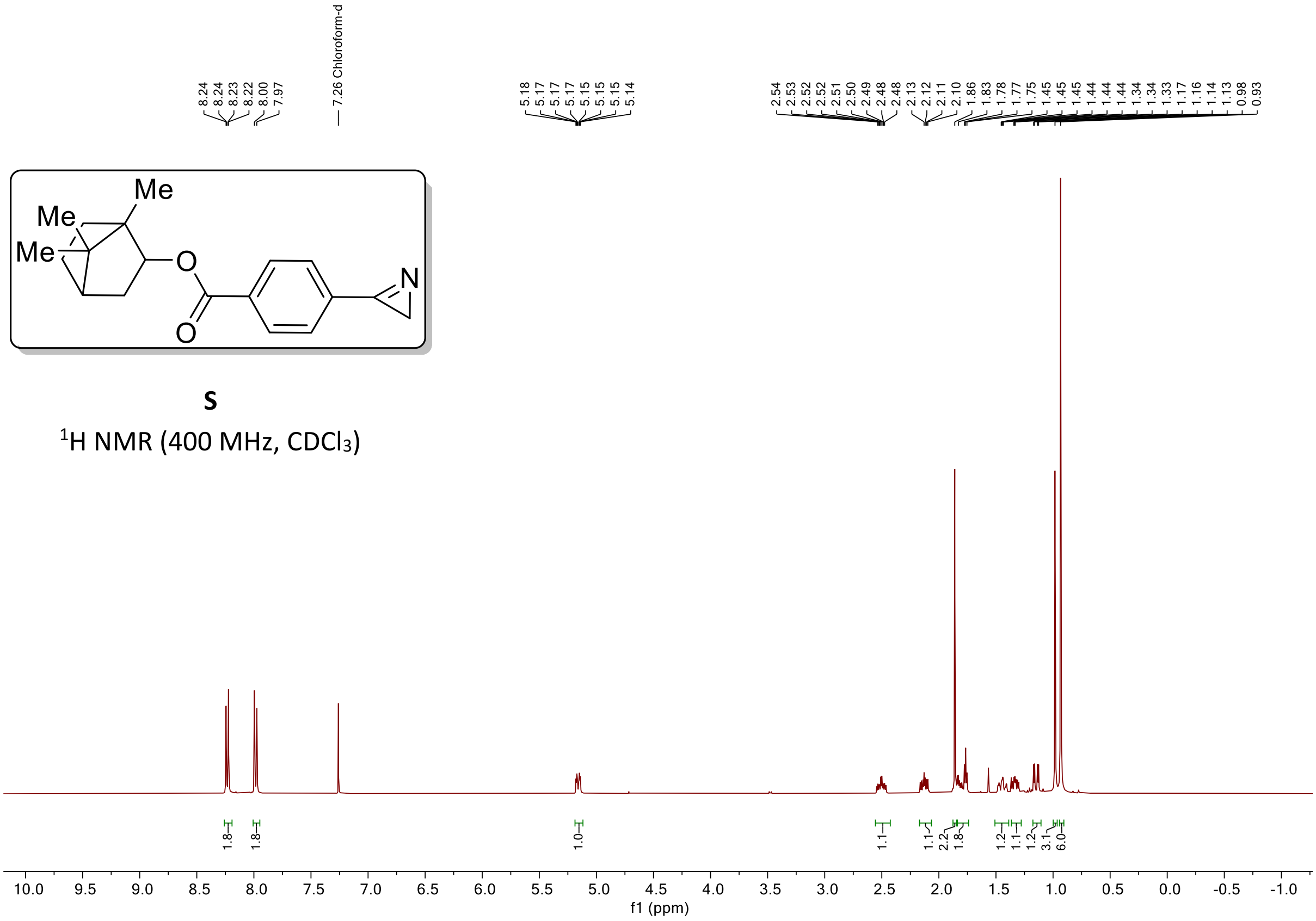
Me
Me
Me
O
O
N
S
1H NMR (400 MHz, CDCl3)
7.26 Chloroform-d
f1 (ppm)

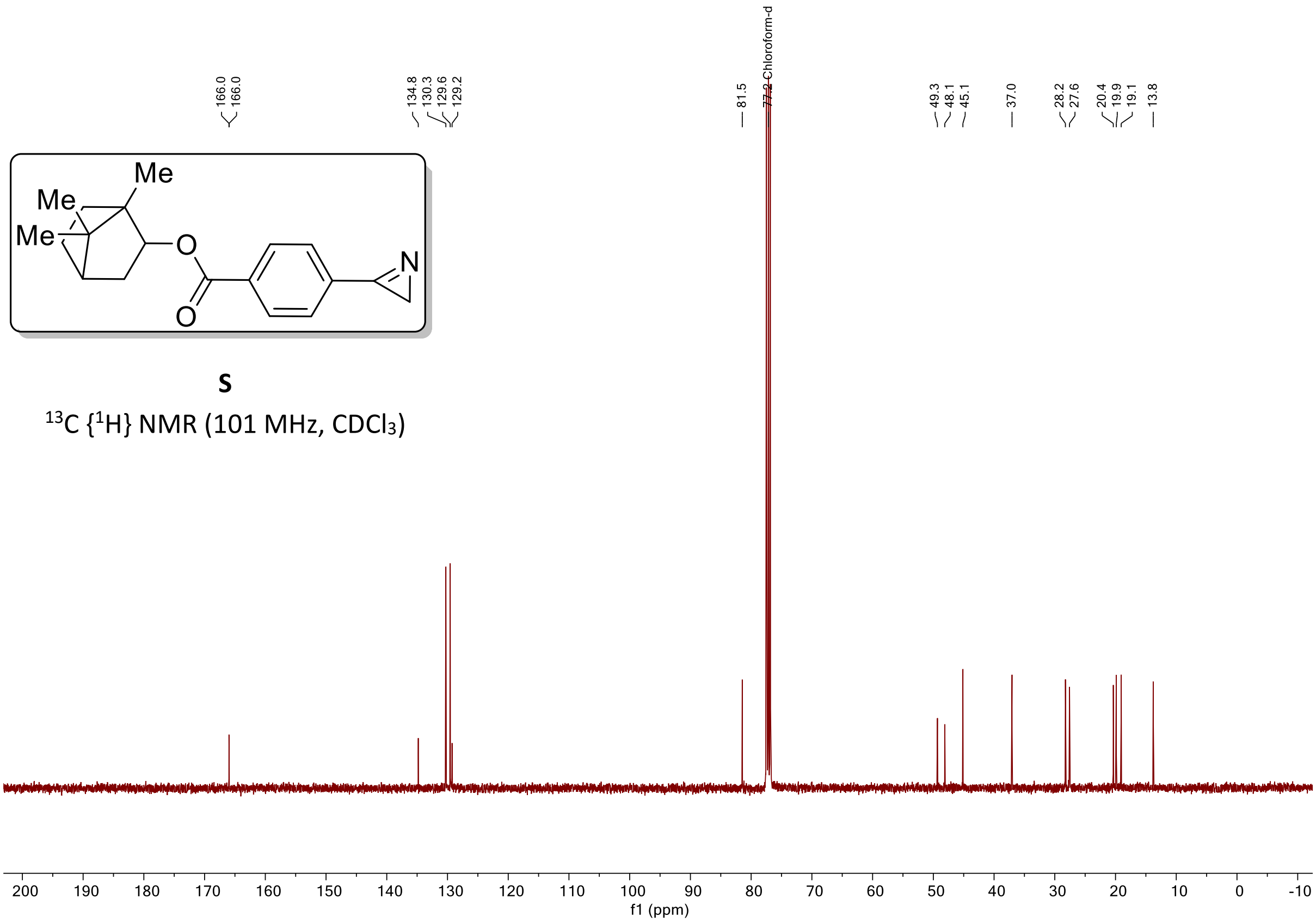
Me
Me
Me
O
O
N
S
13C {1H} NMR (101 MHz, CDCl3)
77.2 Chloroform-d
f1 (ppm)

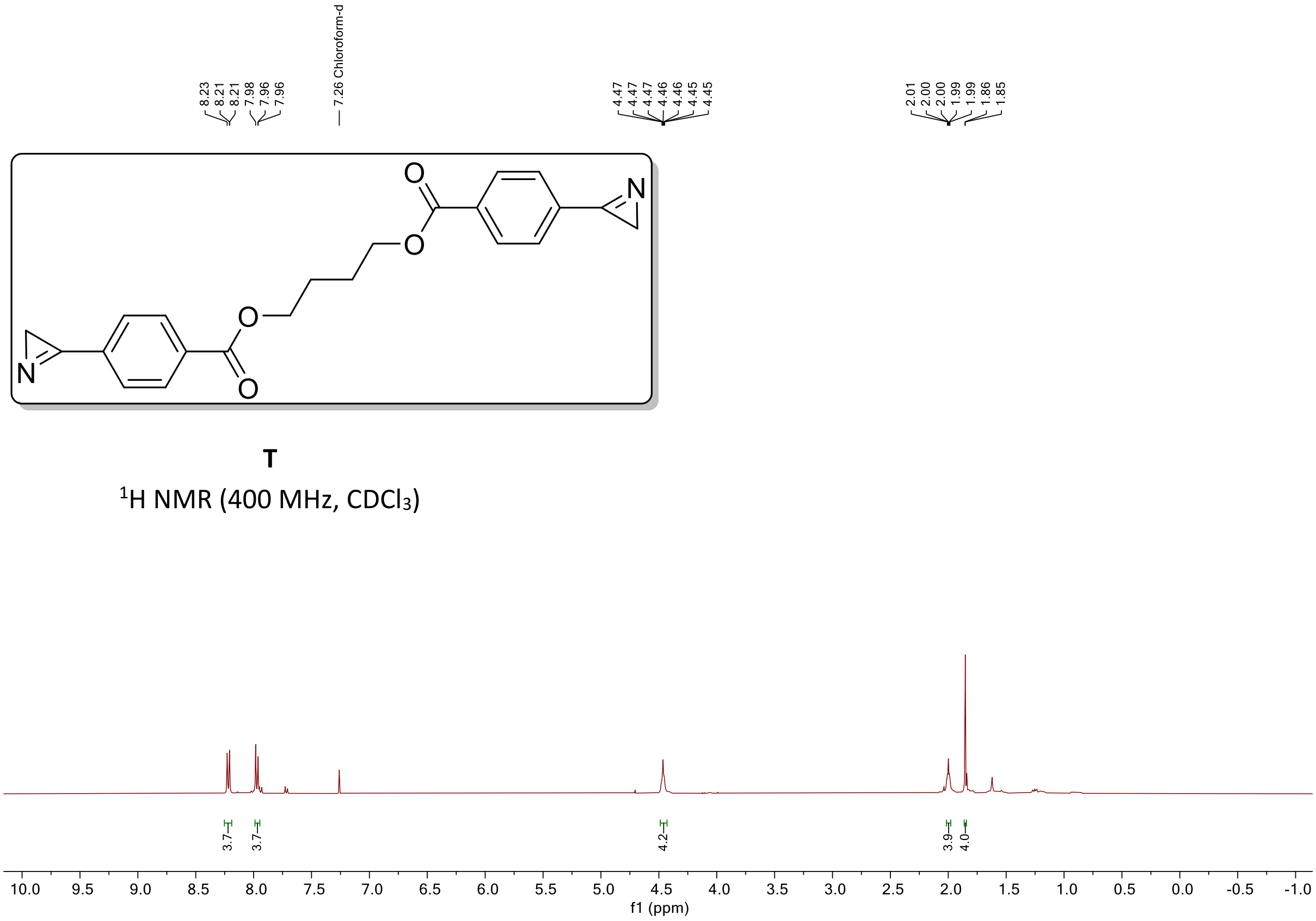


**T**

$^{1}H$ NMR (400 MHz, $CDCl_3$)

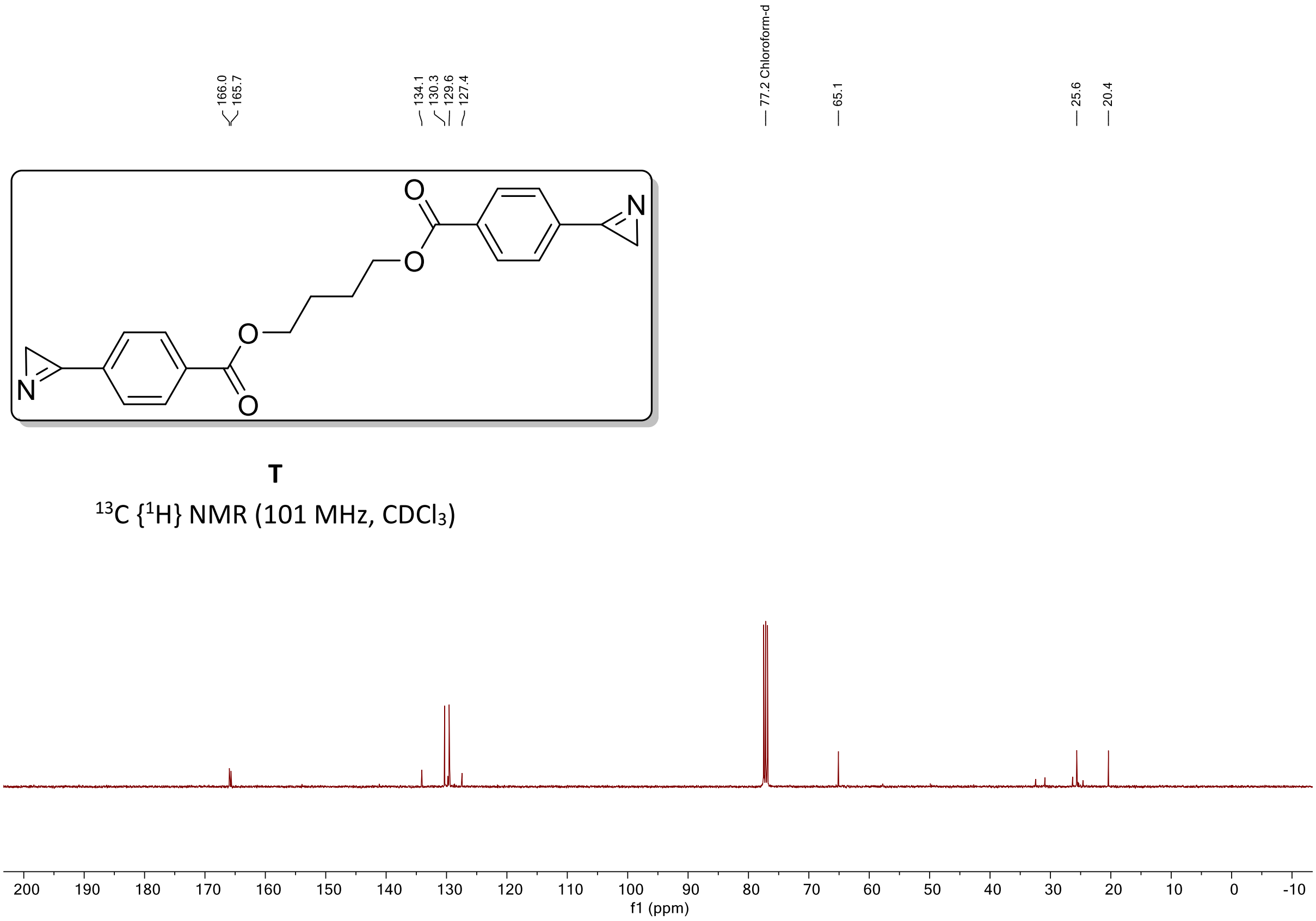


**T**

$^{13}C$ {$^{1}H$} NMR (101 MHz, $CDCl_3$)

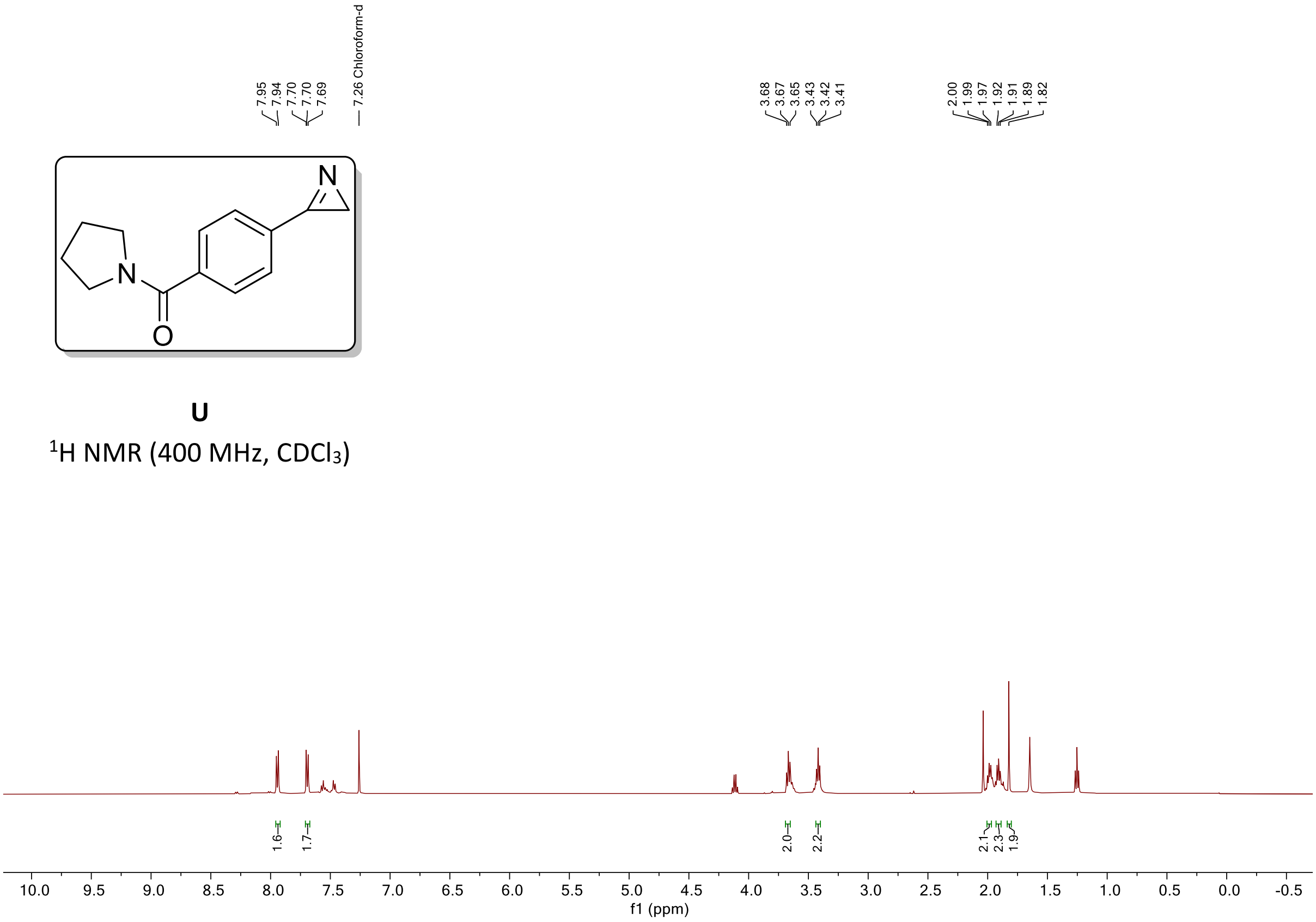


**U**

$^{1}H$ NMR (400 MHz, $CDCl_3$)

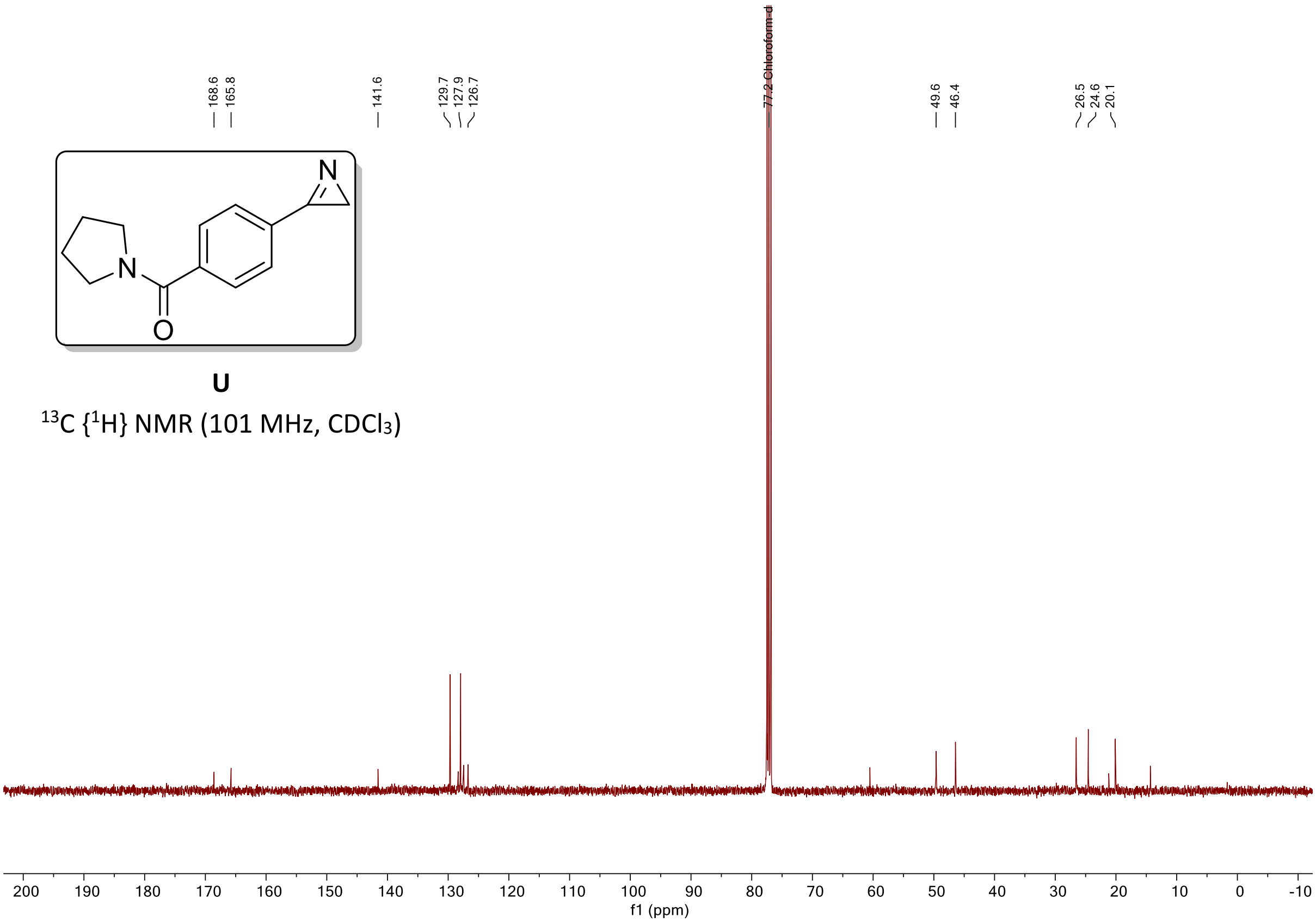


**U**

$^{13}C$ {$^{1}H$} NMR (101 MHz, $CDCl_3$)

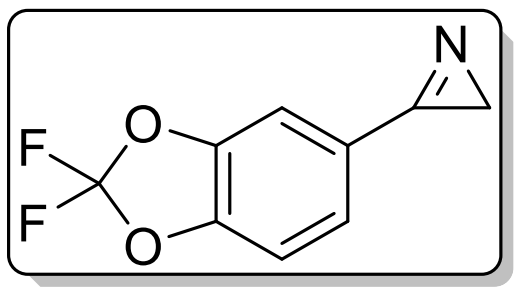


**Y**

$^{1}H$ NMR (400 MHz, $CDCl_3$)

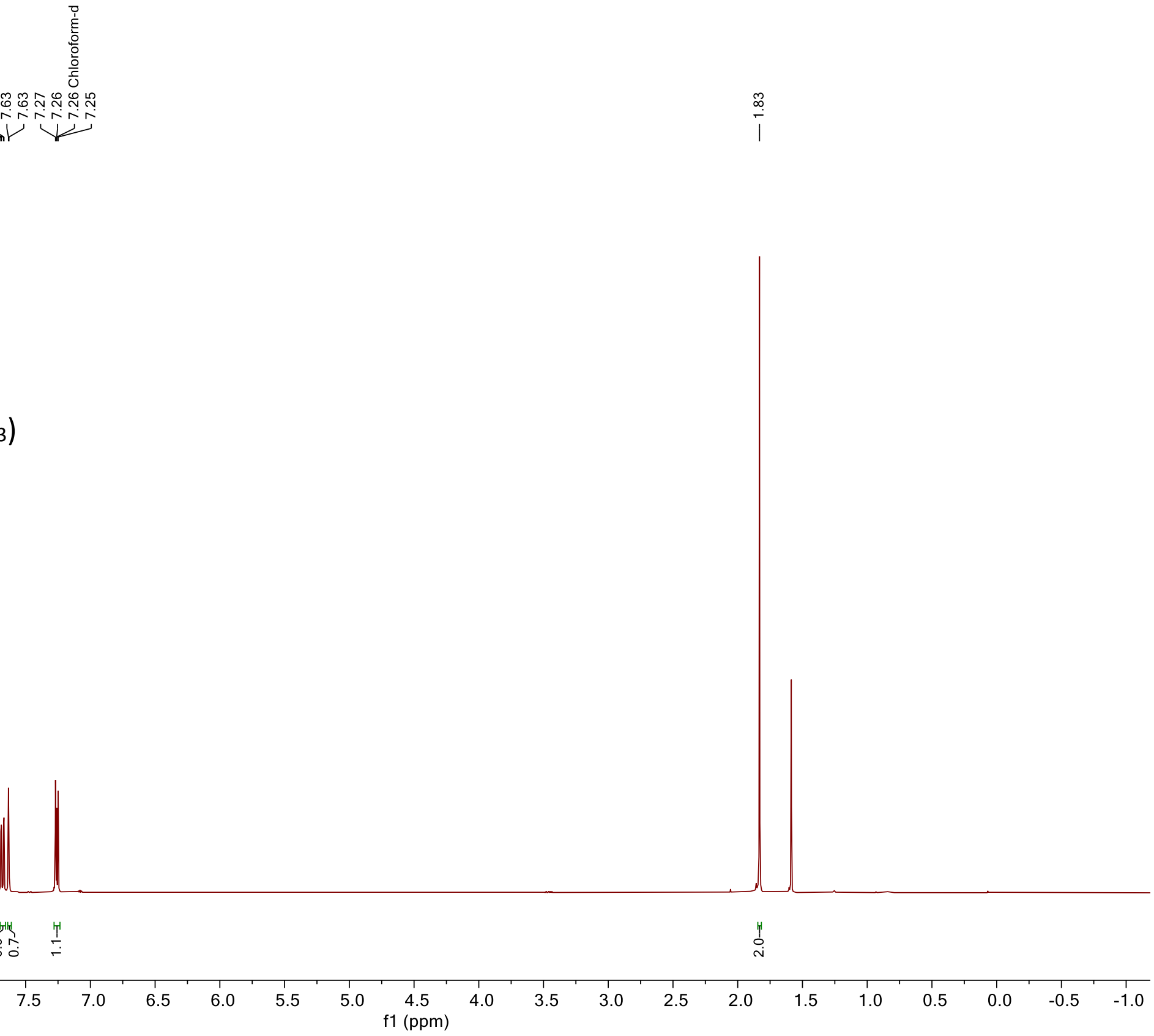


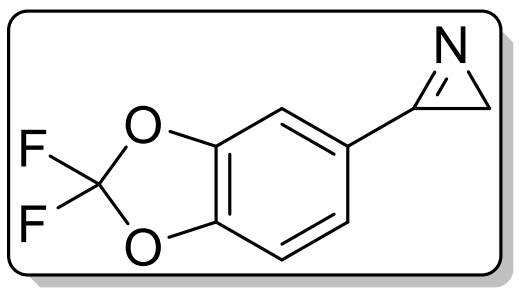


**Y**

$^{19}F$ NMR (377 MHz, $CDCl_3$)

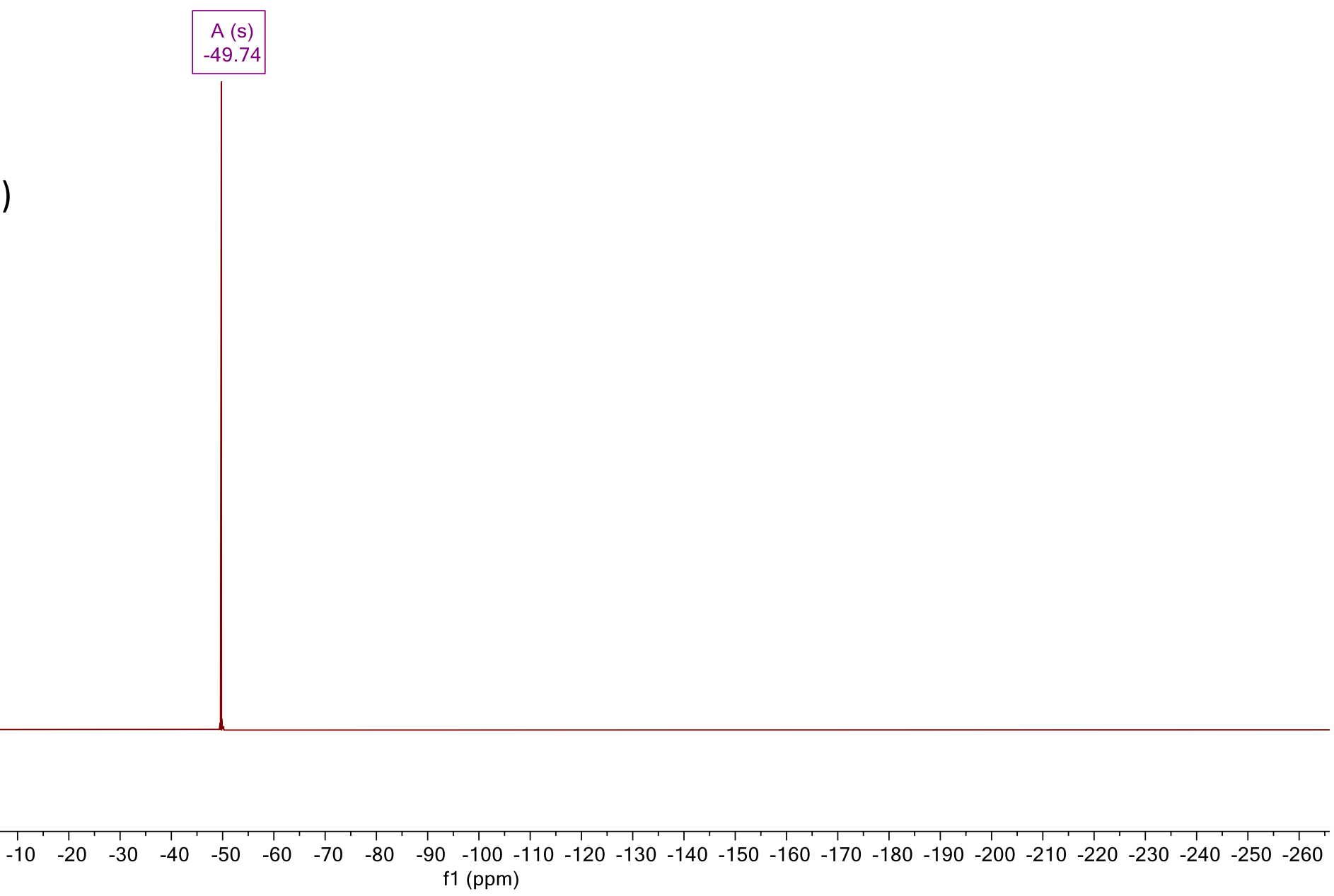

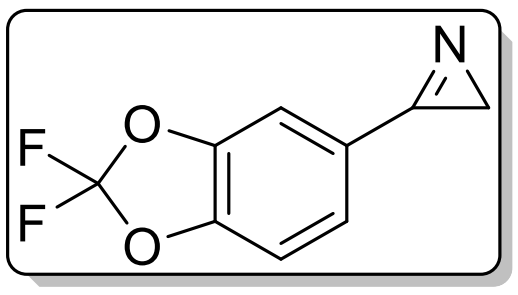


**Y**

$^{13}C\ \{^1H\}$ NMR (101 MHz, $CDCl_3$)

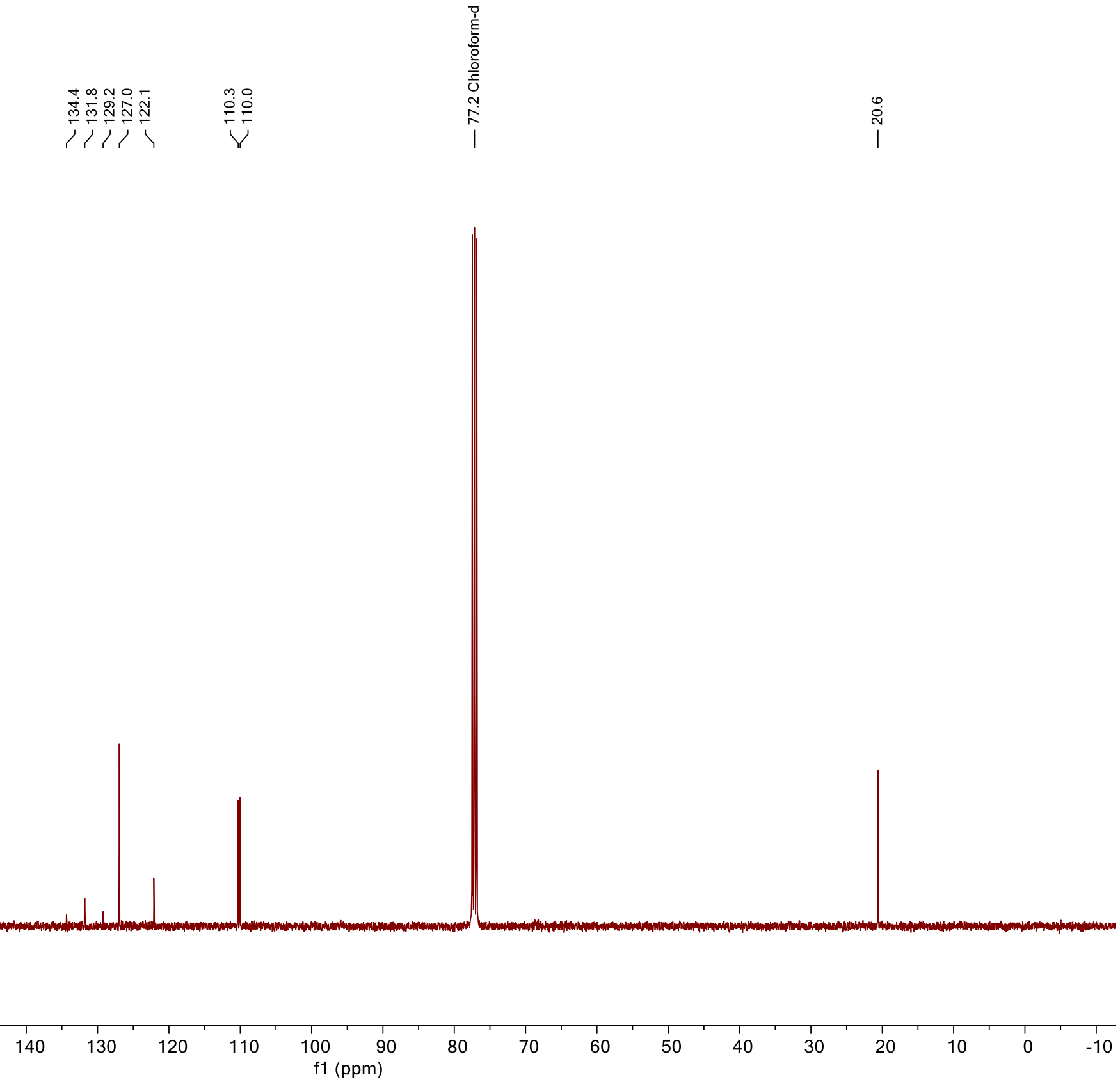

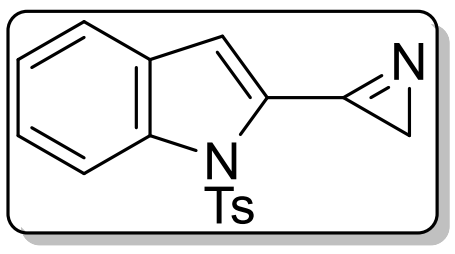


**Z**

$^{1}H$ NMR (400 MHz, $CDCl_3$)

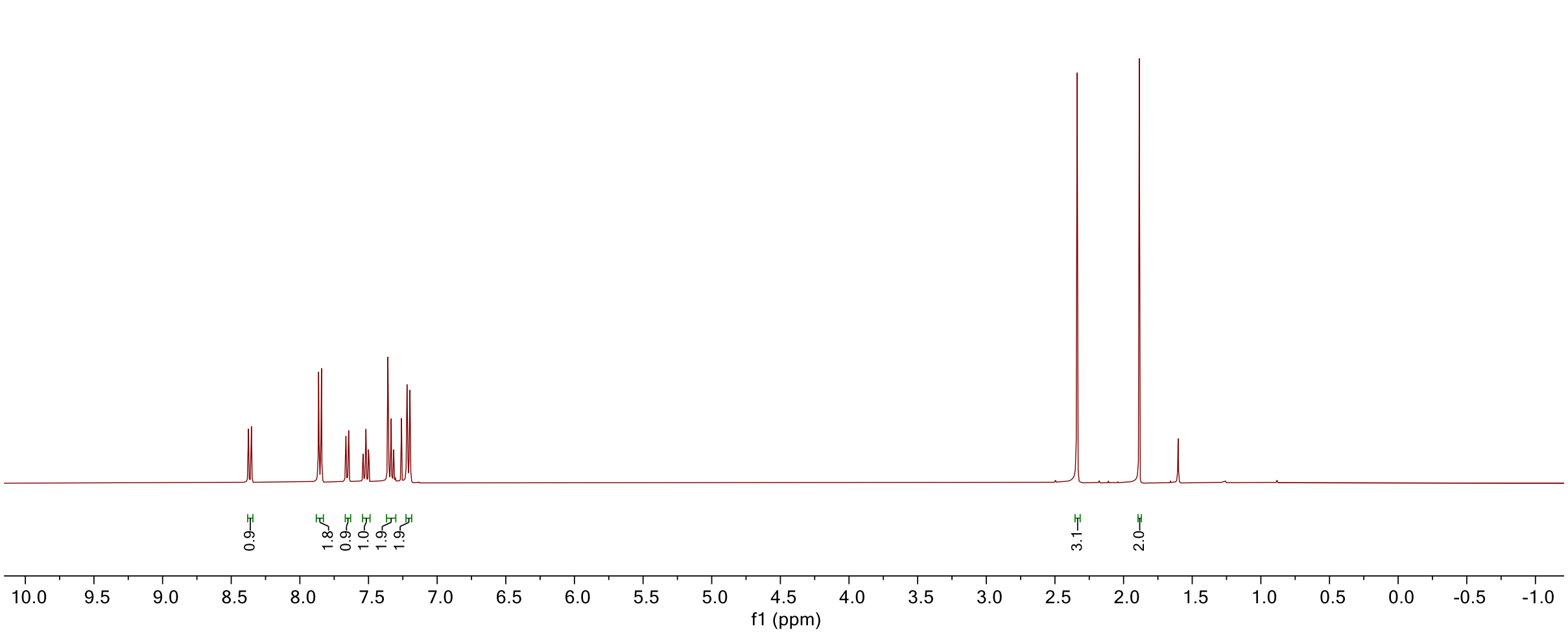


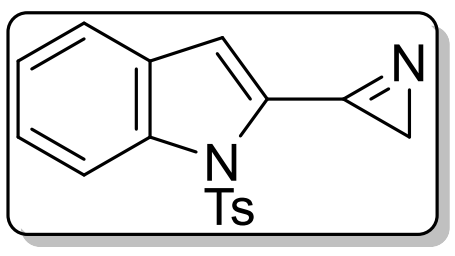


**Z**

$^{13}C$ {$^{1}H$} NMR (101 MHz, $CDCl_3$)

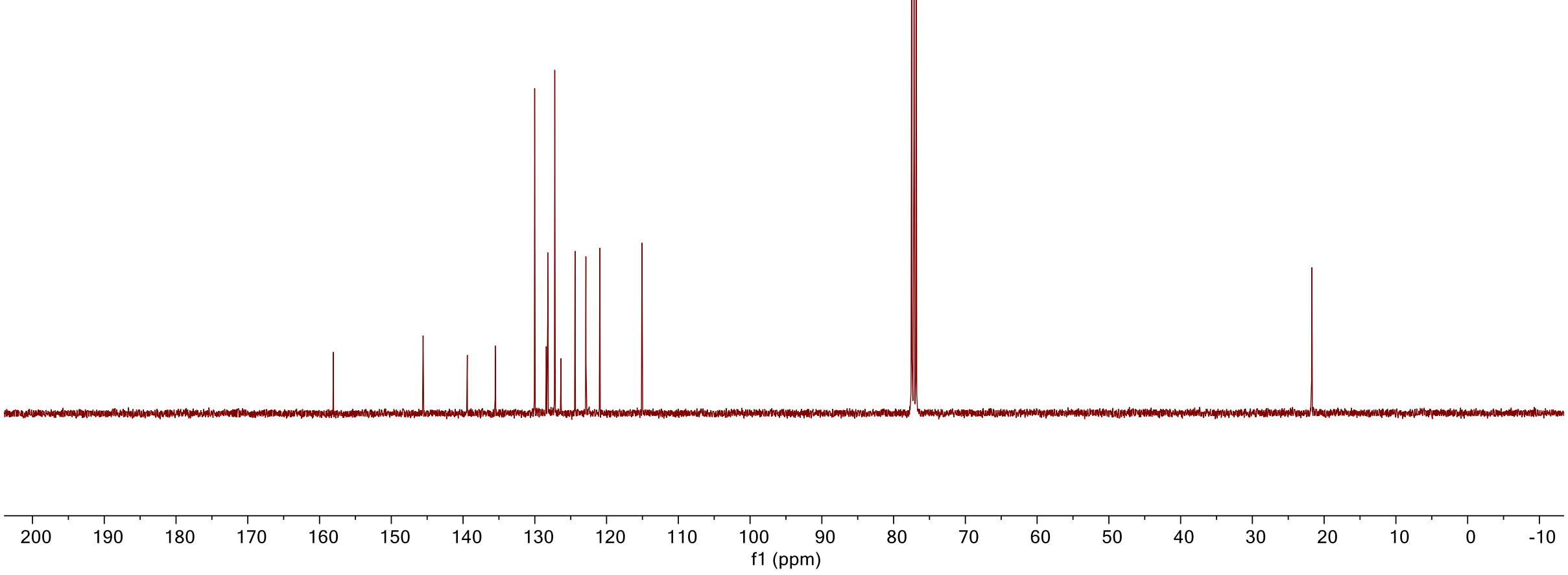

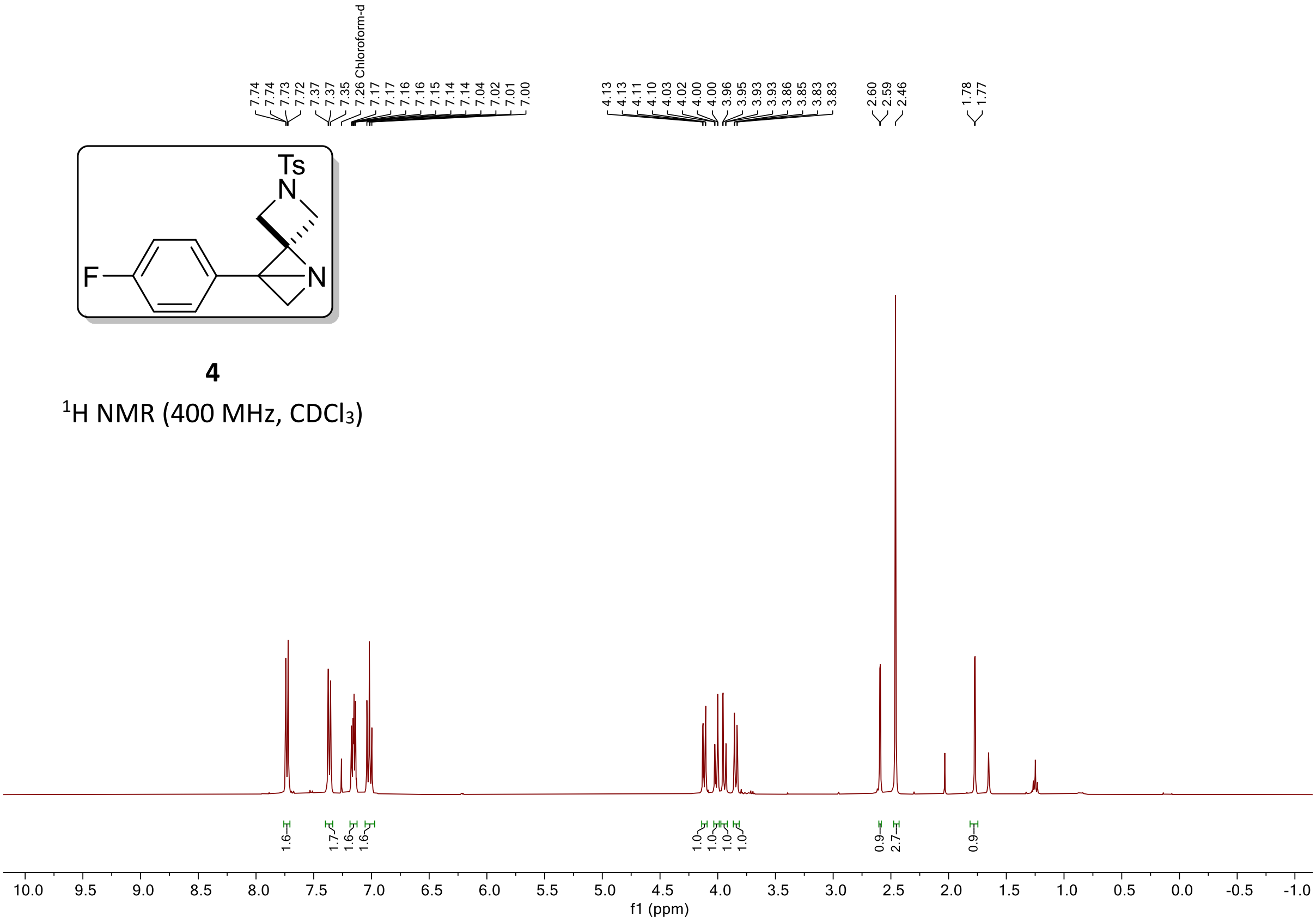

**4**

$^{1}H$ NMR (400 MHz, $CDCl_3$)

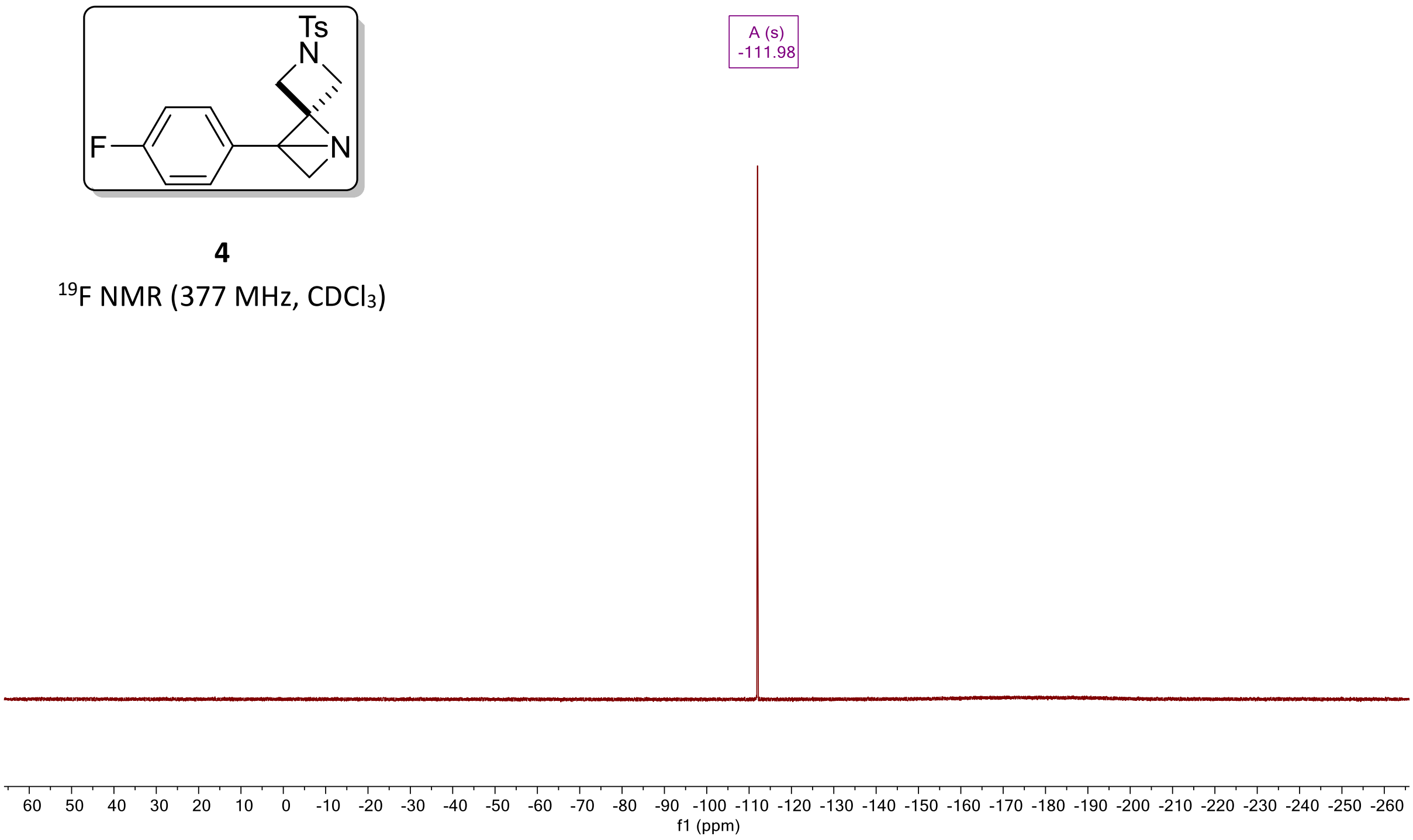

**4**

$^{19}F$ NMR (377 MHz, $CDCl_3$)

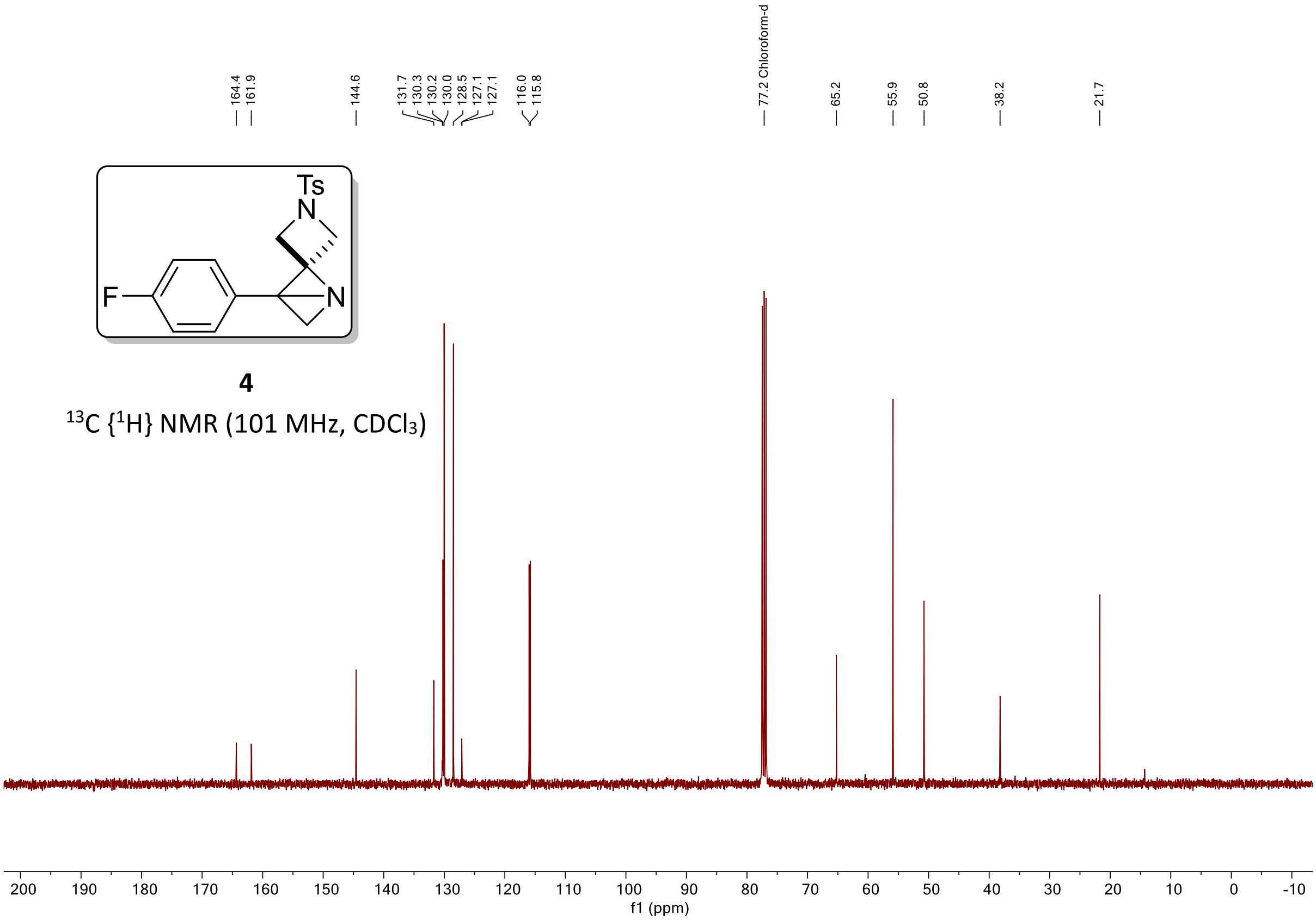

**4**

$^{13}C$ {$^{1}H$} NMR (101 MHz, $CDCl_3$)

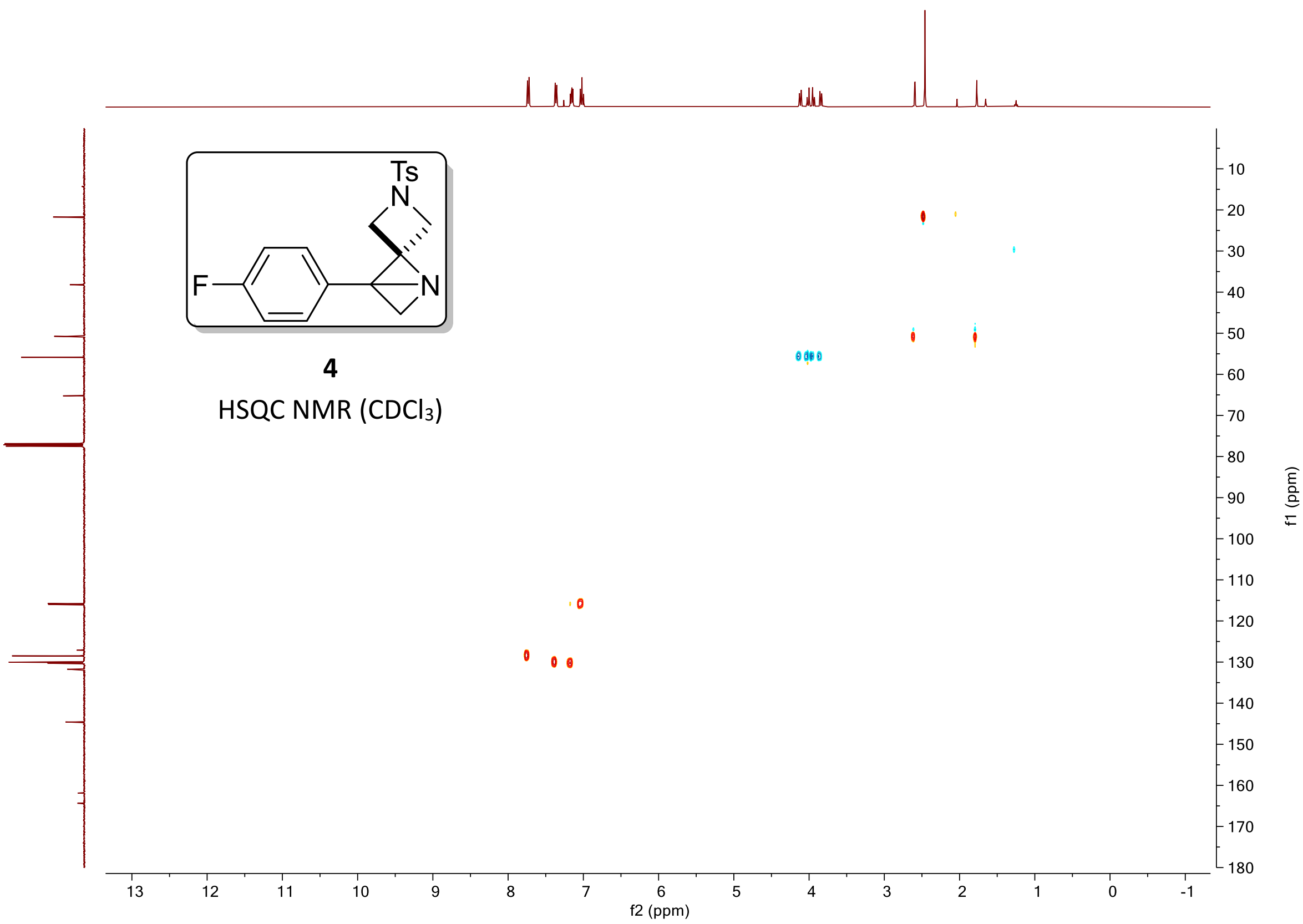

**4**

HSQC NMR ($CDCl_3$)

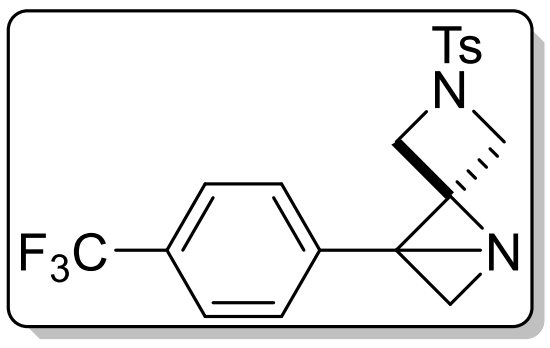


**7**

$^{1}$H NMR (400 MHz, CDCl$_3$)

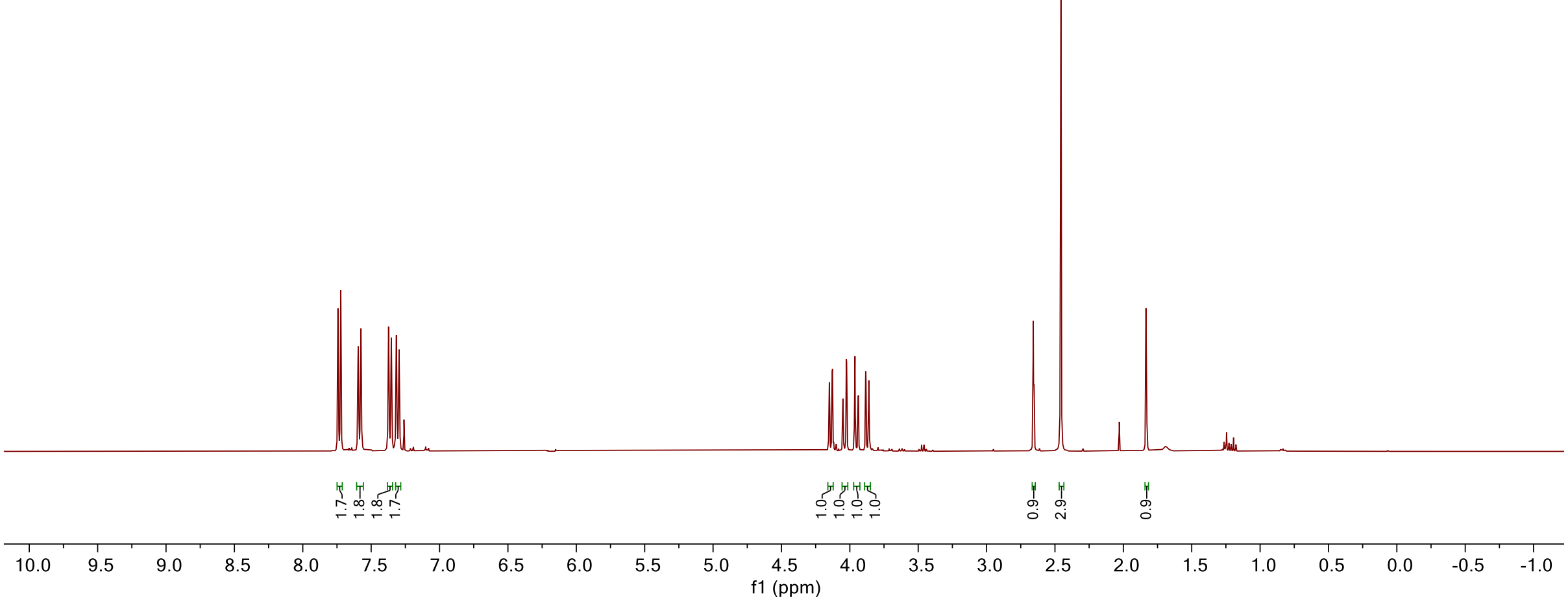


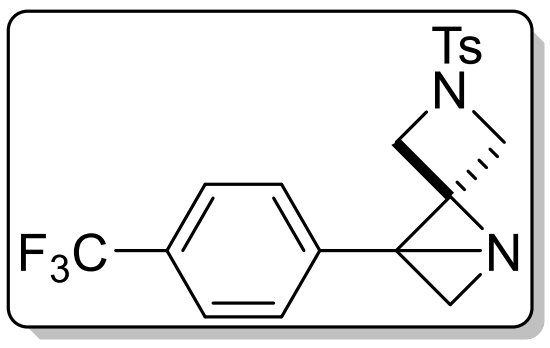


**7**

$^{19}$F NMR (377 MHz, CDCl$_3$)

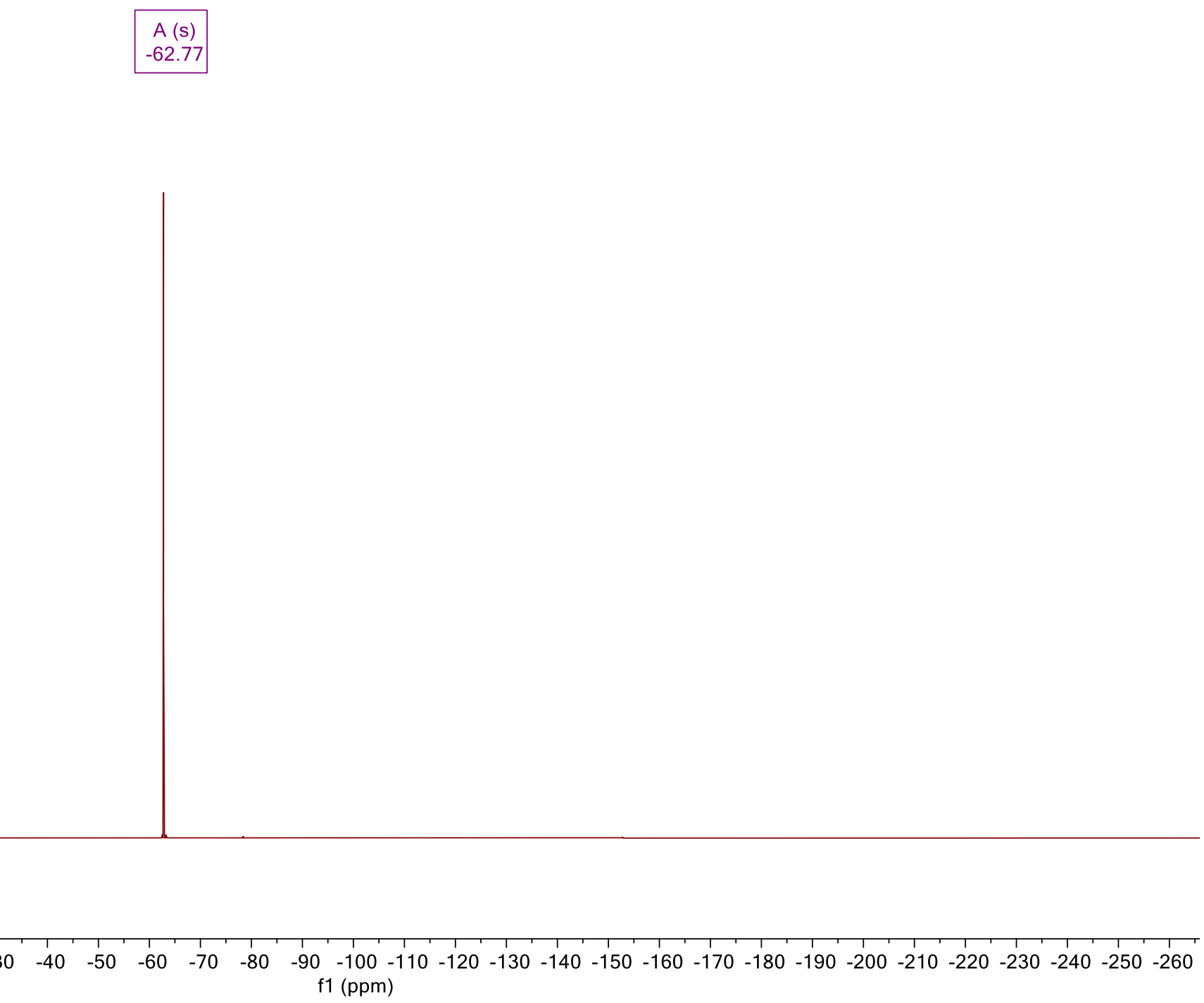

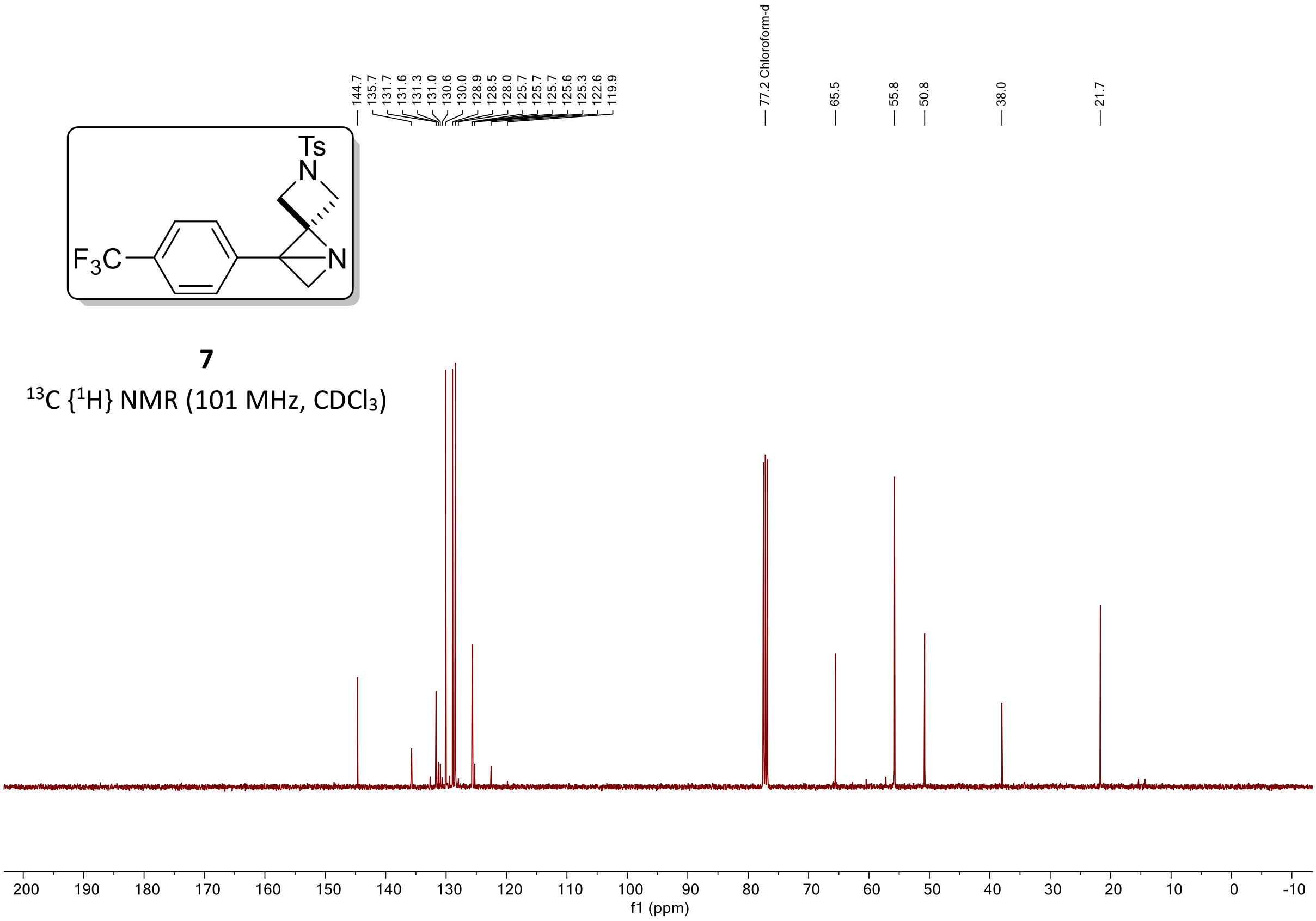


**7**

$^{13}C\{^{1}H\}$ NMR (101 MHz, $CDCl_3$)

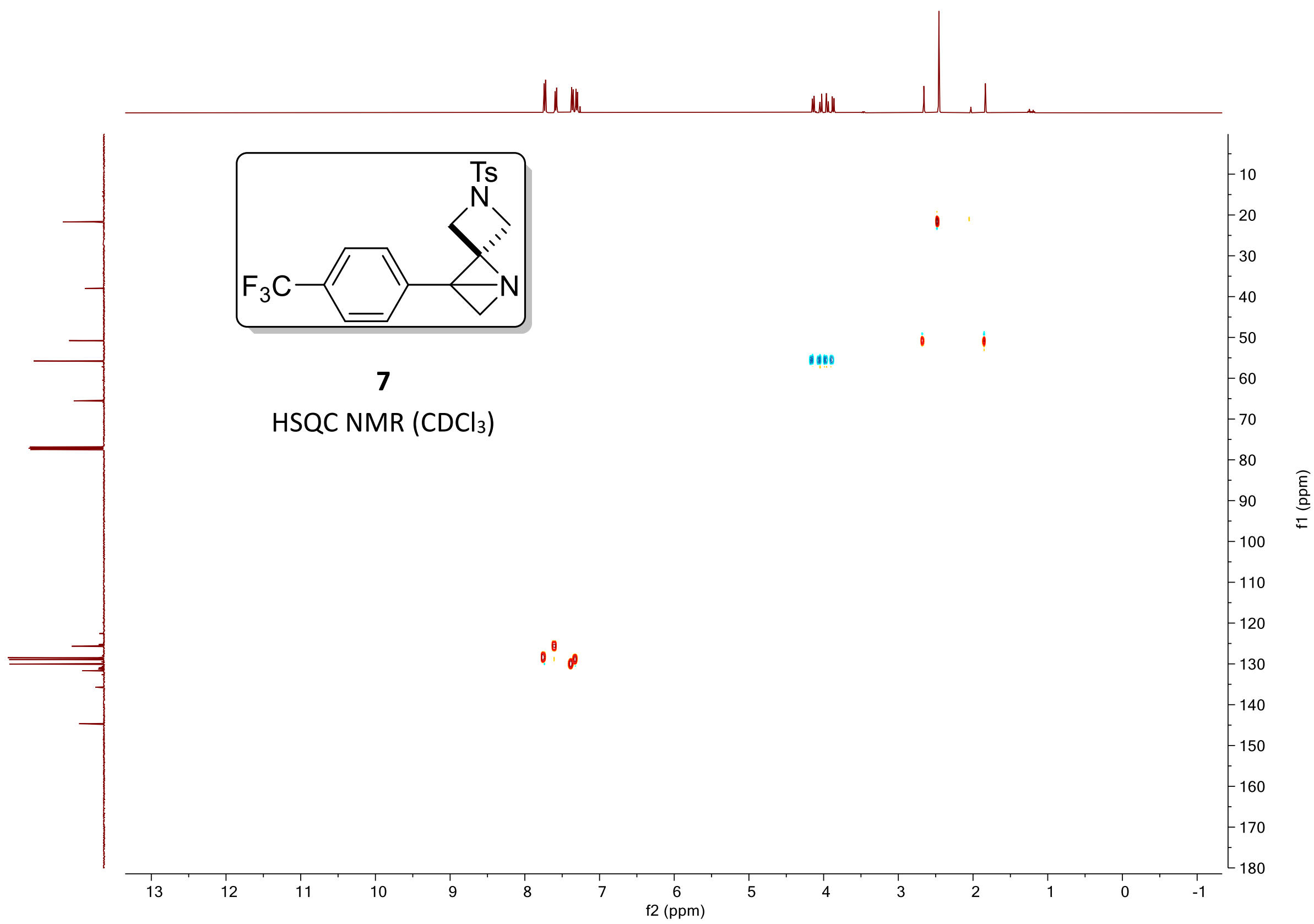


**7**

HSQC NMR ($CDCl_3$)

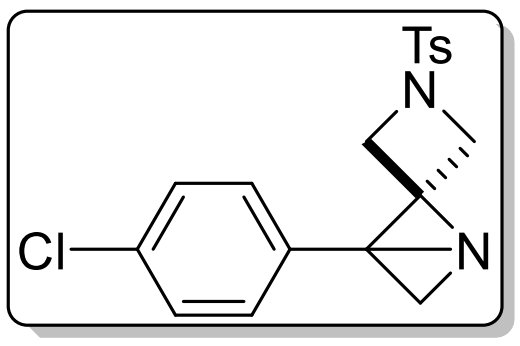


**8**

$^1H$ NMR (400 MHz, $CDCl_3$)

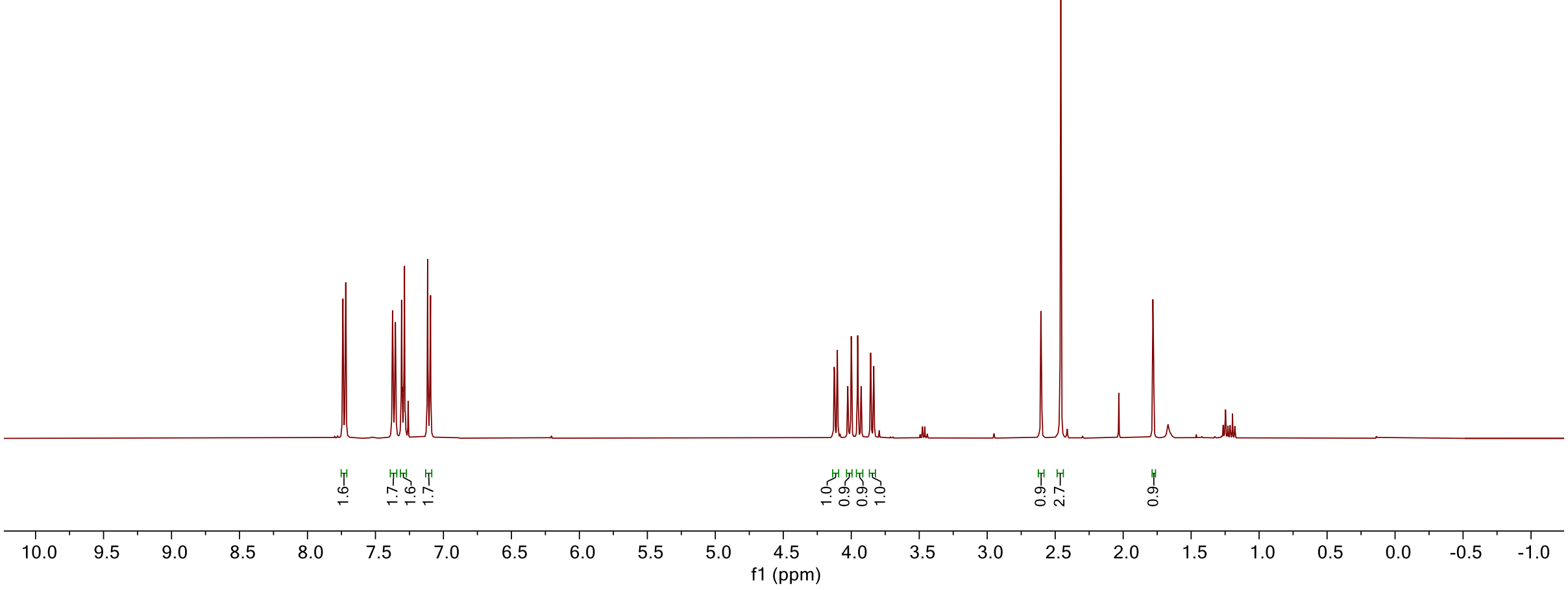


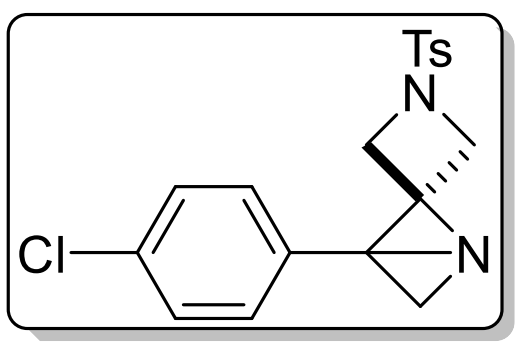


**8**

$^{13}C$ {$^1H$} NMR (101 MHz, $CDCl_3$)

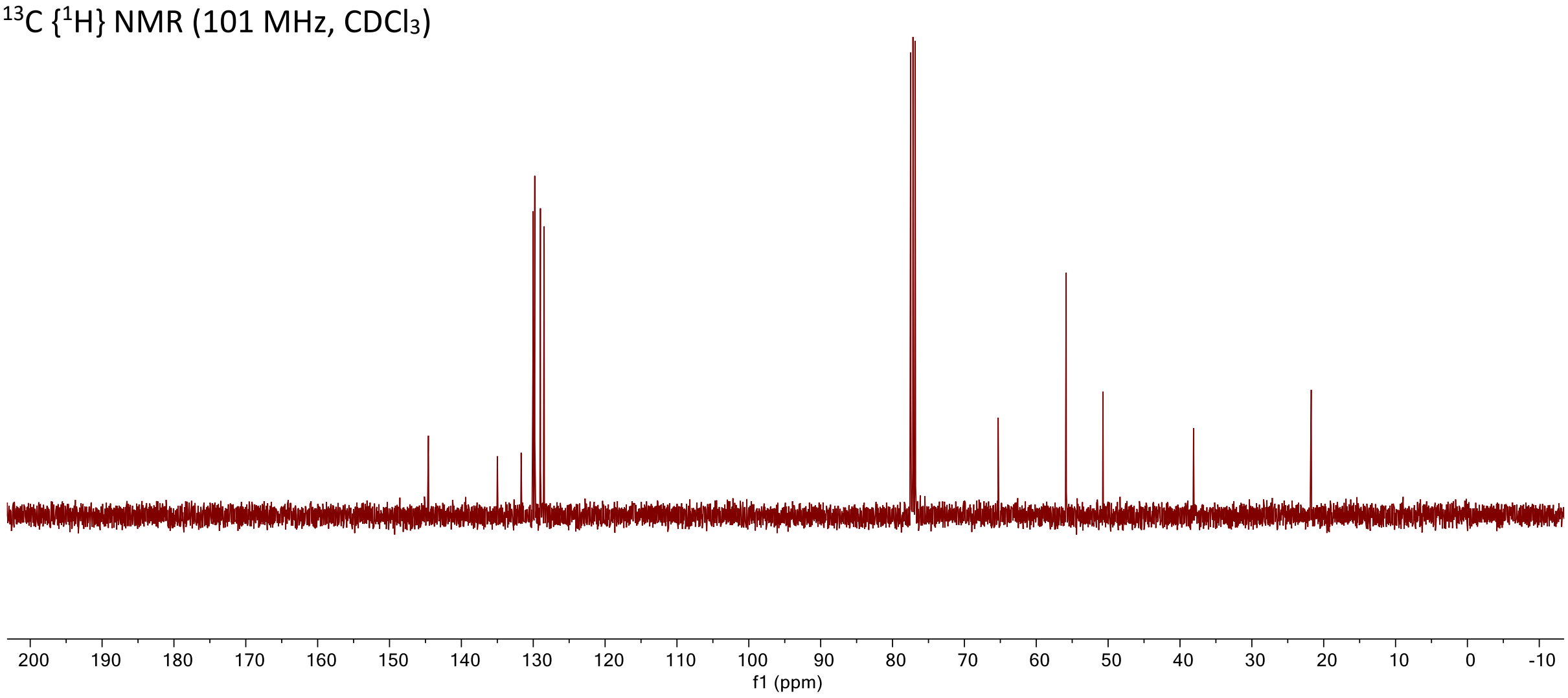

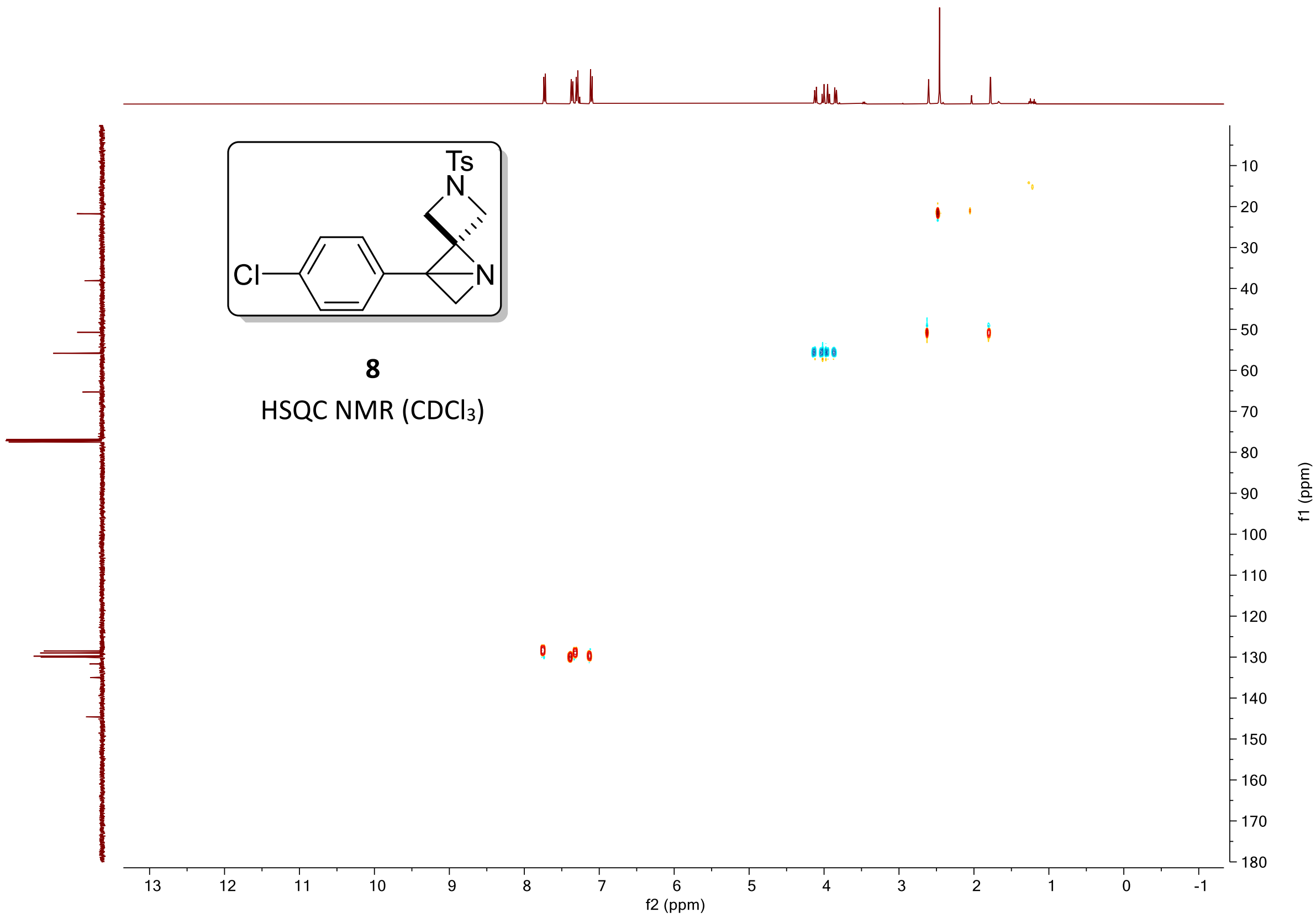
Ts
N
Cl
N
8
HSQC NMR ($CDCl_3$)
f2 (ppm)
f1 (ppm)

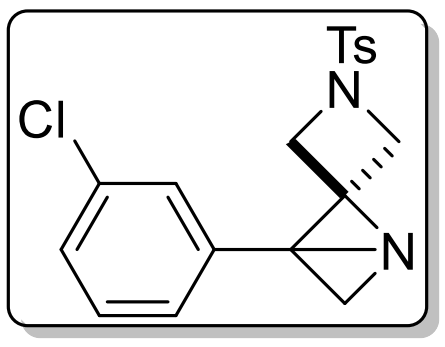

**9**

$^{1}$H NMR (400 MHz, $CDCl_3$)

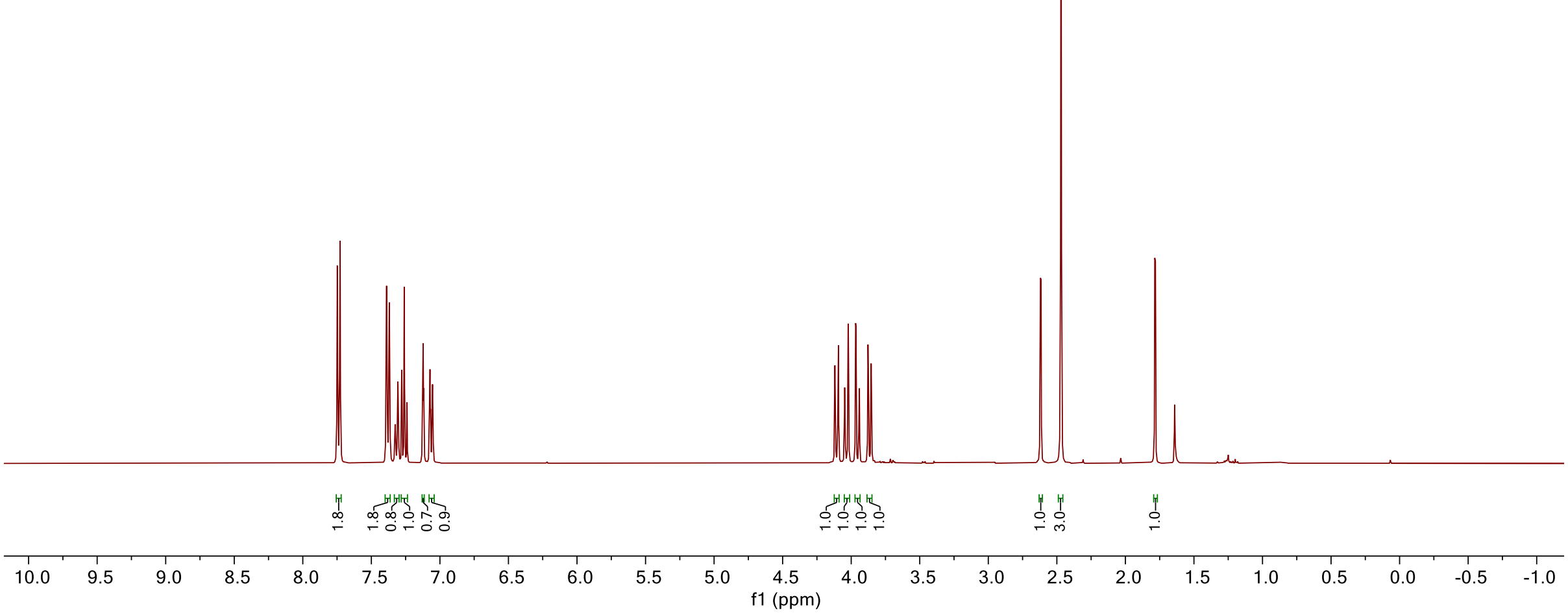


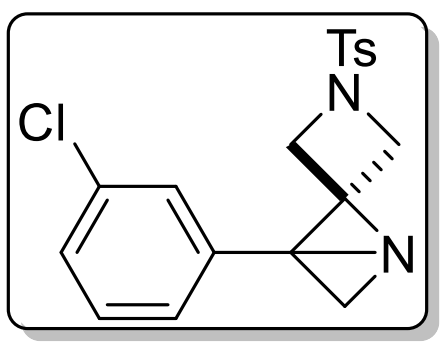

**9**

$^{13}$C {$^{1}$H} NMR (101 MHz, $CDCl_3$)

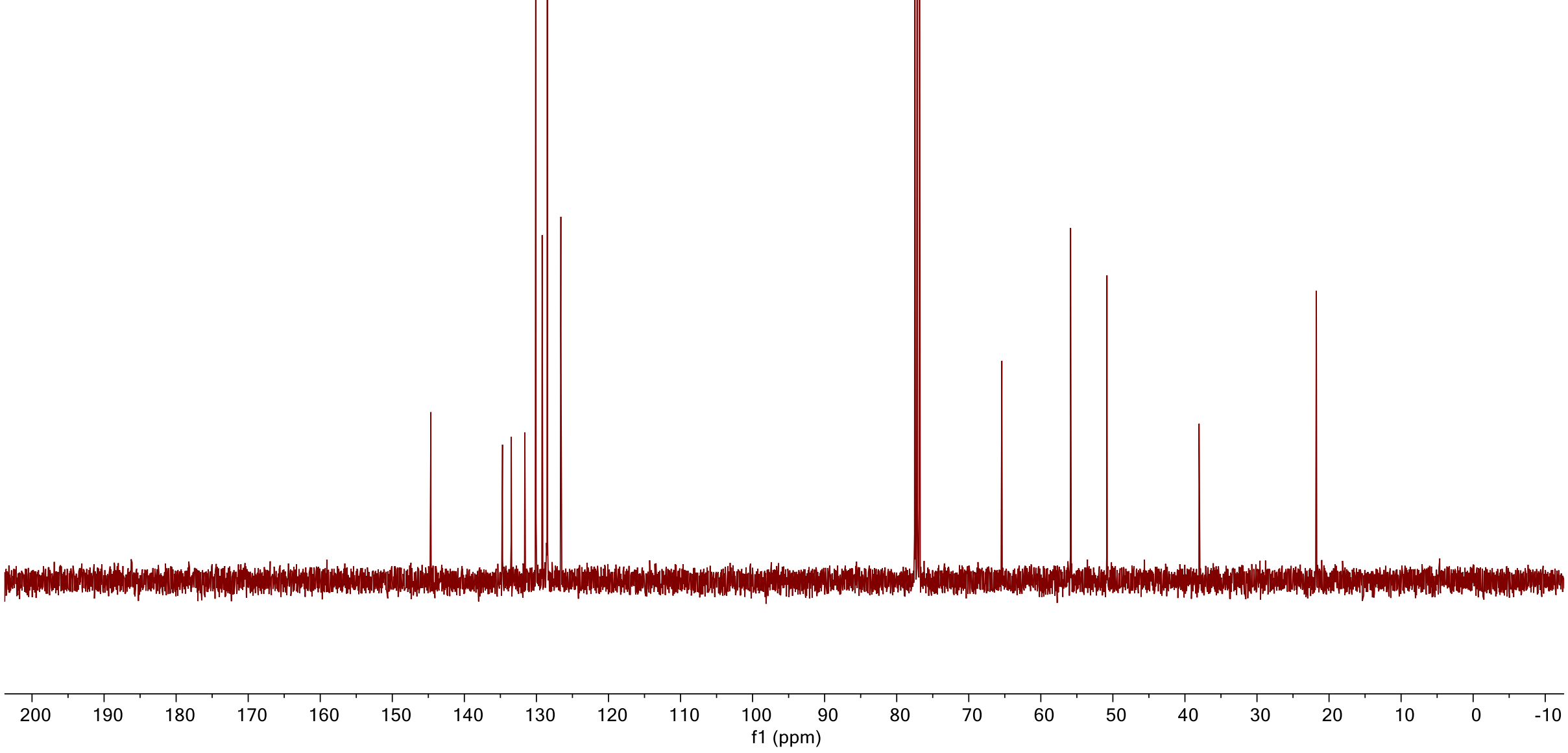

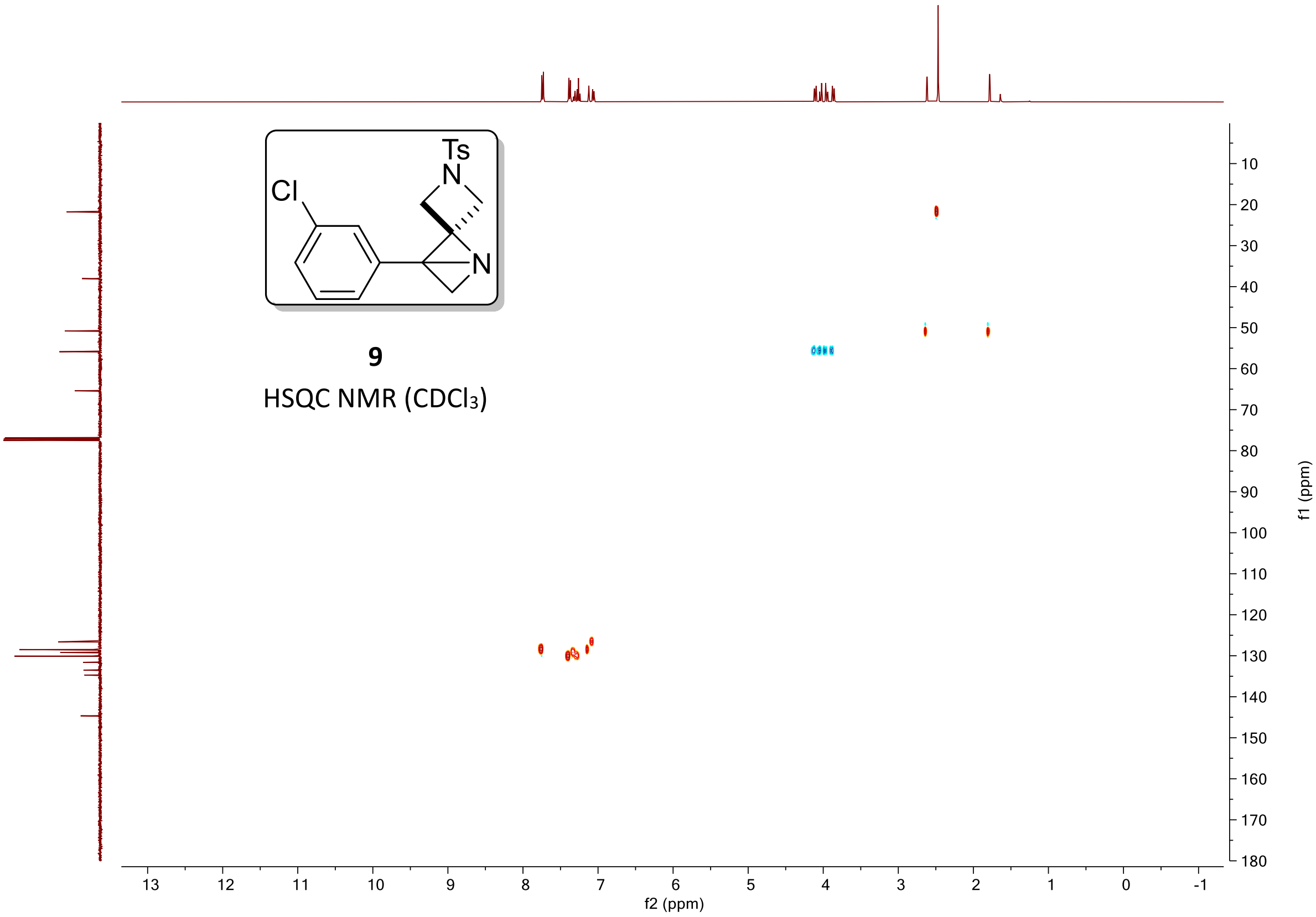


**9**

HSQC NMR ($CDCl_3$)

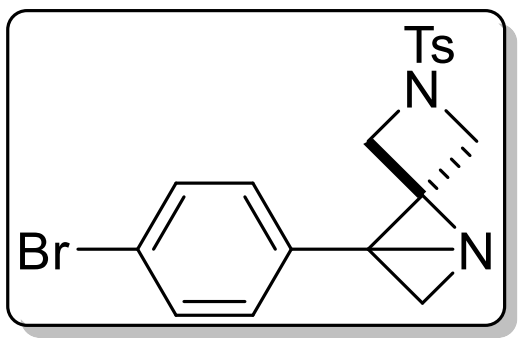


**10**

$^{1}$H NMR (400 MHz, $CDCl_3$)

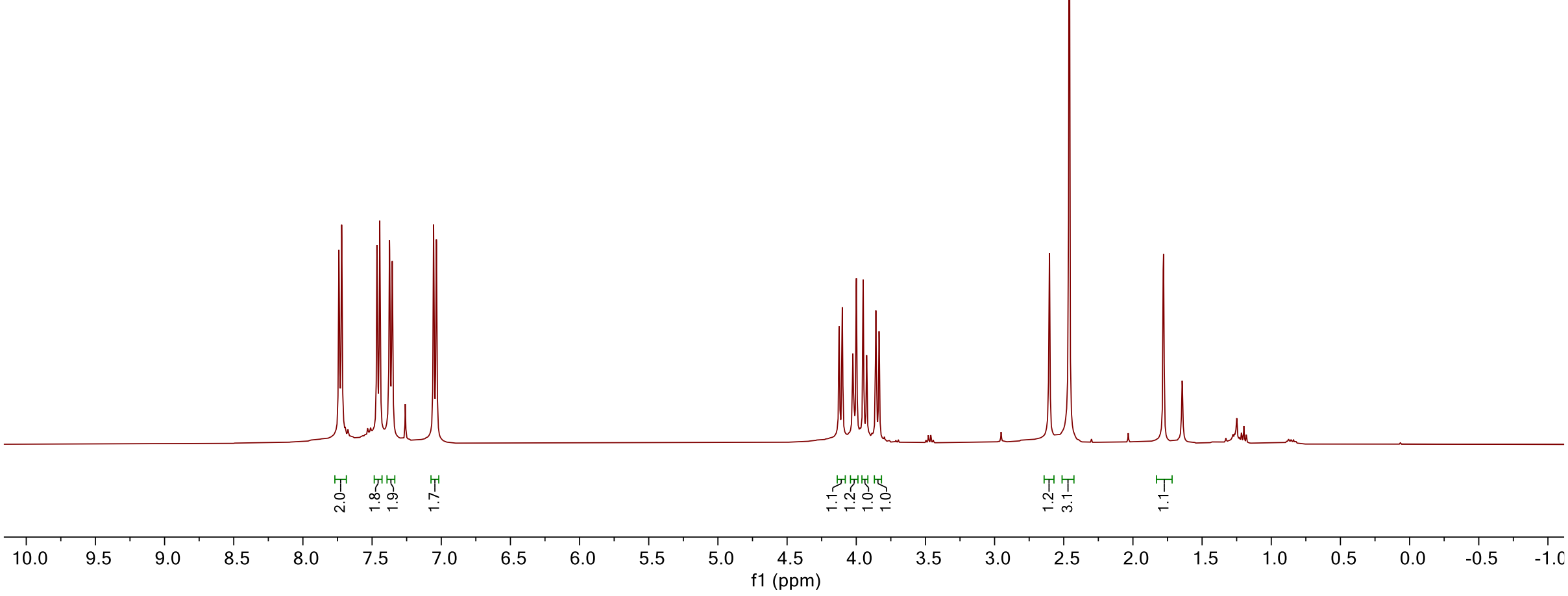


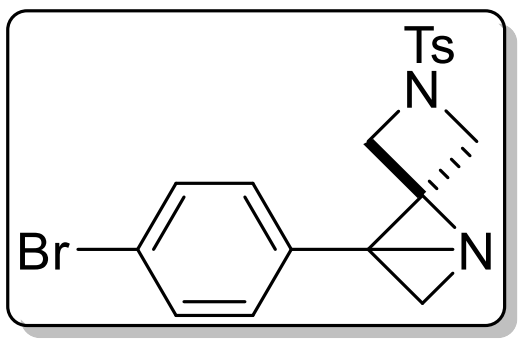


**10**

$^{13}$C {$^{1}$H} NMR (101 MHz, $CDCl_3$)

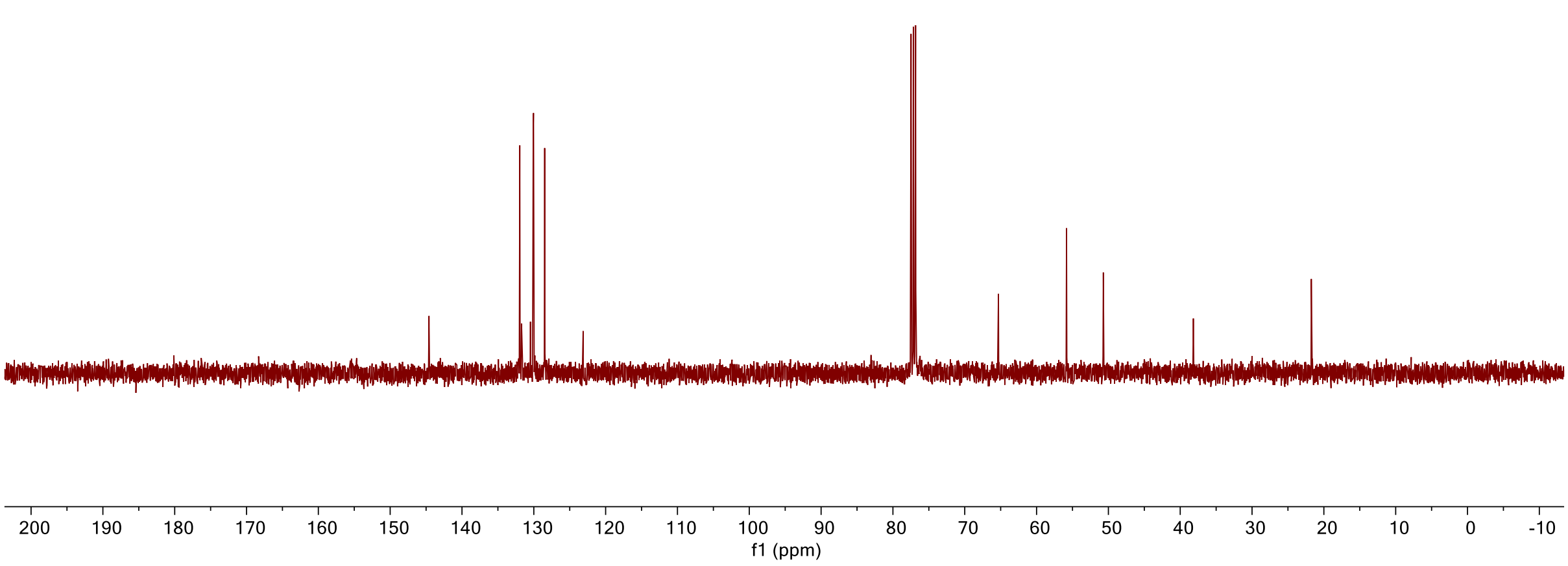

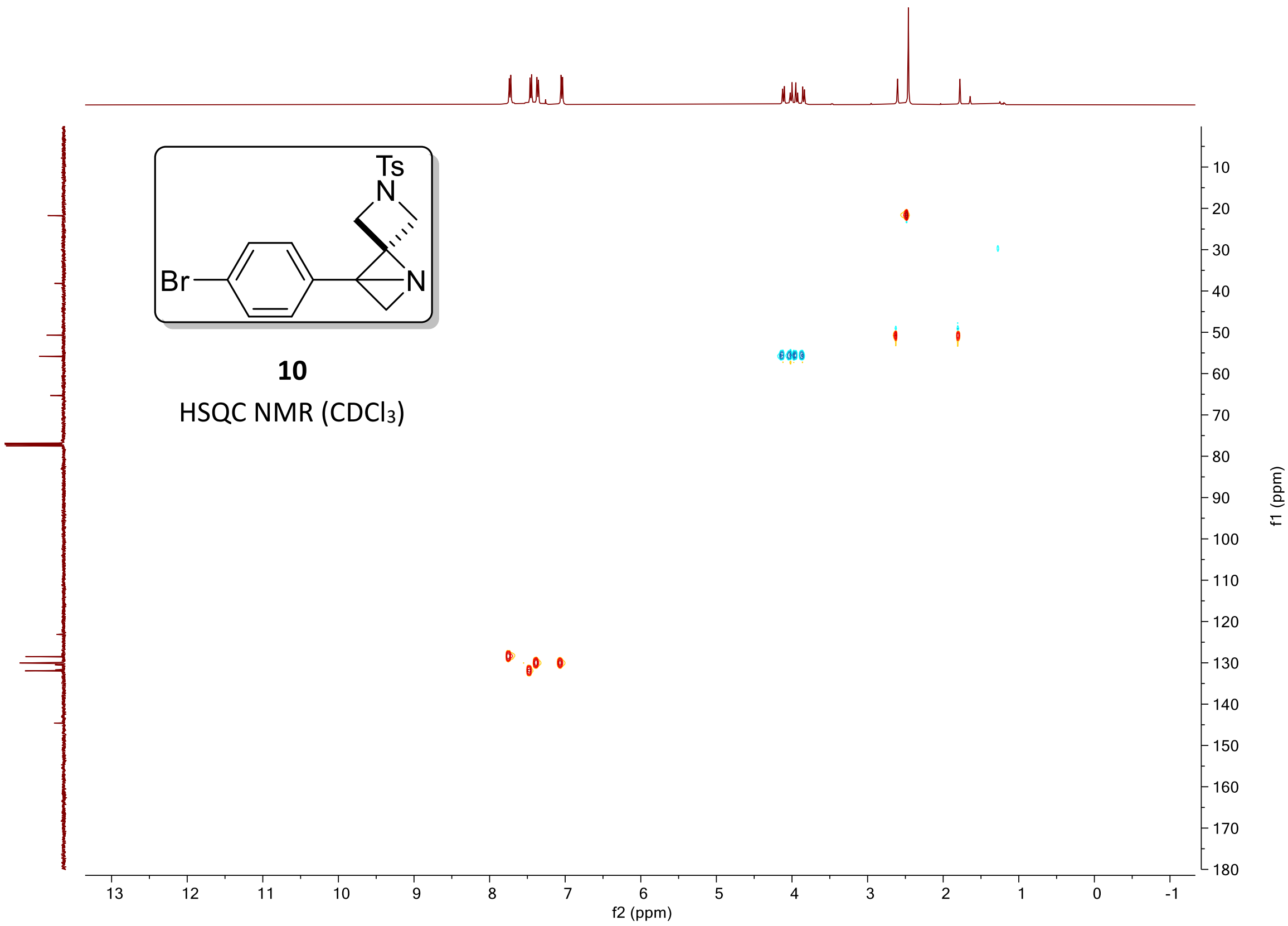
Ts
N
Br
N
10
HSQC NMR (CDCl3)
f1 (ppm)
f2 (ppm)
13
12
11
10
9
8
7
6
5
4
3
2
1
0
-1
10
20
30
40
50
60
70
80
90
100
110
120
130
140
150
160
170
180

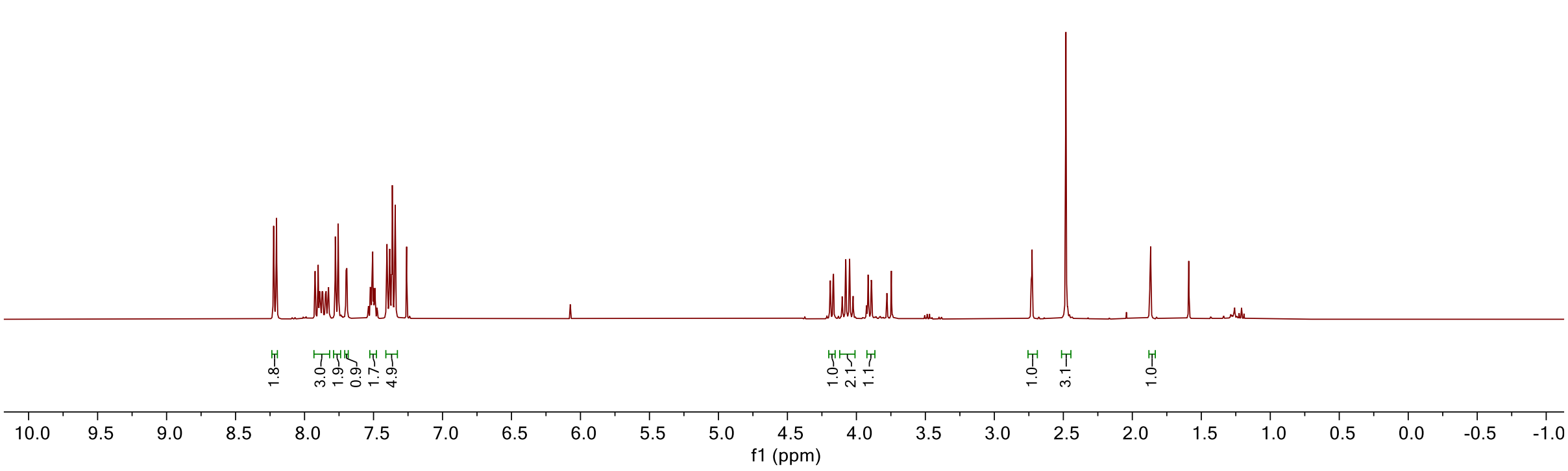


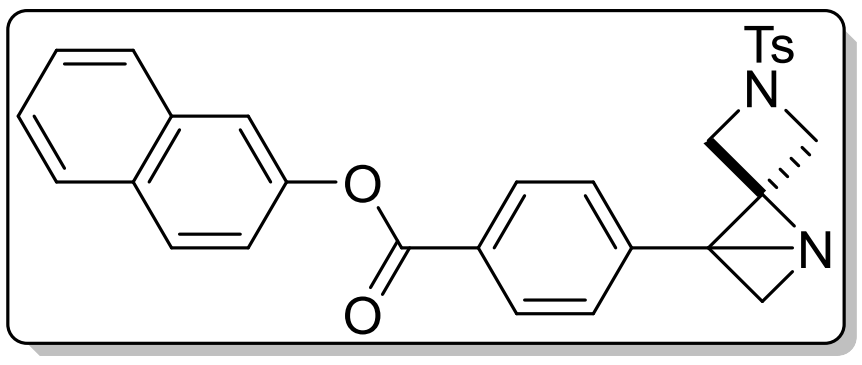


**11**

$^{1}H$ NMR (400 MHz, $CDCl_3$)

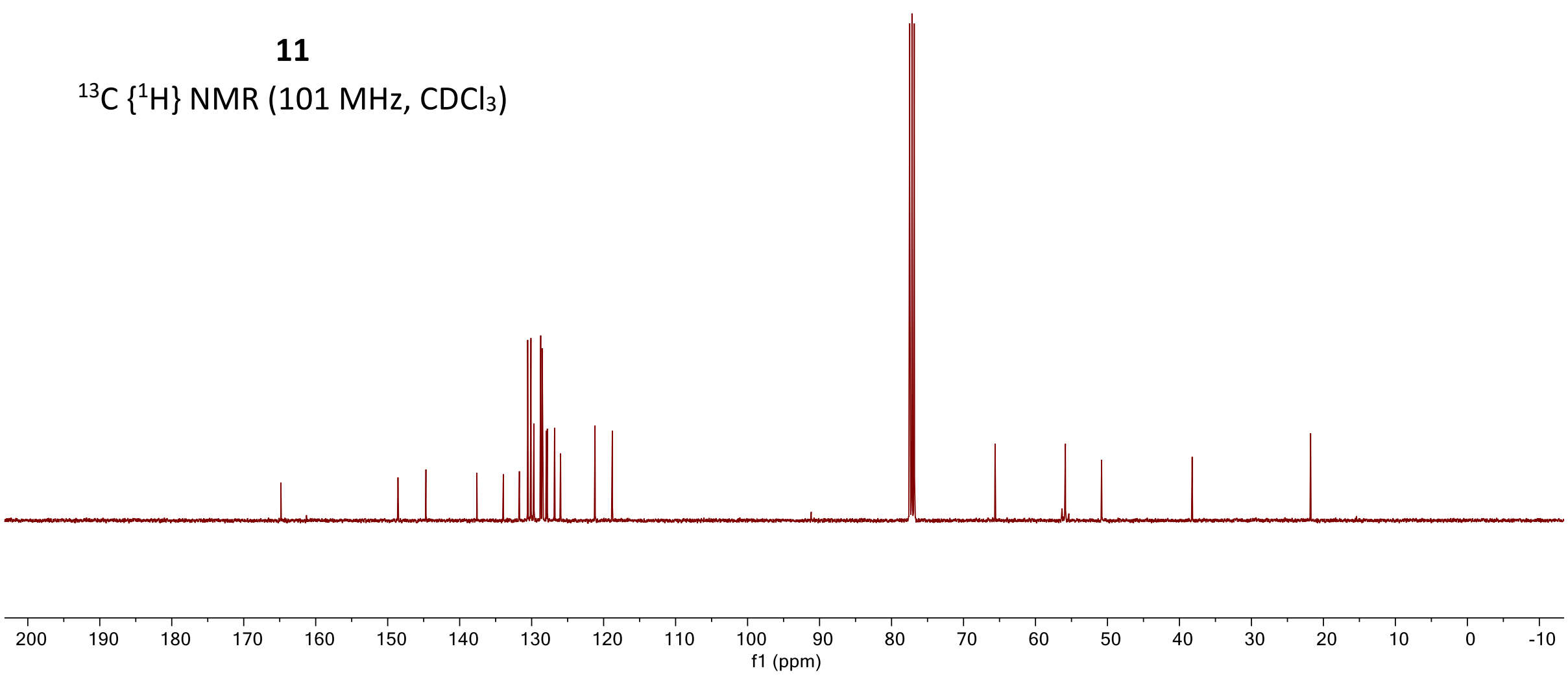


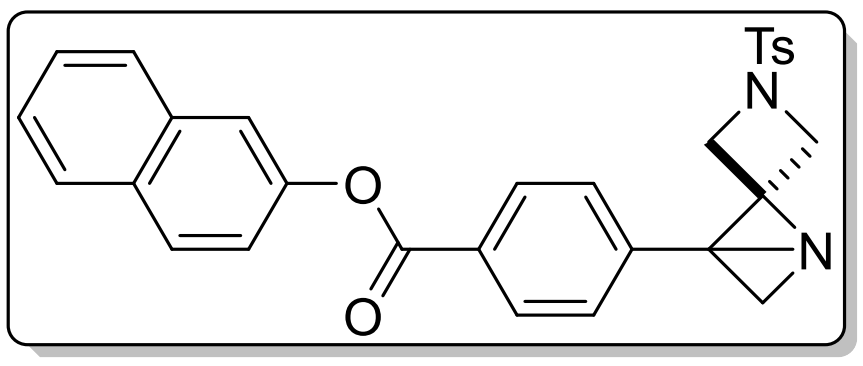


**11**

$^{13}C$ {$^{1}H$} NMR (101 MHz, $CDCl_3$)

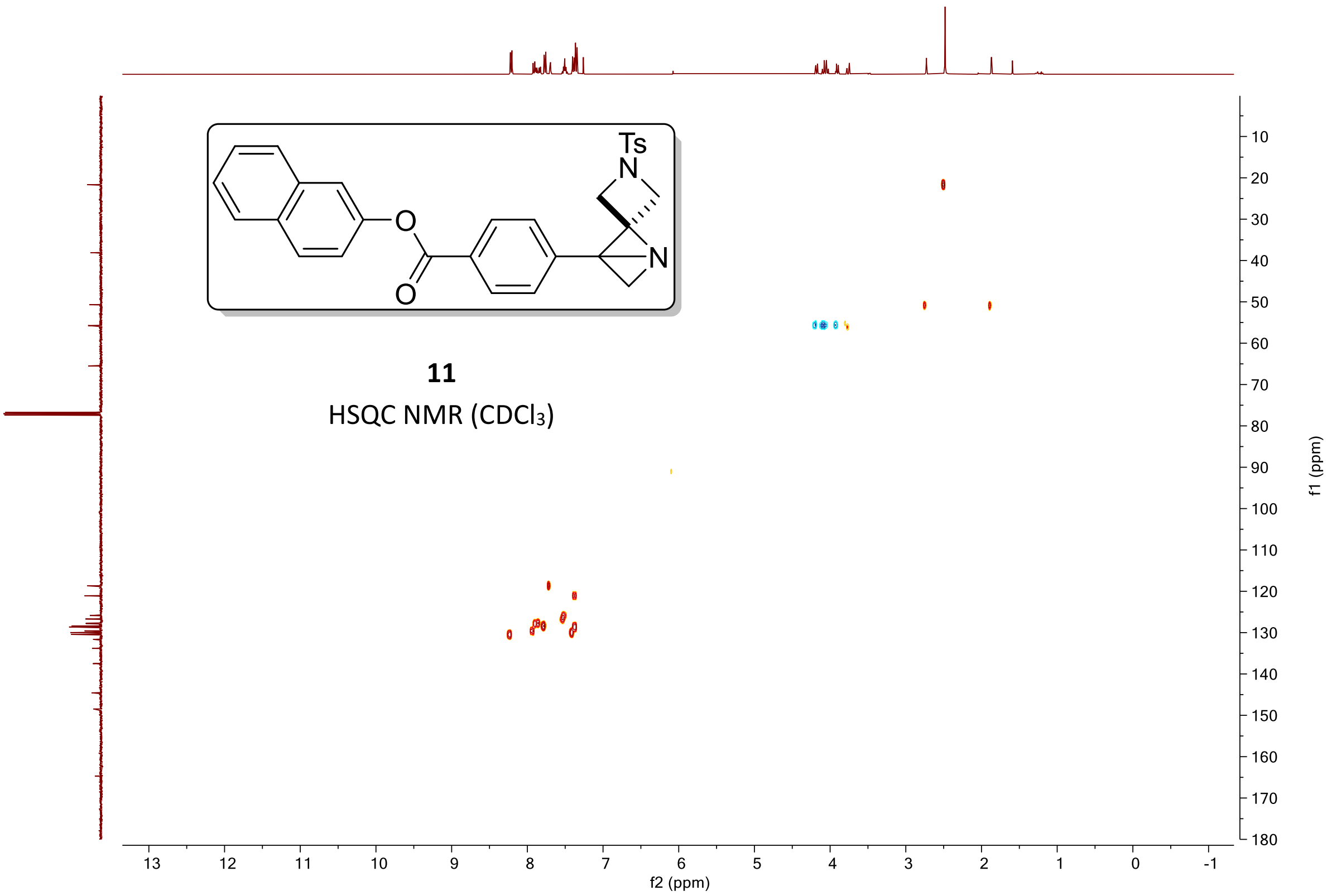


**11**
HSQC NMR ($CDCl_3$)

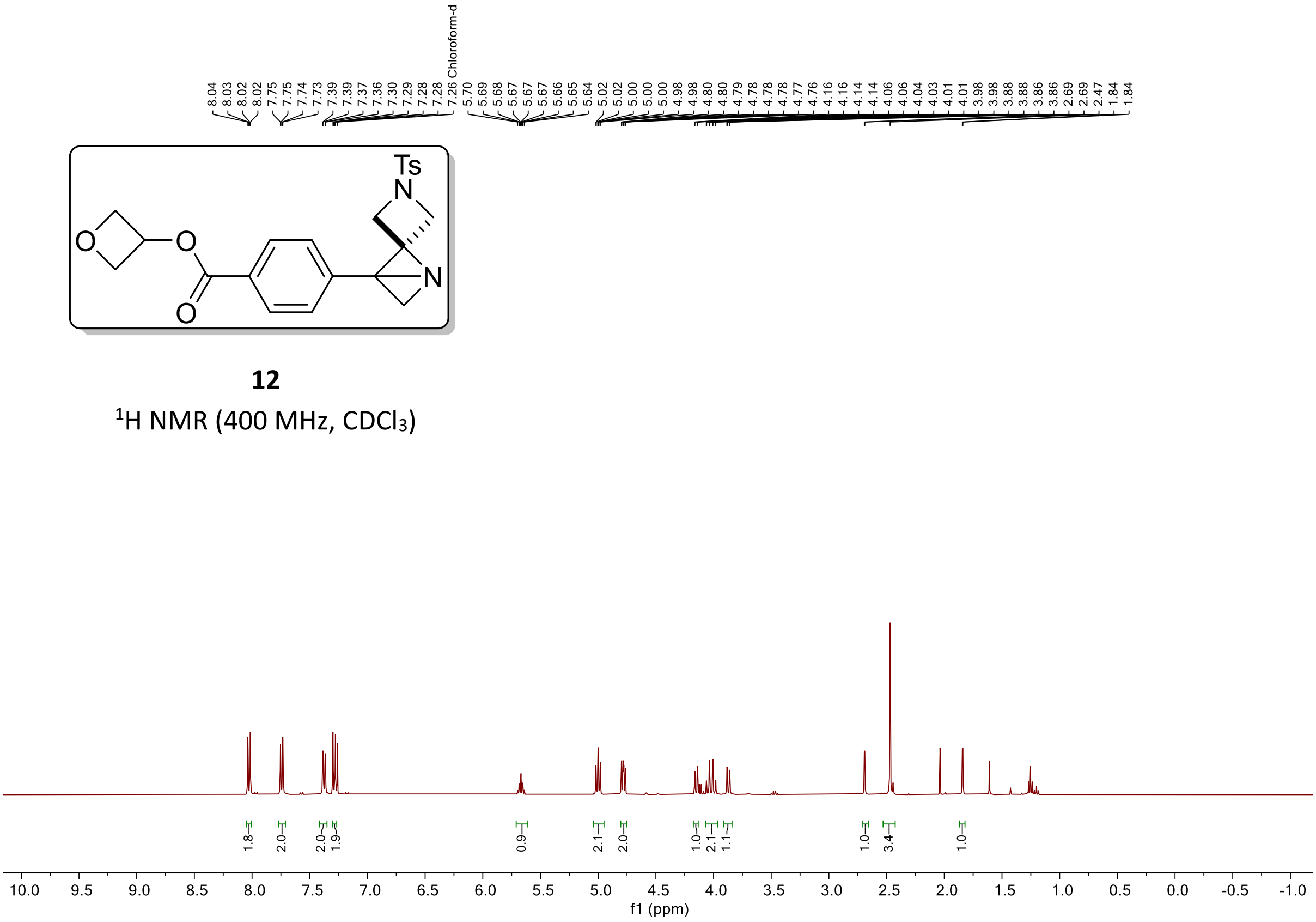

**12**

$^{1}H$ NMR (400 MHz, $CDCl_3$)

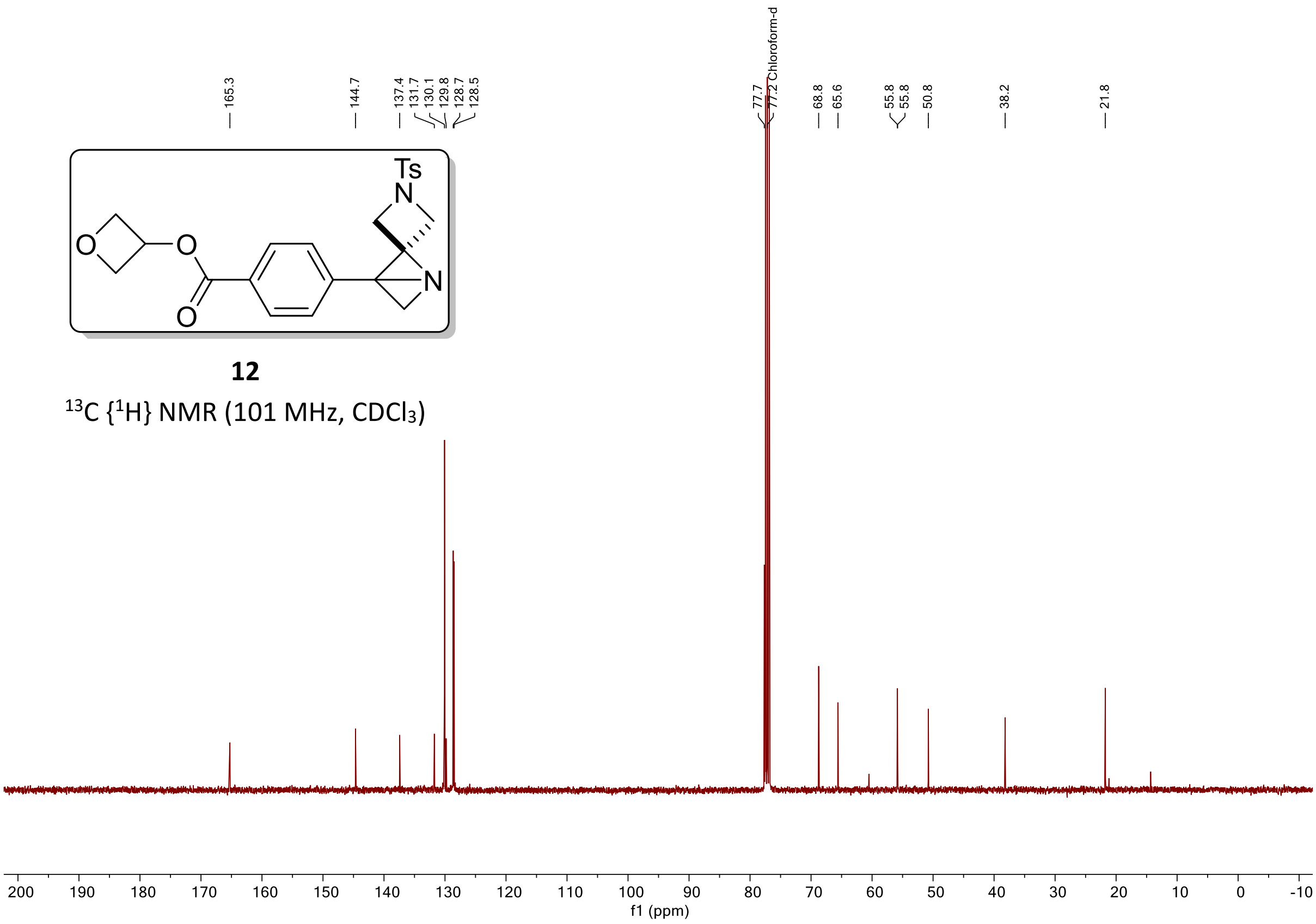

**12**

$^{13}C\{^{1}H\}$ NMR (101 MHz, $CDCl_3$)

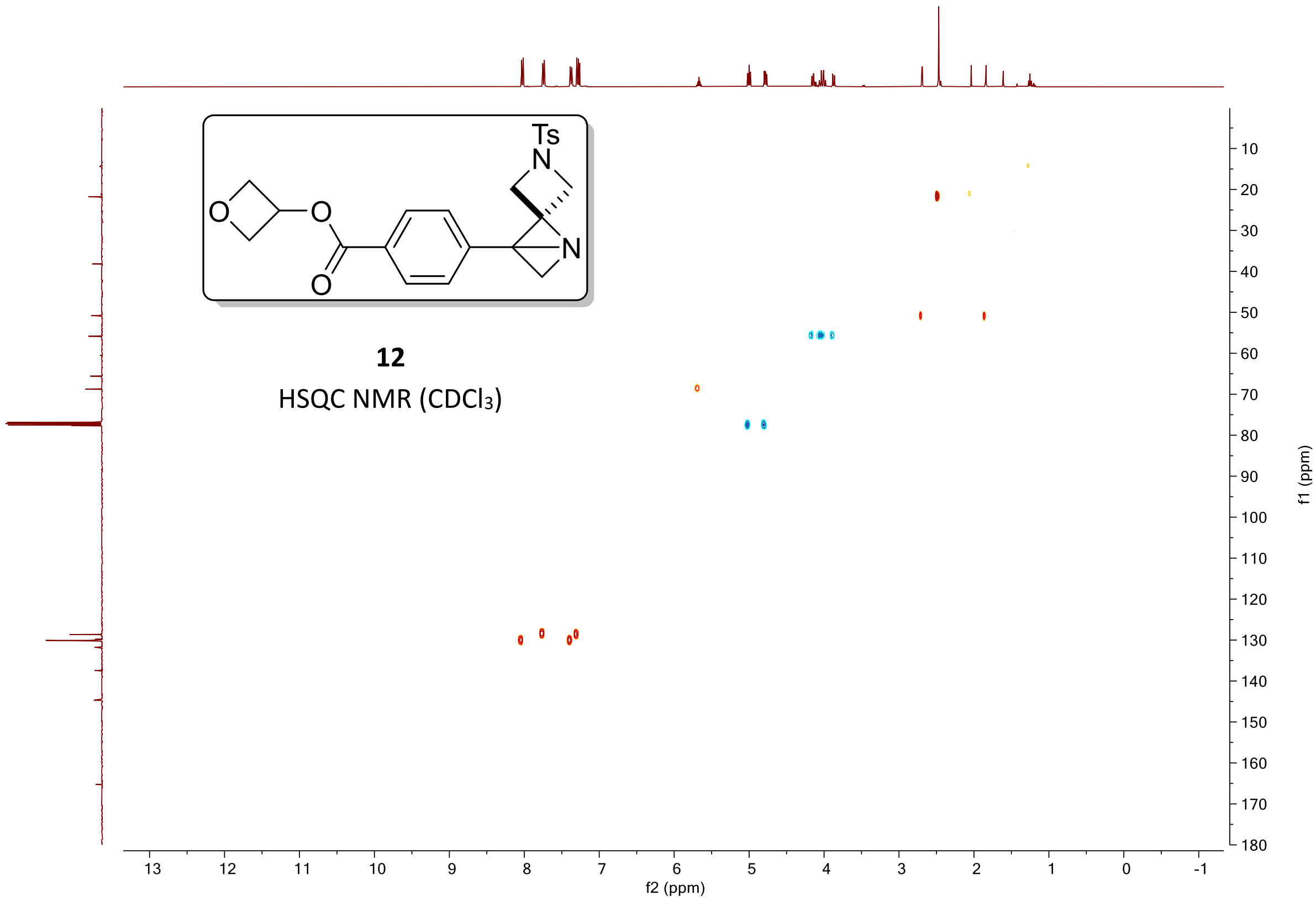


**12**

HSQC NMR ($CDCl_3$)

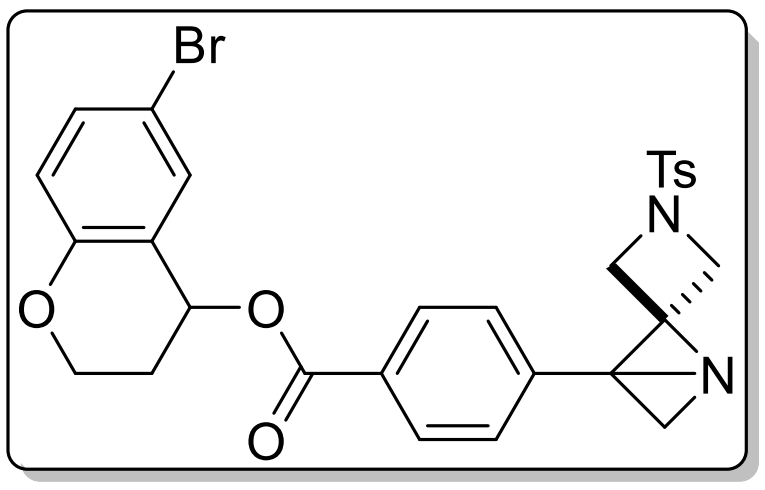


**13**

$^{1}$H NMR (400 MHz, $CDCl_3$)

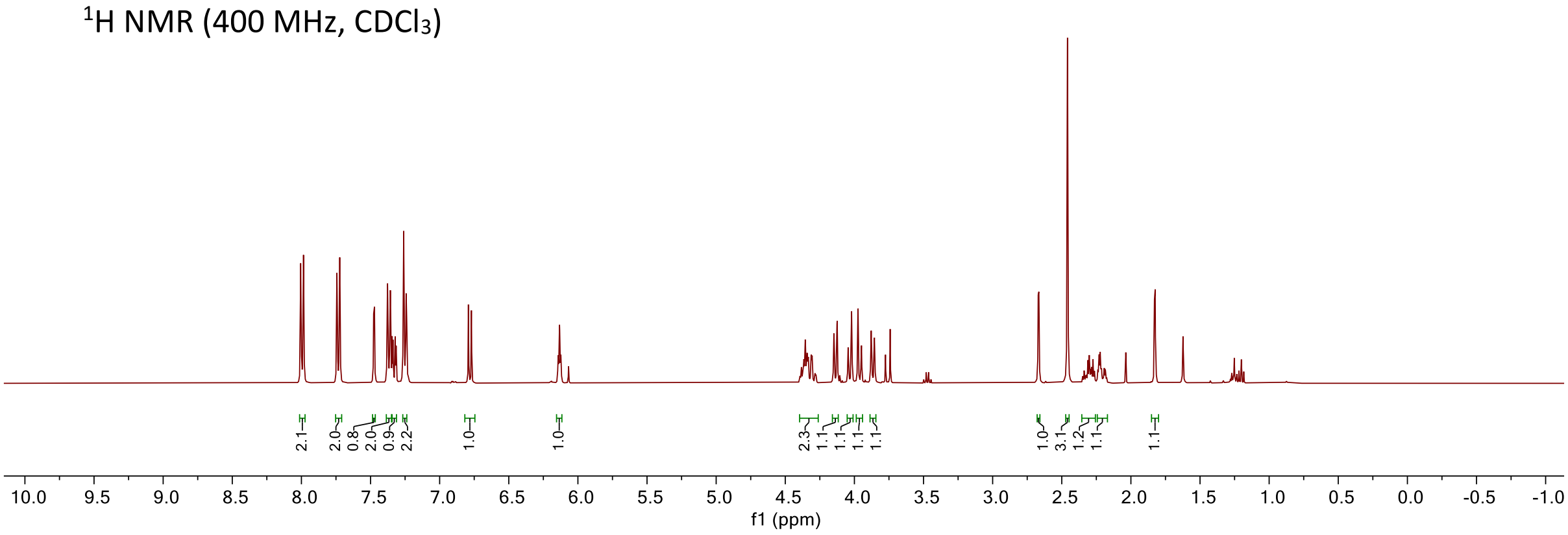


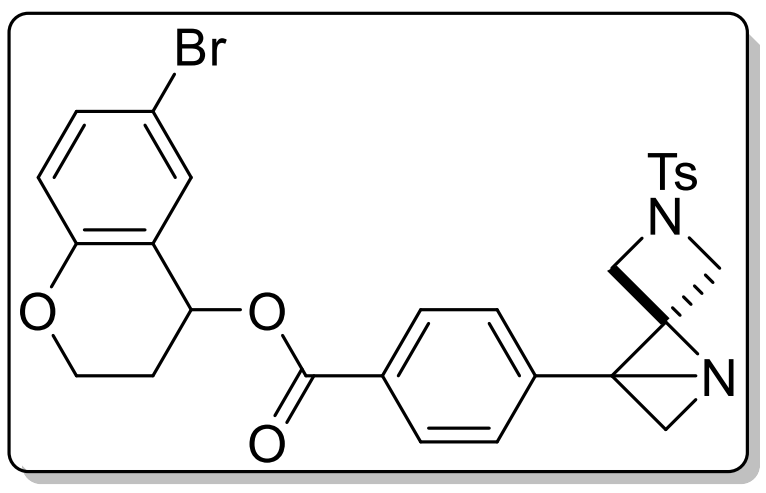


**13**

$^{13}$C {$^{1}$H} NMR (101 MHz, $CDCl_3$)

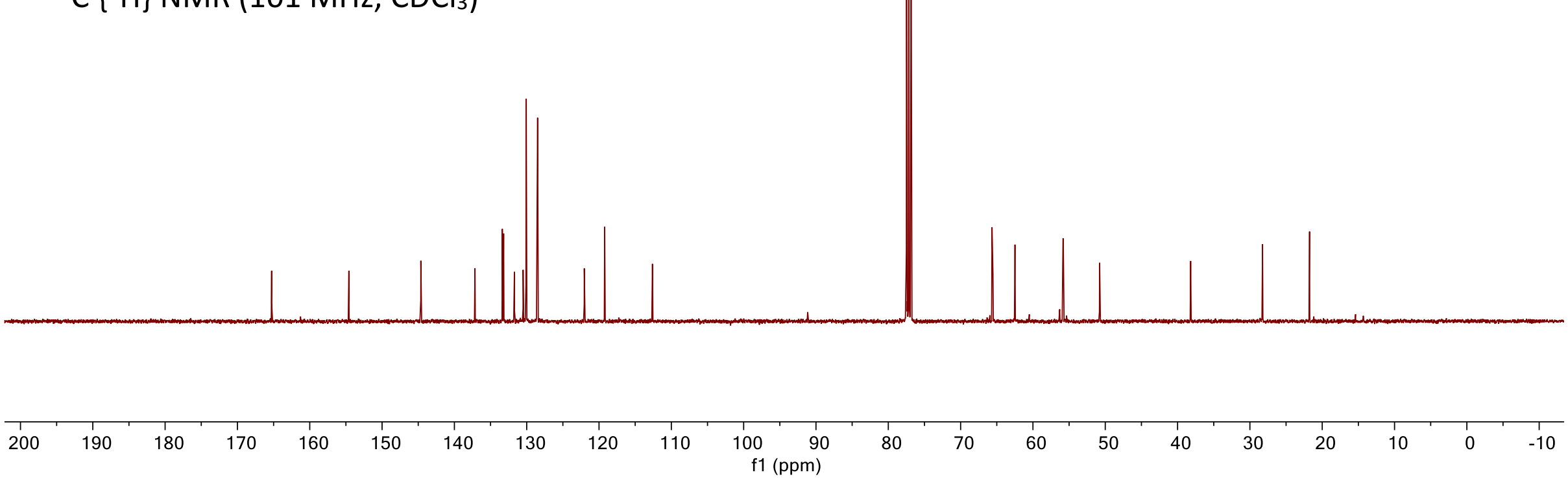

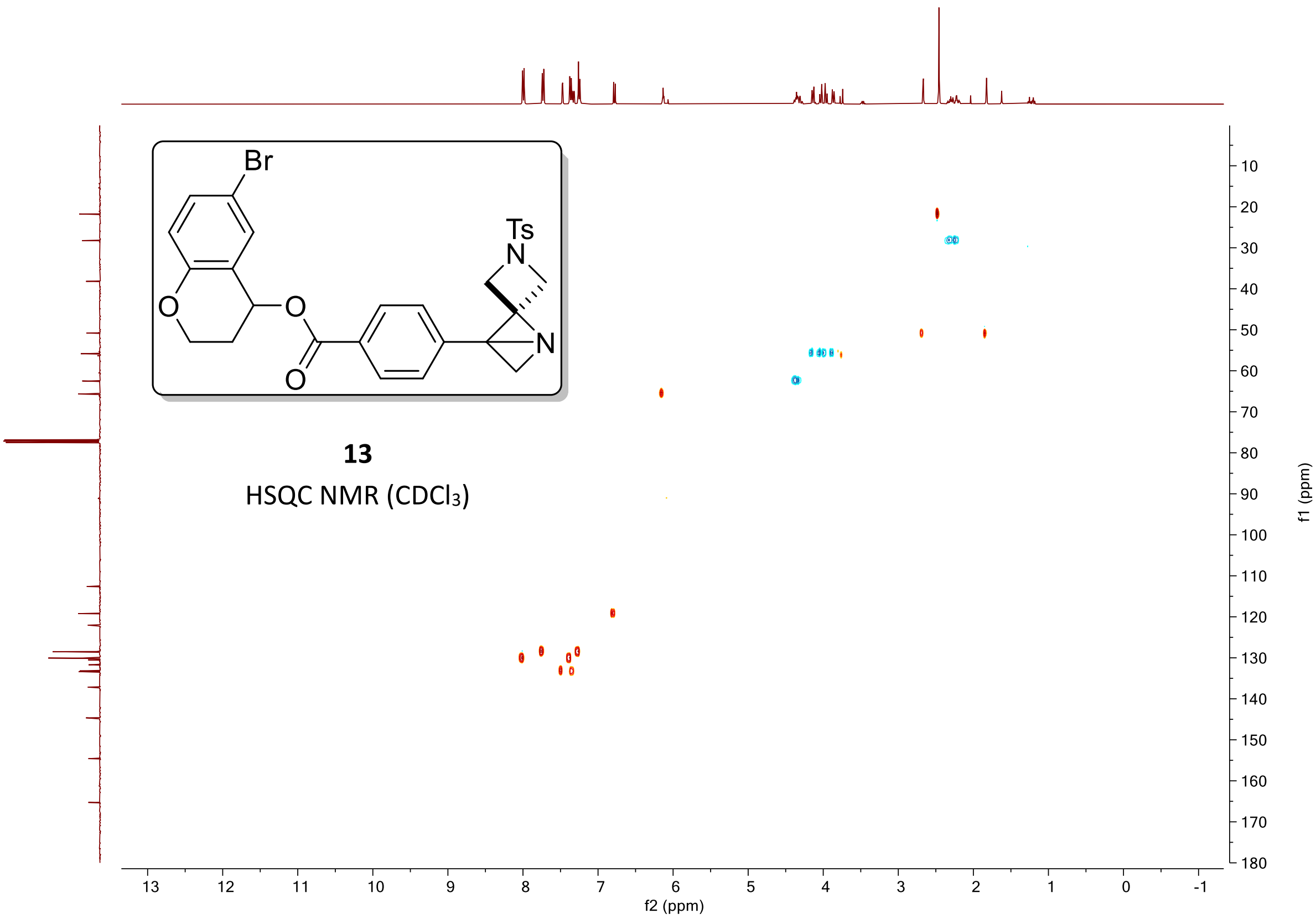


**13**

HSQC NMR ($CDCl_3$)

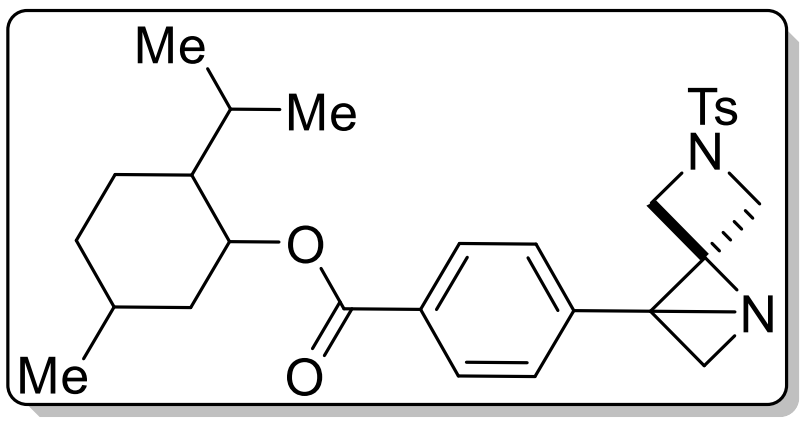

**14**

$^{1}$H NMR (400 MHz, $CDCl_3$)

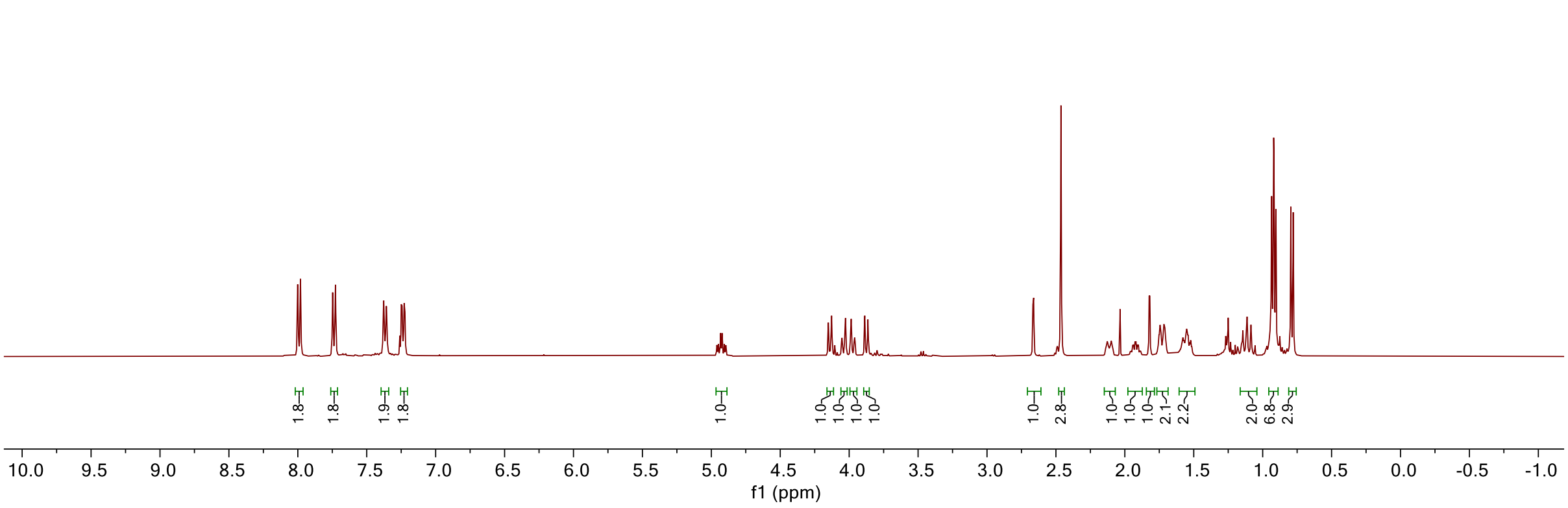

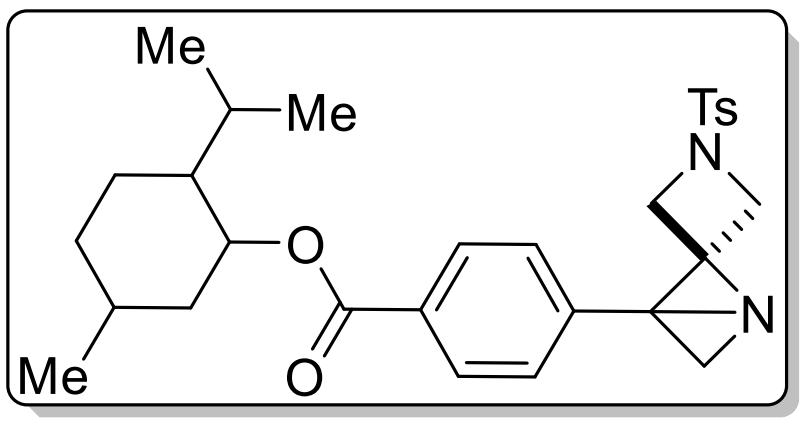

**14**

$^{13}$C {$^{1}$H} NMR (101 MHz, $CDCl_3$)

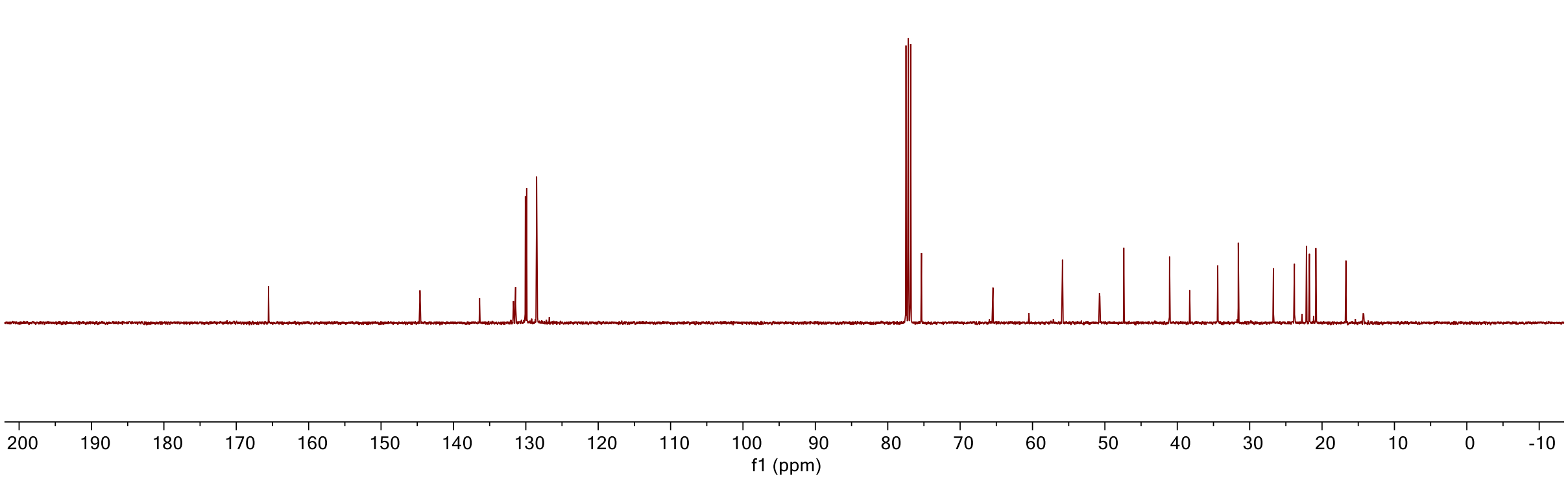

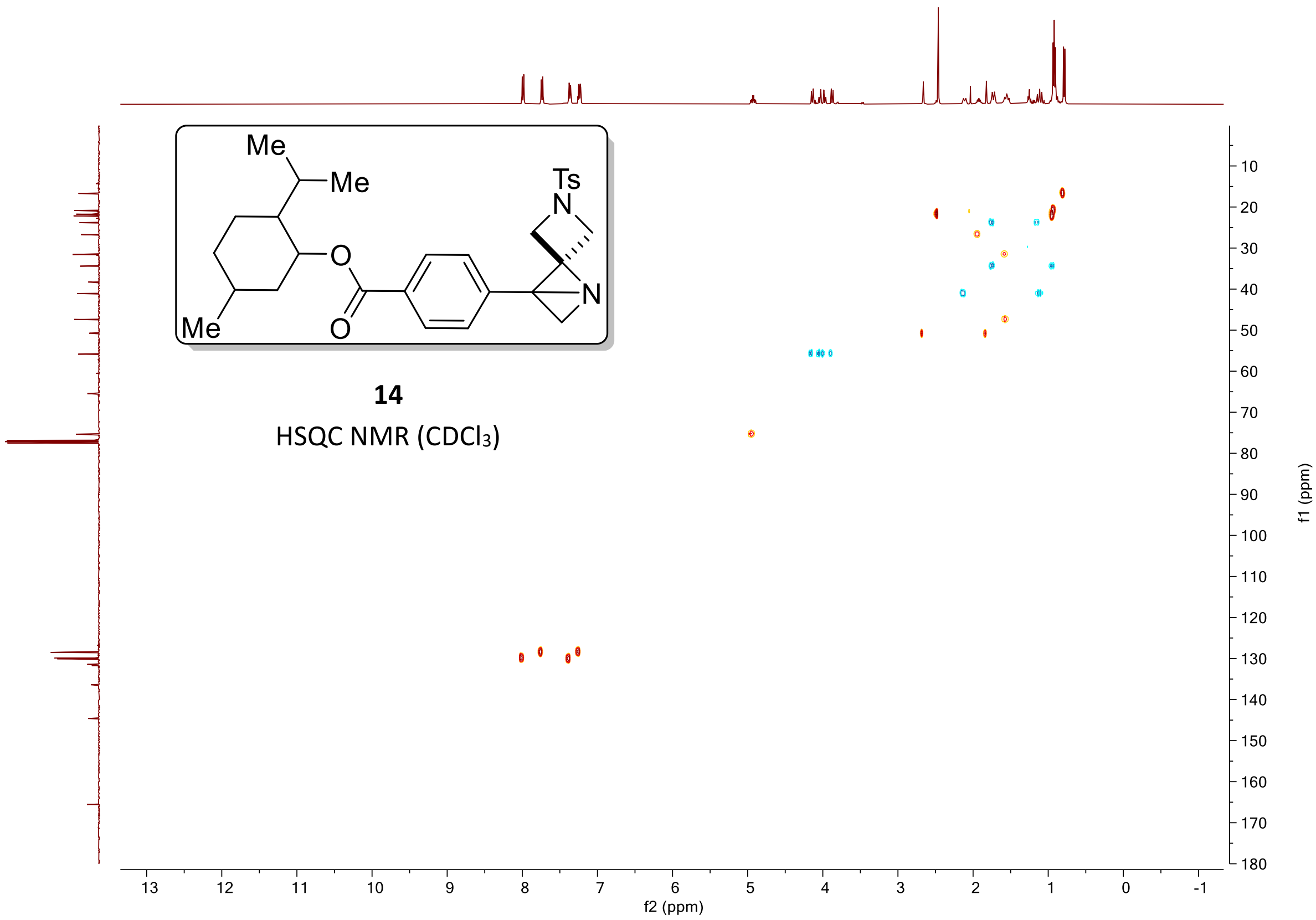


**14**

HSQC NMR ($CDCl_3$)

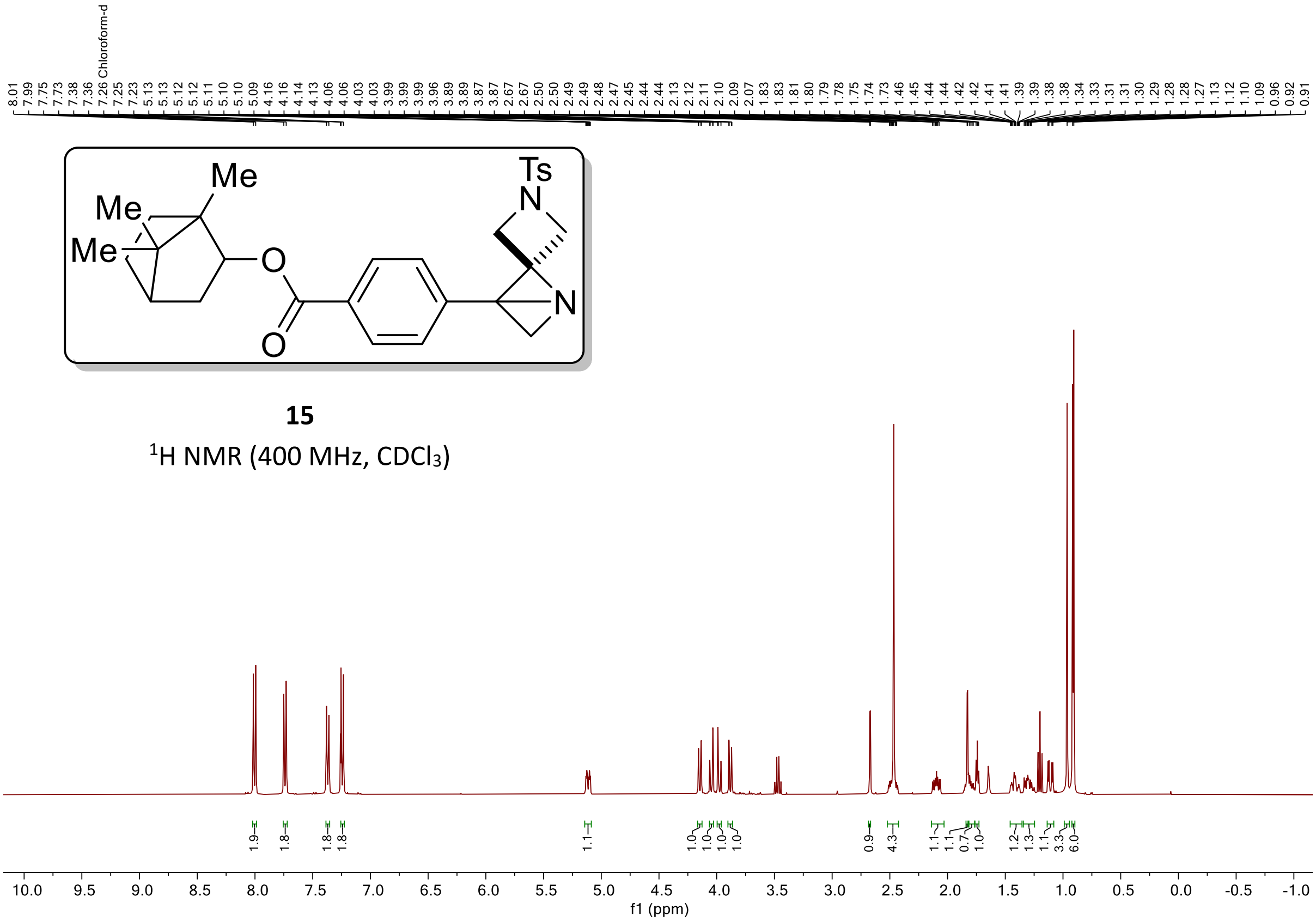

**15**

$^{1}H$ NMR (400 MHz, $CDCl_3$)

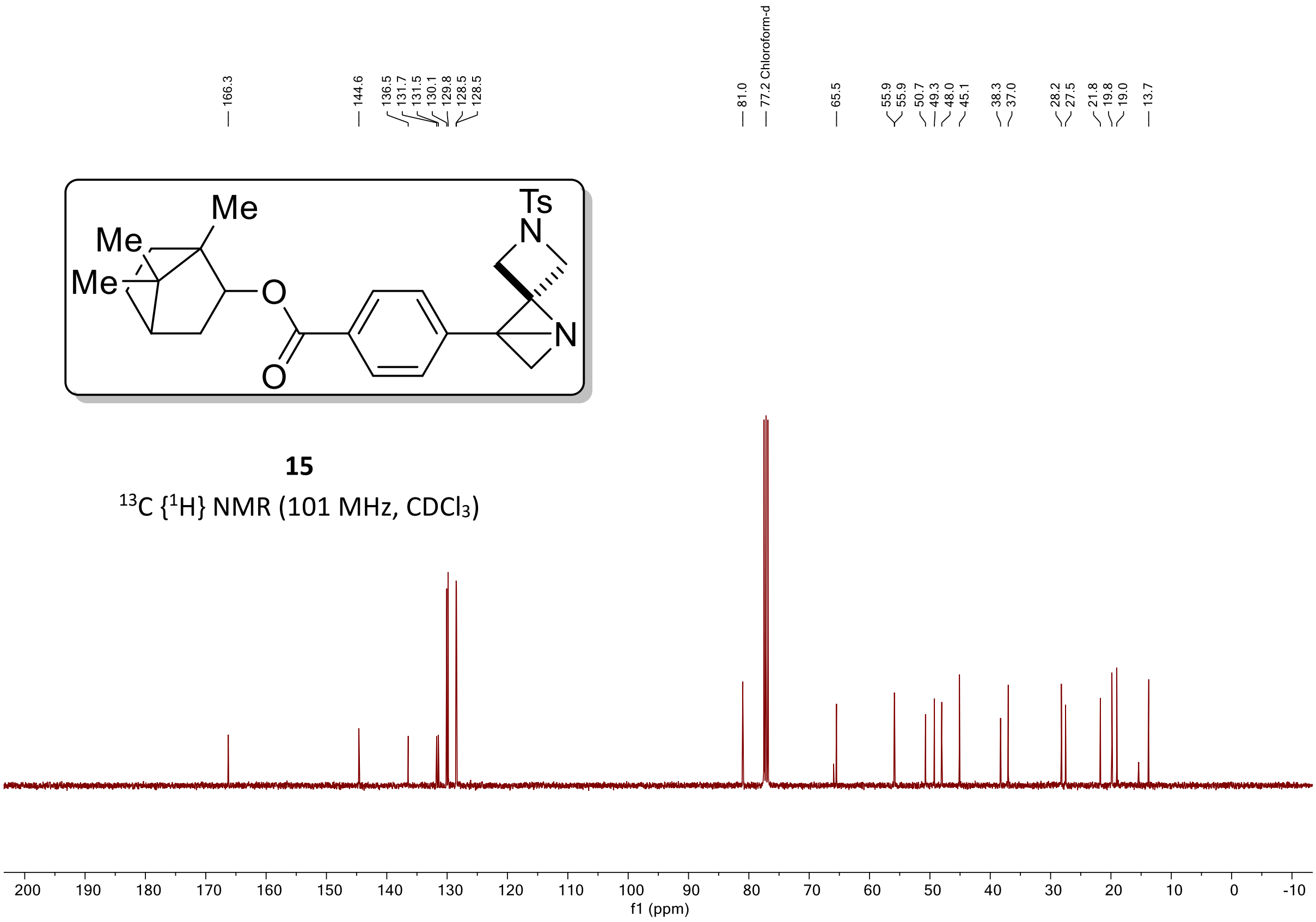

**15**

$^{13}C$ {$^{1}H$} NMR (101 MHz, $CDCl_3$)

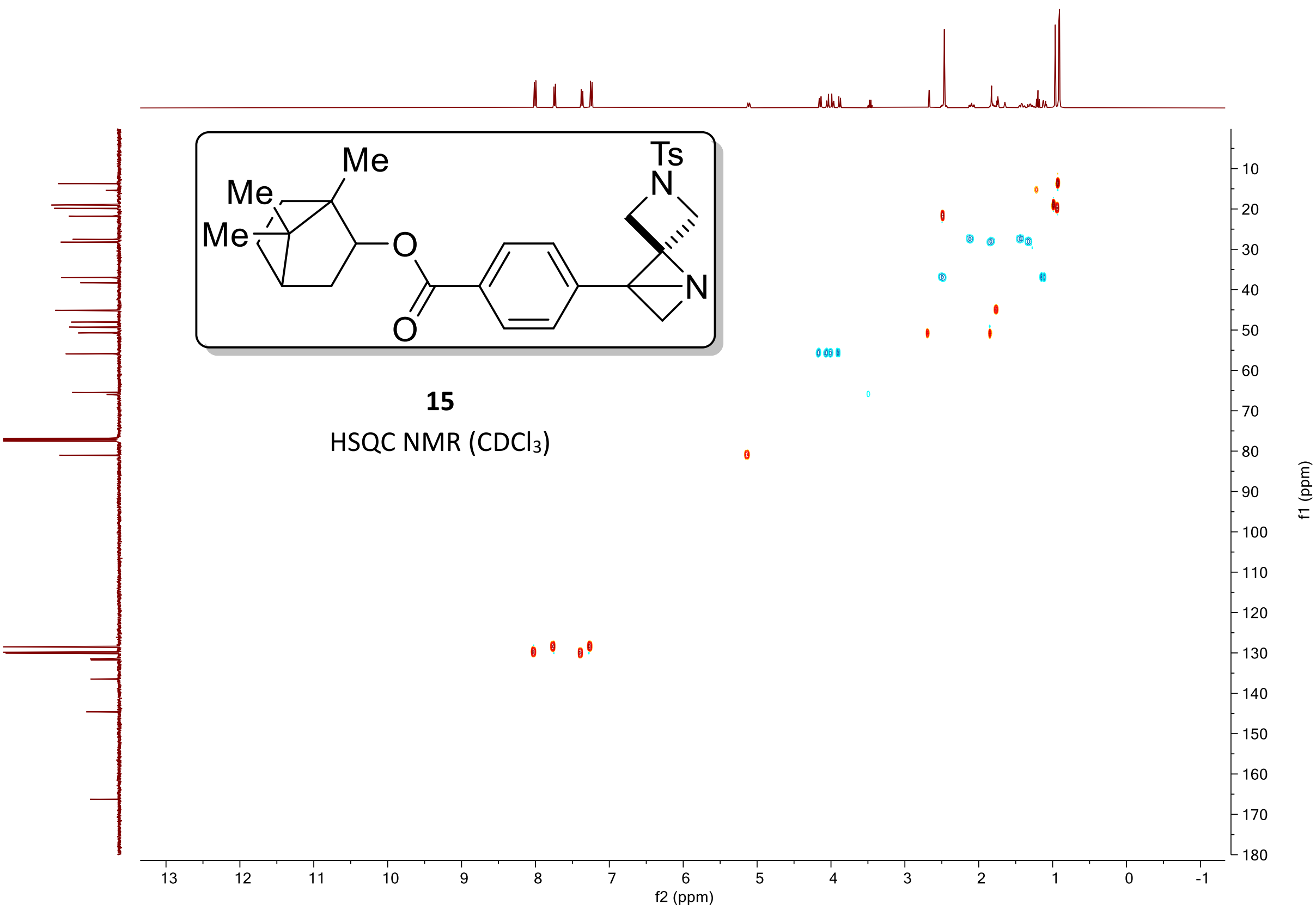


**15**

HSQC NMR ($CDCl_3$)

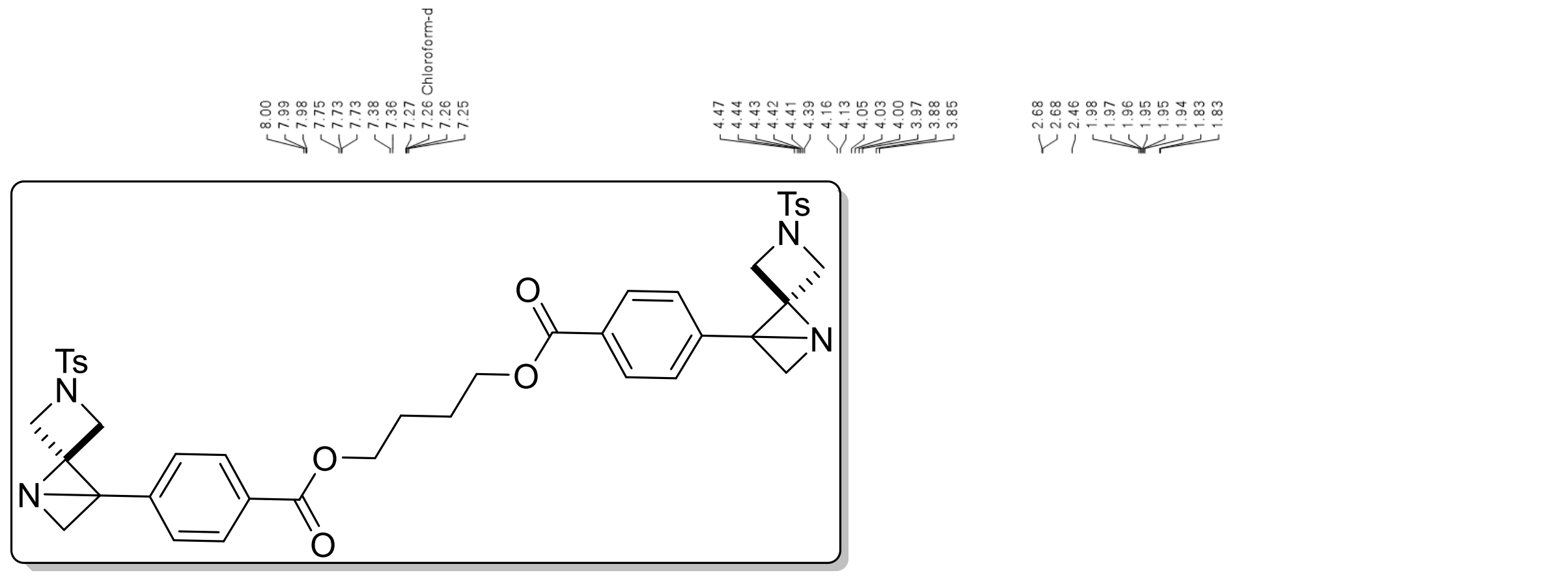


**16**

$^{1}$H NMR (400 MHz, $CDCl_3$)

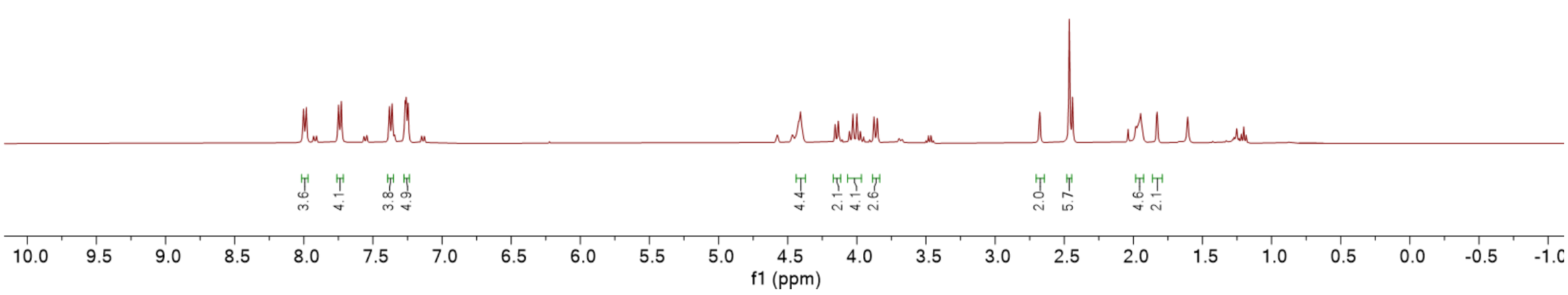


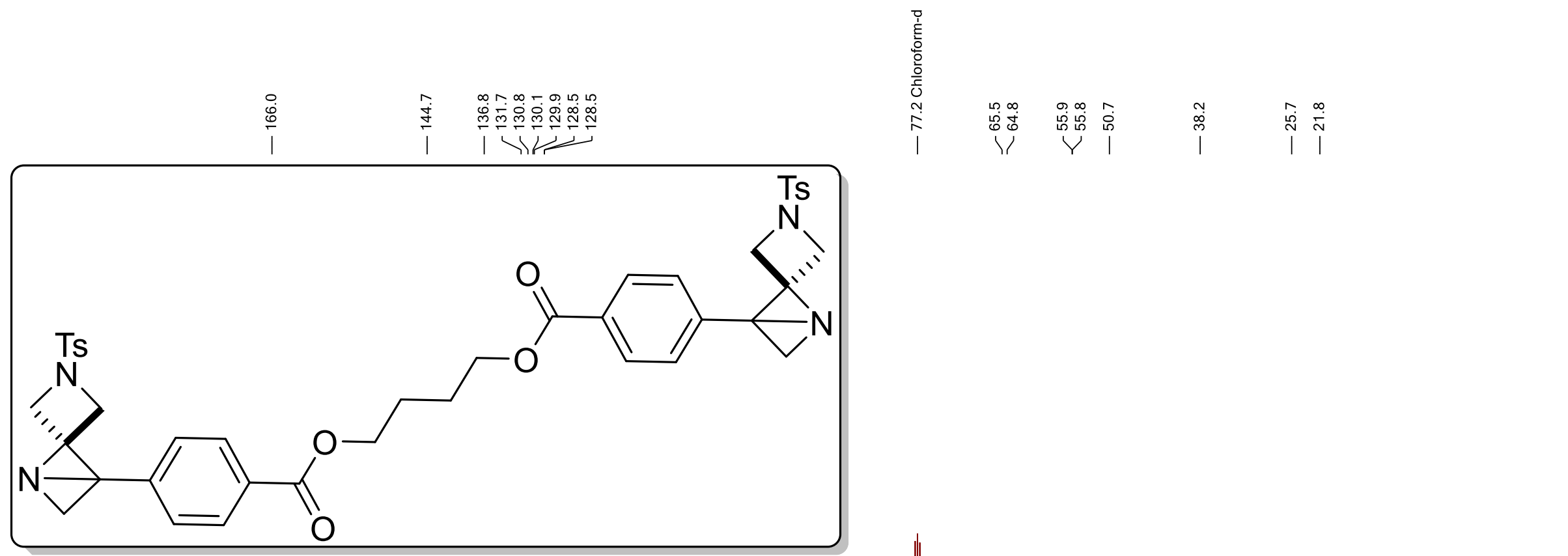


**16**

$^{13}$C {$^{1}$H} NMR (101 MHz, $CDCl_3$)

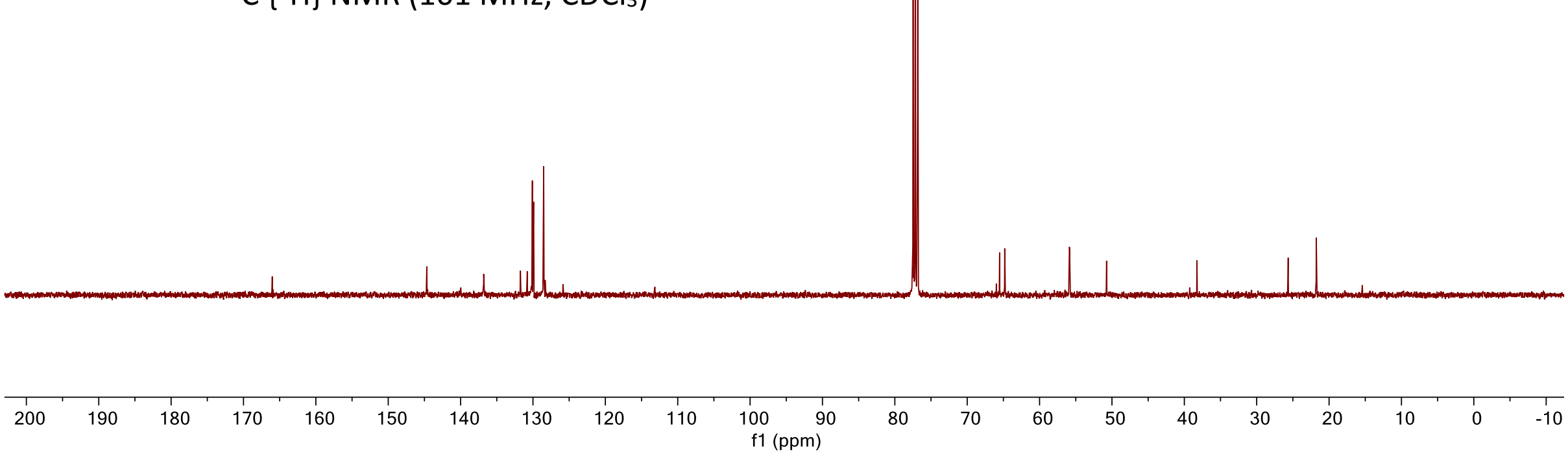

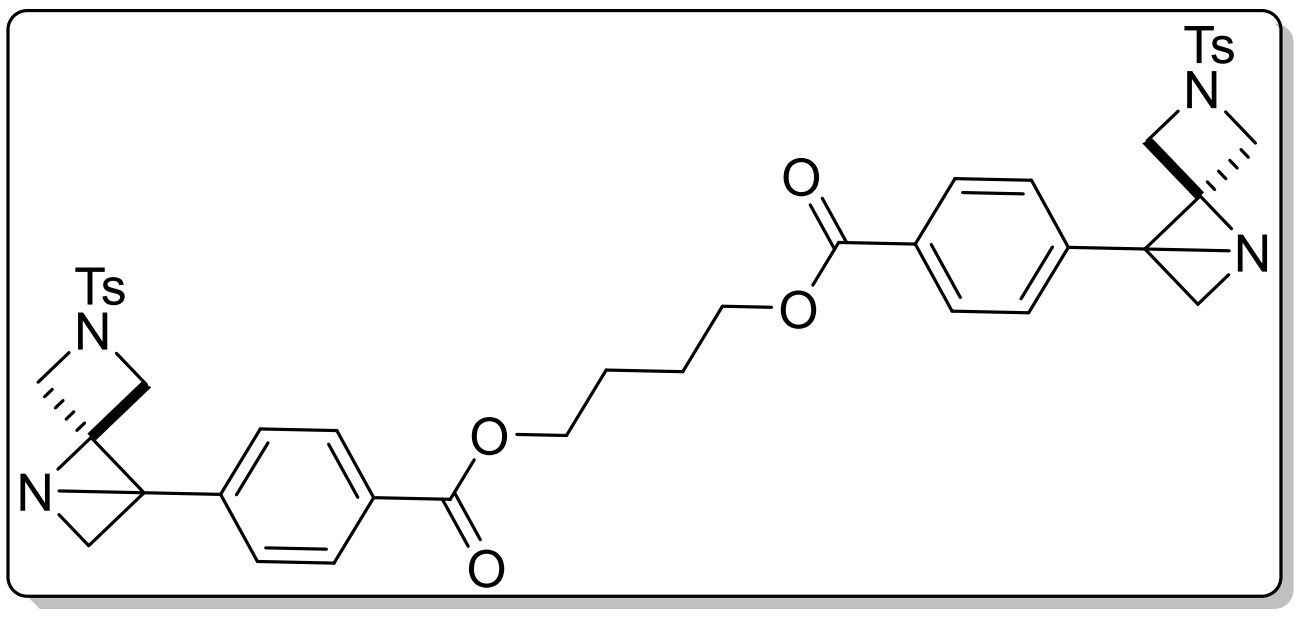

**16**

HSQC NMR ($CDCl_3$)

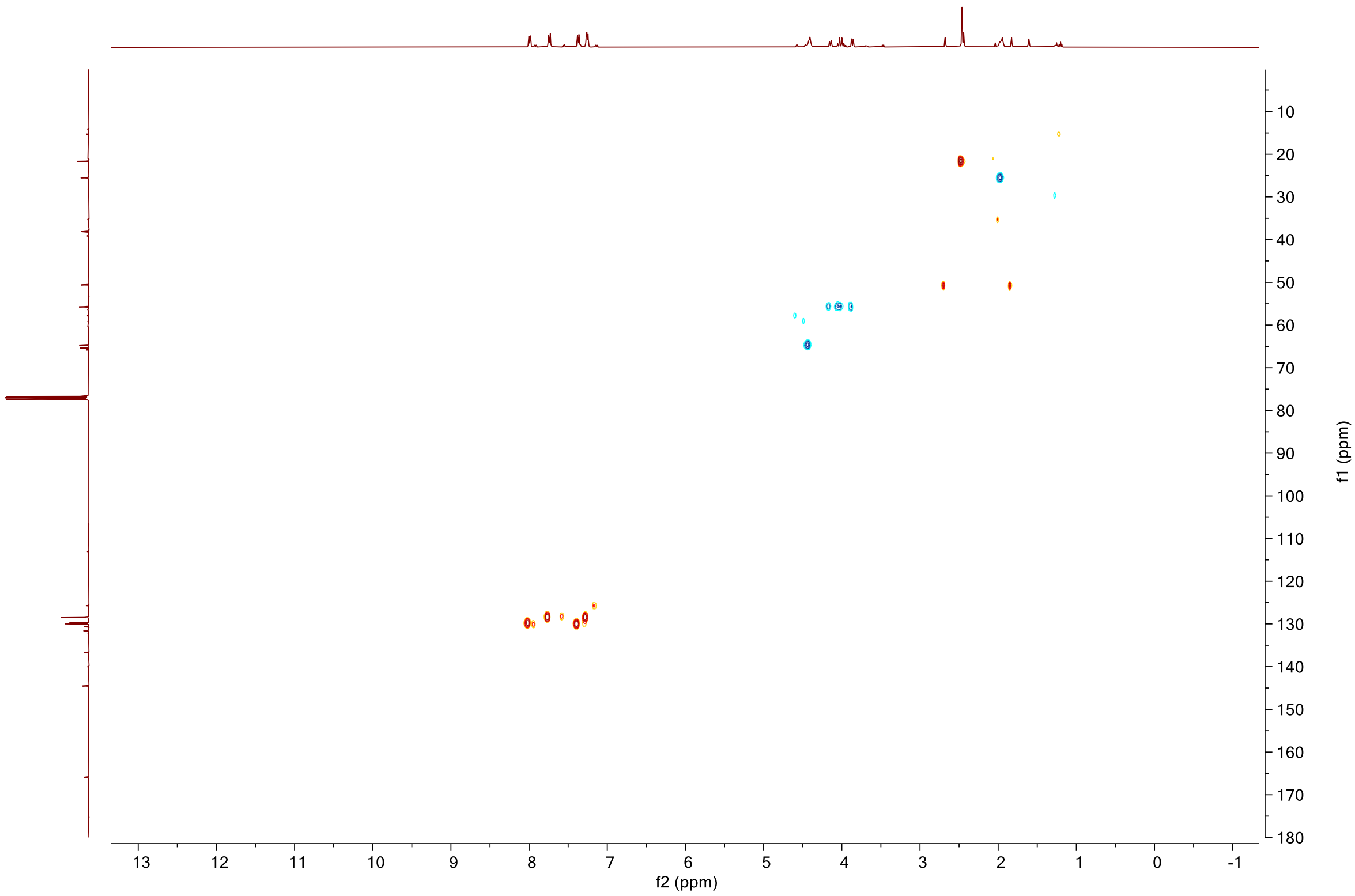

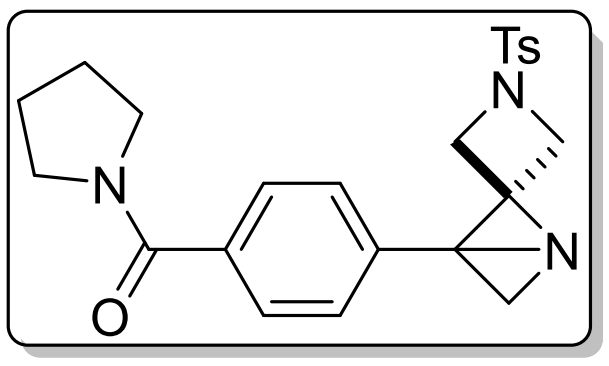

**17**

$^{1}H$ NMR (400 MHz, $CDCl_3$)

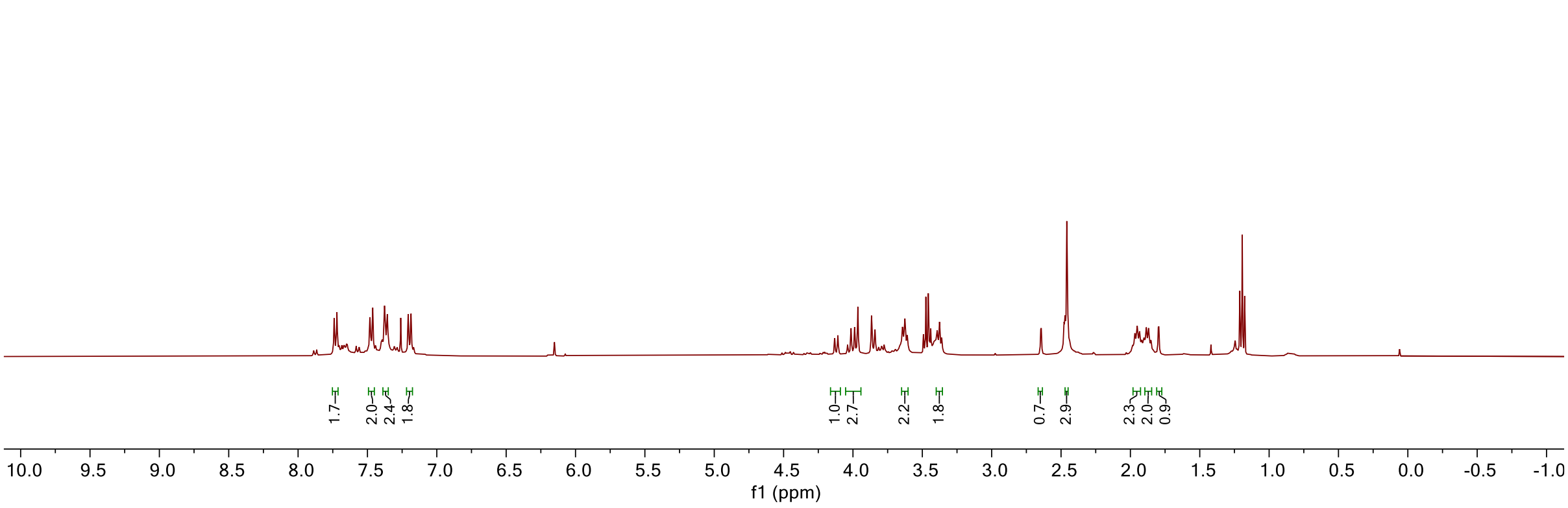


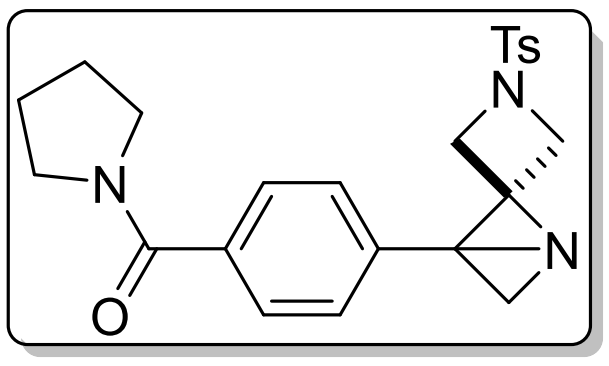

**17**

$^{13}C\{^{1}H\}$ NMR (101 MHz, $CDCl_3$)

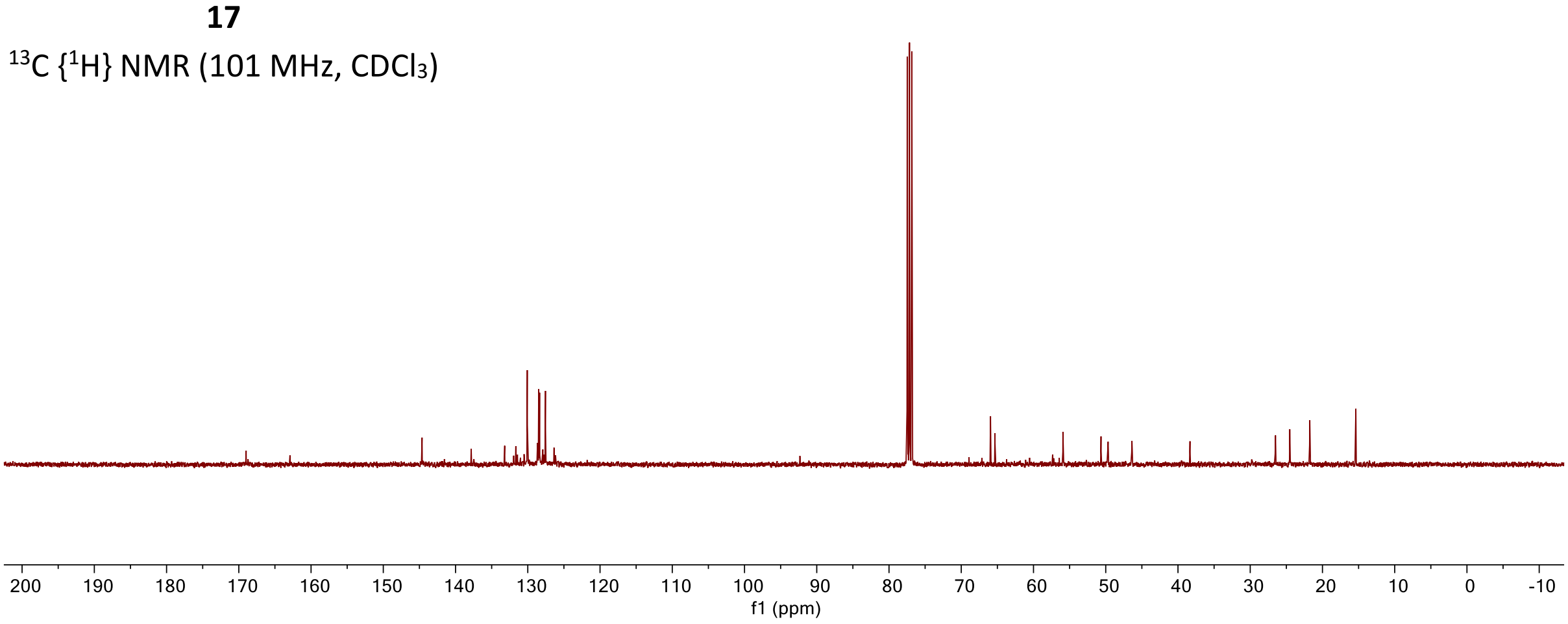

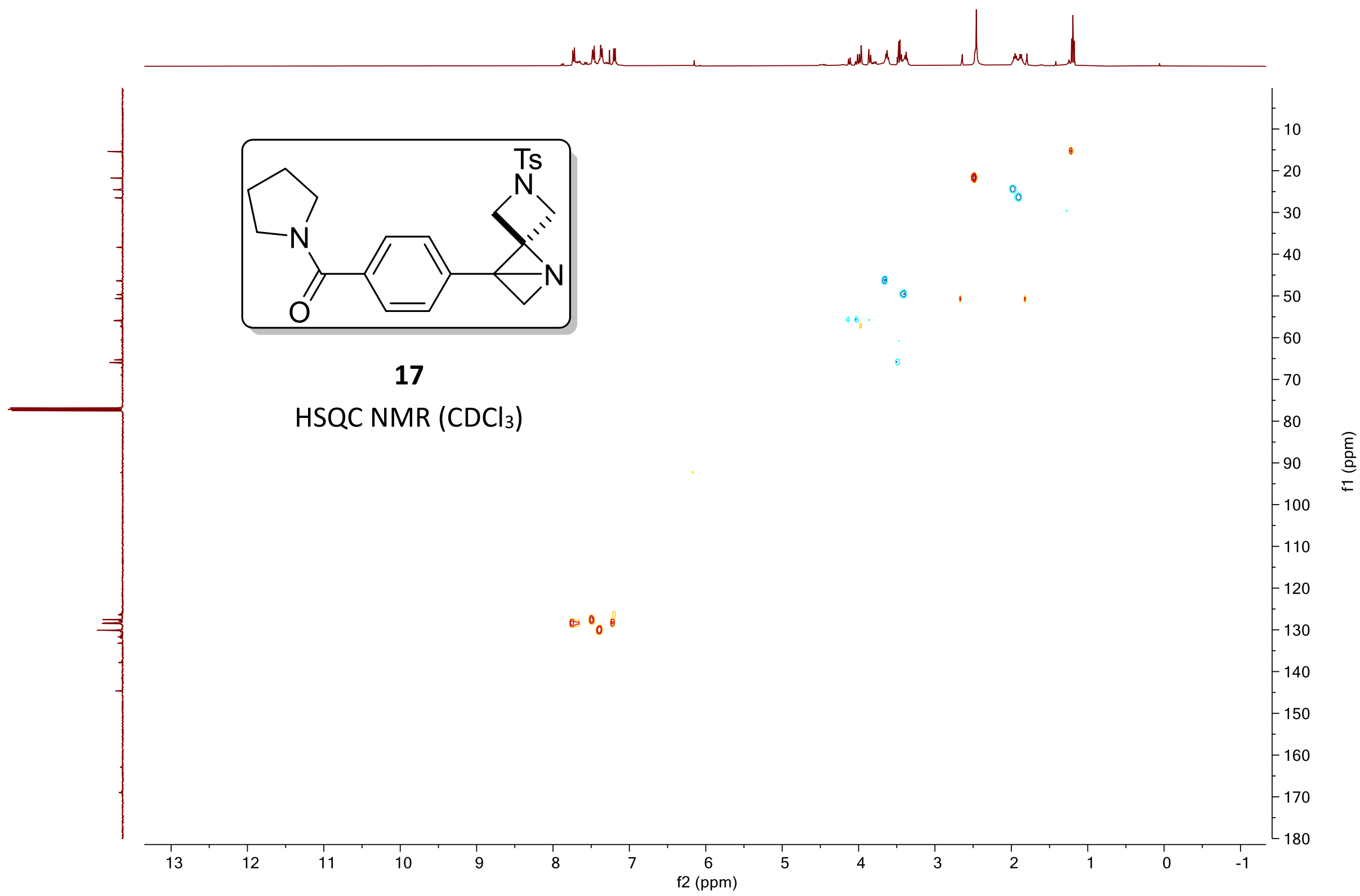
Ts
N
N
N
O
17
HSQC NMR (CDCl3)
f2 (ppm)
f1 (ppm)
13
12
11
10
9
8
7
6
5
4
3
2
1
0
-1
10
20
30
40
50
60
70
80
90
100
110
120
130
140
150
160
170
180

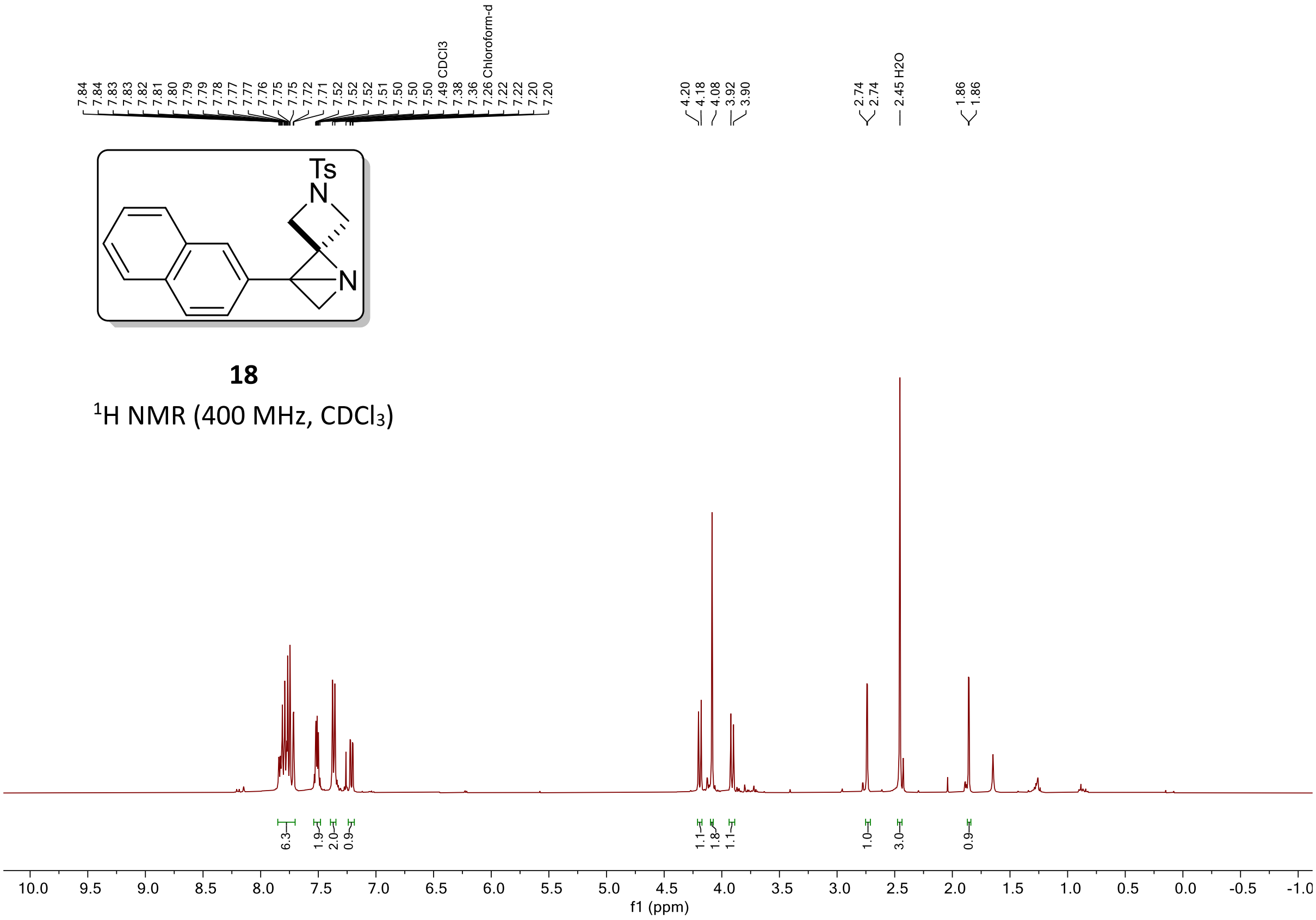


**18**

$^{1}H$ NMR (400 MHz, $CDCl_3$)

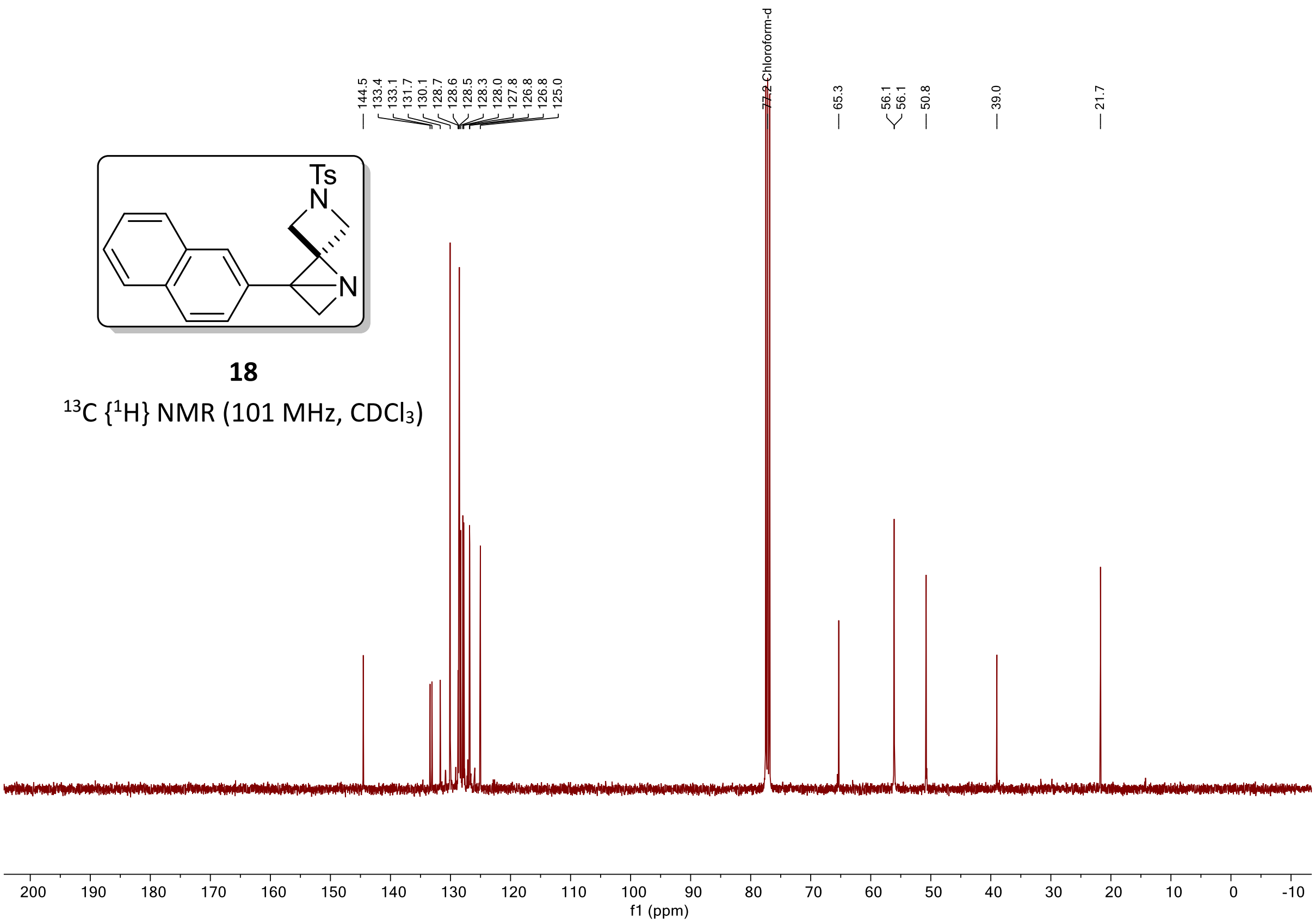


**18**

$^{13}C$ {$^{1}H$} NMR (101 MHz, $CDCl_3$)

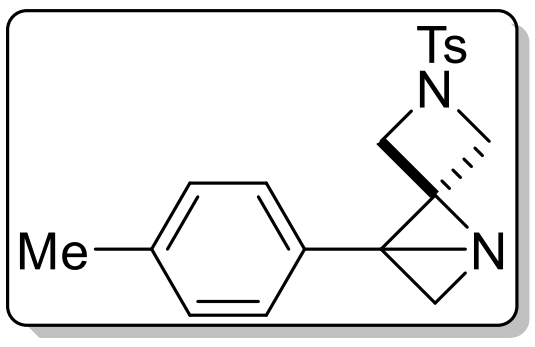


**19**

$^{1}$H NMR (400 MHz, $CDCl_3$)

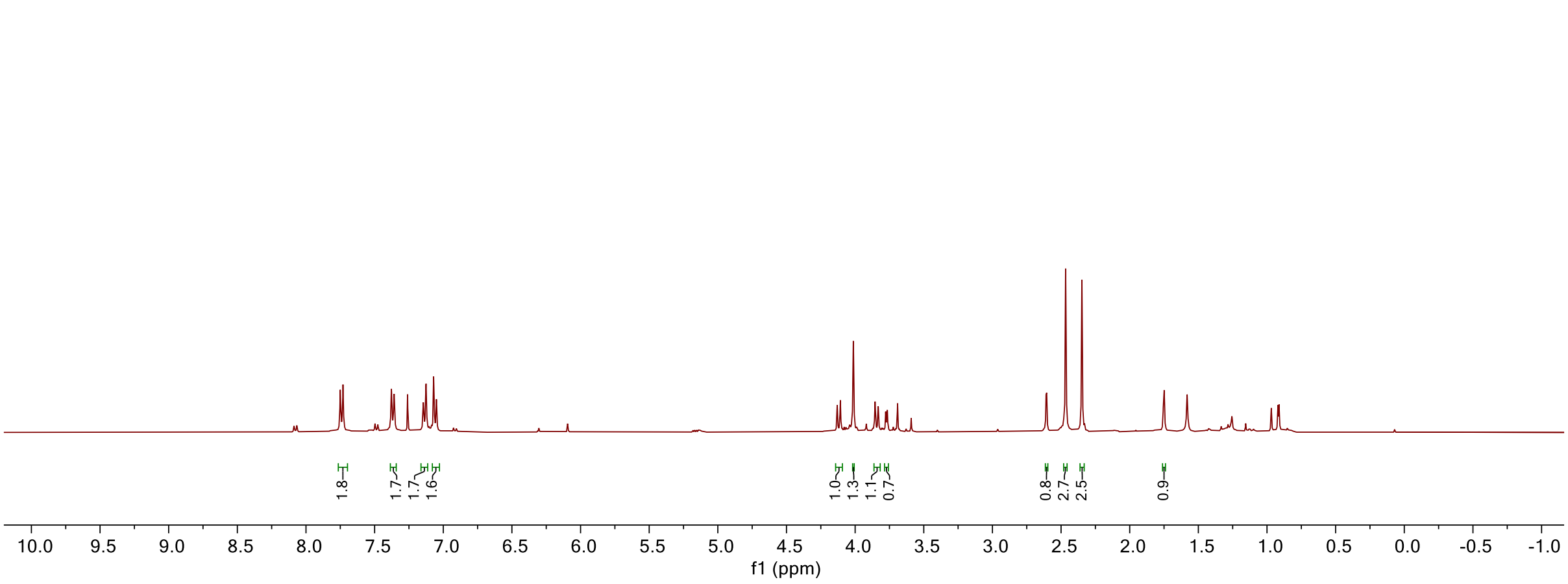


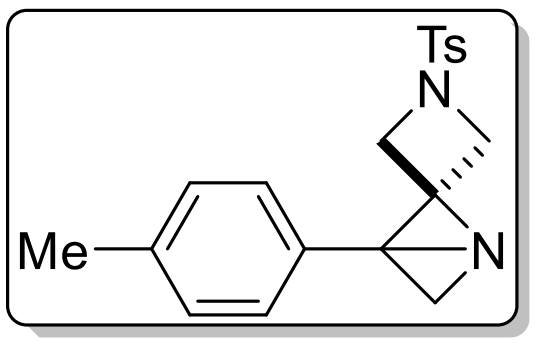


**19**

$^{13}$C {$^{1}$H} NMR (101 MHz, $CDCl_3$)

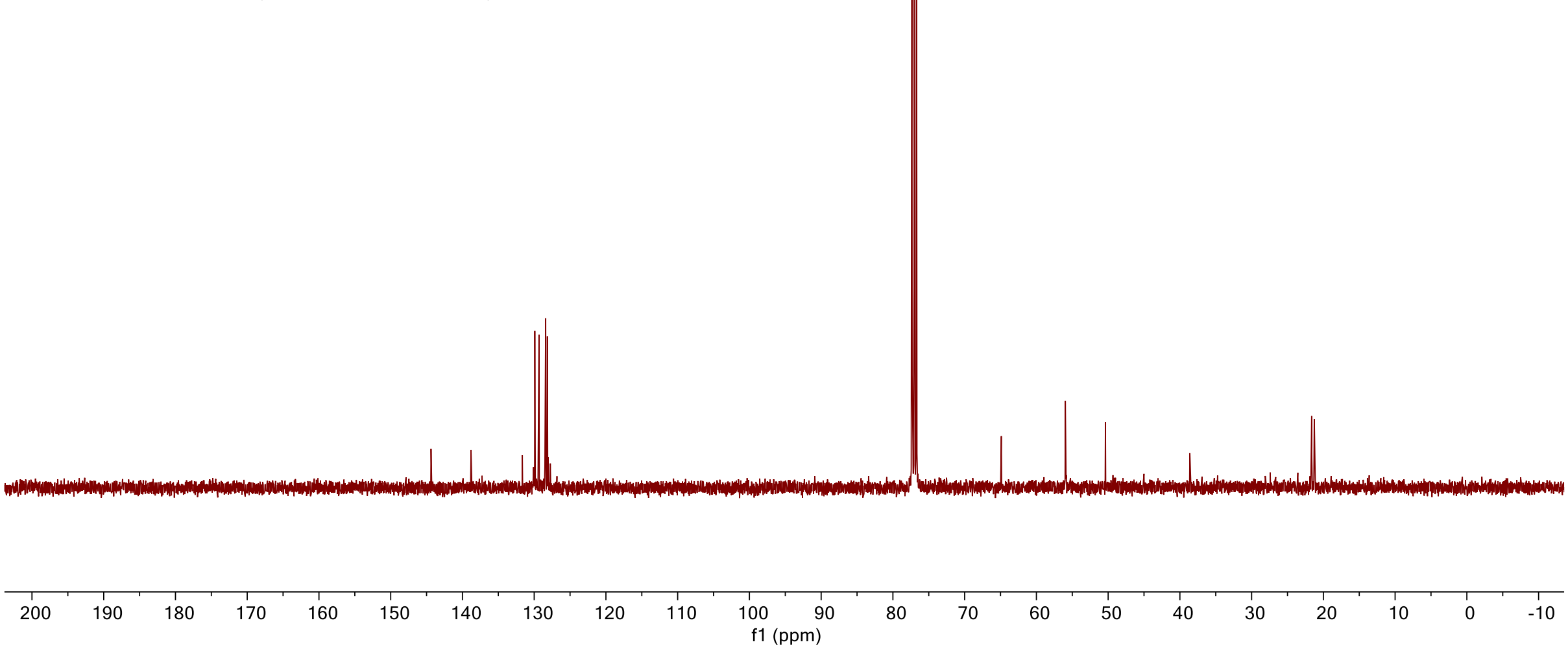

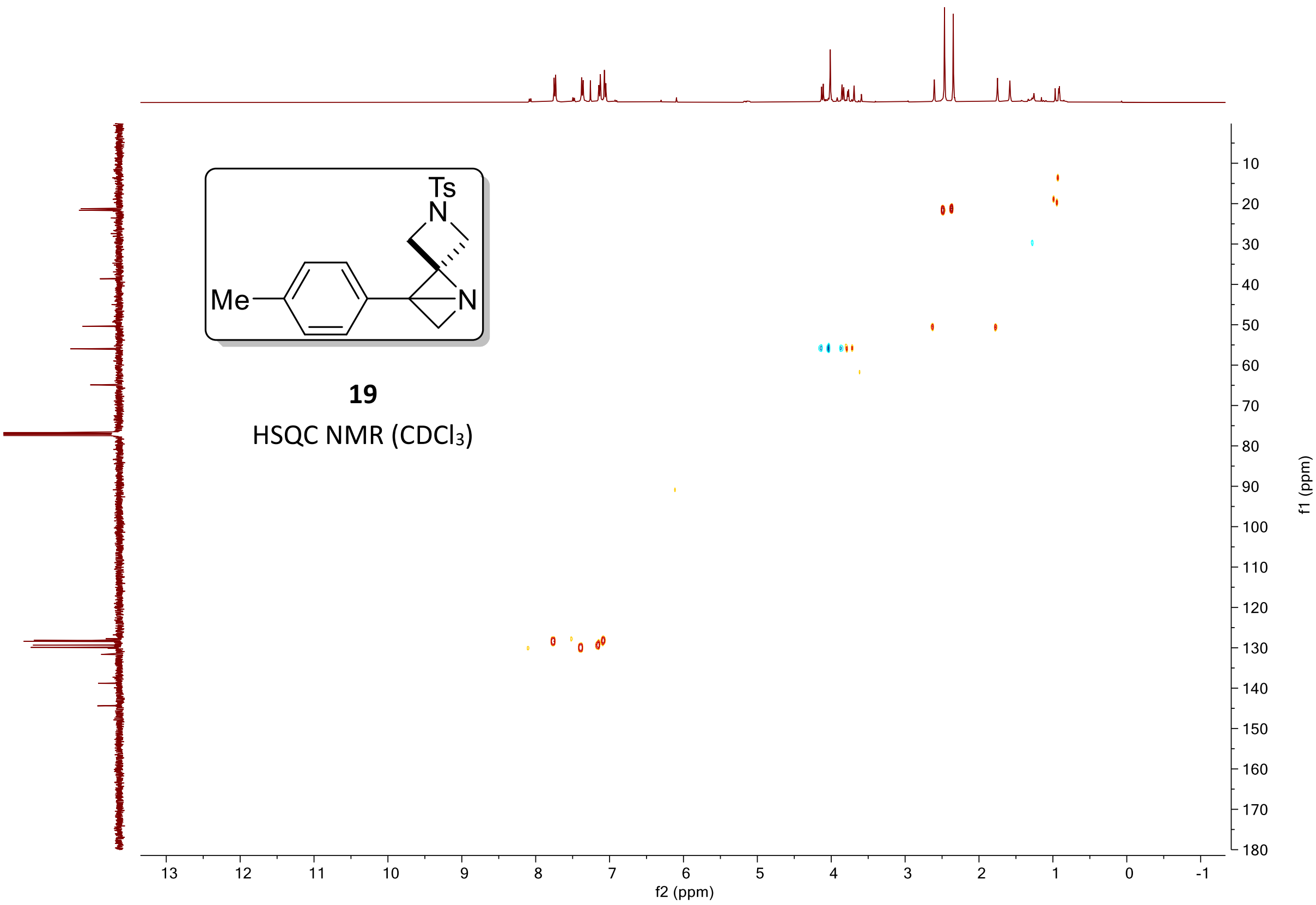
Ts
N
N
Me
19
HSQC NMR (CDCl3)
f2 (ppm)
f1 (ppm)

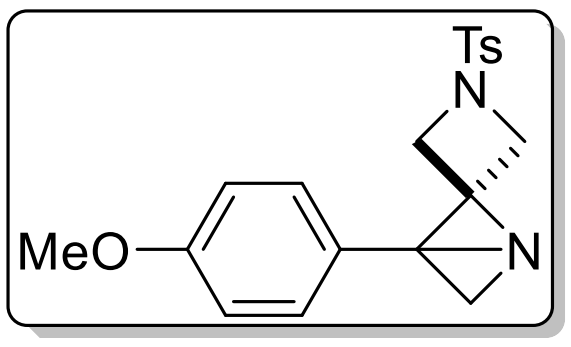

**20**

$^{1}H$ NMR (400 MHz, $CDCl_3$)

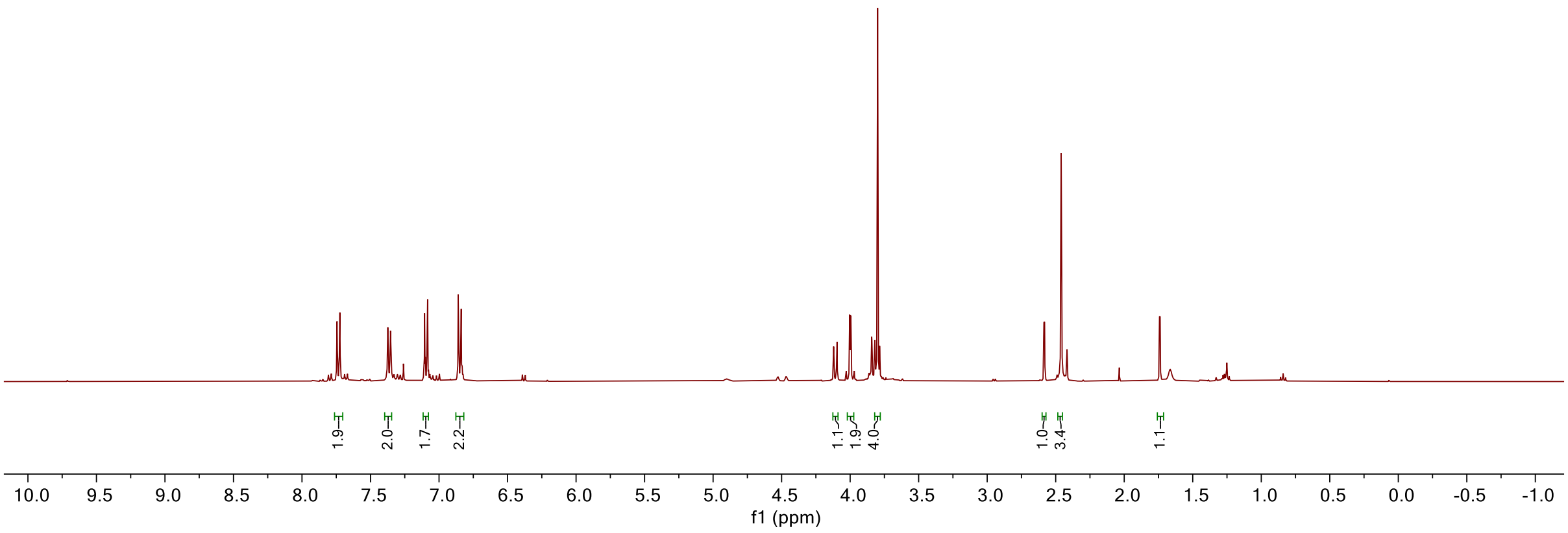

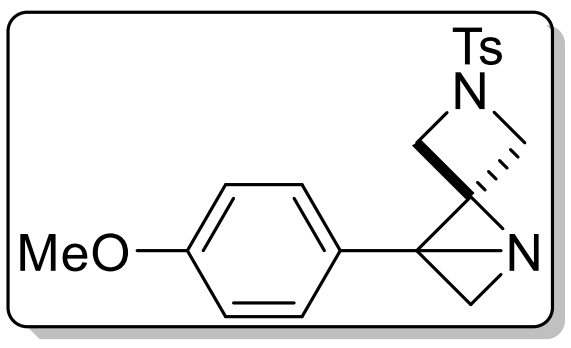

**20**

$^{13}C\{^{1}H\}$ NMR (101 MHz, $CDCl_3$)

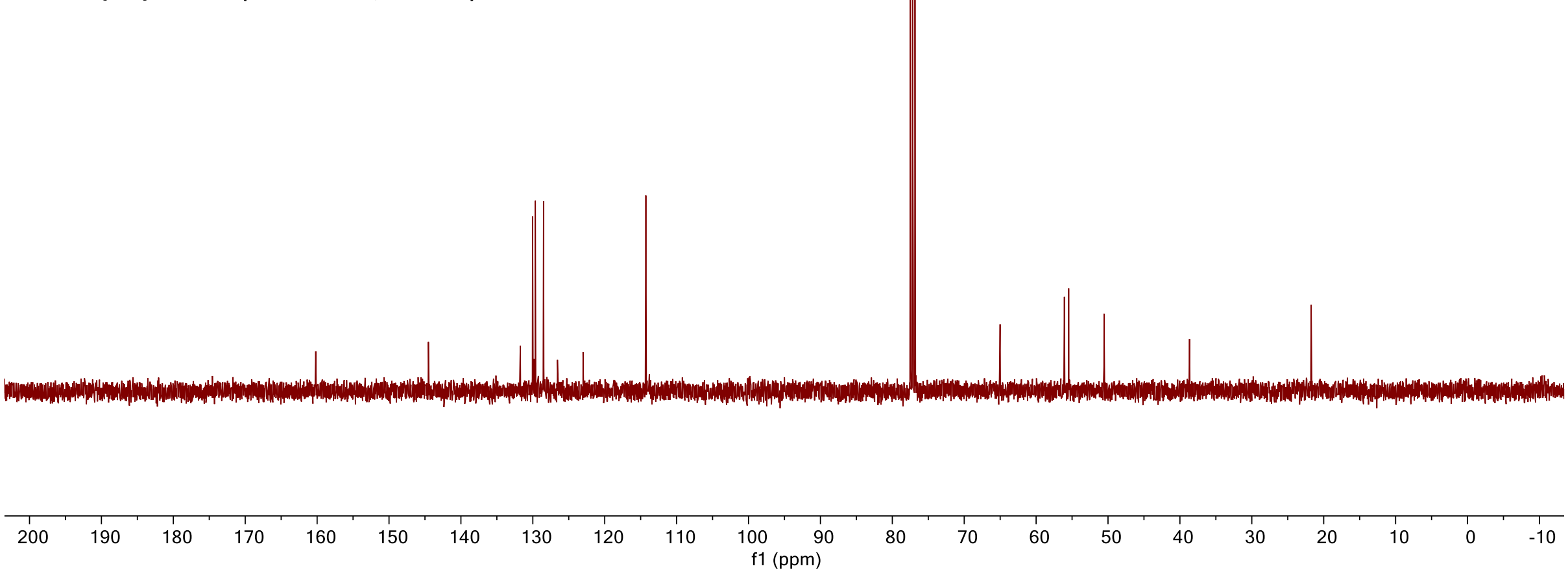

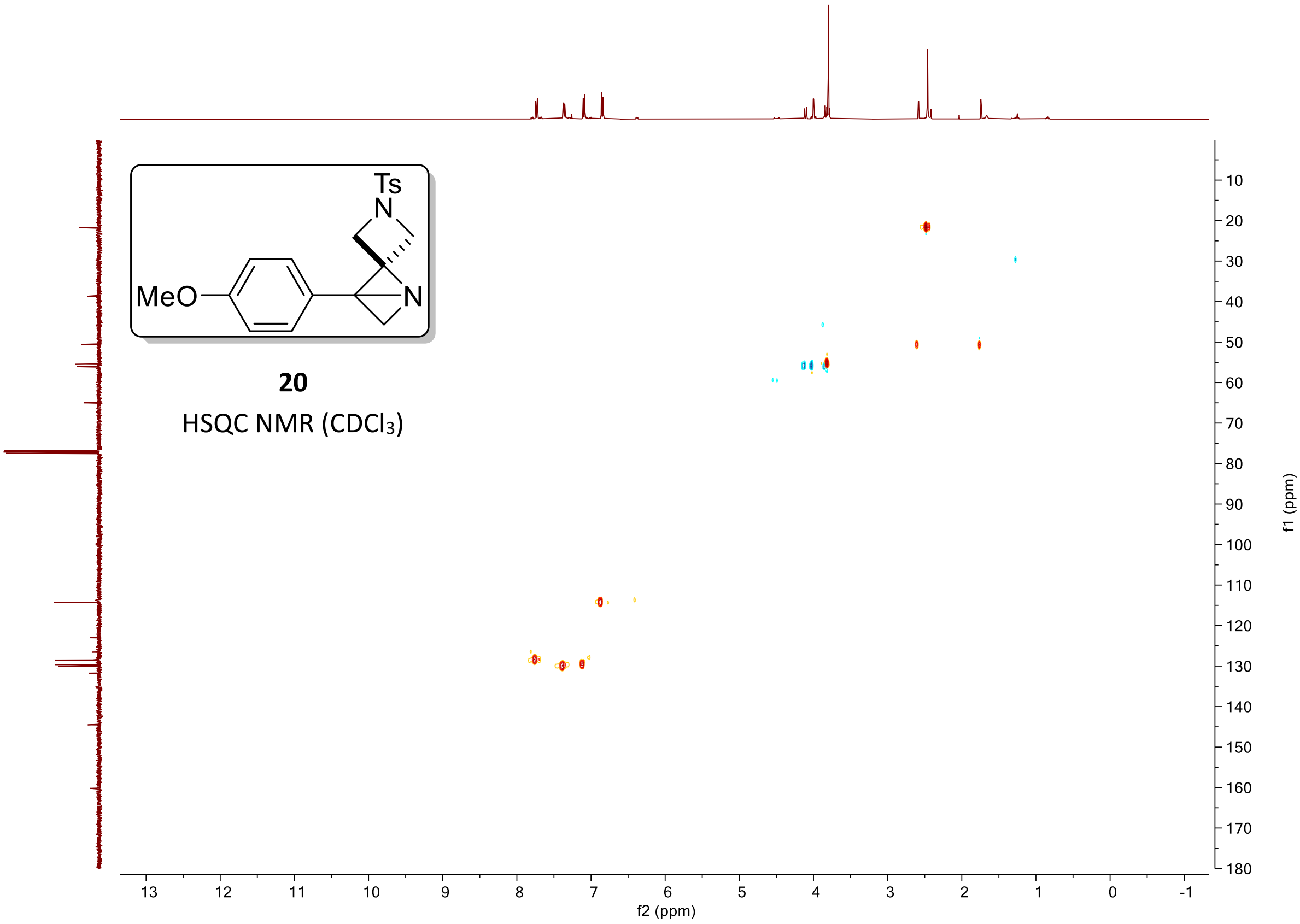


**20**

HSQC NMR ($CDCl_3$)

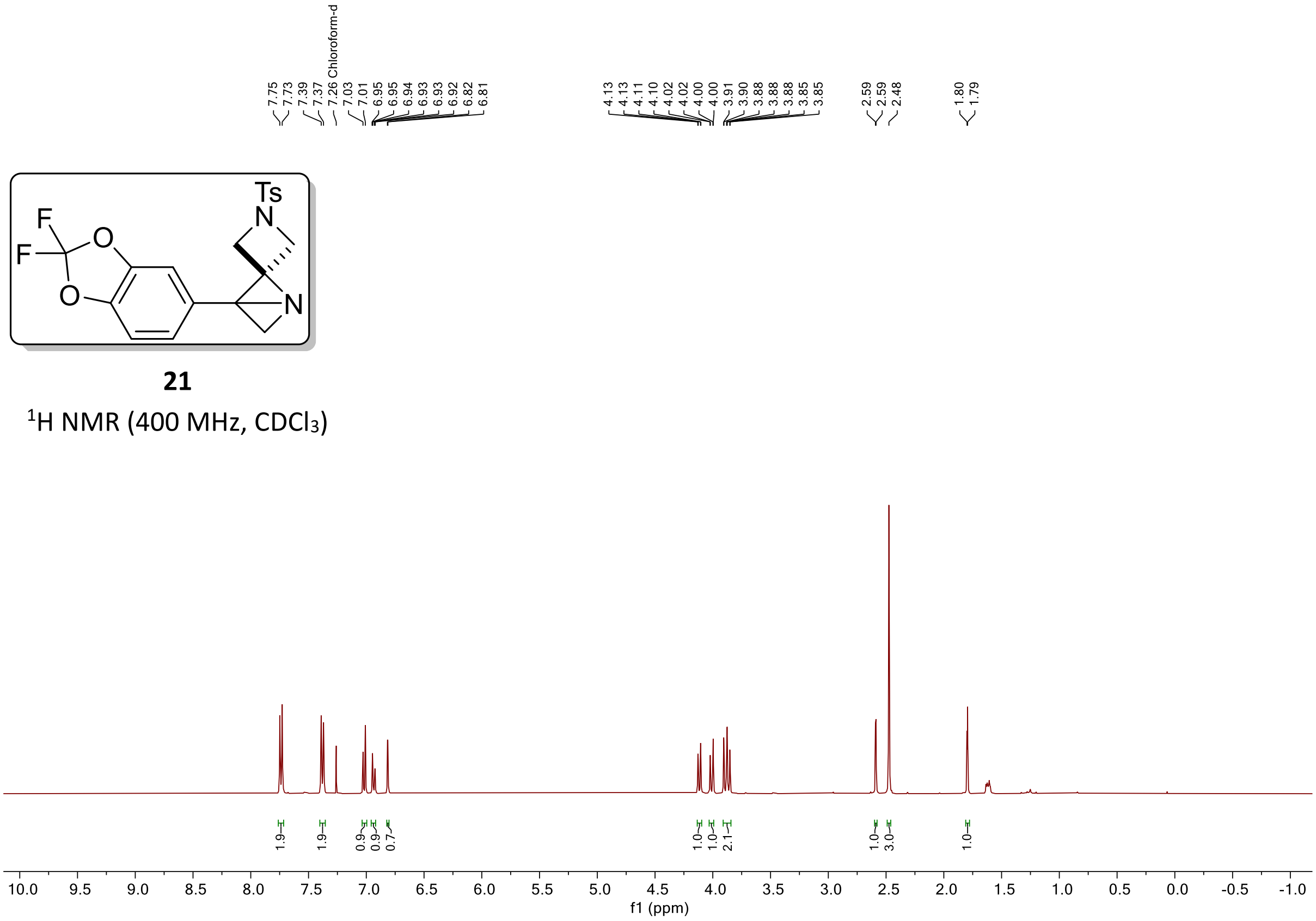


**21**

$^{1}$H NMR (400 MHz, $CDCl_3$)

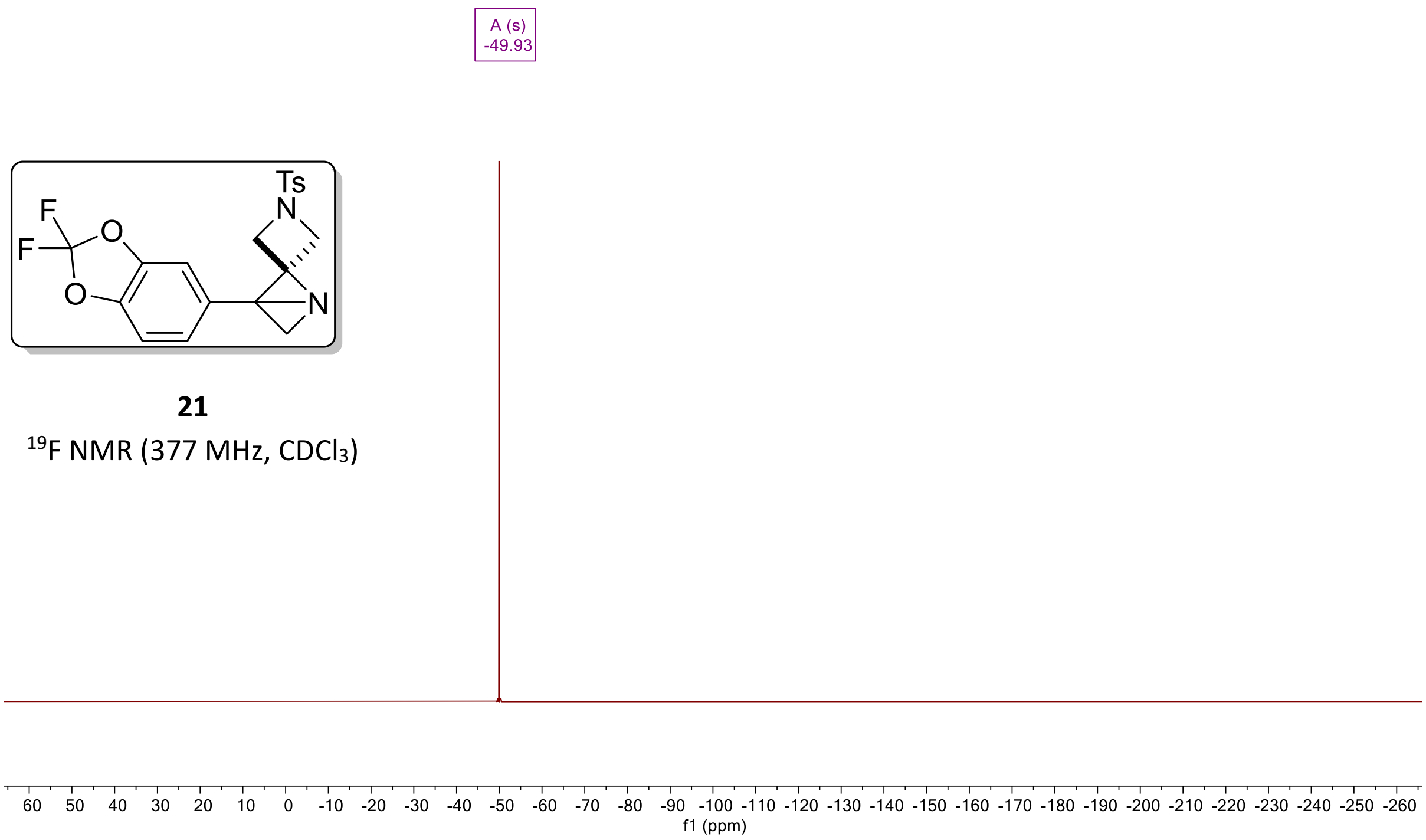


**21**

$^{19}$F NMR (377 MHz, $CDCl_3$)

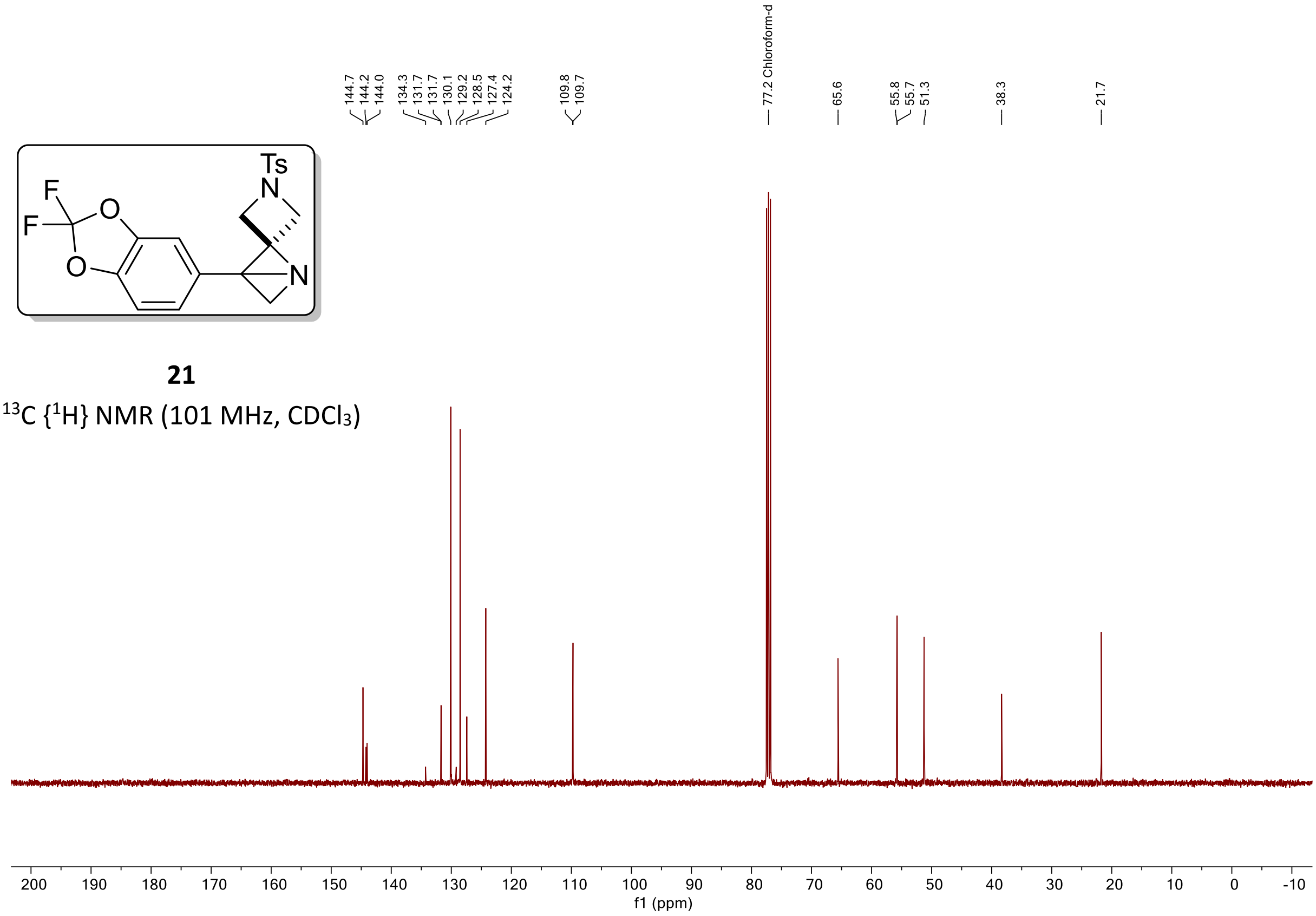


**21**

$^{13}C$ {$^{1}H$} NMR (101 MHz, $CDCl_3$)

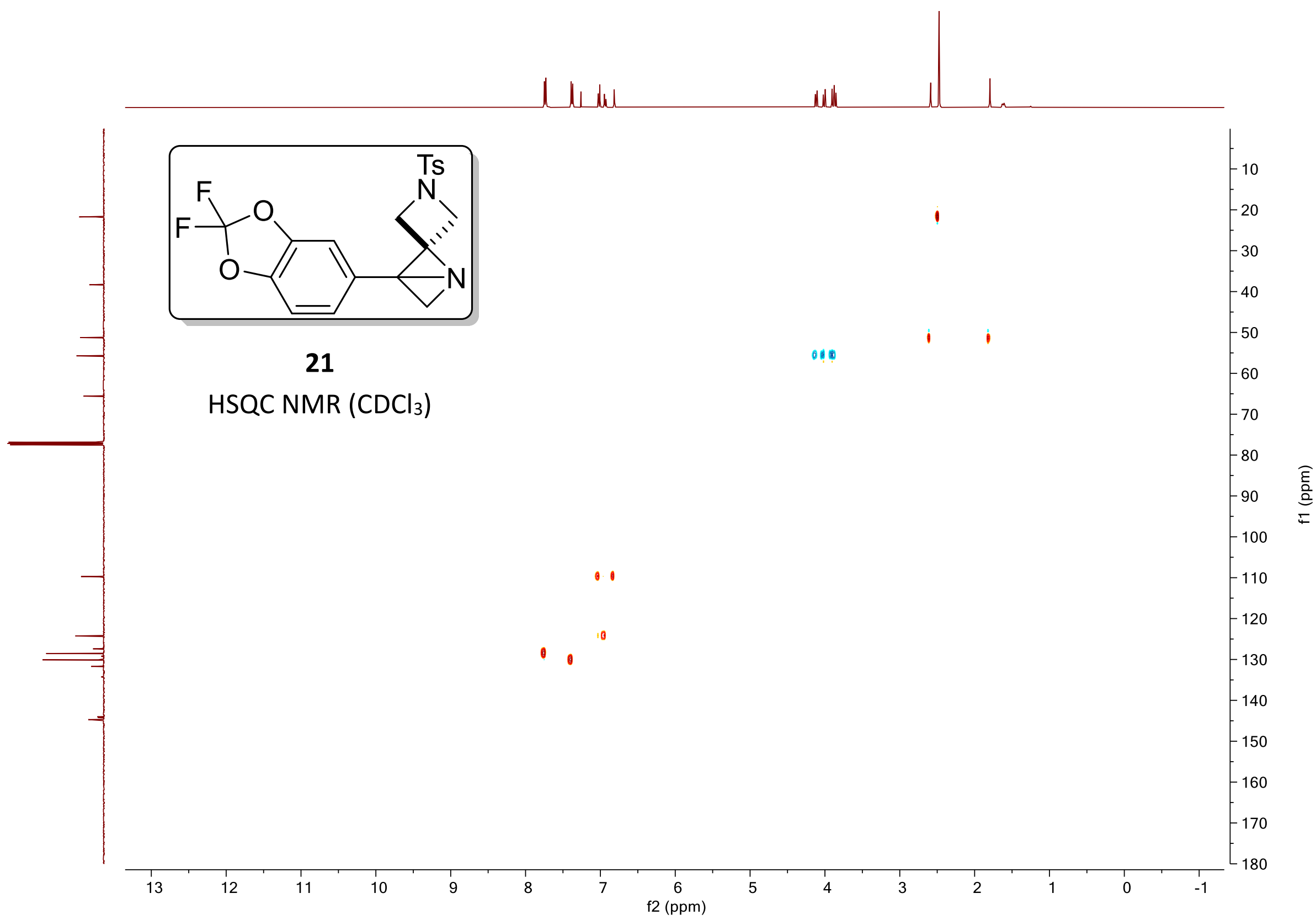


**21**

HSQC NMR ($CDCl_3$)

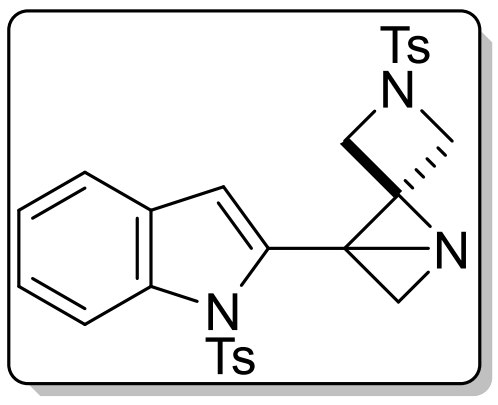


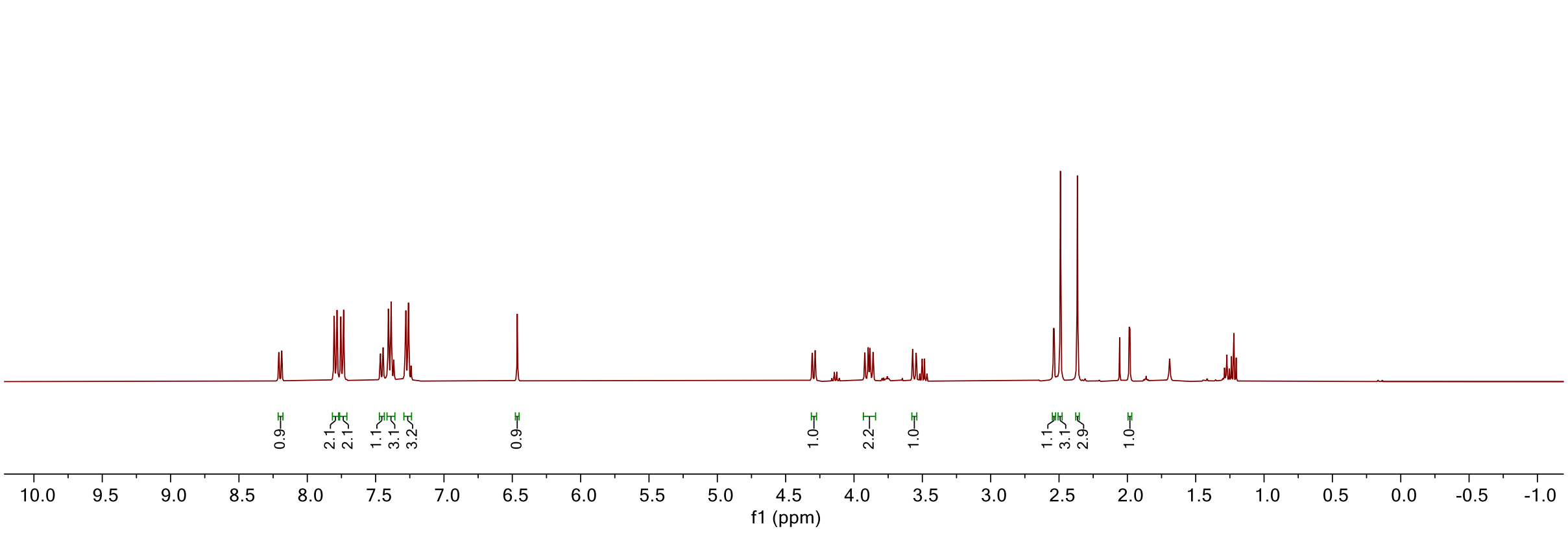


**22**

$^{1}$H NMR (400 MHz, CDCl$_3$)

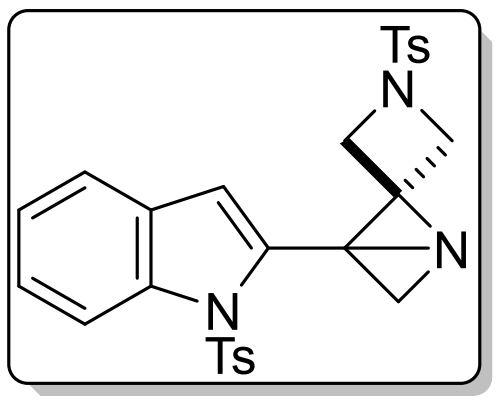


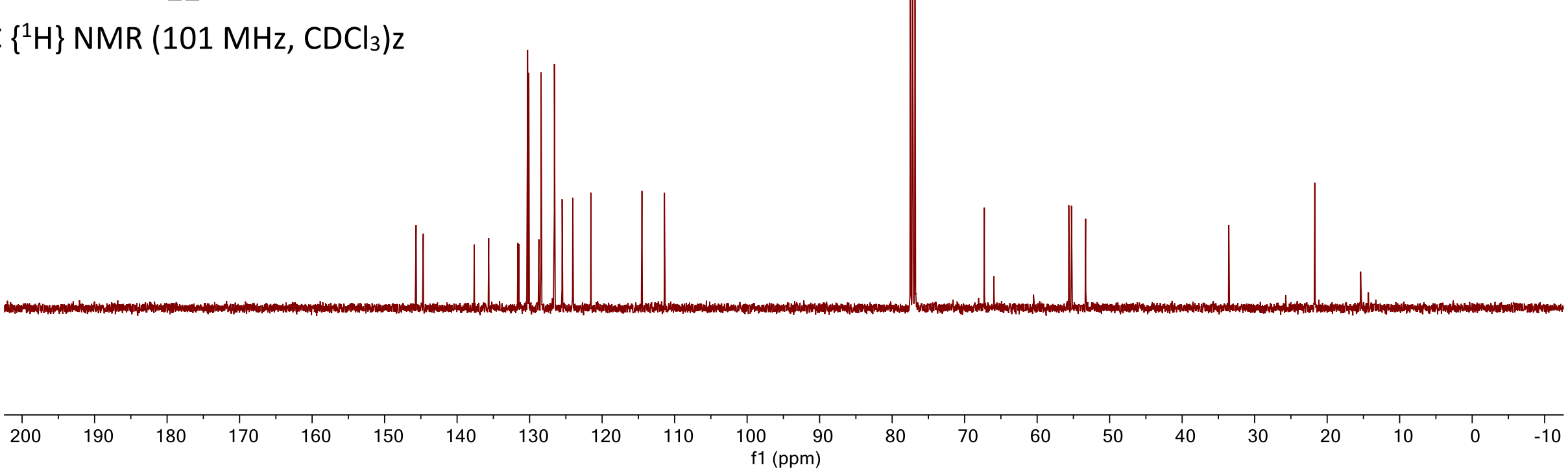


**22**

$^{13}$C {$^{1}$H} NMR (101 MHz, CDCl$_3$)z

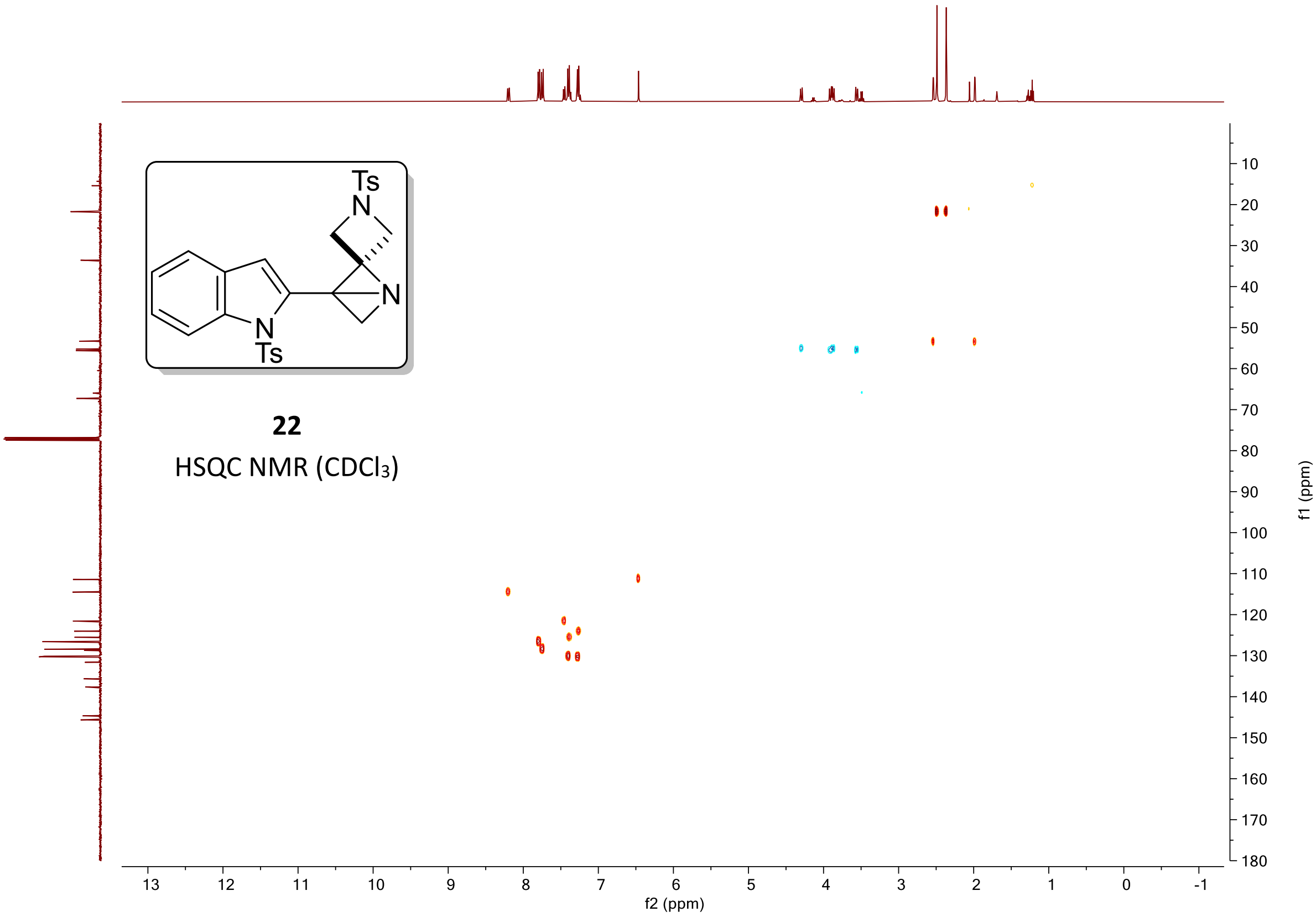
Ts
N
N
N
Ts
22
HSQC NMR (CDCl3)
f2 (ppm)
f1 (ppm)

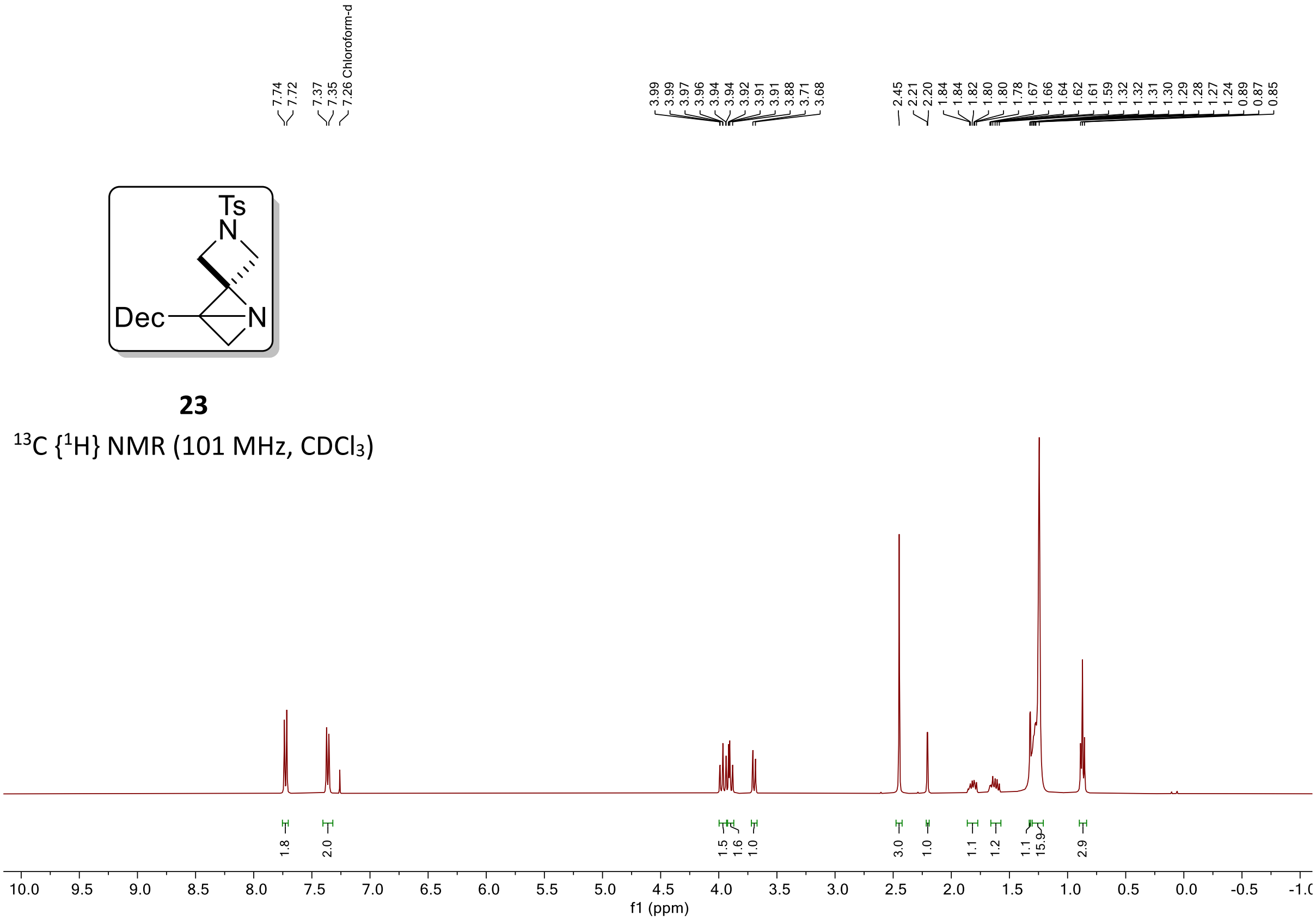

**23**

$^{13}C\ \{^1H\}$ NMR (101 MHz, $CDCl_3$)

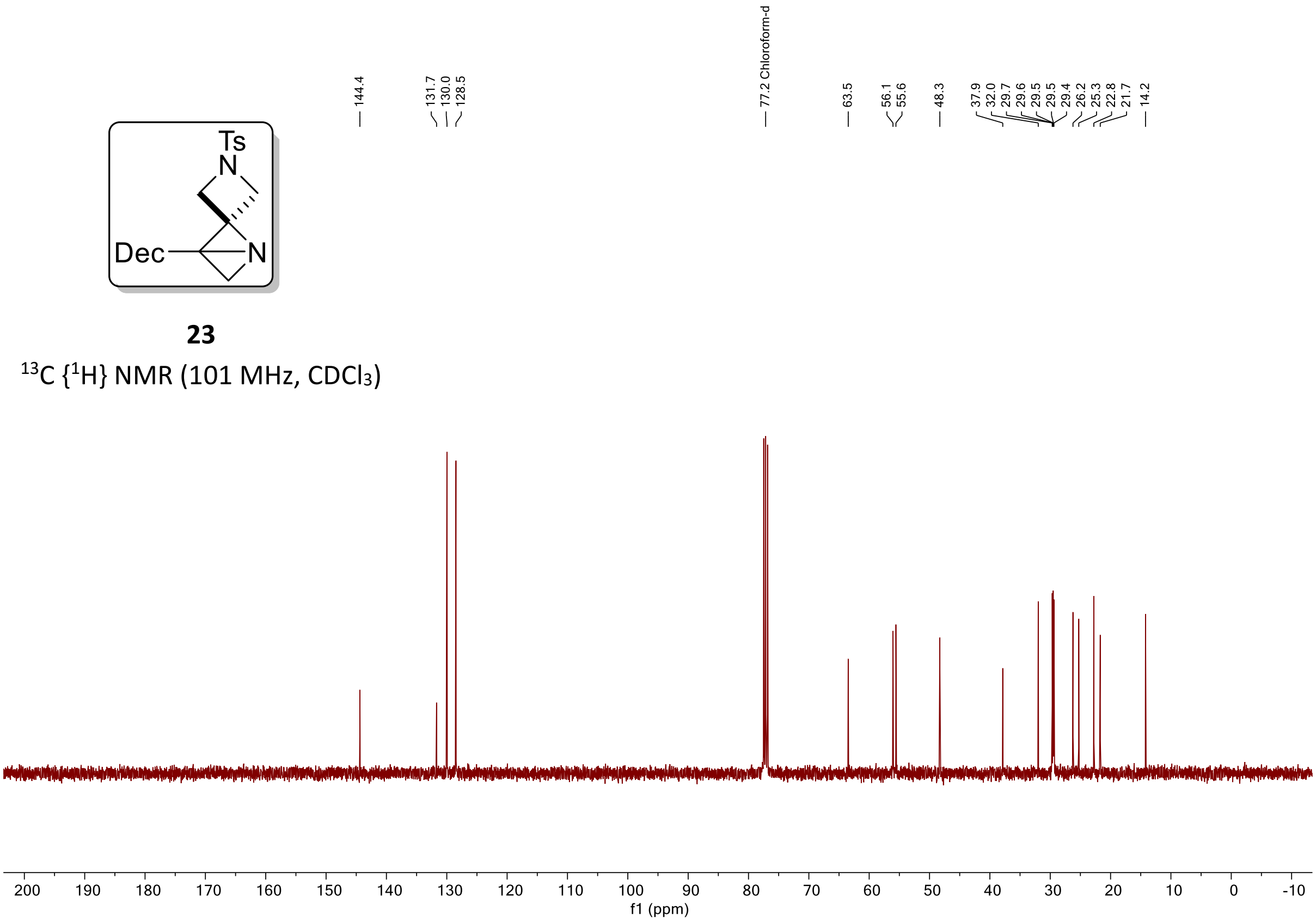

**23**

$^{13}C\ \{^1H\}$ NMR (101 MHz, $CDCl_3$)

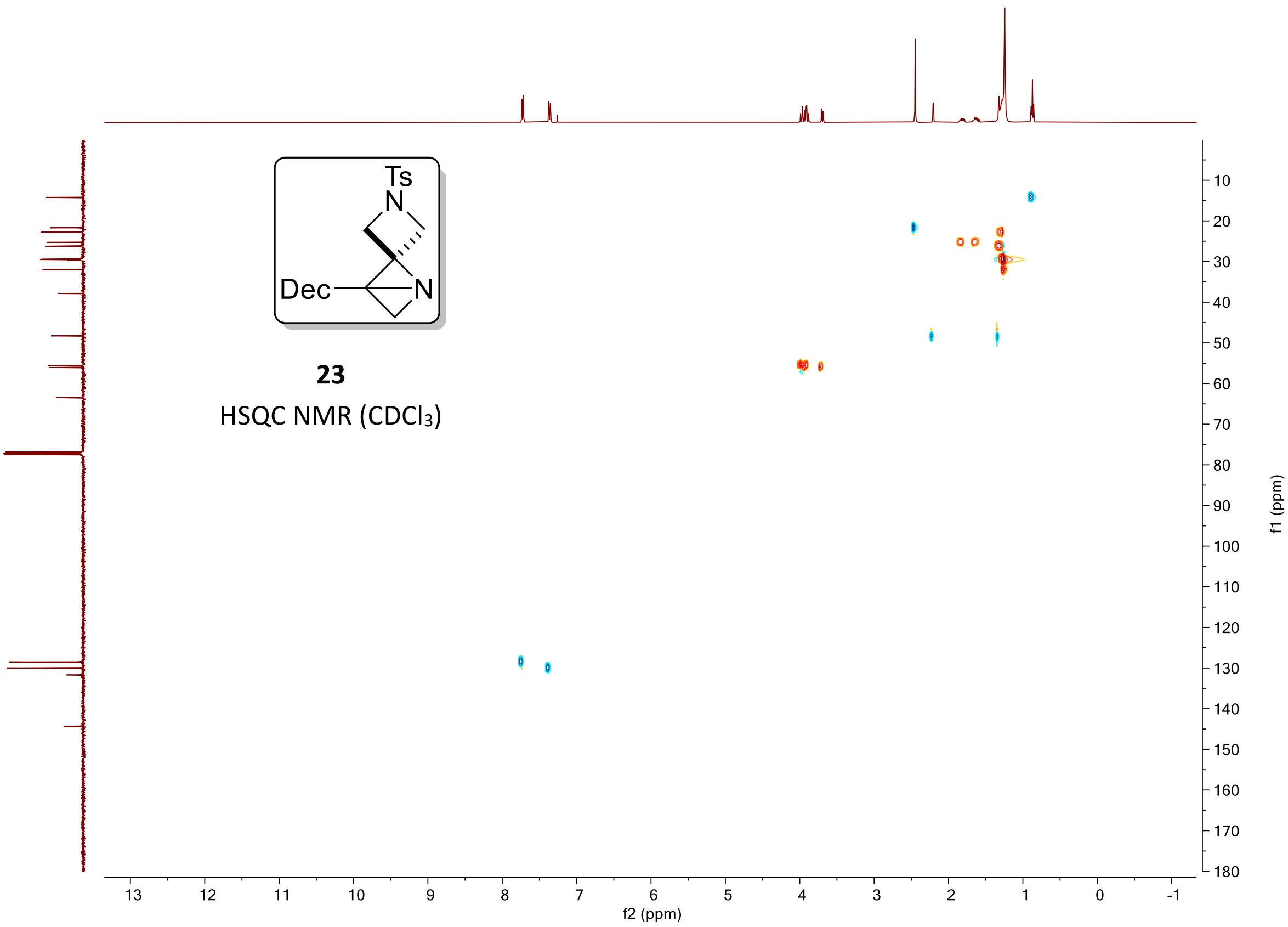

Ts
N
Dec
N
23
HSQC NMR (CDCl3)
f2 (ppm)
f1 (ppm)

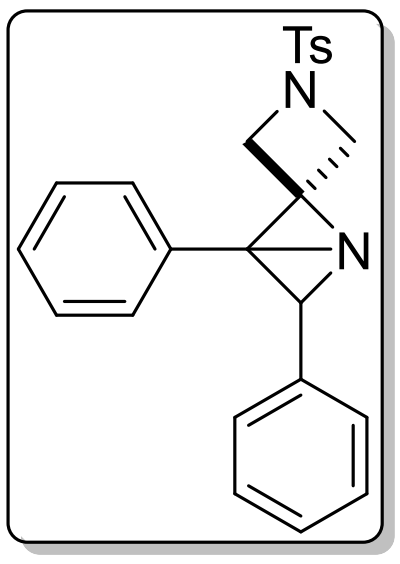

**24**

$^{1}H$ NMR (400 MHz, $CDCl_3$)

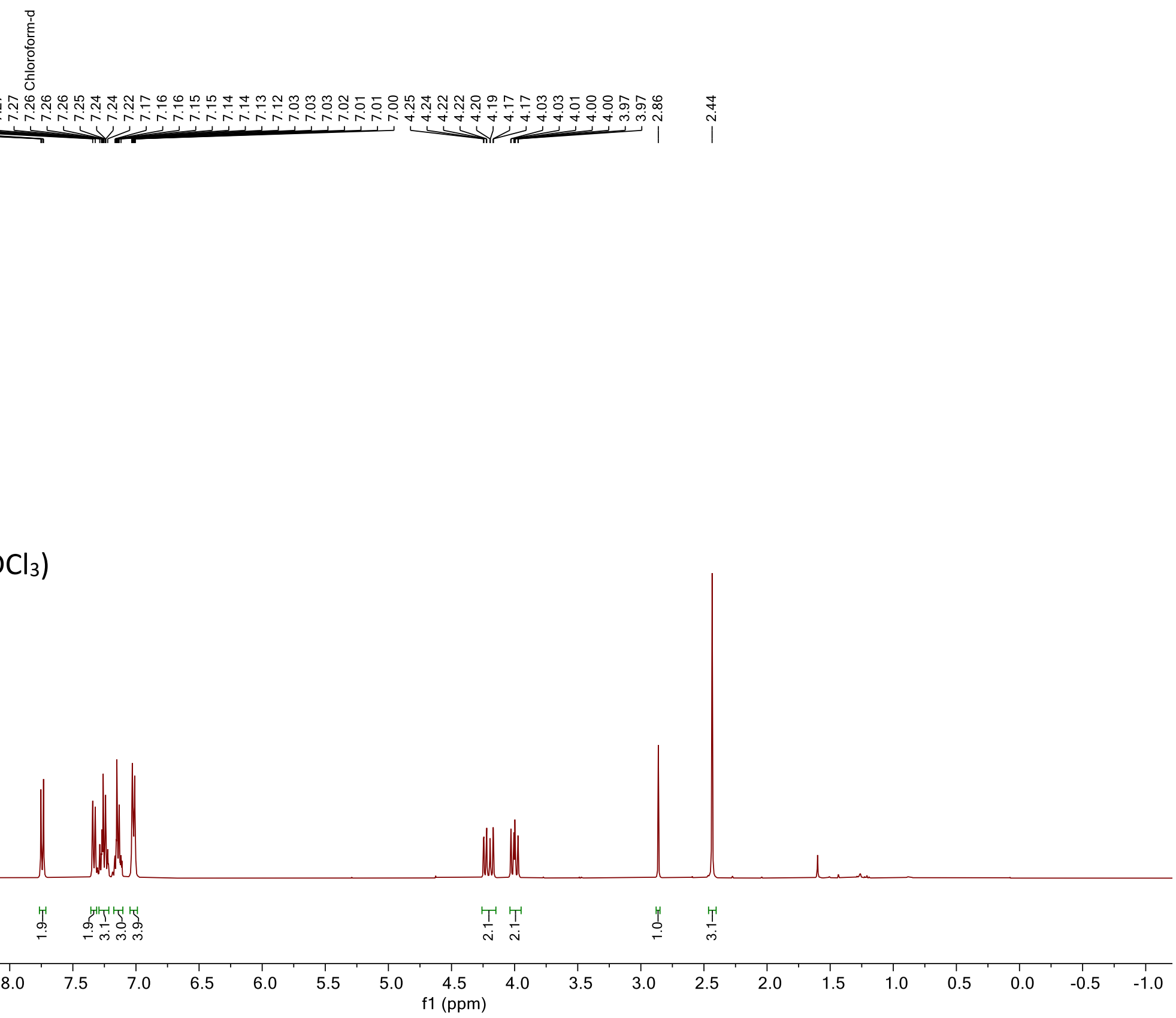

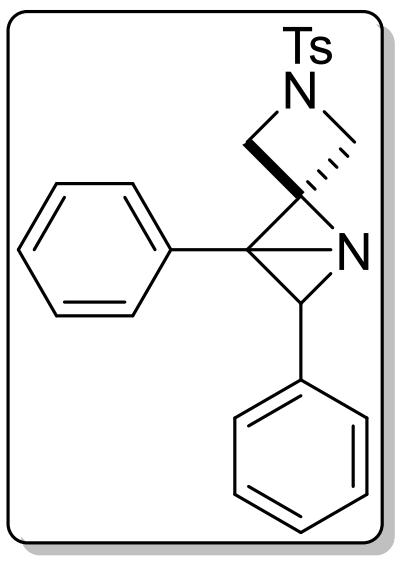

**24**

$^{13}C$ {$^{1}H$} NMR (101 MHz, $CDCl_3$)

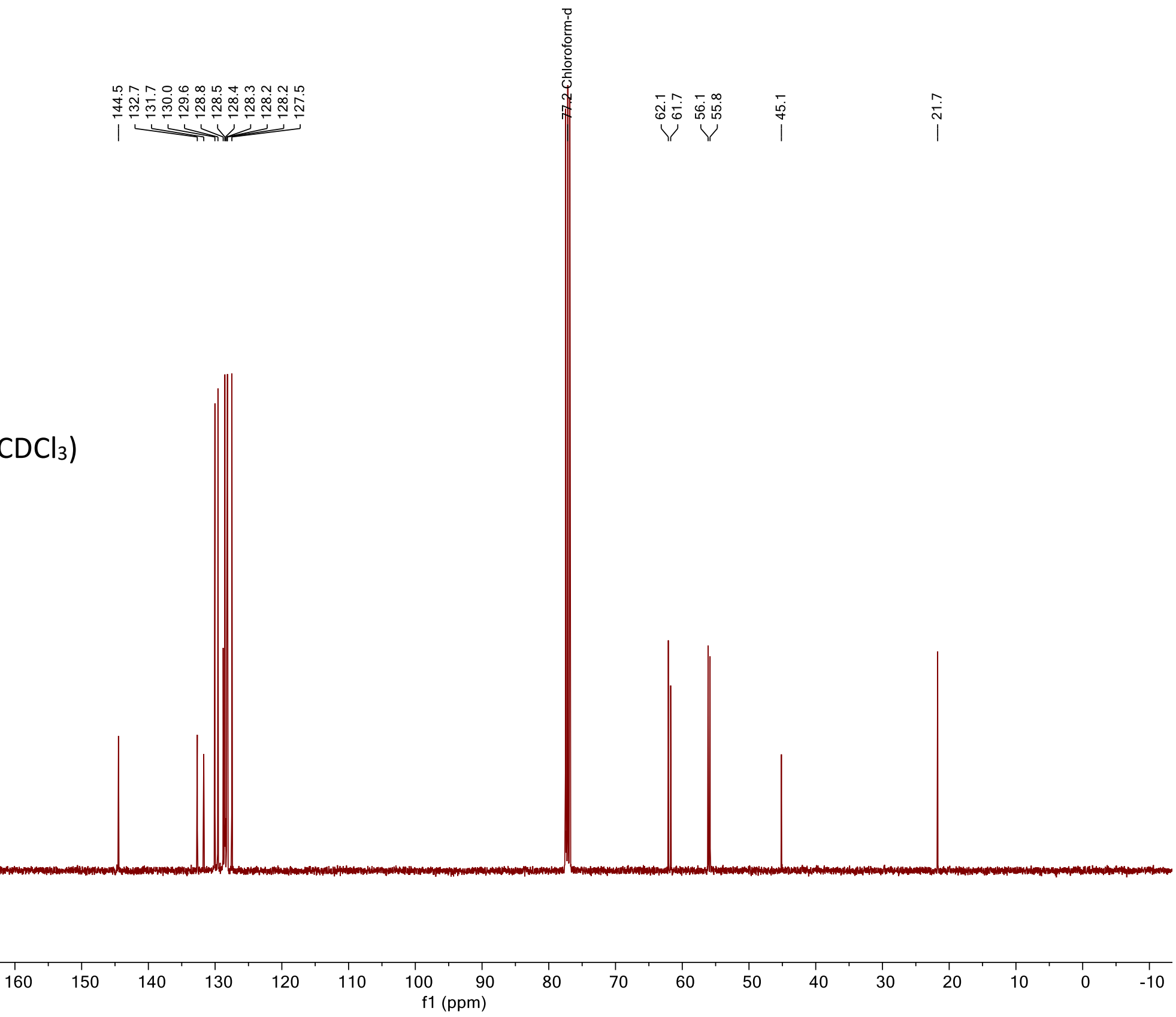

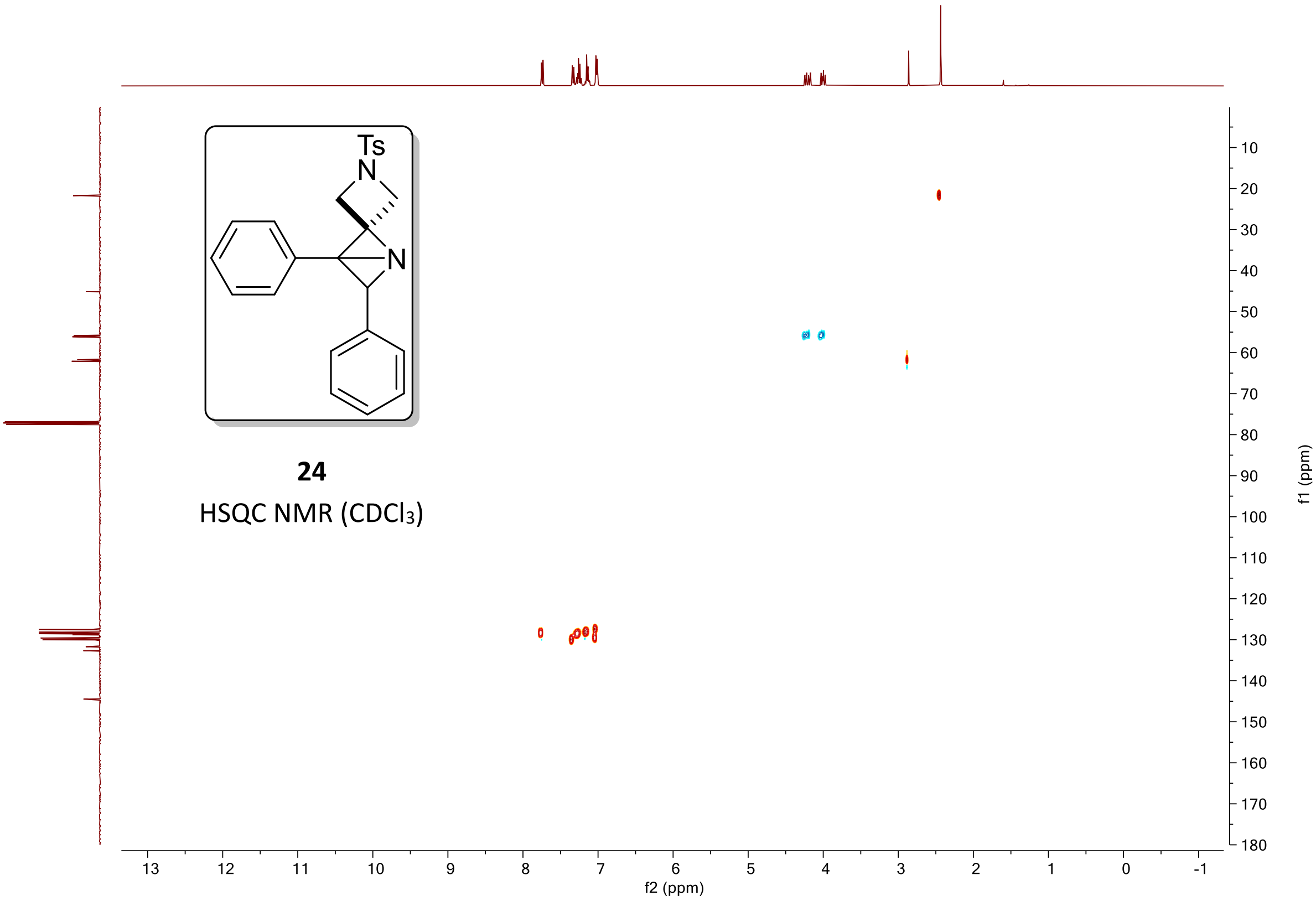


**24**

HSQC NMR ($CDCl_3$)

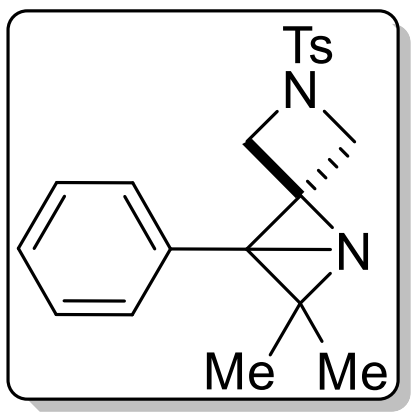


**25**

$^{1}H$ NMR (400 MHz, $CDCl_3$)

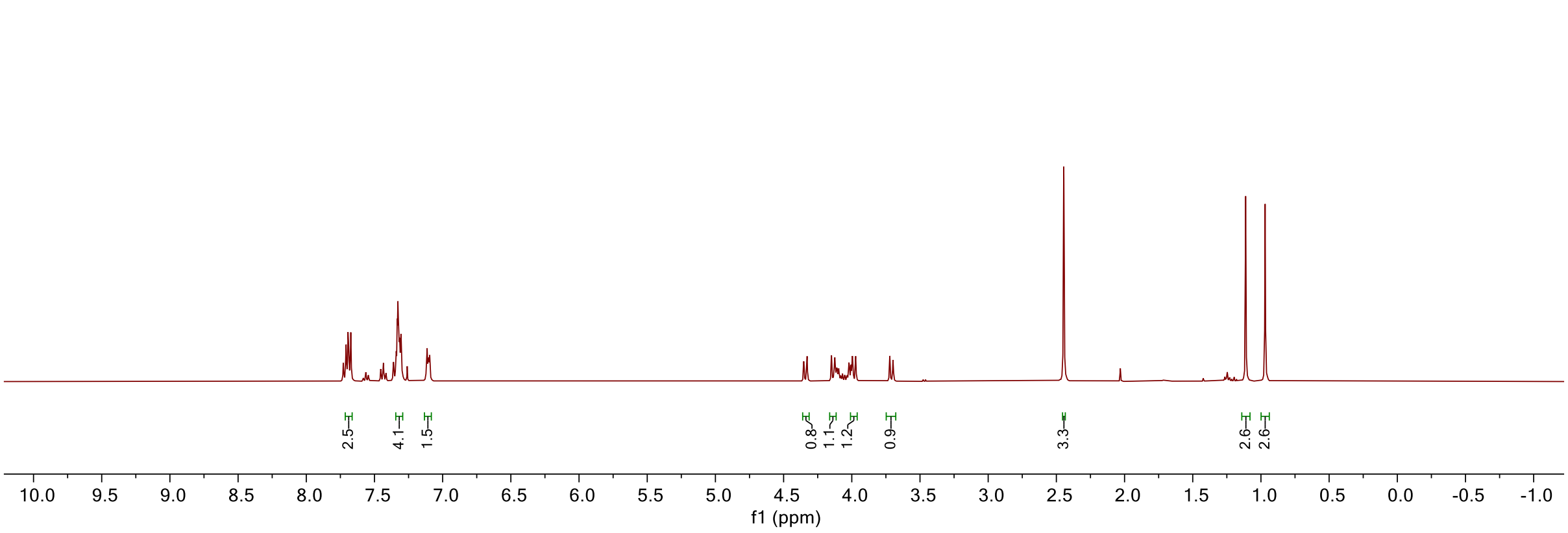


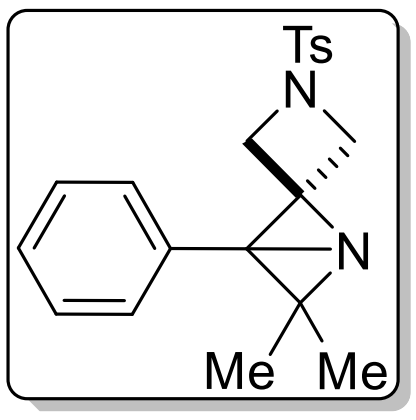


**25**

$^{13}C$ {$^{1}H$} NMR (101 MHz, $CDCl_3$)

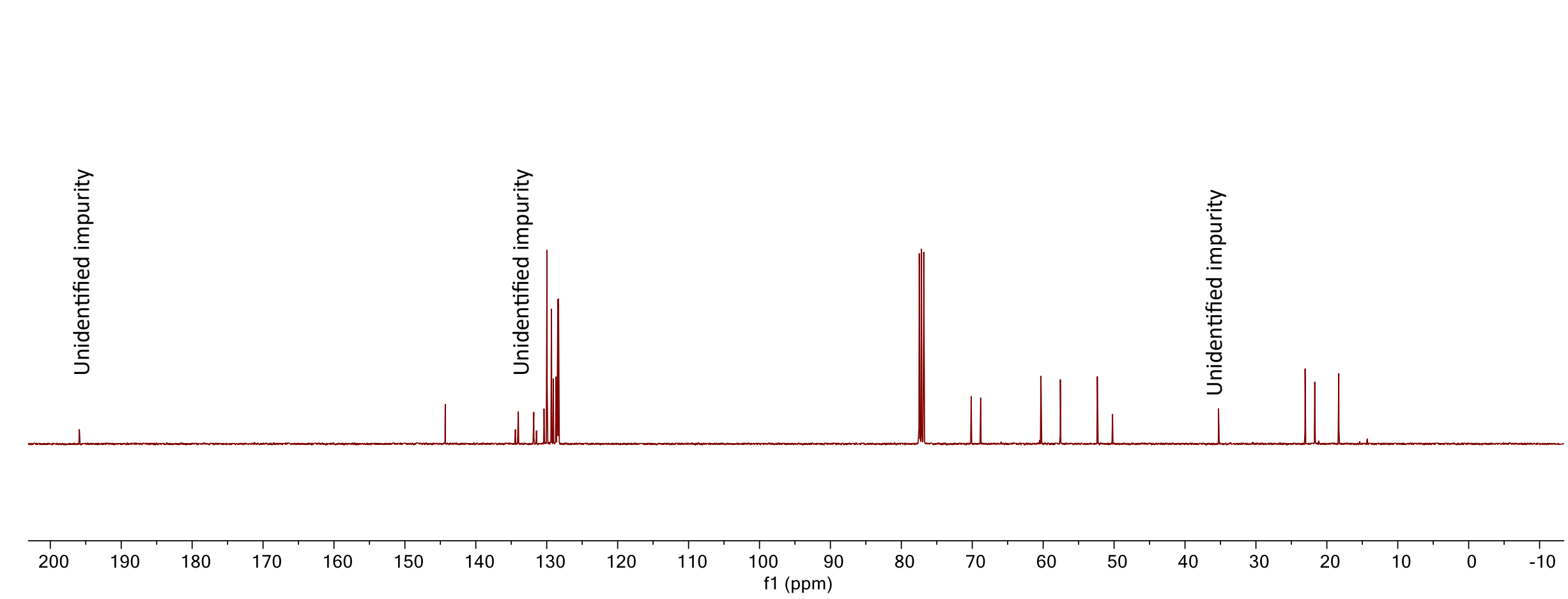

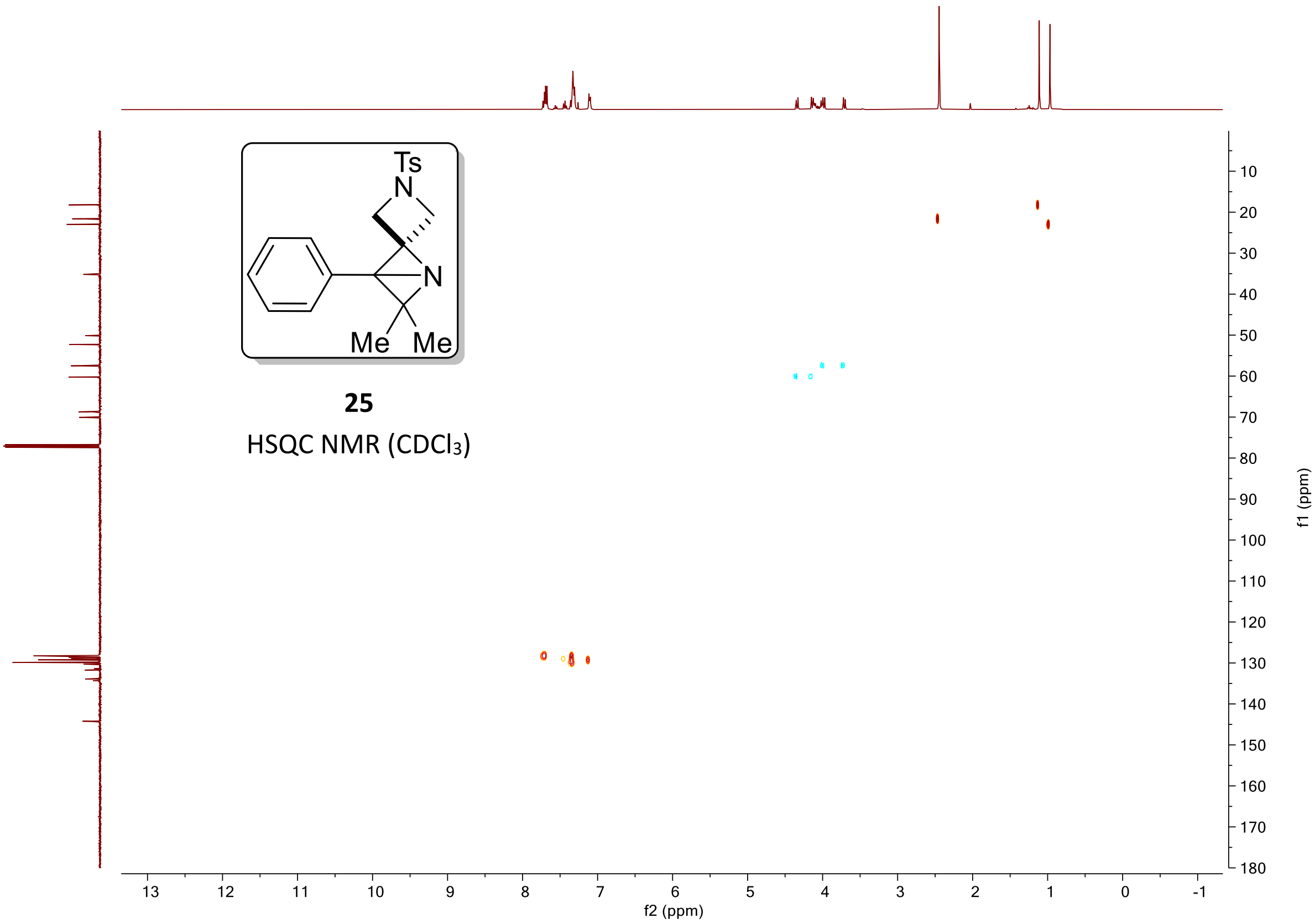
Ts
N
N
Me
Me
25
HSQC NMR (CDCl3)
f1 (ppm)
f2 (ppm)

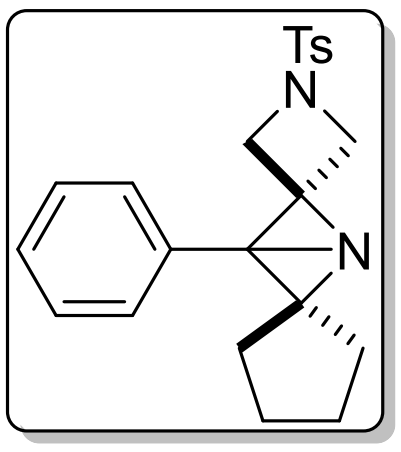

**26**

$^1$H NMR (400 MHz, CDCl$_3$)

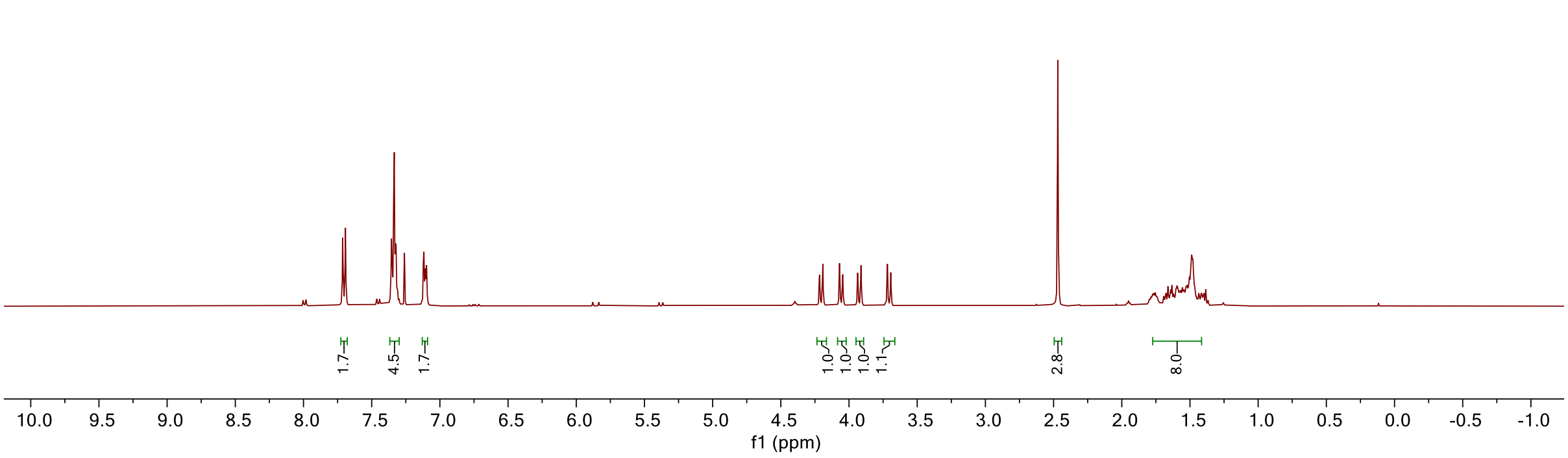


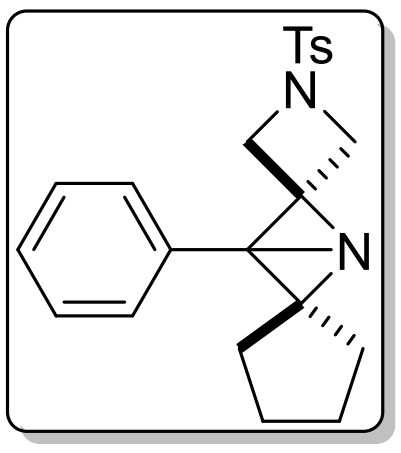

**26**

$^{13}$C {$^1$H} NMR (101 MHz, CDCl$_3$)

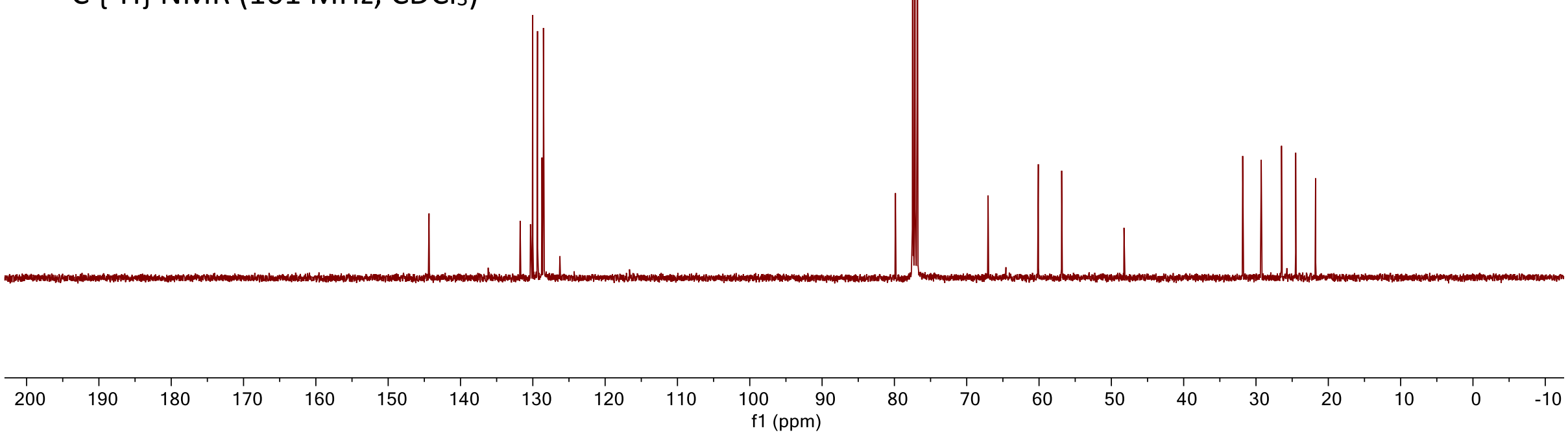

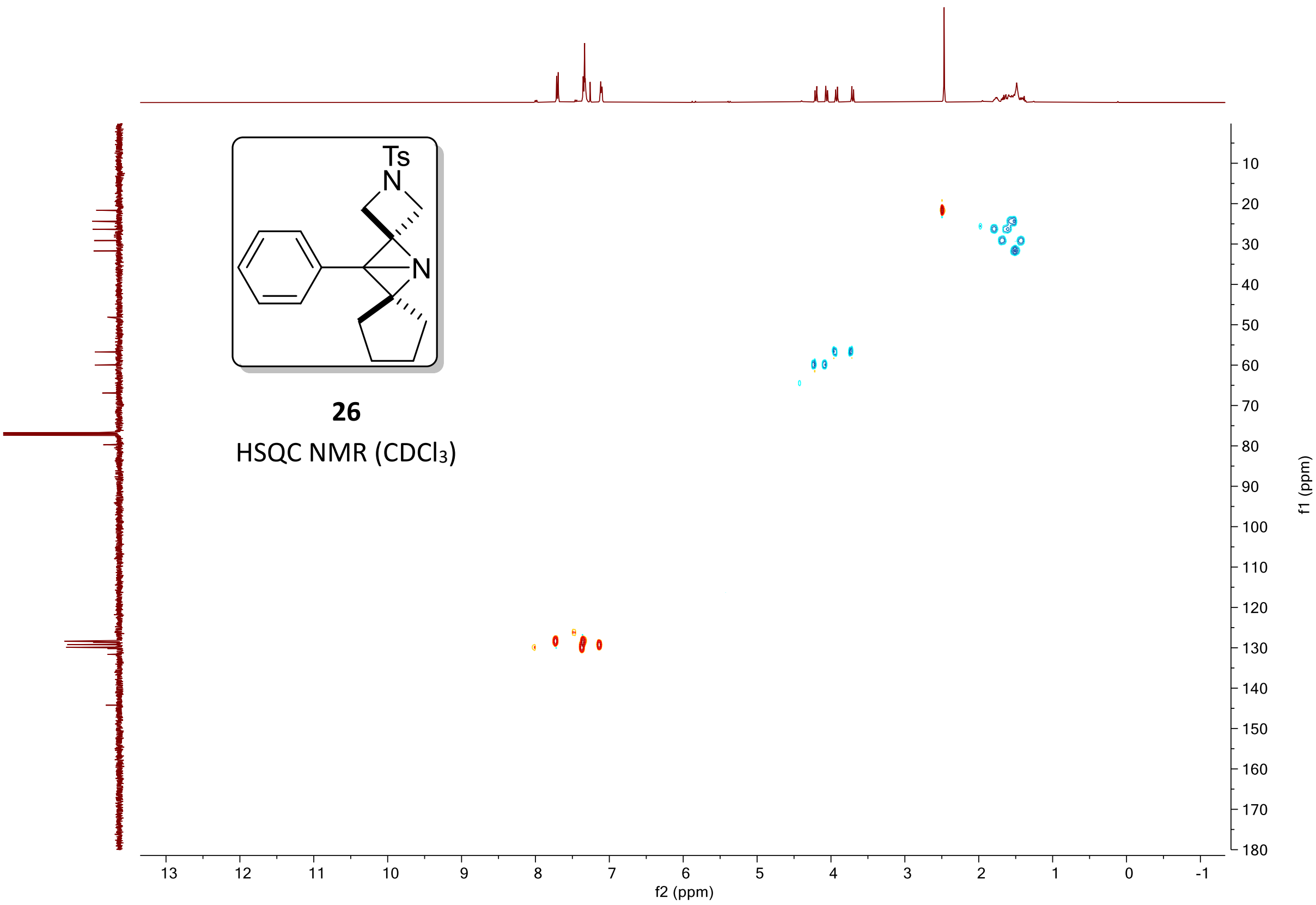


**26**

HSQC NMR ($CDCl_3$)

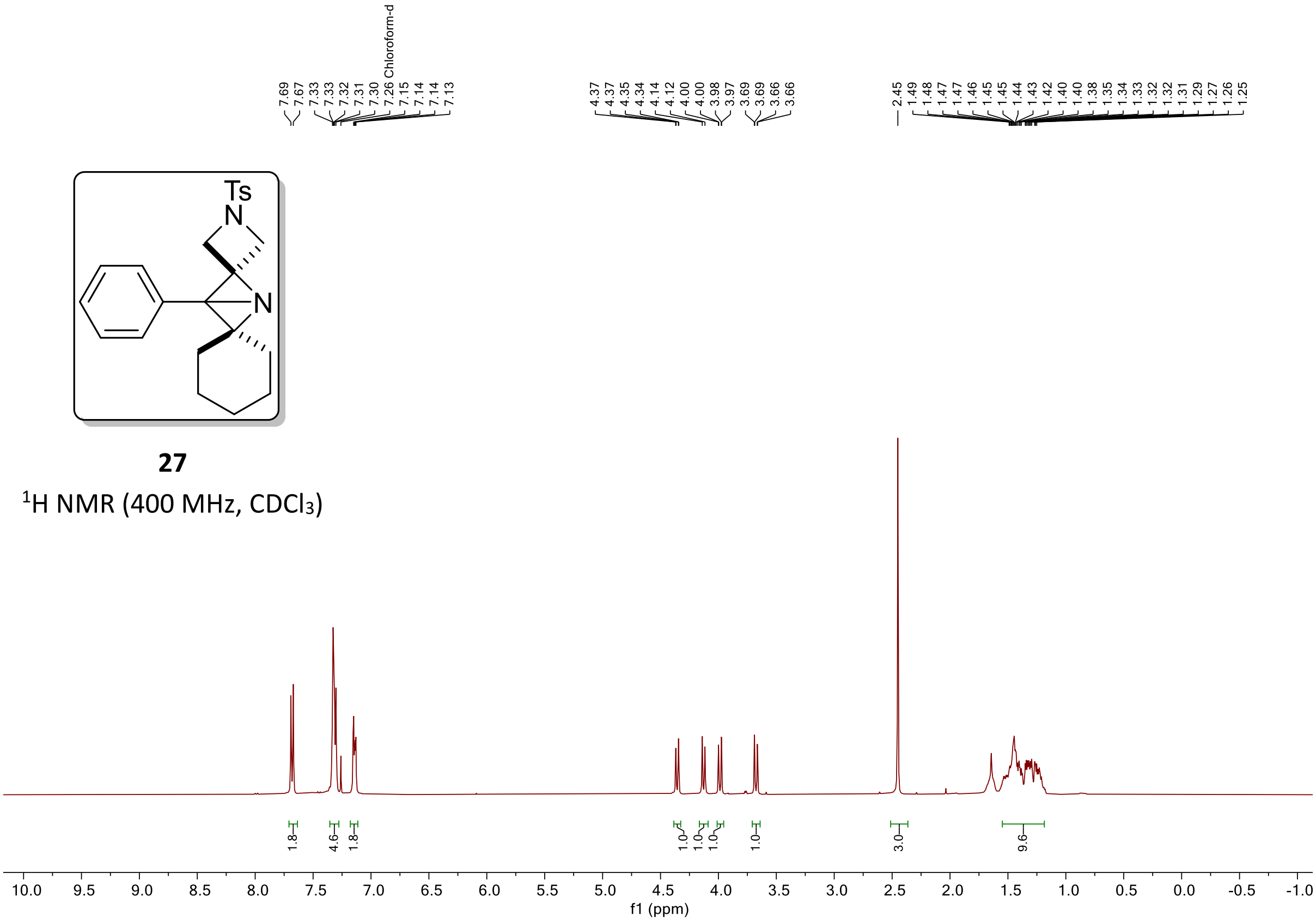


**27**

$^1$H NMR (400 MHz, $CDCl_3$)

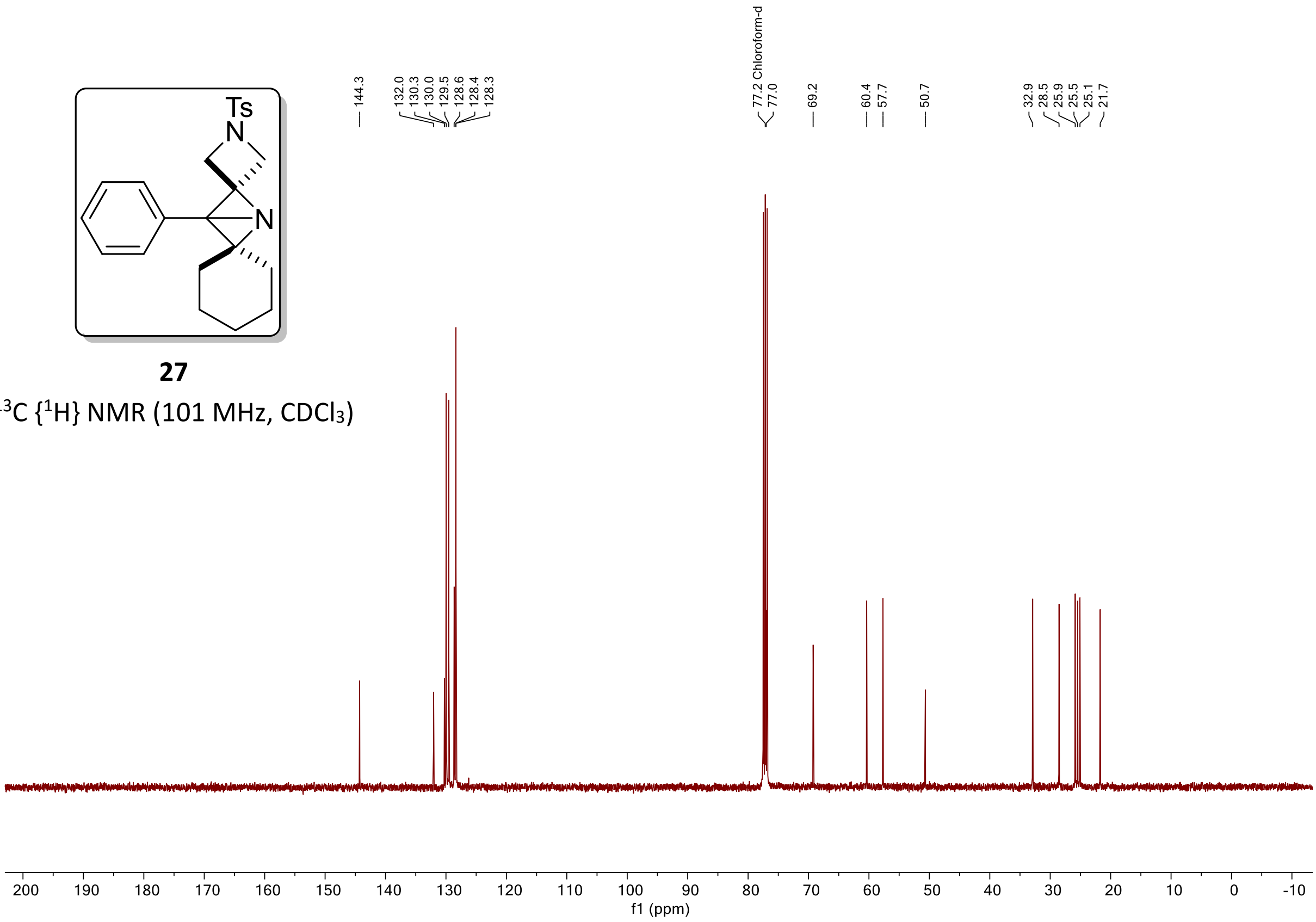


**27**

$^{13}$C {$^1$H} NMR (101 MHz, $CDCl_3$)

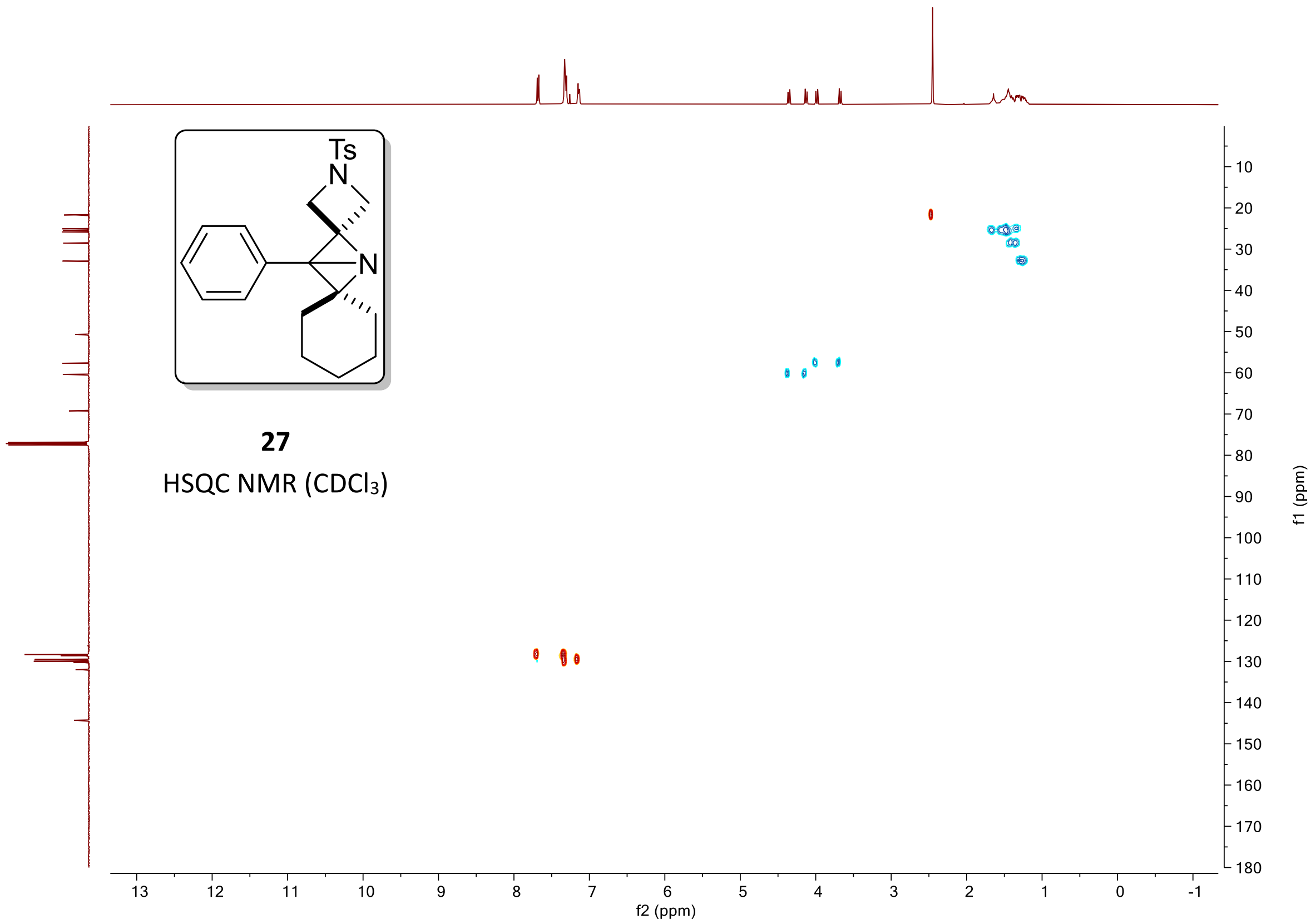


**27**

HSQC NMR ($CDCl_3$)

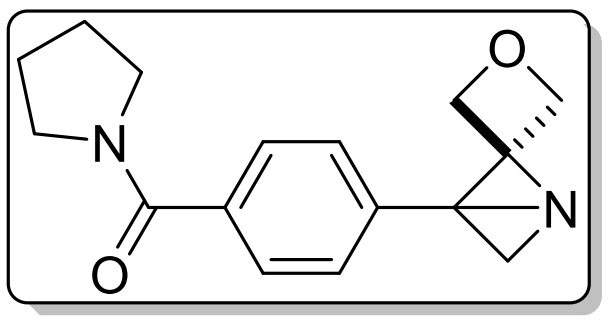

**28**

$^{1}$H NMR (400 MHz, $CDCl_3$)

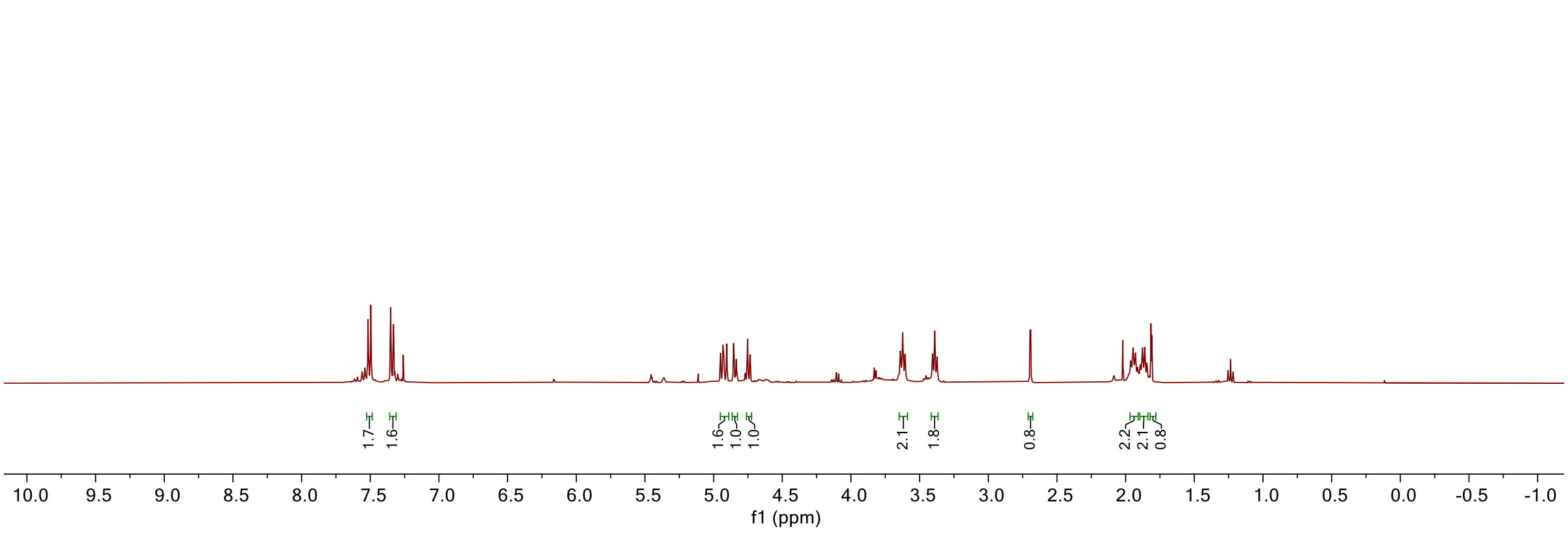


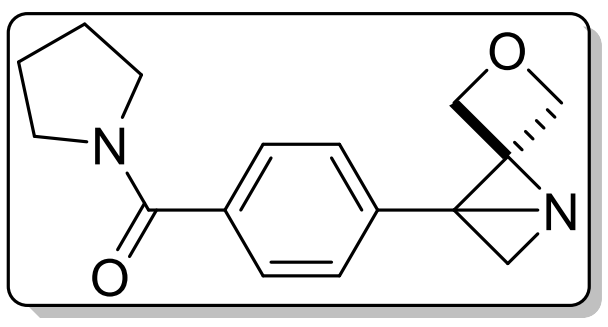

**28**

$^{13}$C {$^{1}$H} NMR (101 MHz, $CDCl_3$)

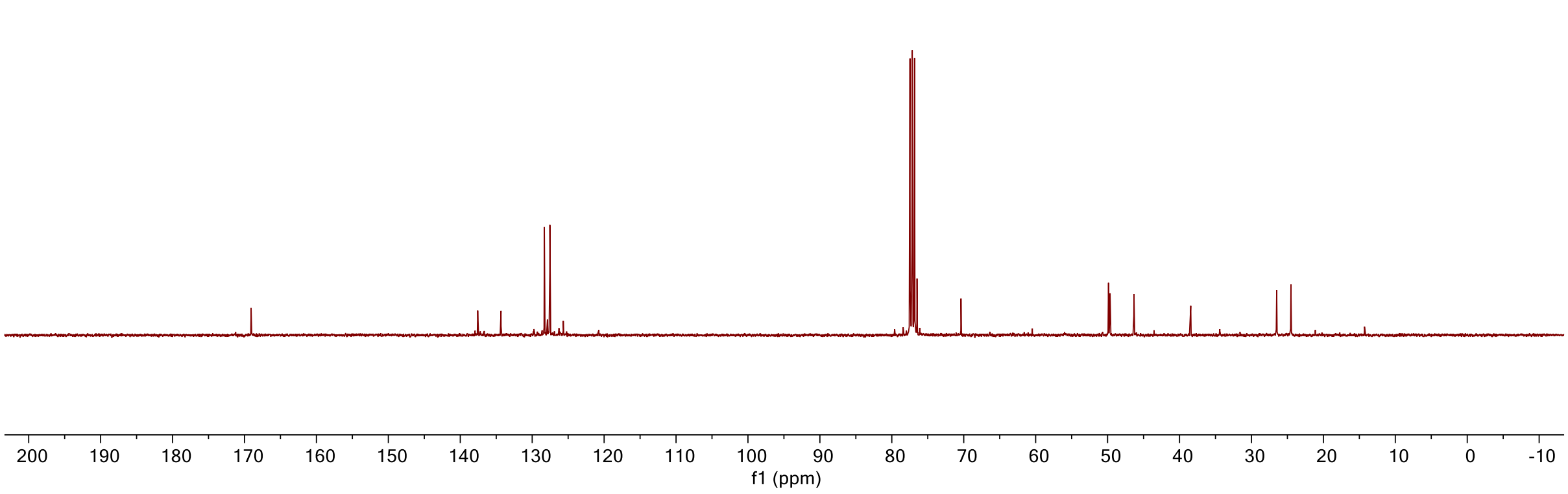

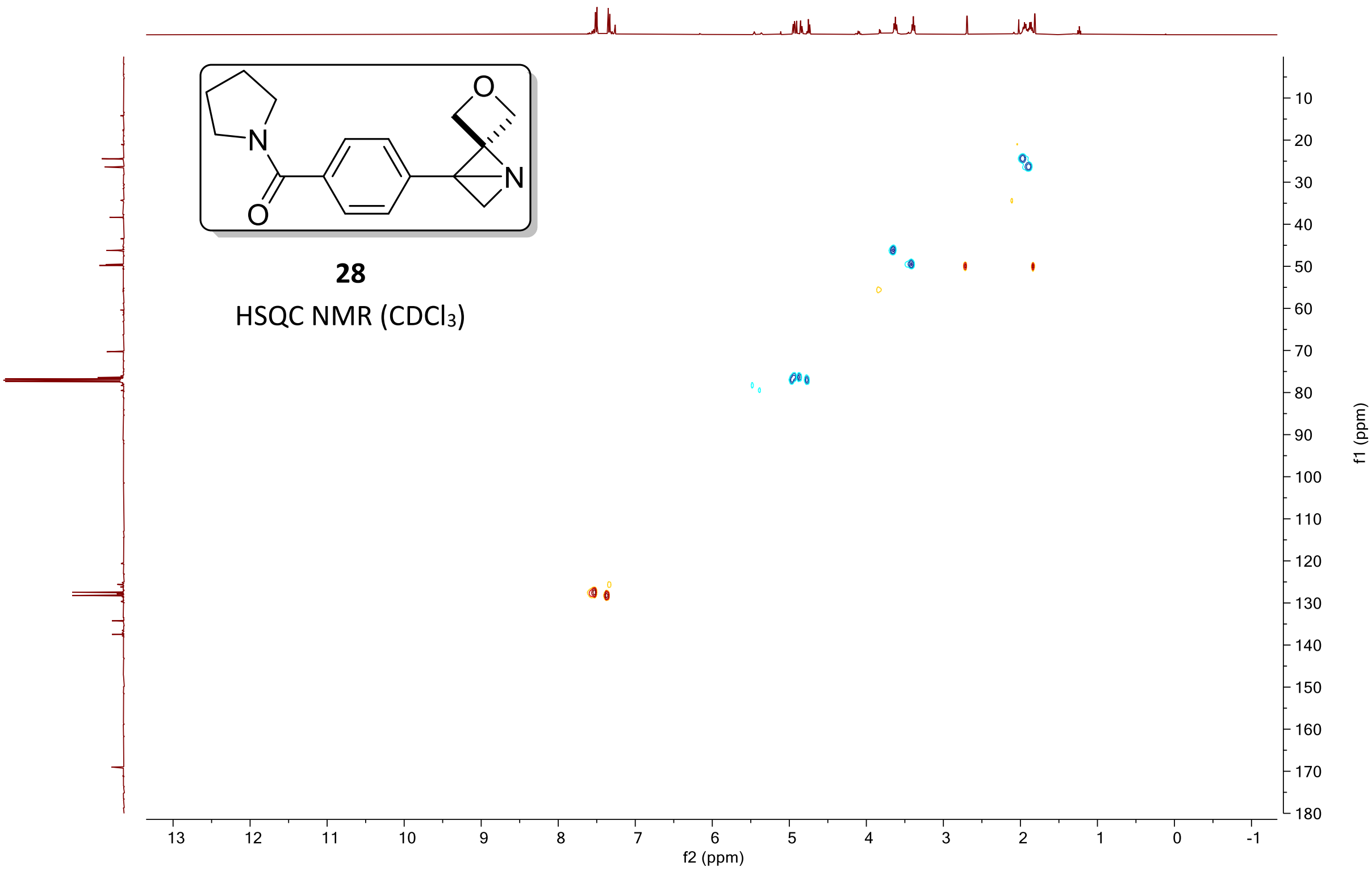


**28**

HSQC NMR ($CDCl_3$)

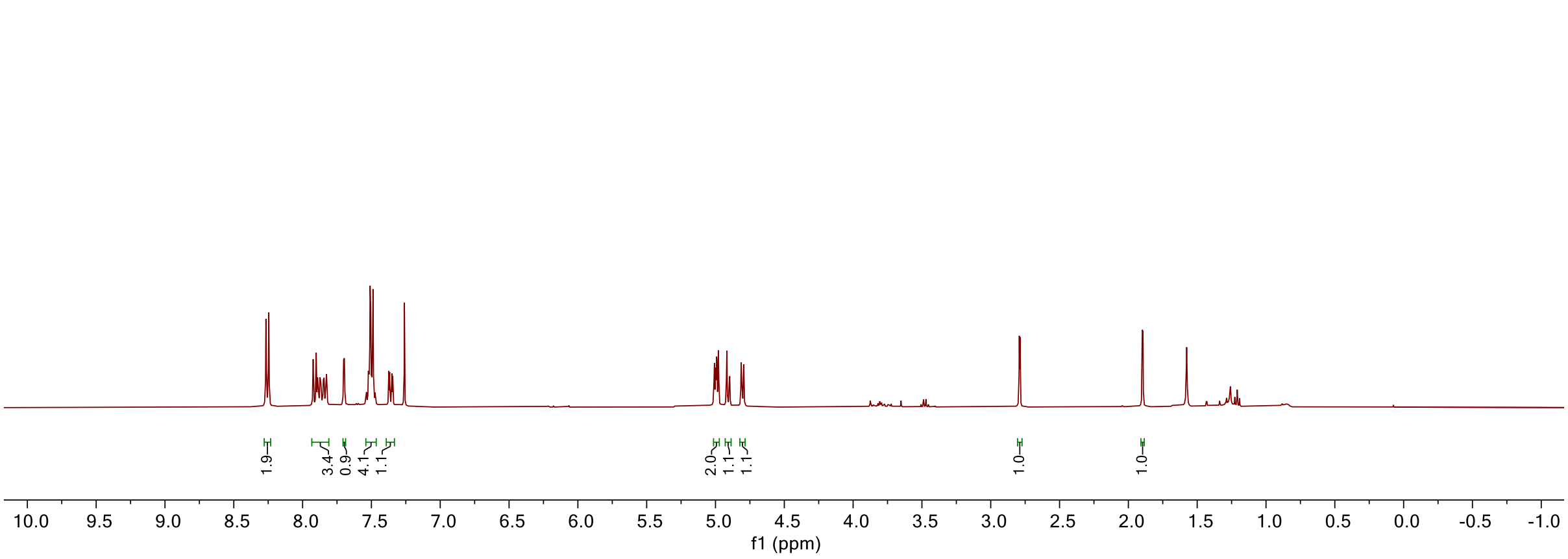


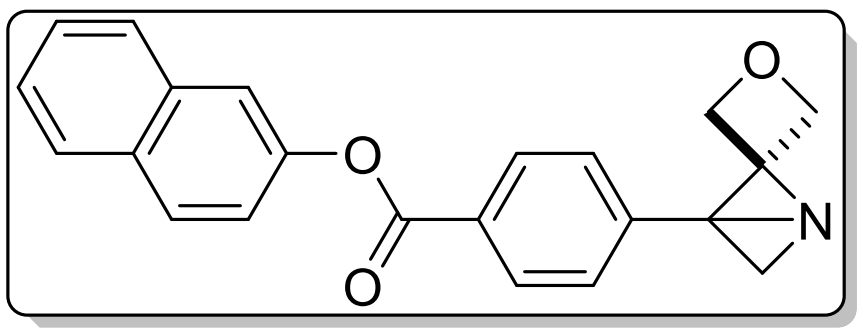


**29**

$^{1}H$ NMR (400 MHz, $CDCl_3$)

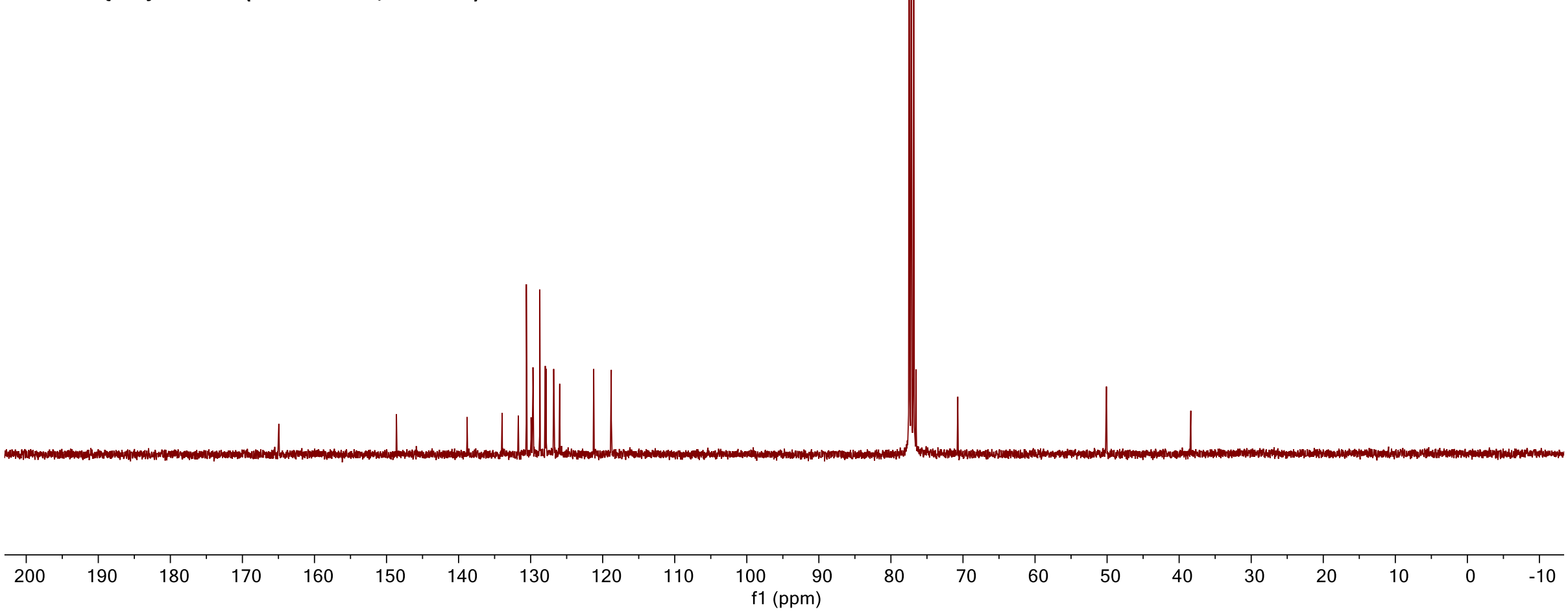


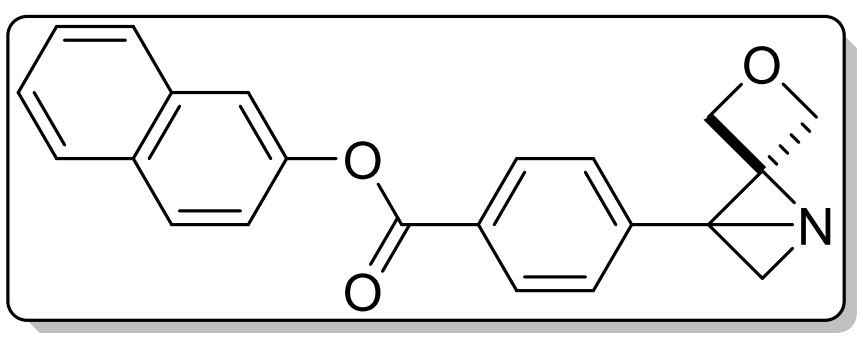


**29**

$^{13}C$ {$^{1}H$} NMR (101 MHz, $CDCl_3$)

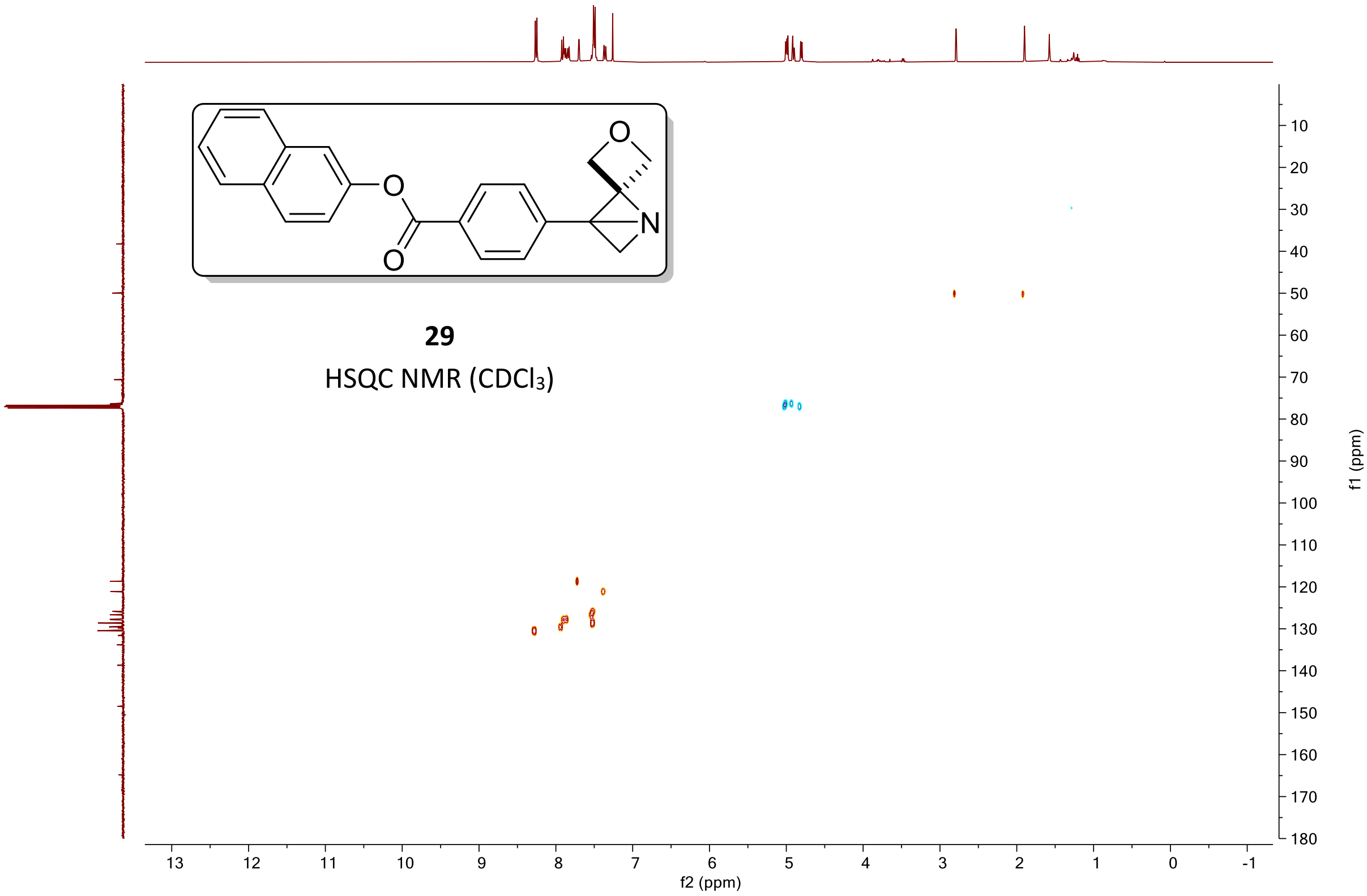


**29**

HSQC NMR ($CDCl_3$)

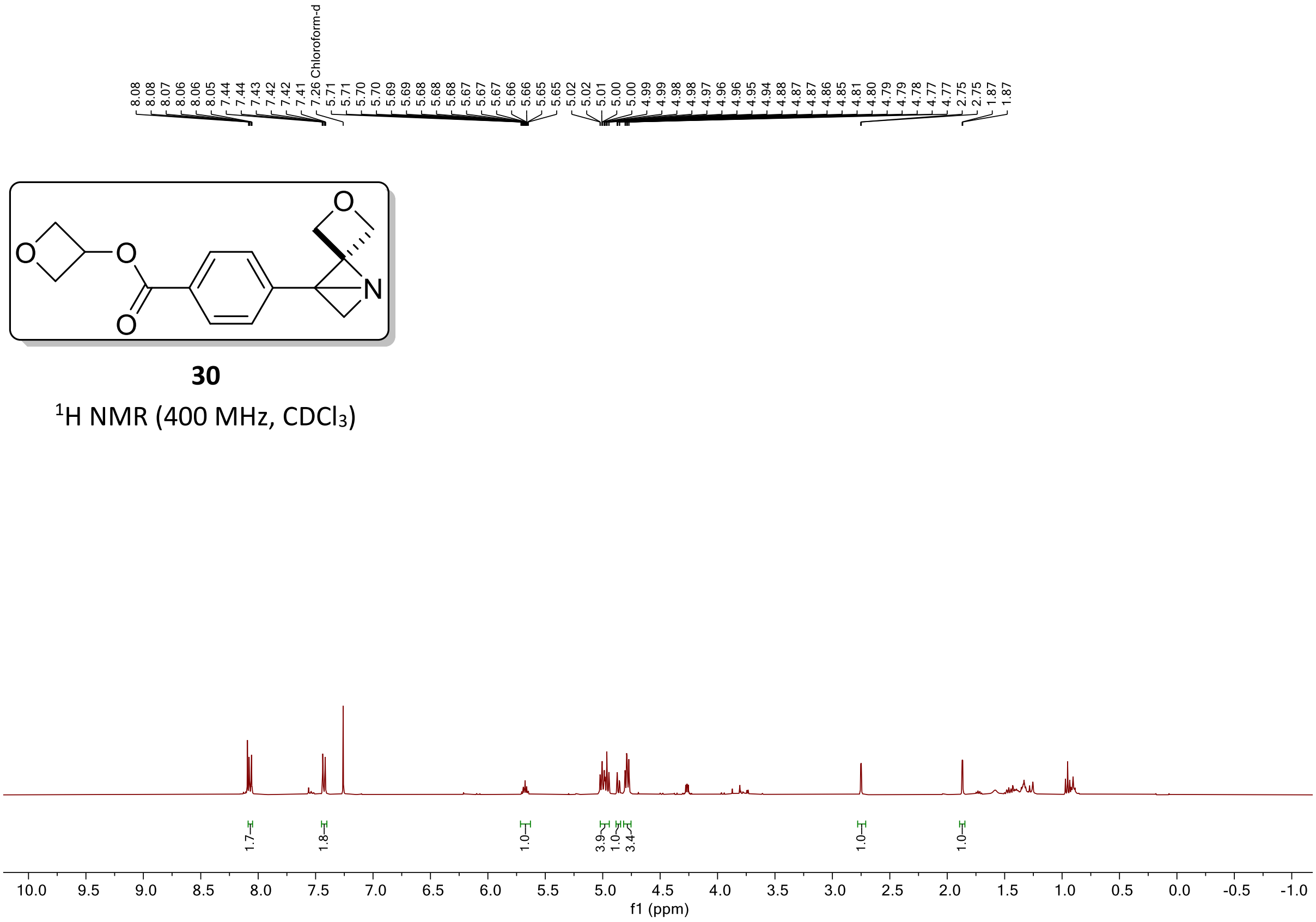

**30**

$^1$H NMR (400 MHz, $CDCl_3$)

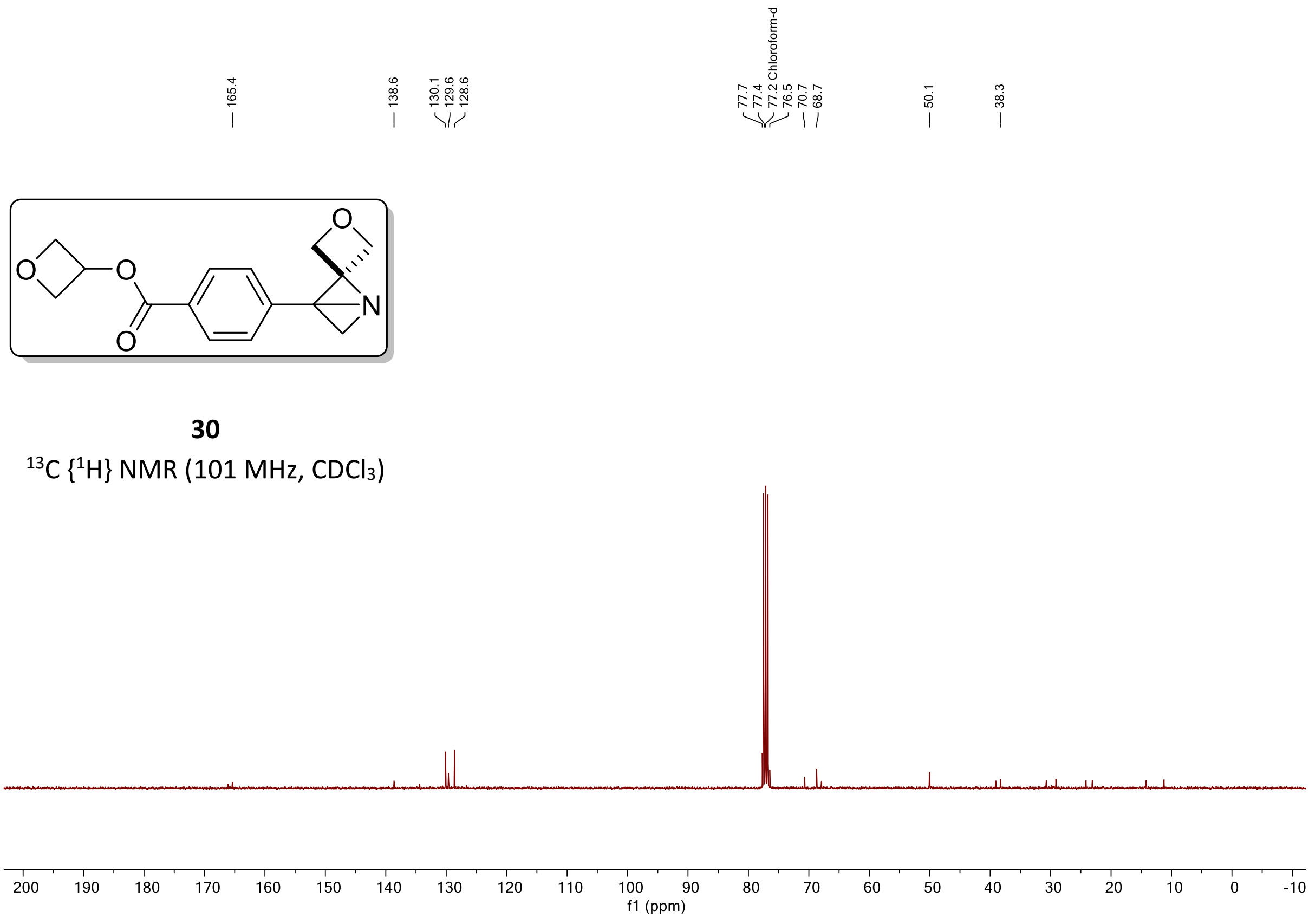

**30**

$^{13}$C {$^1$H} NMR (101 MHz, $CDCl_3$)

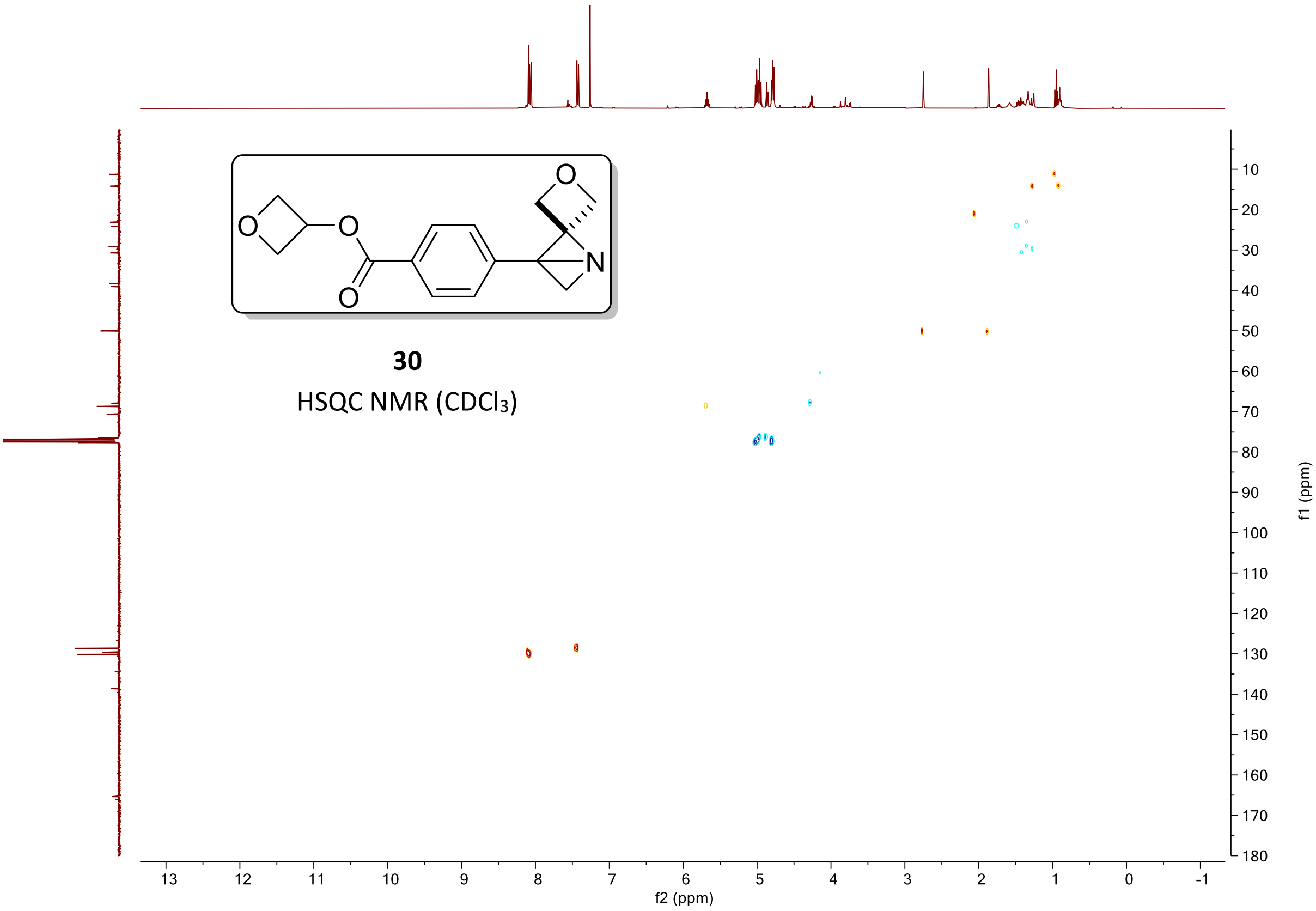

O
O
O
O
O
N
30
HSQC NMR ($CDCl_3$)
f2 (ppm)
f1 (ppm)

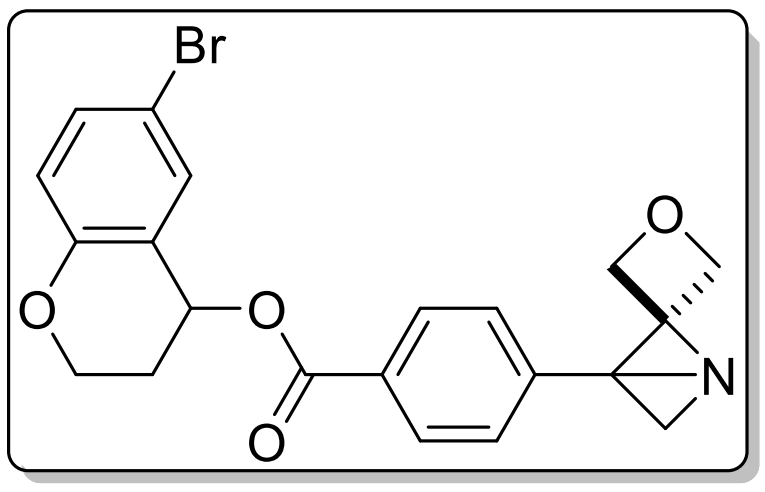


**31**

$^1$H NMR (400 MHz, $CDCl_3$)

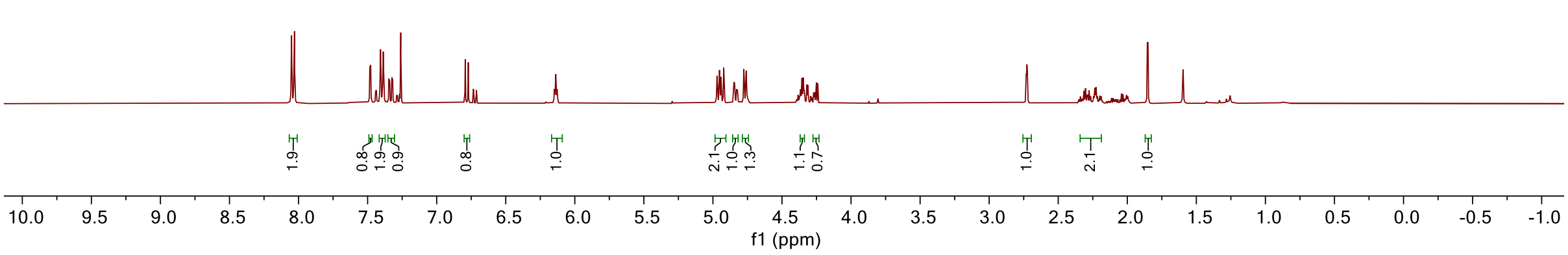


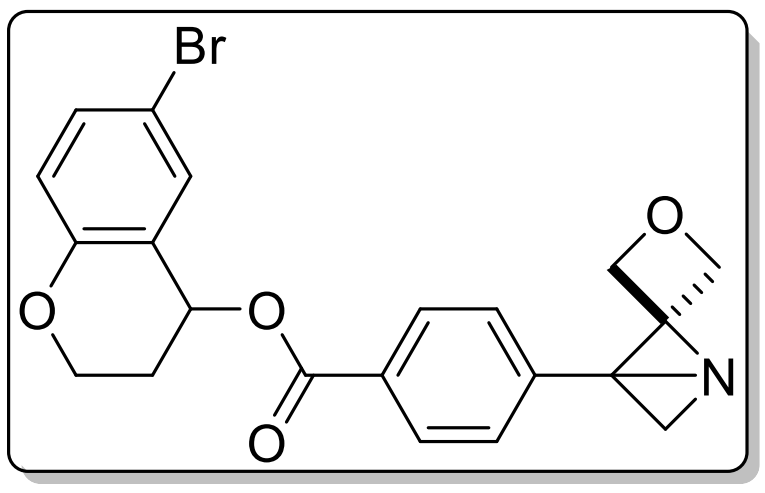


**31**

$^{13}$C {$^1$H} NMR (101 MHz, $CDCl_3$)

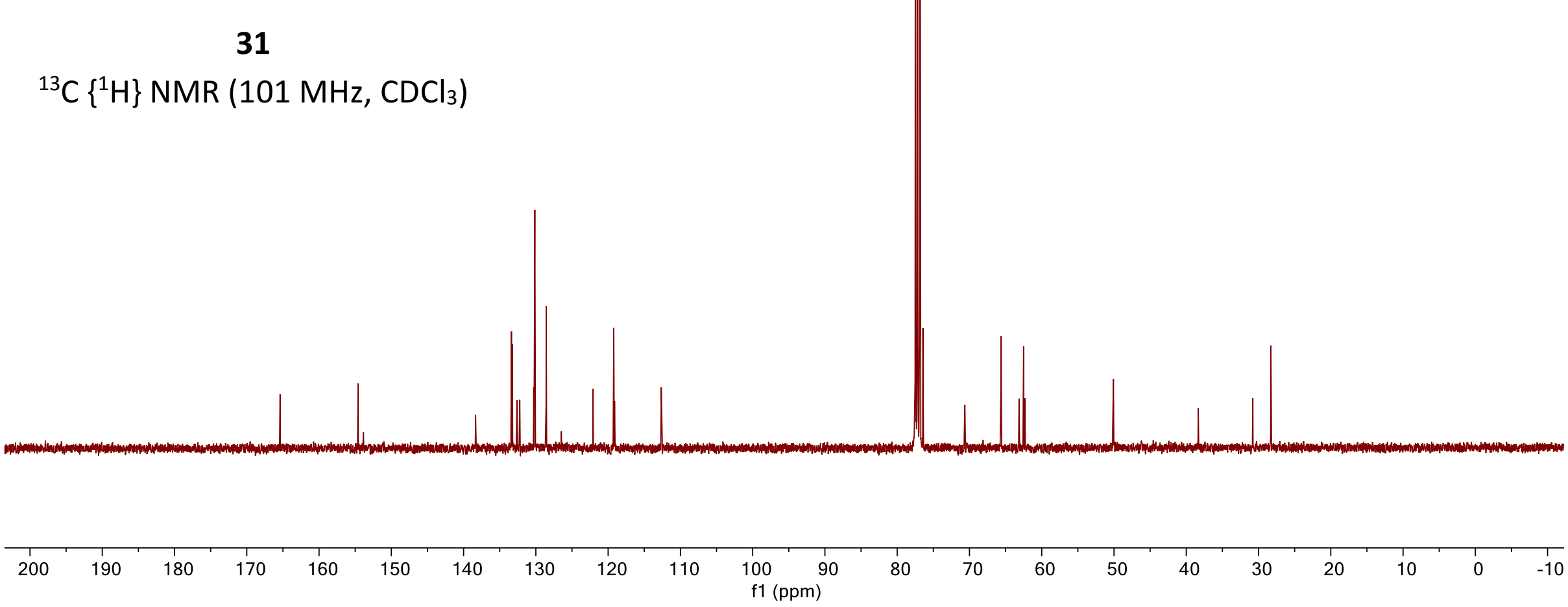

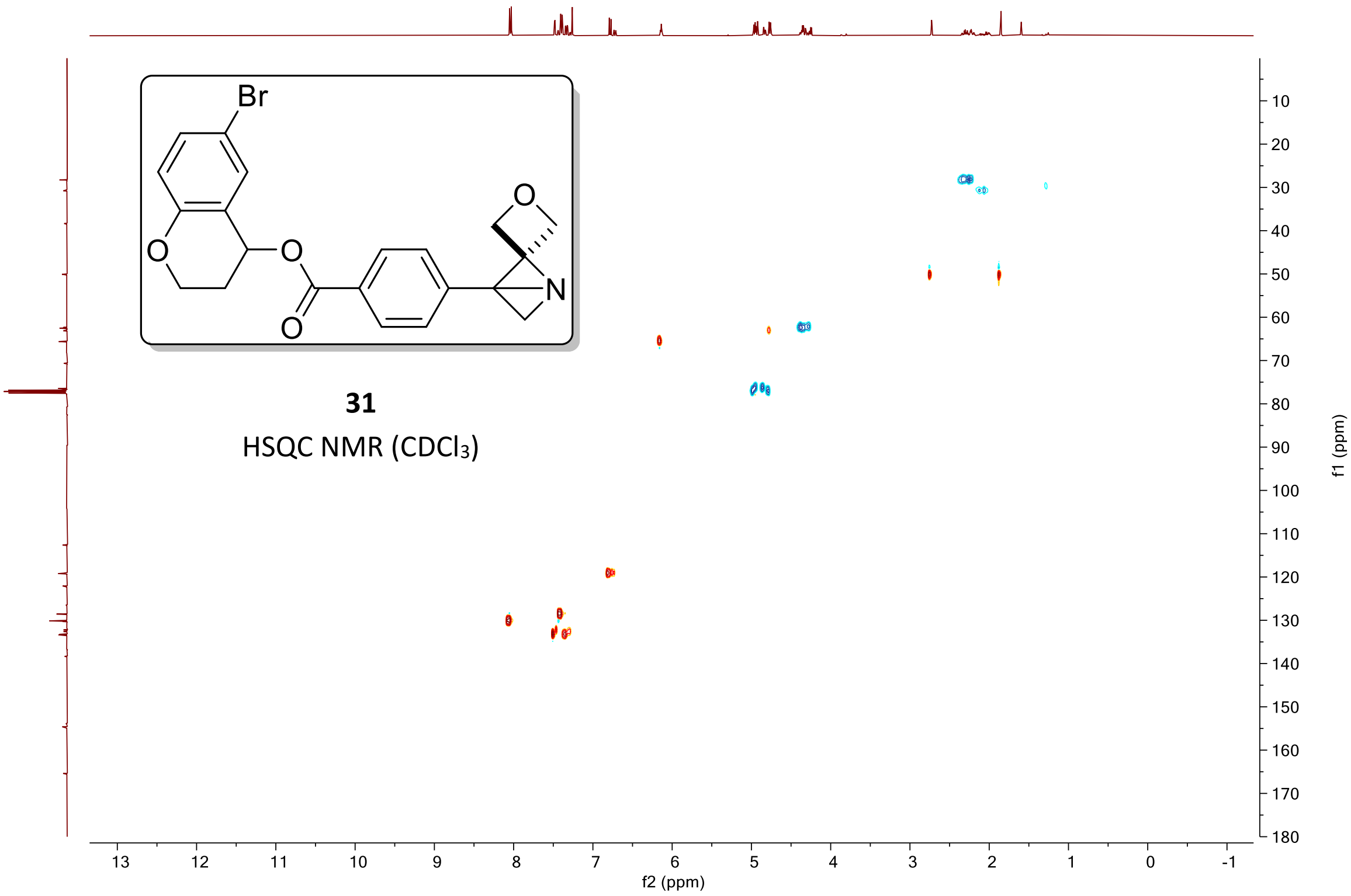
Br
O
O
O
O
N
31
HSQC NMR (CDCl$_3$)
f2 (ppm)
f1 (ppm)

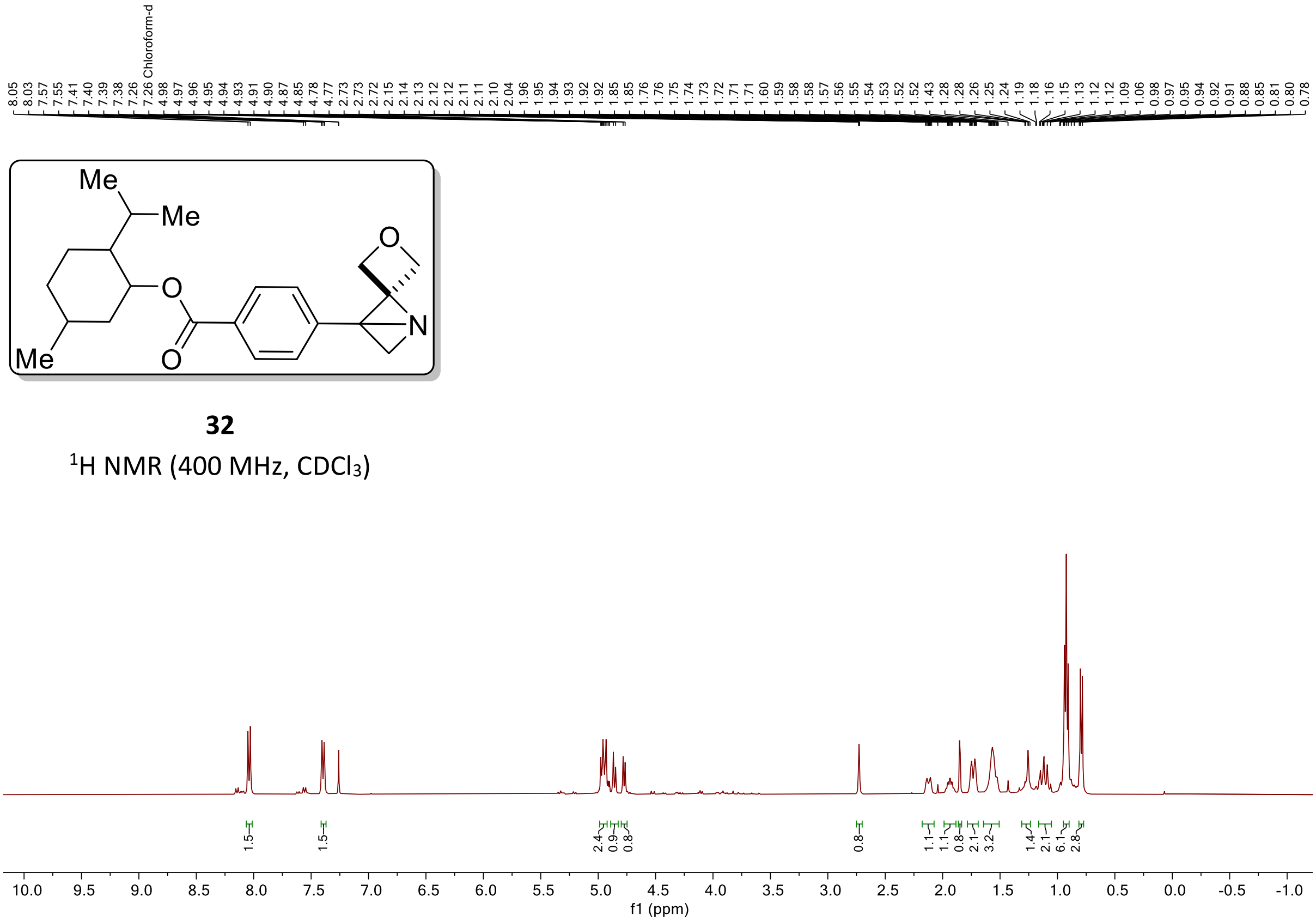


**32**

$^{1}H$ NMR (400 MHz, $CDCl_3$)

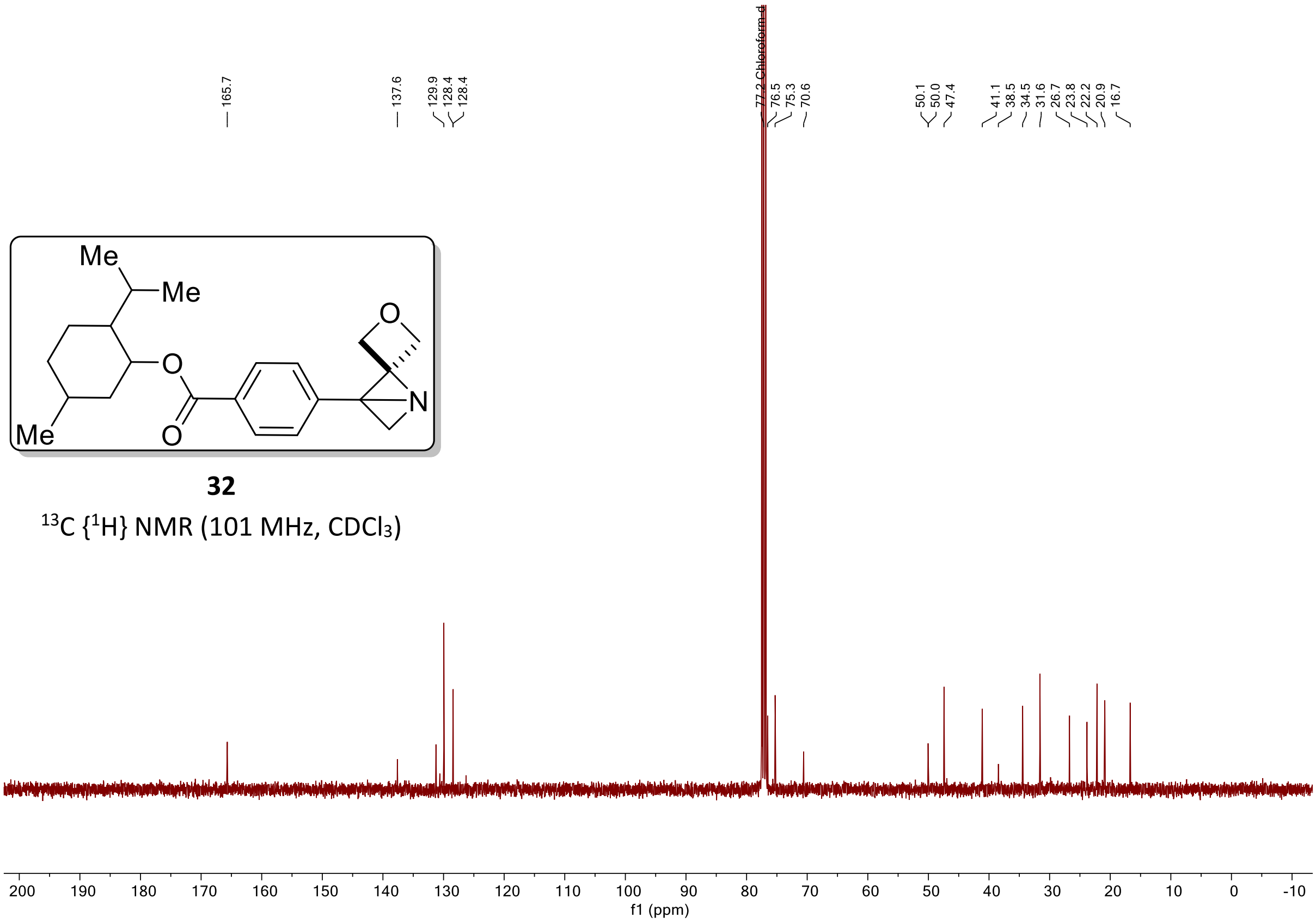


**32**

$^{13}C$ {$^{1}H$} NMR (101 MHz, $CDCl_3$)

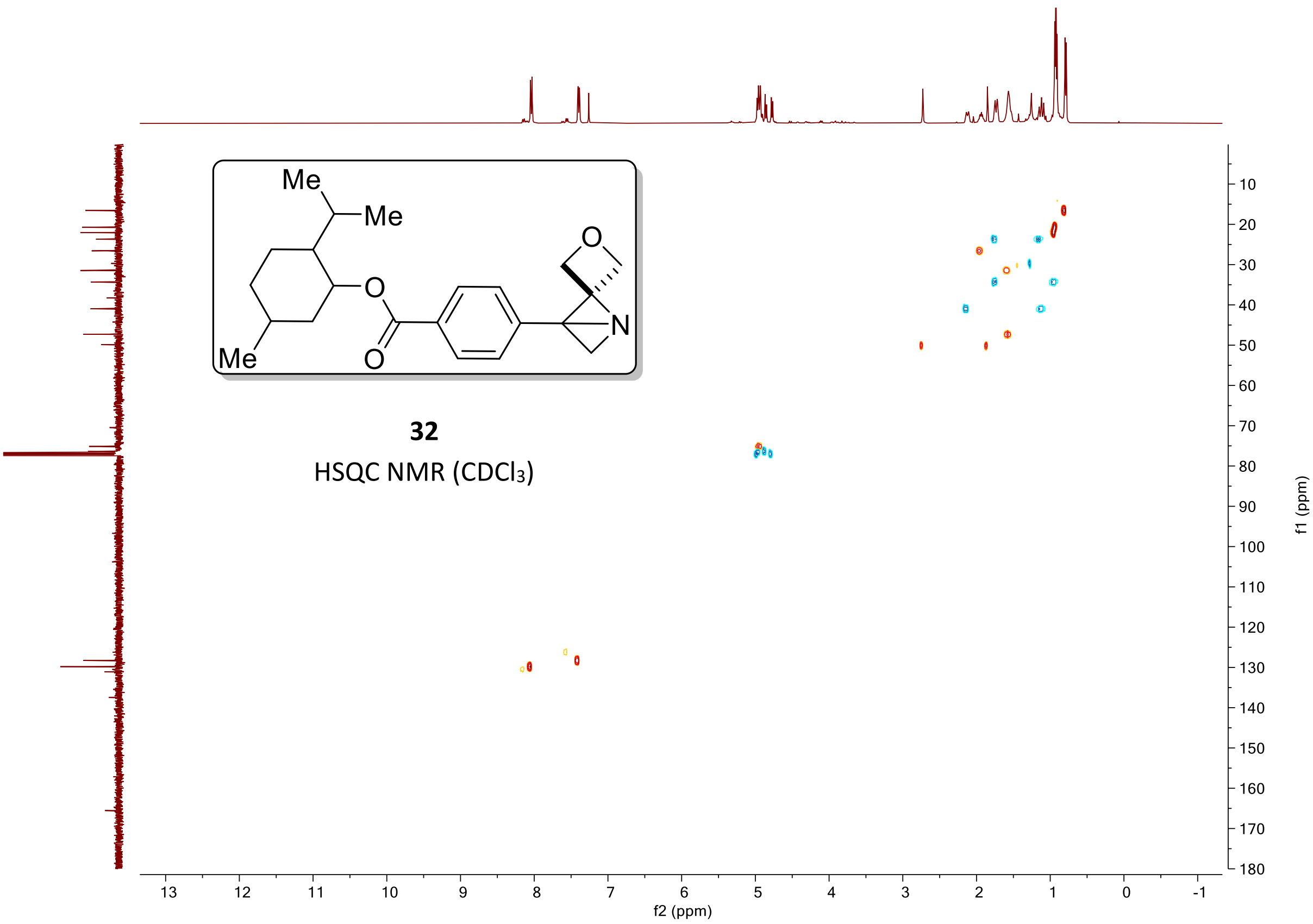


**32**

HSQC NMR ($CDCl_3$)

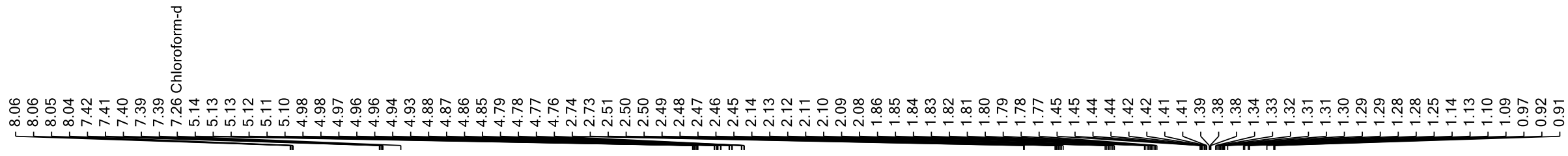


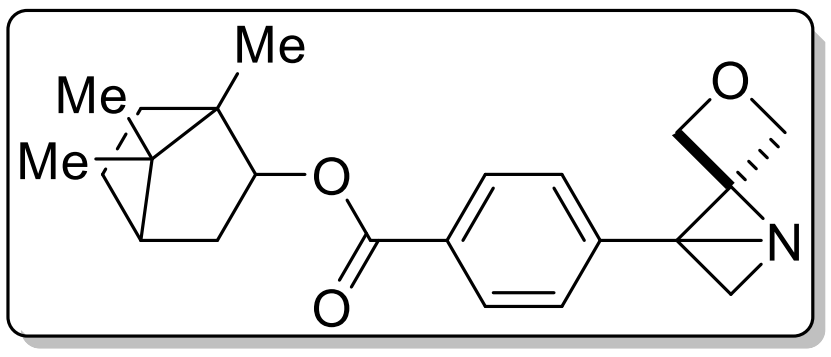


**33**

$^{1}H$ NMR (400 MHz, $CDCl_3$)

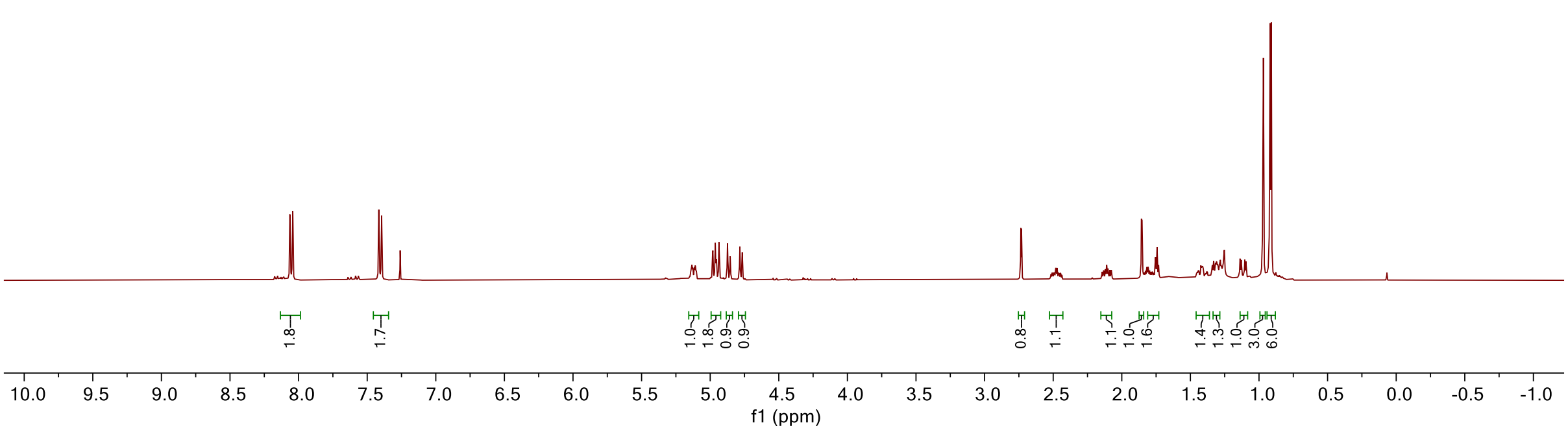


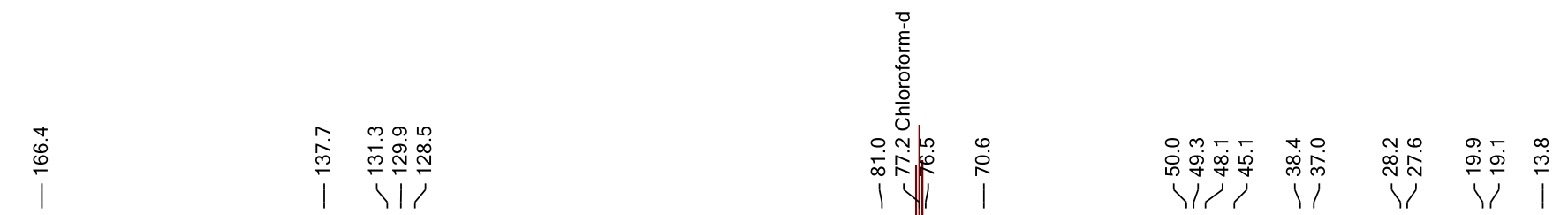


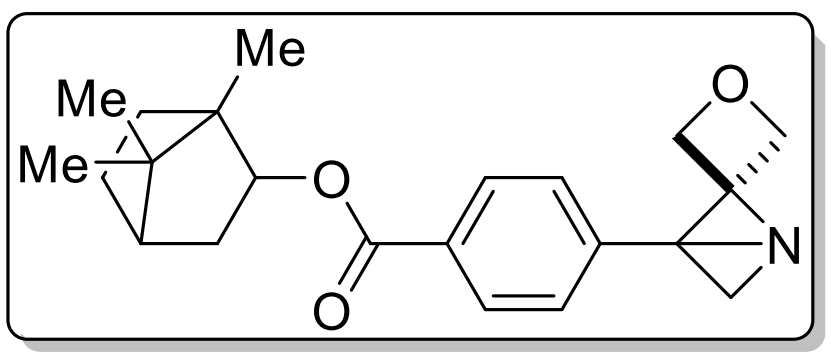


**33**

$^{13}C$ {$^{1}H$} NMR (101 MHz, $CDCl_3$)

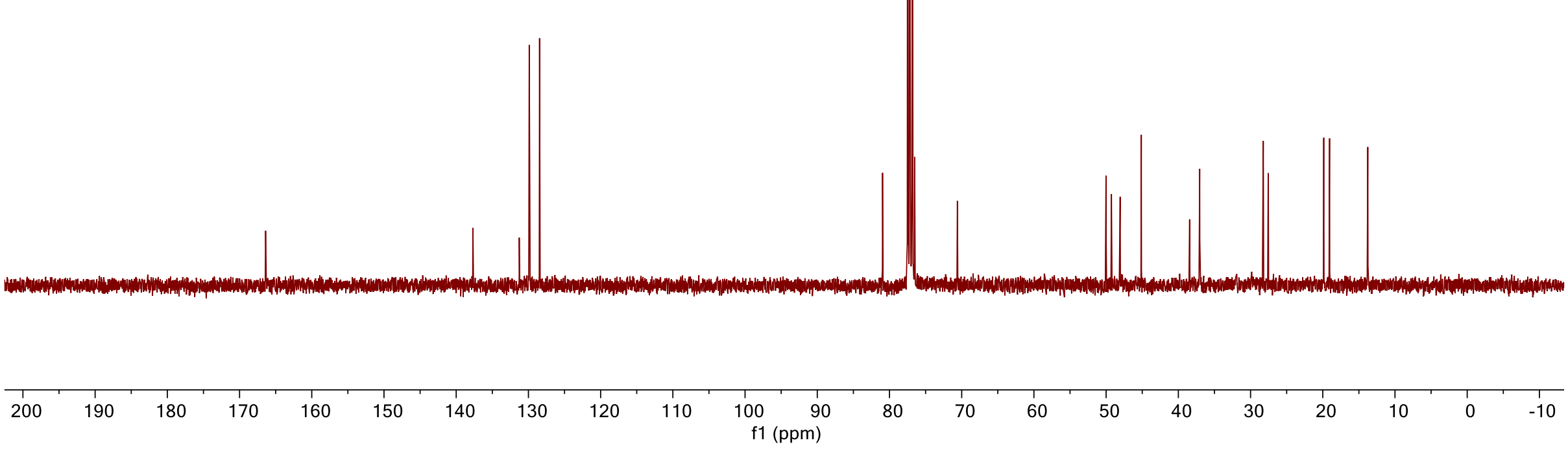

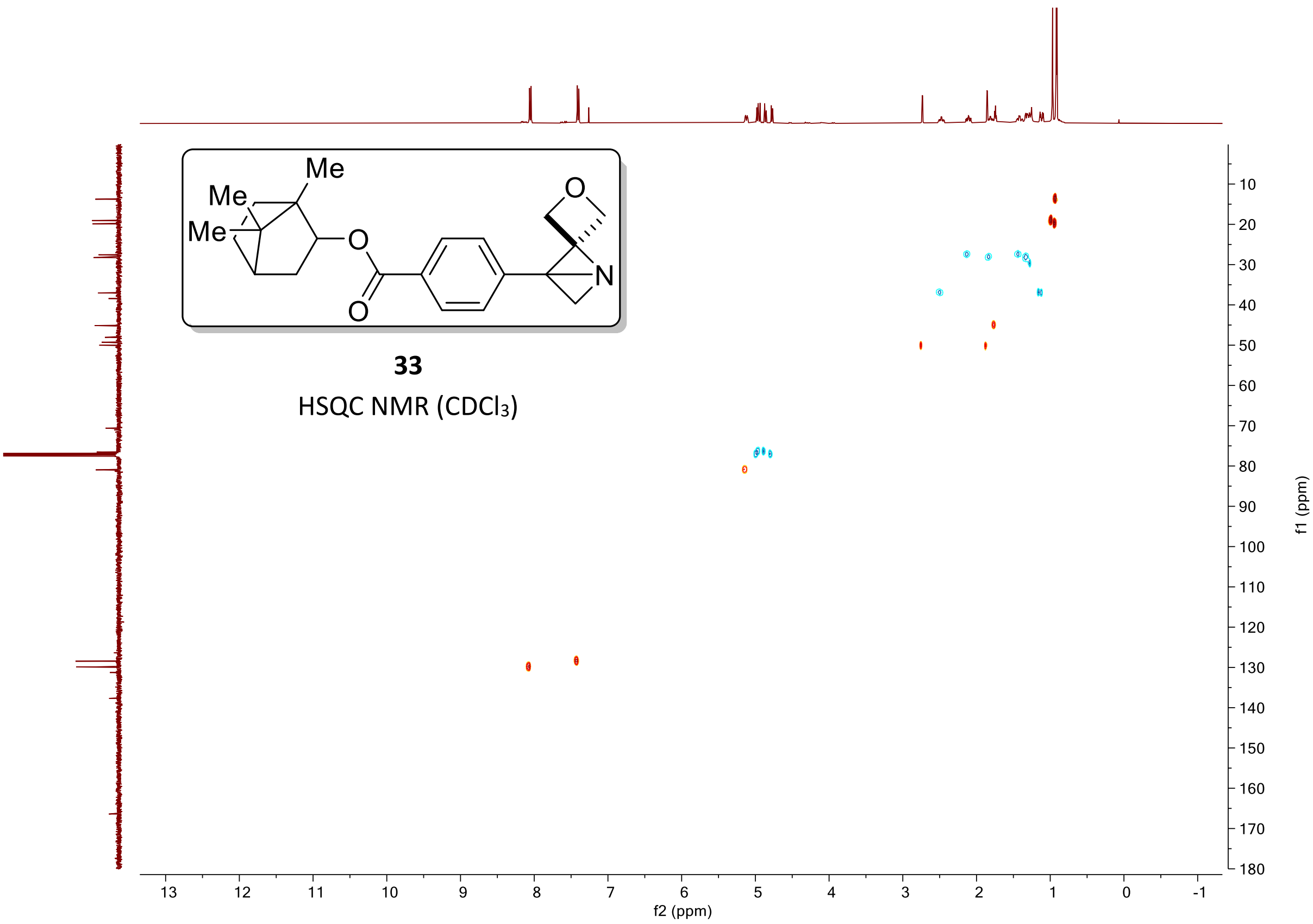


**33**
HSQC NMR ($CDCl_3$)

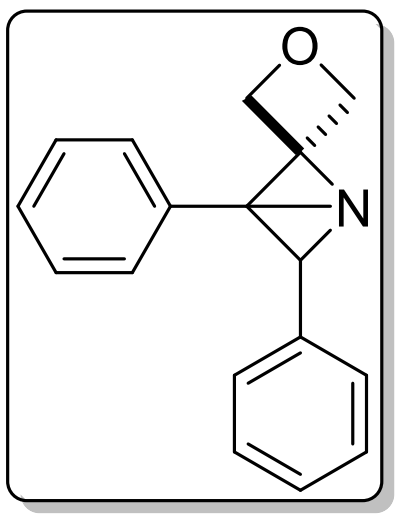

**34**

$^{1}$H NMR (400 MHz, $CDCl_3$)

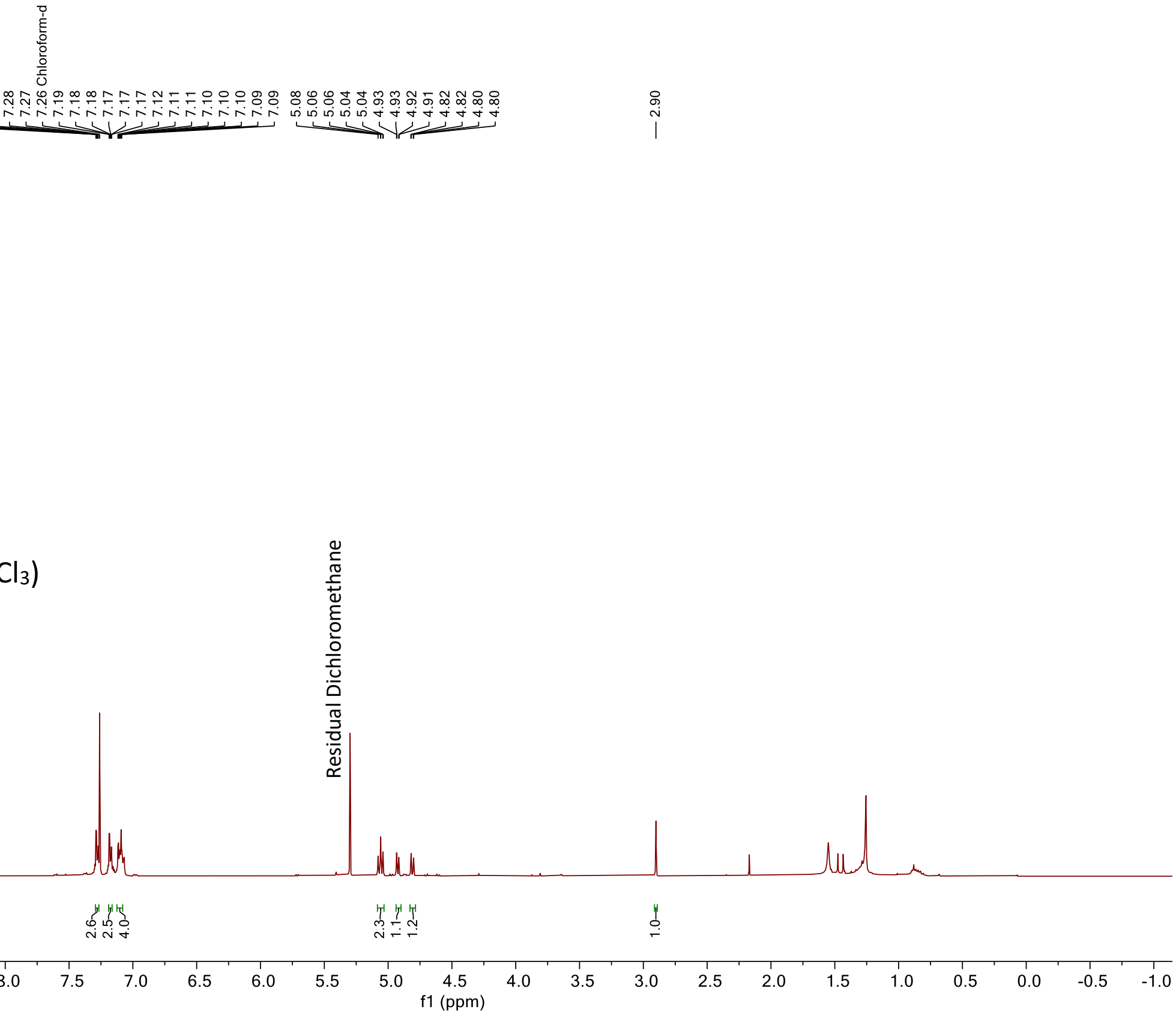


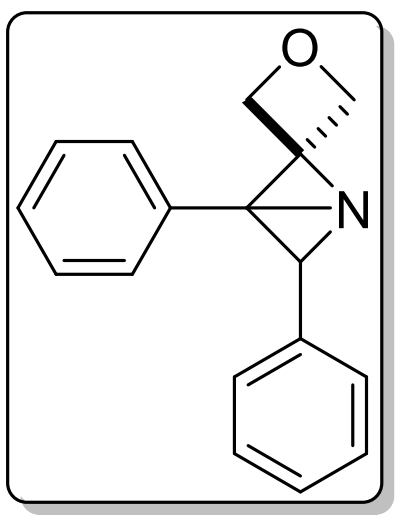

**34**

$^{13}$C {$^{1}$H} NMR (101 MHz, $CDCl_3$)

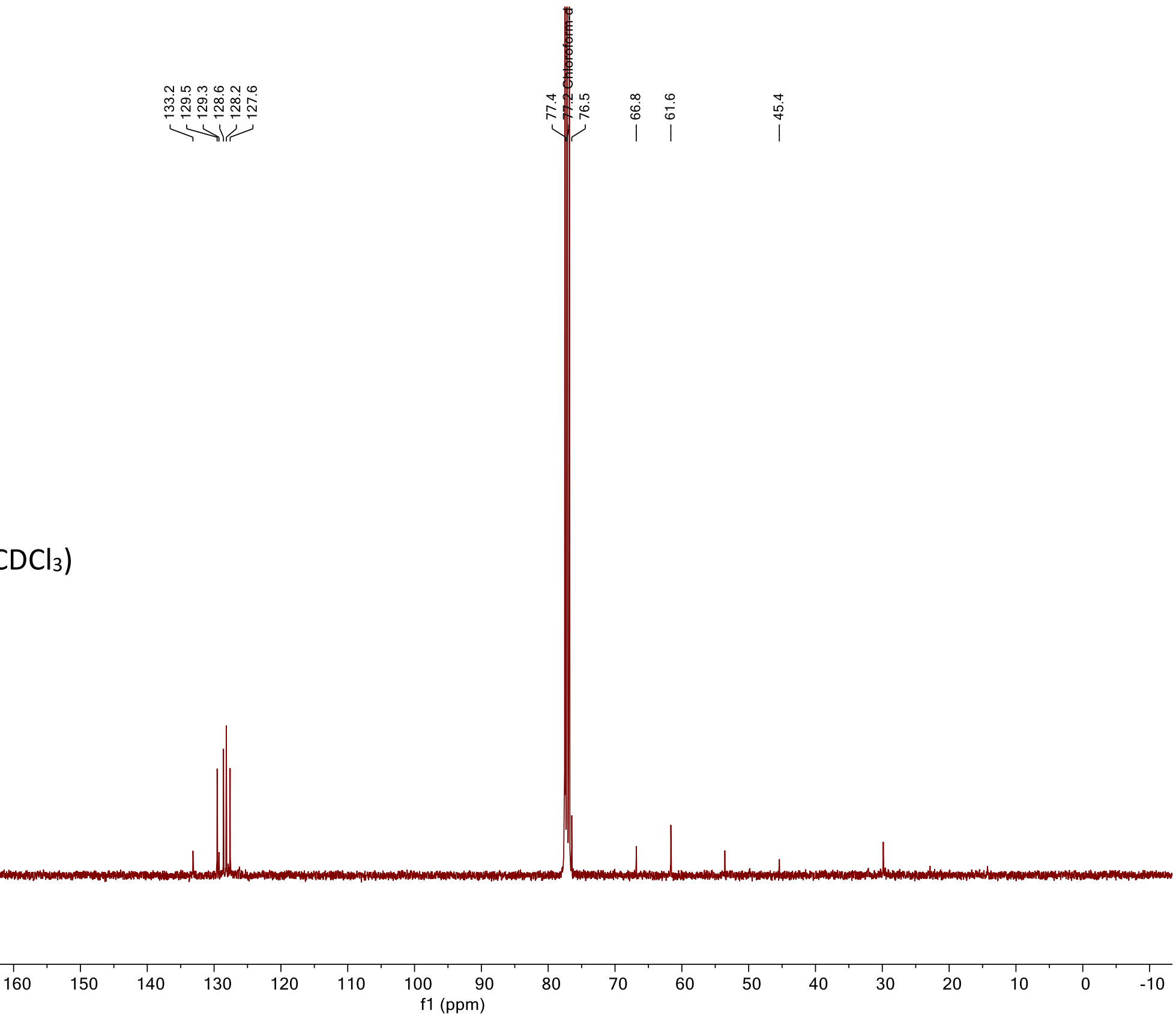

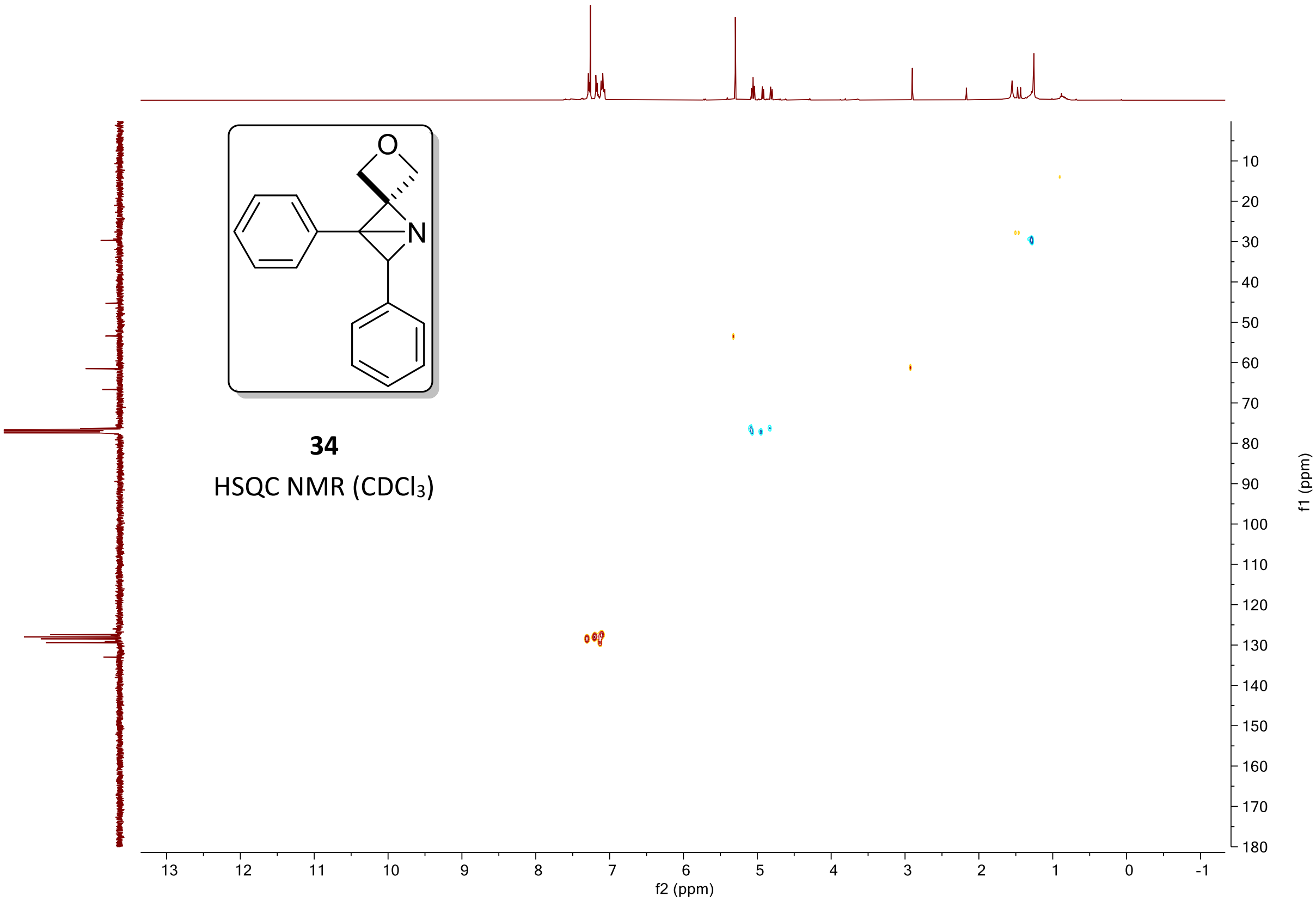


**34**

HSQC NMR ($CDCl_3$)

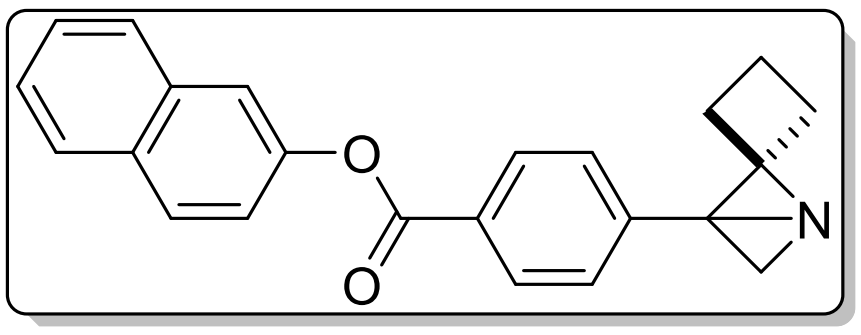


**43**

$^{1}$H NMR (400 MHz, $CDCl_3$)

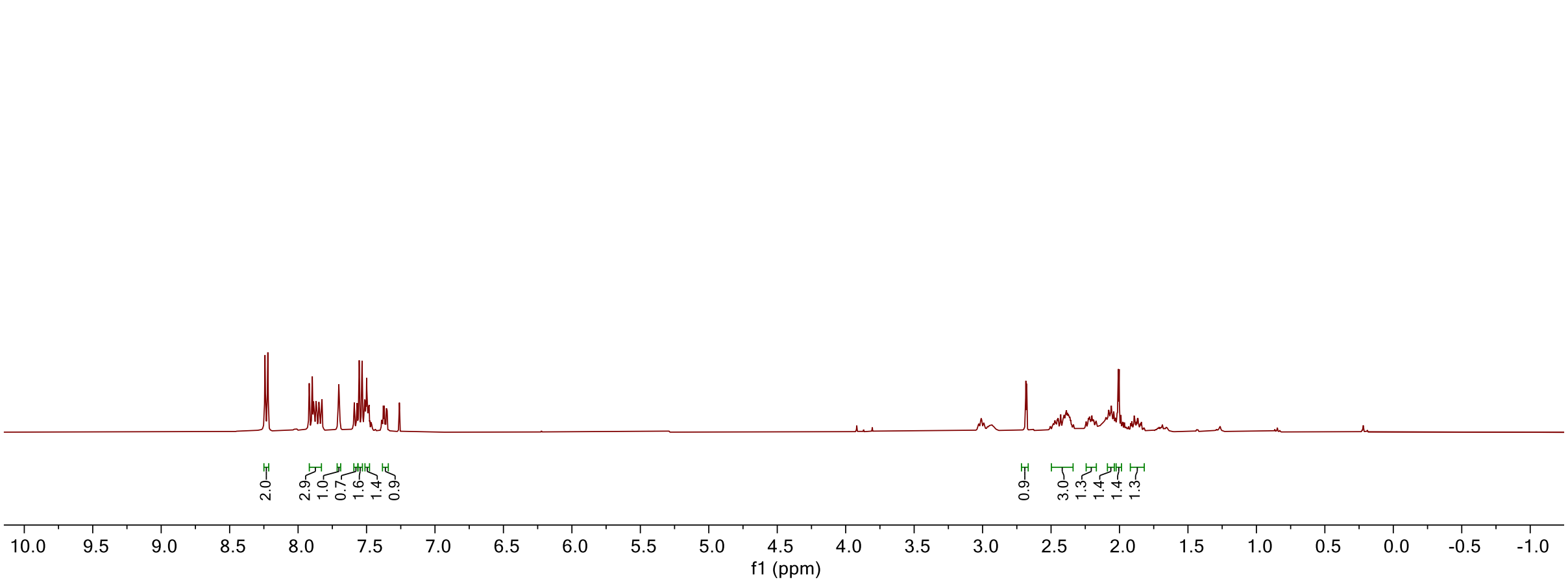


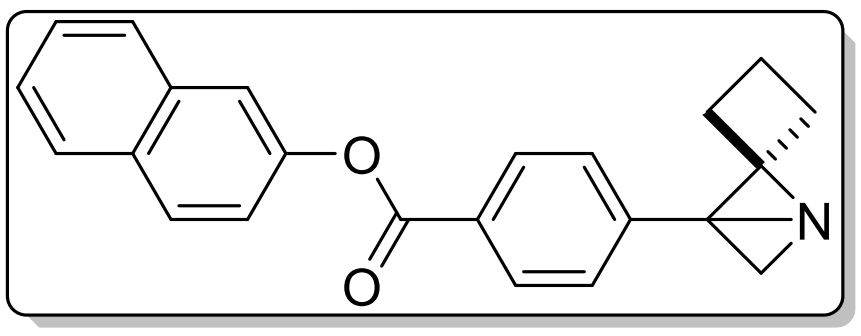


**43**

$^{13}C$ {$^{1}$H} NMR (101 MHz, $CDCl_3$)

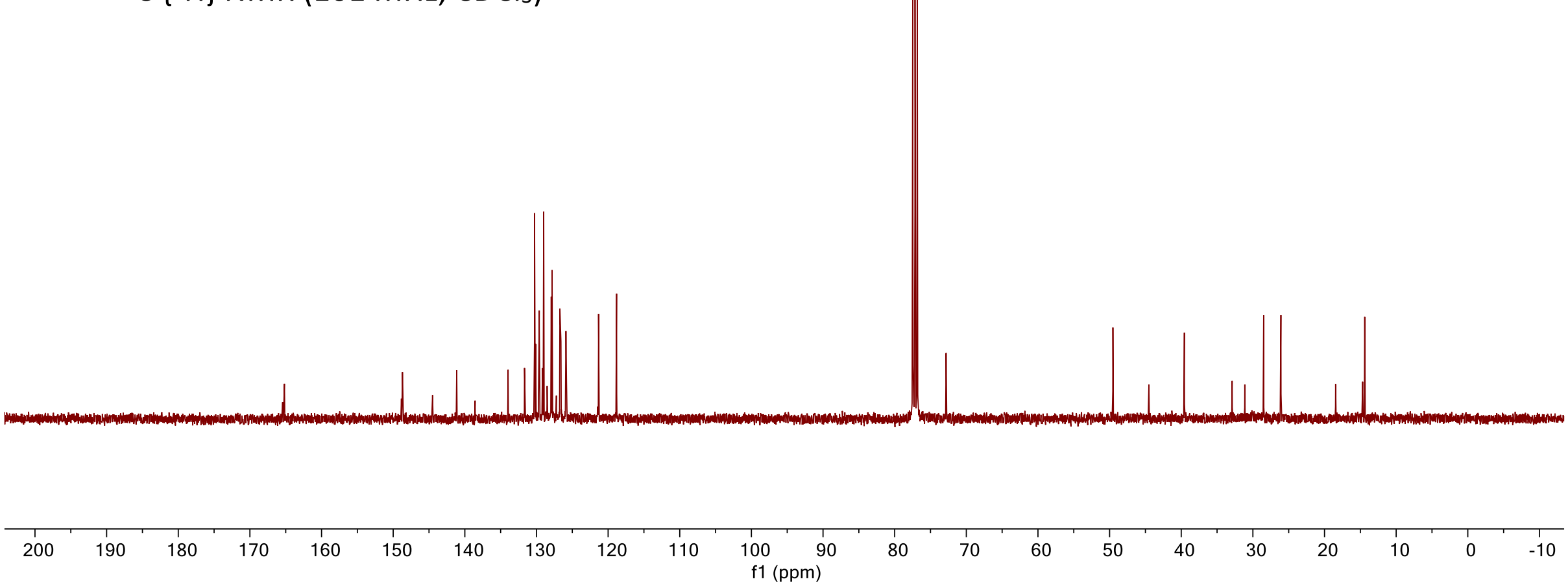

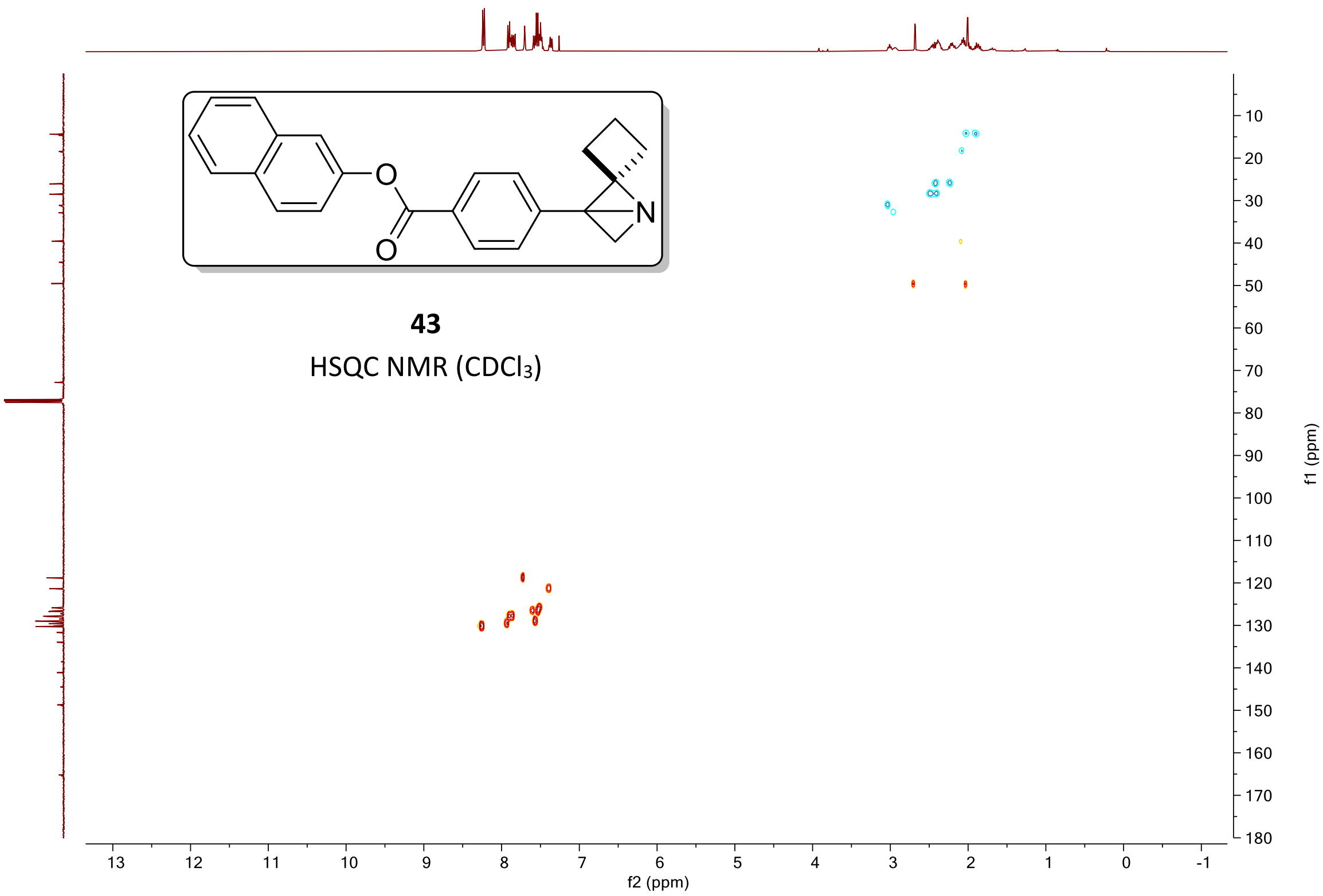


**43**

HSQC NMR ($CDCl_3$)

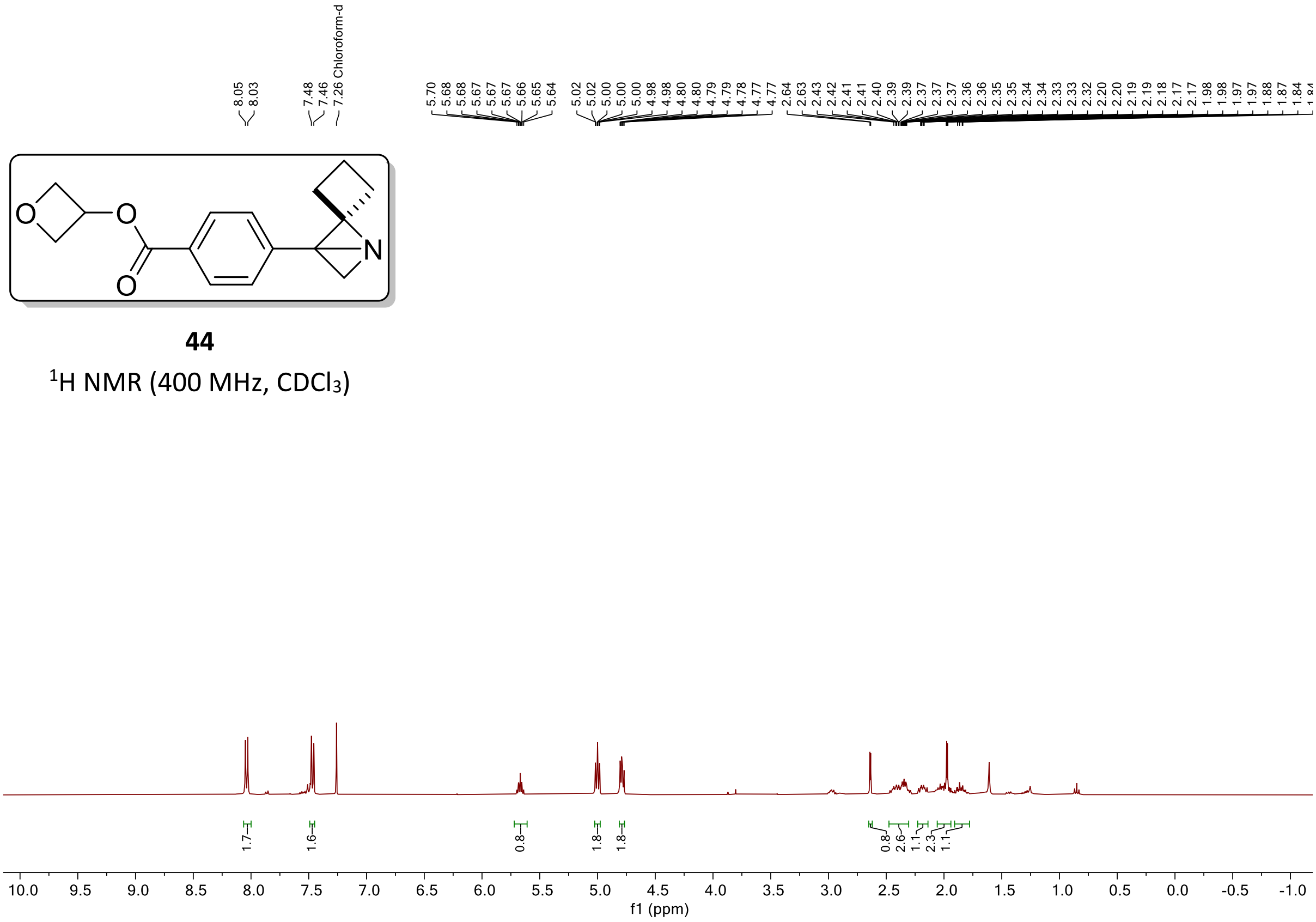

**44**

$^{1}$H NMR (400 MHz, $CDCl_3$)

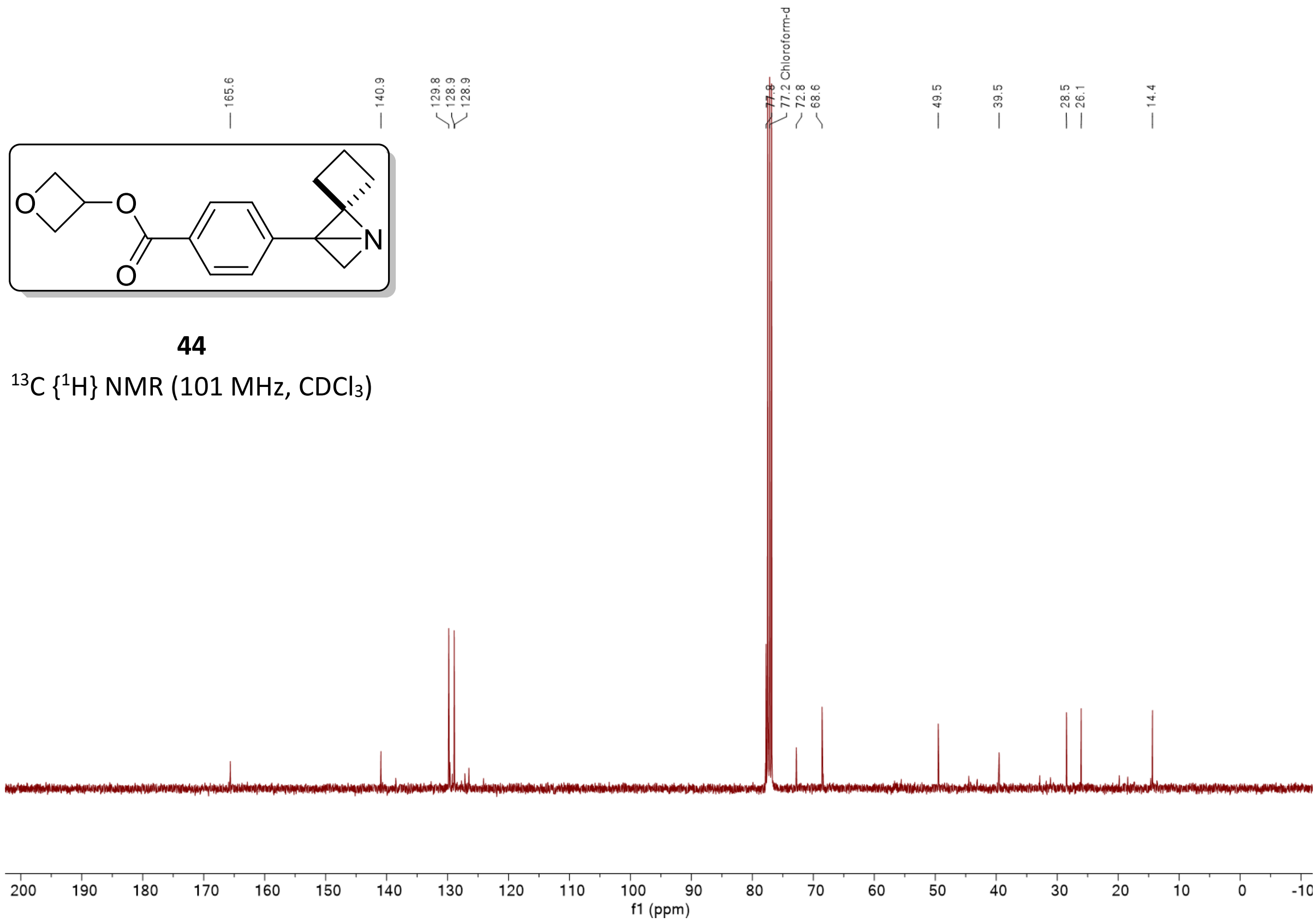

**44**

$^{13}$C {$^{1}$H} NMR (101 MHz, $CDCl_3$)

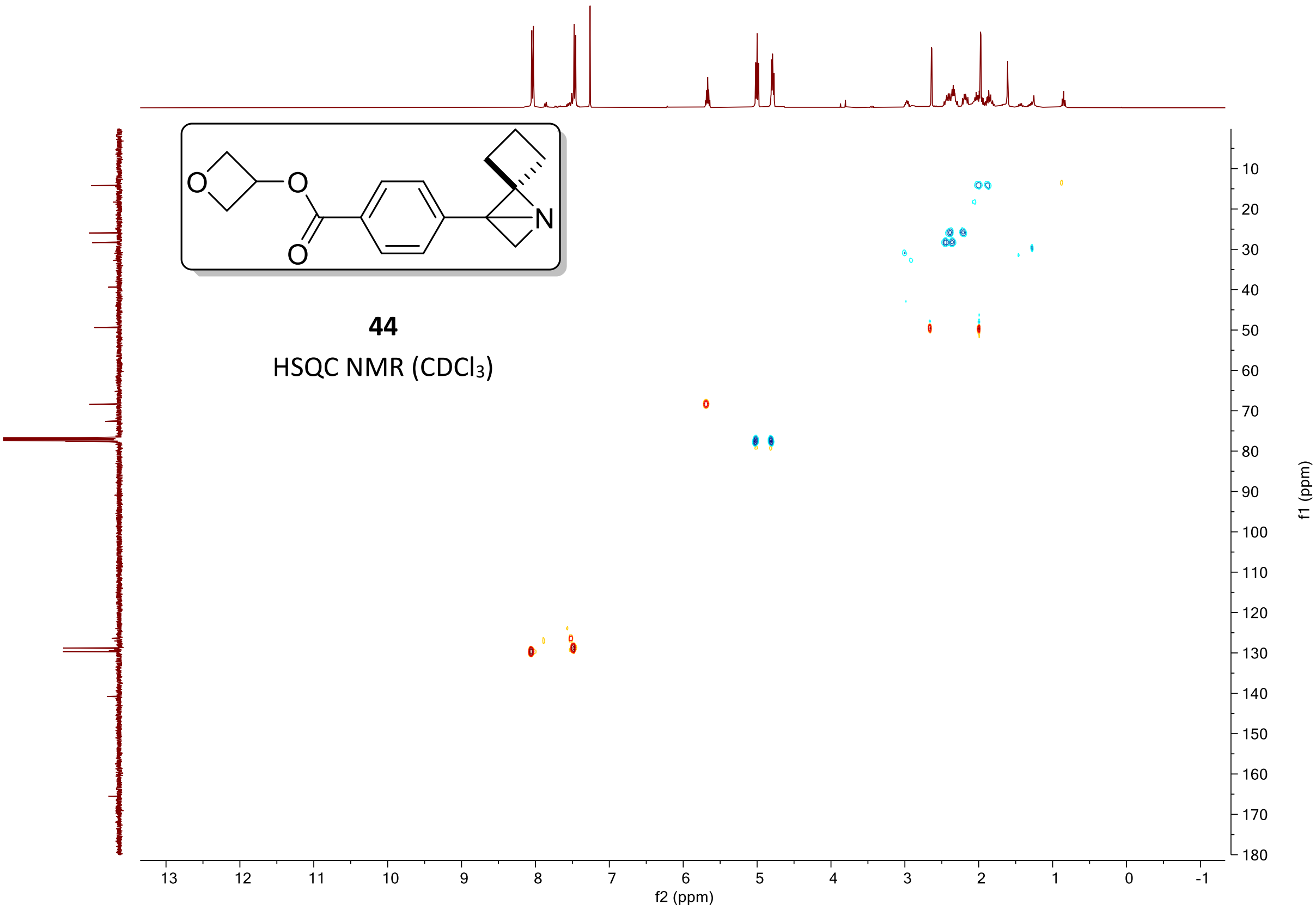
O
O
O
N
44
HSQC NMR (CDCl3)
f2 (ppm)
f1 (ppm)

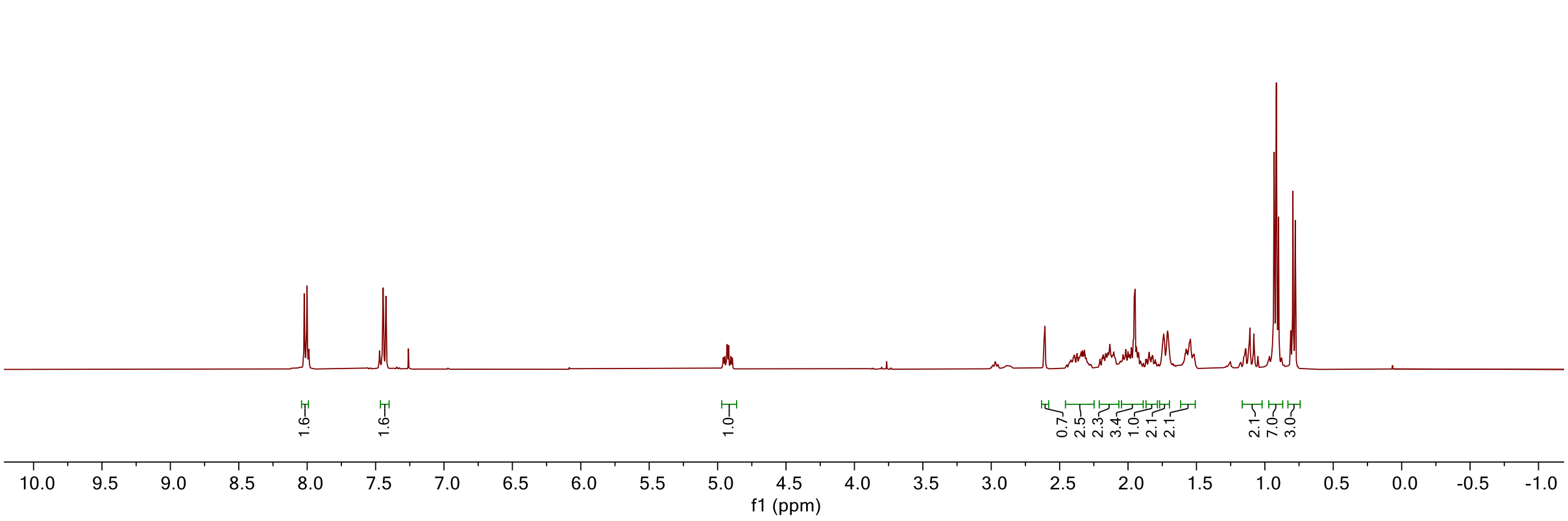


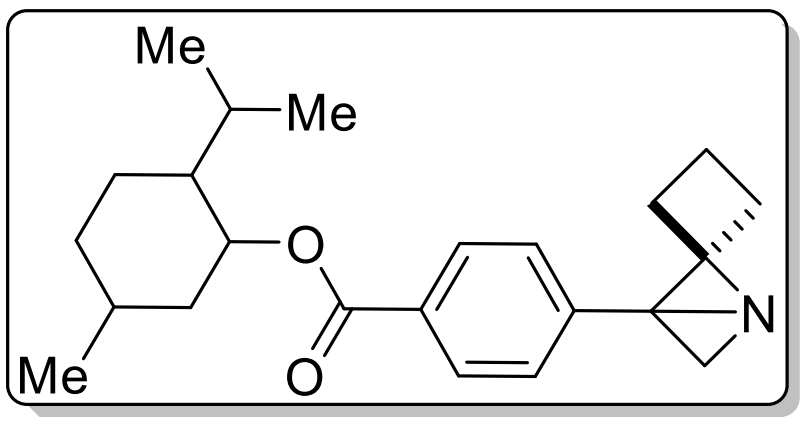


**45**

$^{1}$H NMR (400 MHz, $CDCl_3$)

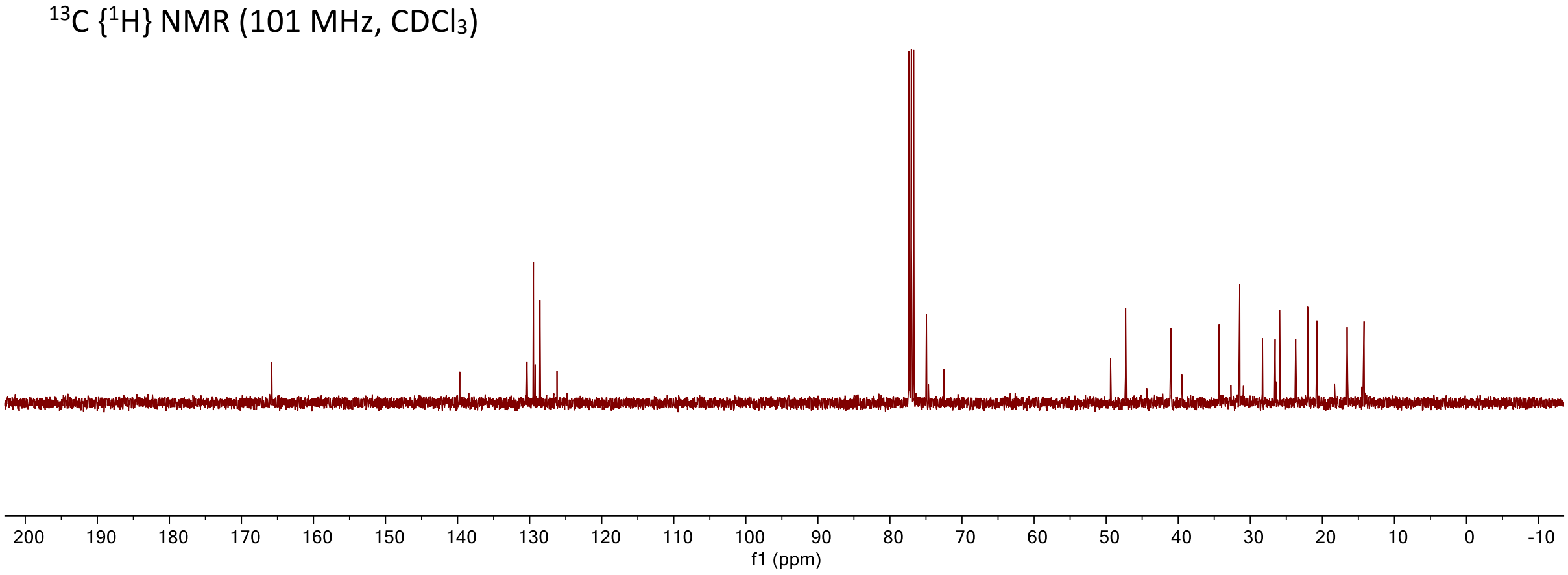


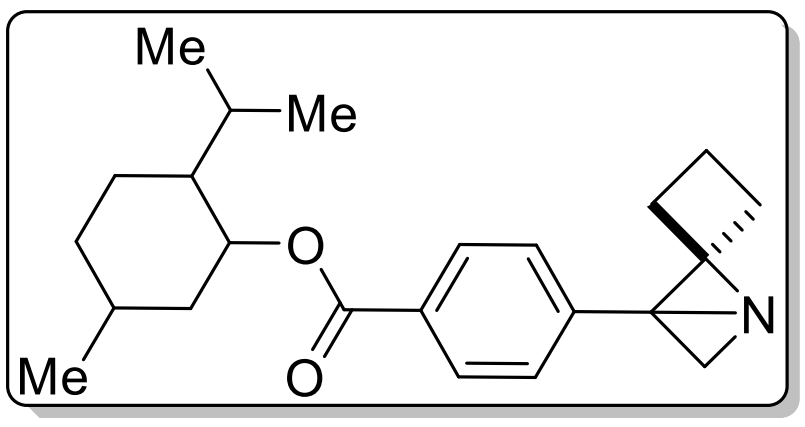


**45**

$^{13}$C {$^{1}$H} NMR (101 MHz, $CDCl_3$)

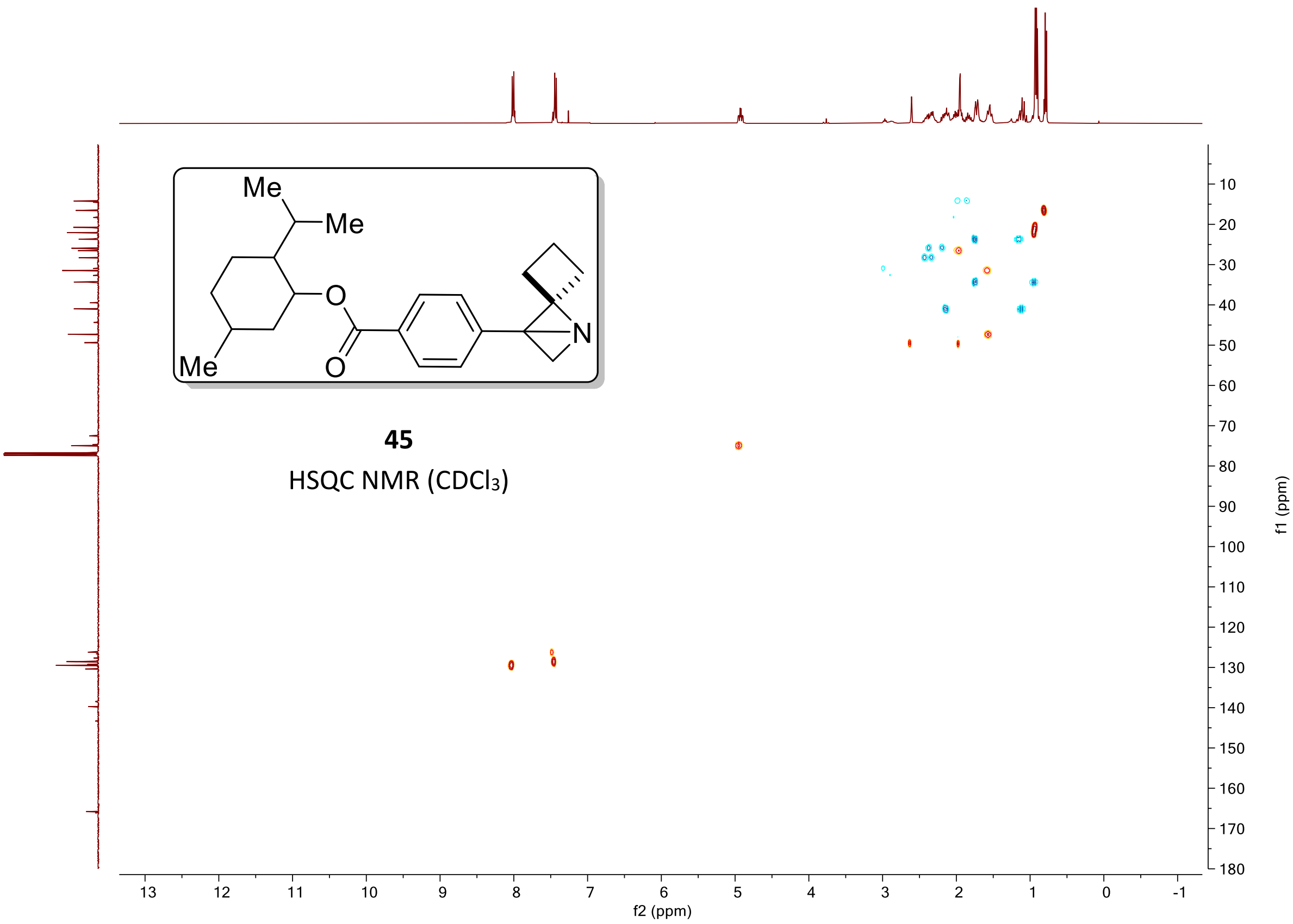
Me
Me
O
O
N
Me
45
HSQC NMR (CDCl3)
f1 (ppm)
f2 (ppm)

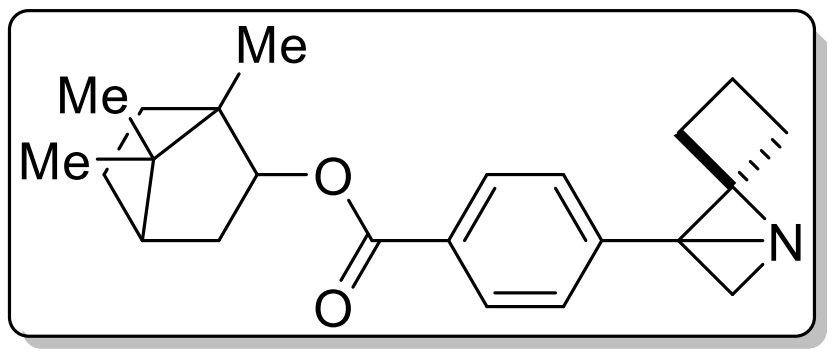


**46**

$^{1}$H NMR (400 MHz, $CDCl_3$)

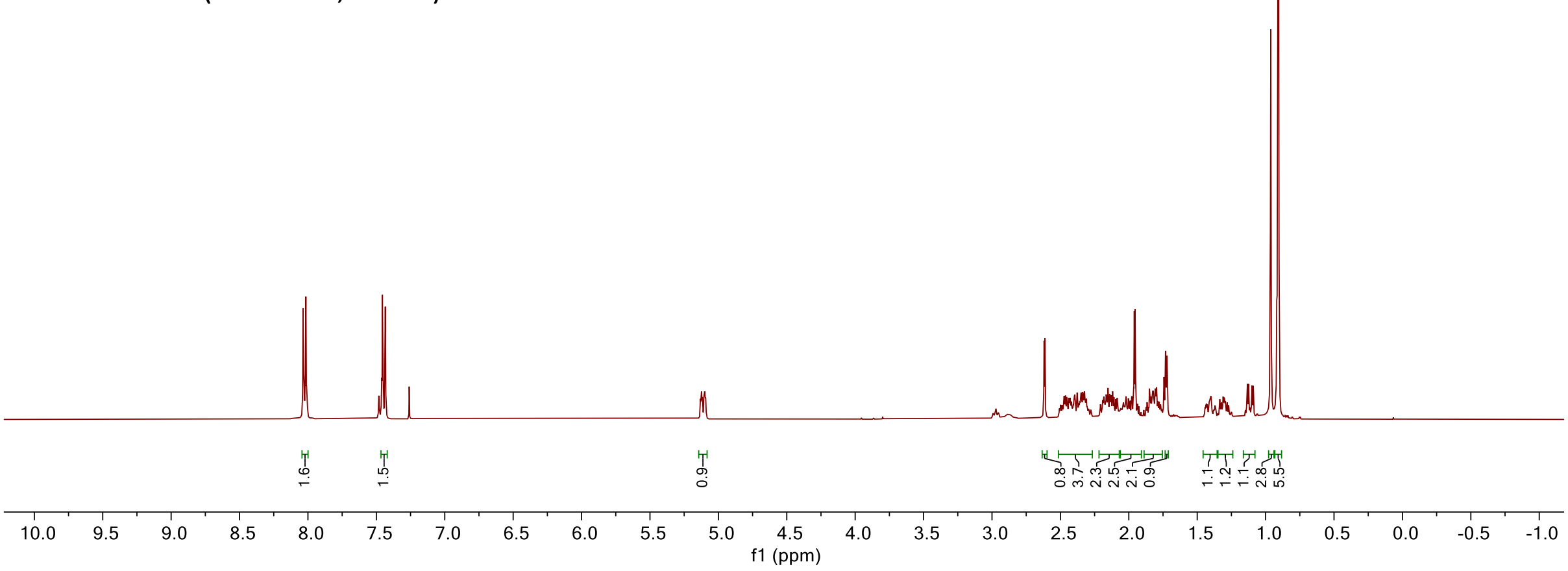


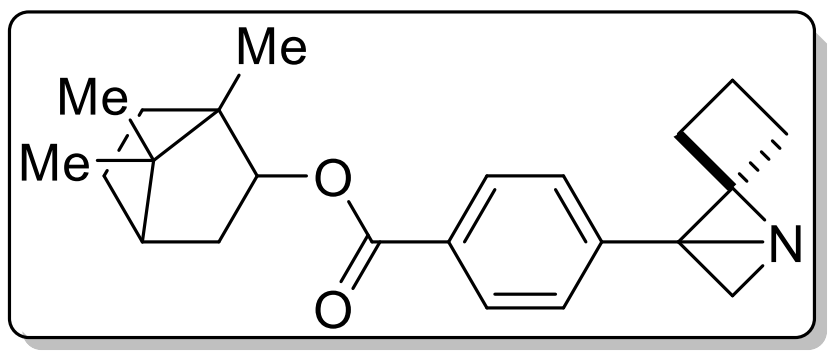


**46**

$^{13}$C {$^{1}$H} NMR (101 MHz, $CDCl_3$)

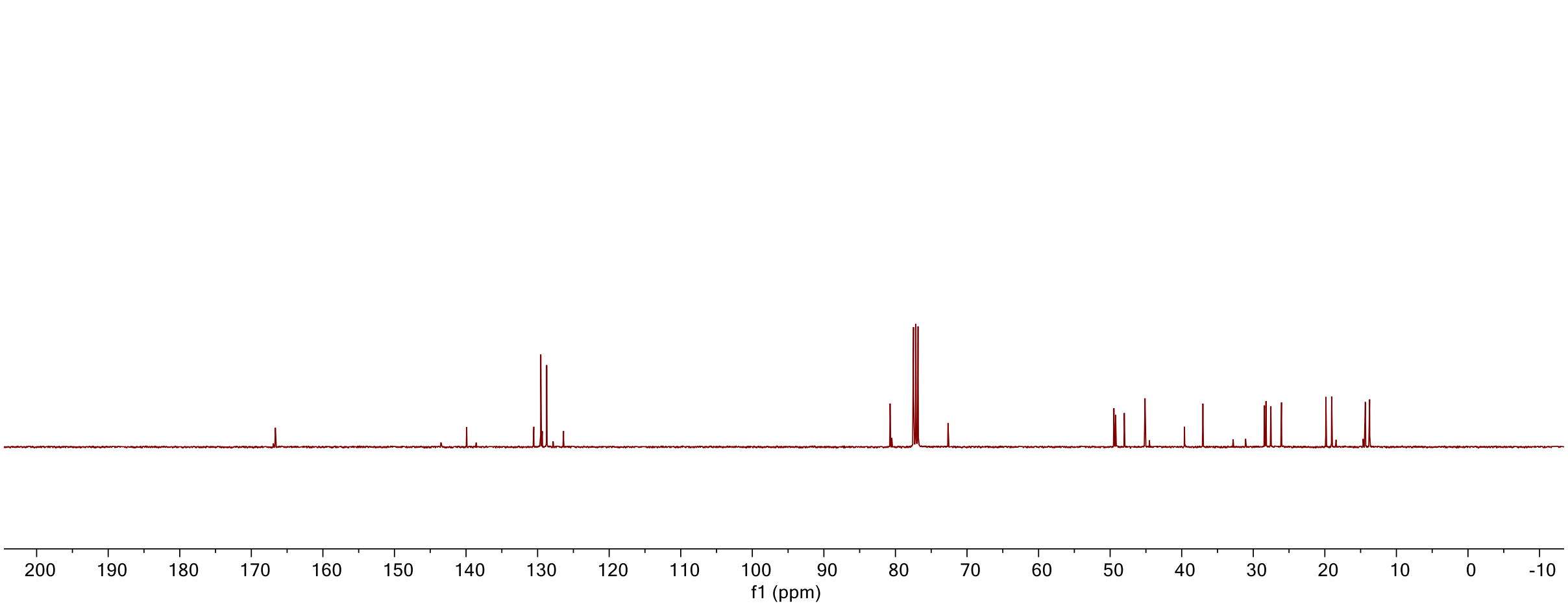

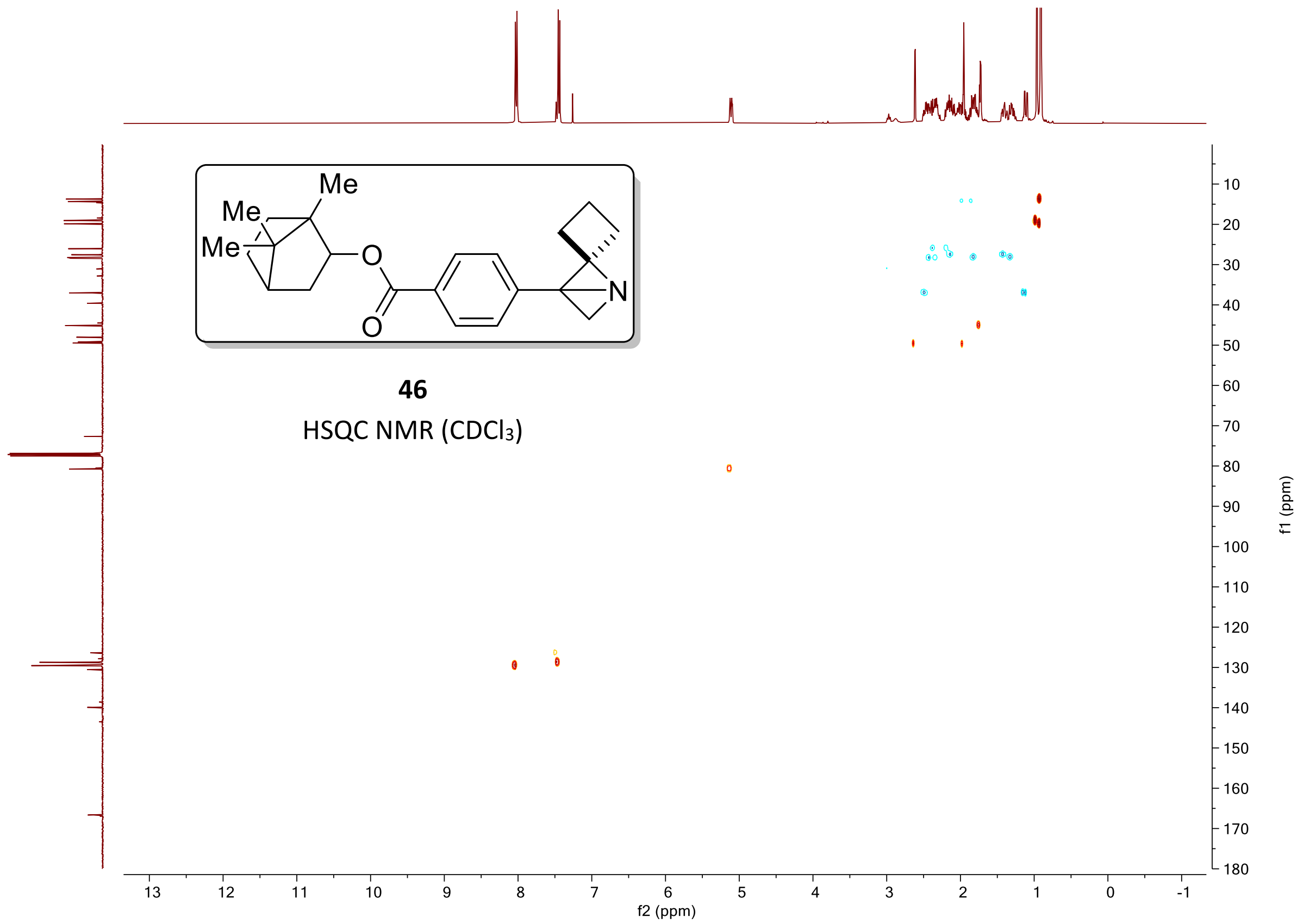

Me
Me
Me
O
O
N
46
HSQC NMR (CDCl3)
f2 (ppm)
f1 (ppm)

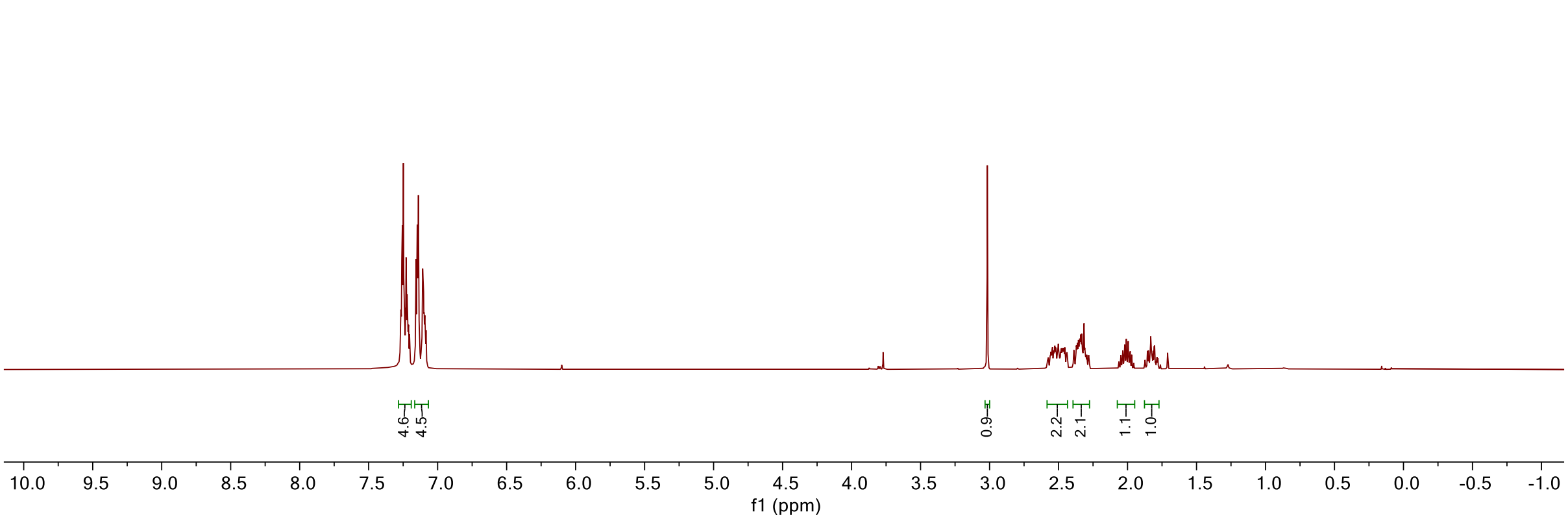


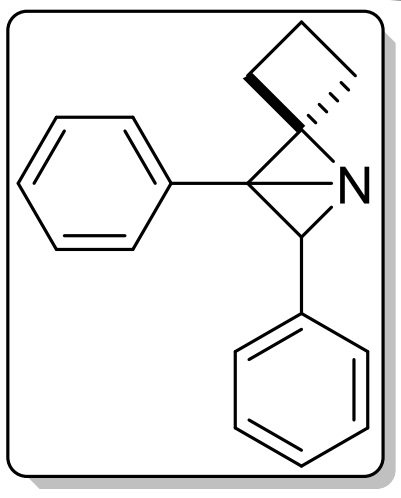

**47**

$^{1}H$ NMR (400 MHz, $CDCl_3$)

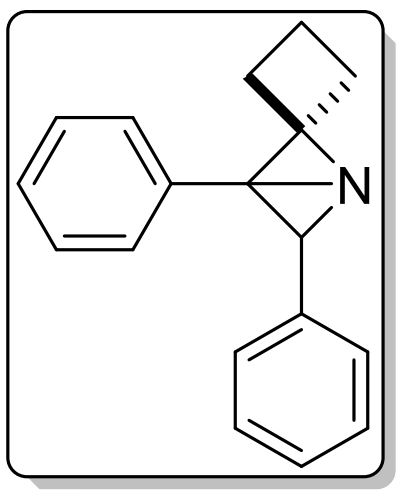

**47**

$^{13}C$ {$^{1}H$} NMR (101 MHz, $CDCl_3$)

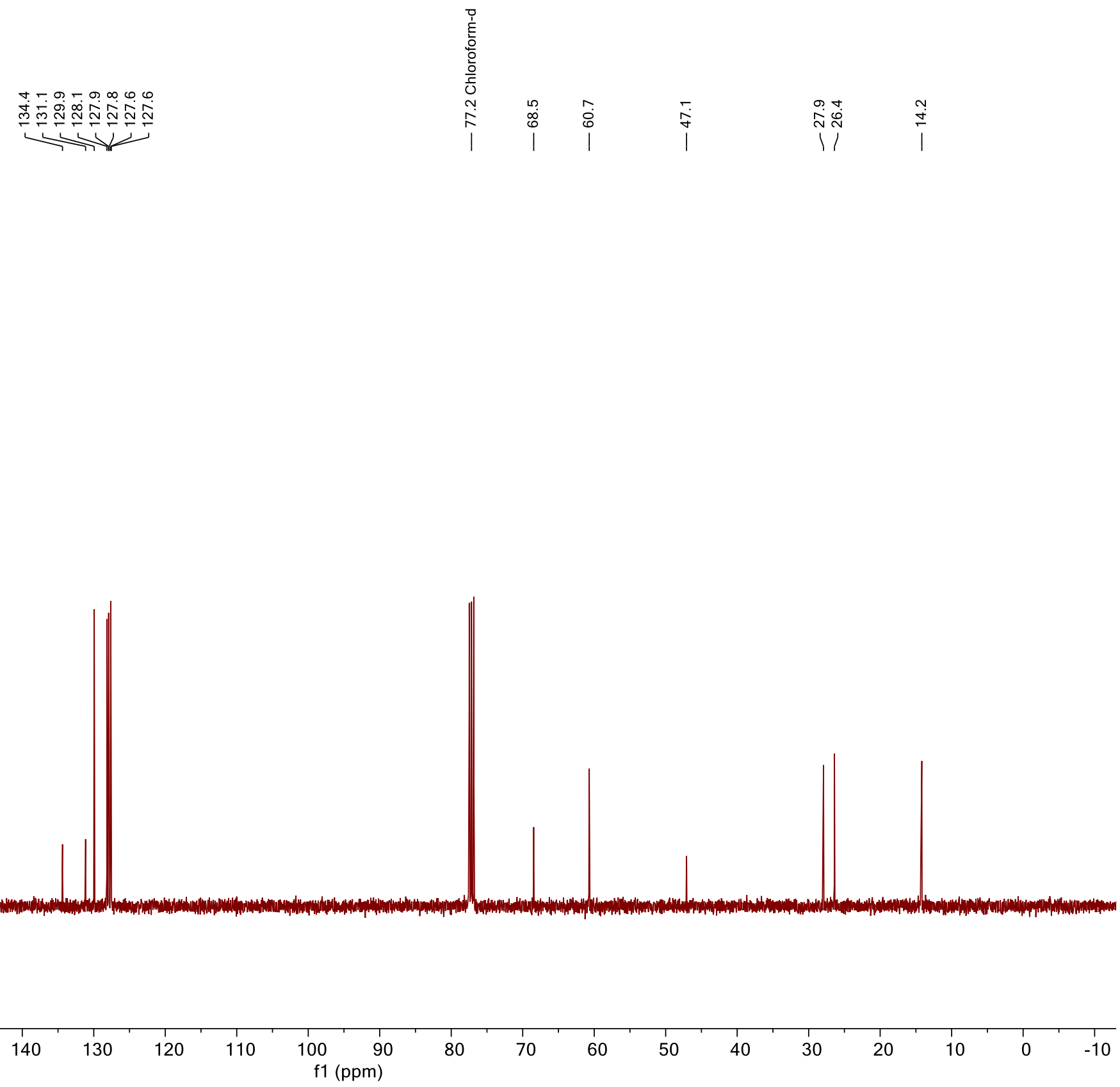

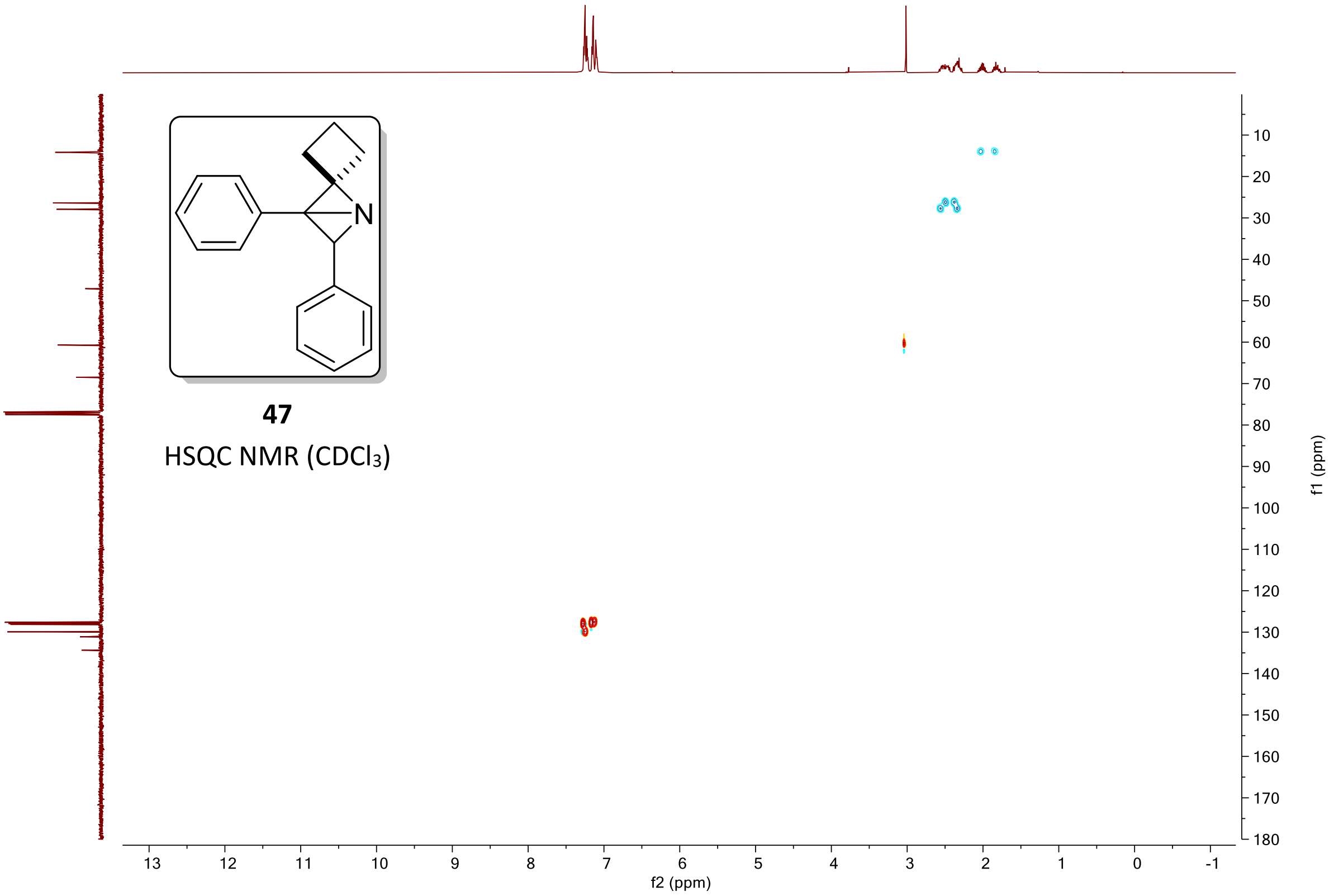


**47**

HSQC NMR ($CDCl_3$)

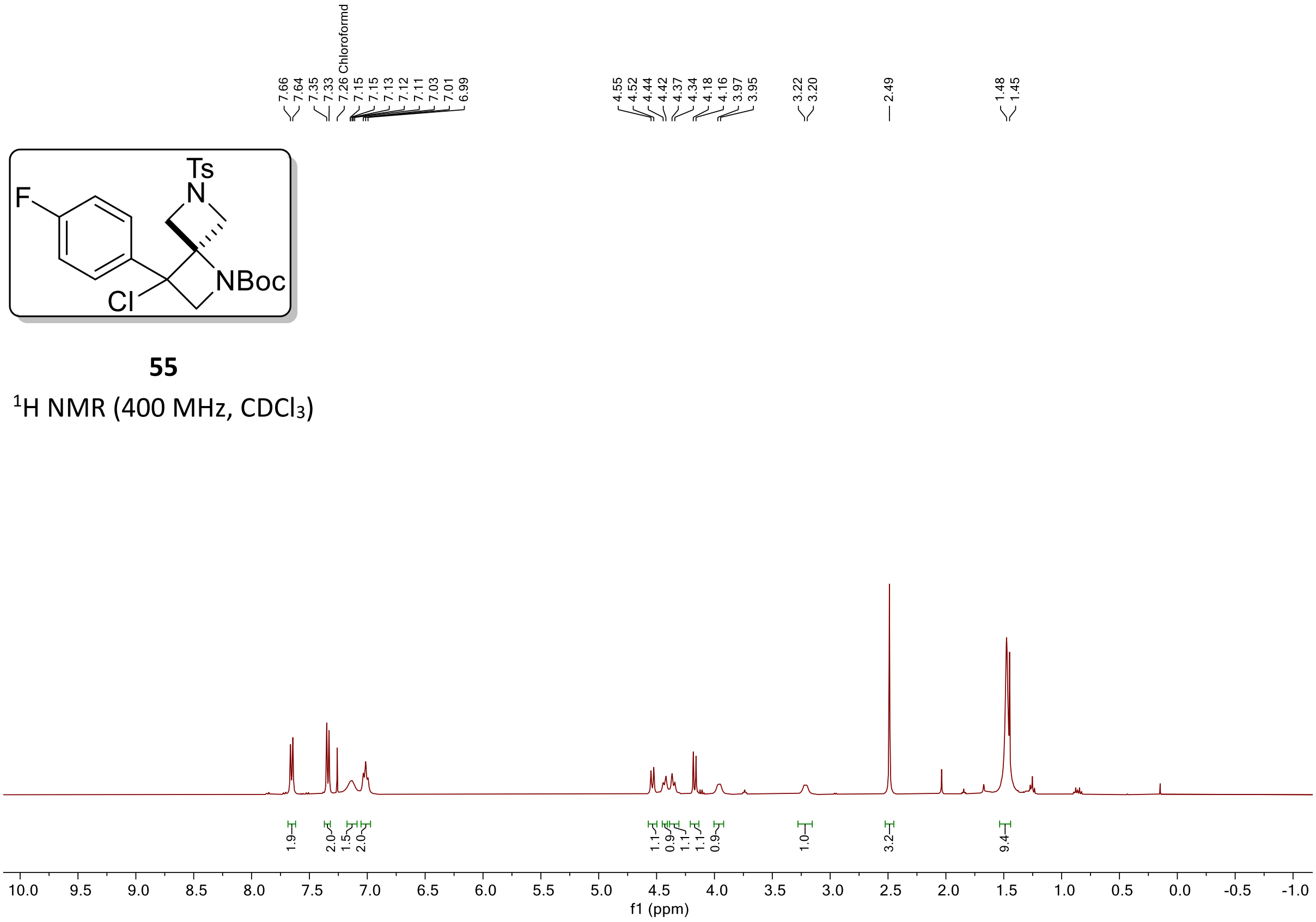


**55**

$^{1}H$ NMR (400 MHz, $CDCl_3$)

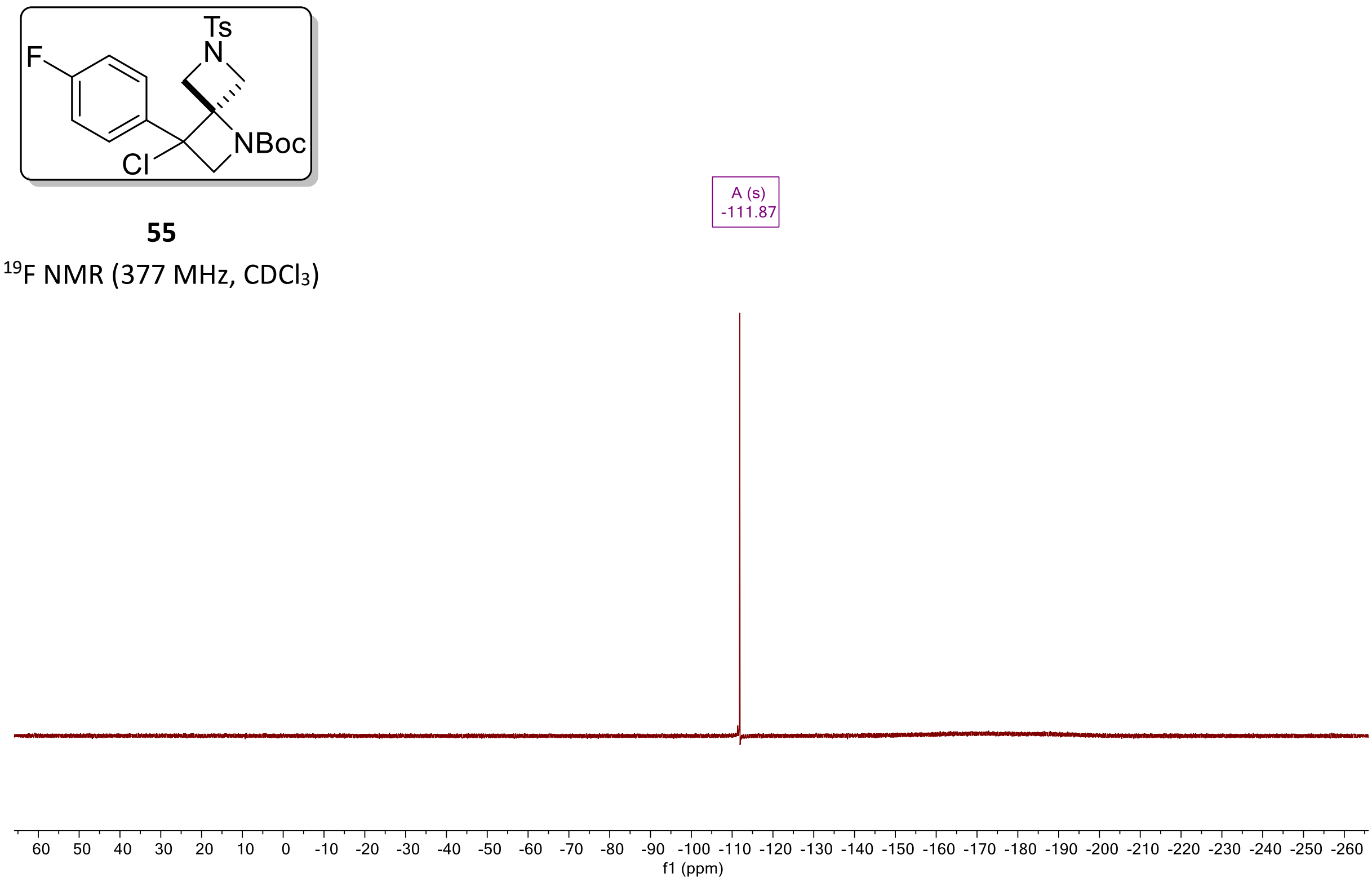


**55**

$^{19}F$ NMR (377 MHz, $CDCl_3$)

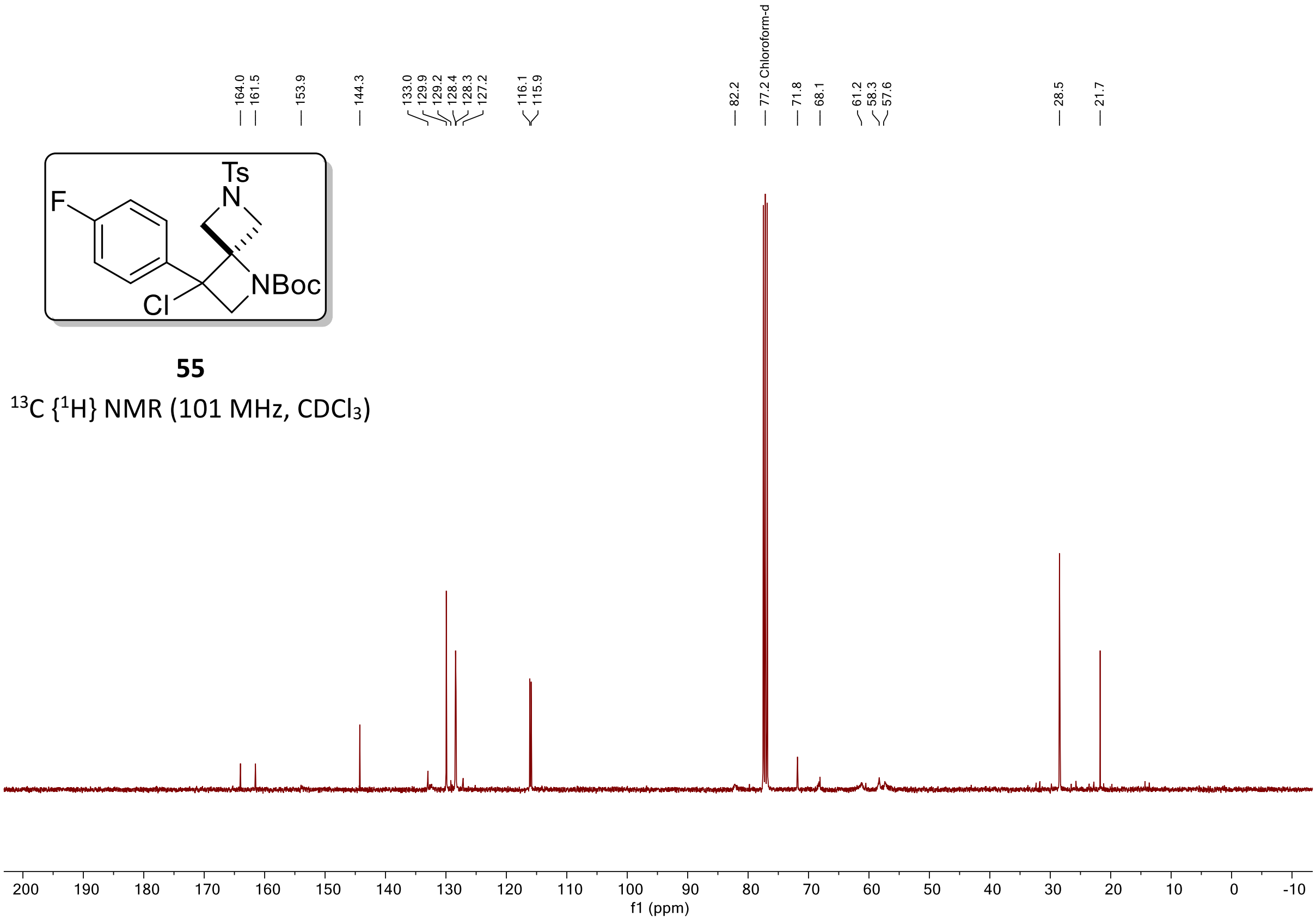


**55**

$^{13}C\{^{1}H\}$ NMR (101 MHz, $CDCl_3$)

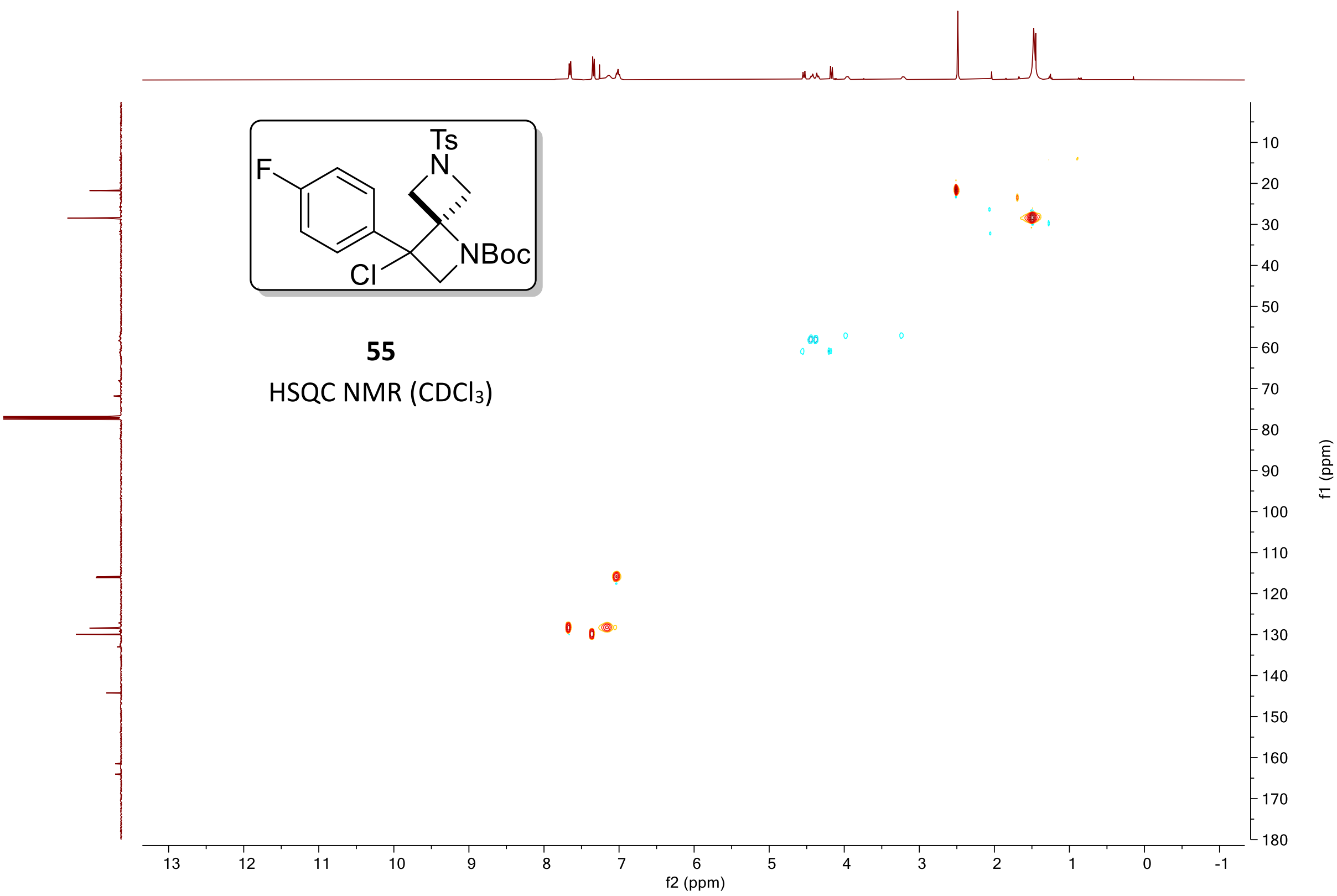


**55**

HSQC NMR ($CDCl_3$)

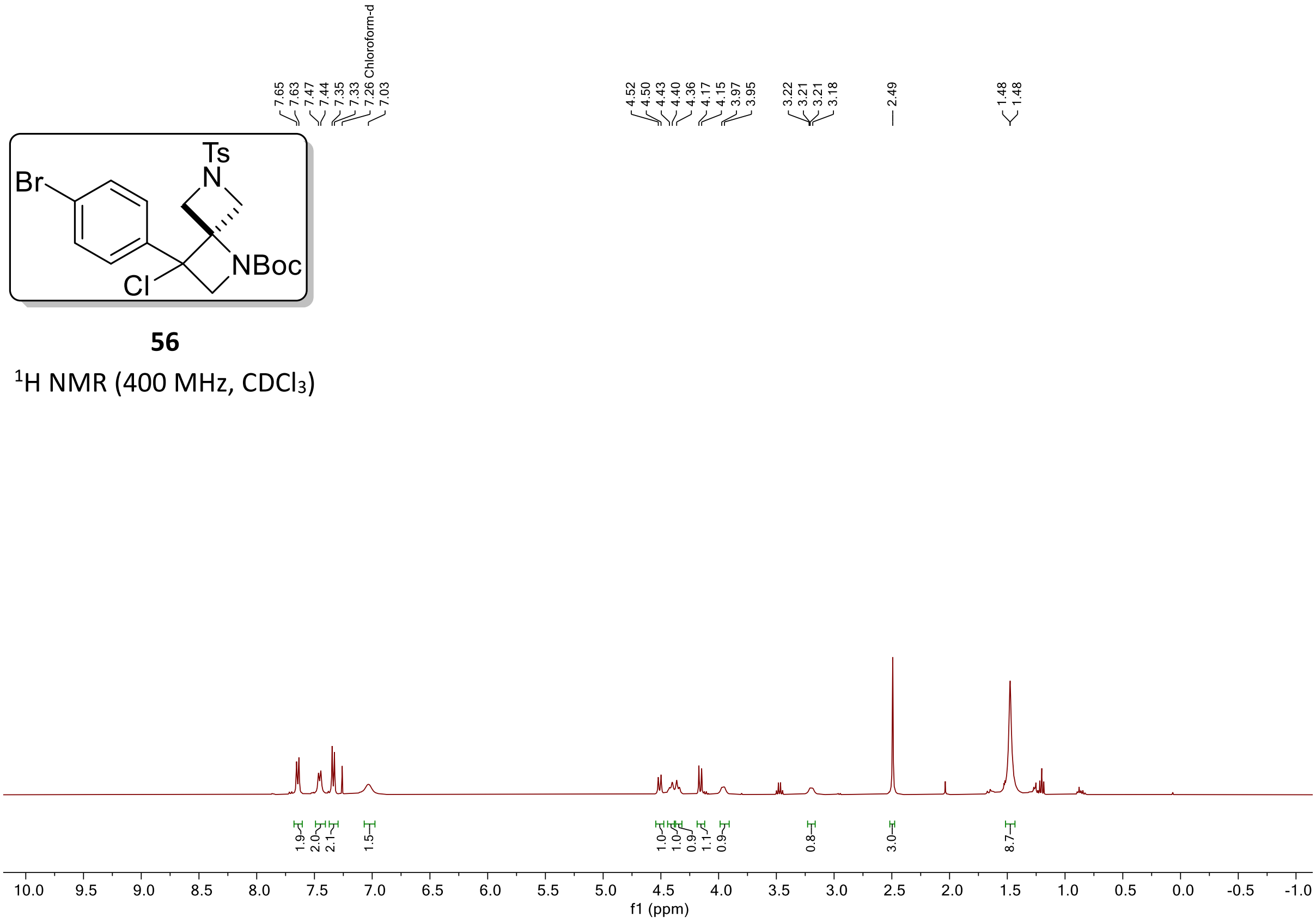

**56**

$^1$H NMR (400 MHz, $CDCl_3$)

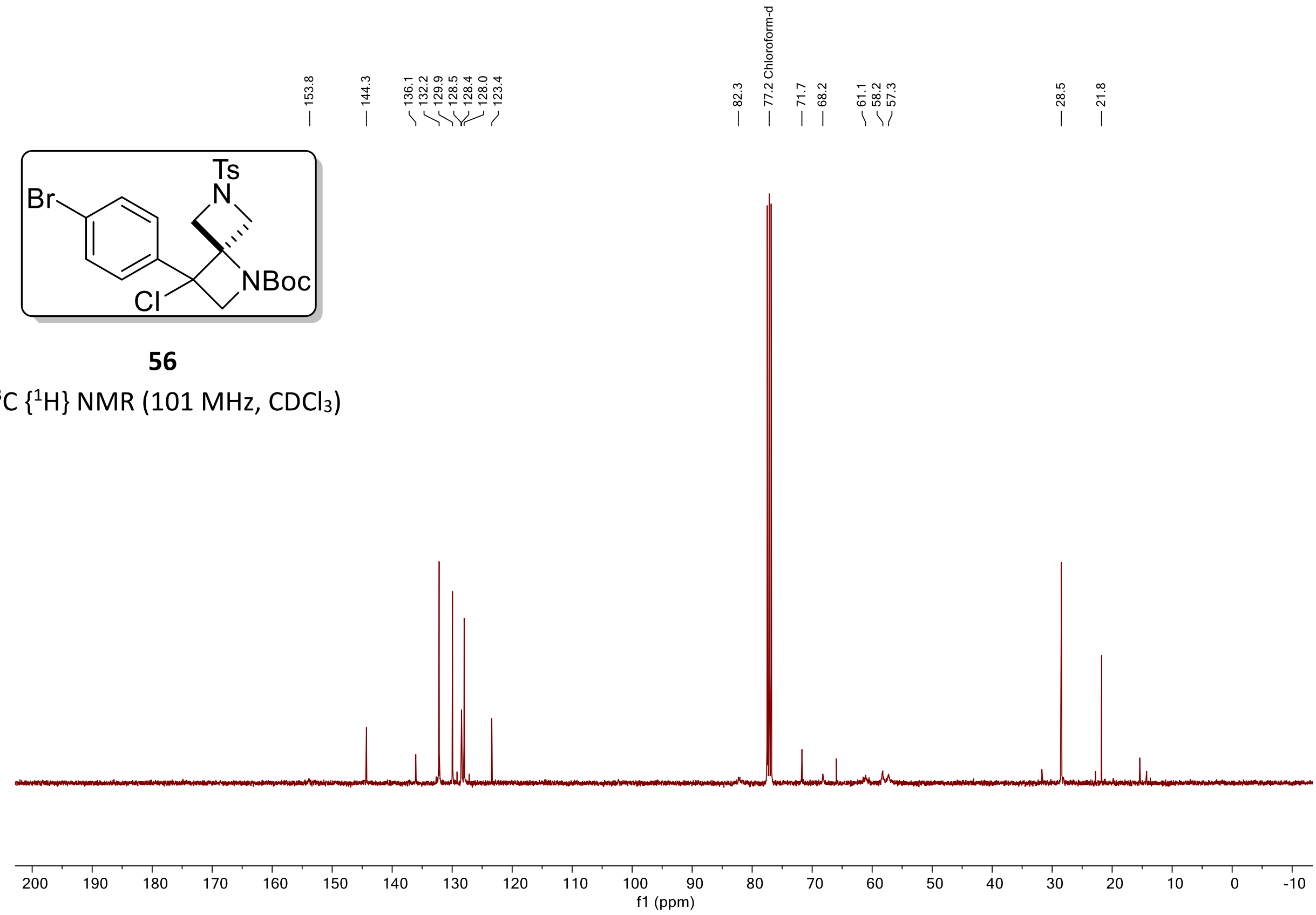

**56**

$^{13}C\{^1H\}$ NMR (101 MHz, $CDCl_3$)

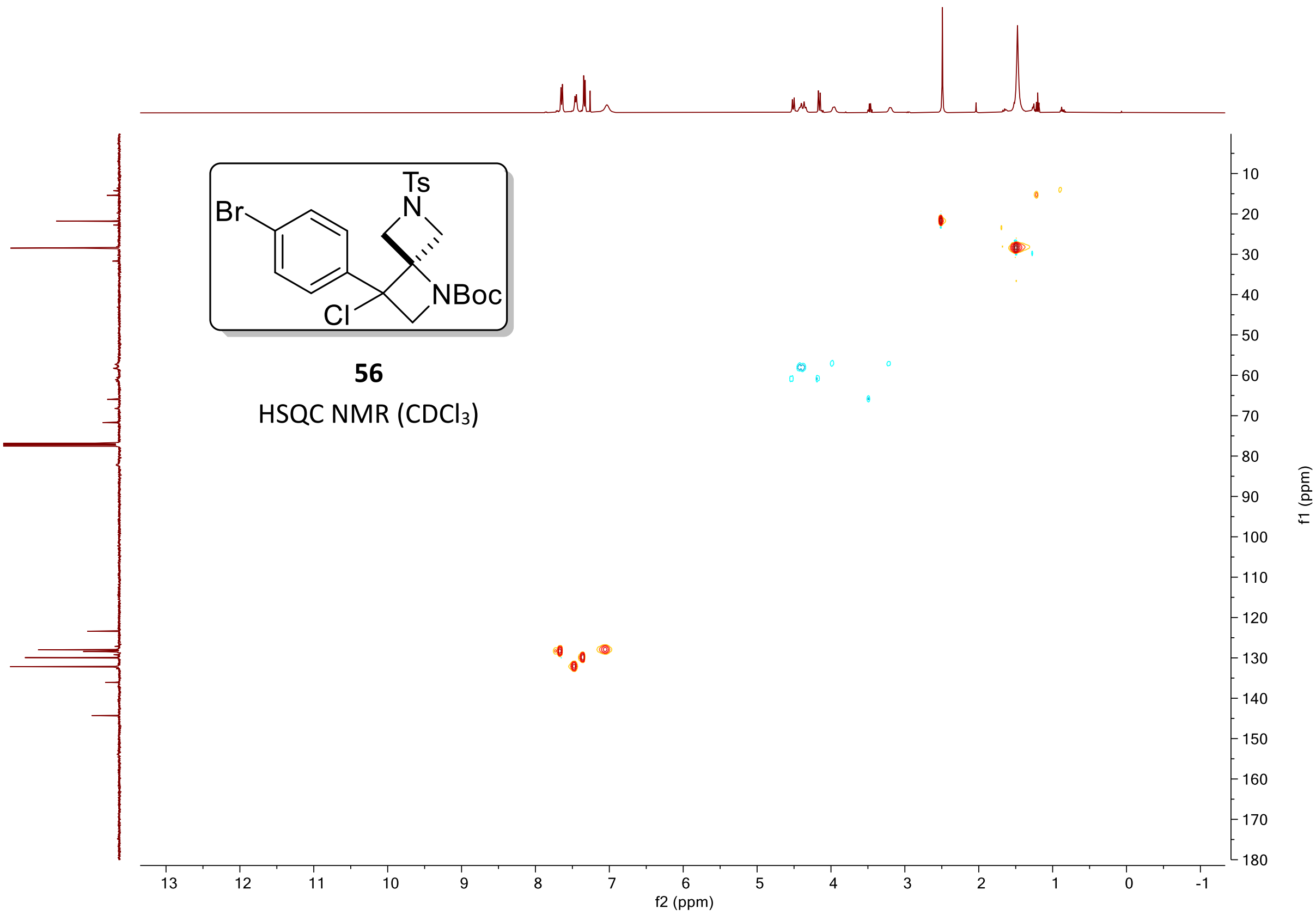


**56**

HSQC NMR ($CDCl_3$)

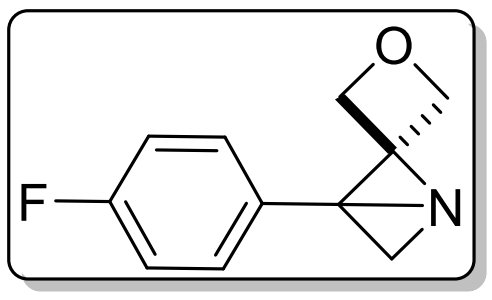

**Crude, 5**

$^{1}$H NMR (400 MHz, $CDCl_3$)

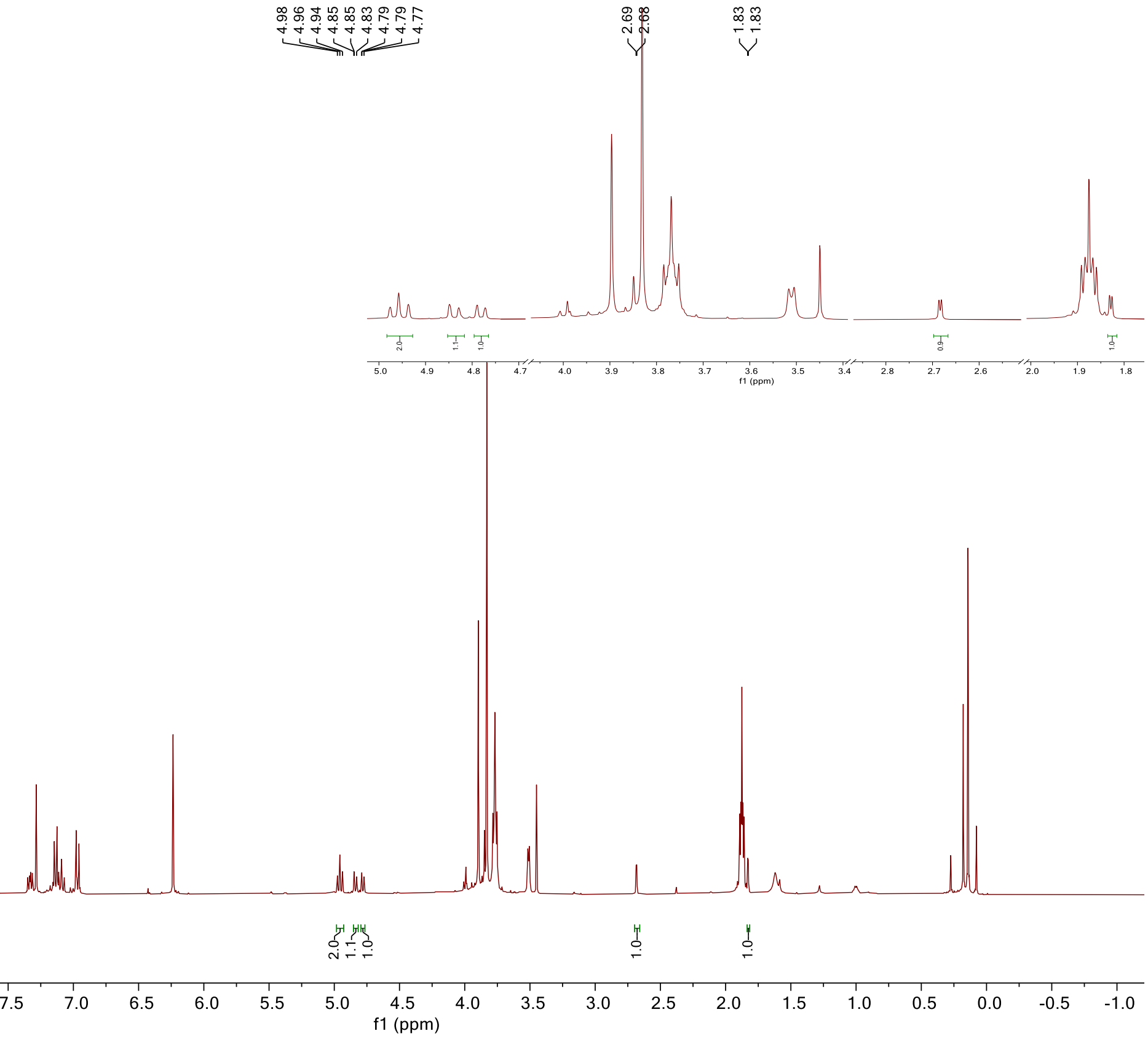

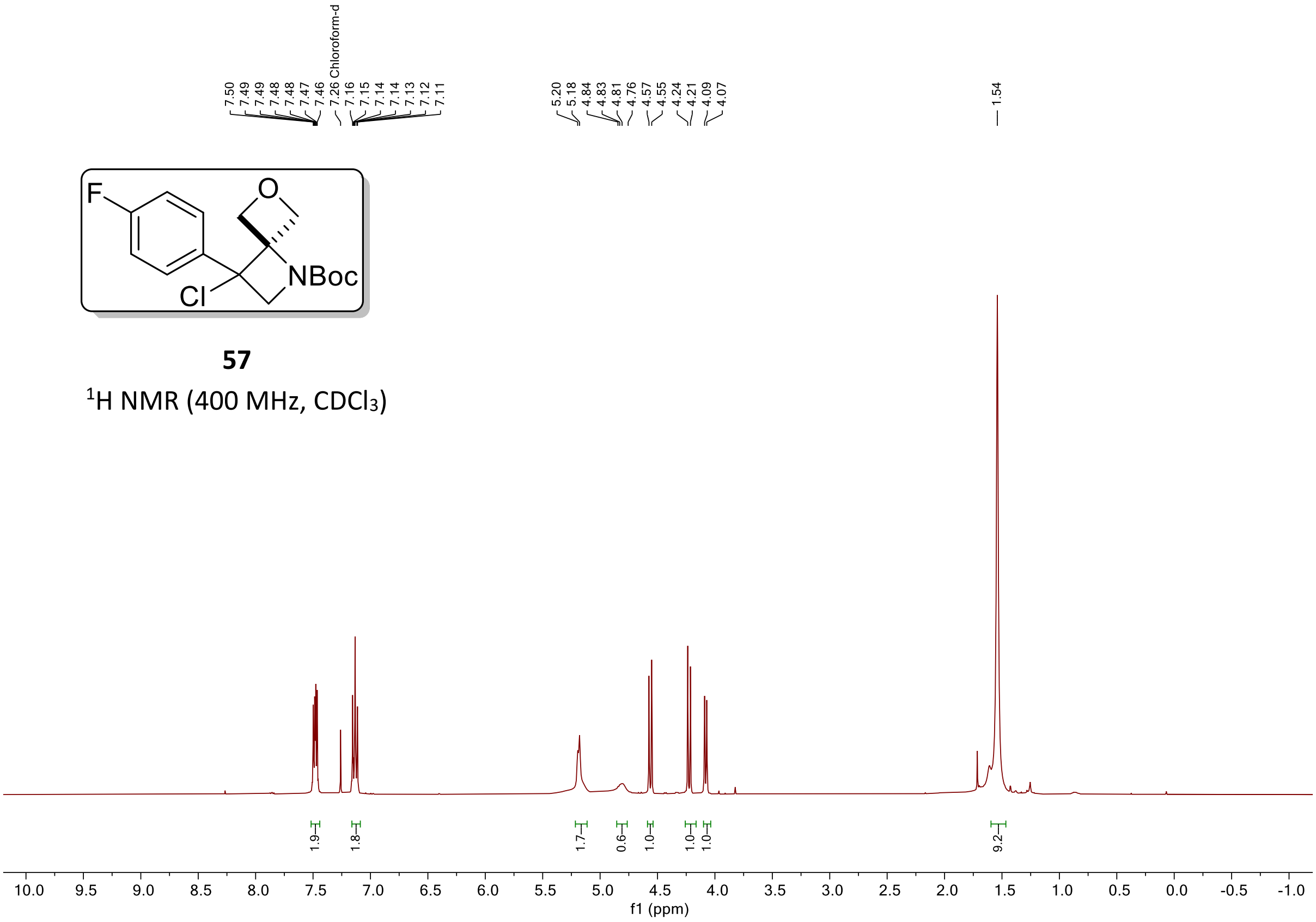


**57**

$^{1}H$ NMR (400 MHz, $CDCl_3$)

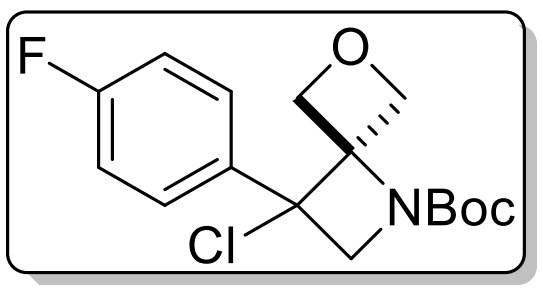


**57**

$^{19}F$ NMR (377 MHz, $CDCl_3$)

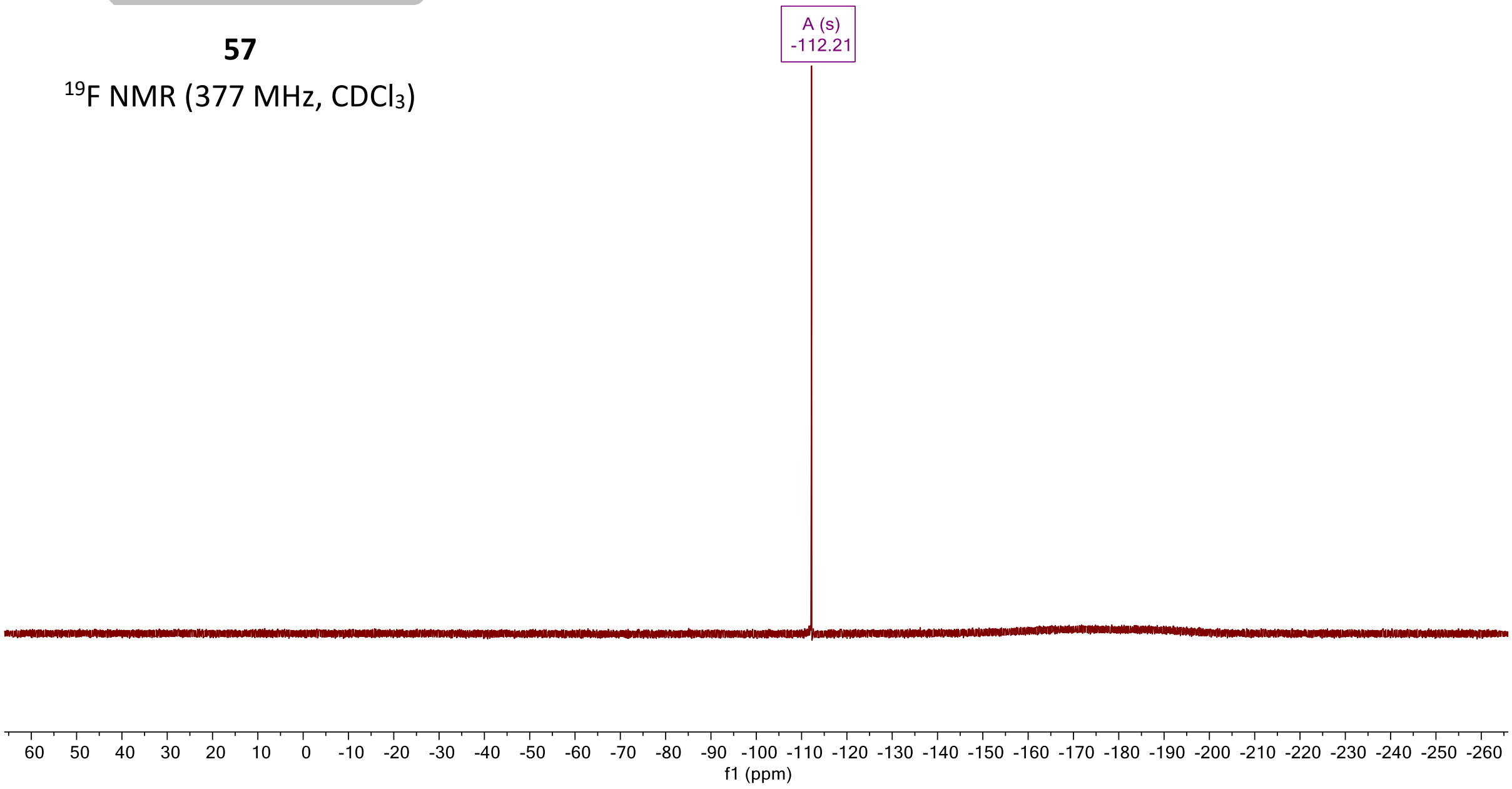

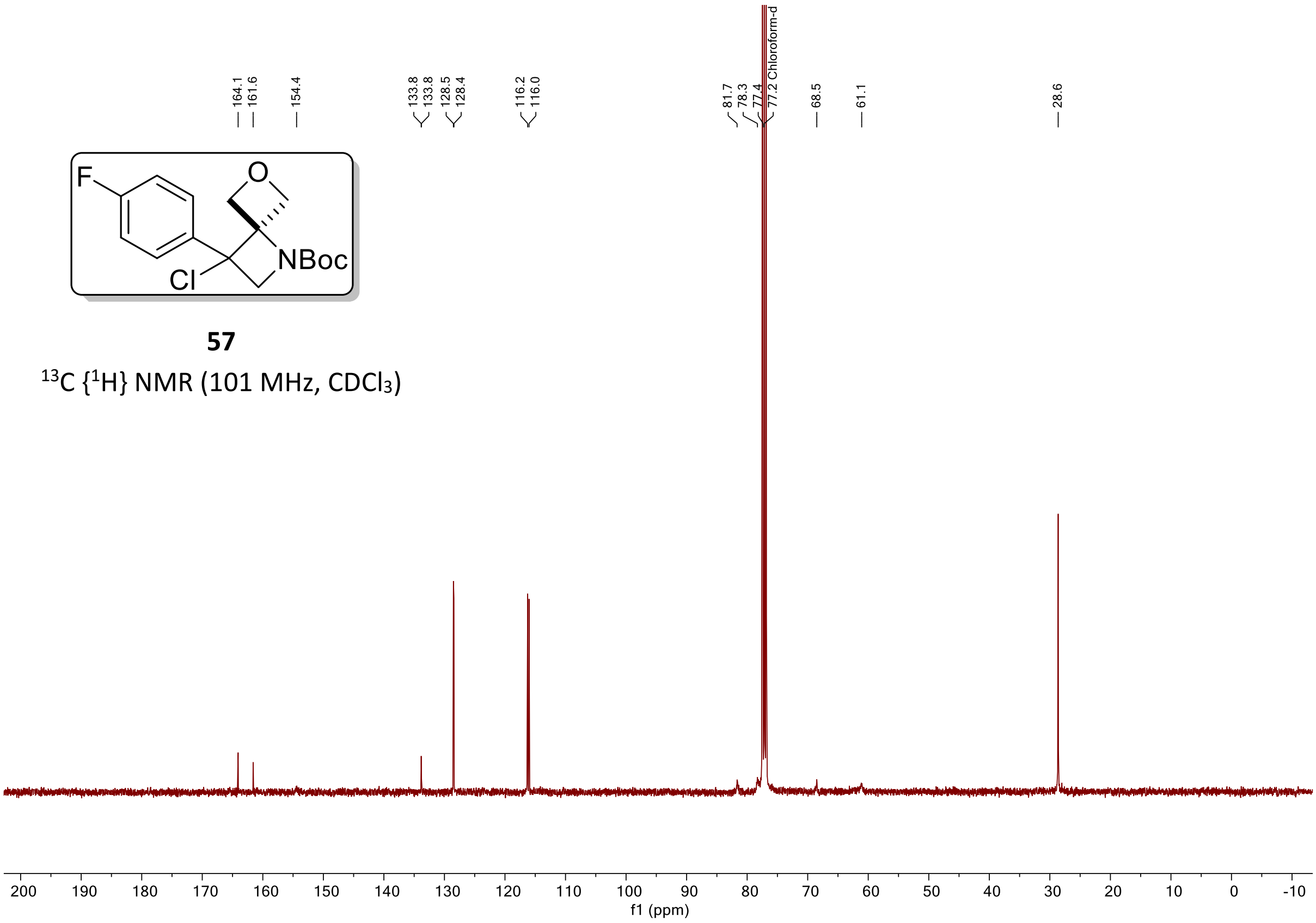
164.1
161.6
154.4
133.8
133.8
128.5
128.4
116.2
116.0
81.7
78.3
77.4
77.2 Chloroform-d
68.5
61.1
28.6
F
O
NBoc
Cl
57
$^{13}C$ {$^{1}H$} NMR (101 MHz, $CDCl_3$)
f1 (ppm)

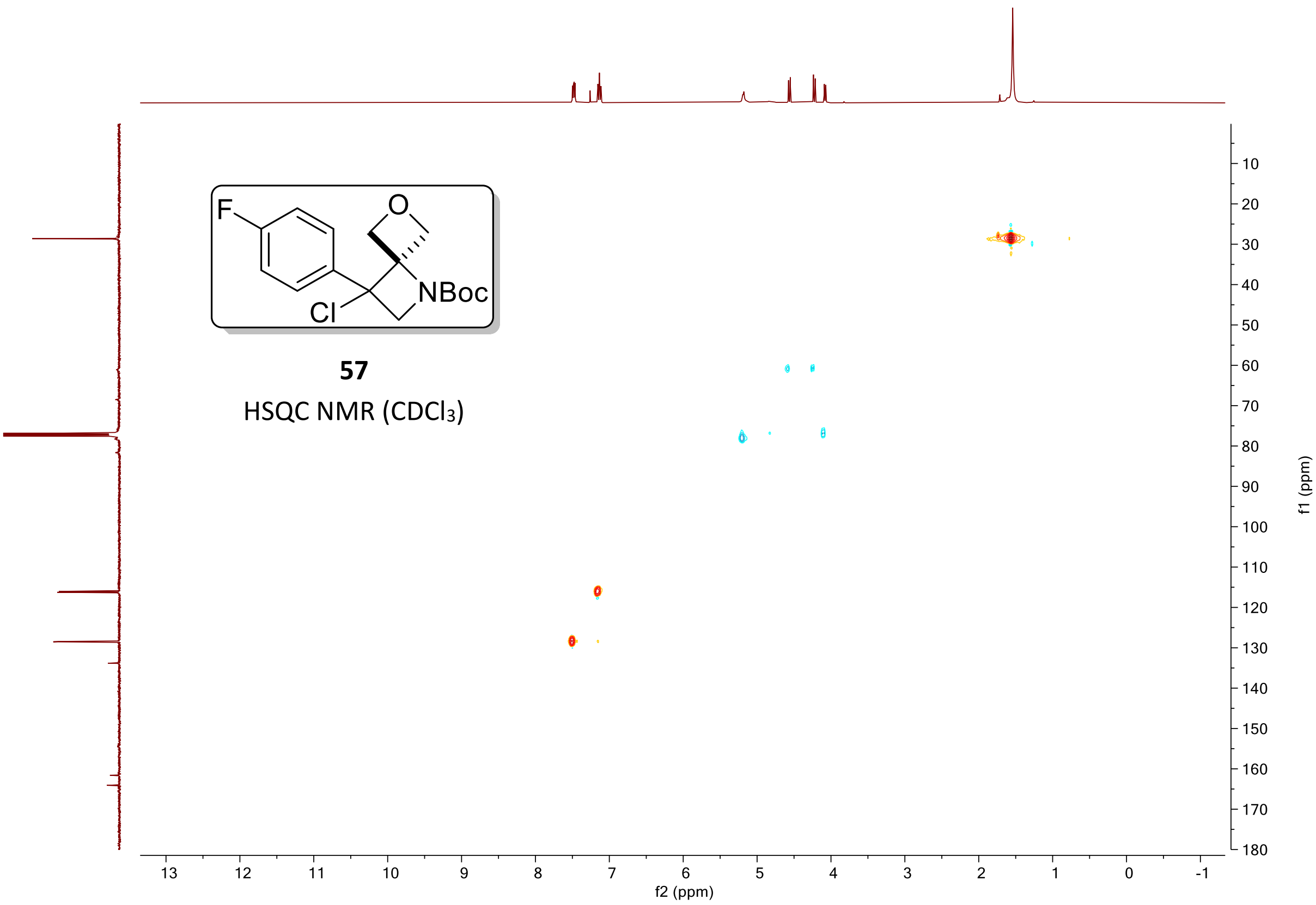
F
O
NBoc
Cl
57
HSQC NMR ($CDCl_3$)
f2 (ppm)
f1 (ppm)

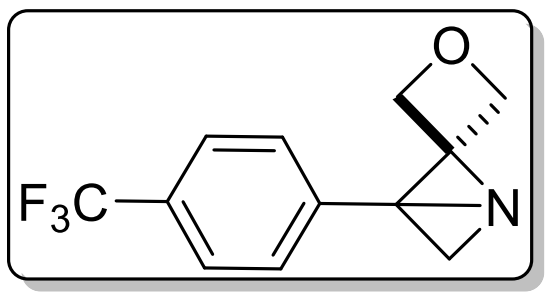

**Crude, 35**

$^{1}H$ NMR (400 MHz, $CDCl_3$)

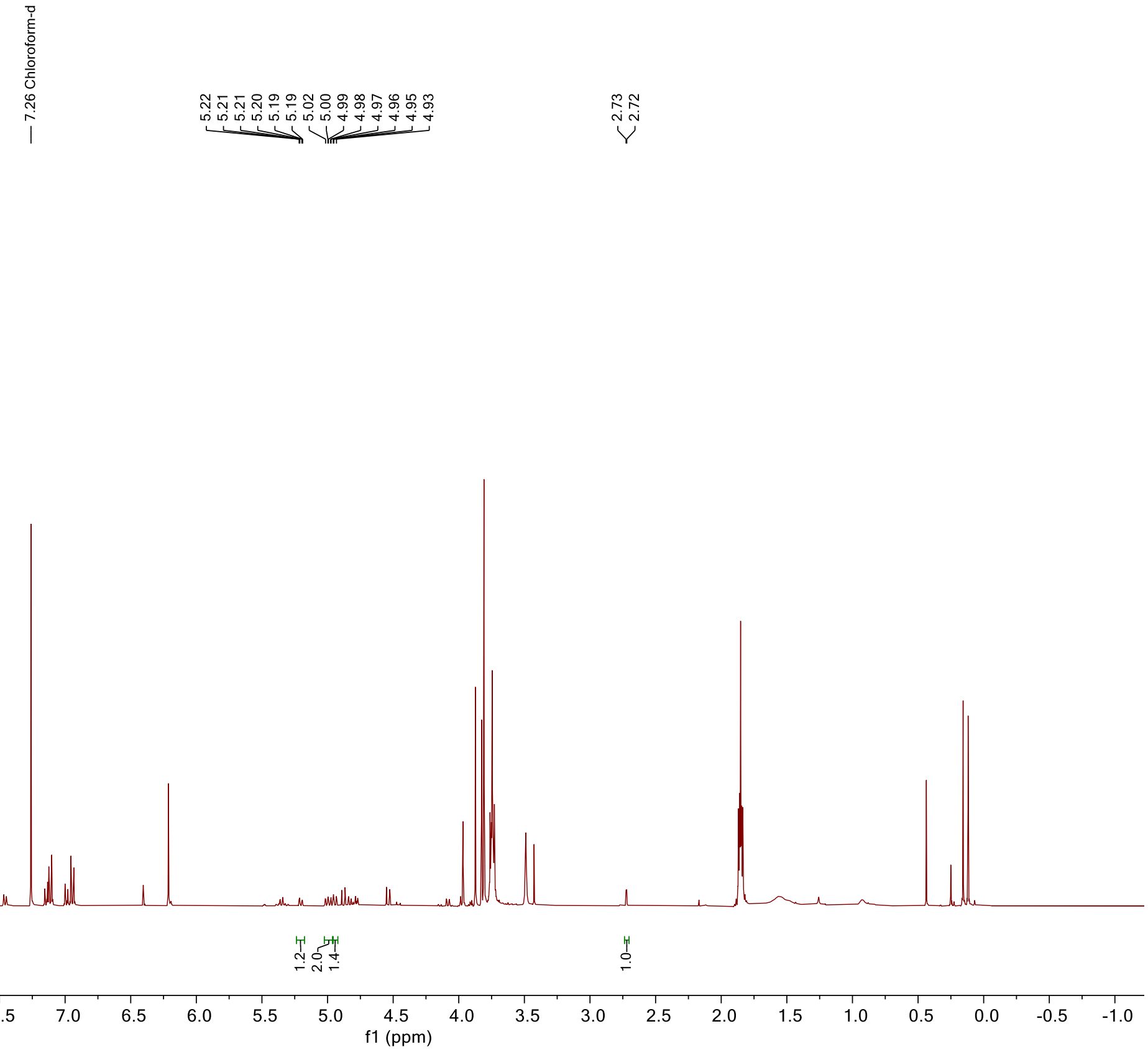

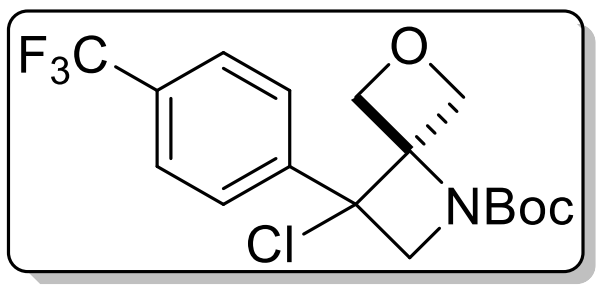

**58**

$^{1}H$ NMR (400 MHz, $CDCl_3$)

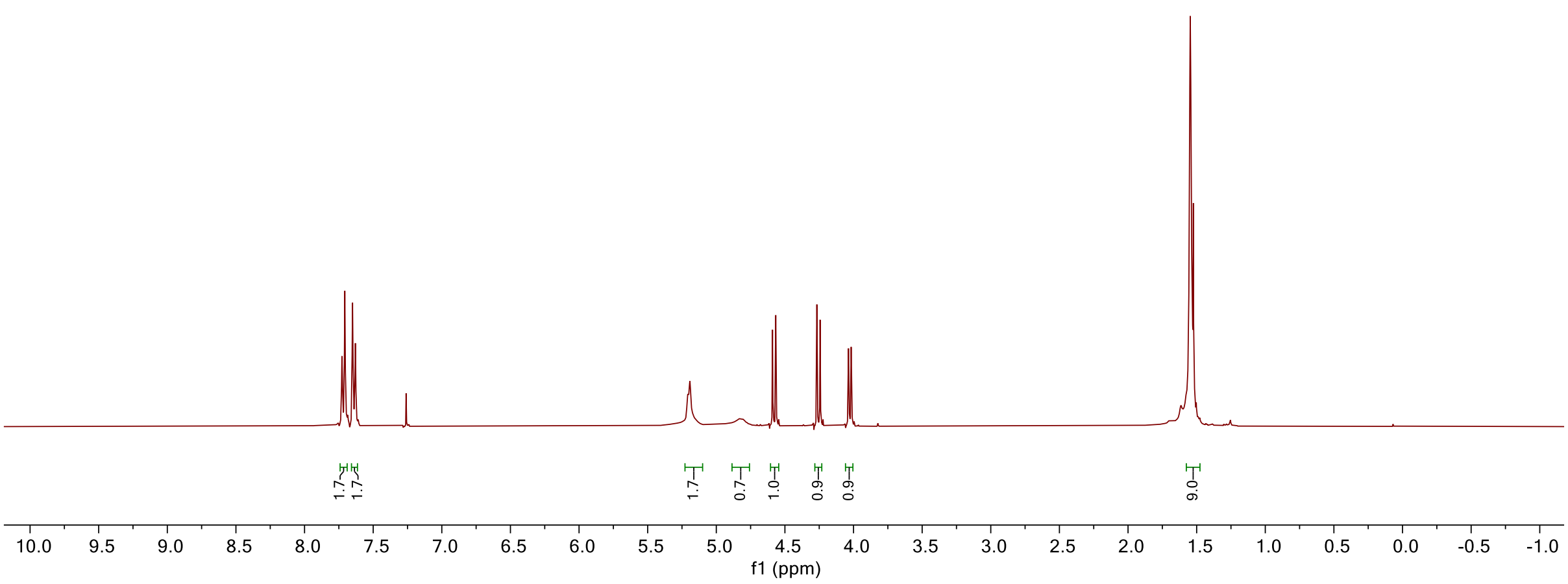


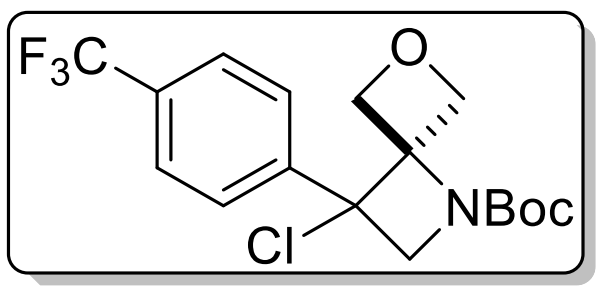

**58**

$^{19}F$ NMR (377 MHz, $CDCl_3$)

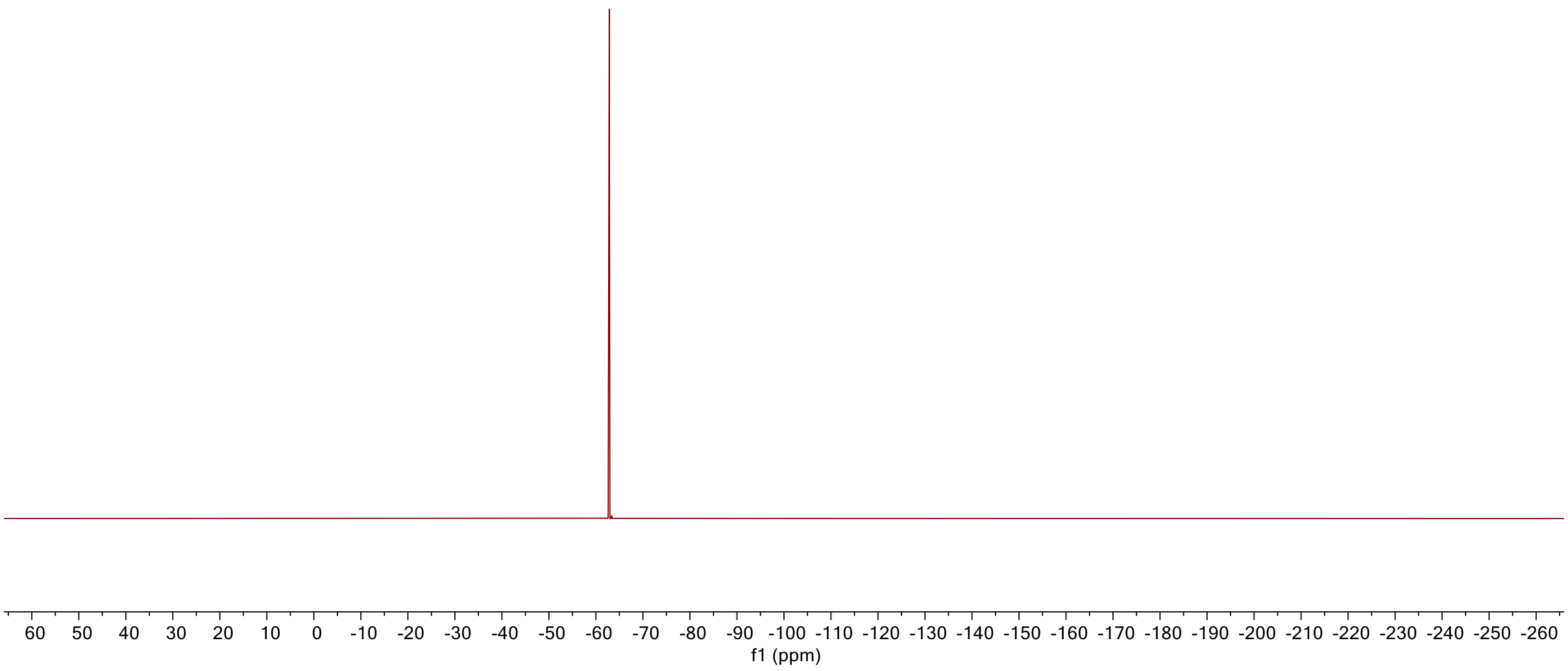

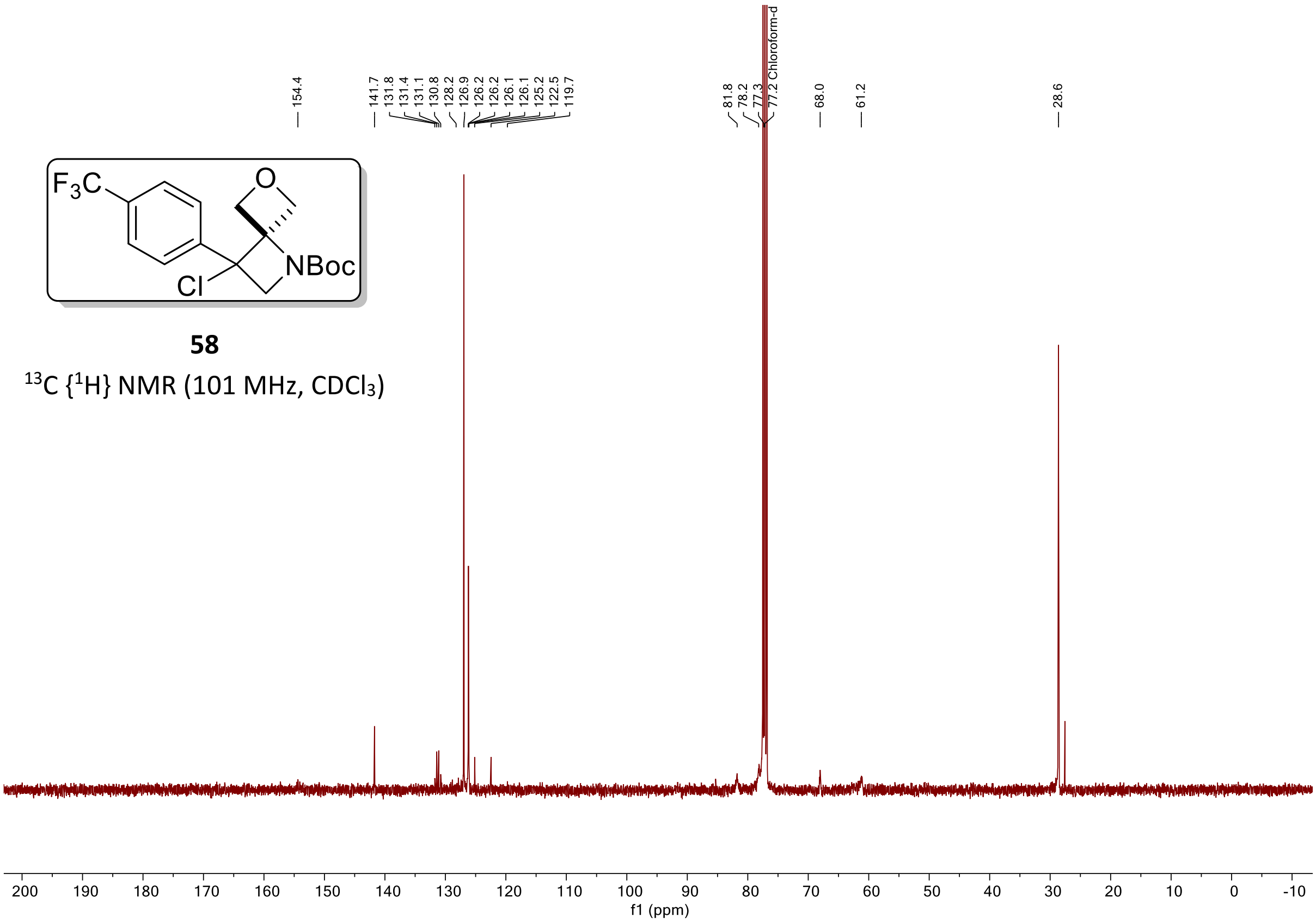


**58**

$^{13}C\ \{^1H\}$ NMR (101 MHz, $CDCl_3$)

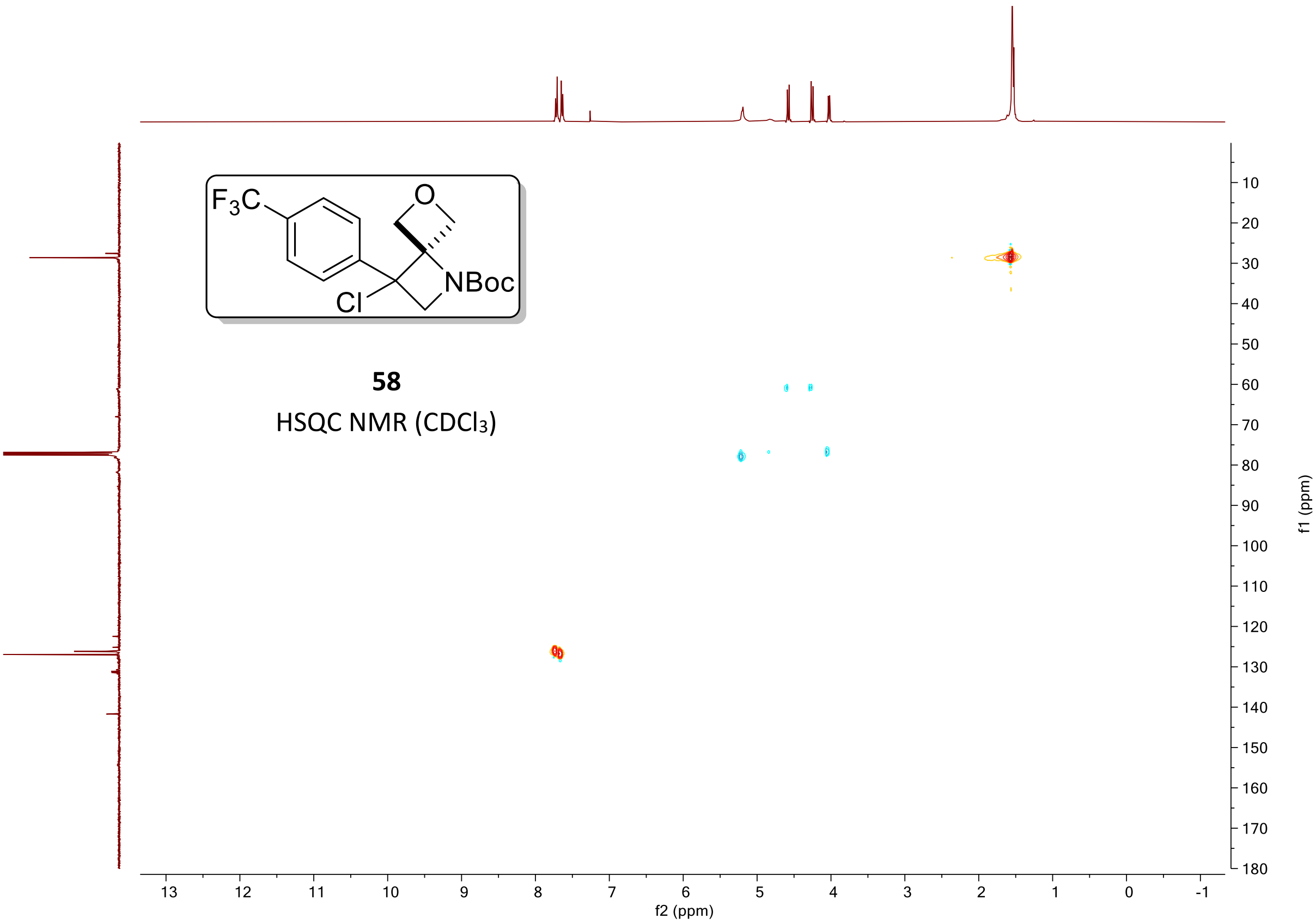


**58**

HSQC NMR ($CDCl_3$)

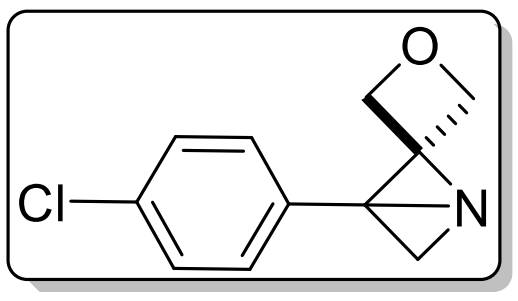

**Crude 36**

$^1H$ NMR (400 MHz, $CDCl_3$)

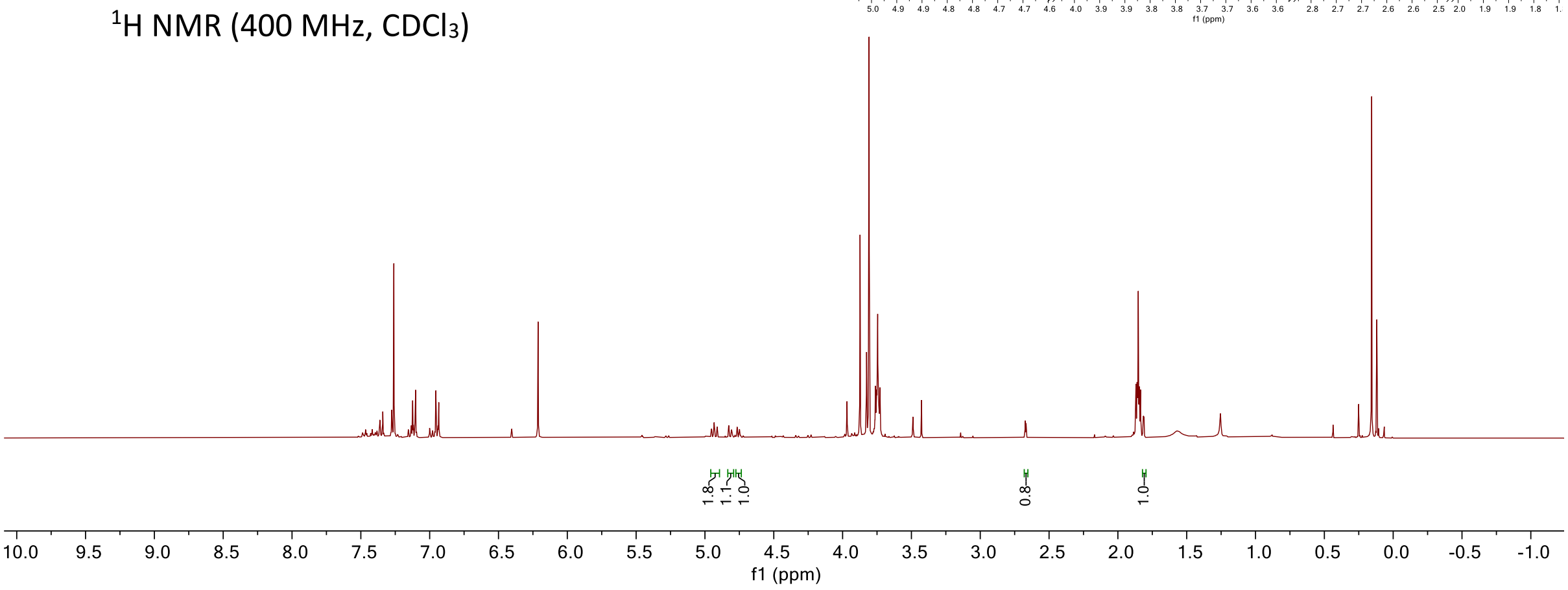

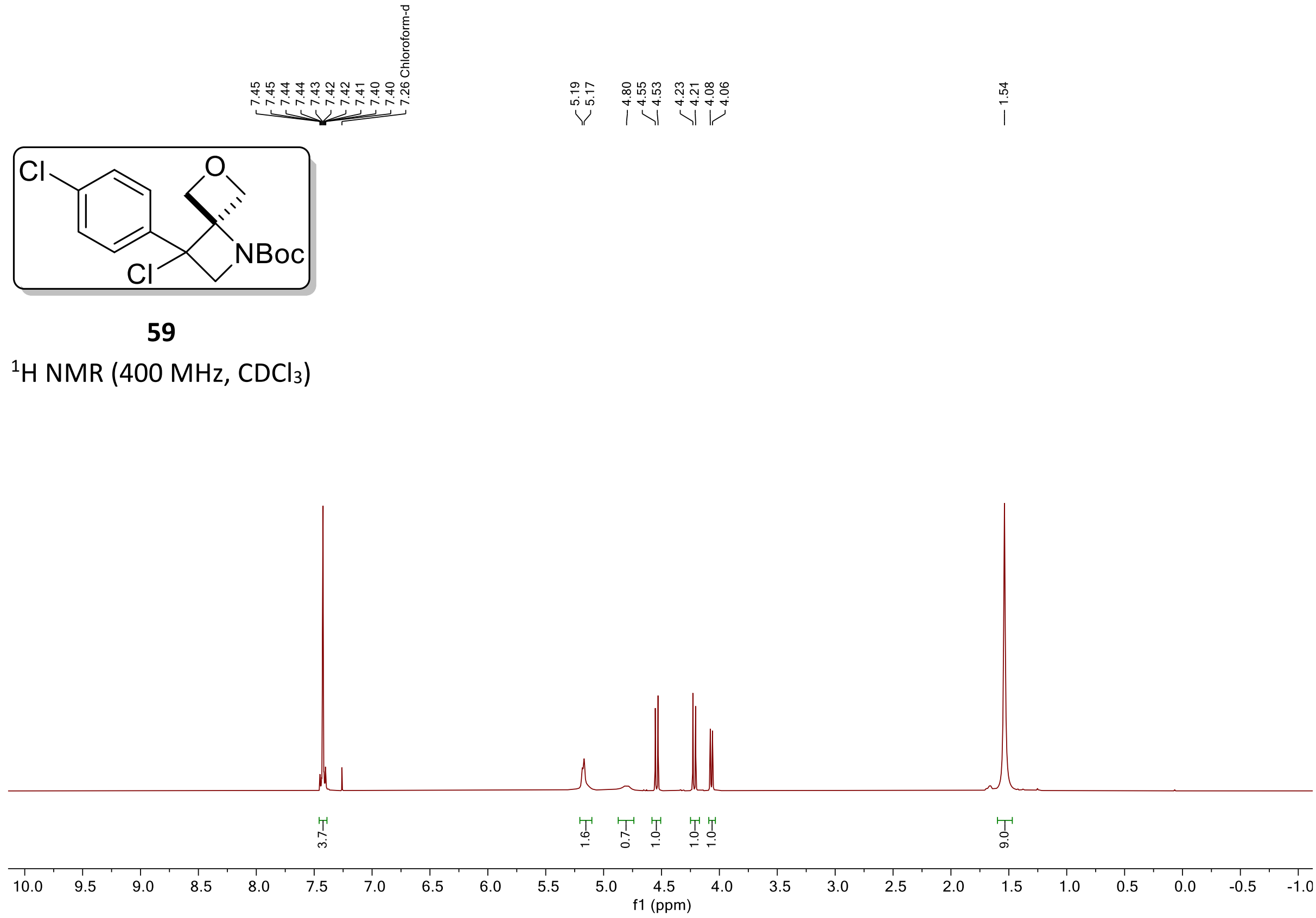


**59**

$^{1}H$ NMR (400 MHz, $CDCl_3$)

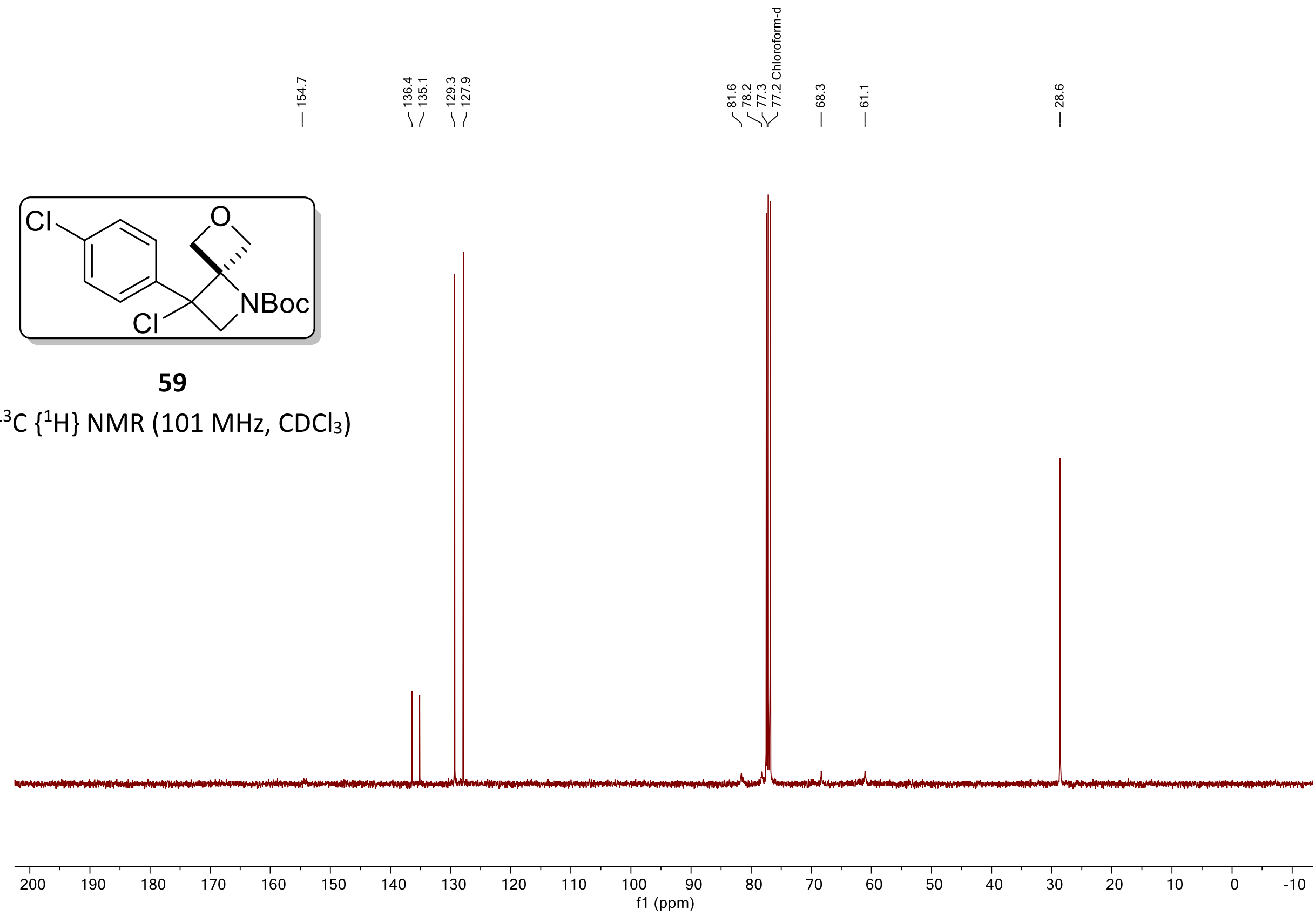


**59**

$^{13}C\{^{1}H\}$ NMR (101 MHz, $CDCl_3$)

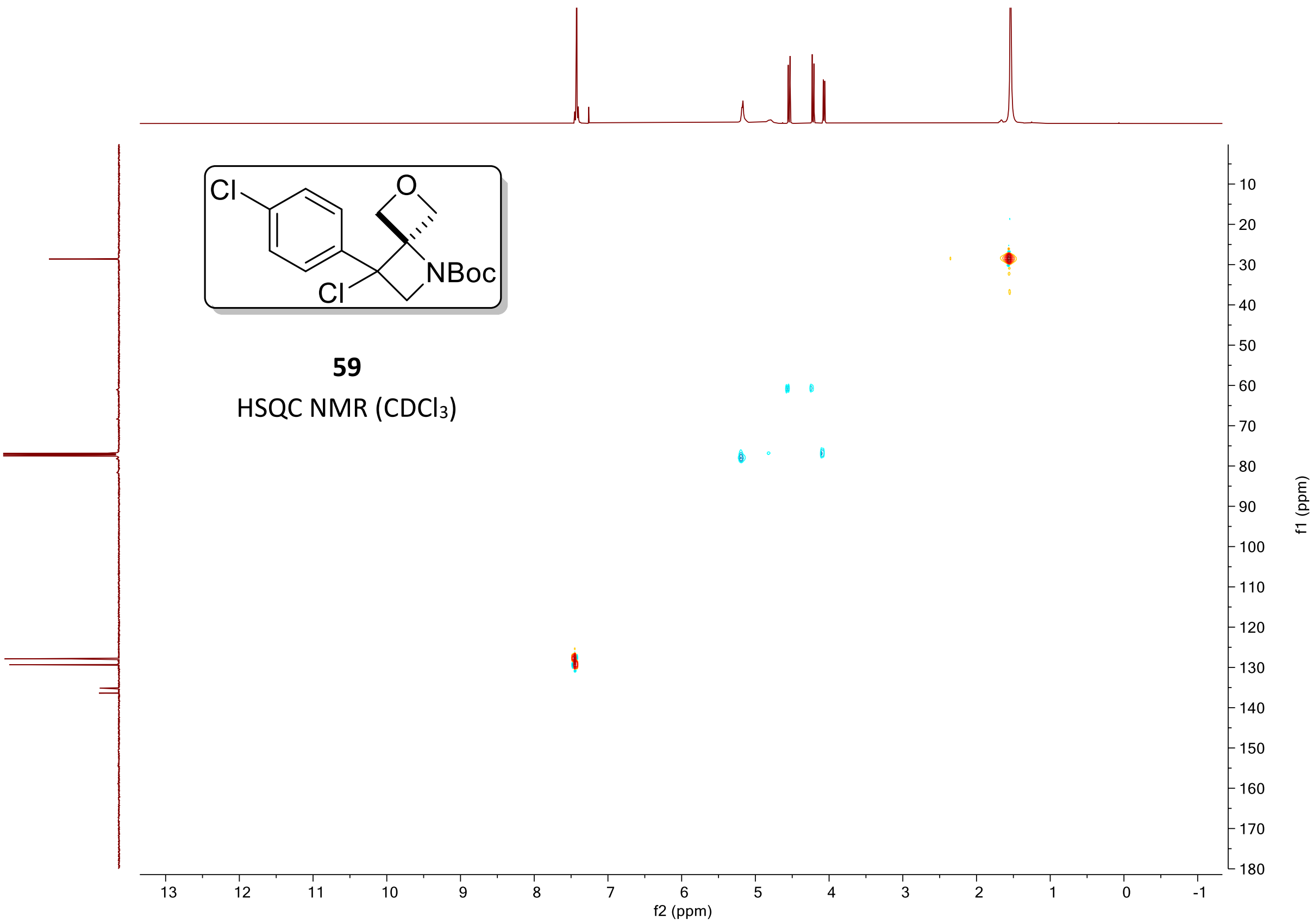


**59**

HSQC NMR ($CDCl_3$)

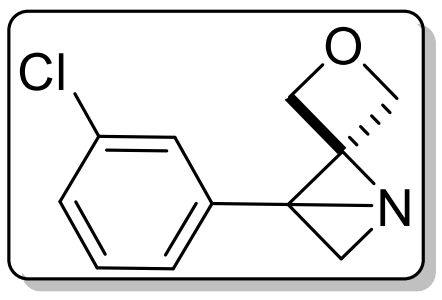

**Crude, 50**

$^{1}H$ NMR (400 MHz, $CDCl_3$)

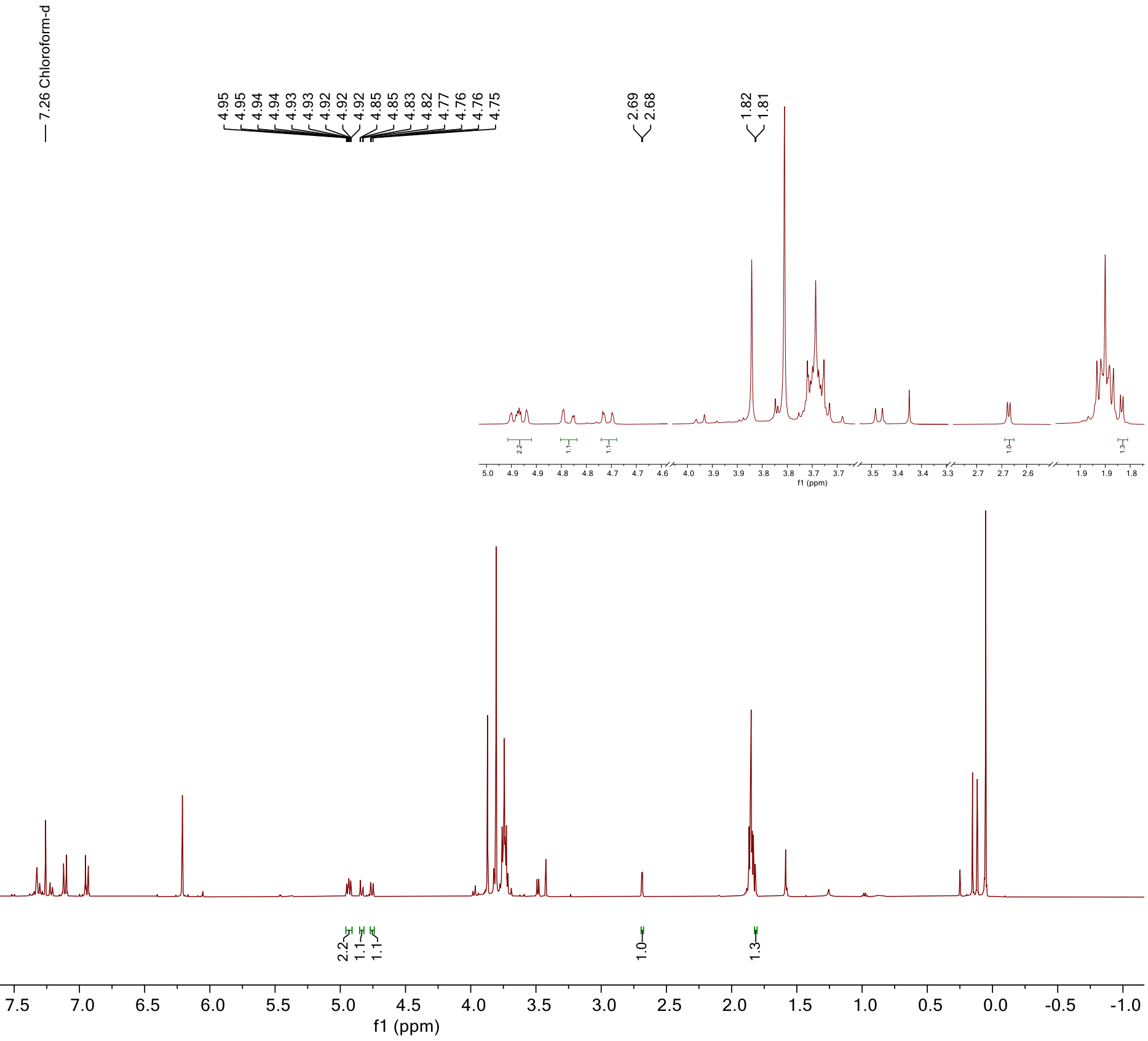

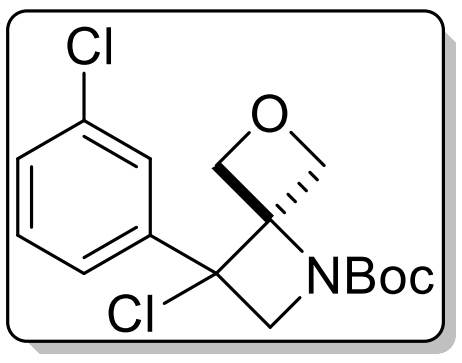

**60**

$^{1}H$ NMR (400 MHz, $CDCl_3$)

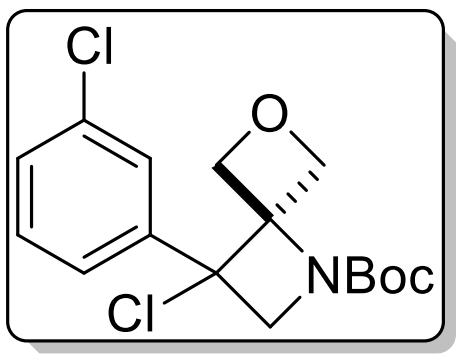

**60**

$^{13}C$ {$^{1}H$} NMR (101 MHz, $CDCl_3$)

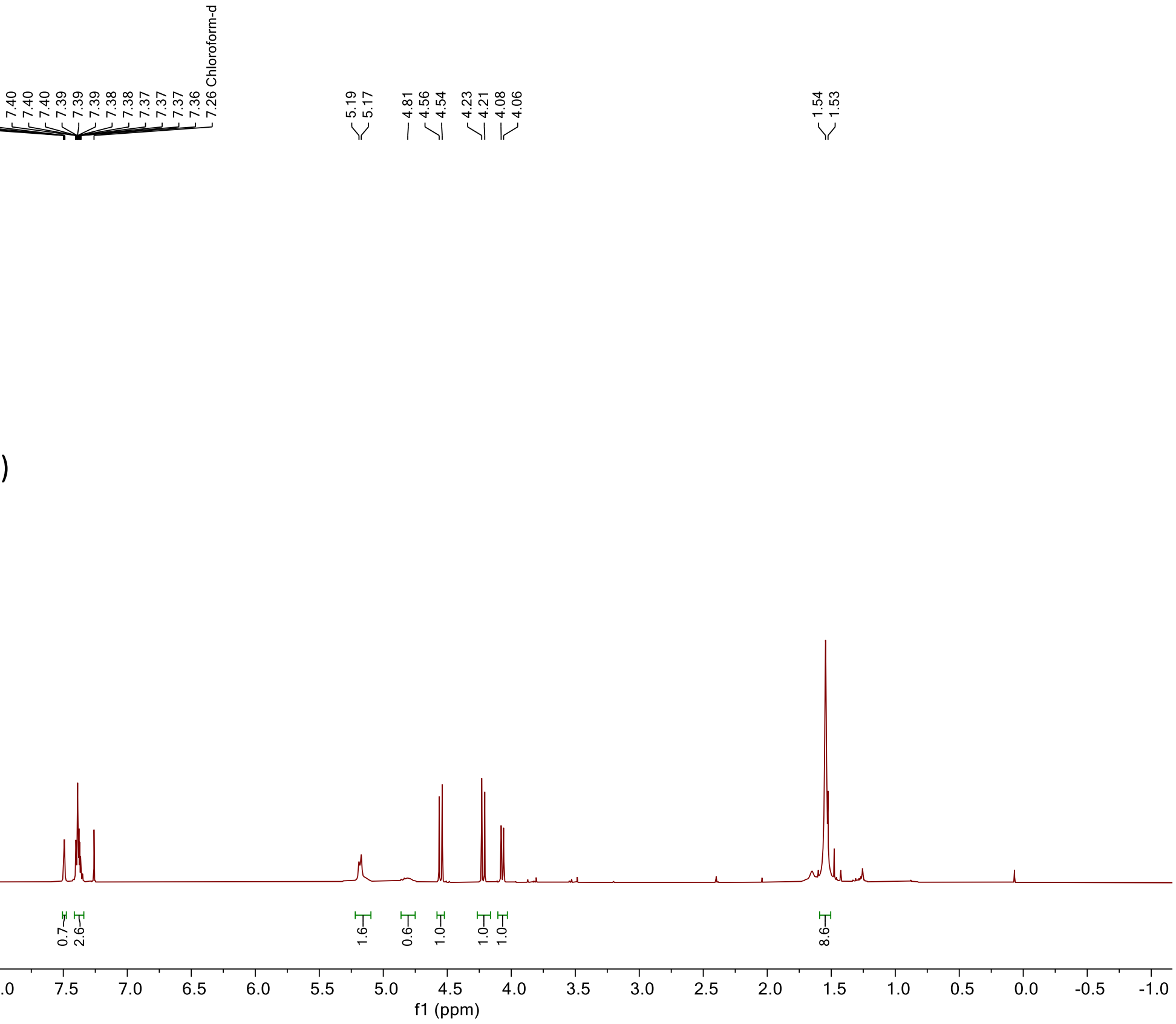


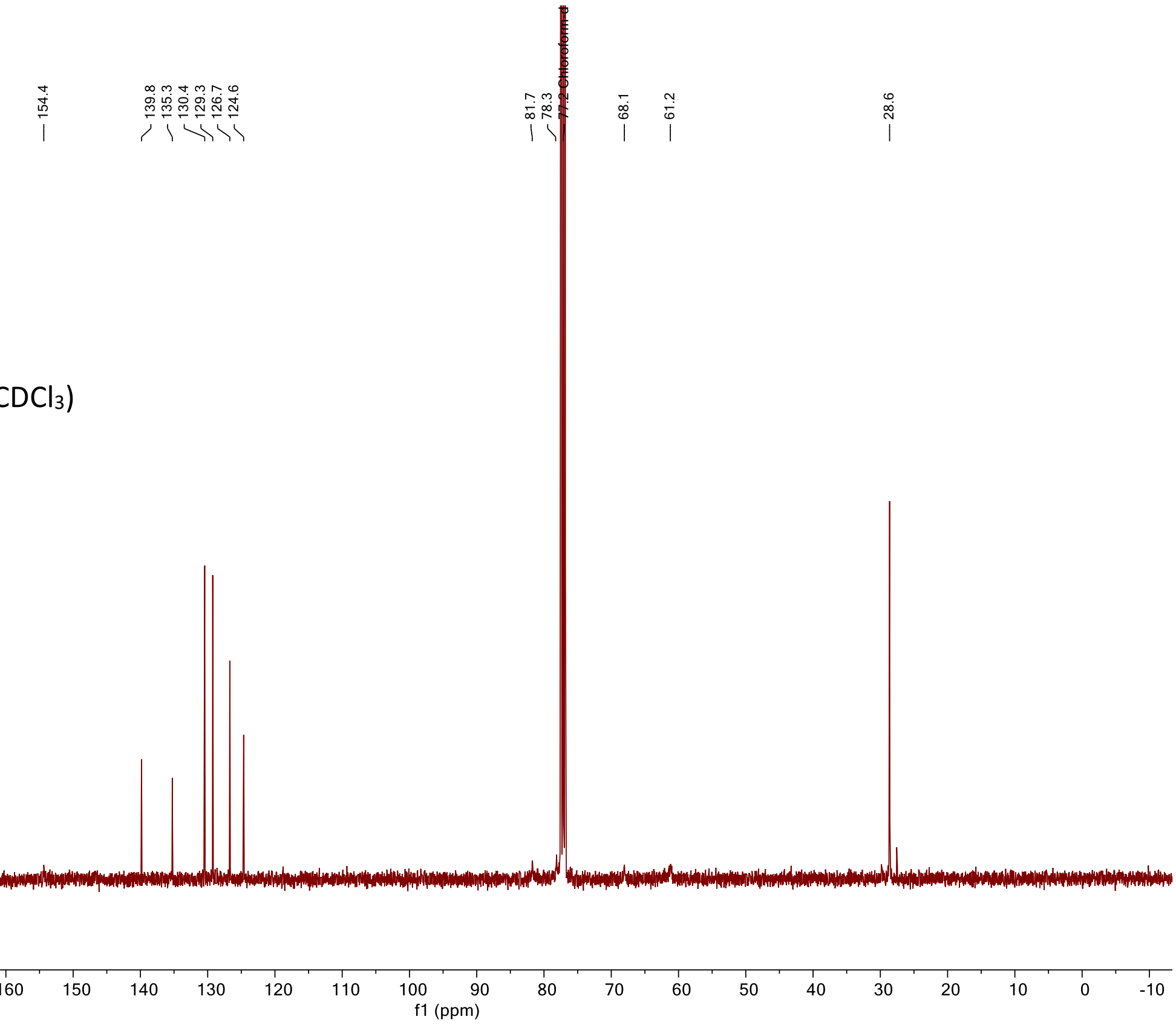

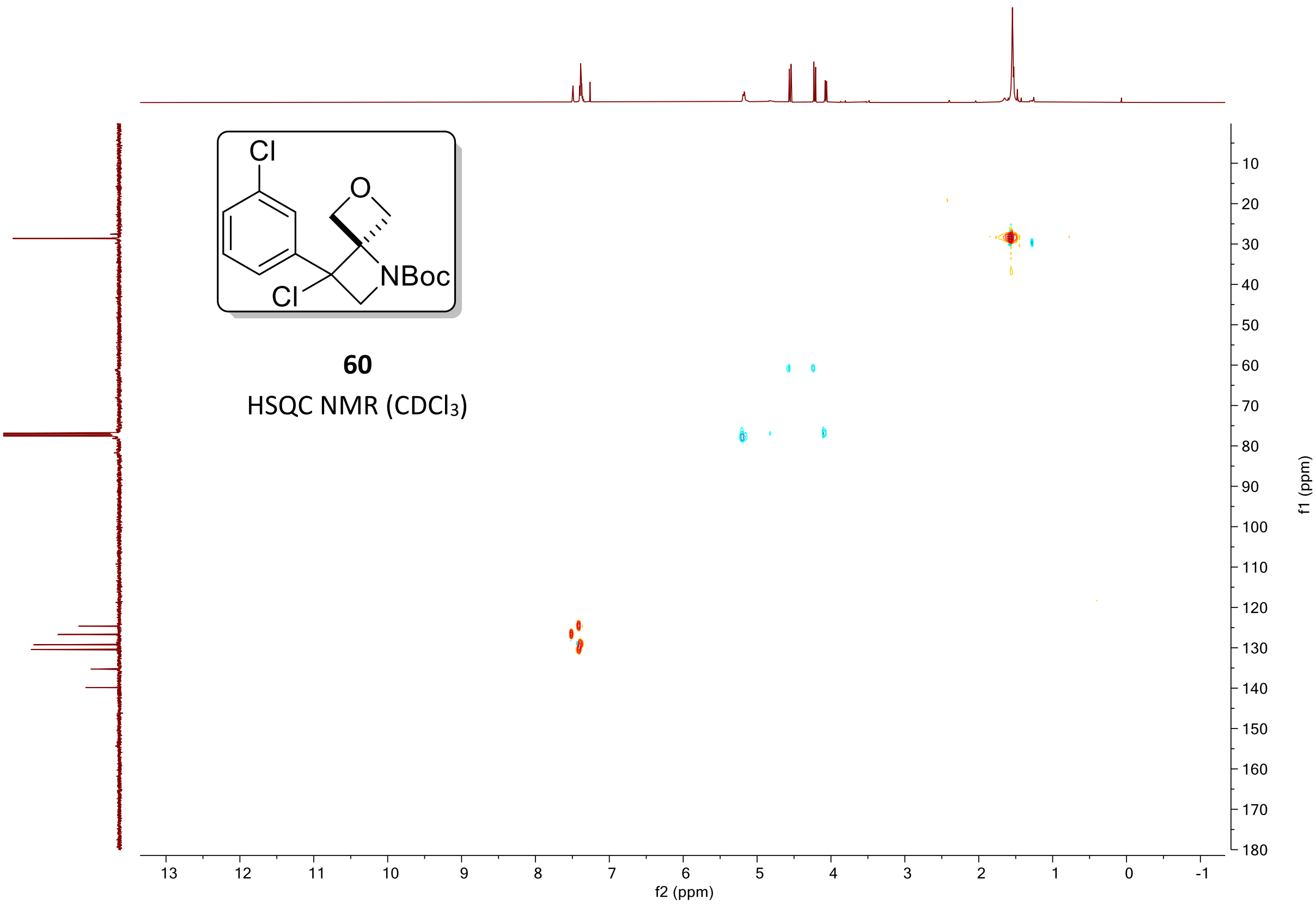


**60**

HSQC NMR ($CDCl_3$)

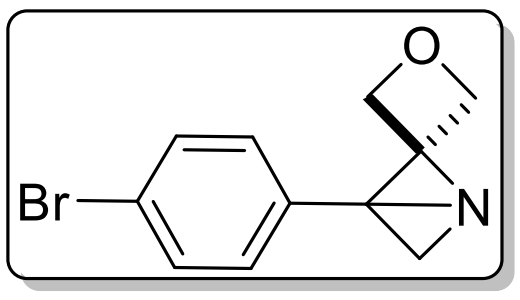

**Crude, 51**

$^{1}H$ NMR (400 MHz, $CDCl_3$)

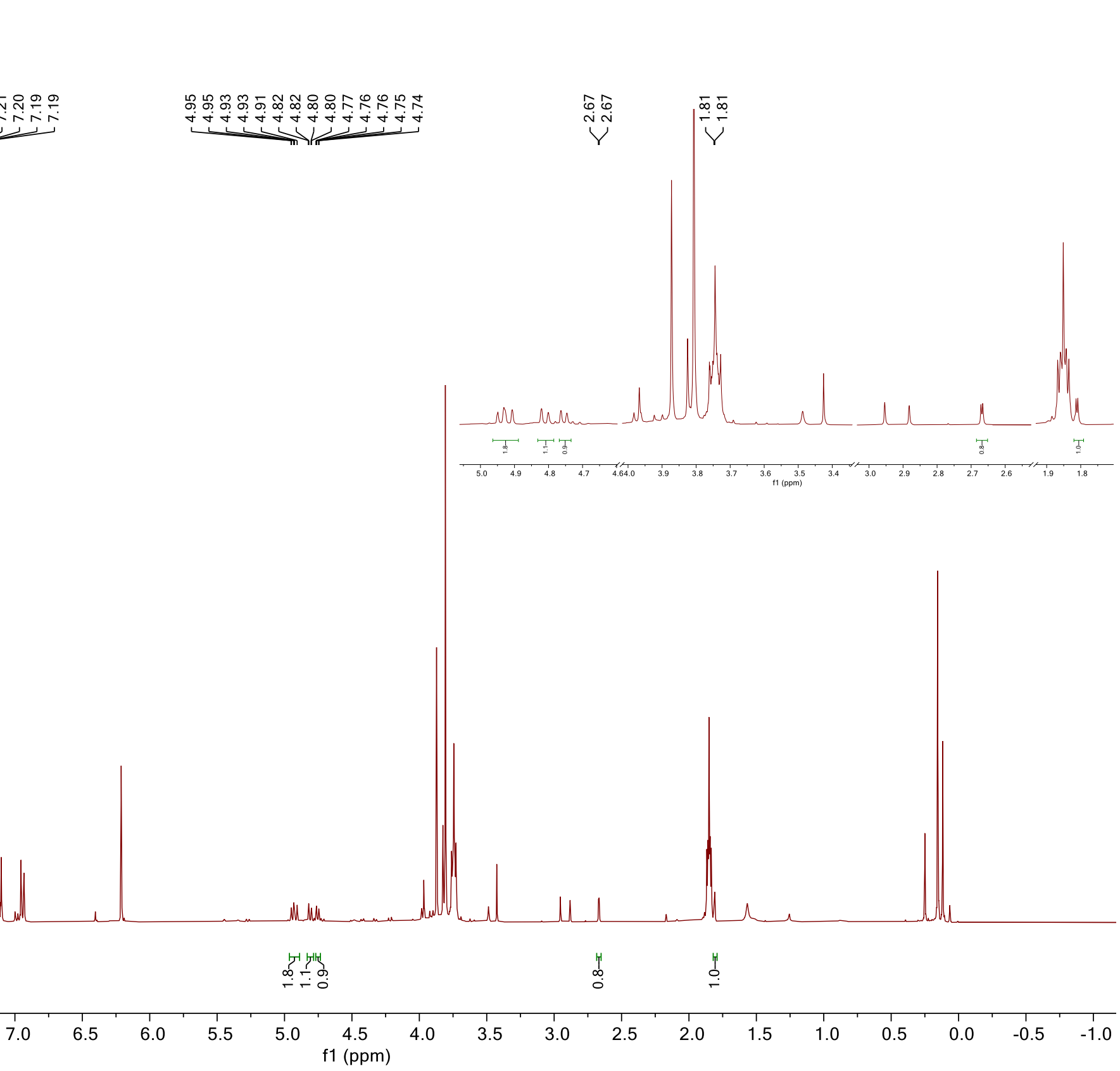

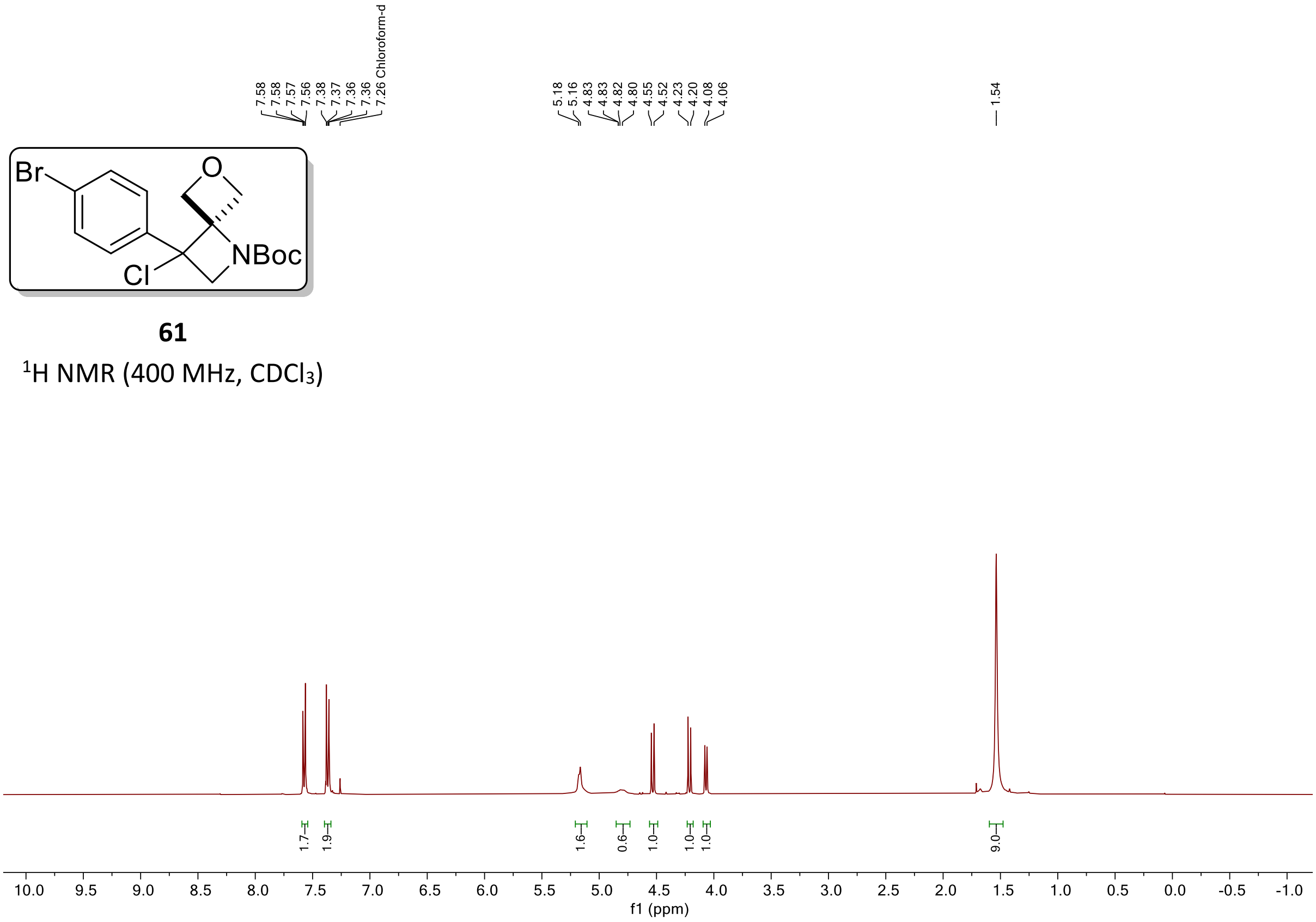


**61**

$^1$H NMR (400 MHz, $CDCl_3$)

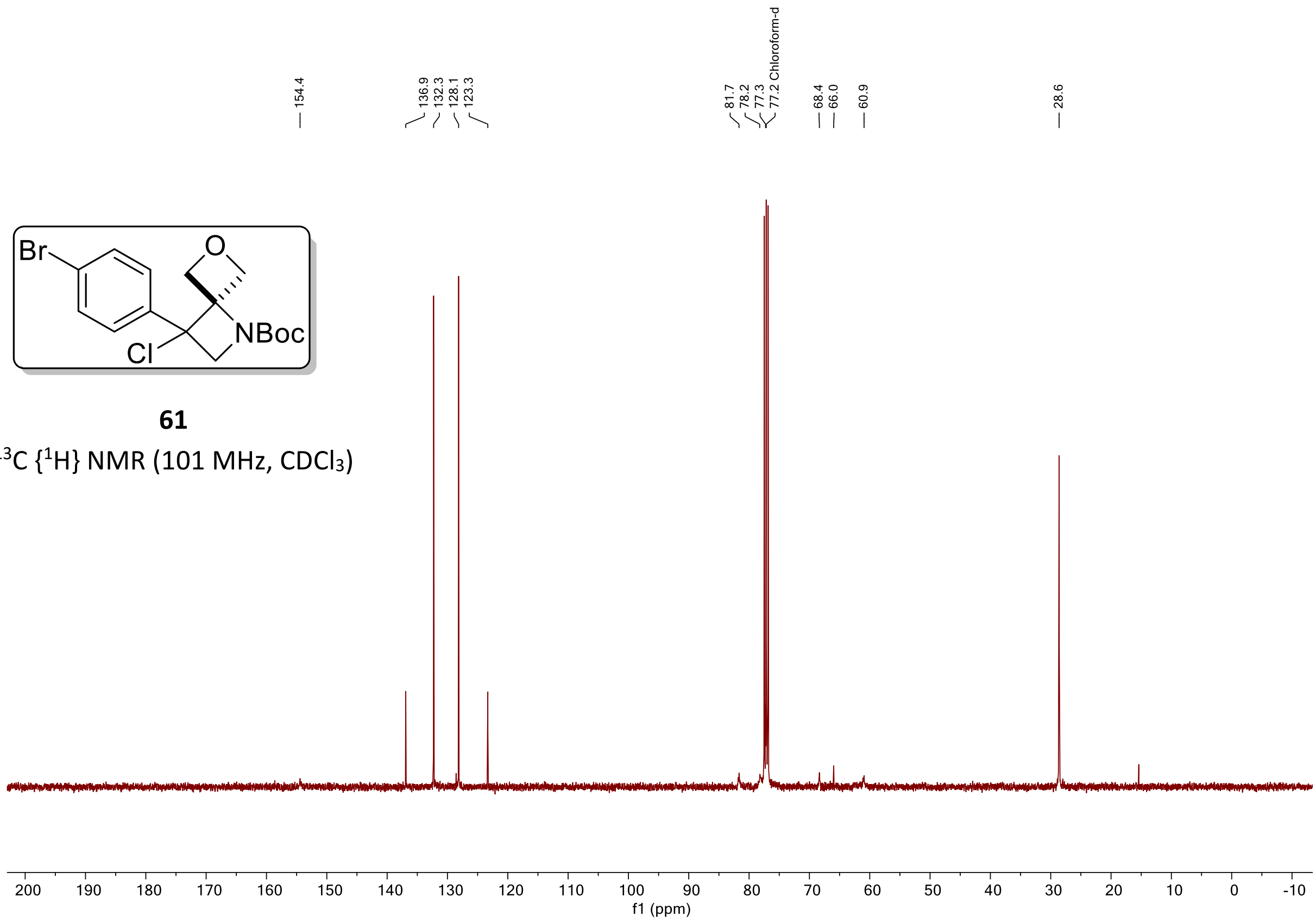


**61**

$^{13}$C {$^1$H} NMR (101 MHz, $CDCl_3$)

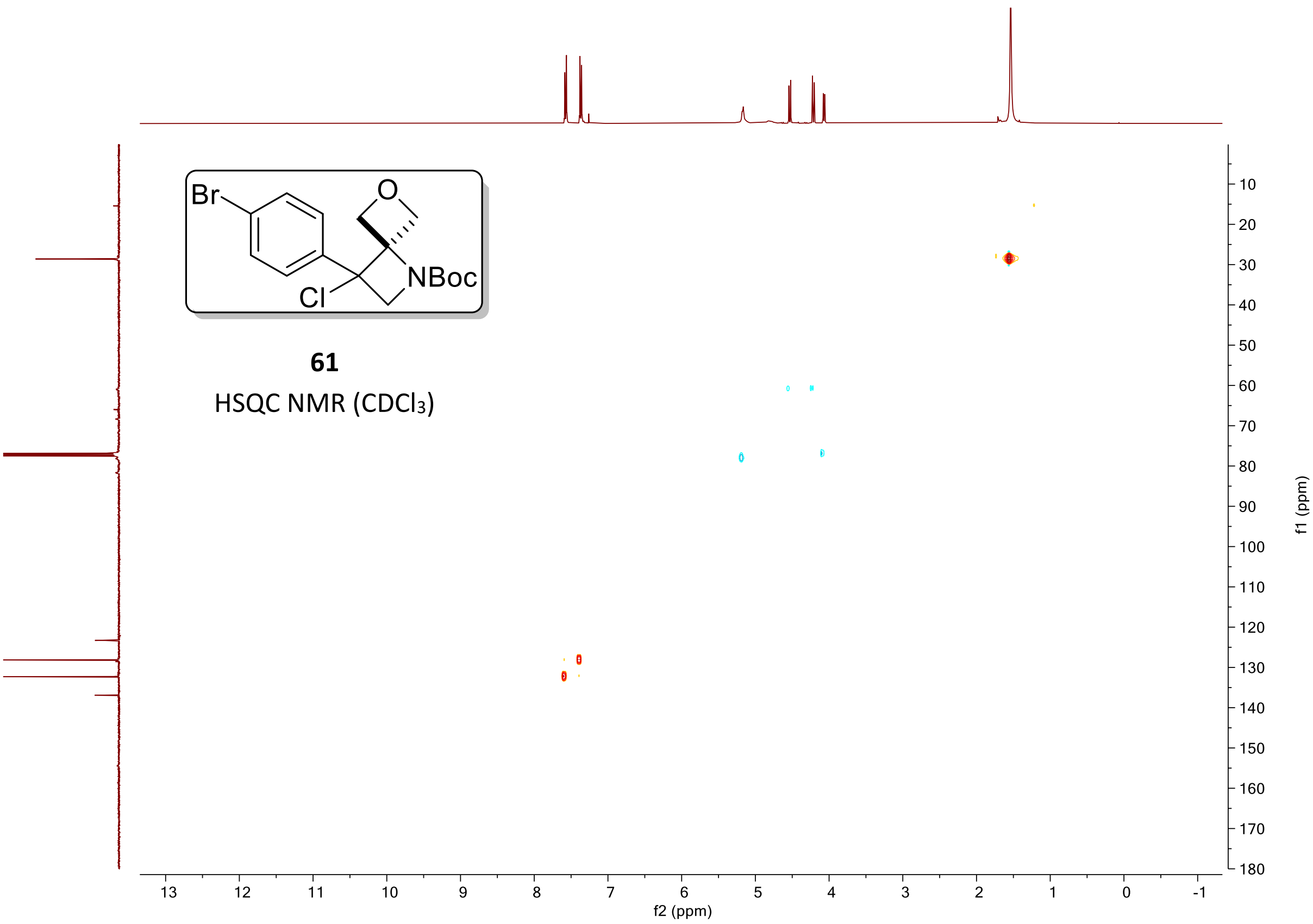

Br
O
NBoc
Cl
61
HSQC NMR (CDCl3)
f2 (ppm)
f1 (ppm)

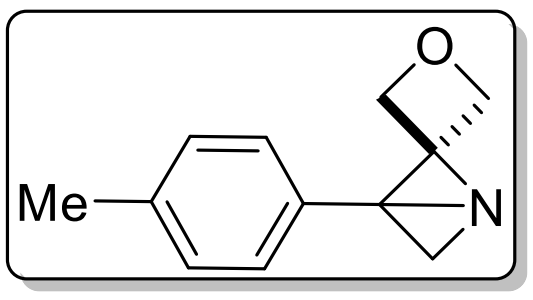


**Crude, 52**

$^{1}H$ NMR (400 MHz, $CDCl_3$)

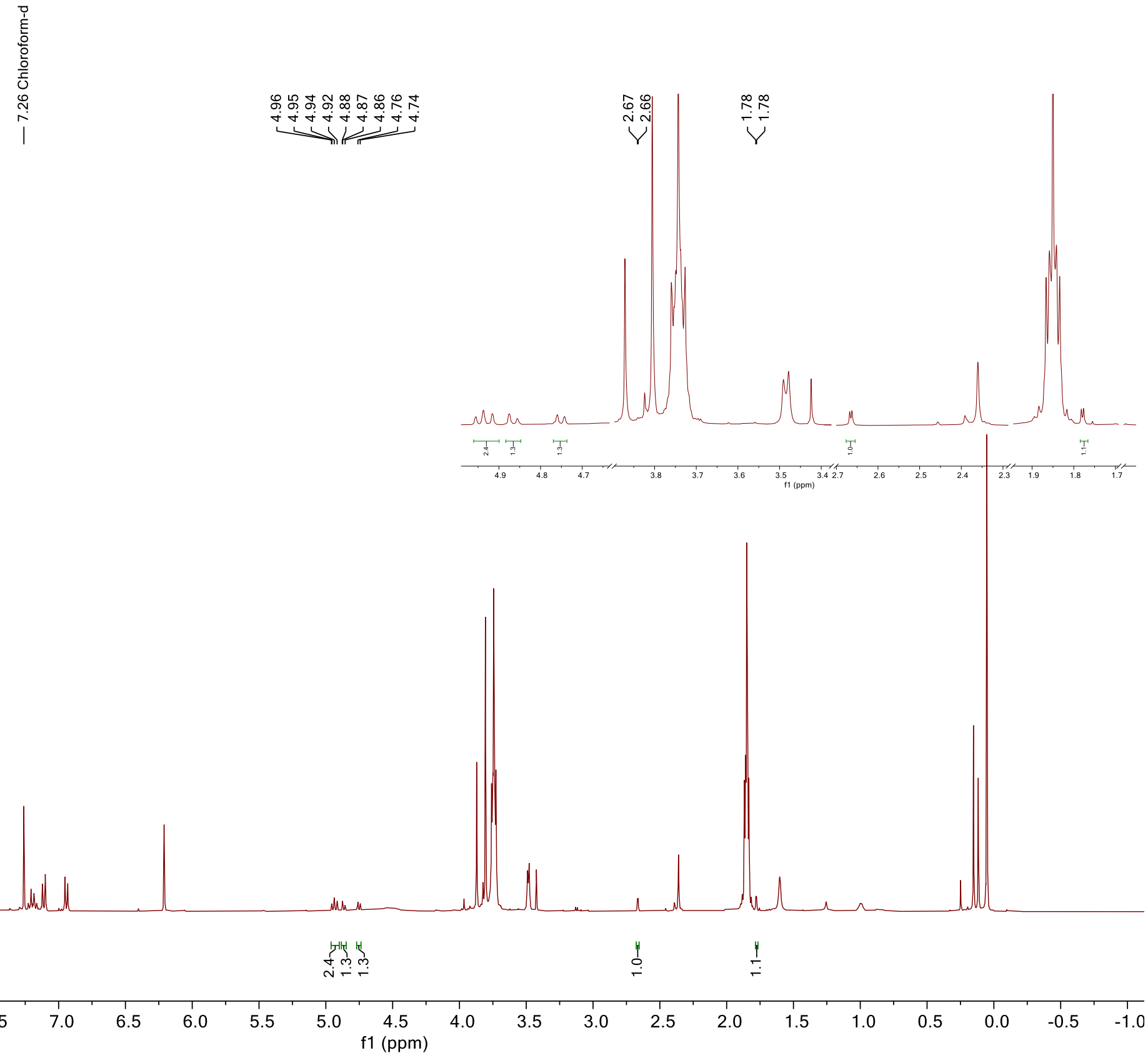

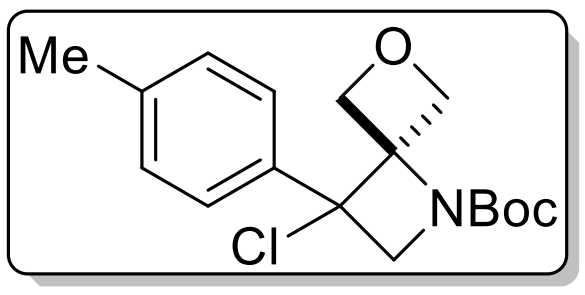


**62**

$^{1}H$ NMR (400 MHz, $CDCl_3$)

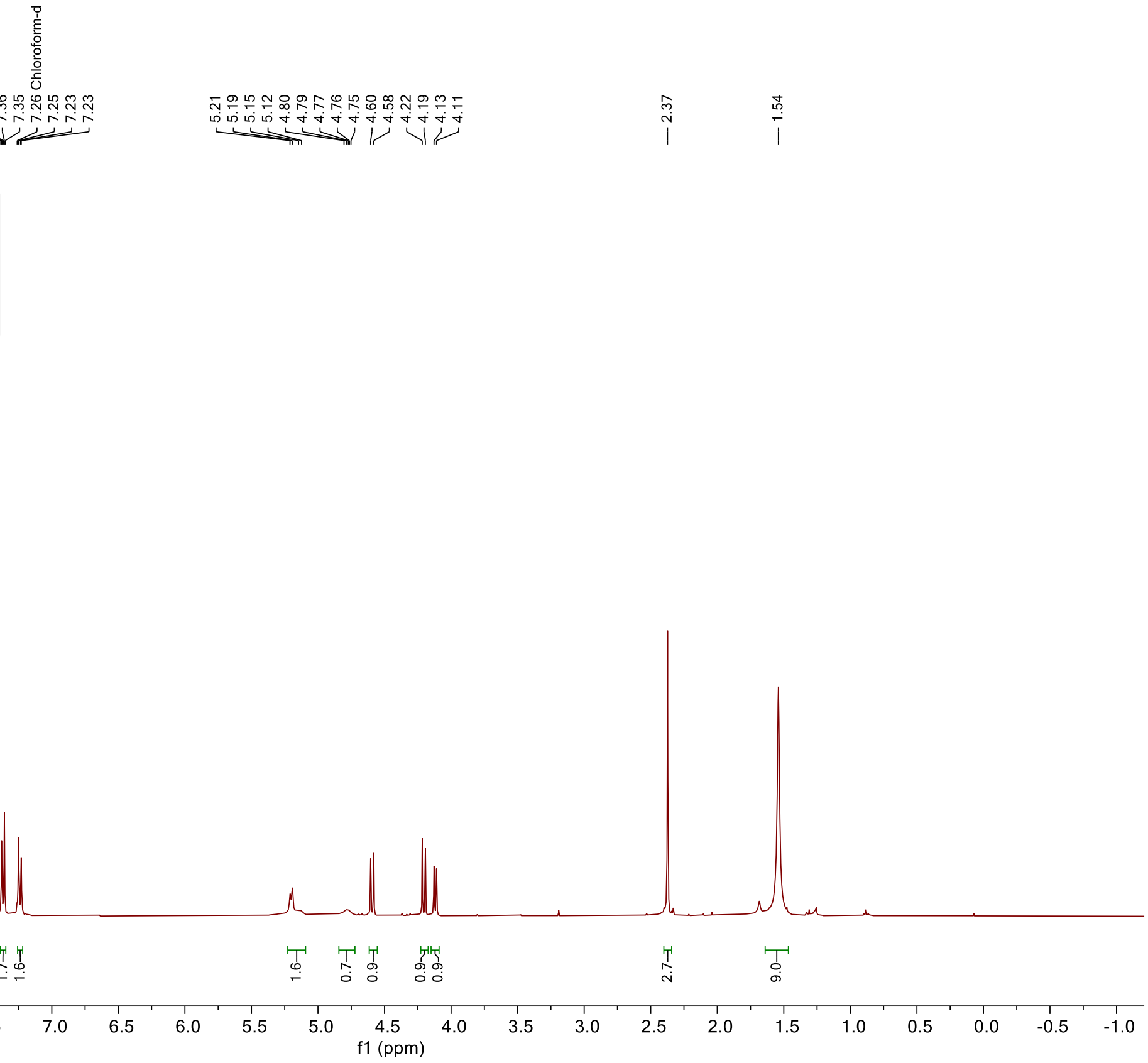


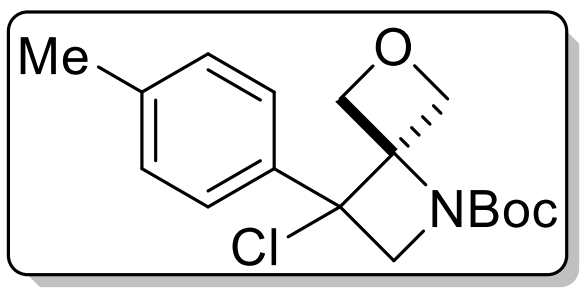


**62**

$^{13}C\ \{^{1}H\}$ NMR (101 MHz, $CDCl_3$)

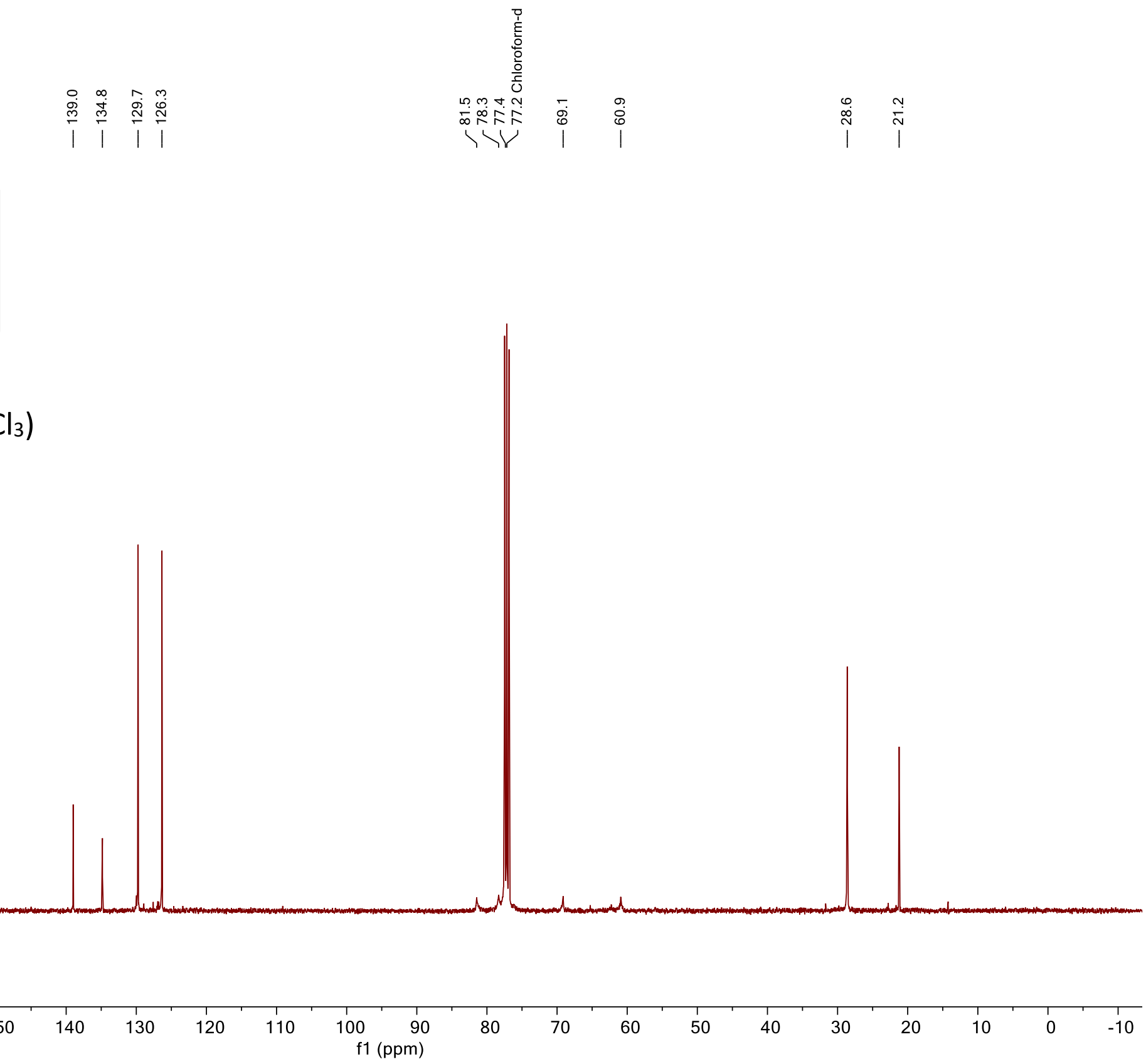

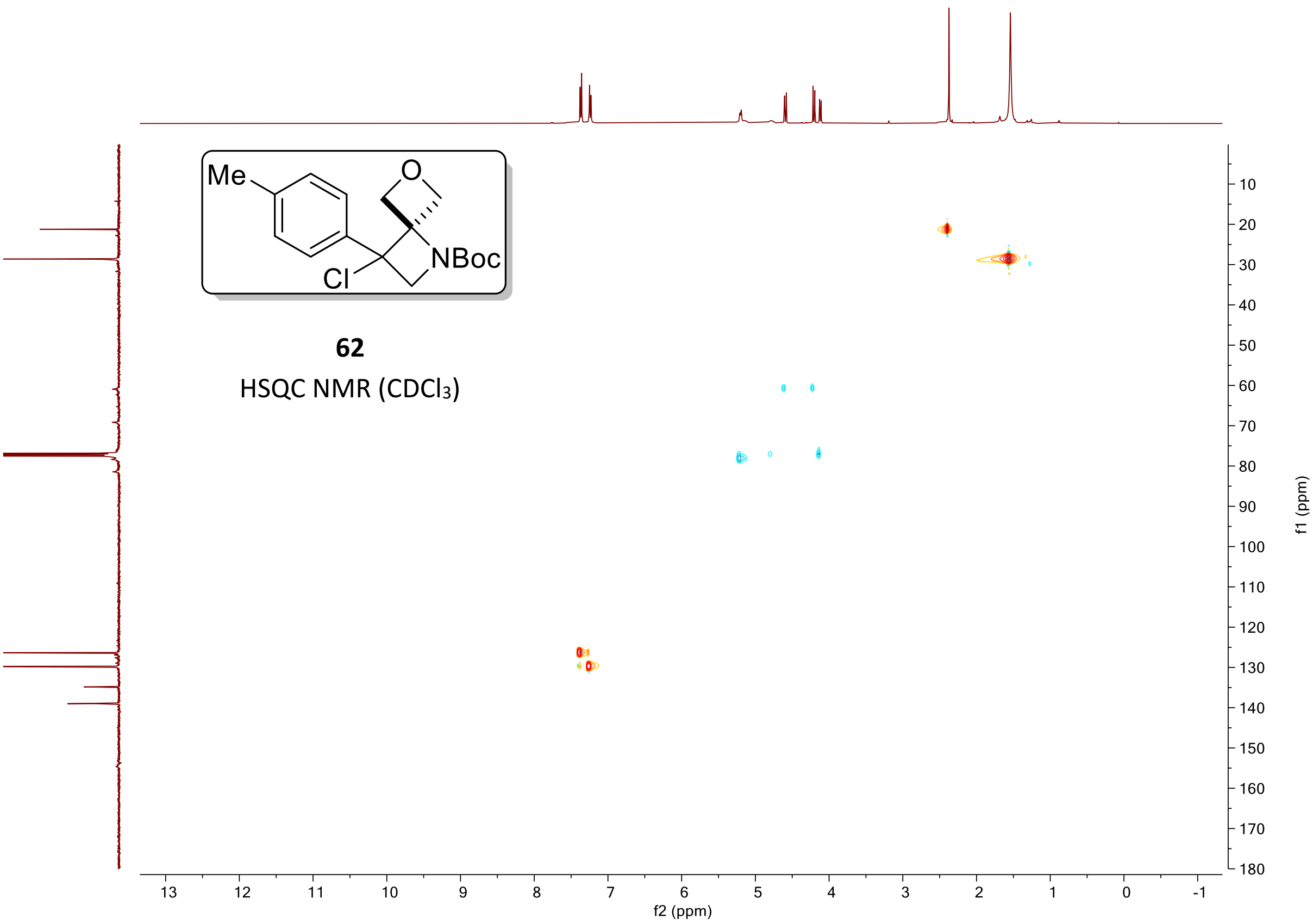

Me
O
NBoc
Cl
62
HSQC NMR (CDCl3)
f2 (ppm)
f1 (ppm)

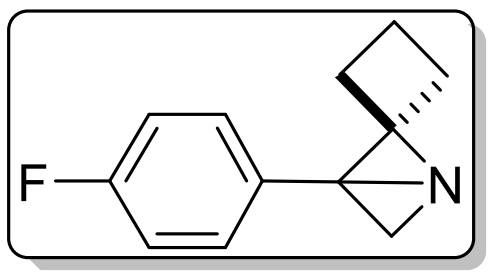

**Crude, 6**

$^{1}$H NMR (400 MHz, $CDCl_3$)

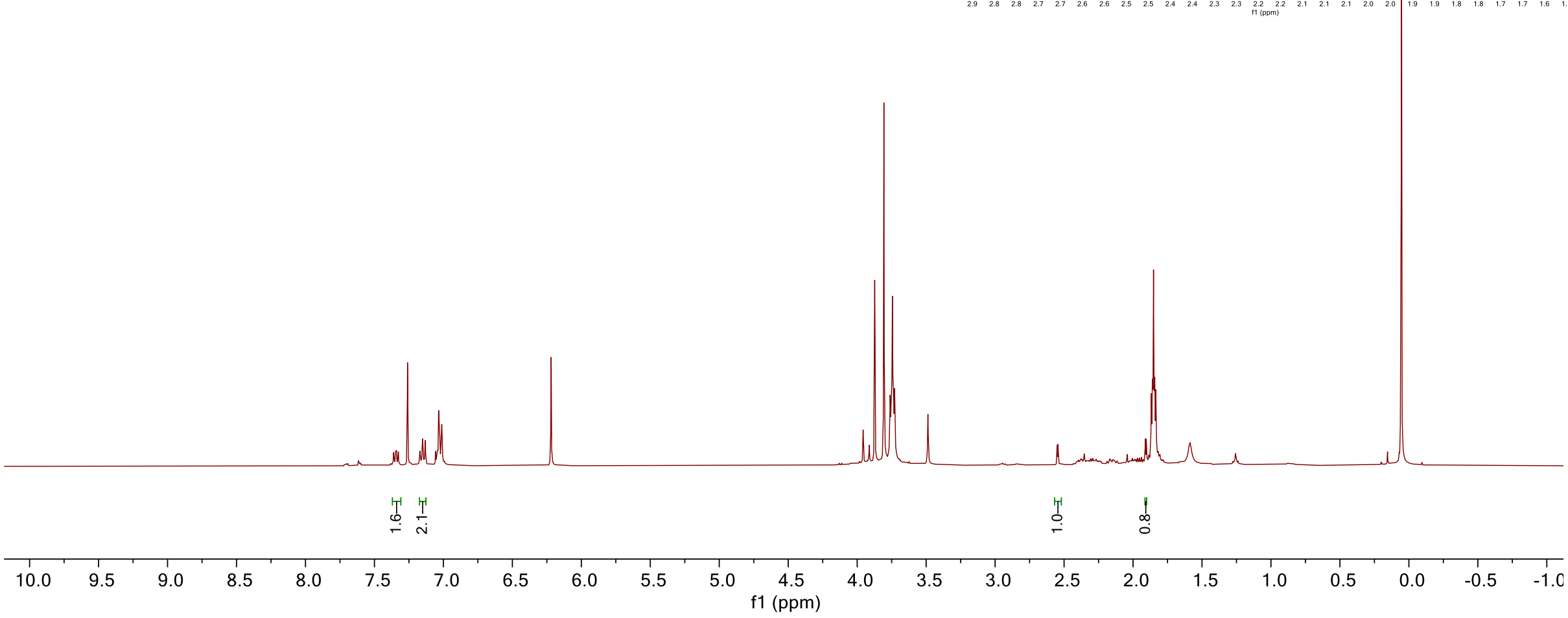

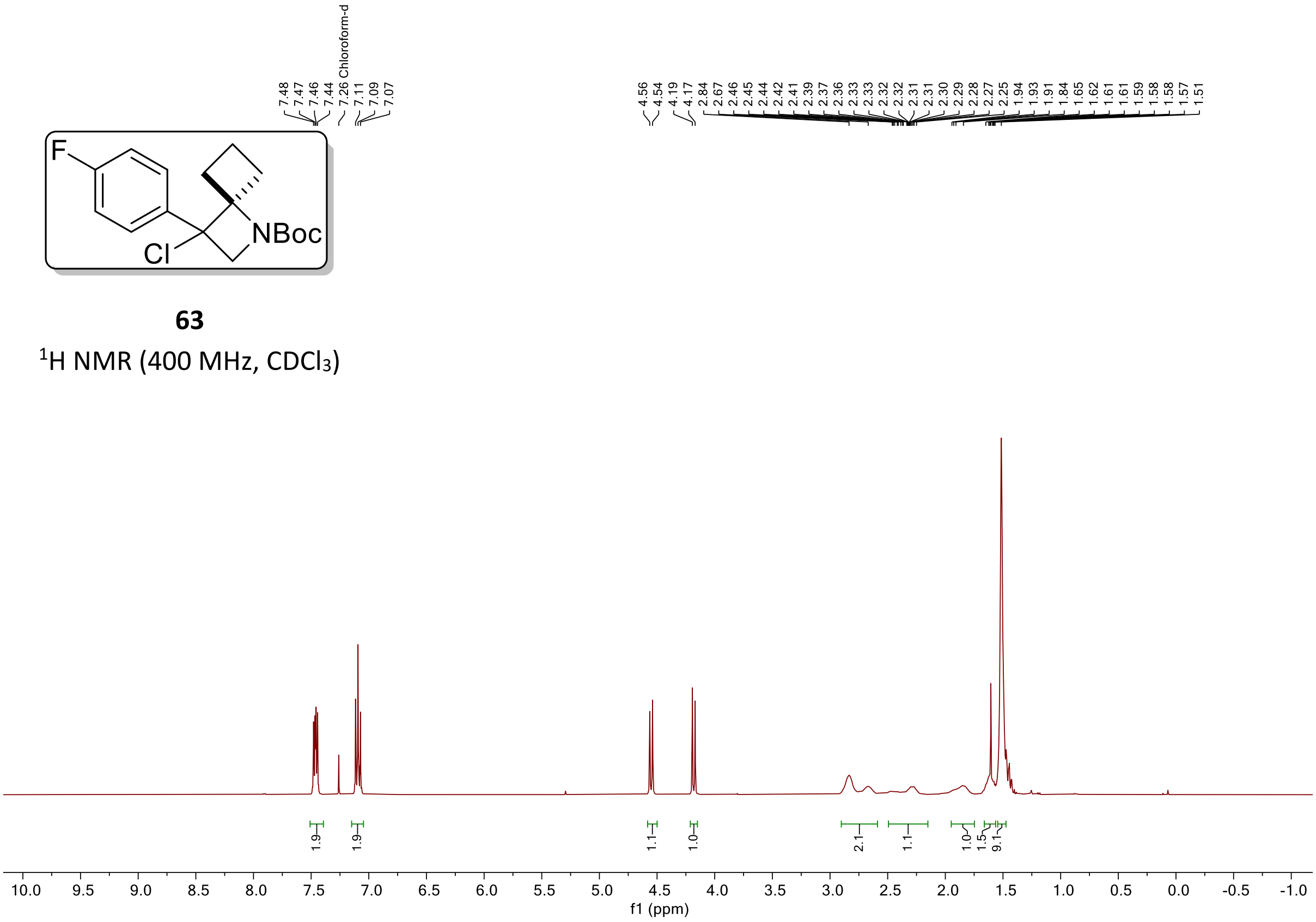

**63**
$^1$H NMR (400 MHz, $CDCl_3$)

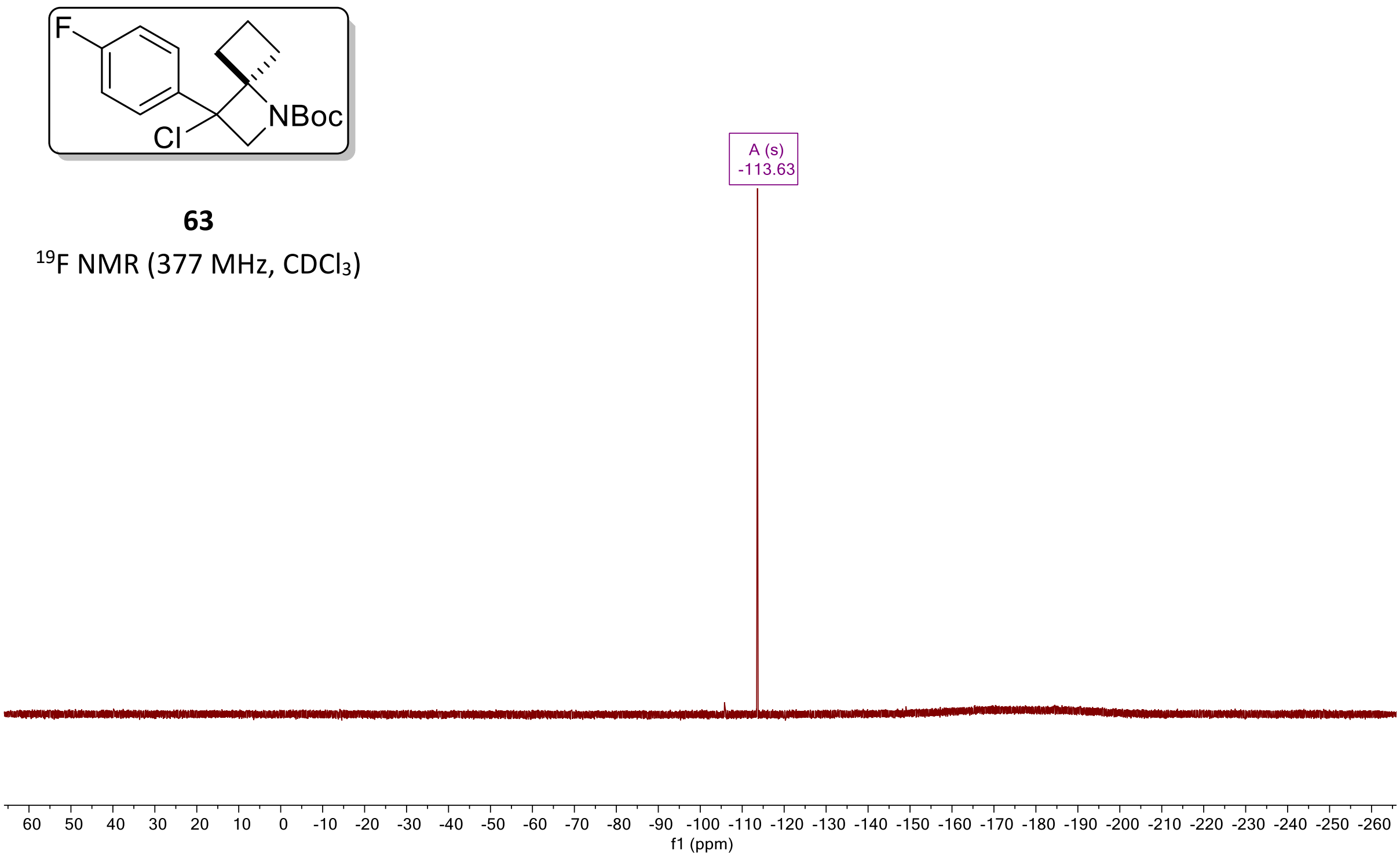

**63**
$^{19}$F NMR (377 MHz, $CDCl_3$)

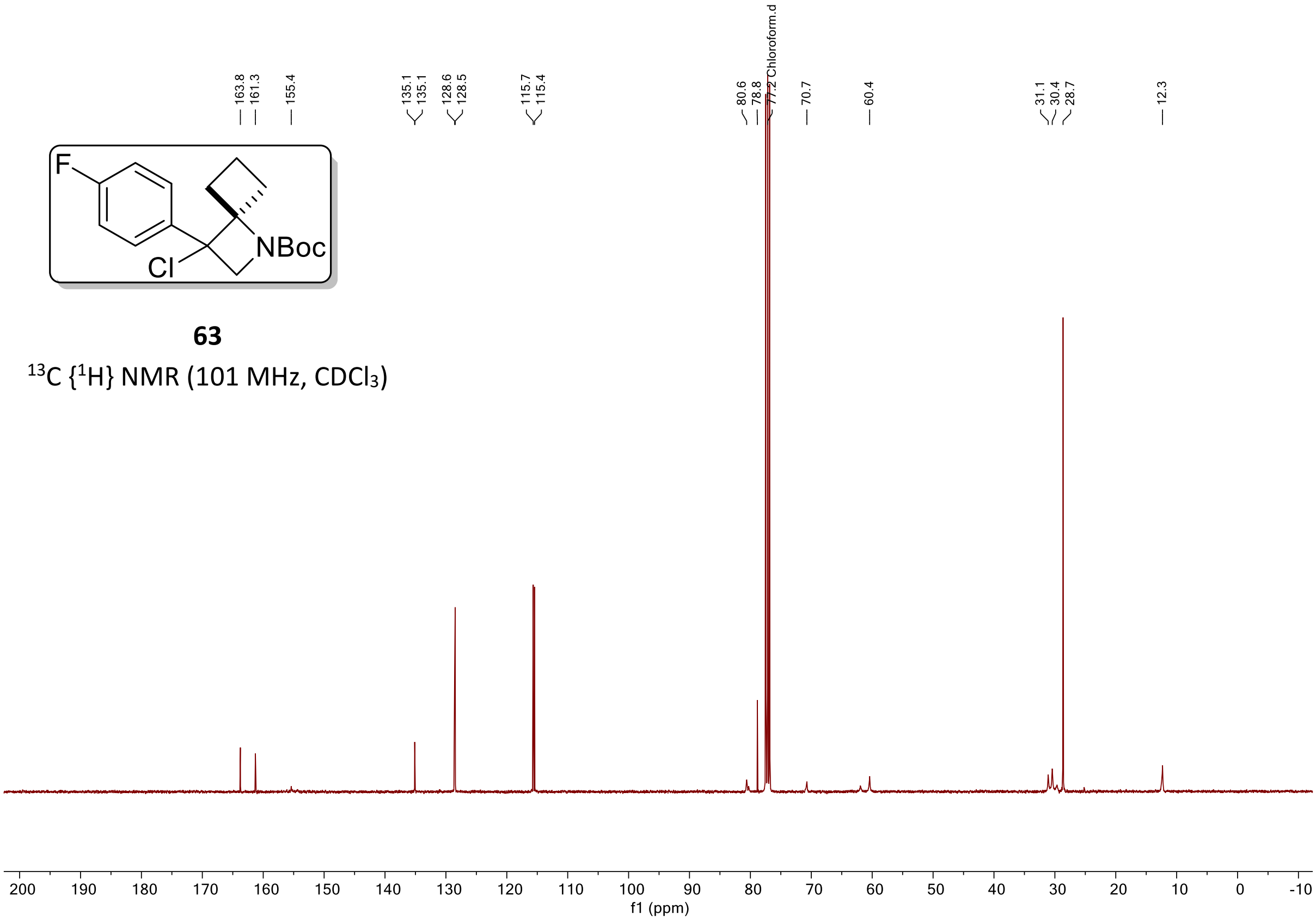

**63**

$^{13}C\ \{^{1}H\}$ NMR (101 MHz, $CDCl_3$)

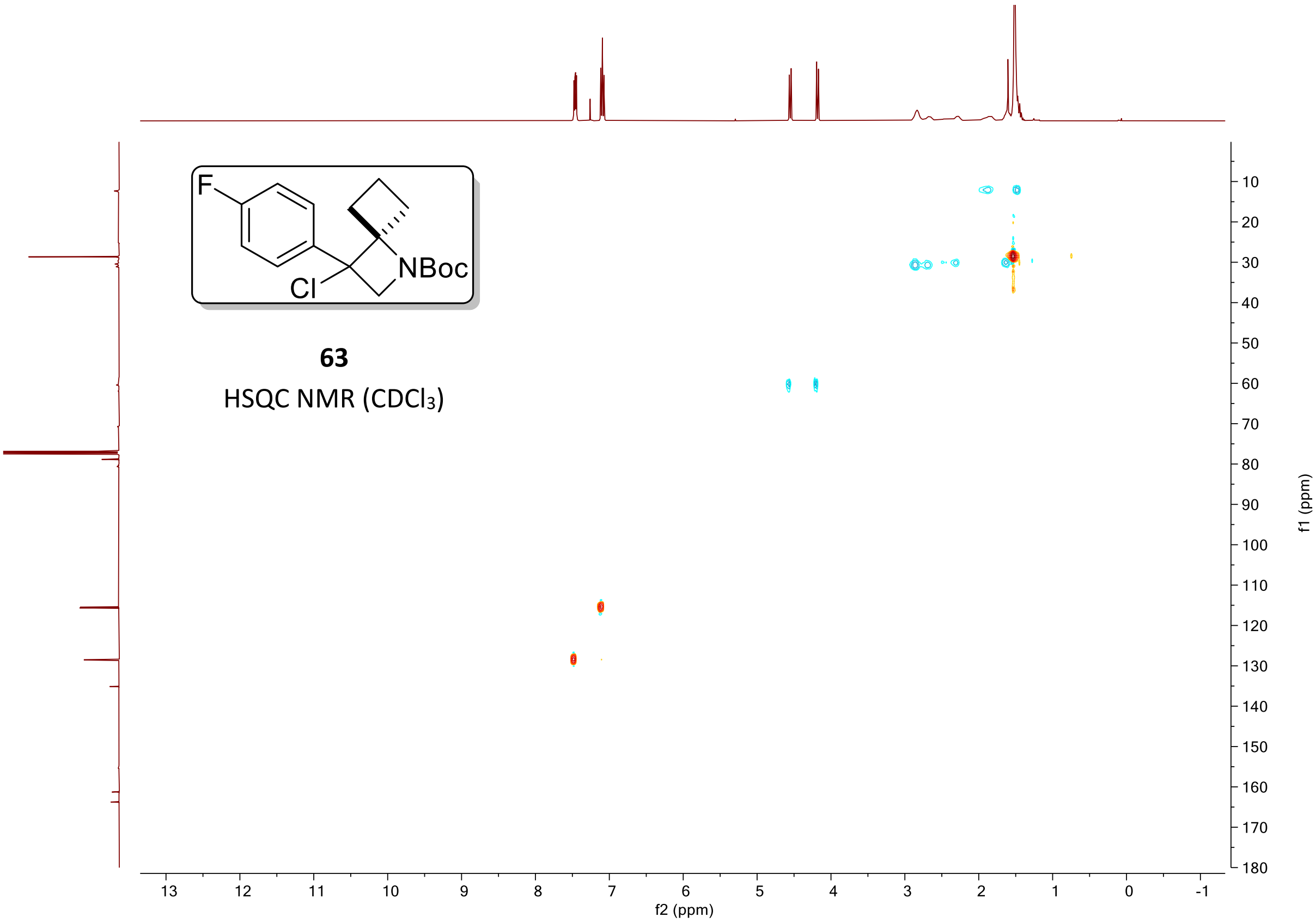

**63**

HSQC NMR ($CDCl_3$)

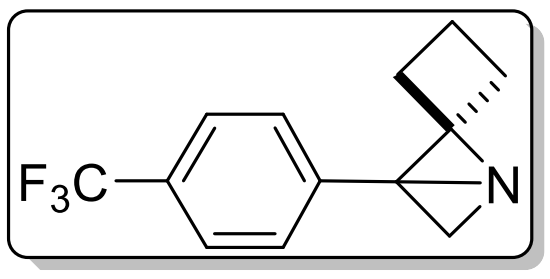


**Crude, 48**

$^{1}H$ NMR (400 MHz, $CDCl_3$)

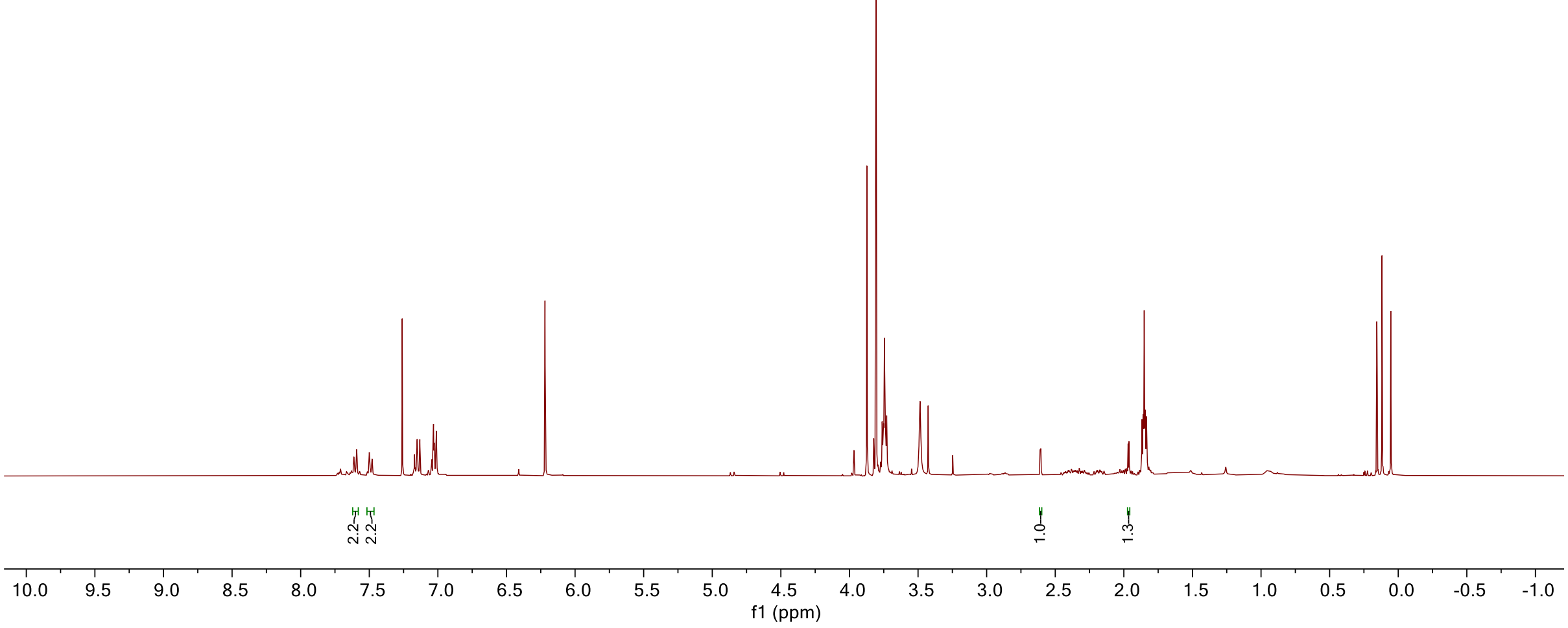

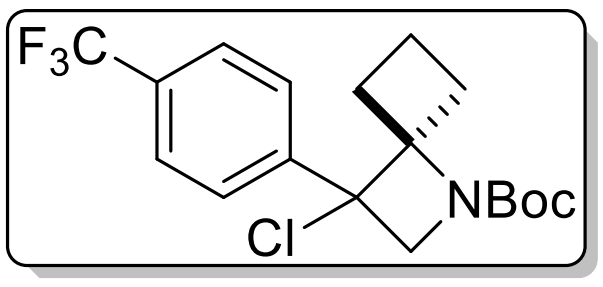


**64**

$^1$H NMR (400 MHz, CDCl$_3$)

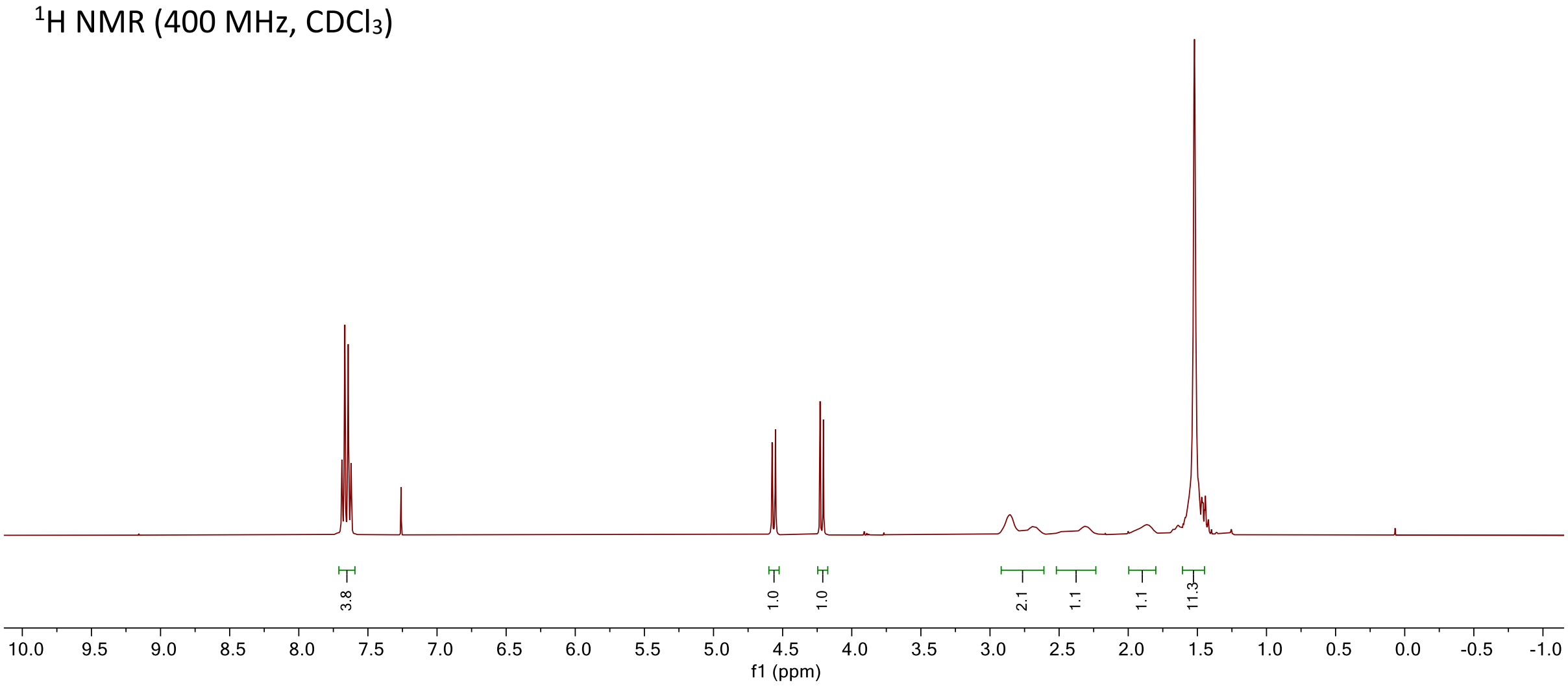


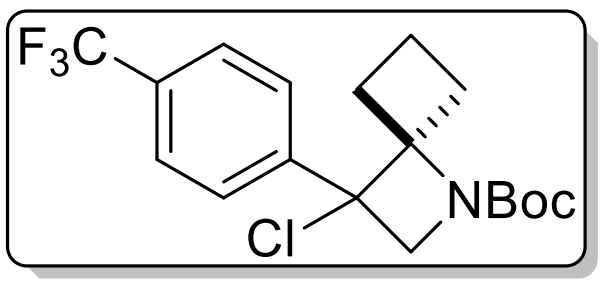


**64**

$^{19}$F NMR (377 MHz, CDCl$_3$)

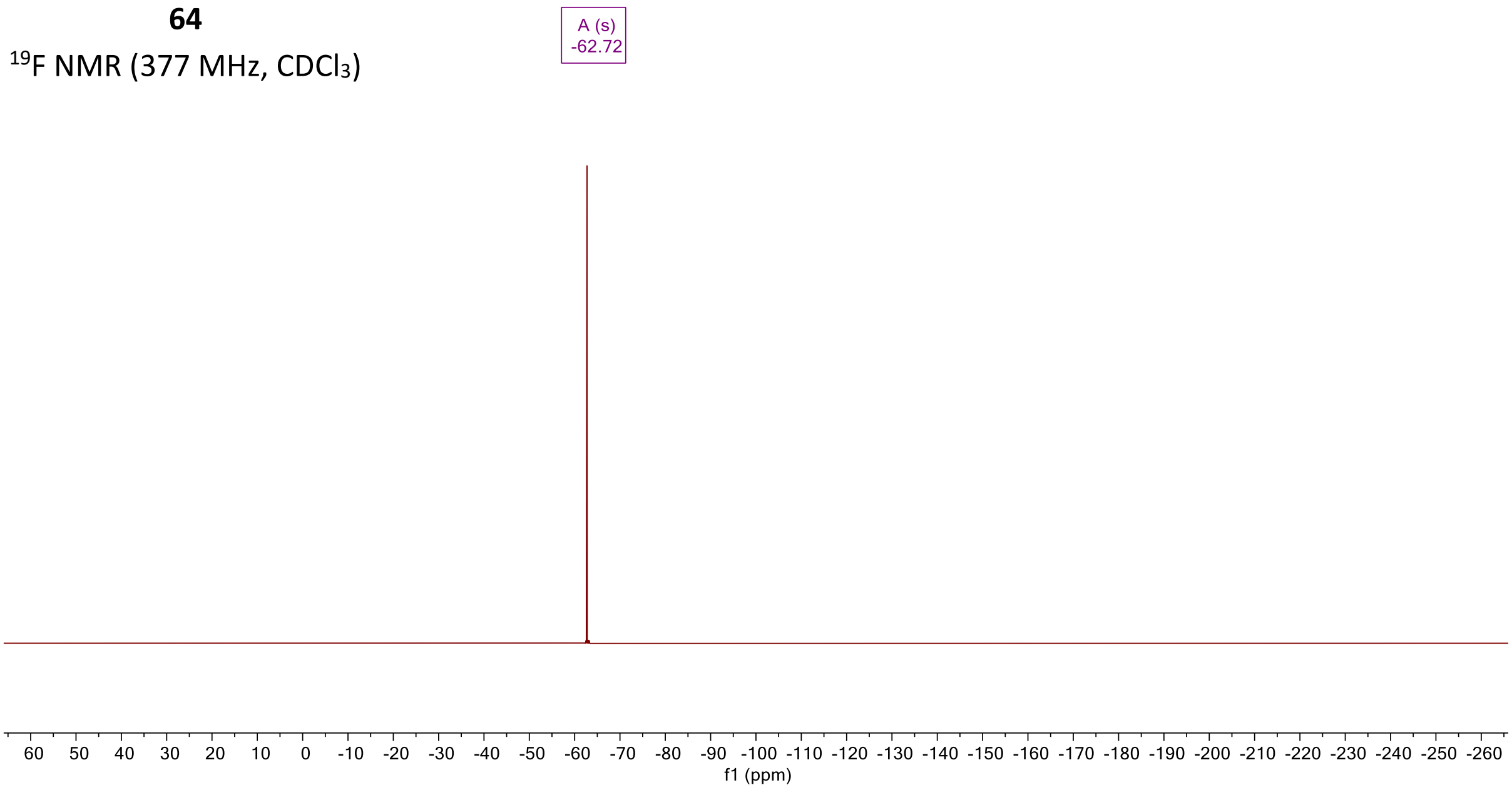

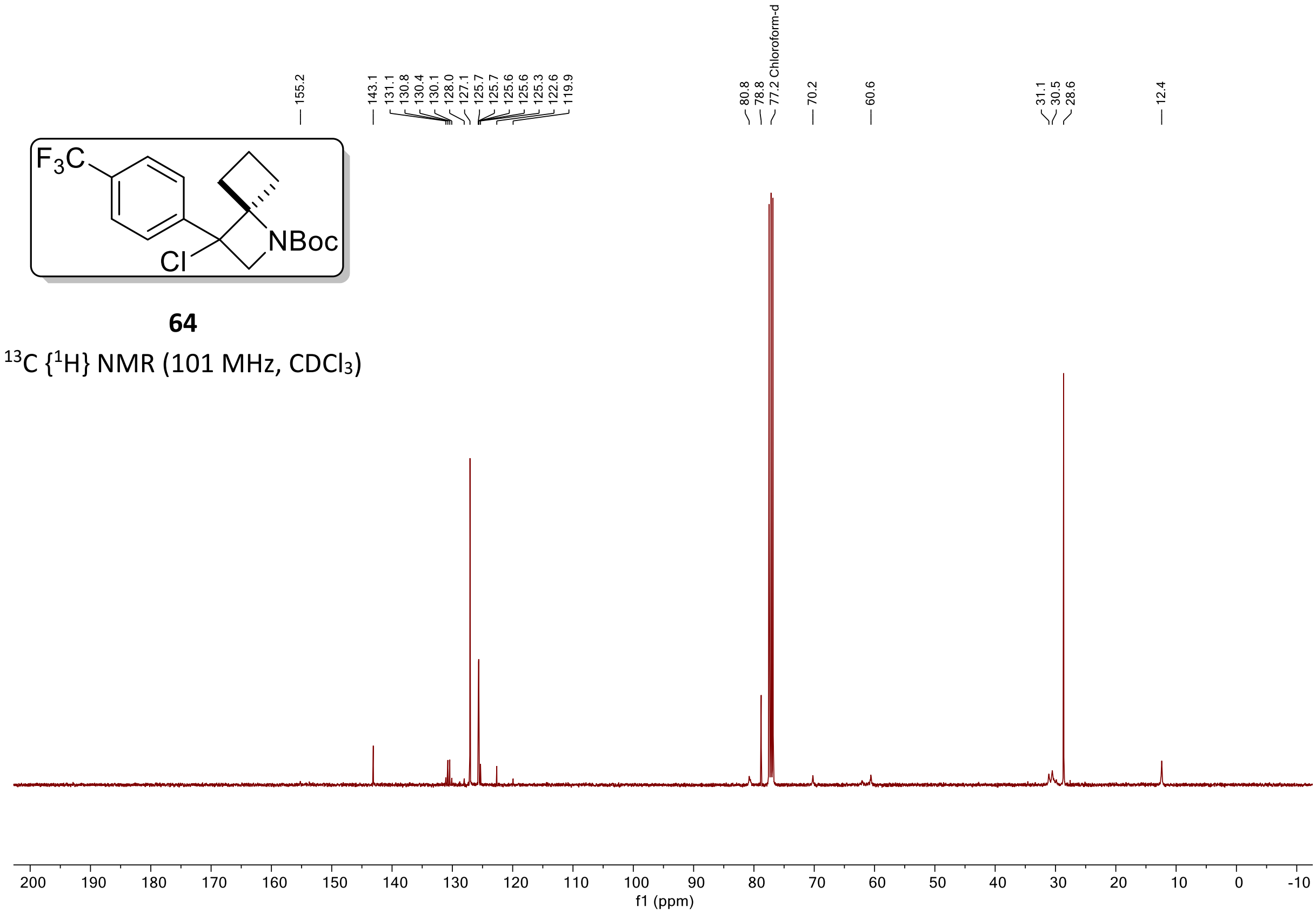

155.2
143.1
131.1
130.8
130.4
130.1
128.0
127.1
125.7
125.7
125.6
125.6
125.3
122.6
119.9
80.8
78.8
77.2 Chloroform-d
70.2
60.6
31.1
30.5
28.6
12.4
$F_3C$
Cl
NBoc
64
$^{13}C\{^1H\}$ NMR (101 MHz, $CDCl_3$)
f1 (ppm)


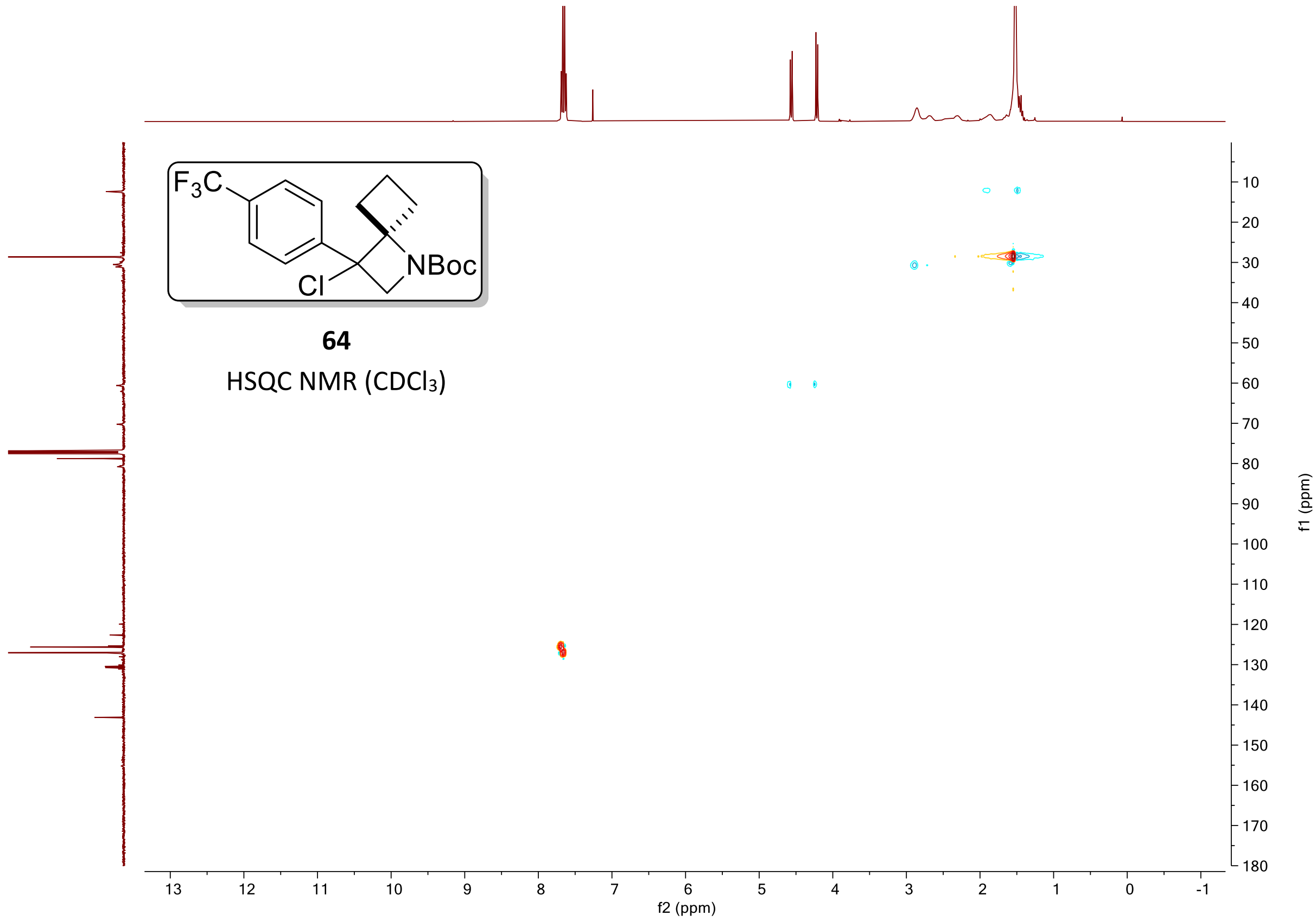

$F_3C$
Cl
NBoc
64
HSQC NMR ($CDCl_3$)
f2 (ppm)
f1 (ppm)

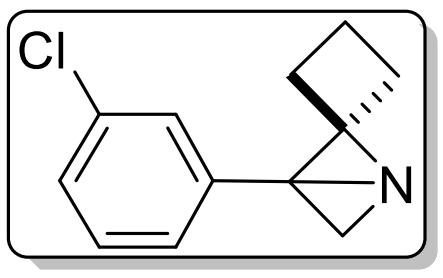

**50**

$^1H$ NMR (400 MHz, $CDCl_3$)

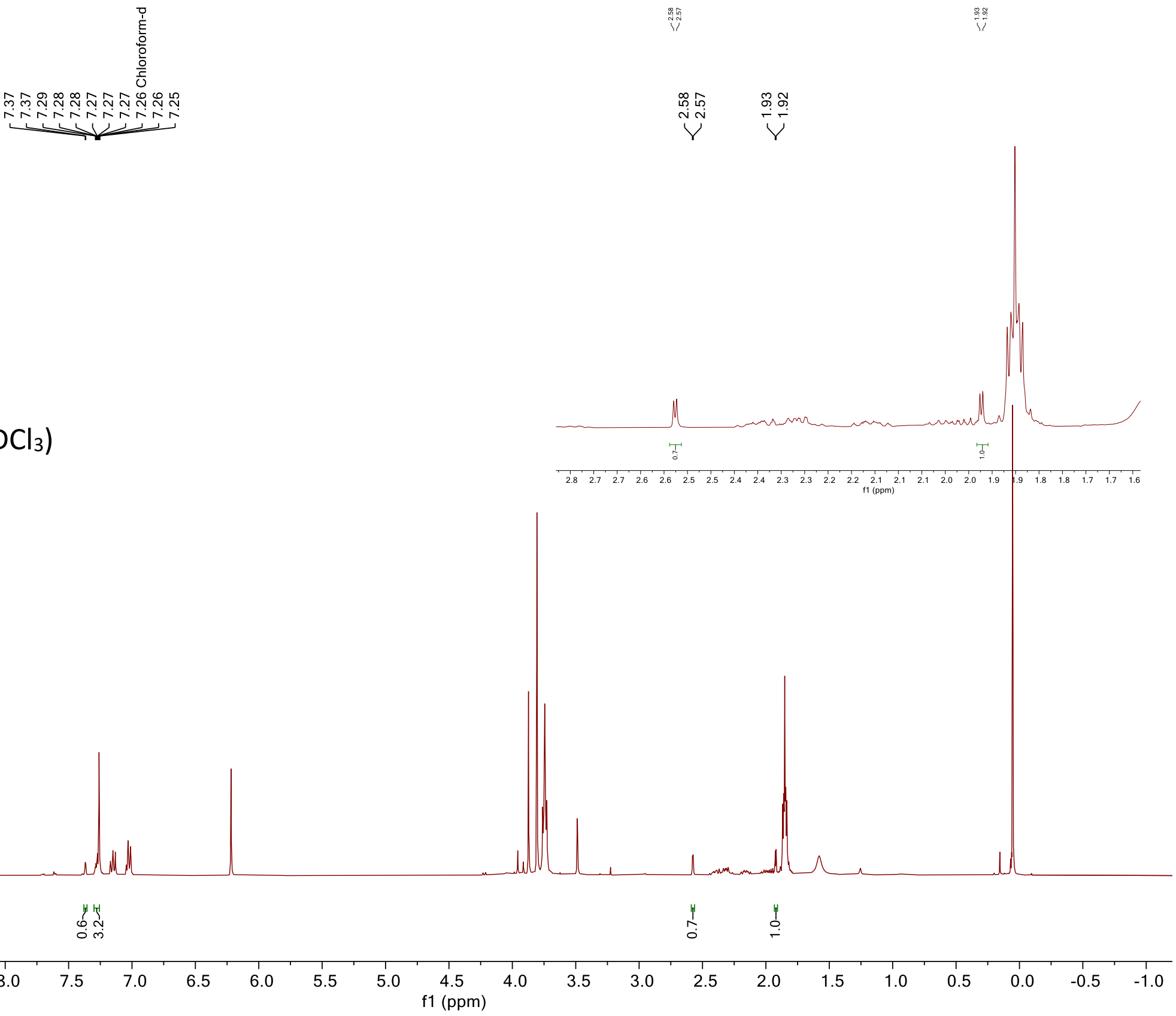

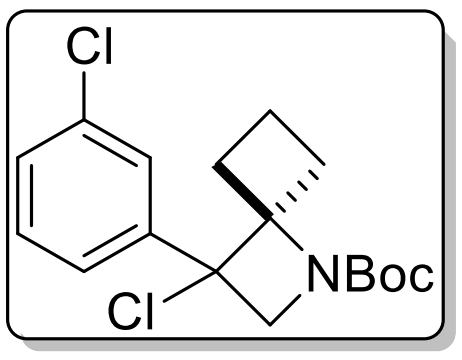


**65**

$^1$H NMR (400 MHz, $CDCl_3$)

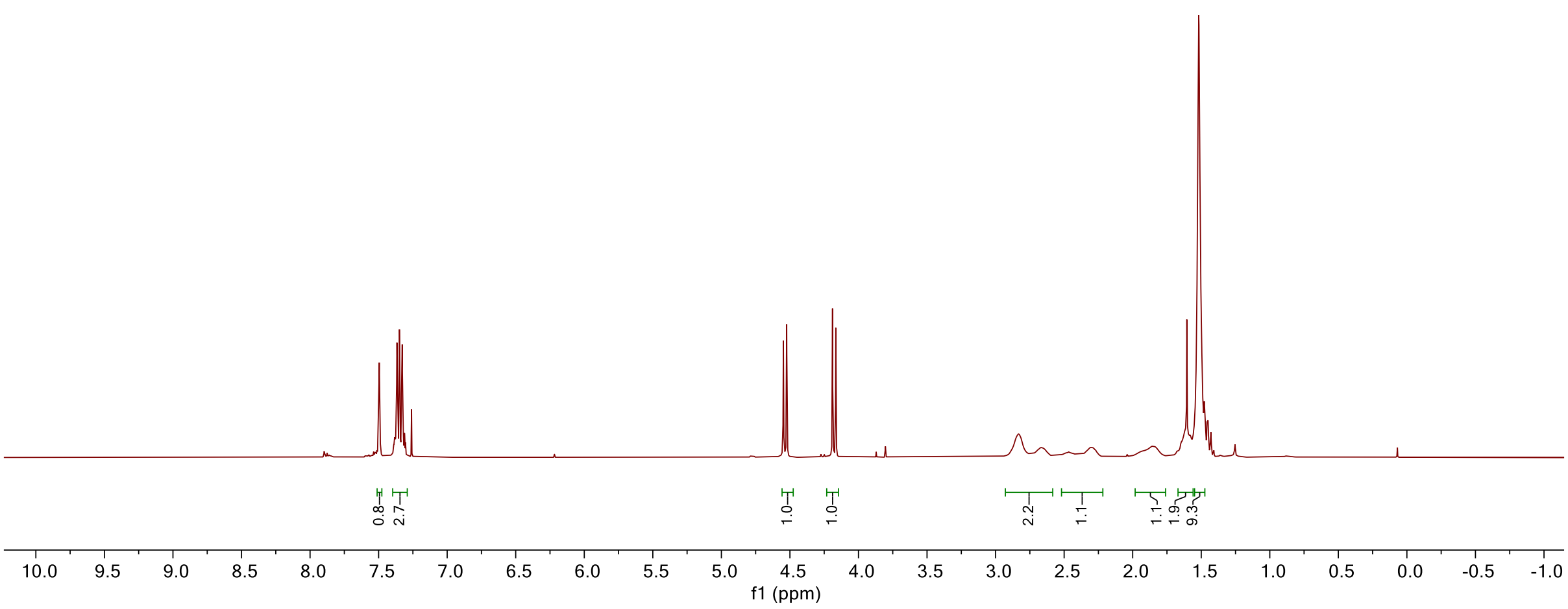


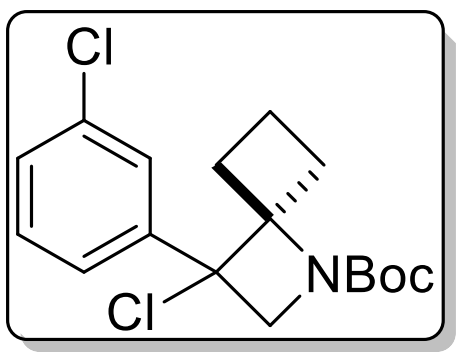


**65**

$^{13}$C {$^1$H} NMR (101 MHz, $CDCl_3$)

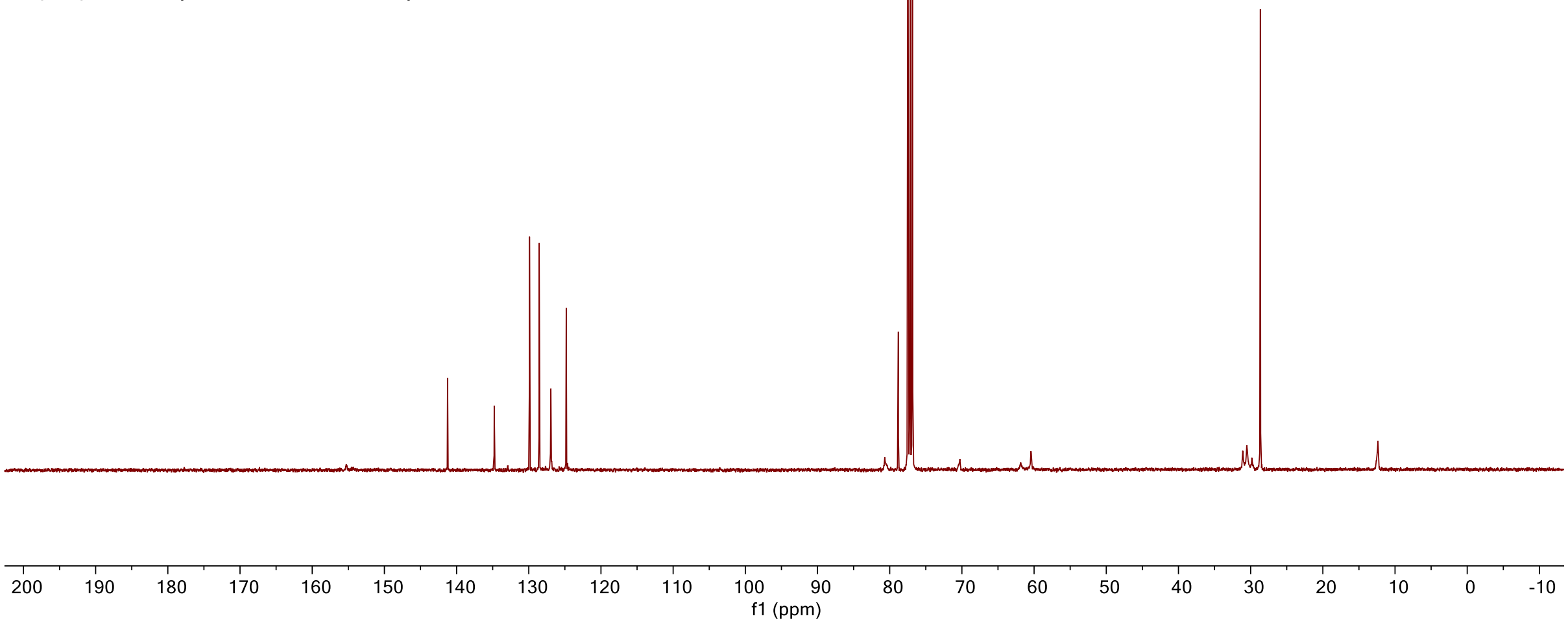

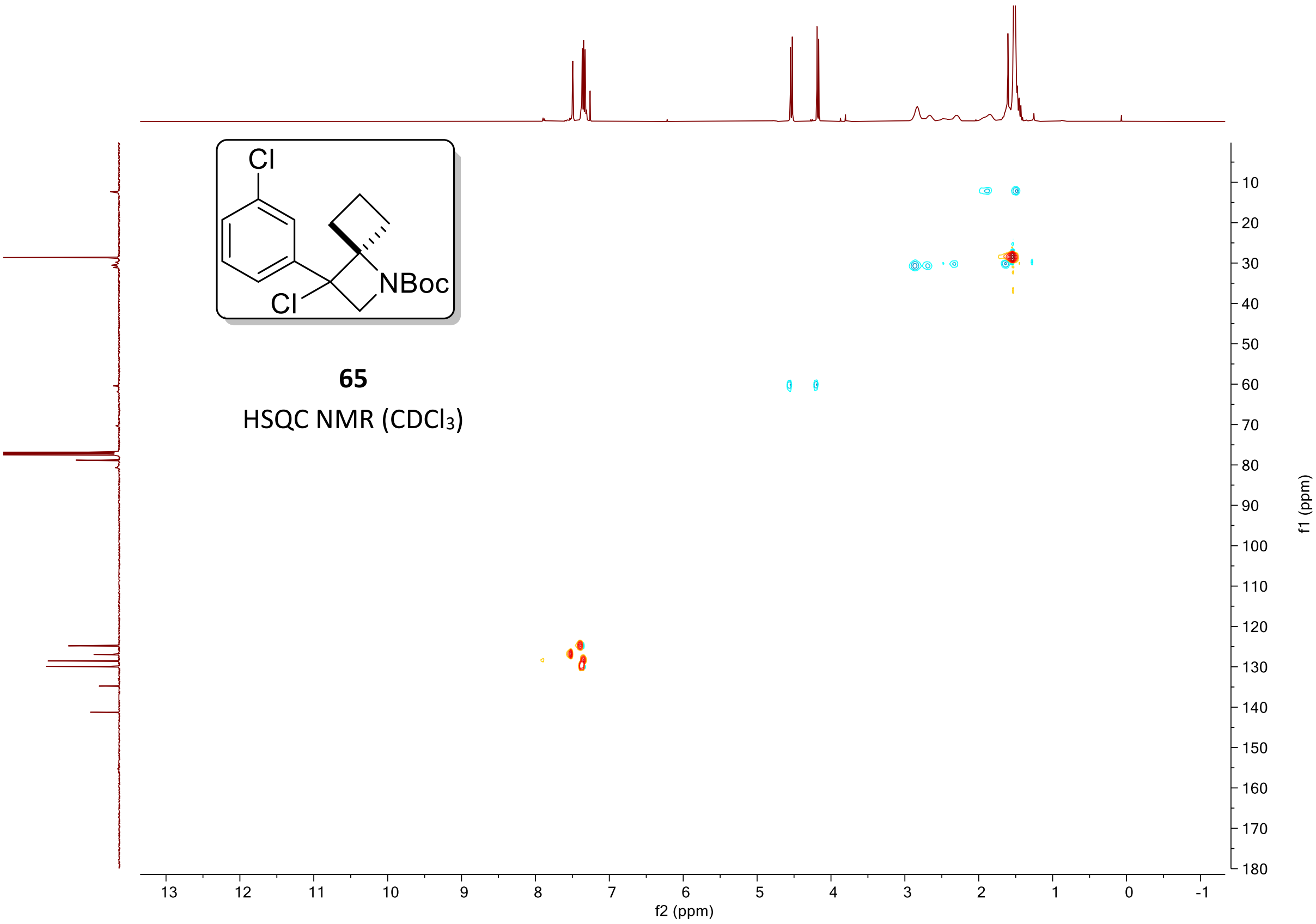


**65**
HSQC NMR ($CDCl_3$)

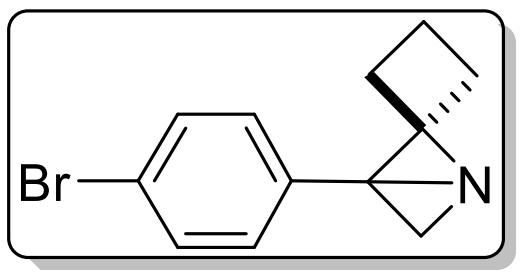

**Crude,51**

$^{1}$H NMR (400 MHz, $CDCl_3$)

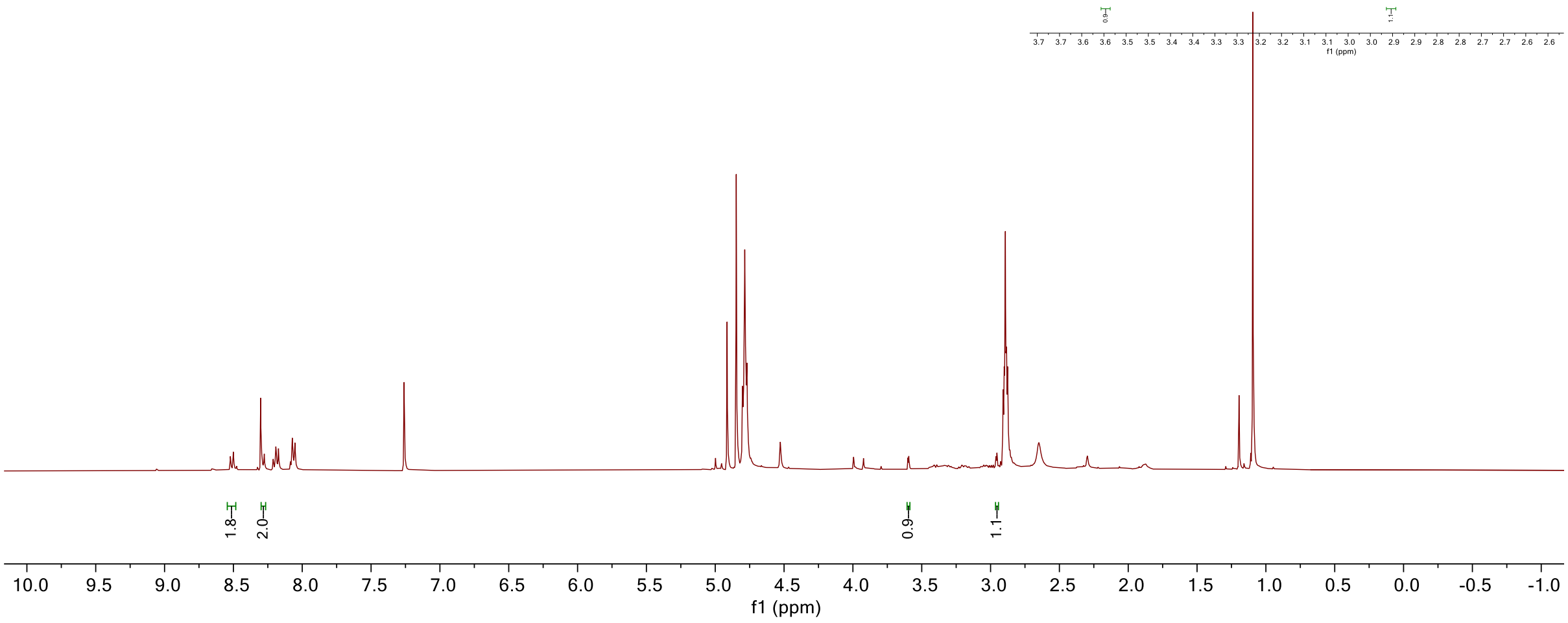

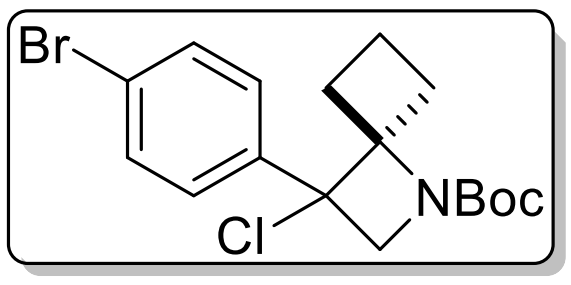

**66**

$^{1}$H NMR (400 MHz, $CDCl_3$)

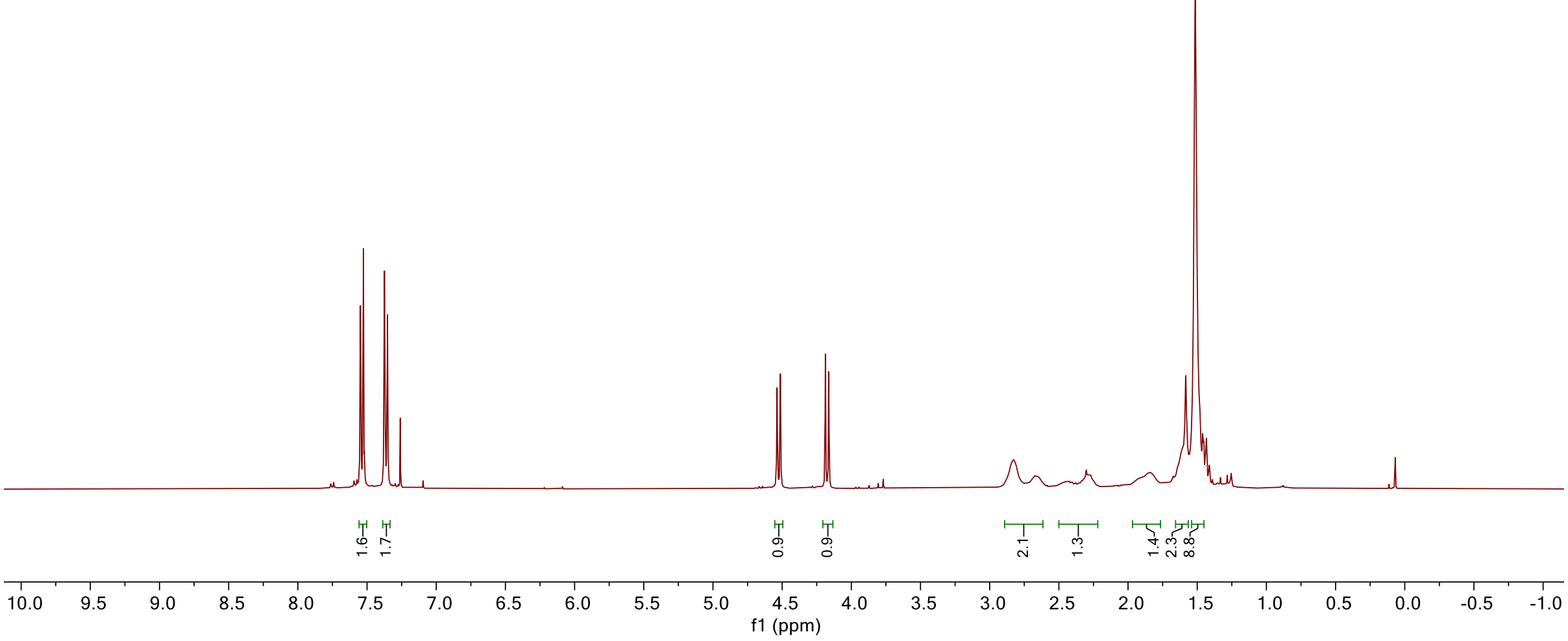


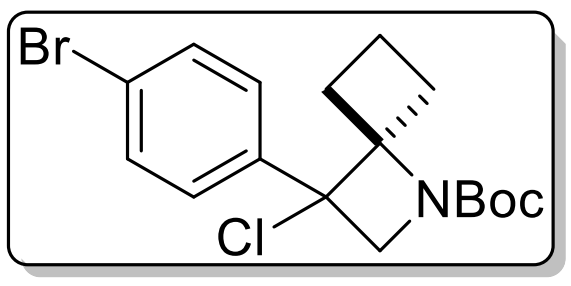

**66**

$^{13}$C {$^{1}$H} NMR (101 MHz, $CDCl_3$)

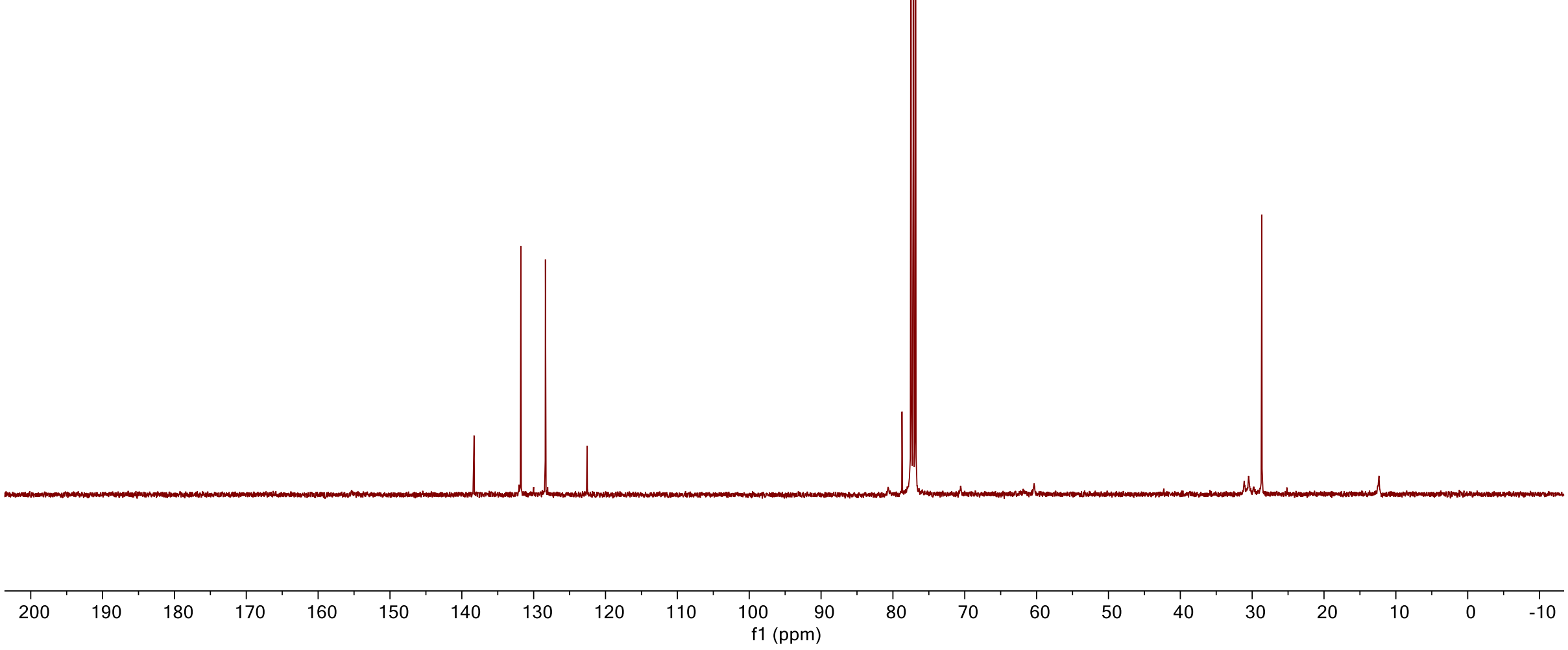

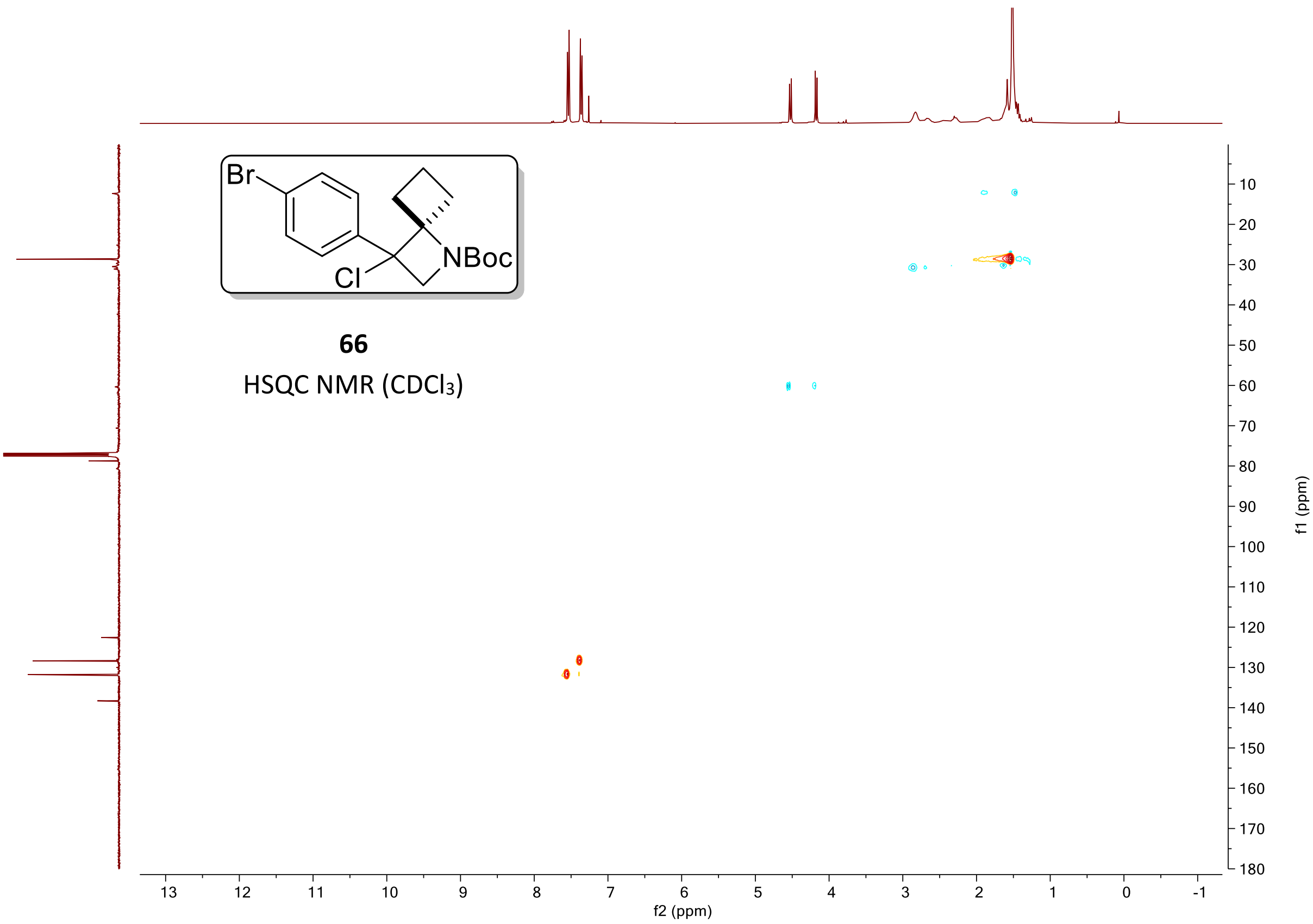


**66**

HSQC NMR ($CDCl_3$)

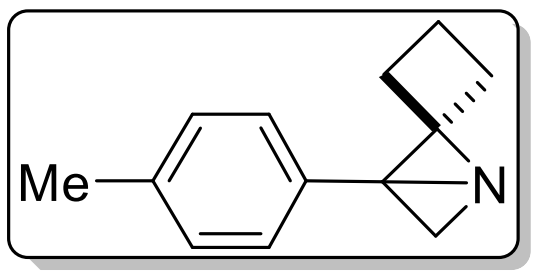


**Crude, 52**

$^{1}$H NMR (400 MHz, $CDCl_3$)

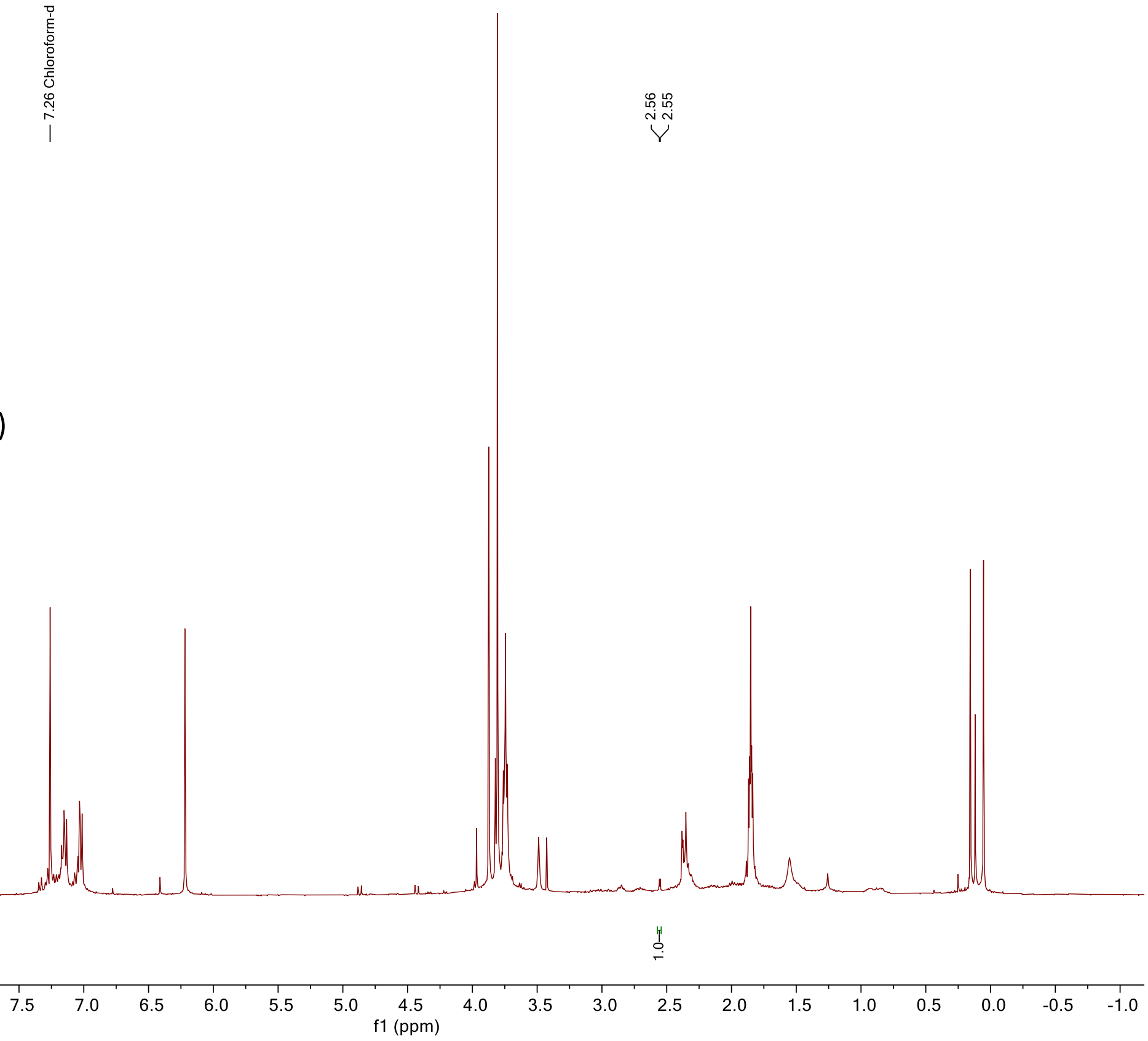

**67**

$^{1}H$ NMR (400 MHz, $CDCl_3$)

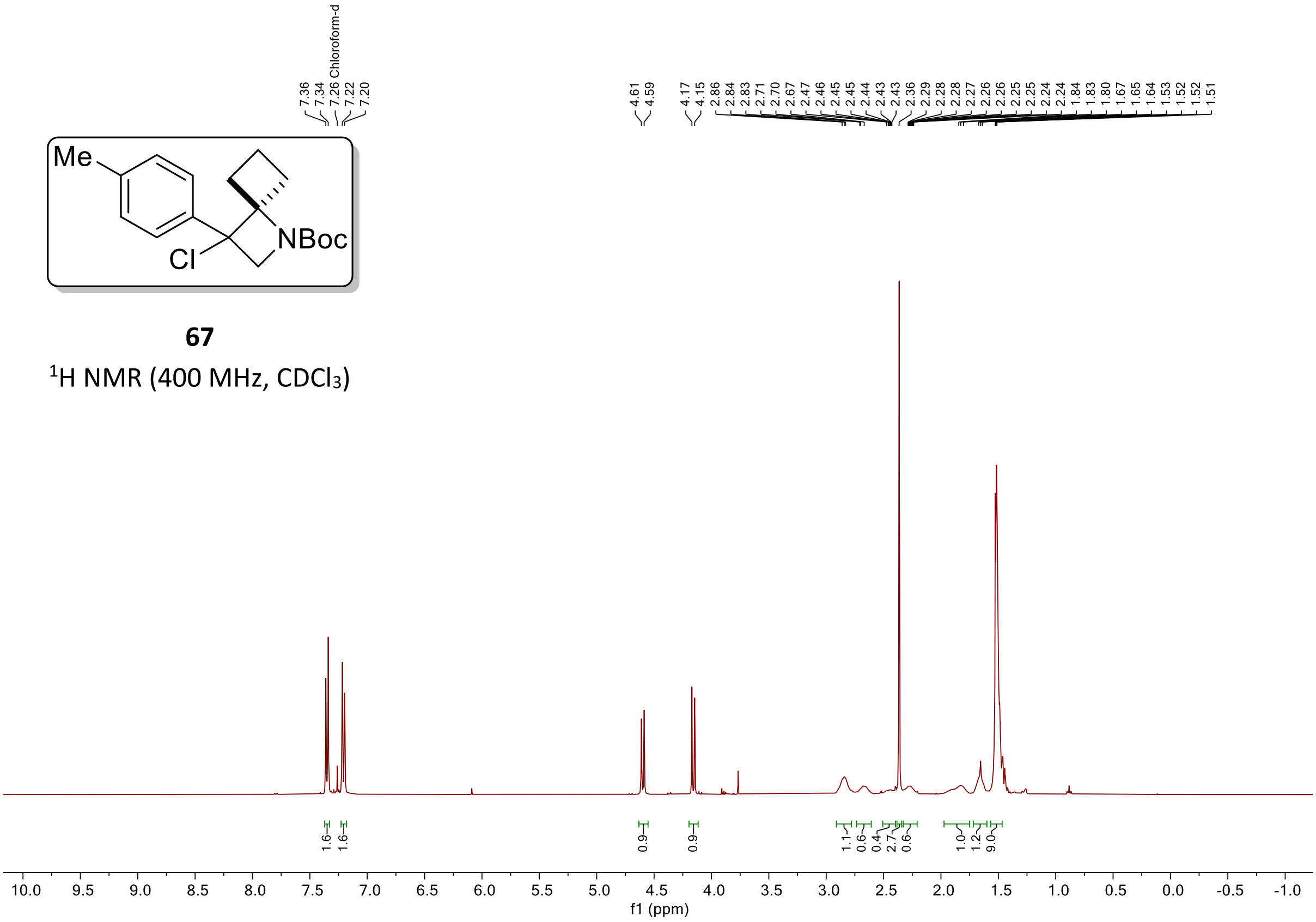


**67**

$^{13}C$ {$^{1}H$} NMR (101 MHz, $CDCl_3$)

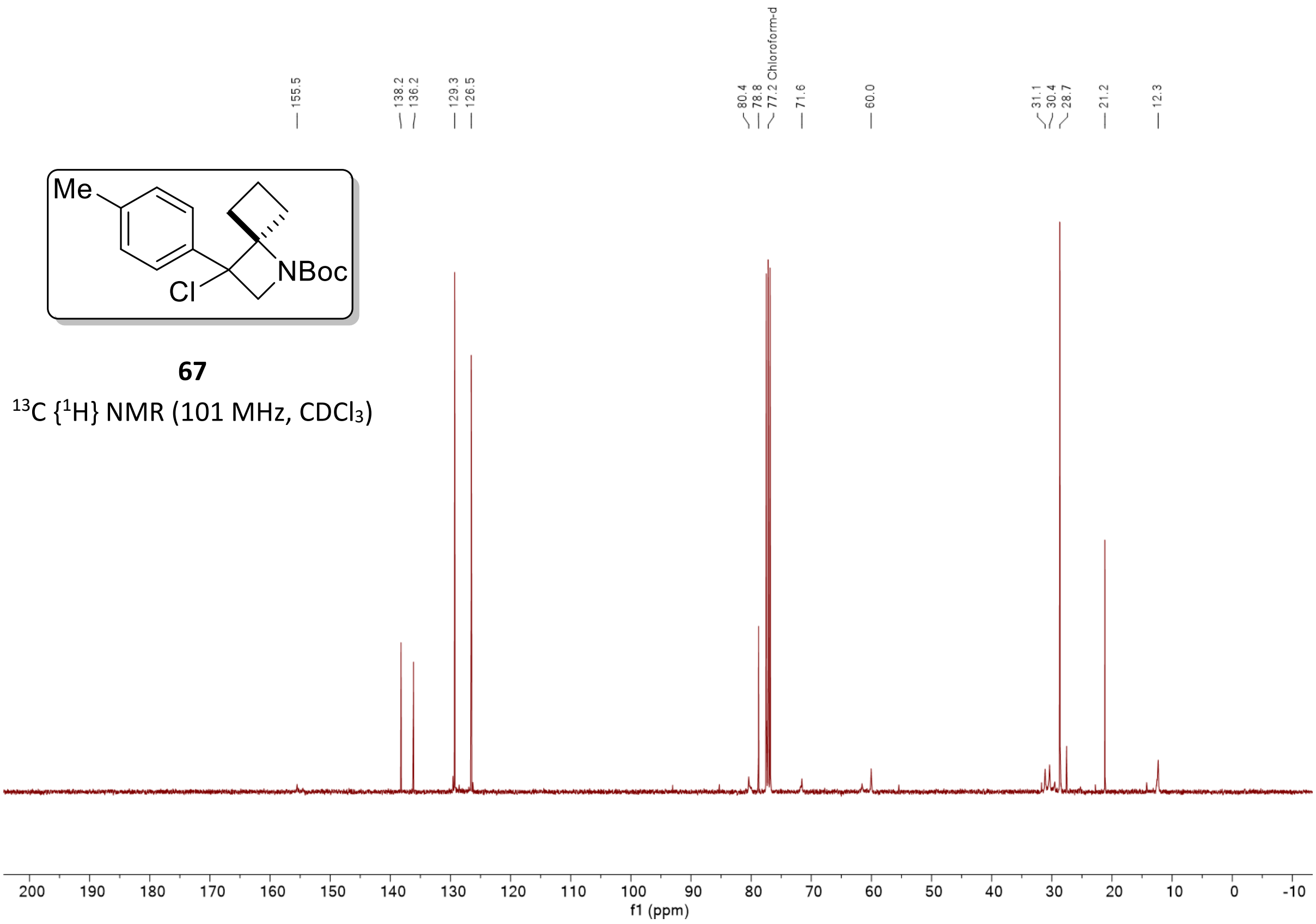

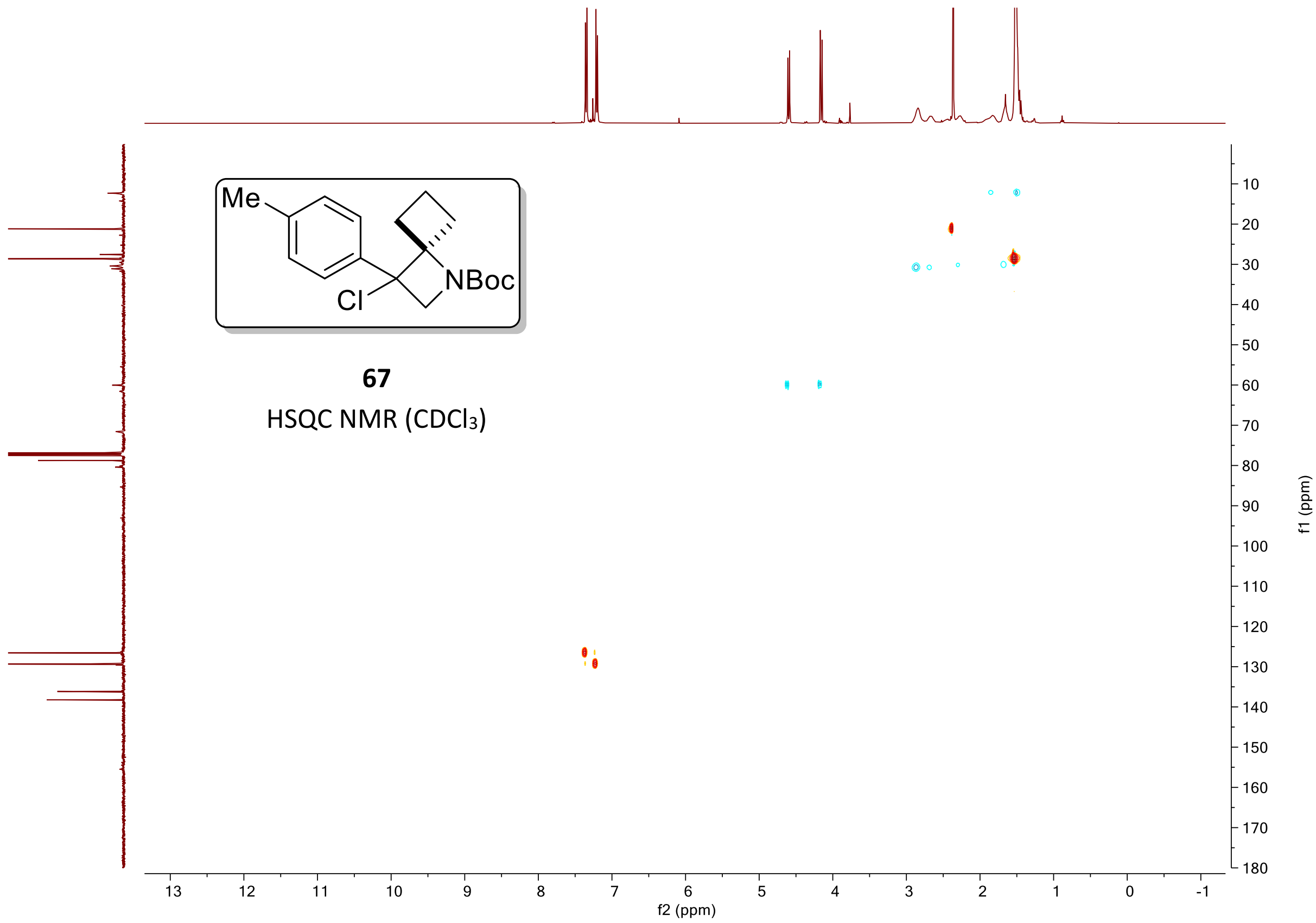

Me
Cl
NBoc
67
HSQC NMR (CDCl3)
f2 (ppm)
f1 (ppm)

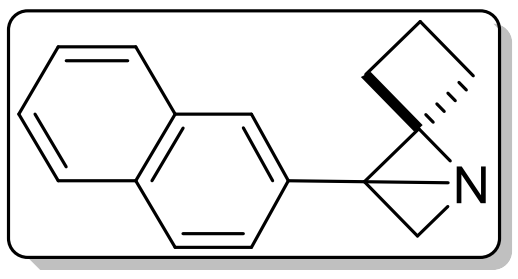

**Crude, 53**

$^{1}H$ NMR (400 MHz, $CDCl_3$)

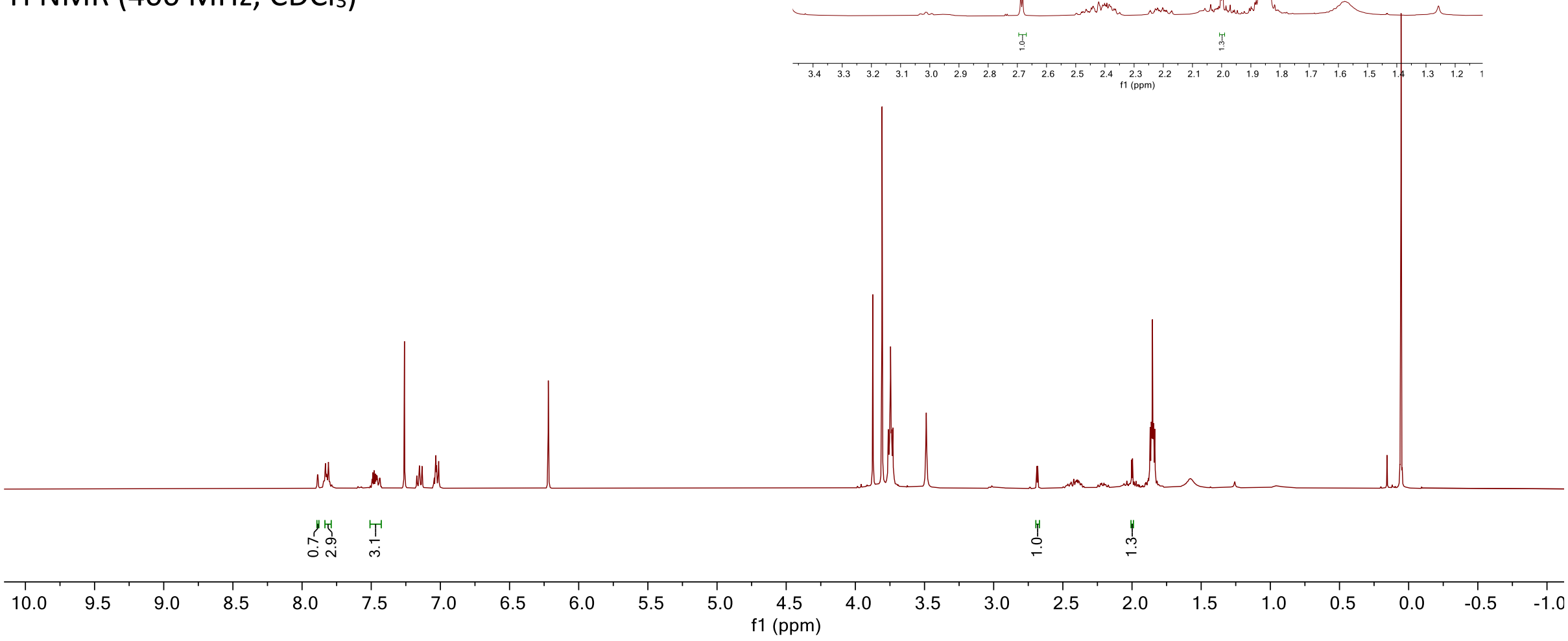

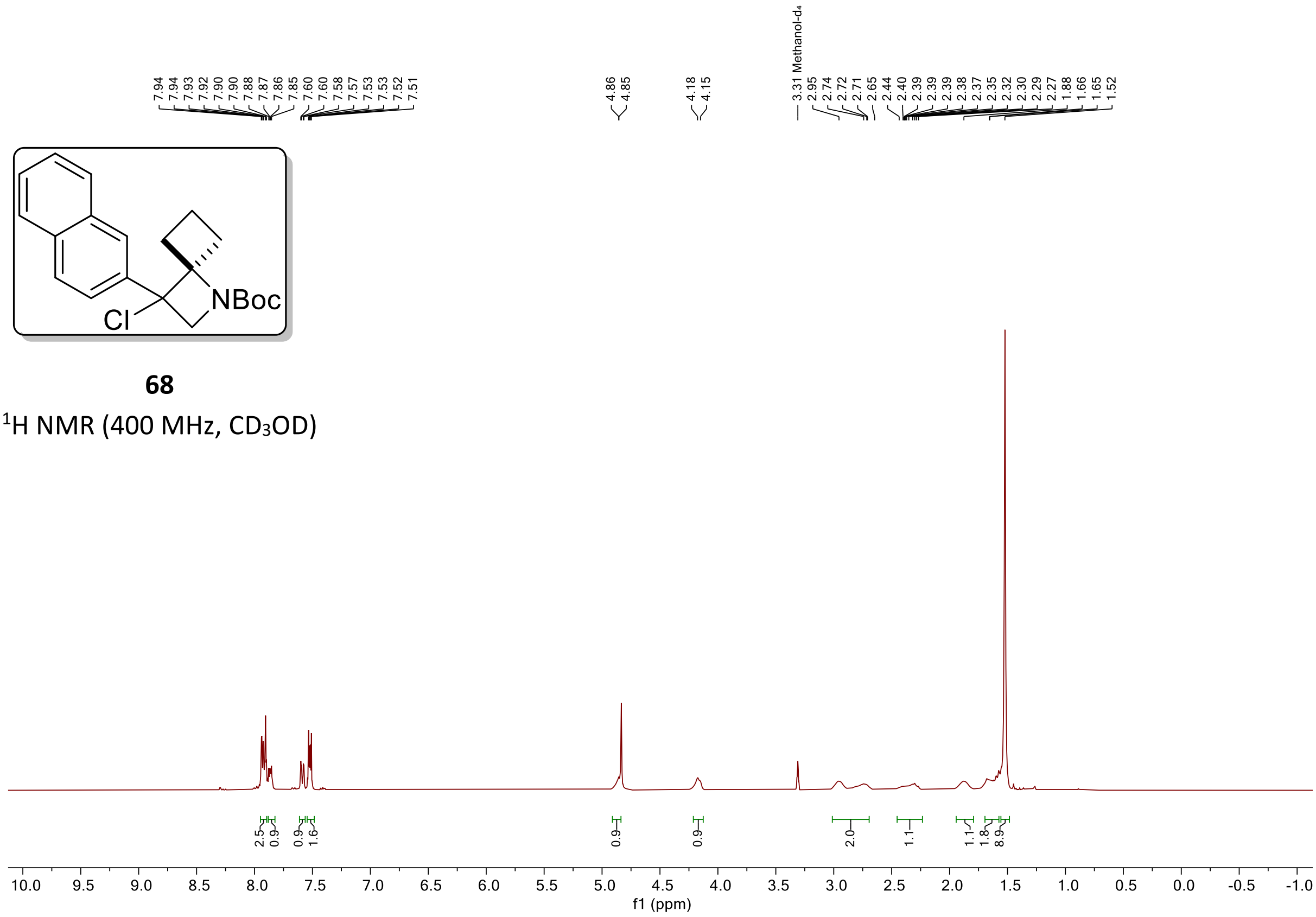

**68**

$^1$H NMR (400 MHz, $CD_3OD$)

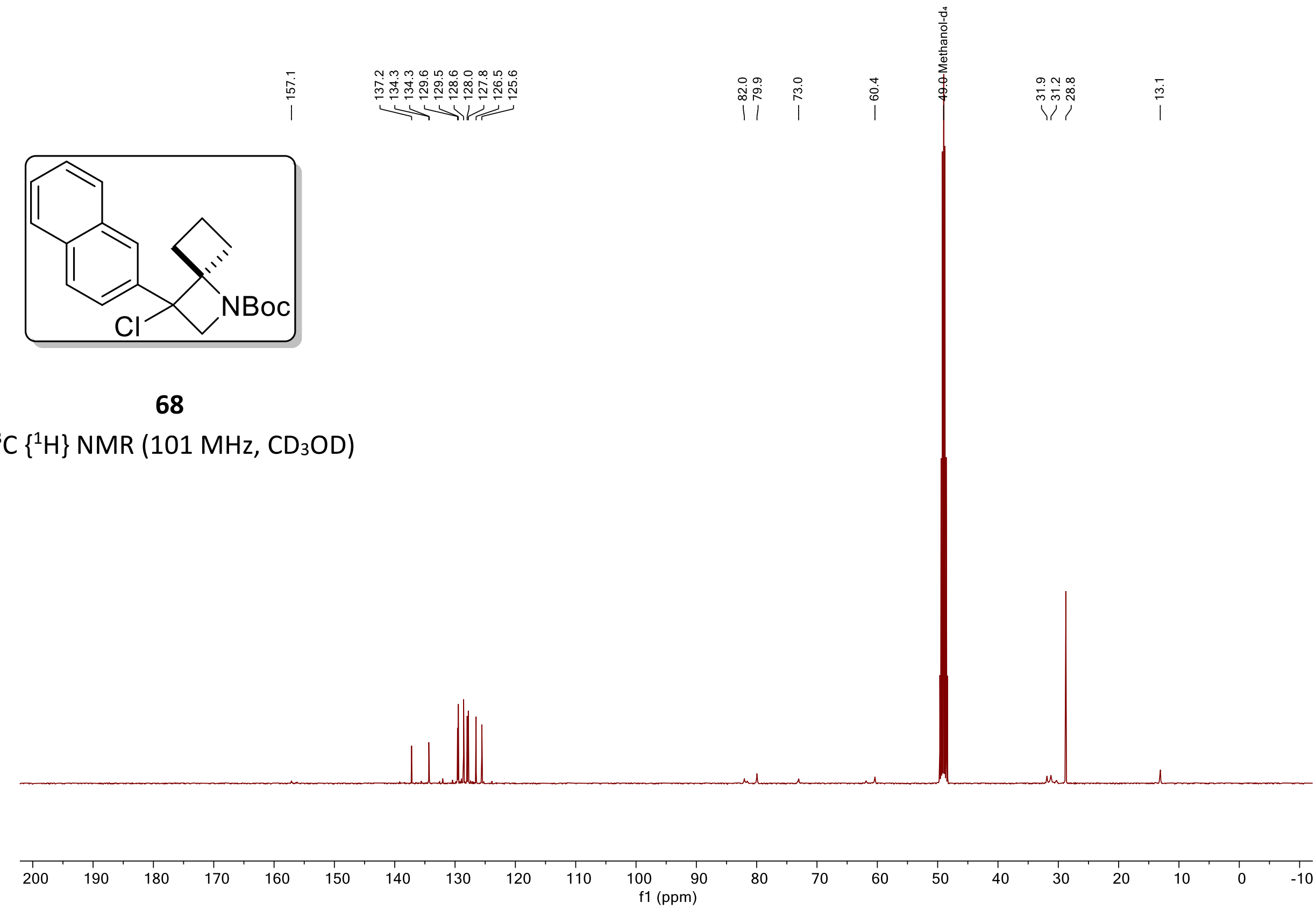

**68**

$^{13}$C {$^1$H} NMR (101 MHz, $CD_3OD$)

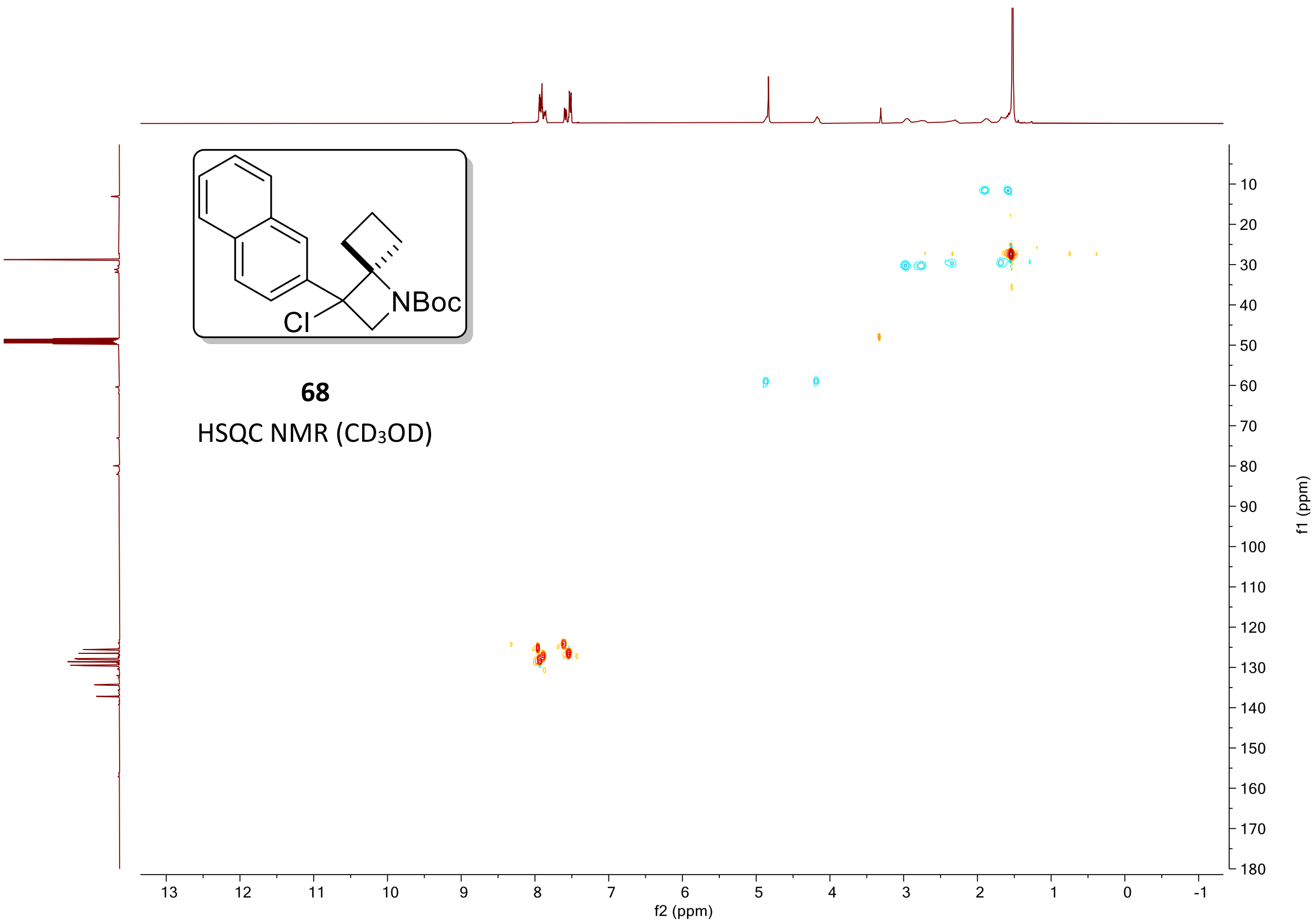
NBoc
Cl
68
HSQC NMR (CD3OD)
f2 (ppm)
f1 (ppm)

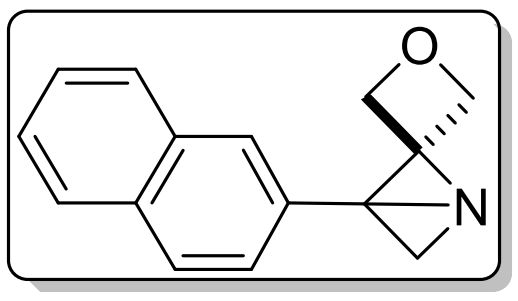

**Crude, 40**

$^{1}$H NMR (400 MHz, $CDCl_3$)

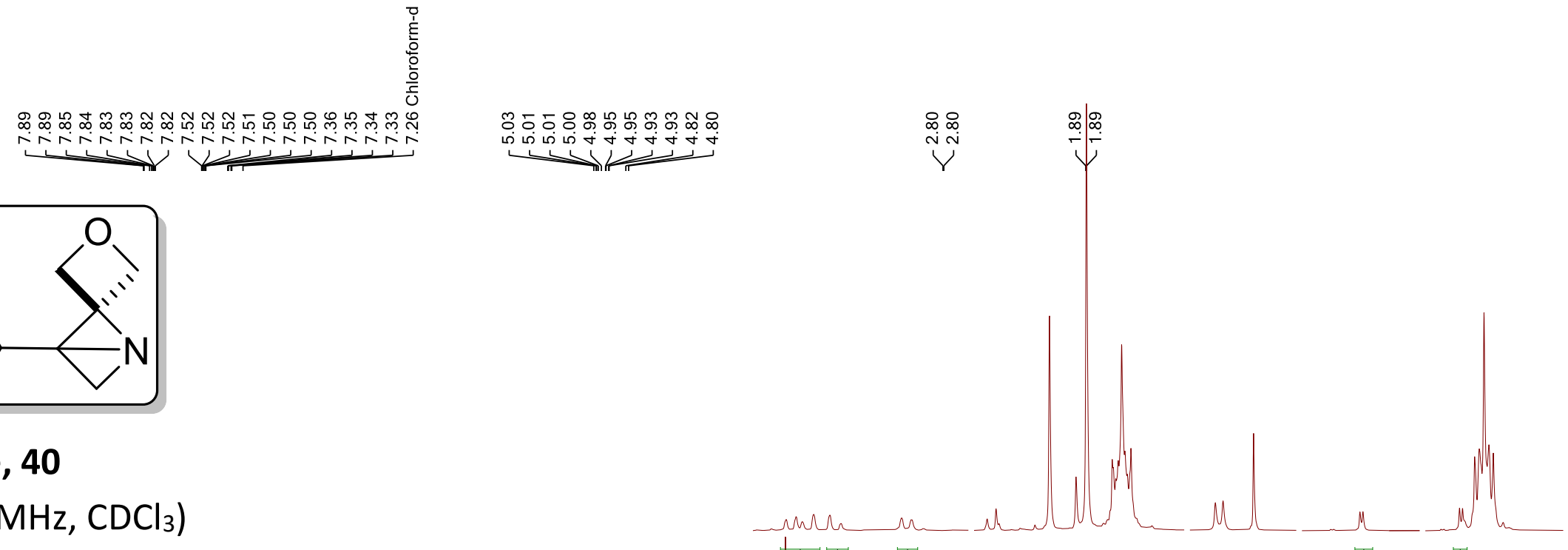

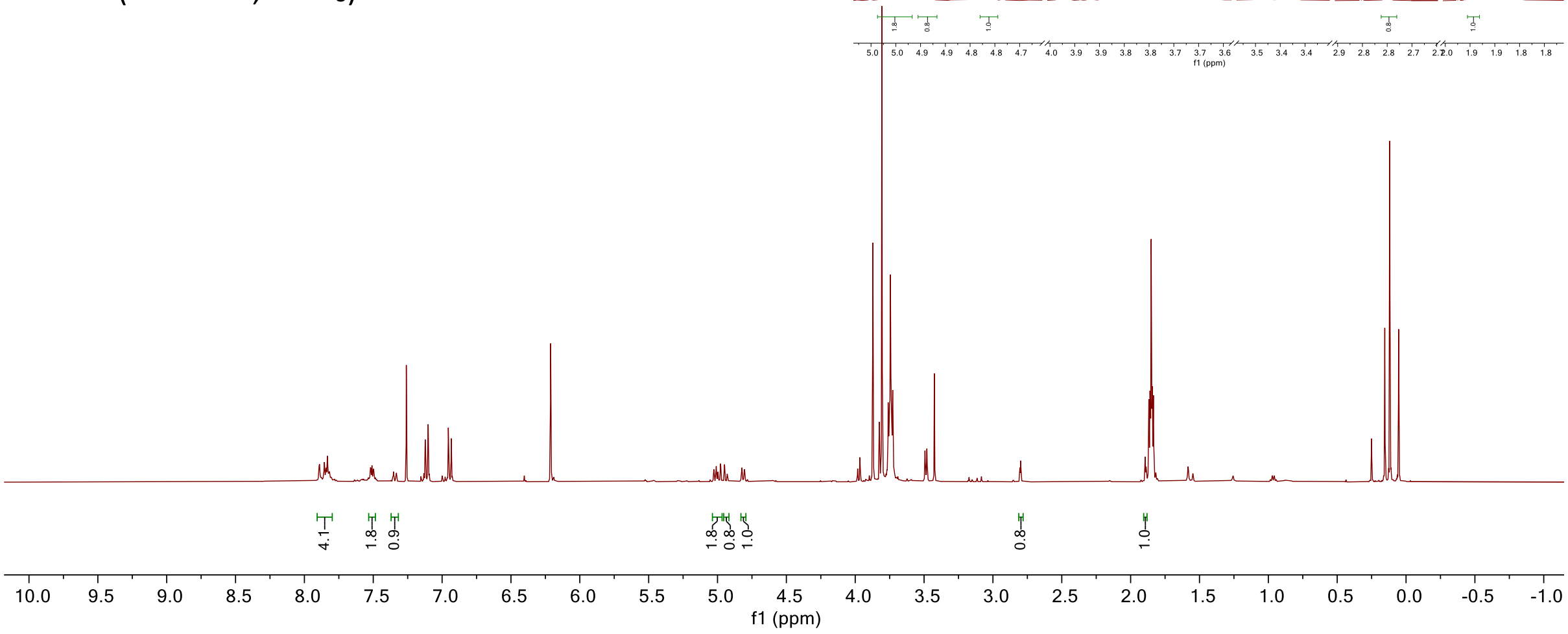

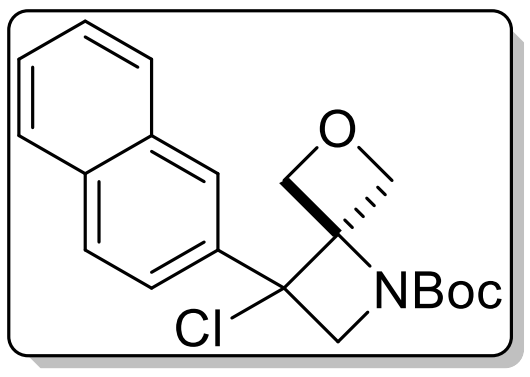


**69**

$^{1}$H NMR (400 MHz, $CDCl_3$)

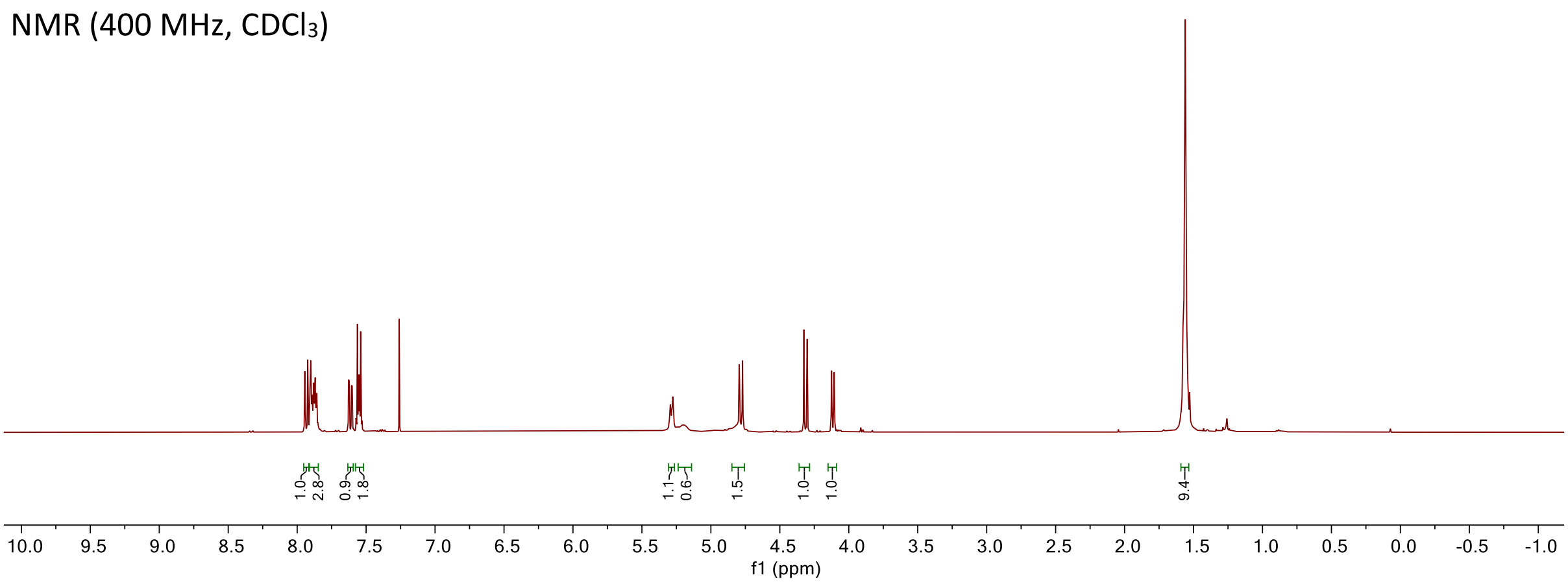


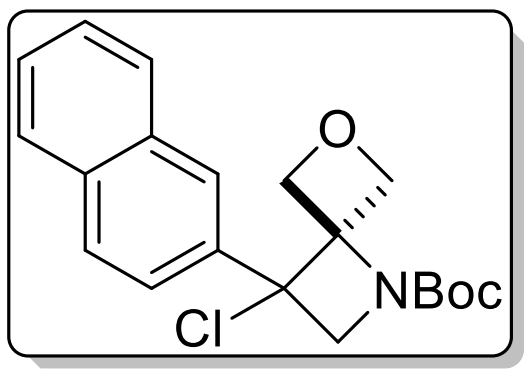


**69**

$^{13}$C {$^{1}$H} NMR (101 MHz, $CDCl_3$)

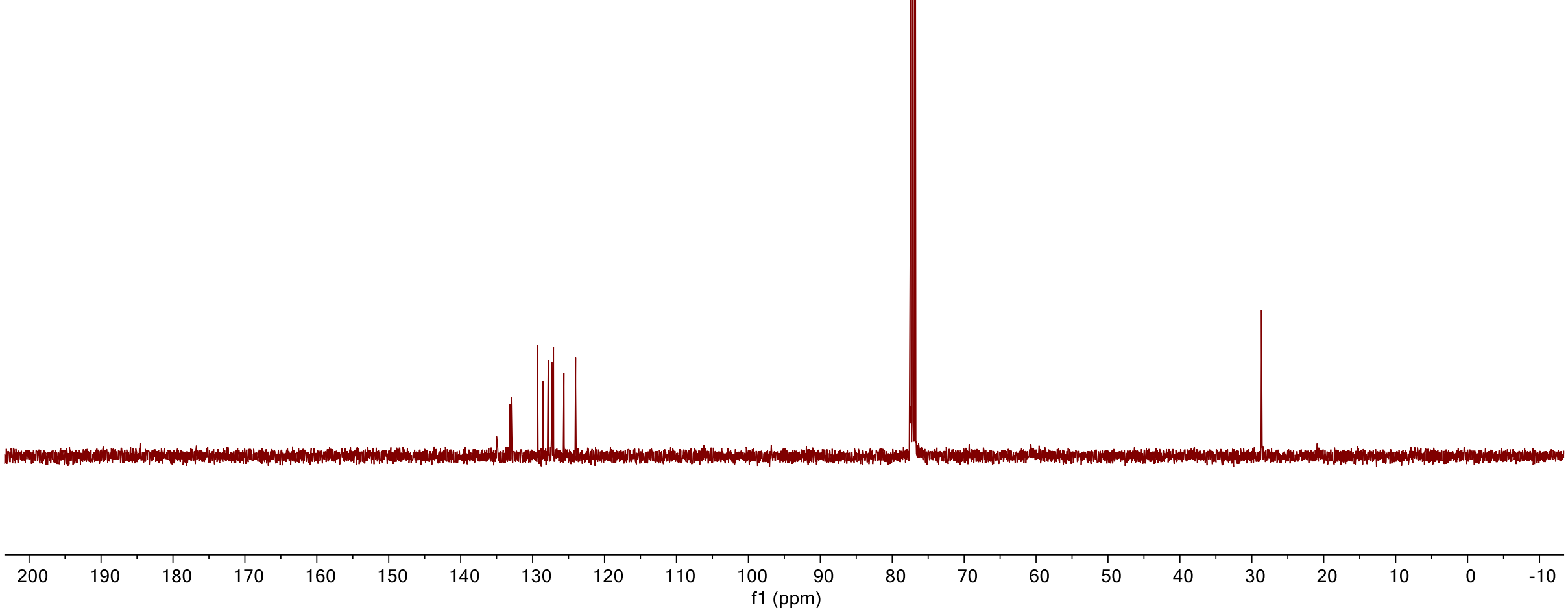

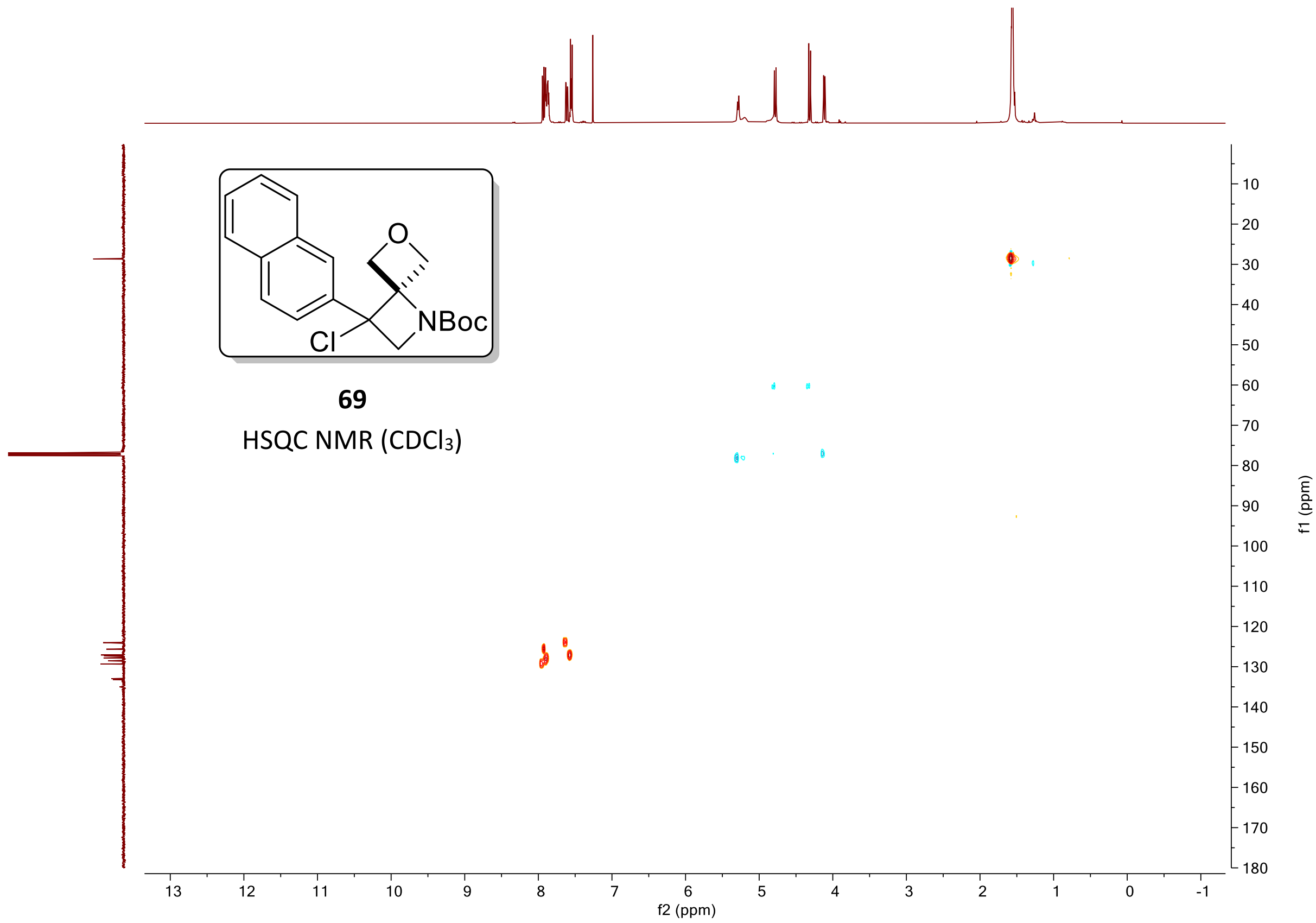
O
NBoc
Cl
69
HSQC NMR (CDCl3)
f1 (ppm)
f2 (ppm)

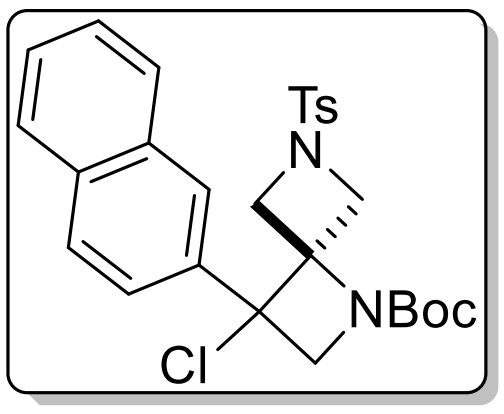


**70**

$^{1}$H NMR (400 MHz, $CDCl_3$)

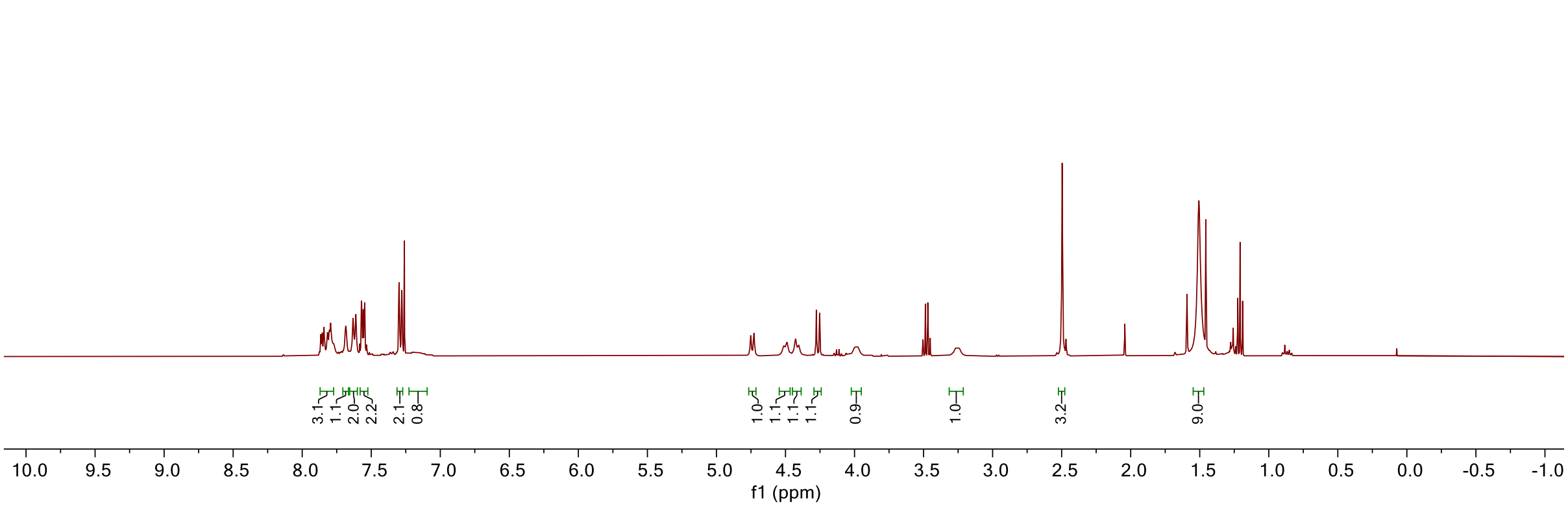


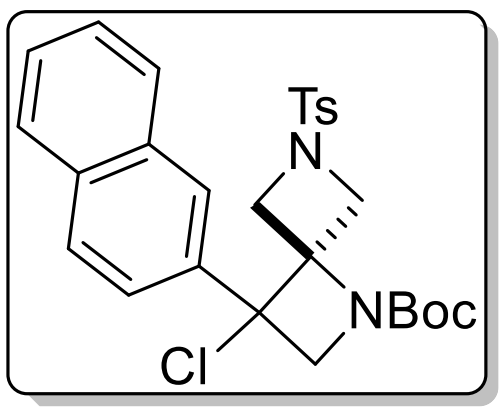


**70**

$^{13}$C {$^{1}$H} NMR (101 MHz, $CDCl_3$)

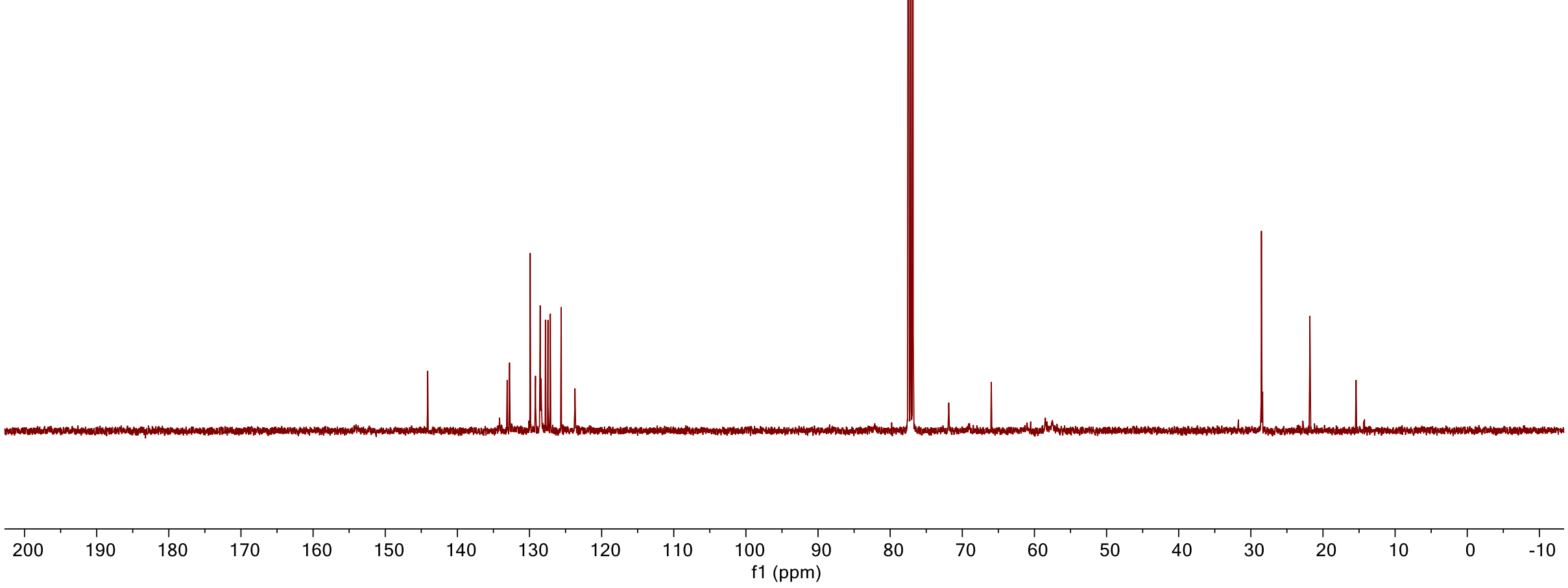

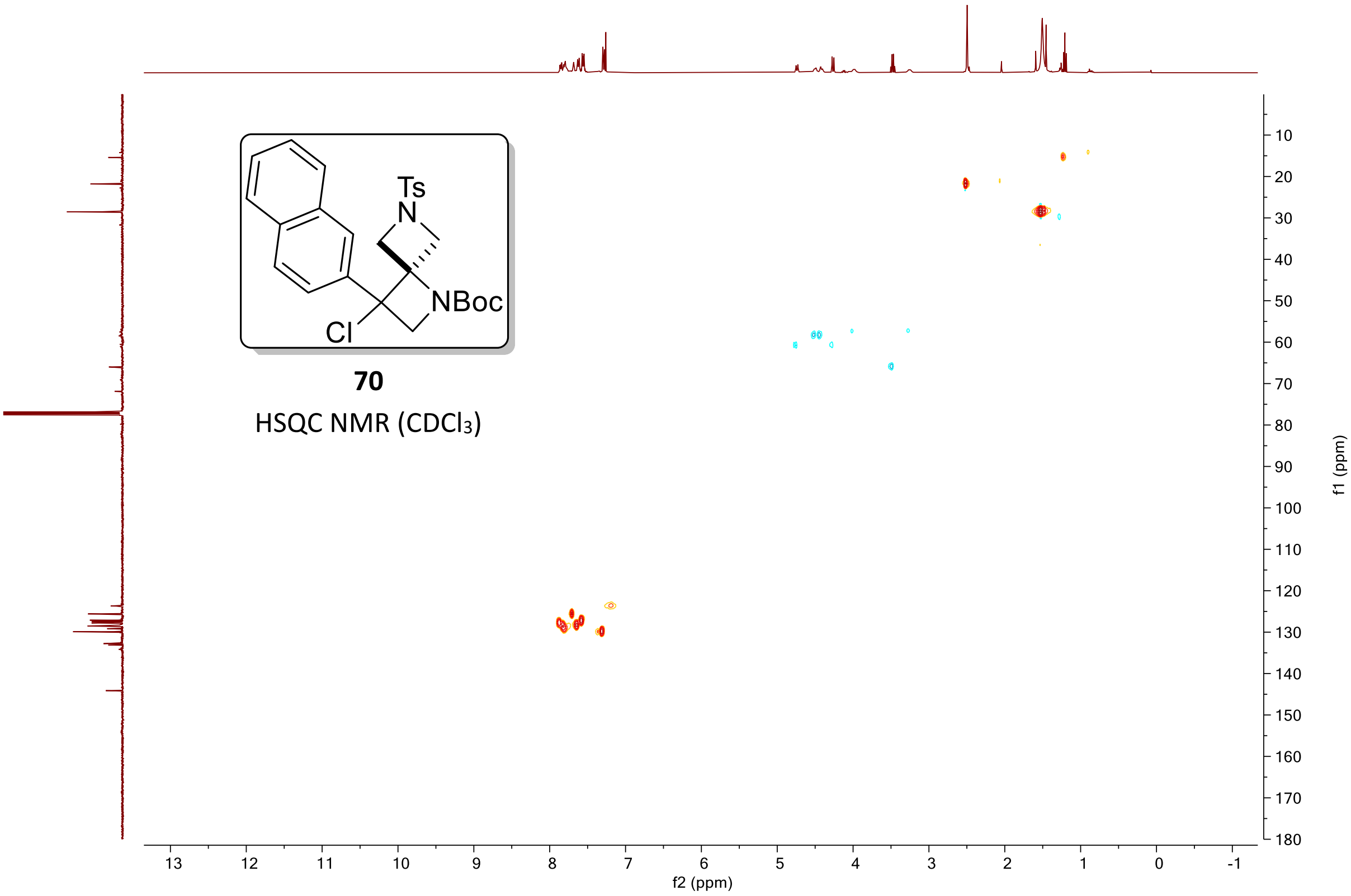

**70**

HSQC NMR ($CDCl_3$)

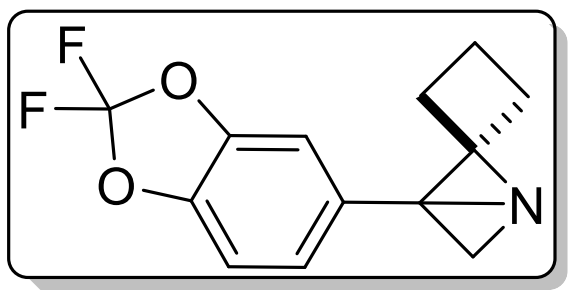

**Crude, 54**

$^1H$ NMR (400 MHz, $CDCl_3$)

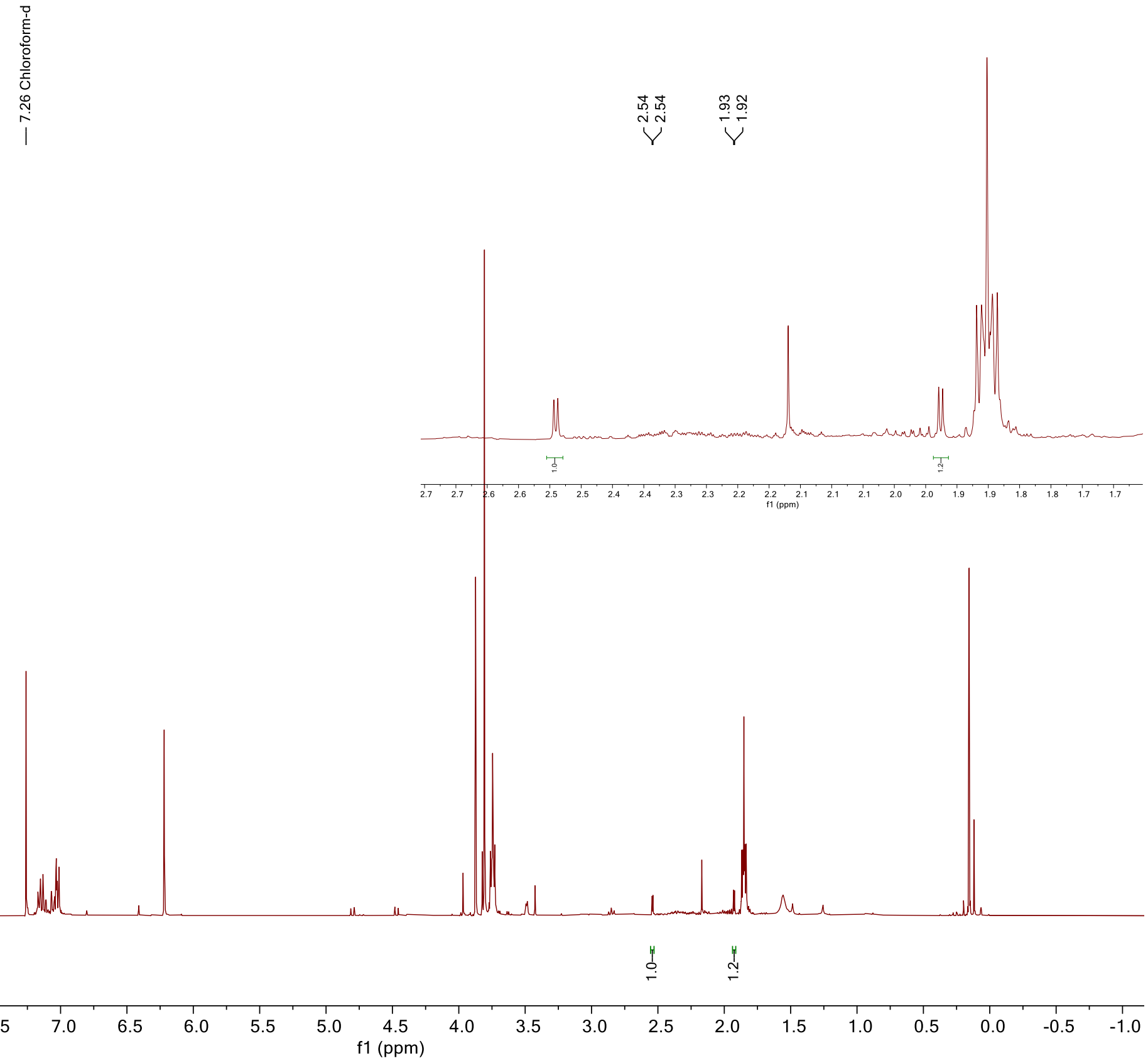

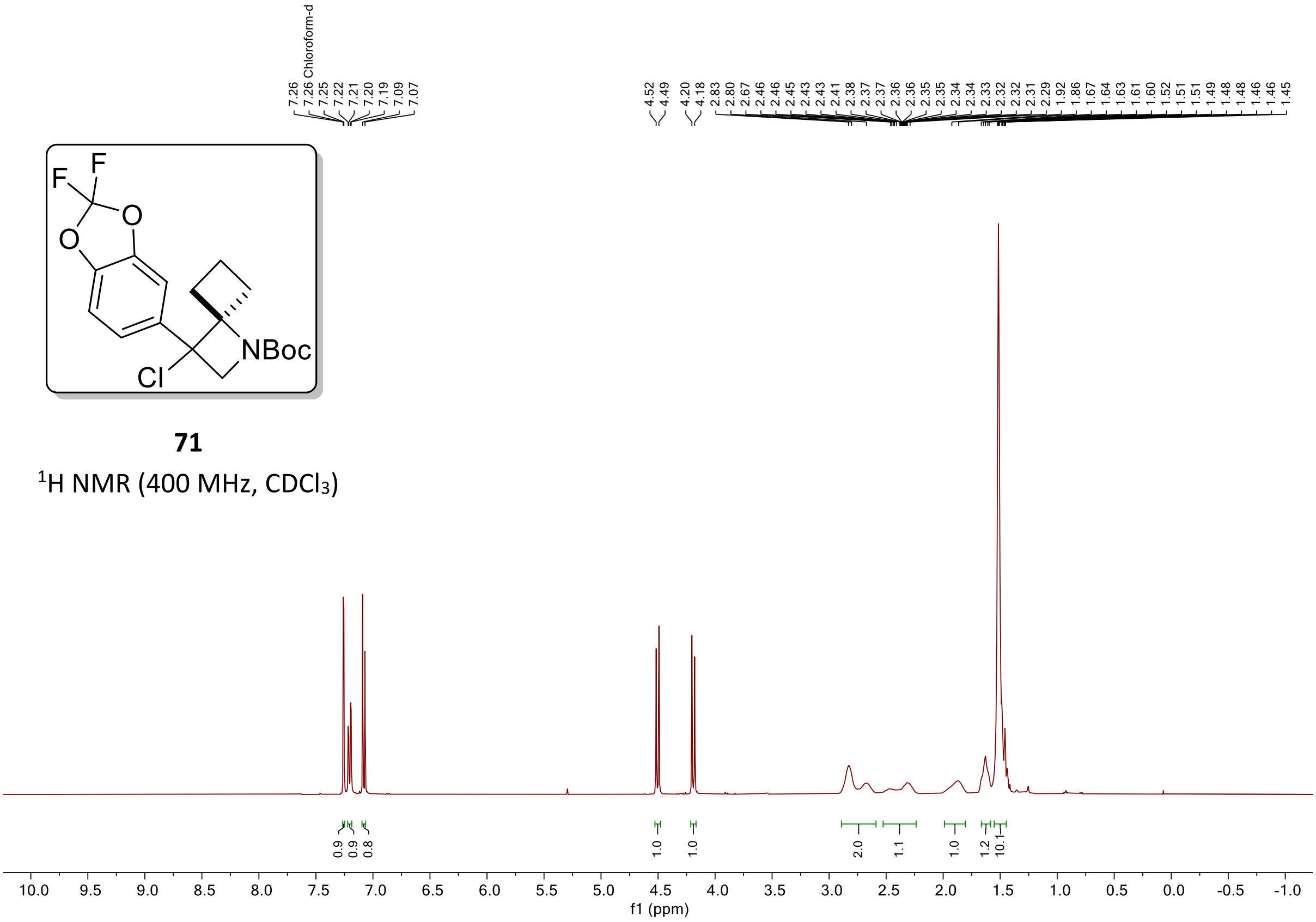


**71**

$^{1}$H NMR (400 MHz, $CDCl_3$)

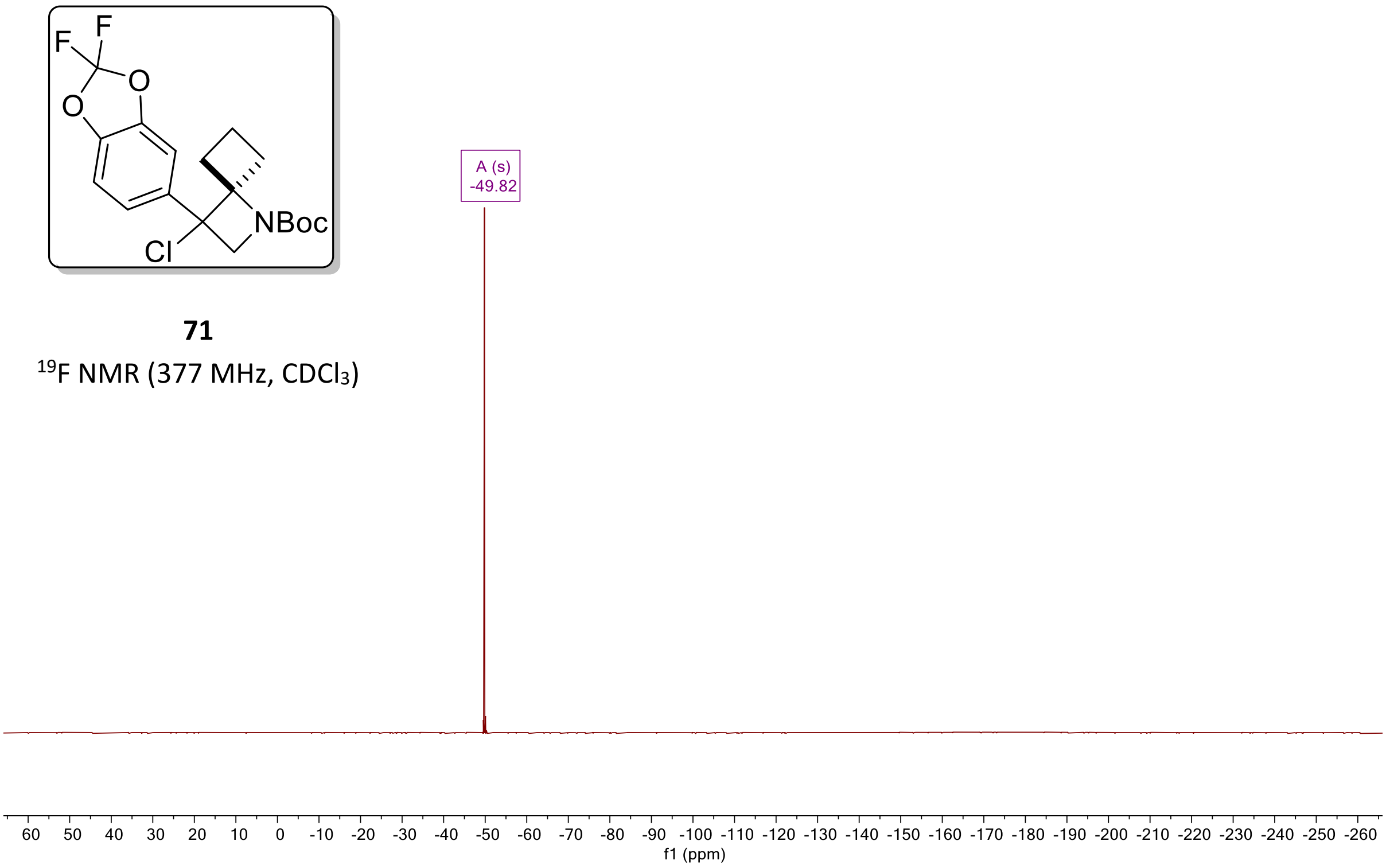


**71**

$^{19}$F NMR (377 MHz, $CDCl_3$)

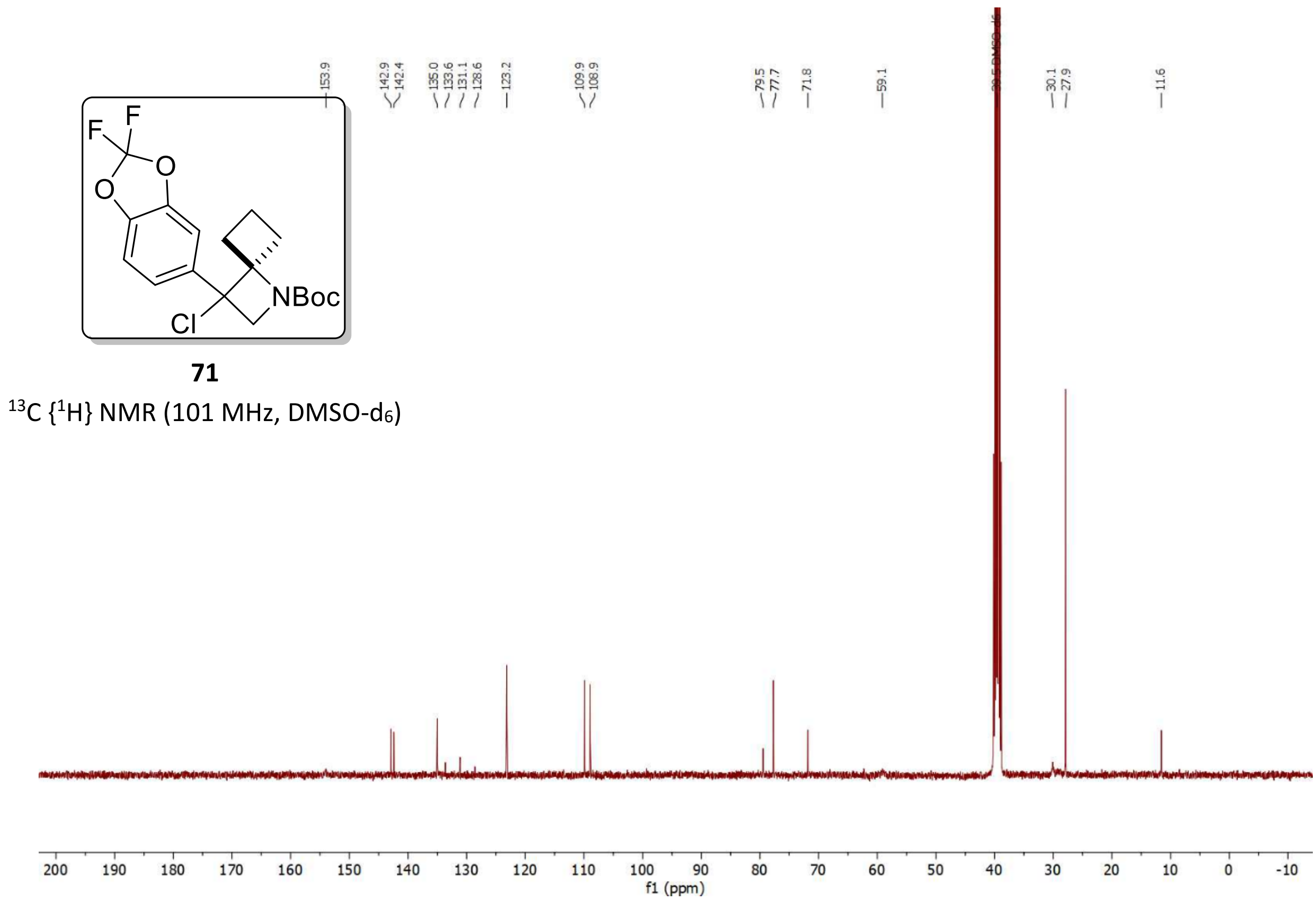

**71**

$^{13}C$ {$^{1}H$} NMR (101 MHz, DMSO-d$_6$)

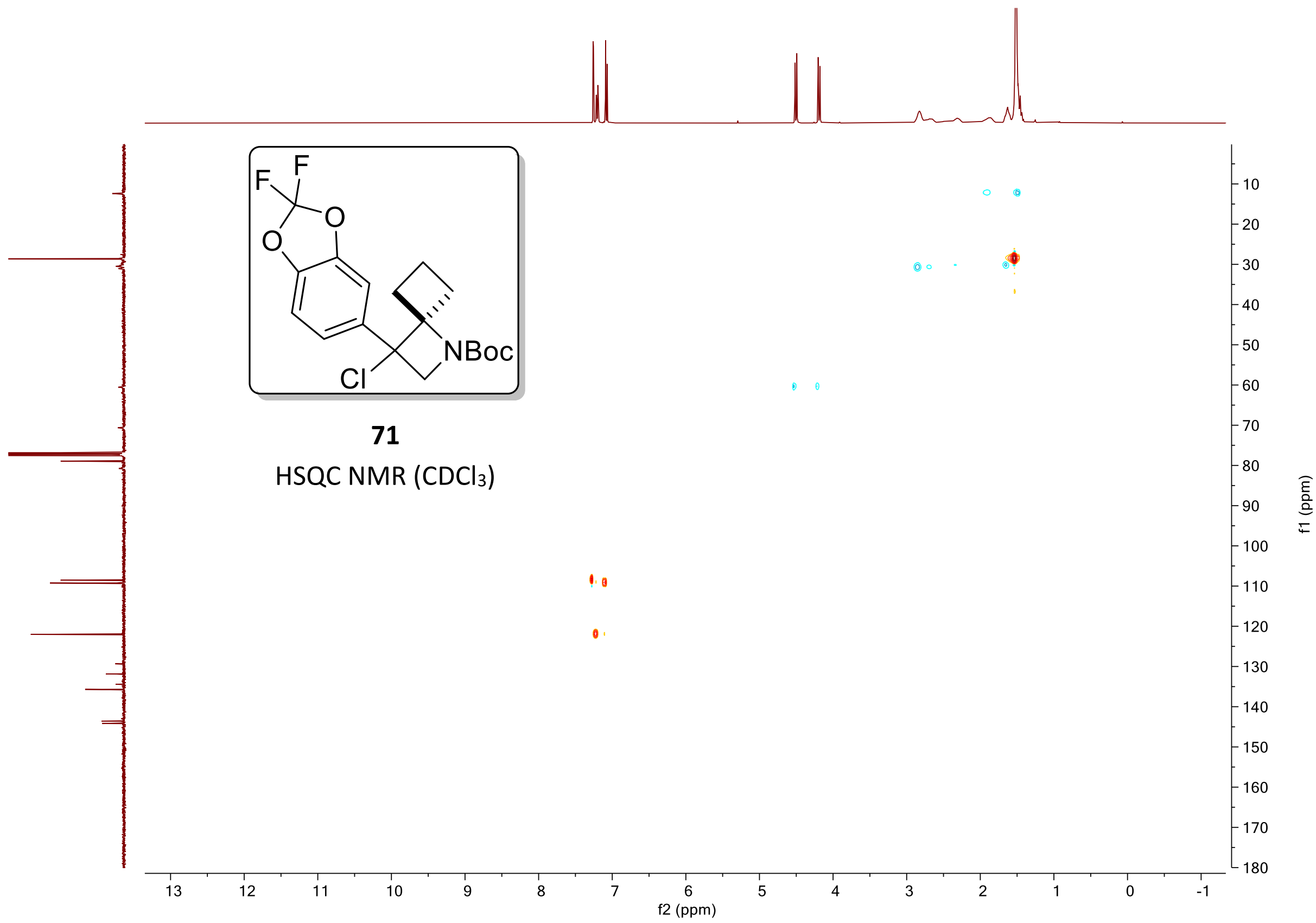

**71**

HSQC NMR ($CDCl_3$)

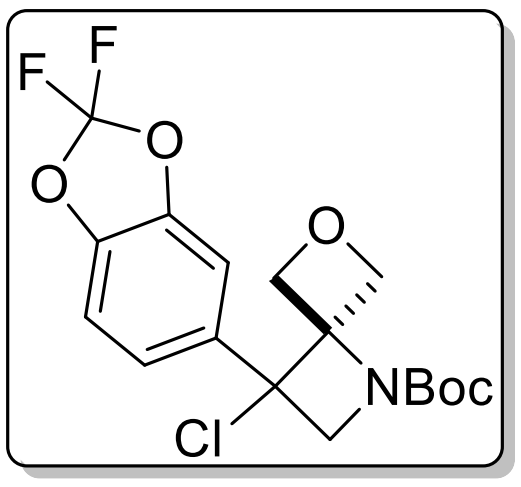

**72**

$^{1}$H NMR (400 MHz, $CDCl_3$)

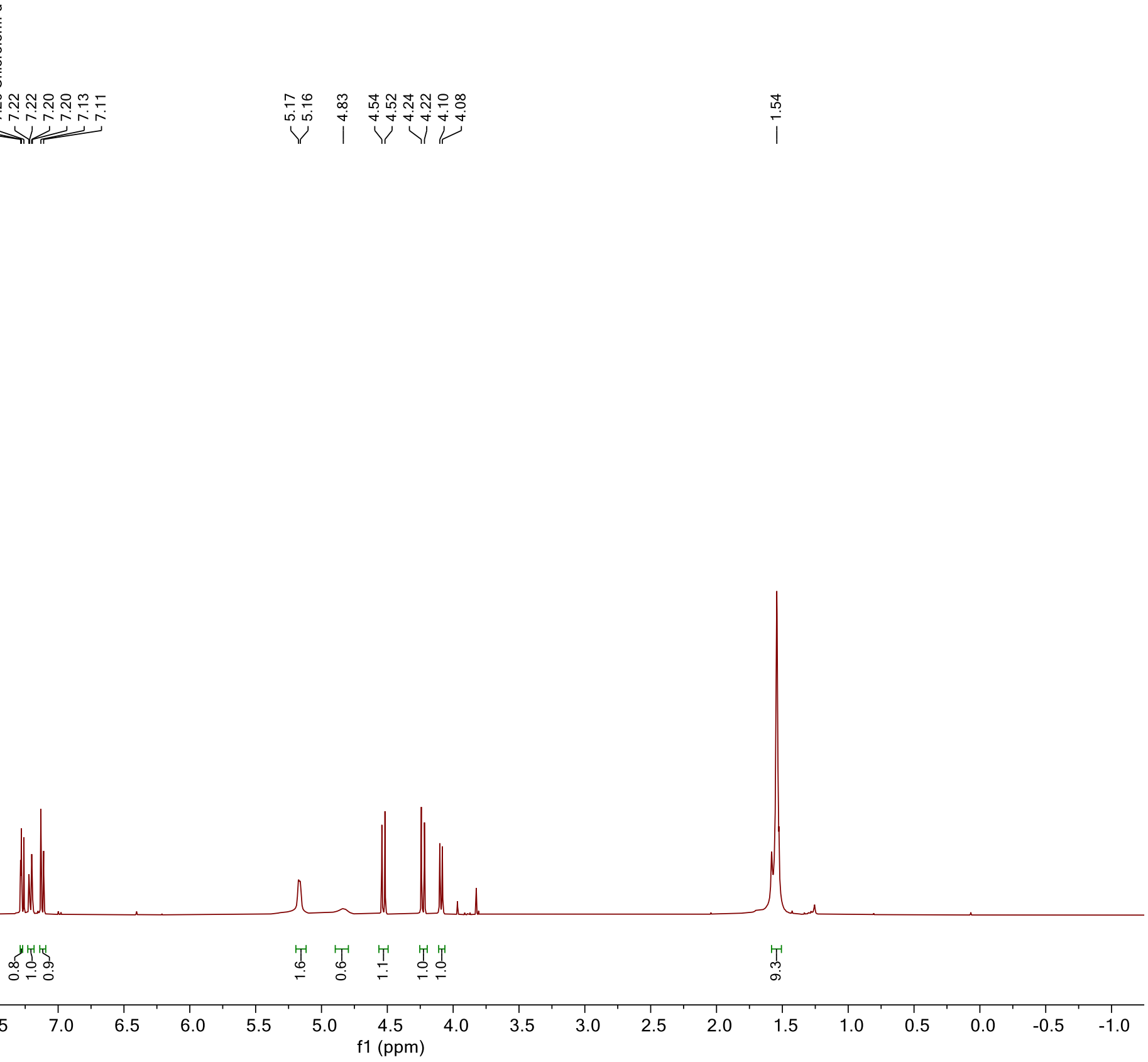


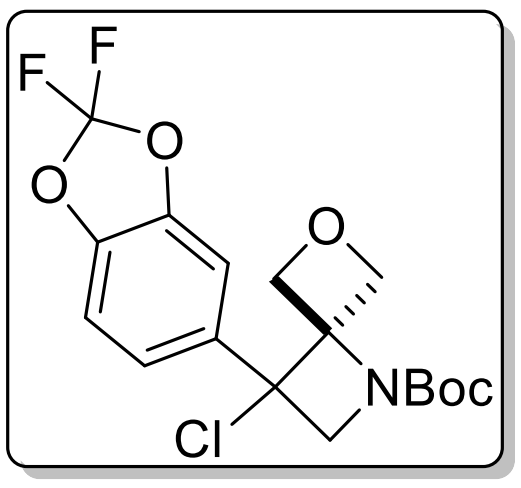

**72**

$^{19}$F NMR (377 MHz, $CDCl_3$)

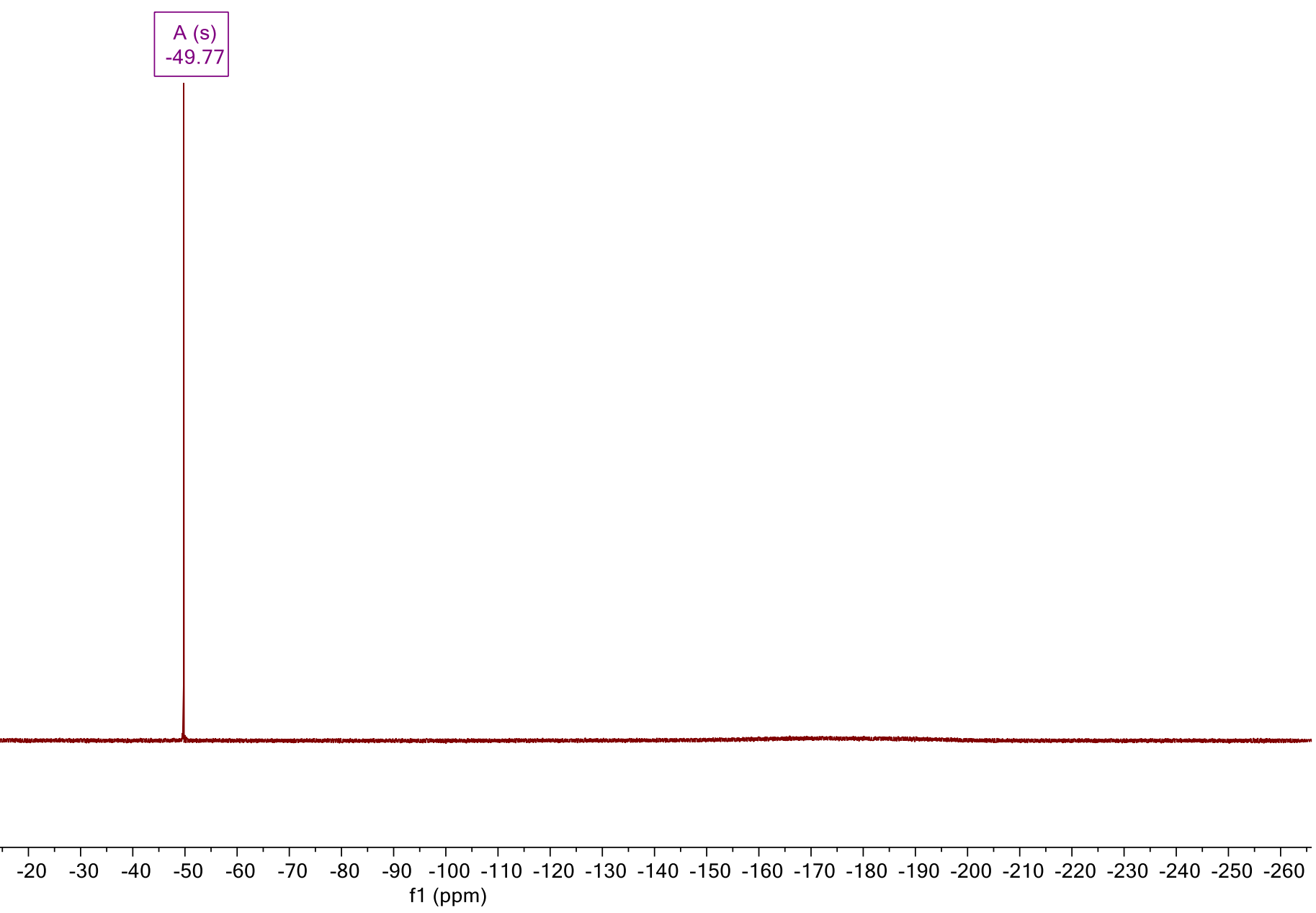

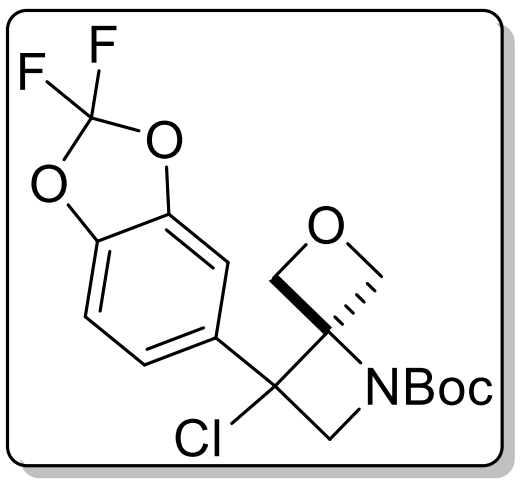

**72**

$^{13}C\ \{^{1}H\}$ NMR (101 MHz, $CDCl_3$)

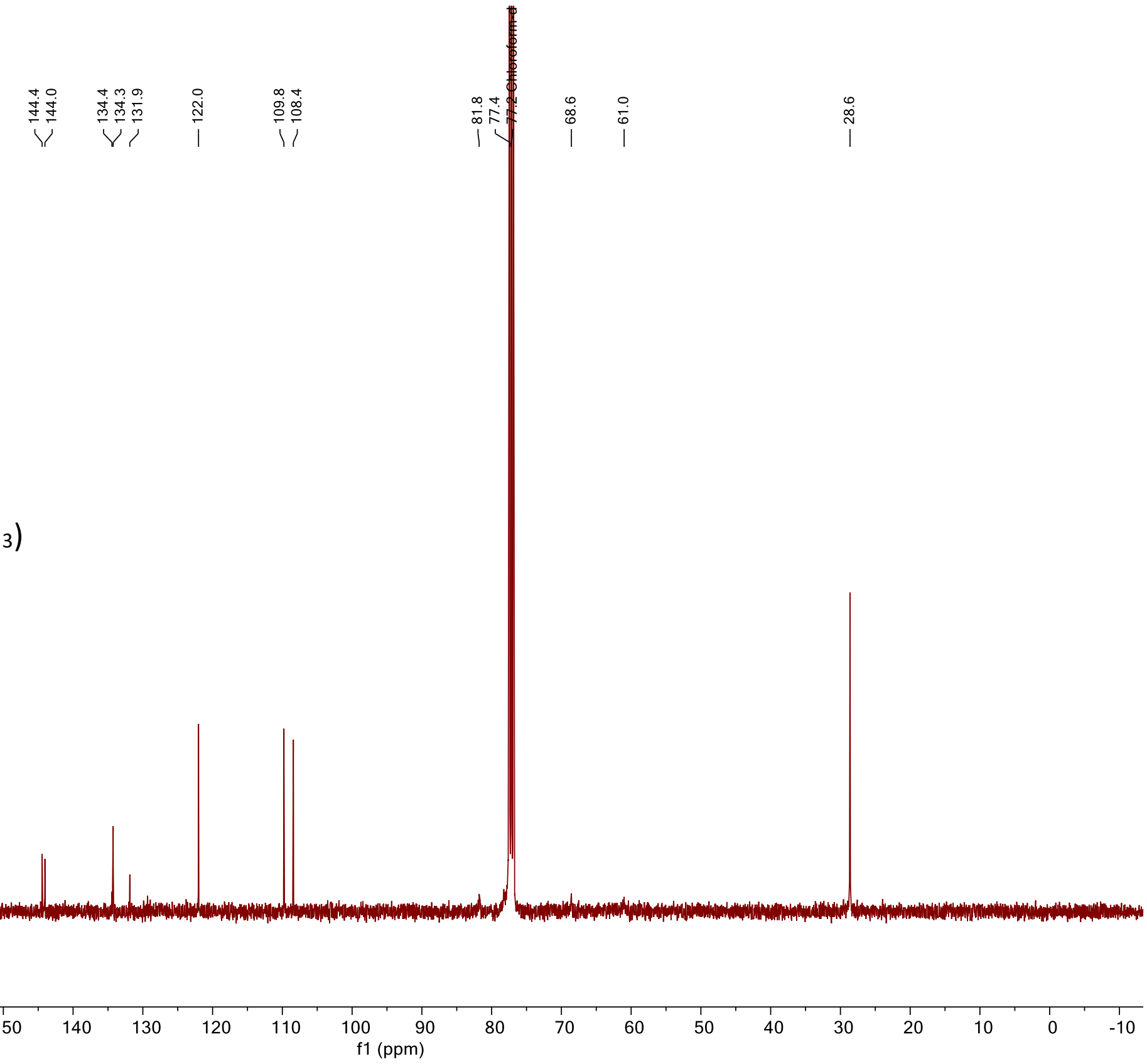


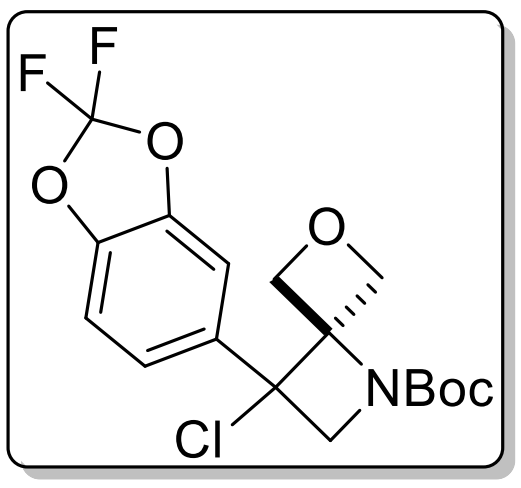

**72**

HSQC NMR ($CDCl_3$)

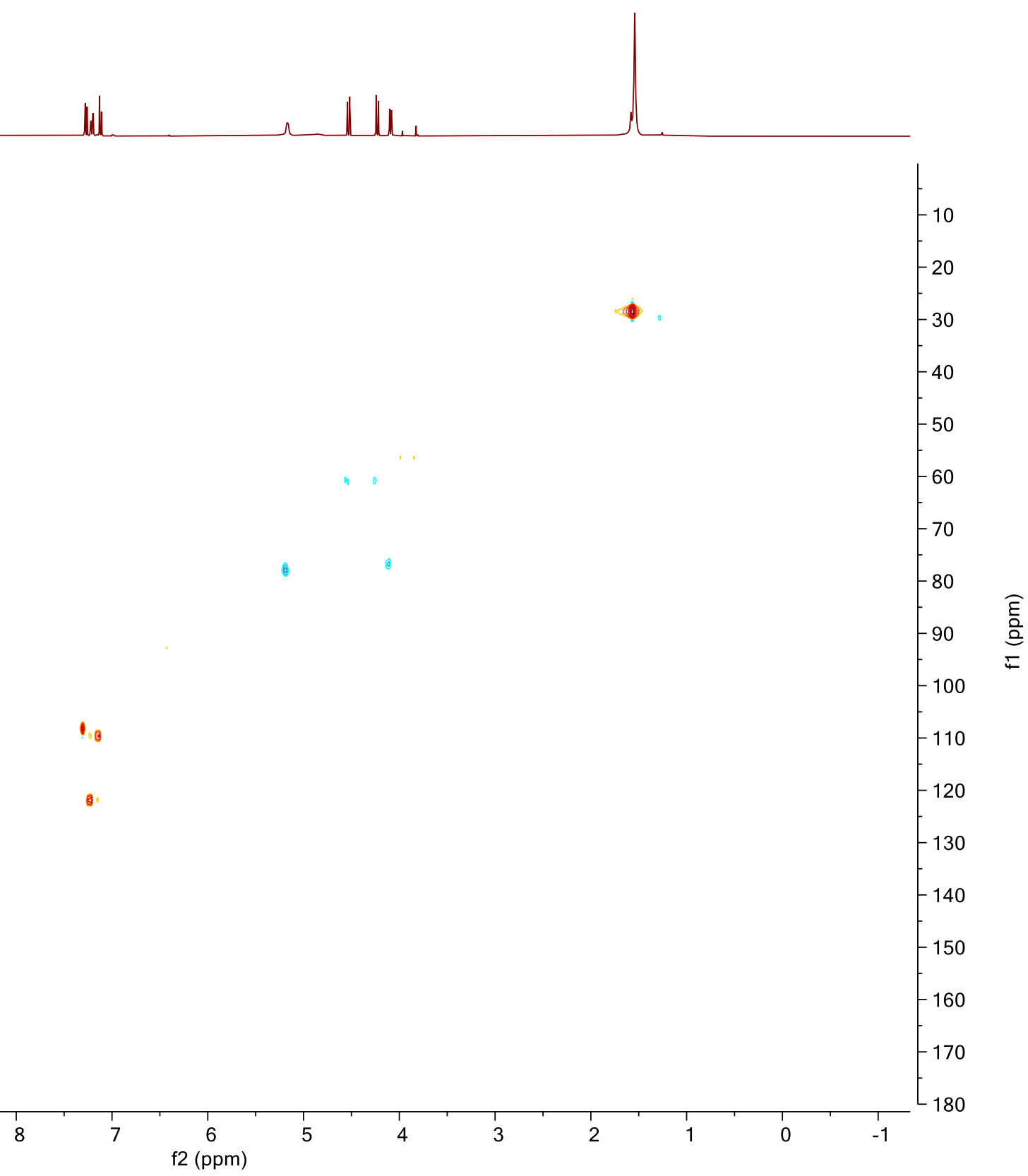

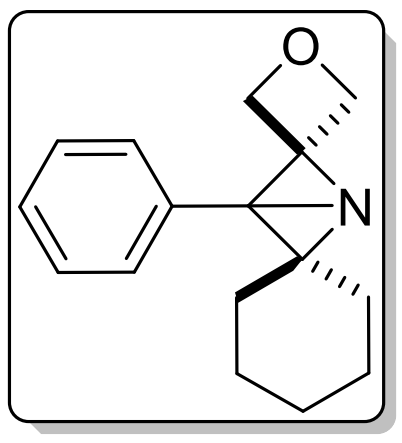


**Crude, 42**

$^{1}$H NMR (400 MHz, $CDCl_3$)

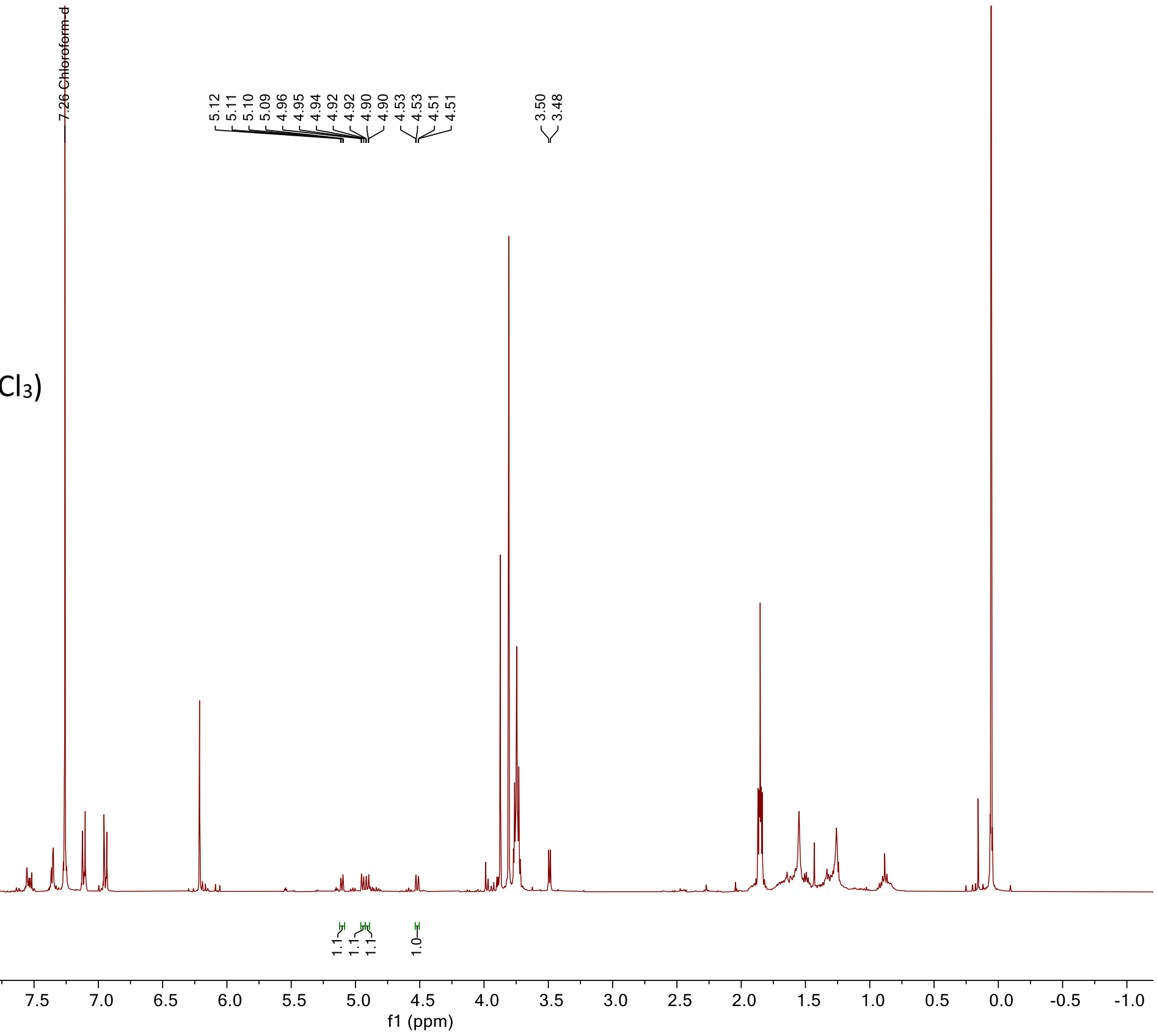

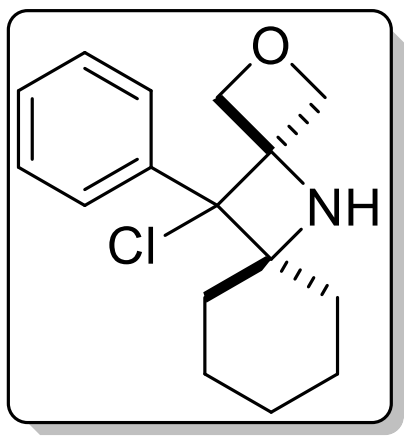

**73**

$^1$H NMR (400 MHz, $CDCl_3$)

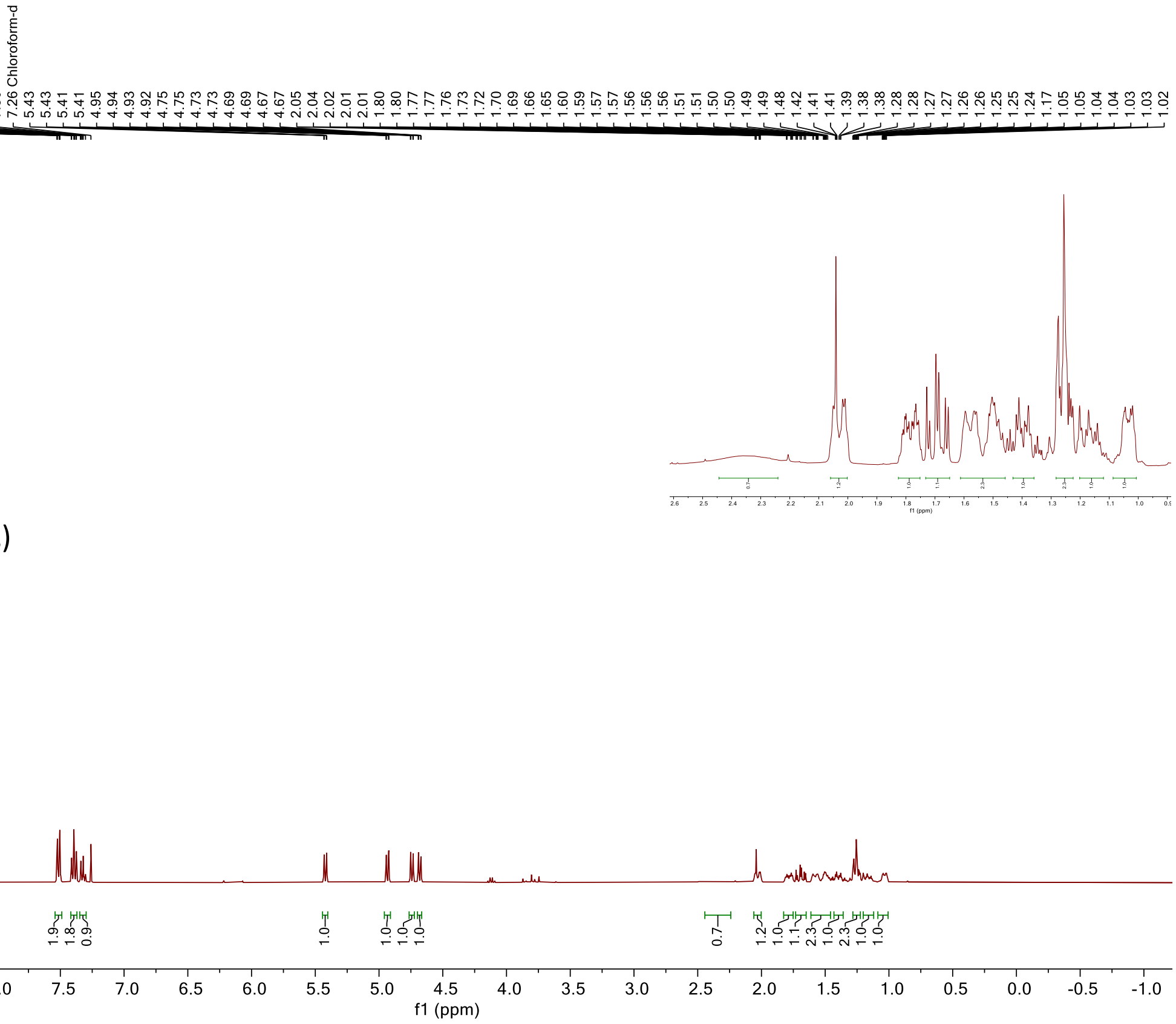

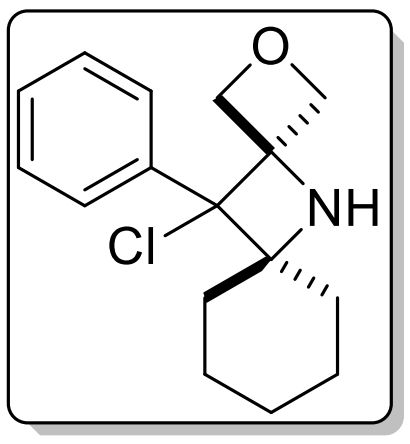

**73**

$^{13}$C {$^1$H} NMR (101 MHz, $CDCl_3$)

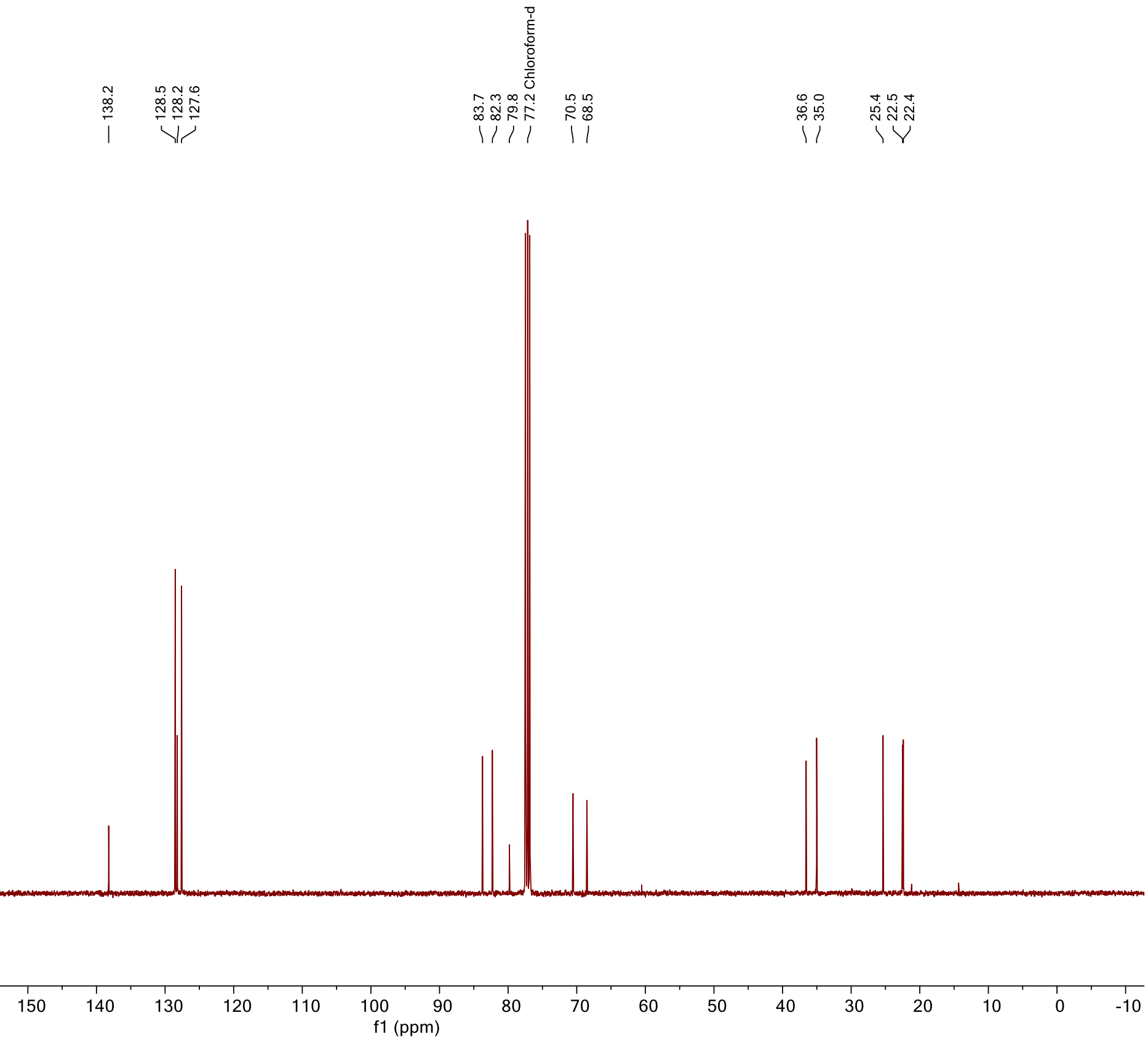

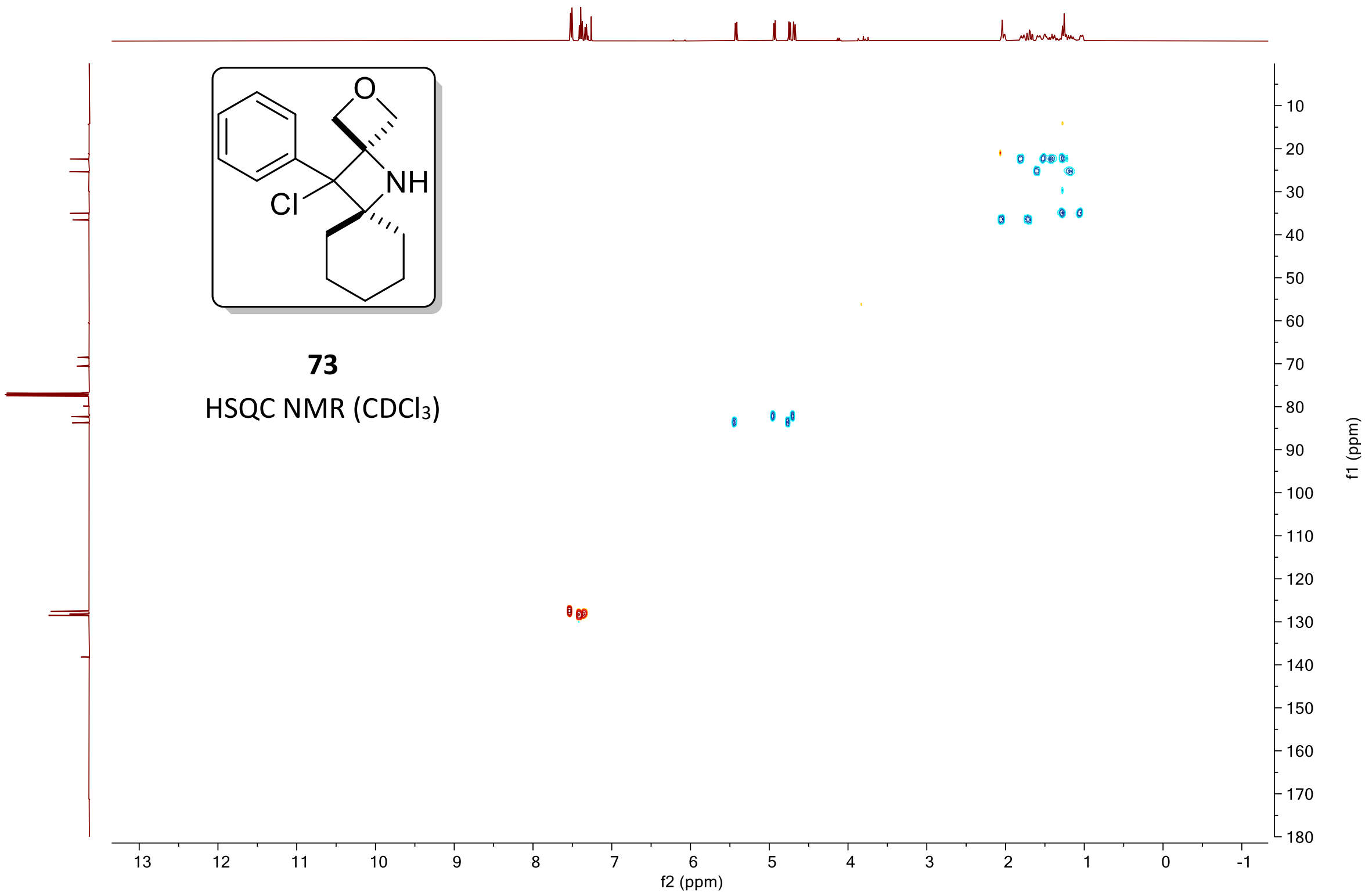


**73**

HSQC NMR ($CDCl_3$)

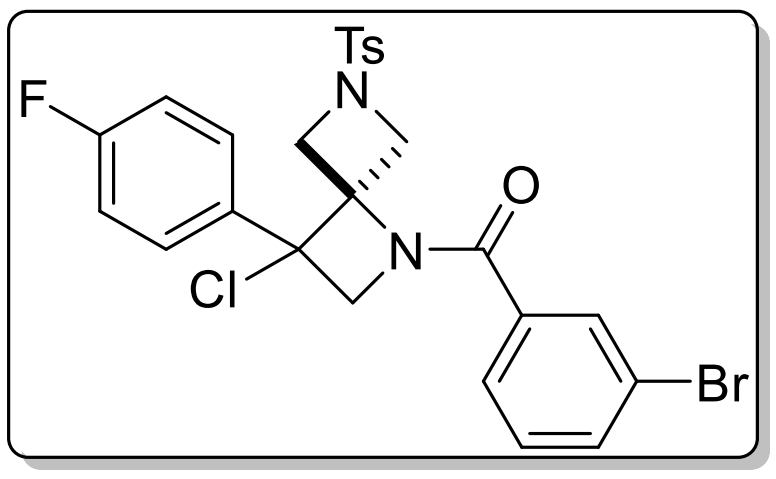

**74**

$^1H$ NMR (400 MHz, $CDCl_3$)

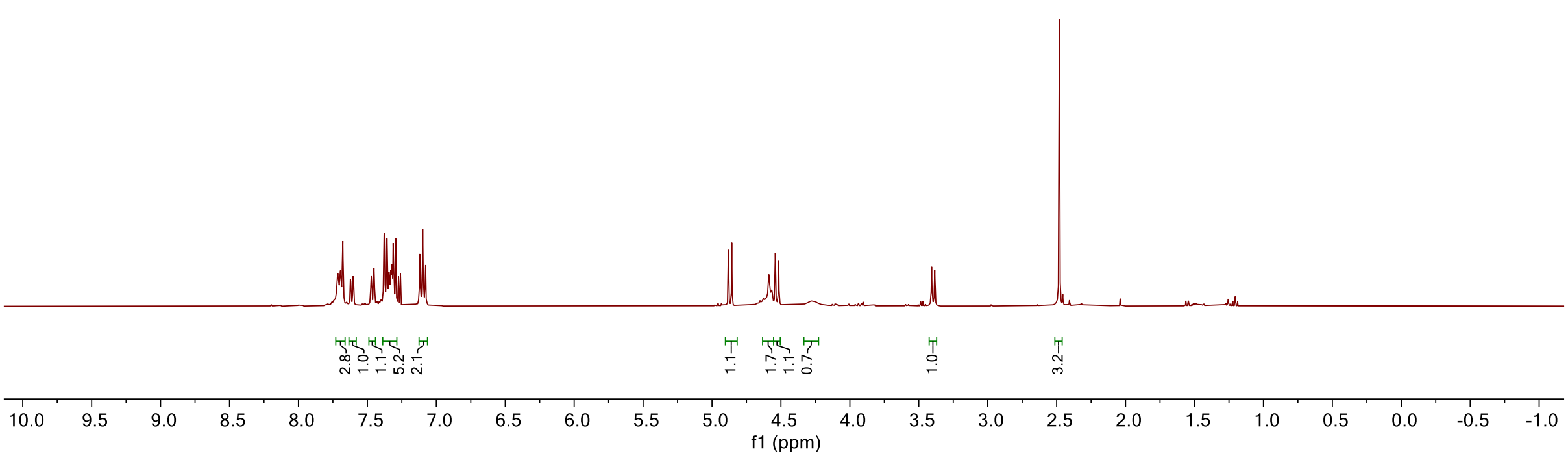

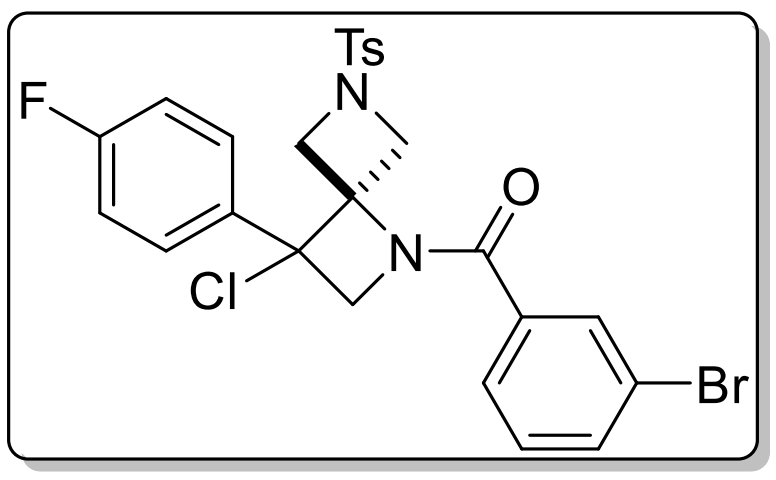

**74**

$^{19}F$ NMR (377 MHz, $CDCl_3$)

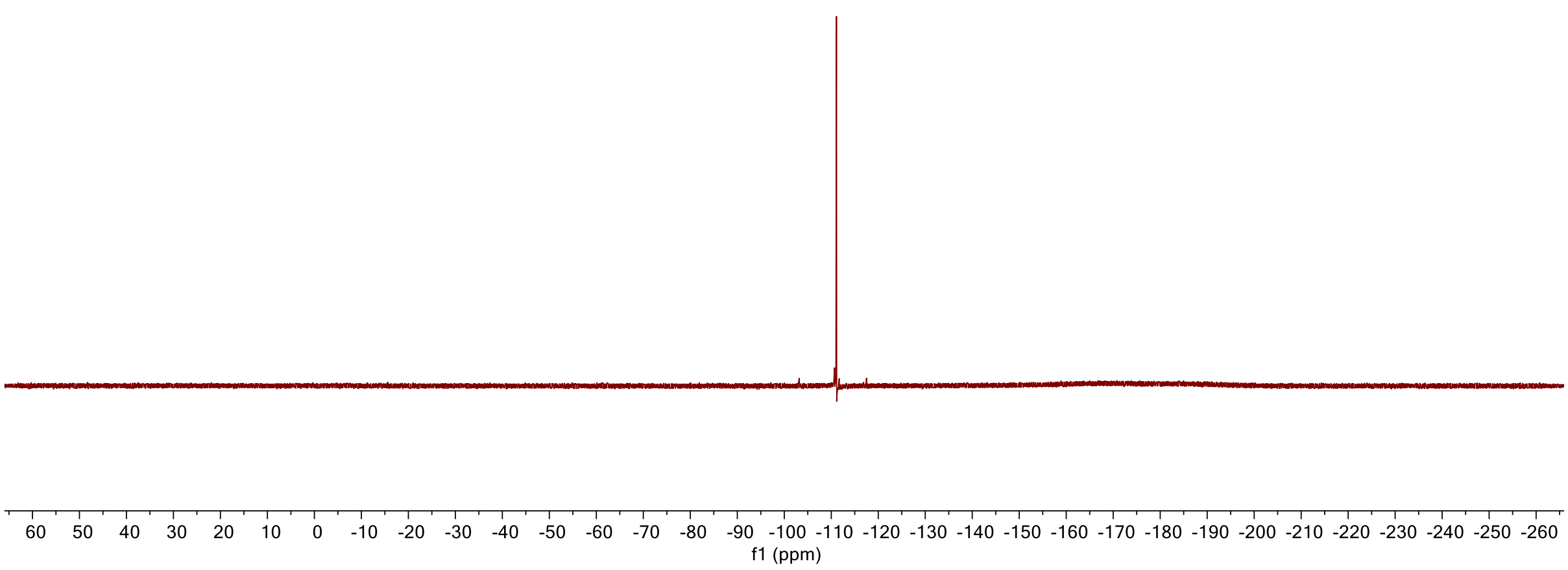

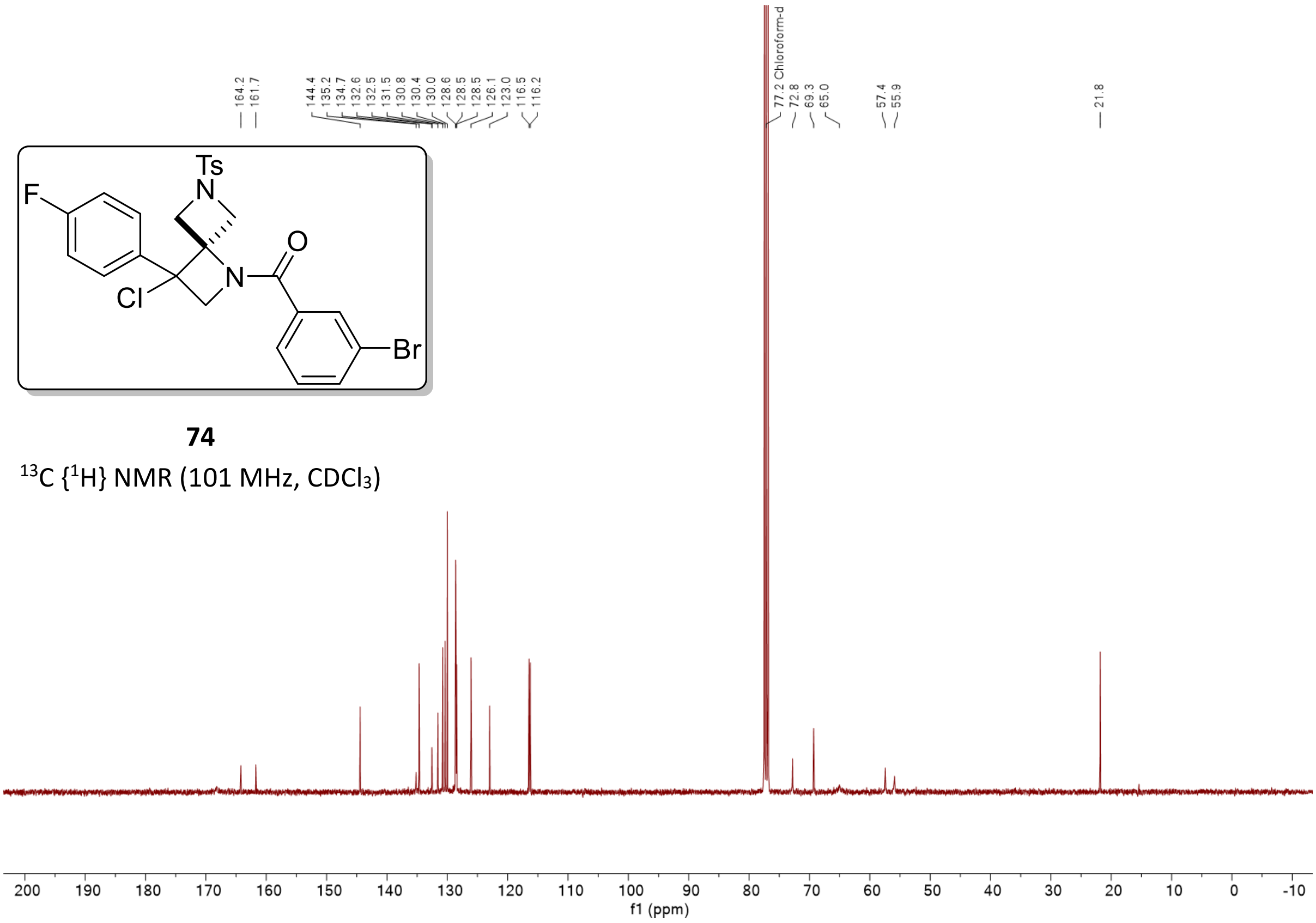

**74**

$^{13}C\{^1H\}$ NMR (101 MHz, $CDCl_3$)

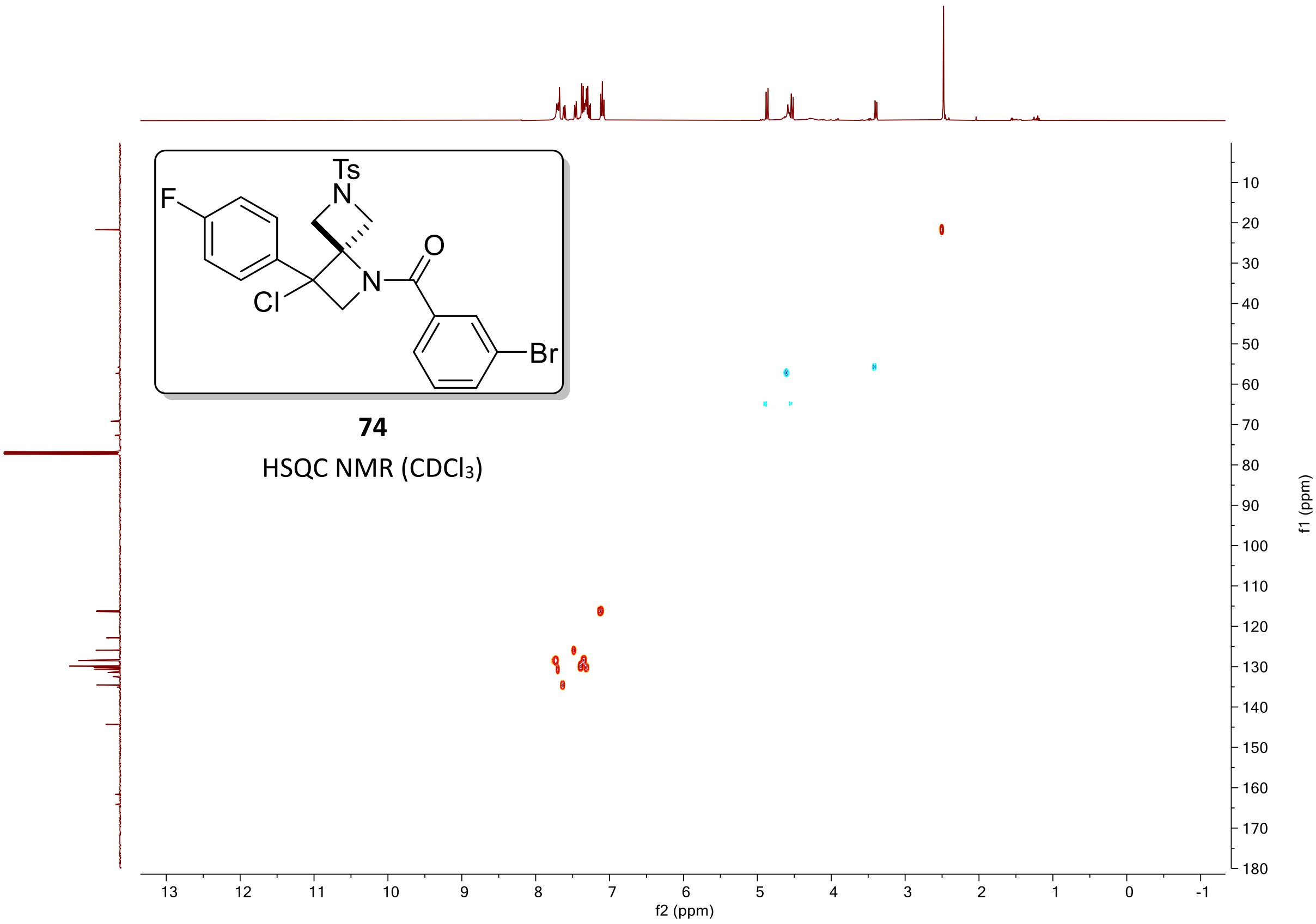

**74**

HSQC NMR ($CDCl_3$)

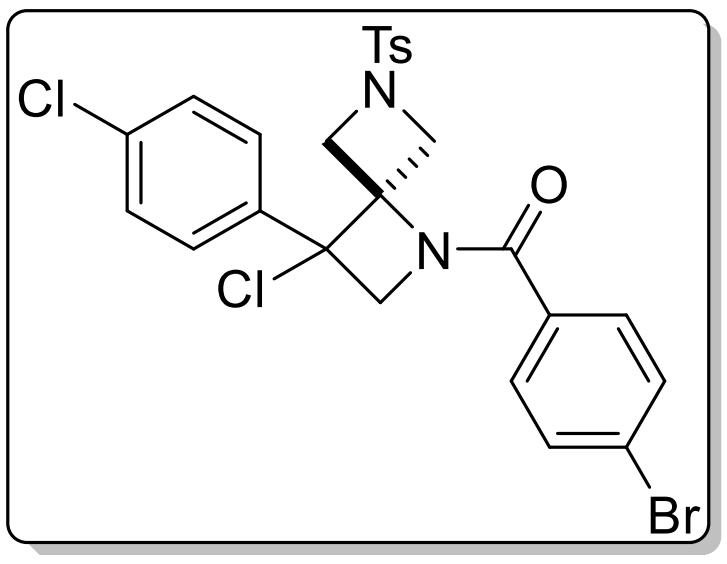


**75**

$^{1}$H NMR (400 MHz, $CDCl_3$)

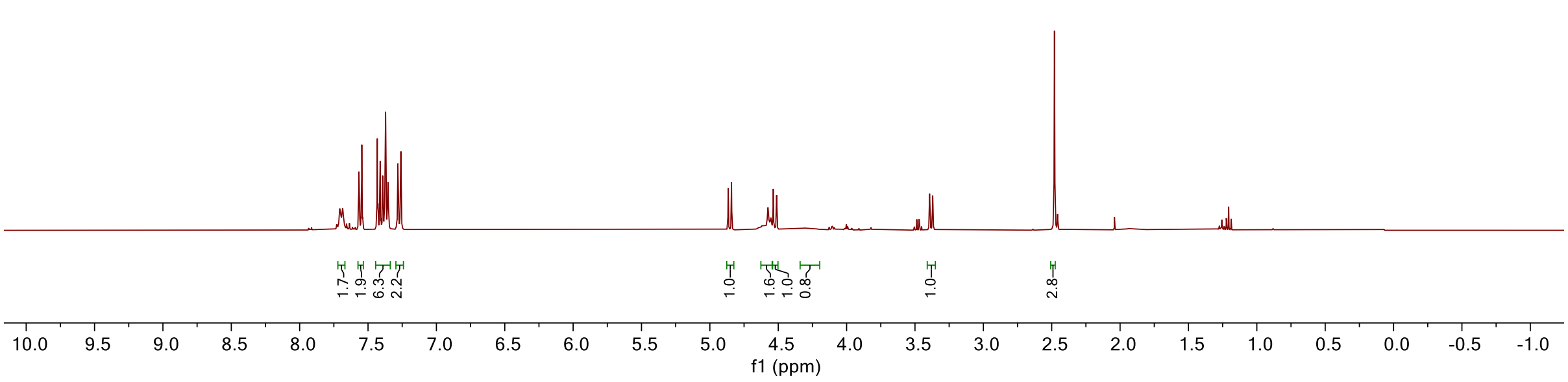


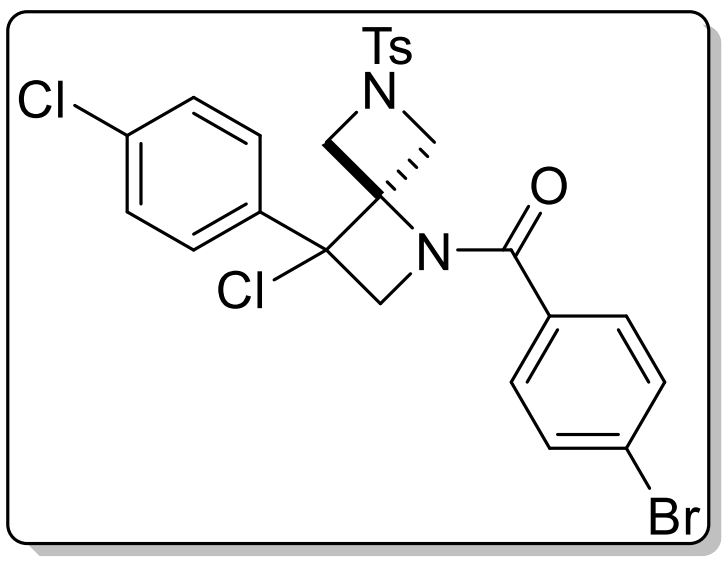


**75**

$^{13}$C {$^{1}$H} NMR (101 MHz, $CDCl_3$)

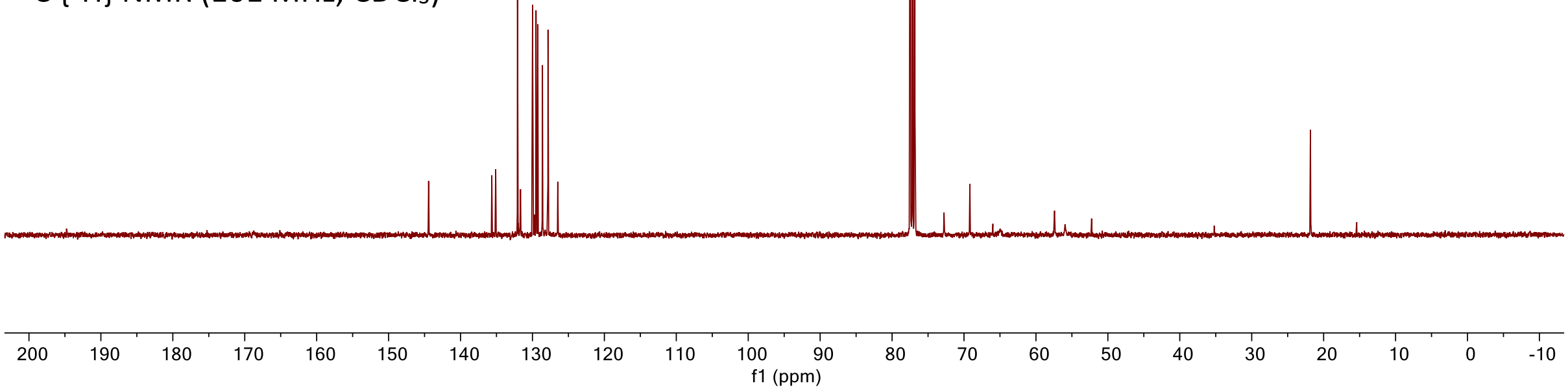

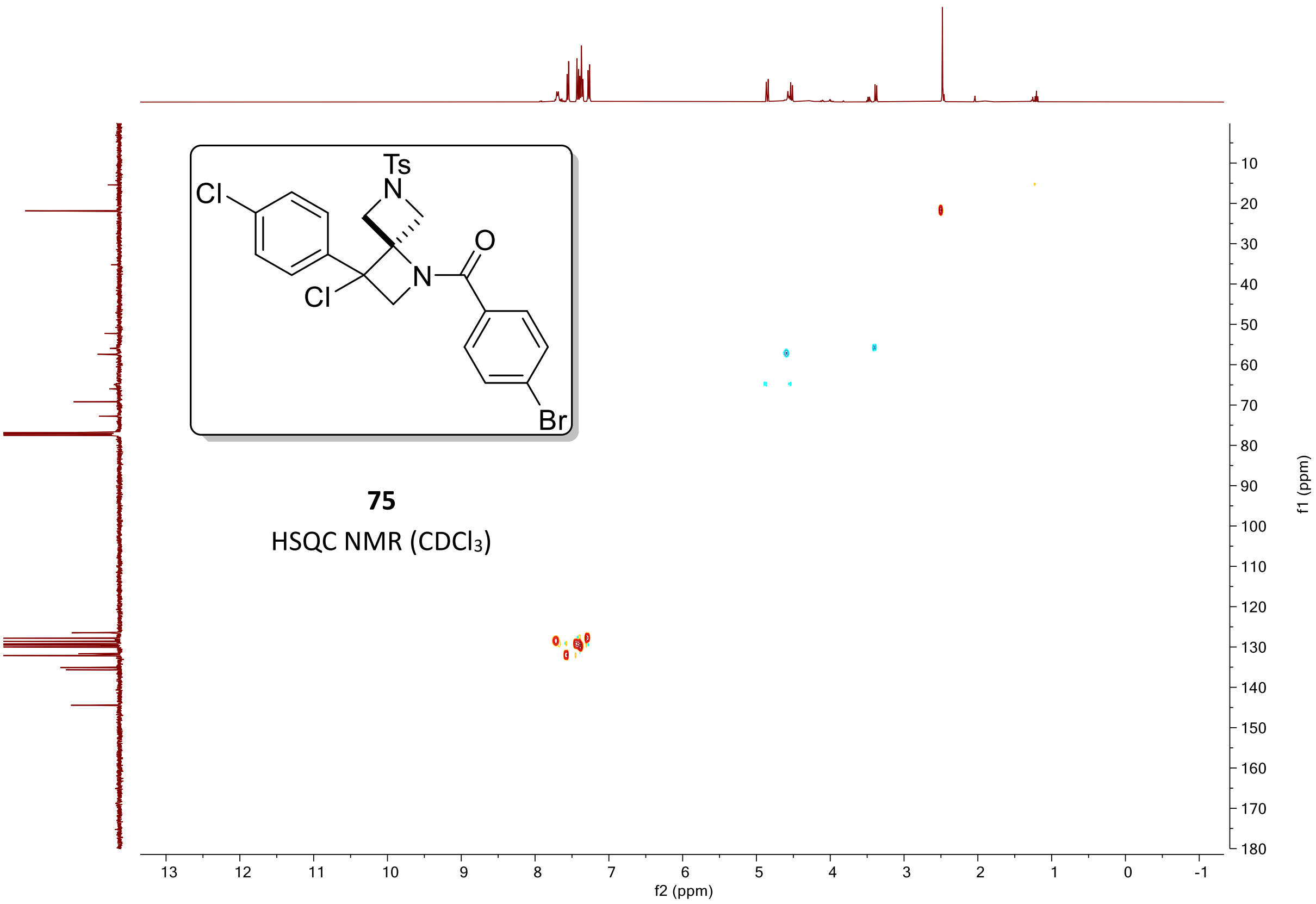


**75**

HSQC NMR ($CDCl_3$)

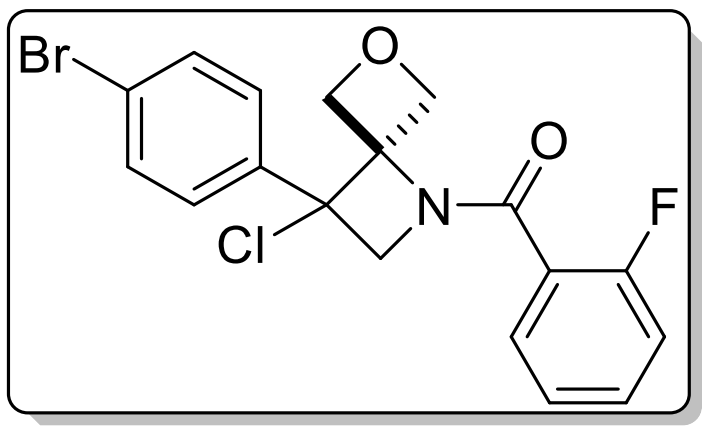

**76**

$^1$H NMR (400 MHz, $CDCl_3$)

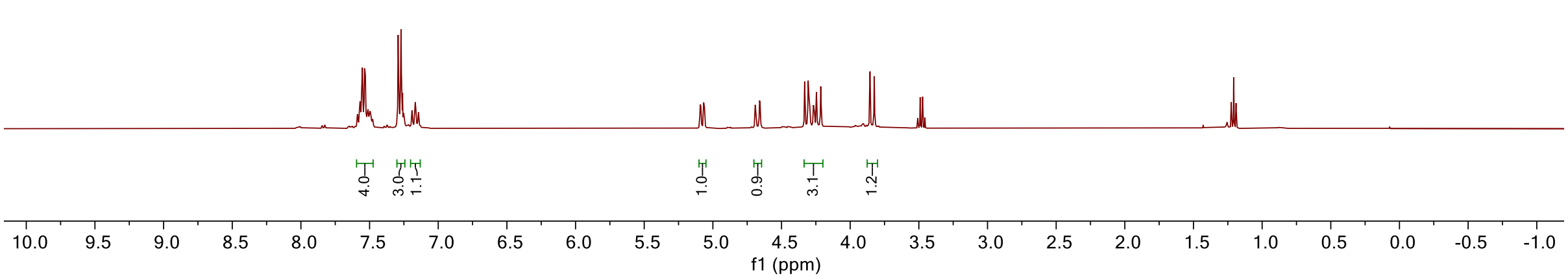

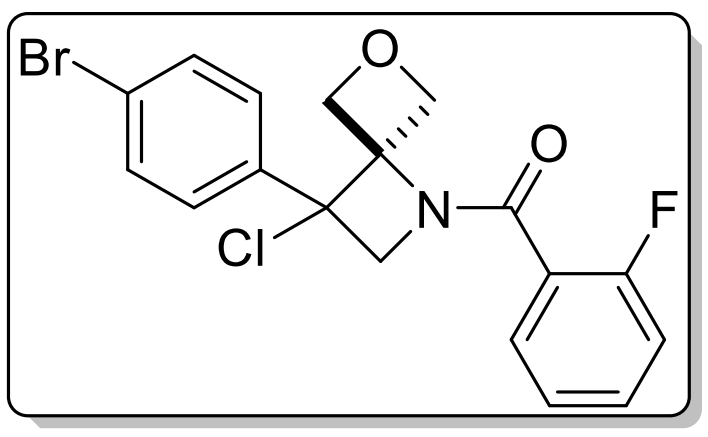

**76**

$^{19}$F NMR (377 MHz, $CDCl_3$)

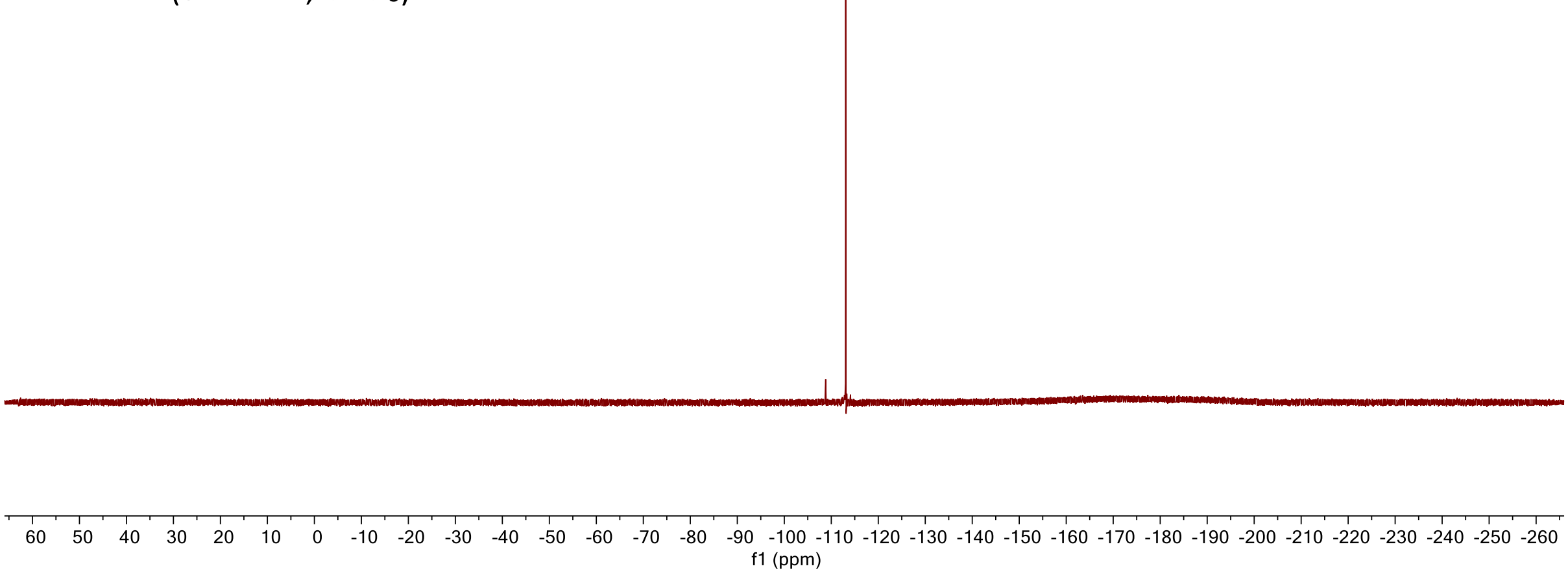

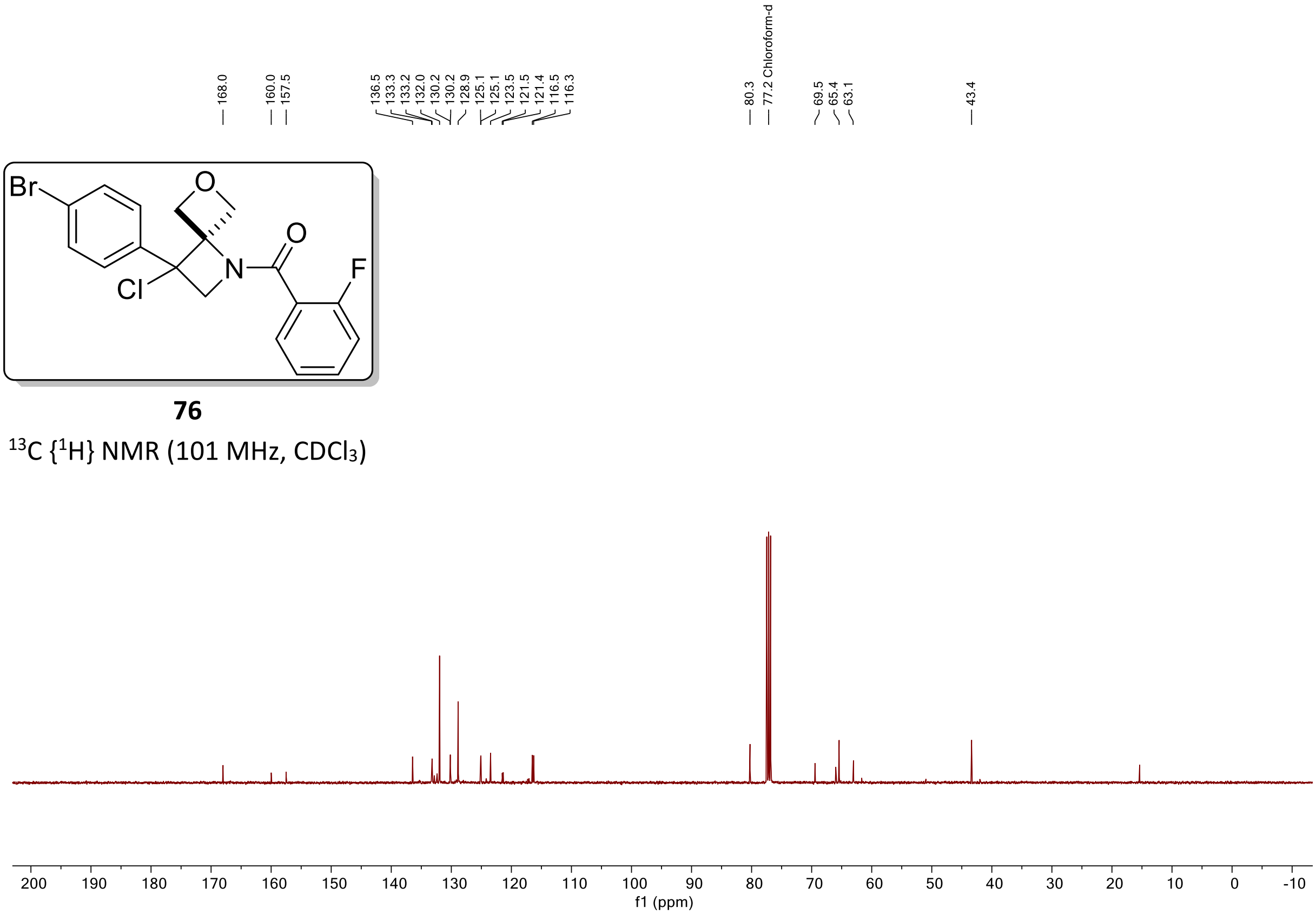


**76**

$^{13}C\ \{^{1}H\}$ NMR (101 MHz, $CDCl_3$)

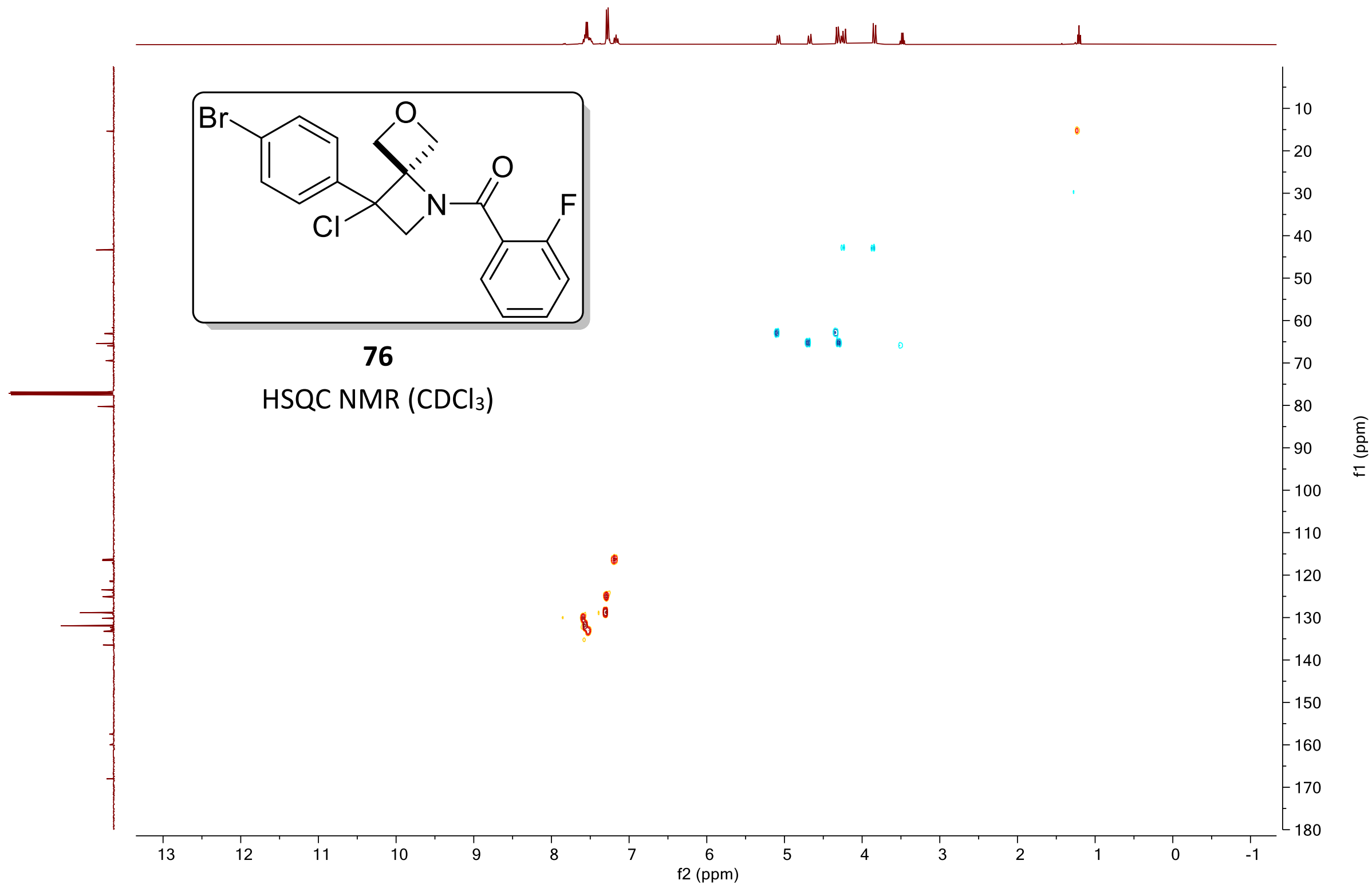


**76**

HSQC NMR ($CDCl_3$)

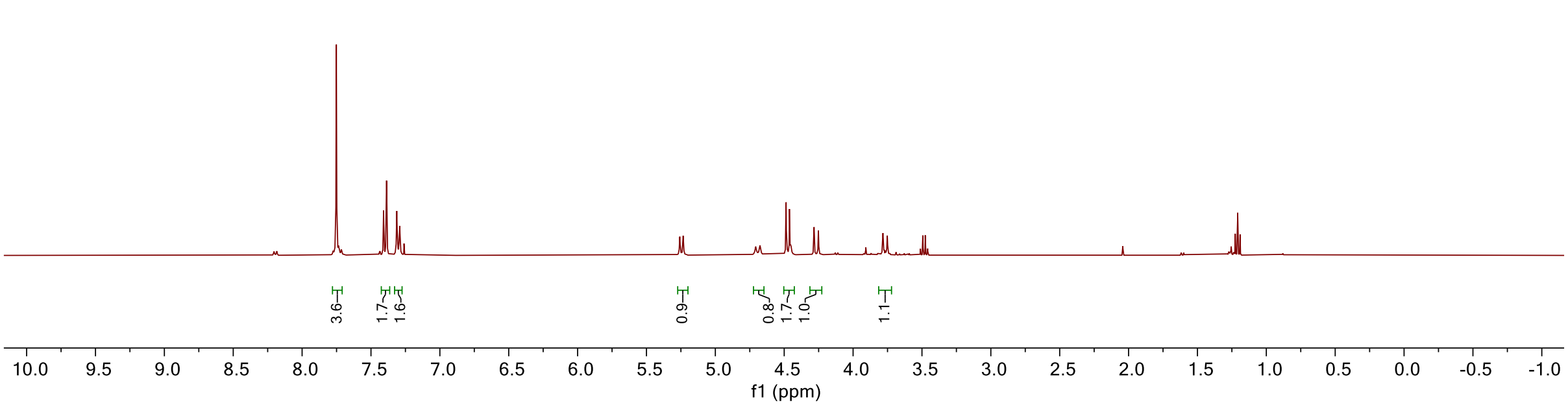


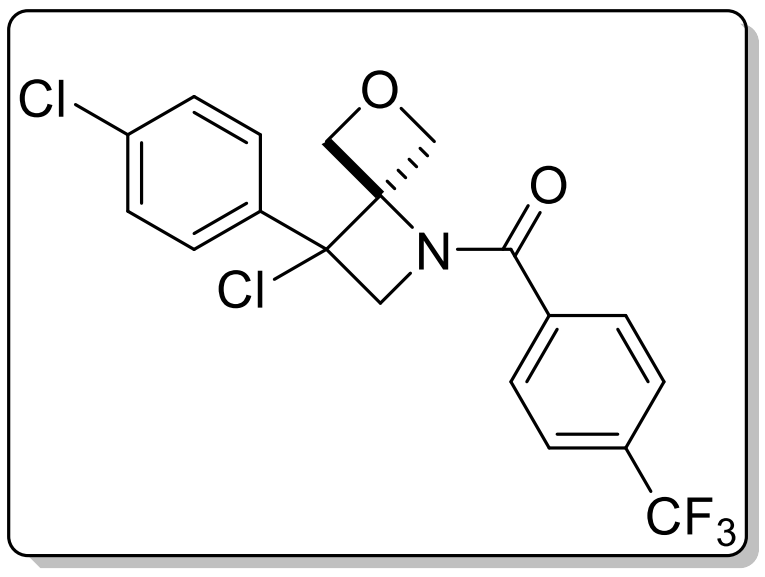


**77**

$^{1}$H NMR (400 MHz, $CDCl_3$)

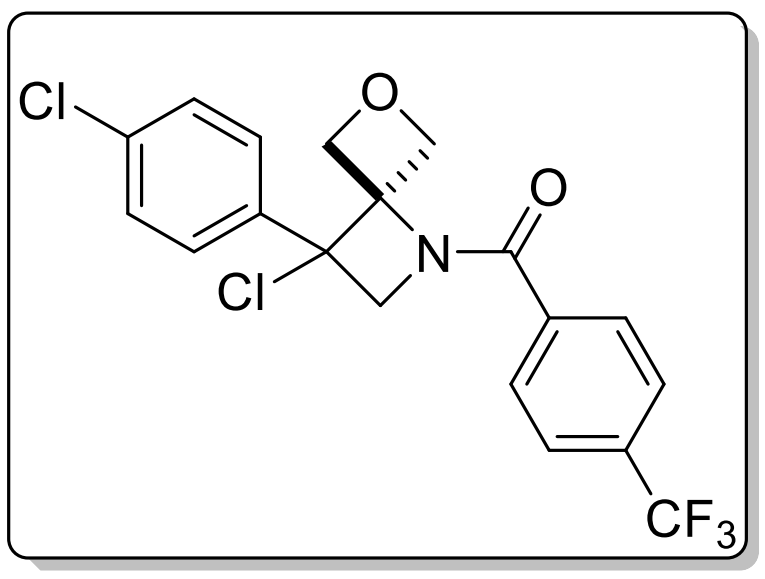


**77**

$^{19}$F NMR (377 MHz, $CDCl_3$)

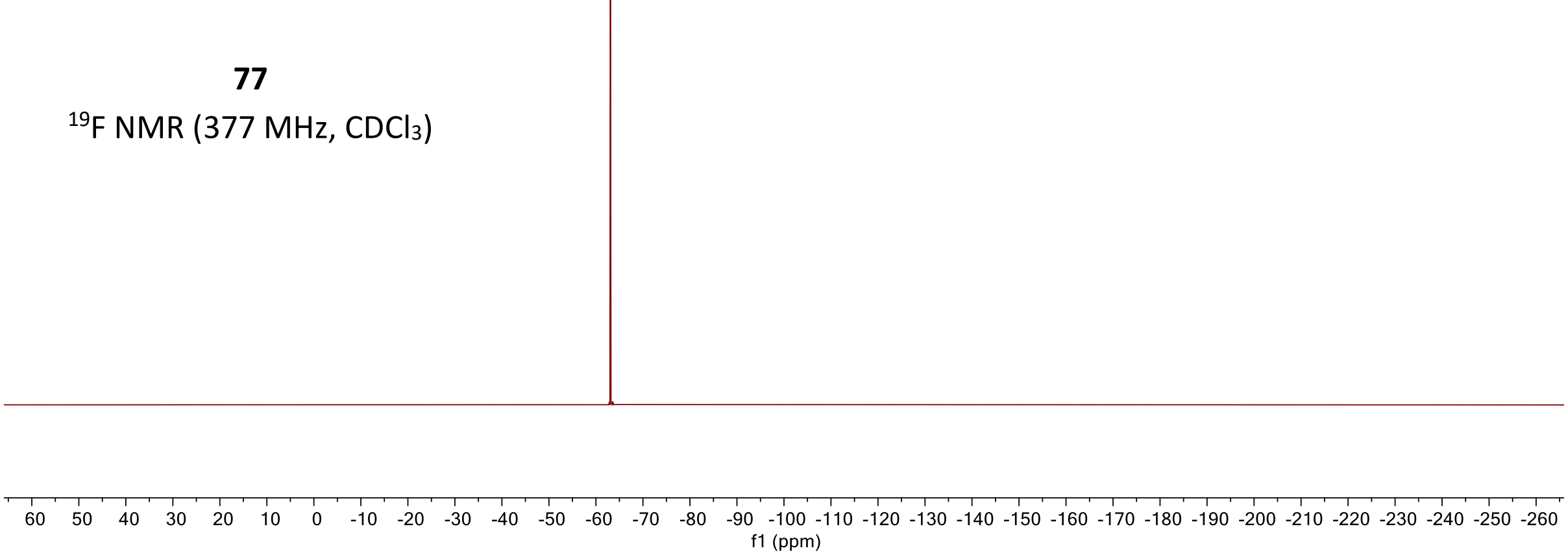

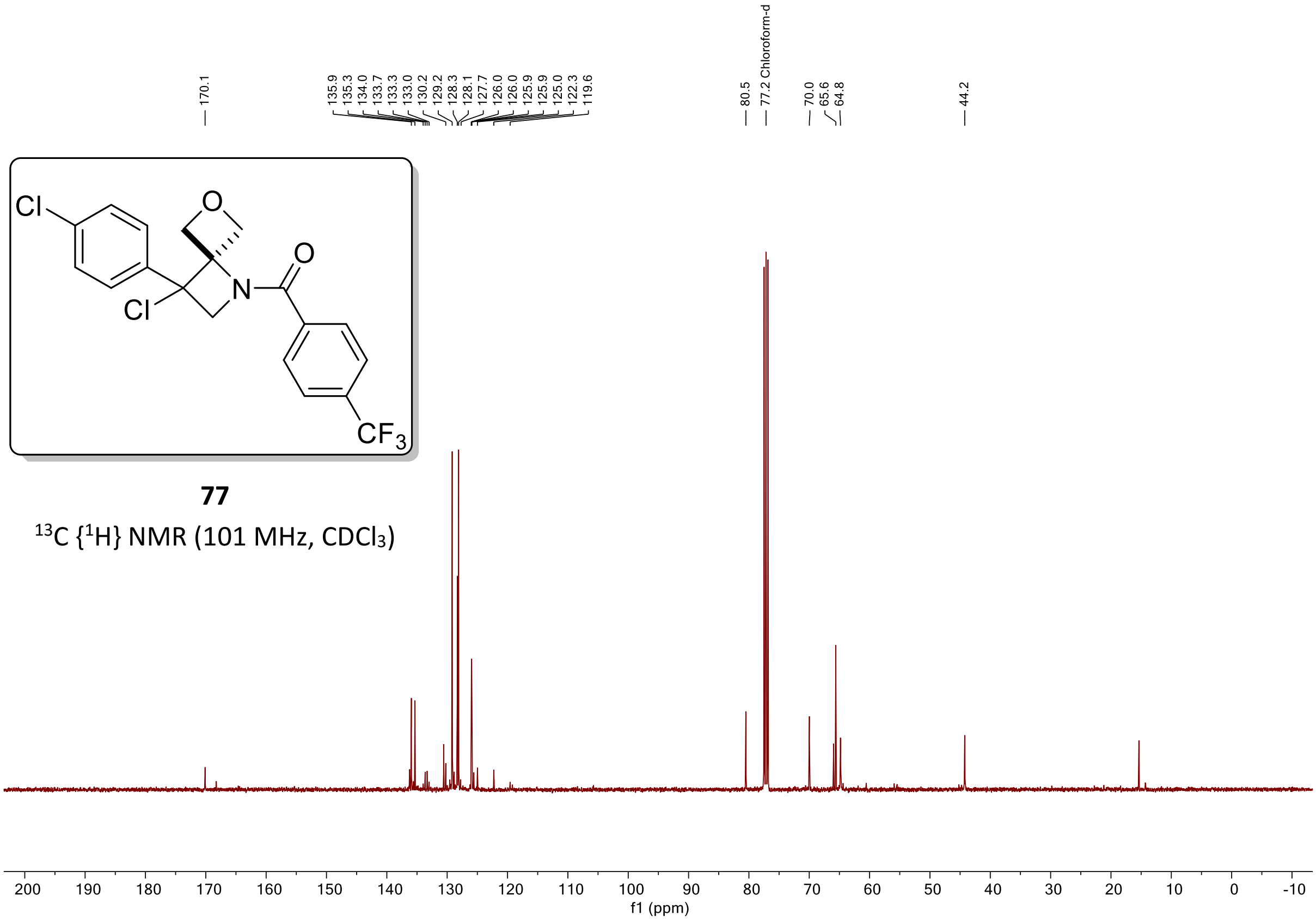


**77**

$^{13}C\ \{^{1}H\}$ NMR (101 MHz, $CDCl_3$)

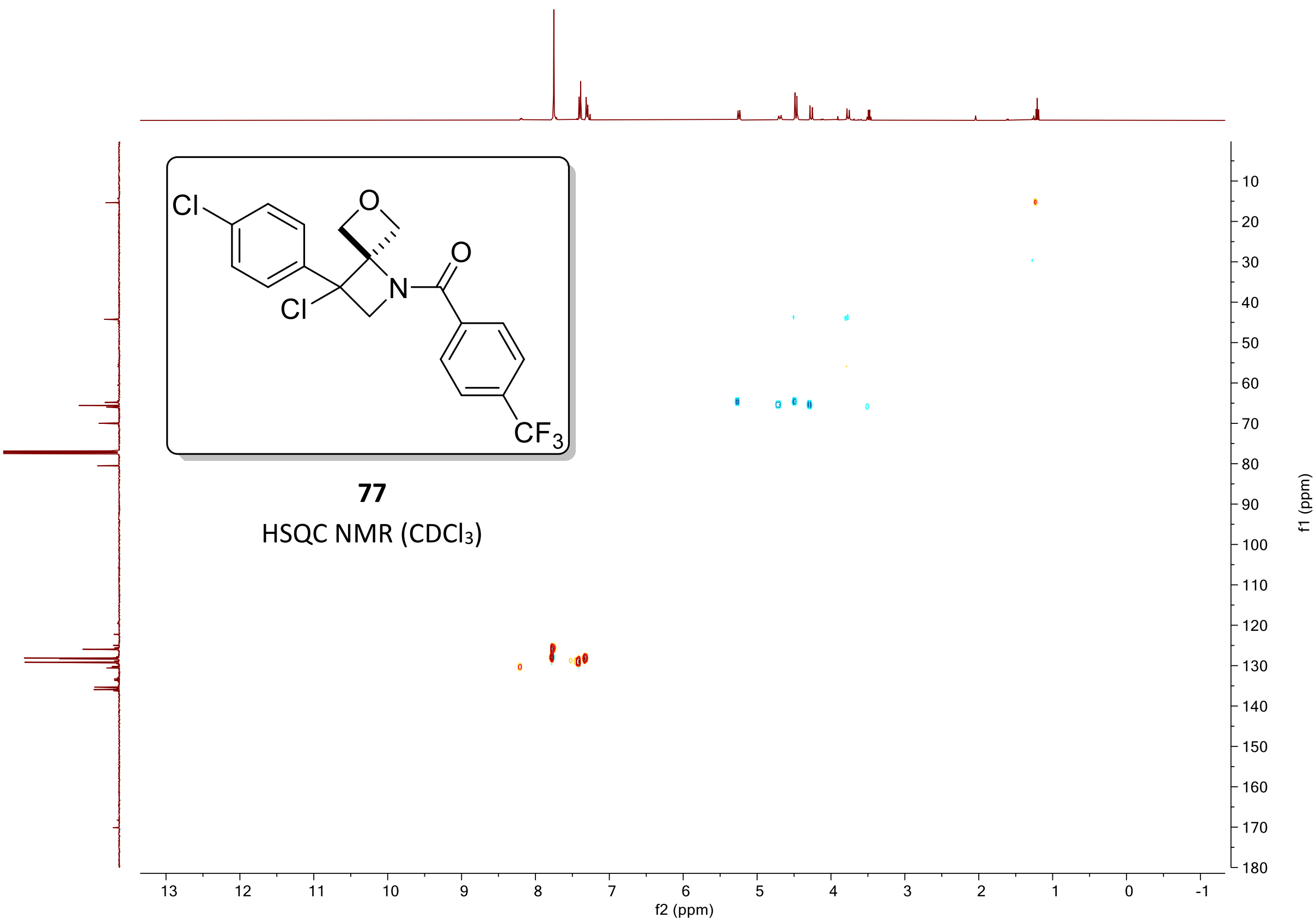


**77**

HSQC NMR ($CDCl_3$)

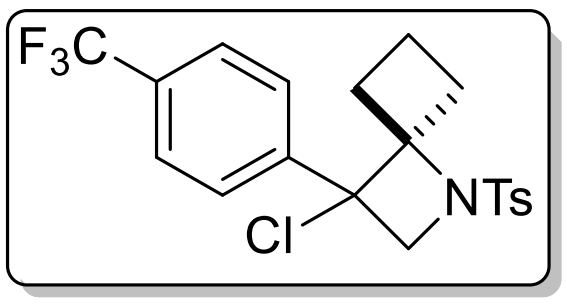


**78**

$^{1}H$ NMR (400 MHz, $CDCl_3$)

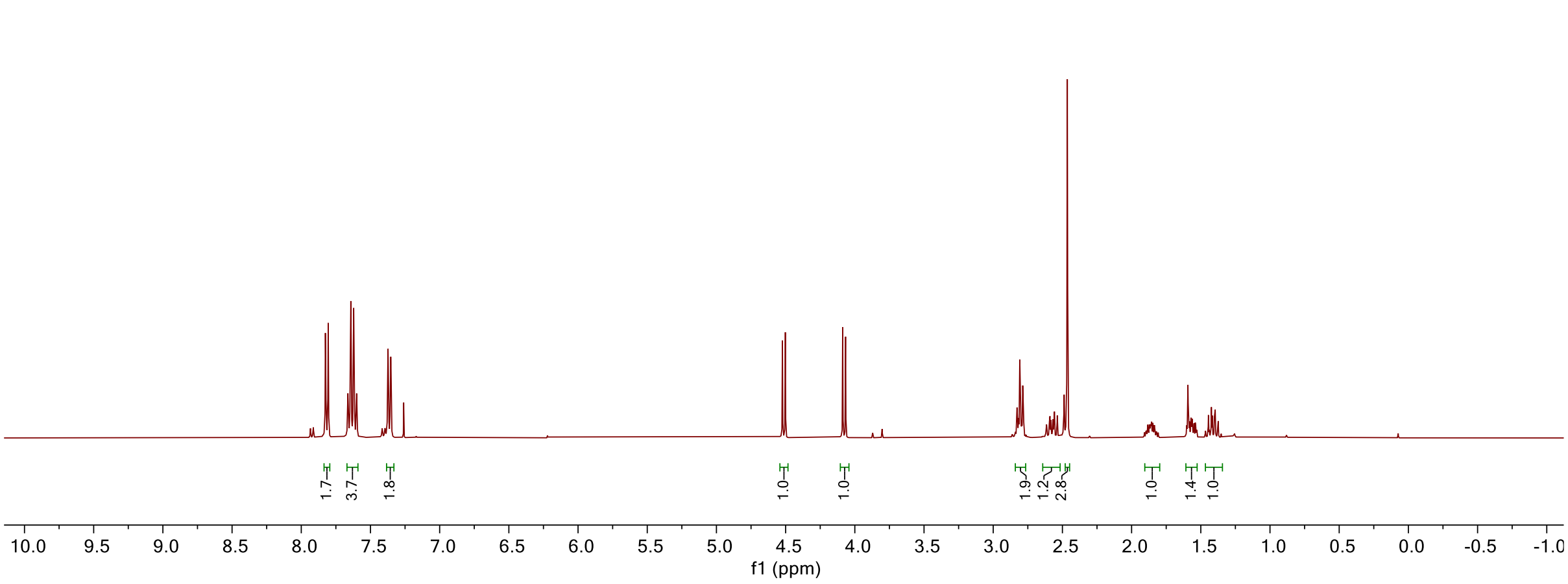


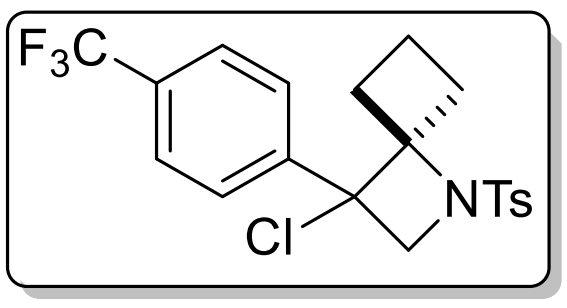


**78**

$^{19}F$ NMR (377 MHz, $CDCl_3$)

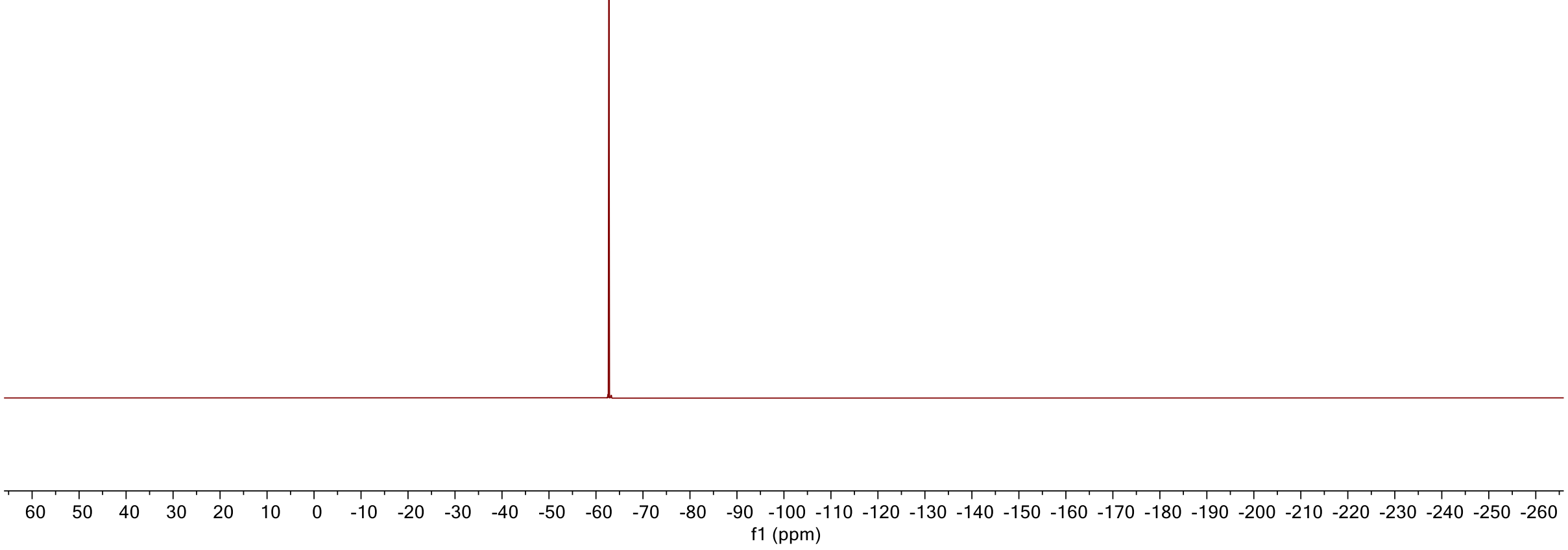

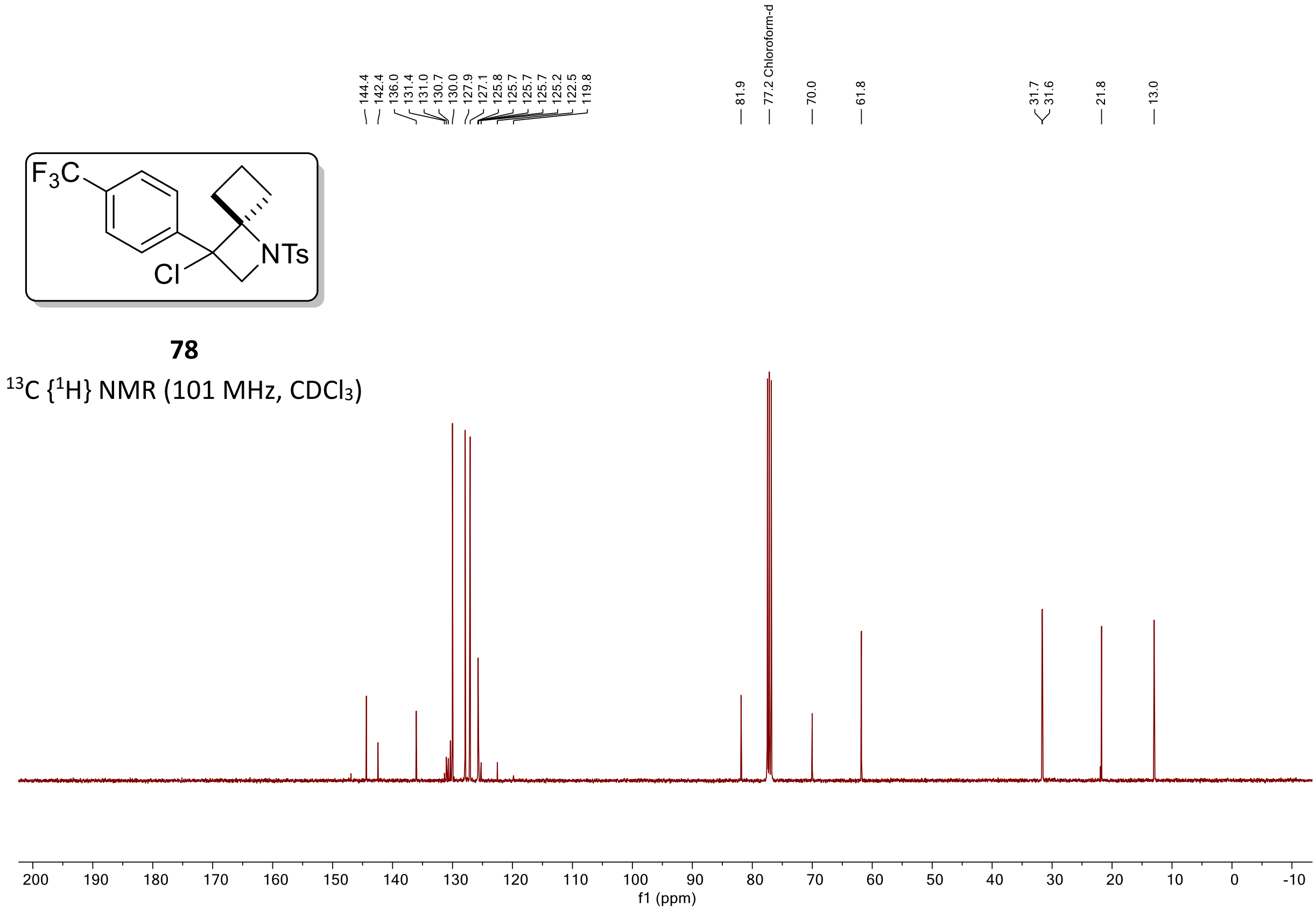


**78**

$^{13}$C {$^{1}$H} NMR (101 MHz, $CDCl_3$)

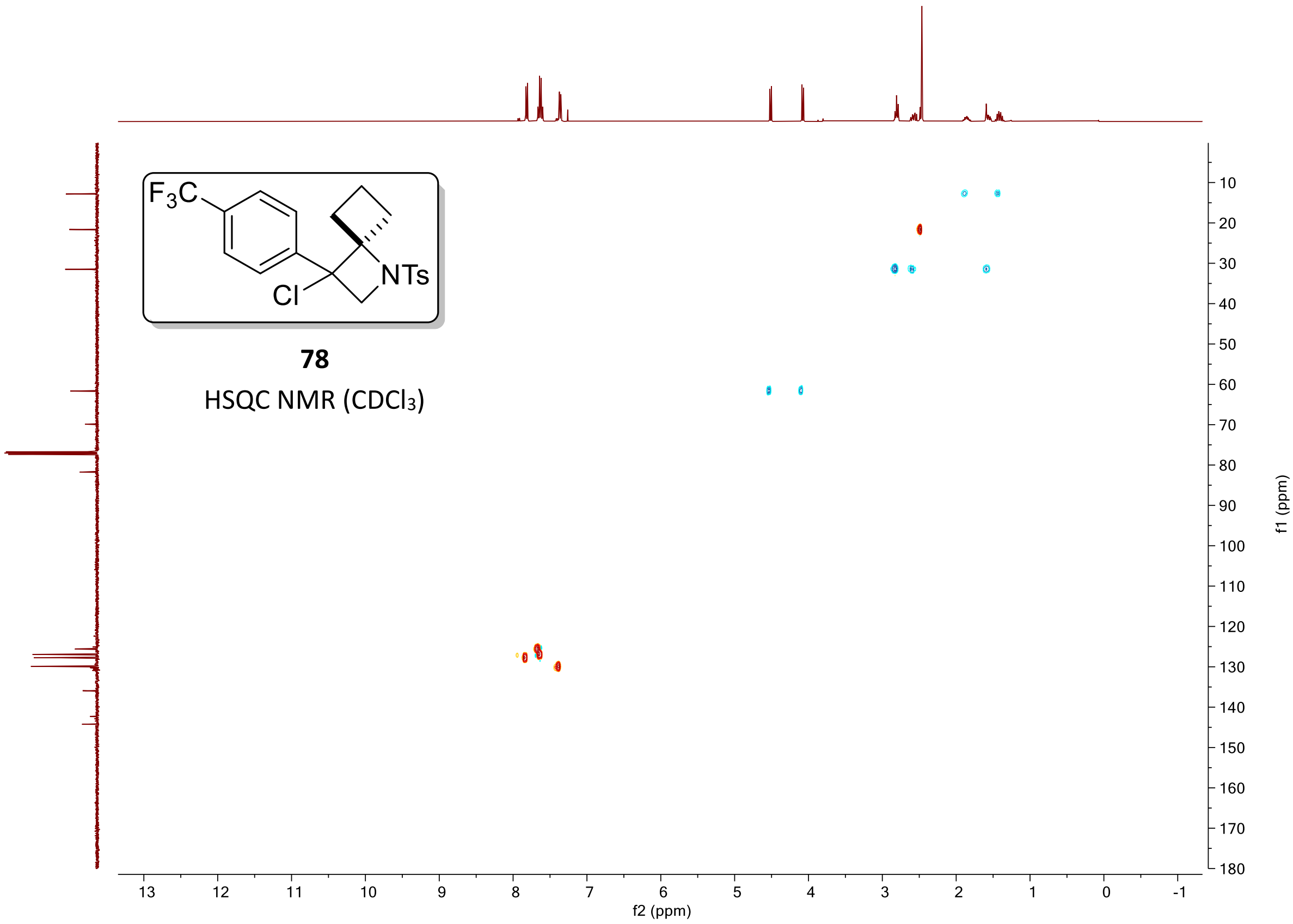


**78**

HSQC NMR ($CDCl_3$)

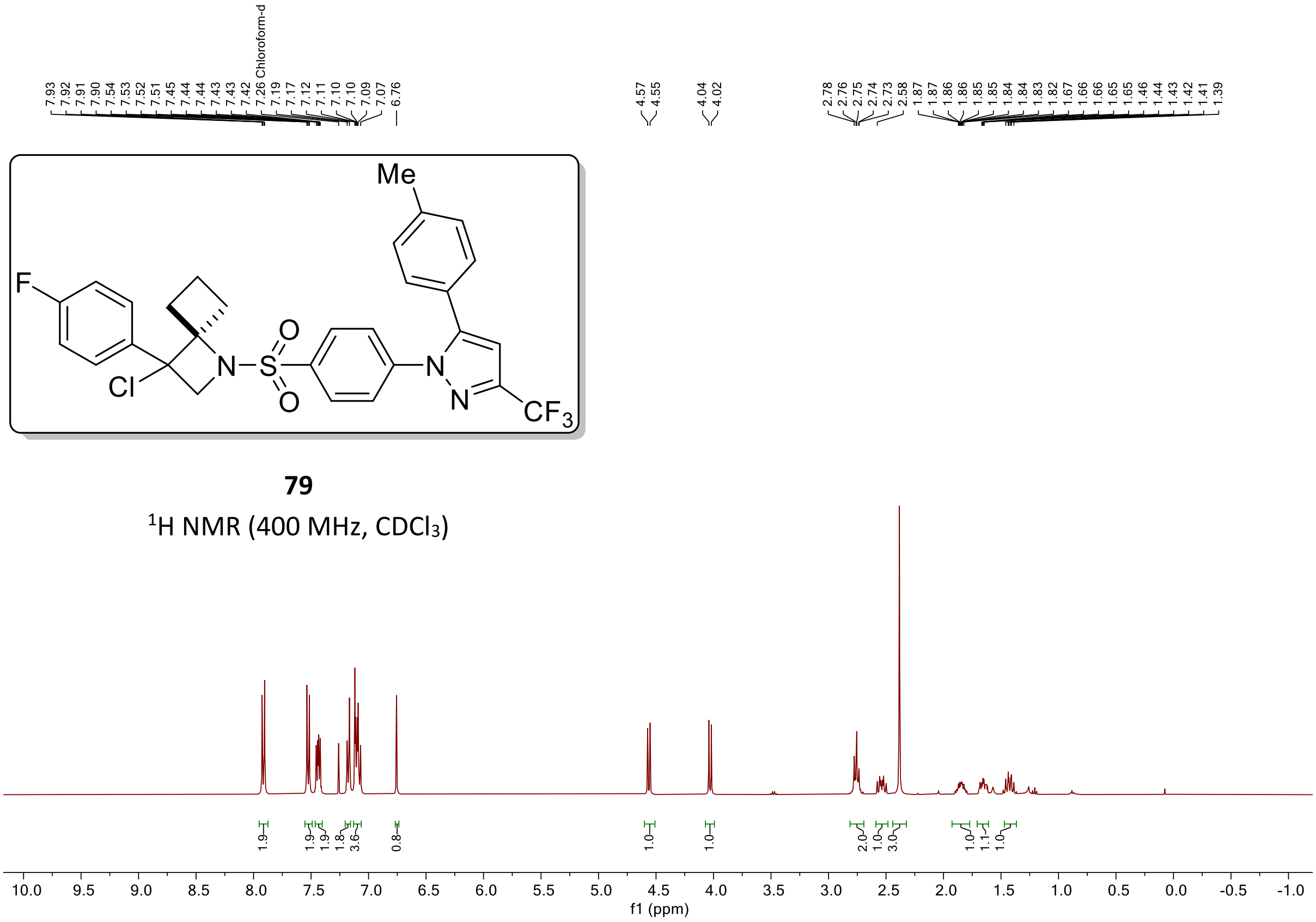

**79**

$^{1}$H NMR (400 MHz, $CDCl_3$)

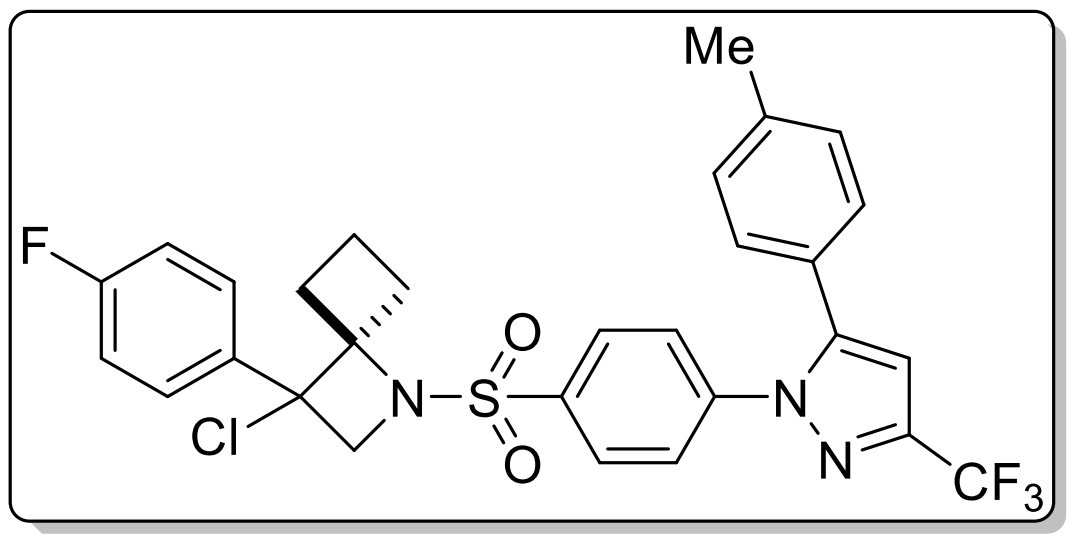

**79**

$^{19}$F NMR (377 MHz, $CDCl_3$)

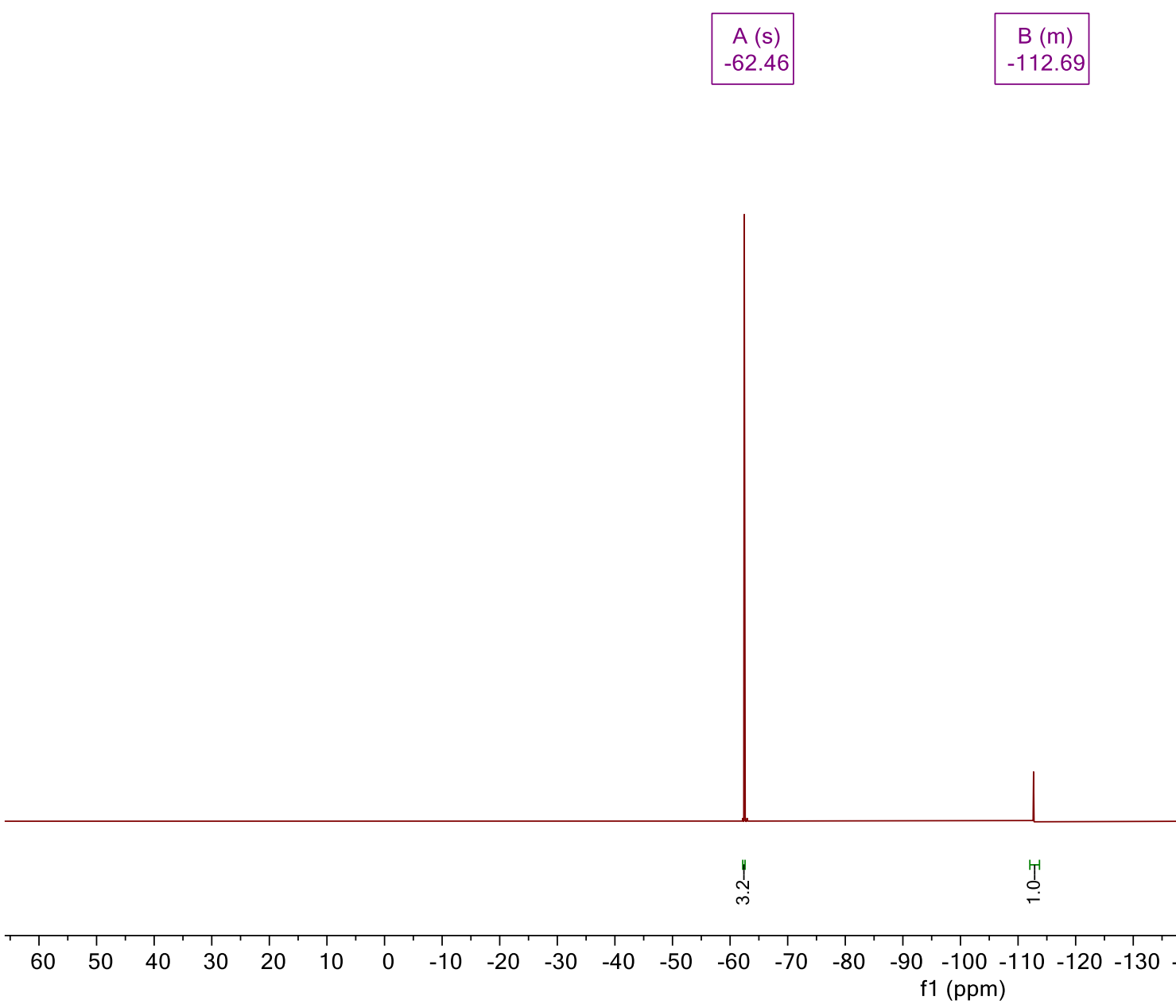

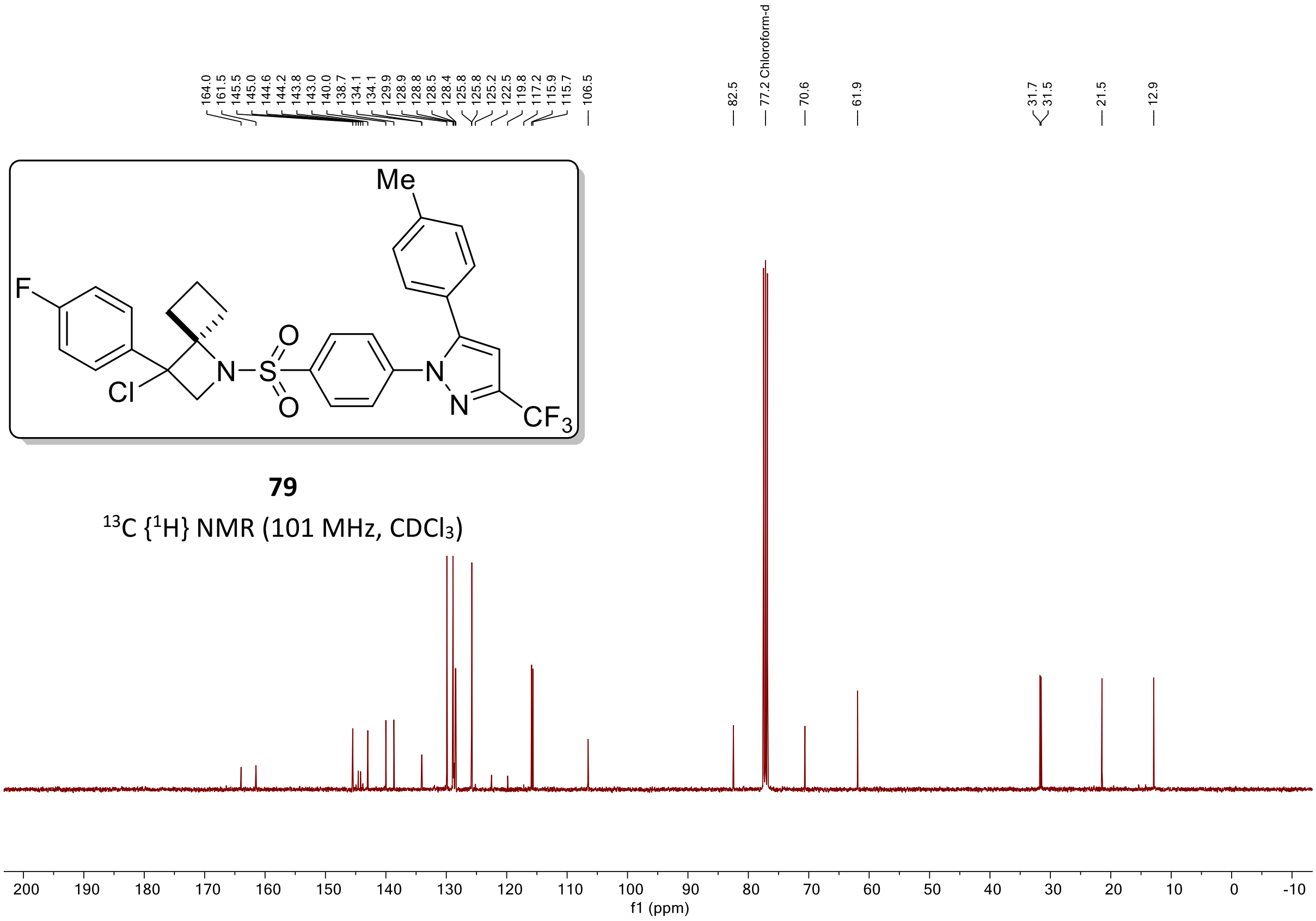


**79**

$^{13}C$ {$^{1}H$} NMR (101 MHz, $CDCl_3$)

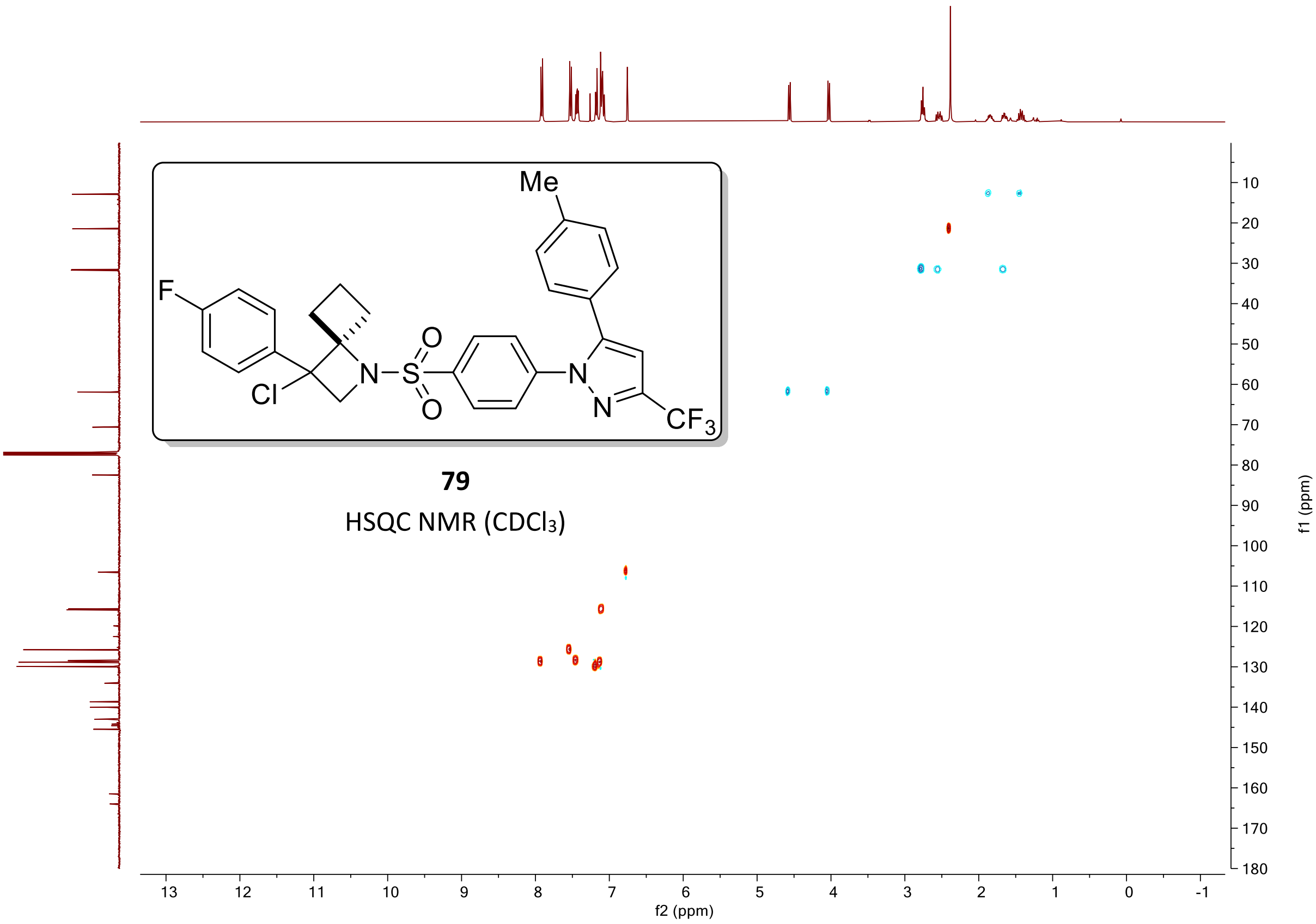


**79**

HSQC NMR ($CDCl_3$)

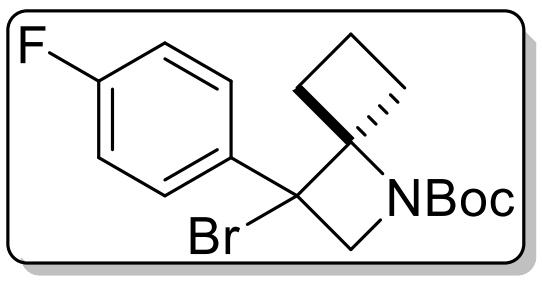

**80**

$^{1}$H NMR (400 MHz, CDCl$_3$)

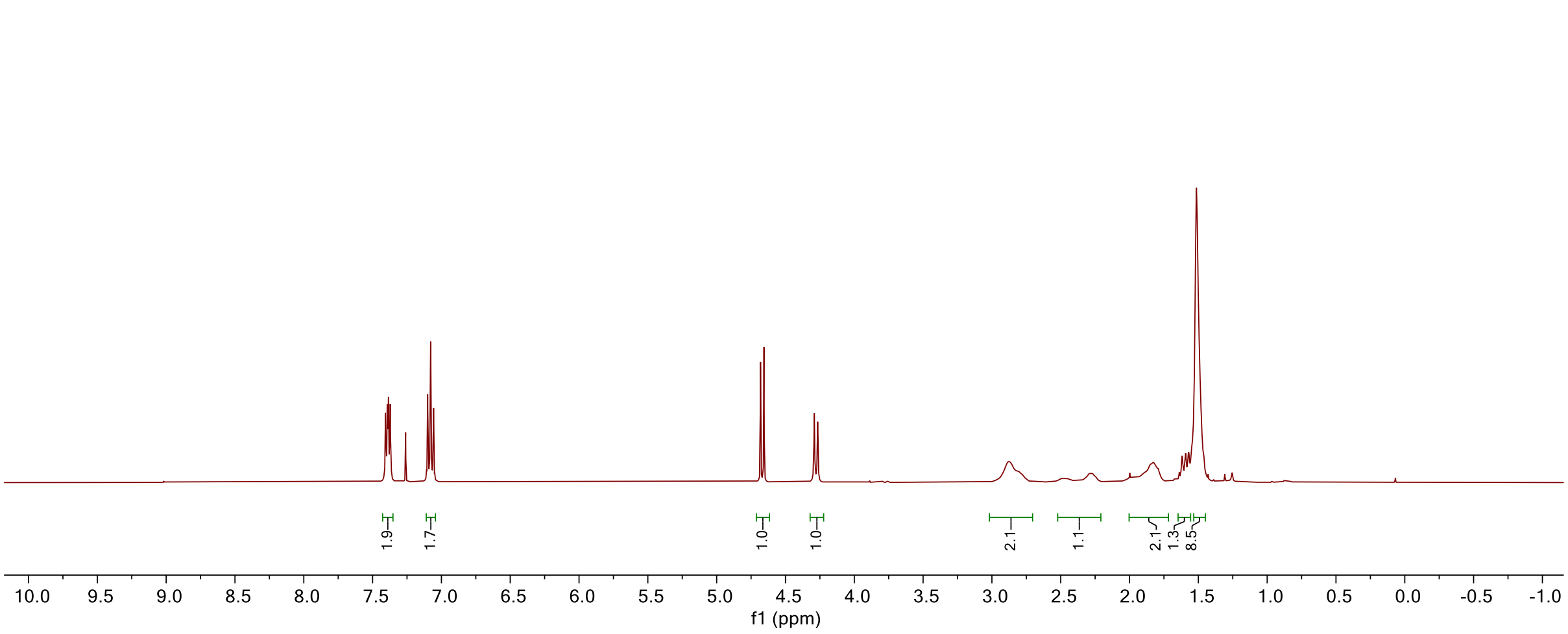


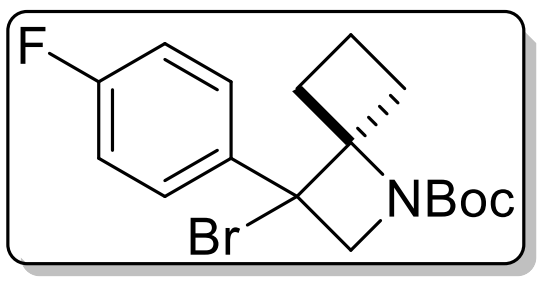

**80**

$^{19}$F NMR (377 MHz, CDCl$_3$)

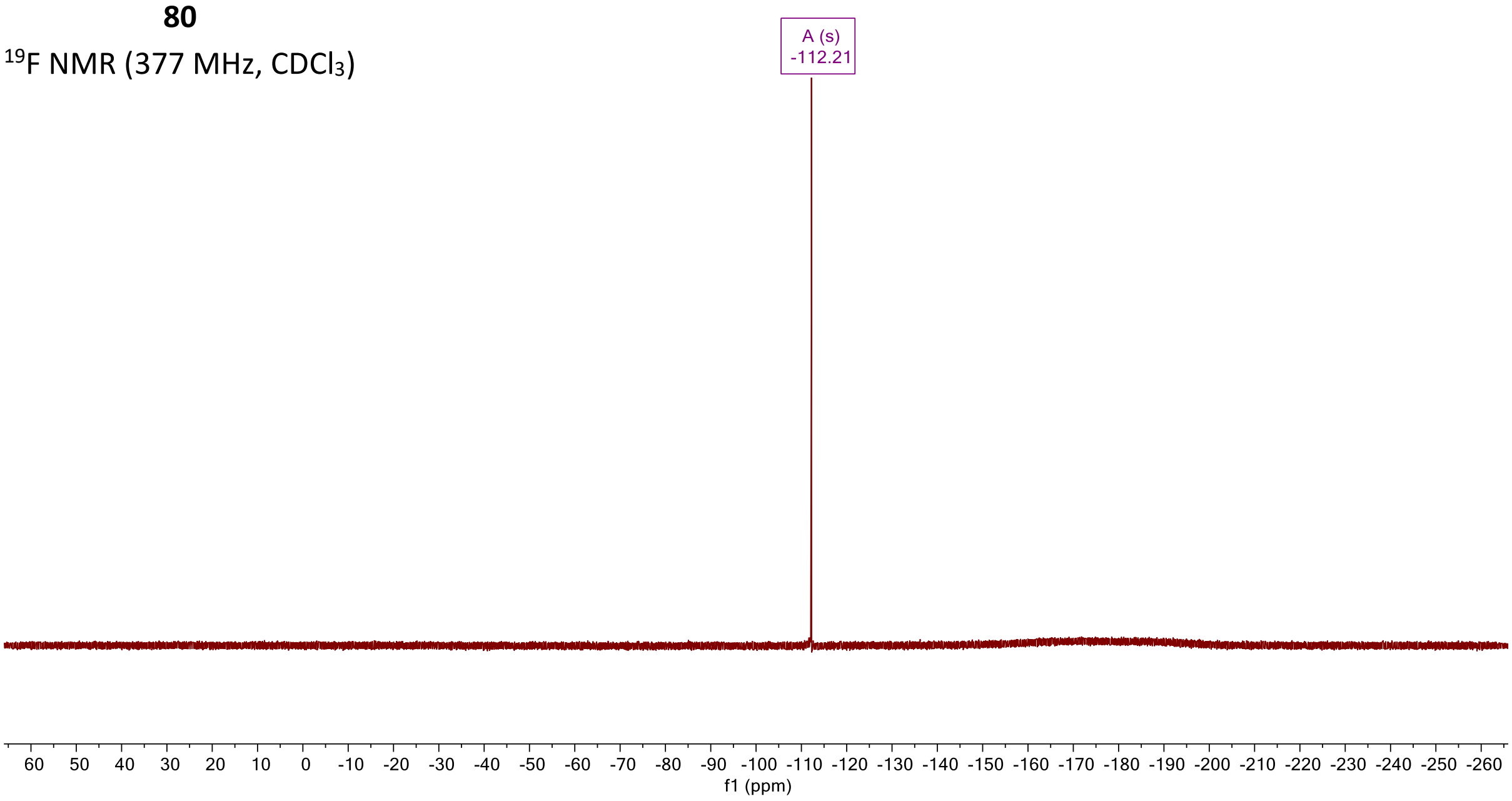

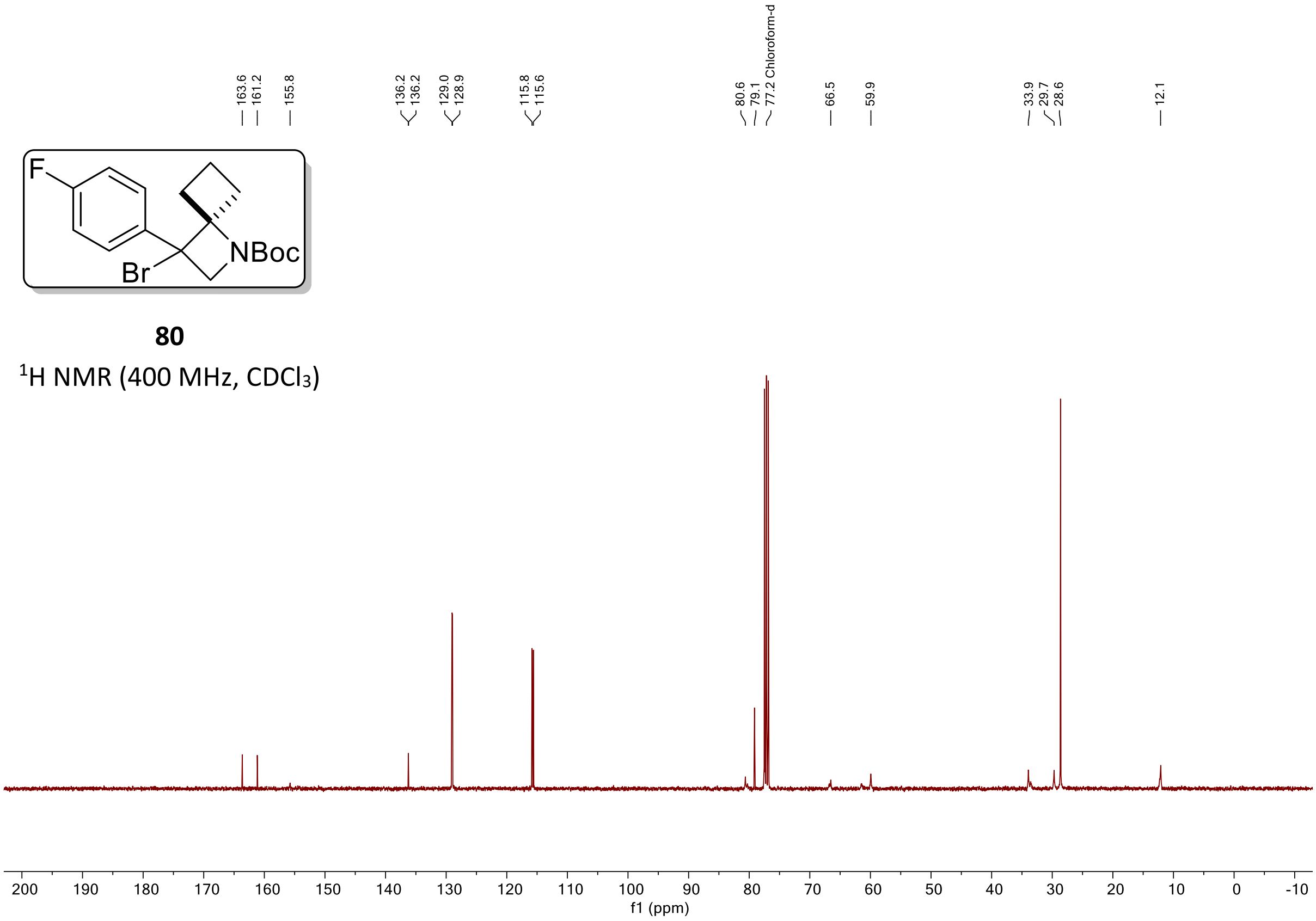


**80**

$^{1}$H NMR (400 MHz, $CDCl_3$)

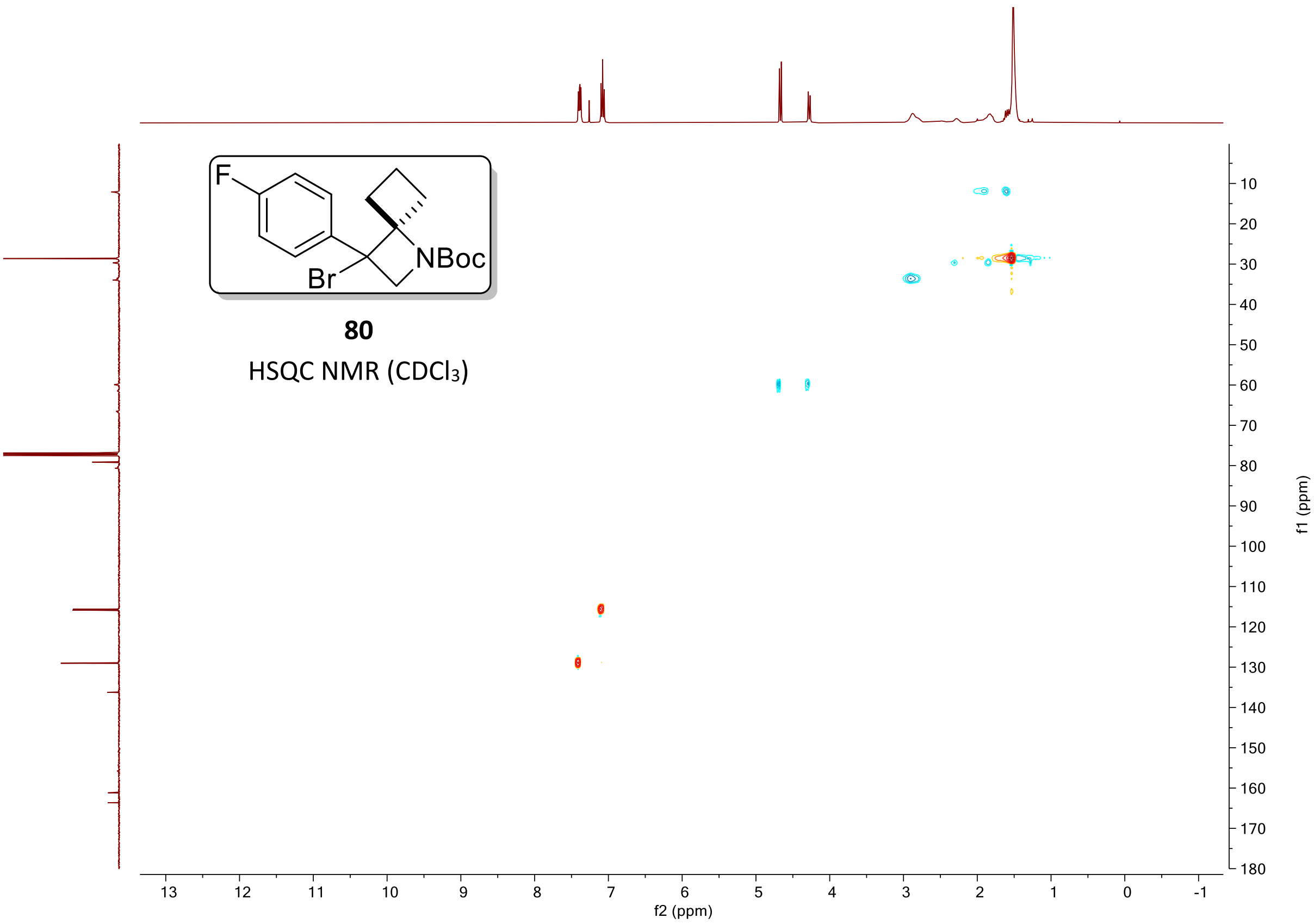


**80**

HSQC NMR ($CDCl_3$)

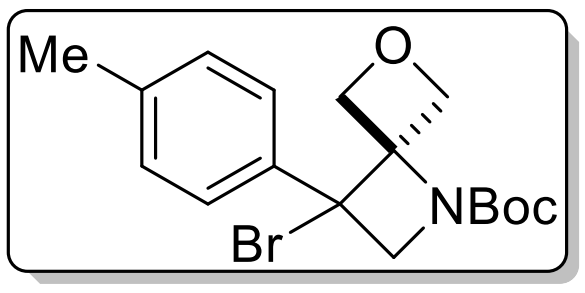


**81**

$^{1}$H NMR (400 MHz, $CDCl_3$)

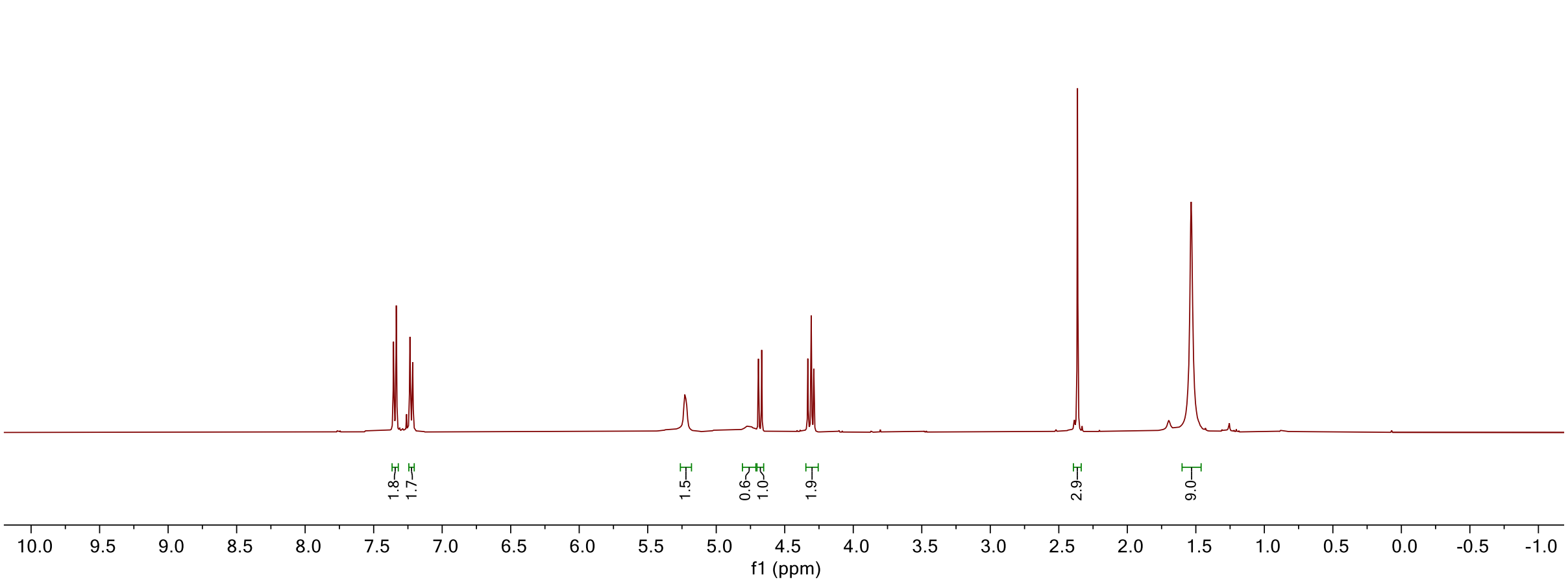


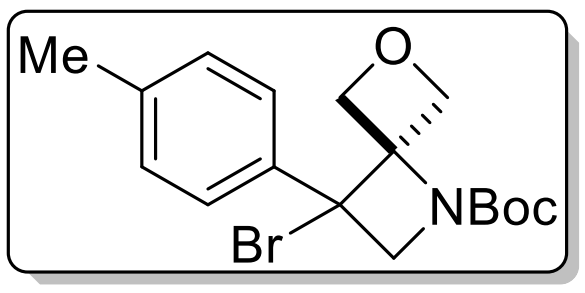


**81**

$^{13}$C {$^{1}$H} NMR (101 MHz, $CDCl_3$)

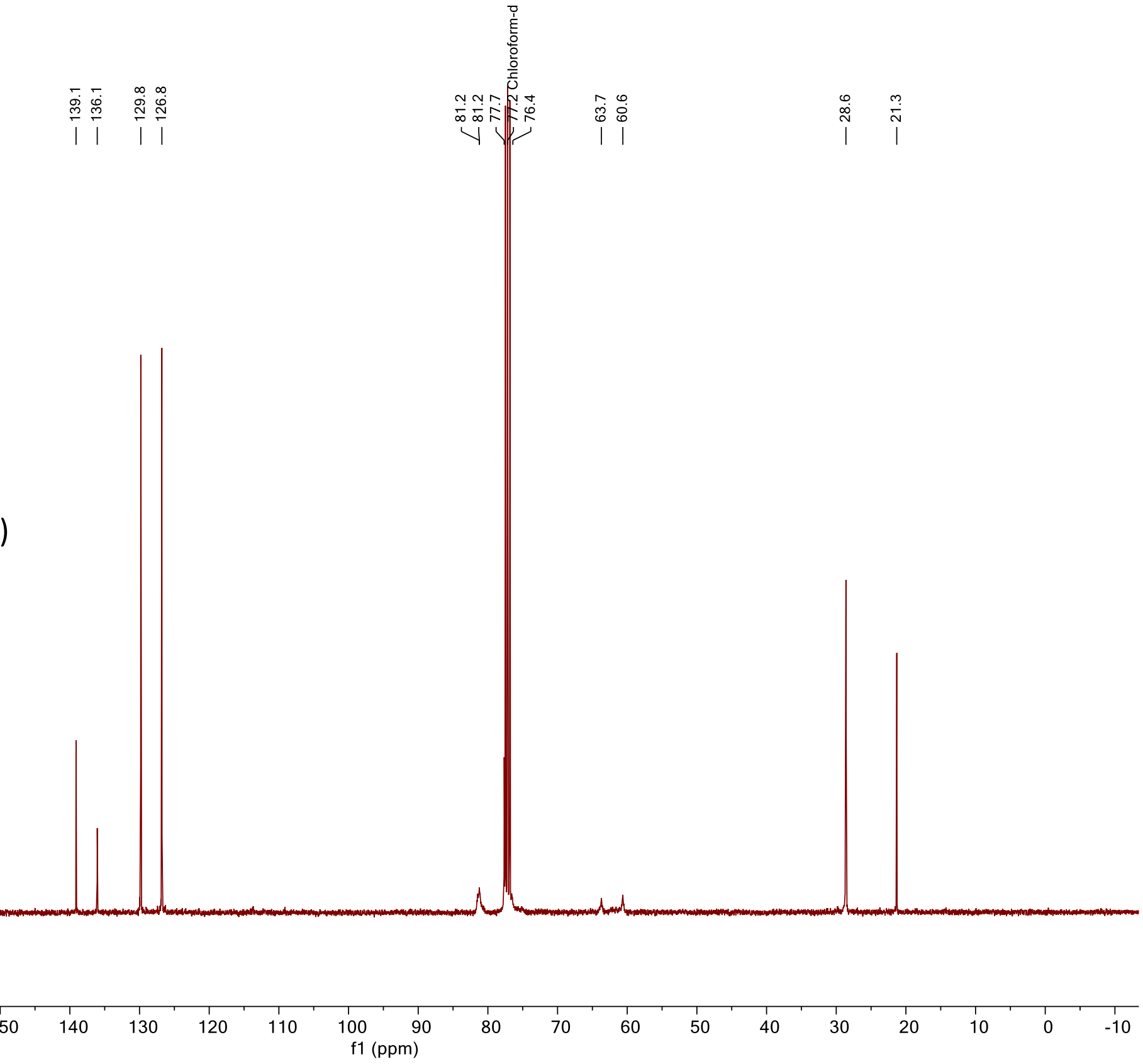

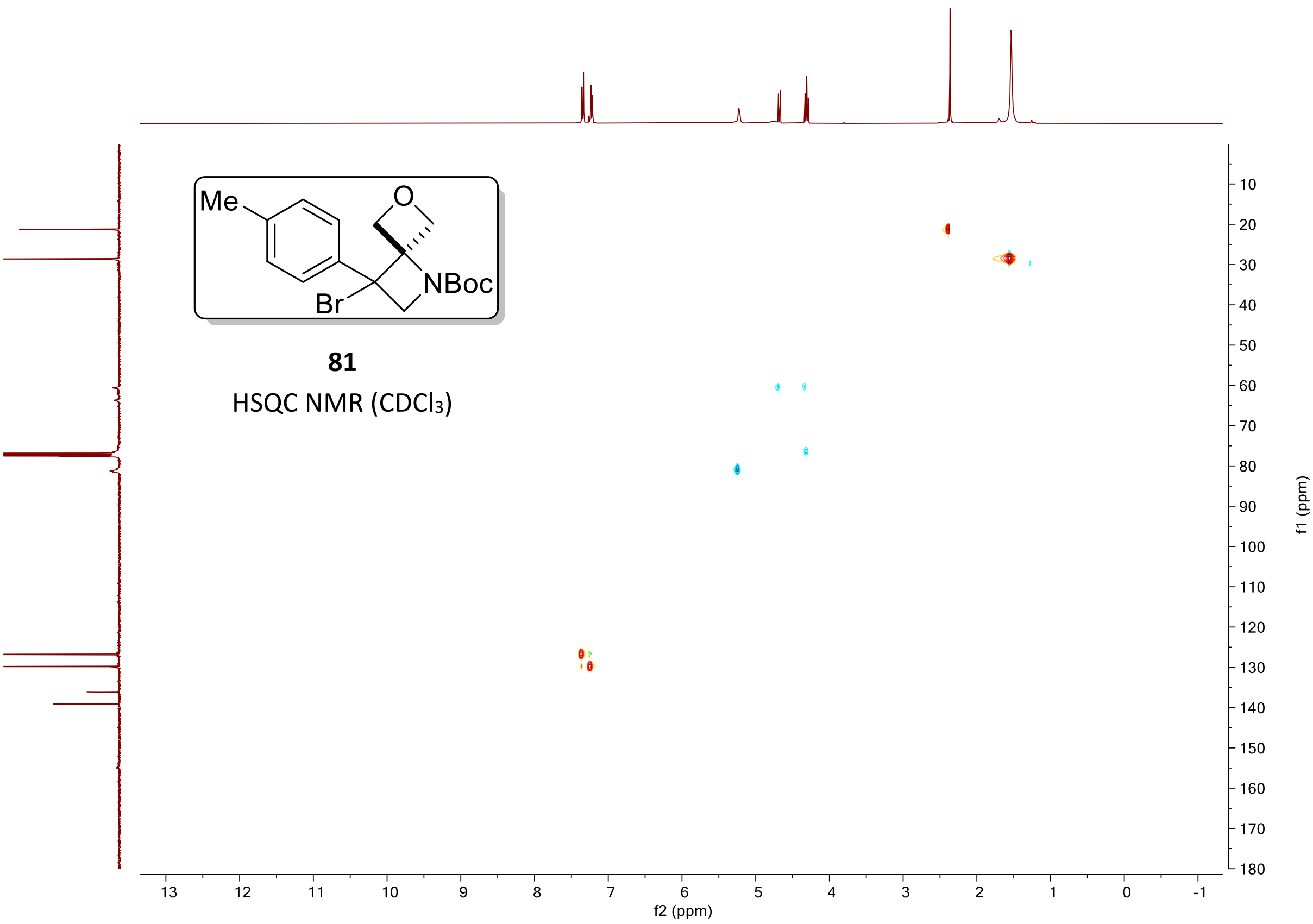
Me
O
NBoc
Br
81
HSQC NMR ($CDCl_3$)
f2 (ppm)
f1 (ppm)

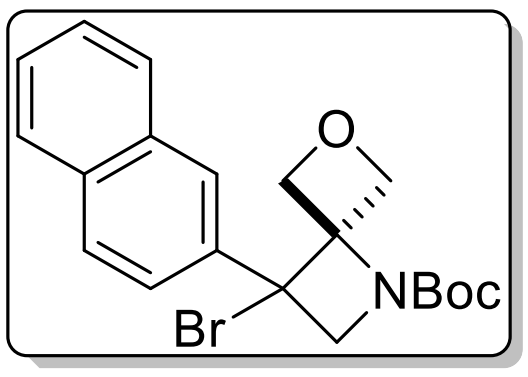


**82**

$^{1}$H NMR (400 MHz, $CDCl_3$)

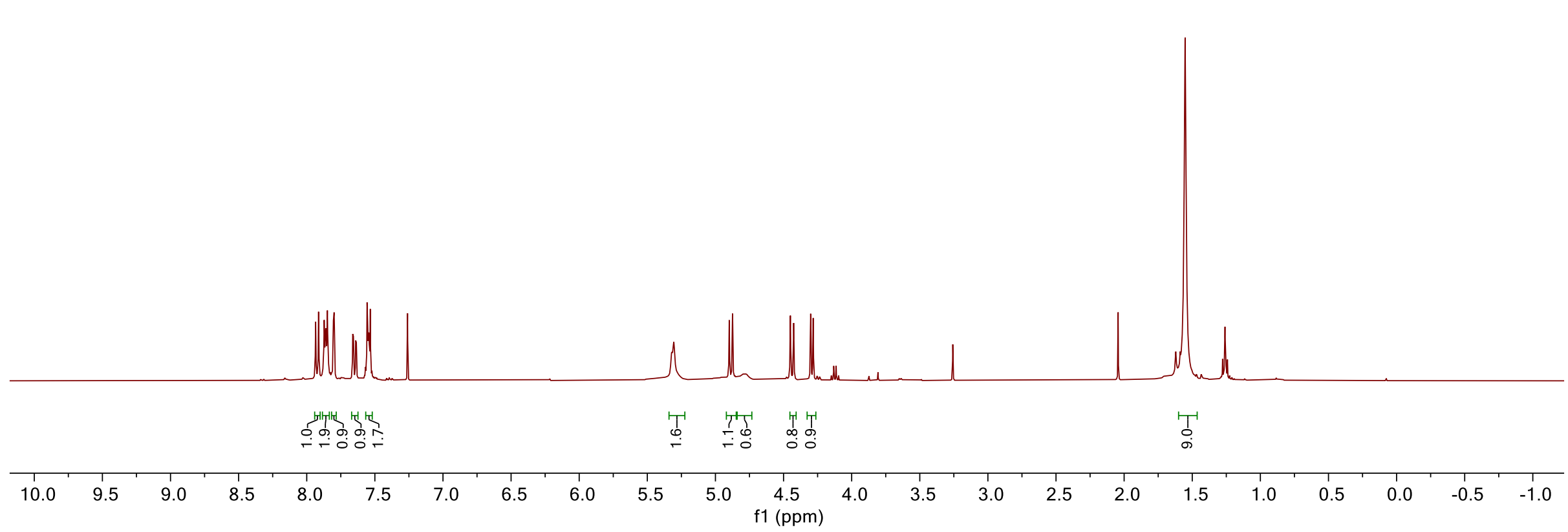


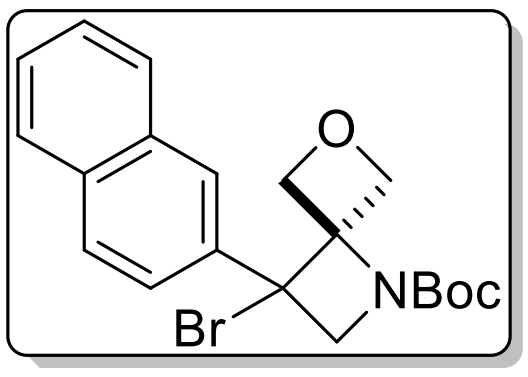


**82**

$^{13}$C {$^{1}$H} NMR (101 MHz, $CDCl_3$)

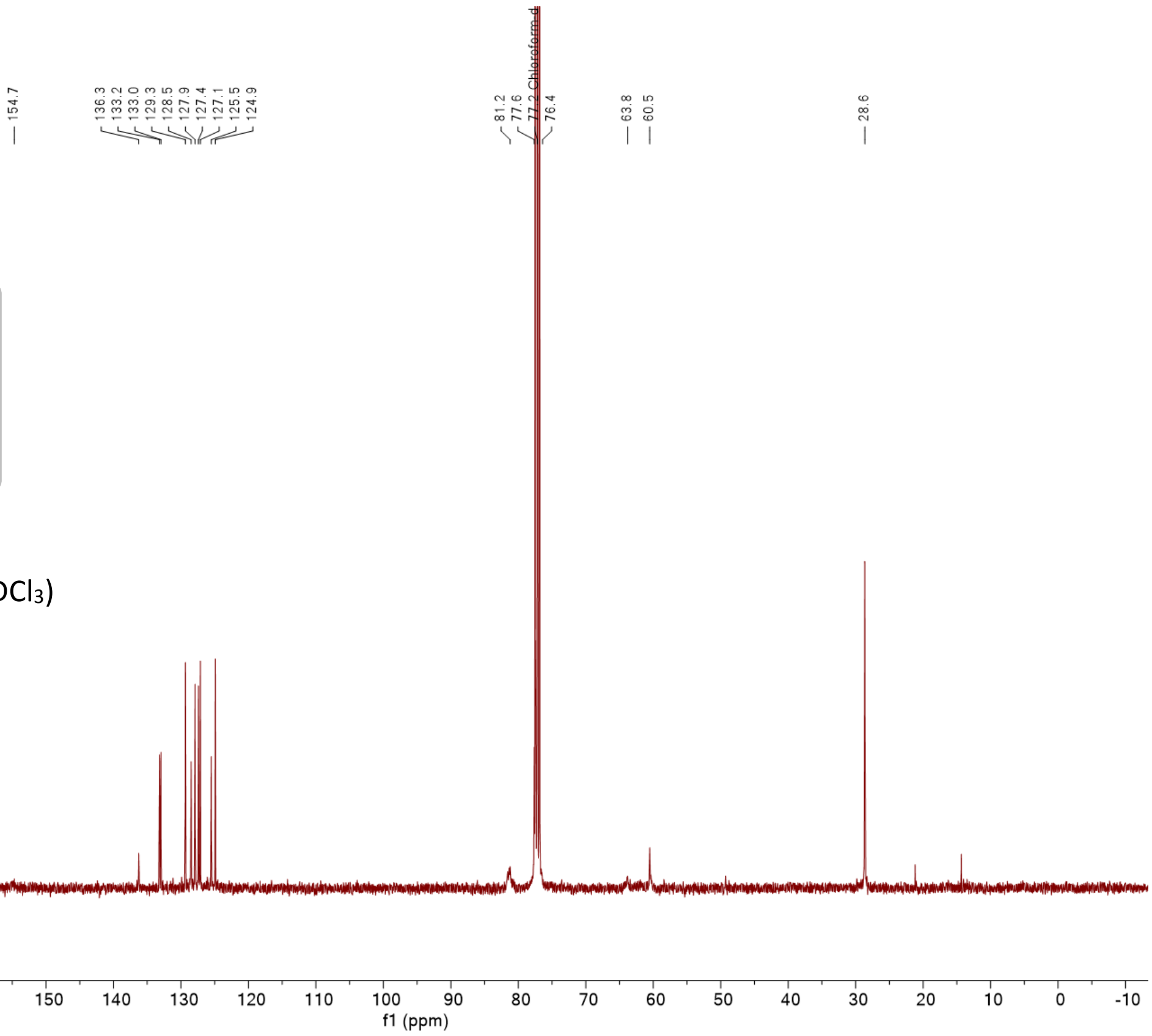

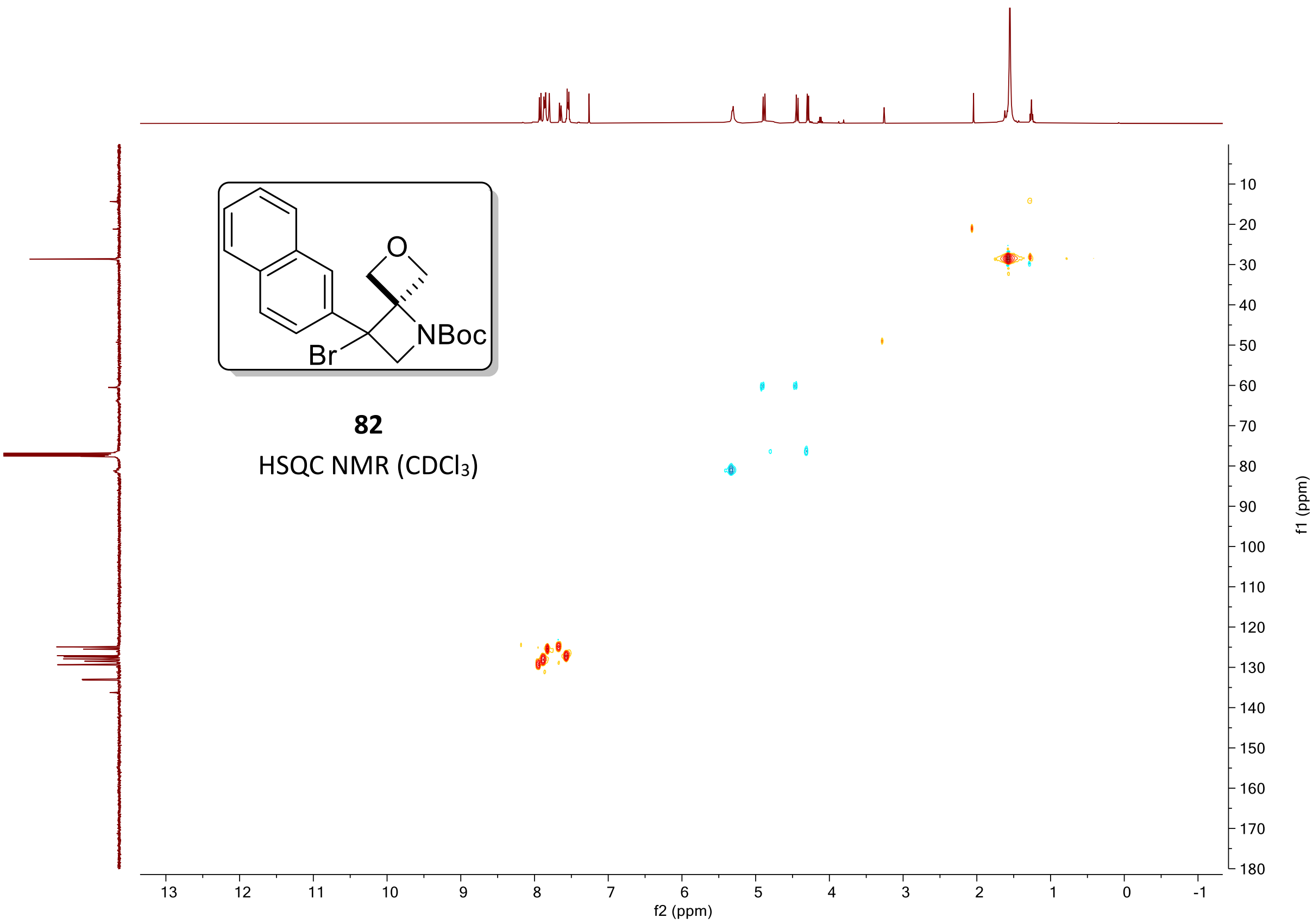


**82**

HSQC NMR ($CDCl_3$)

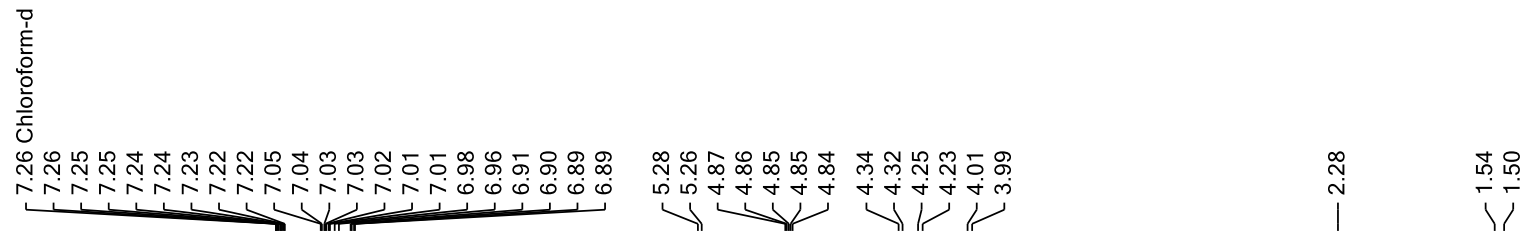


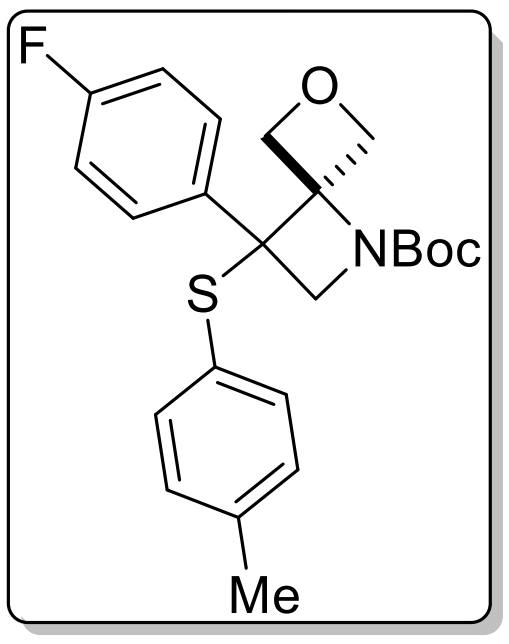


**83**

$^{1}$H NMR (400 MHz, $CDCl_3$)

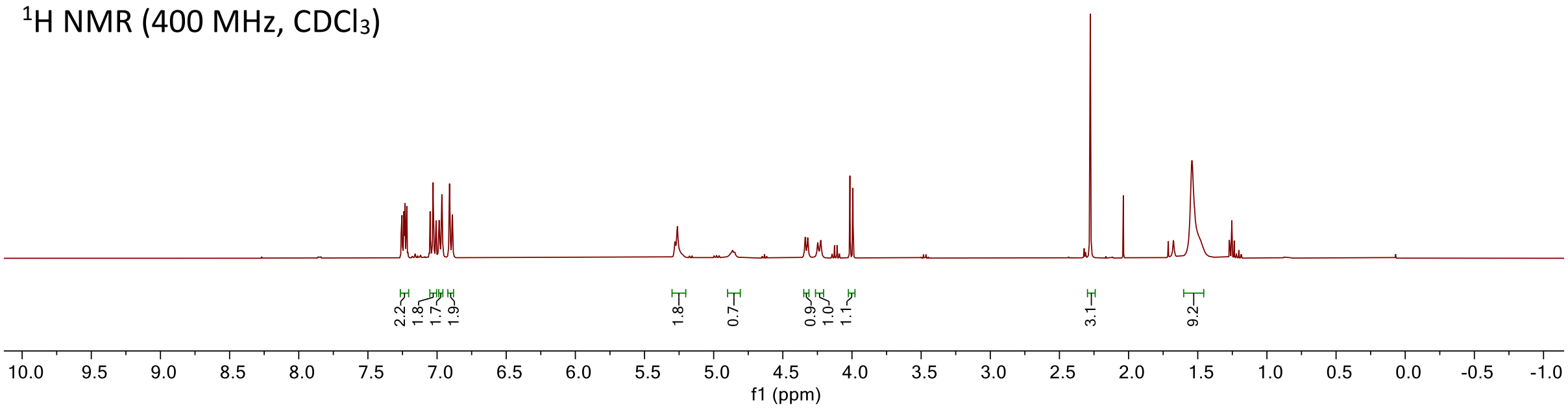


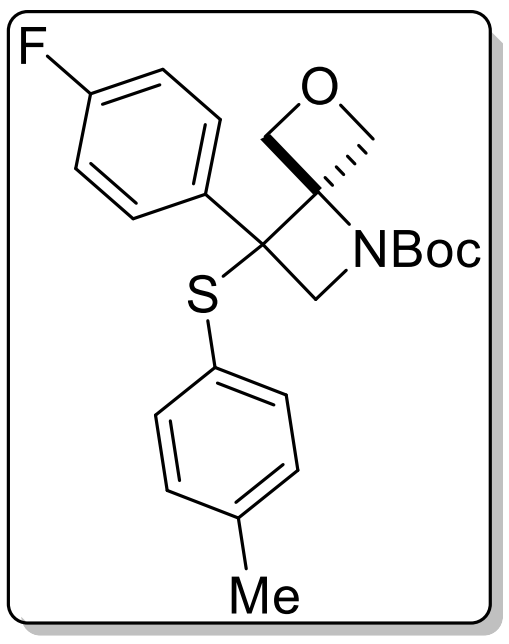


**83**

$^{19}$F NMR (377 MHz, $CDCl_3$)

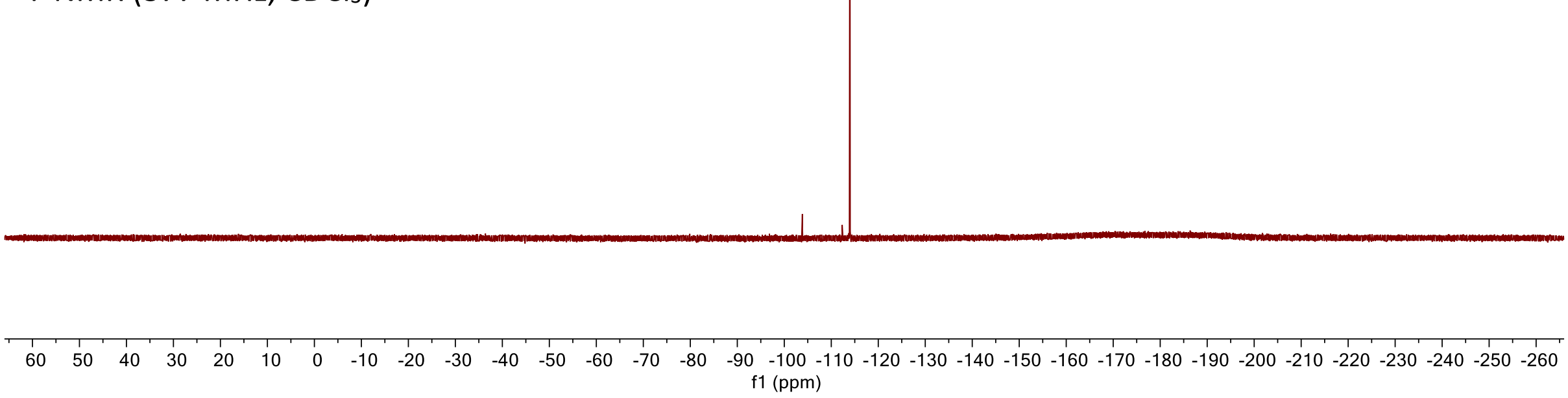

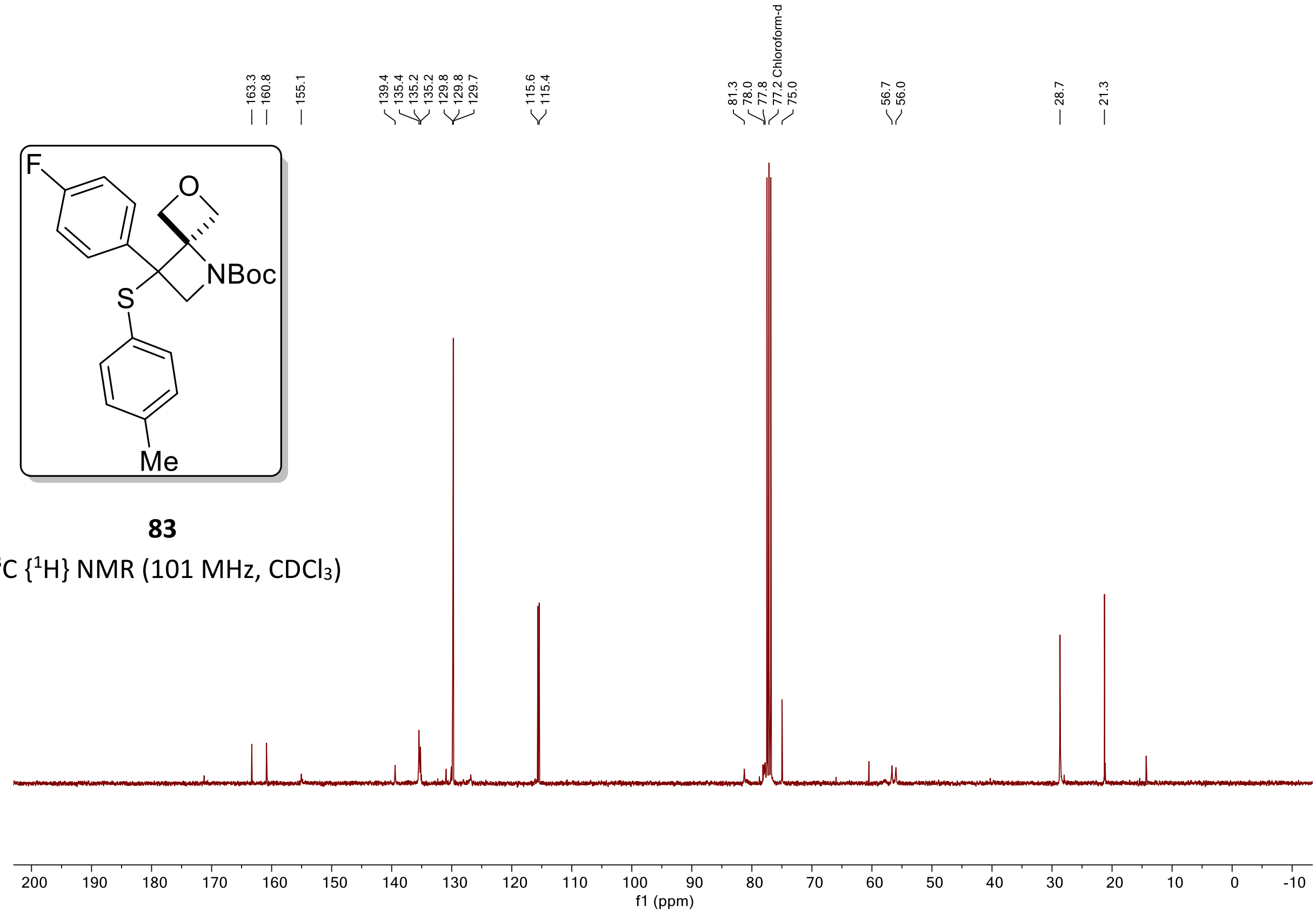


**83**

$^{13}C\ \{^{1}H\}$ NMR (101 MHz, $CDCl_3$)

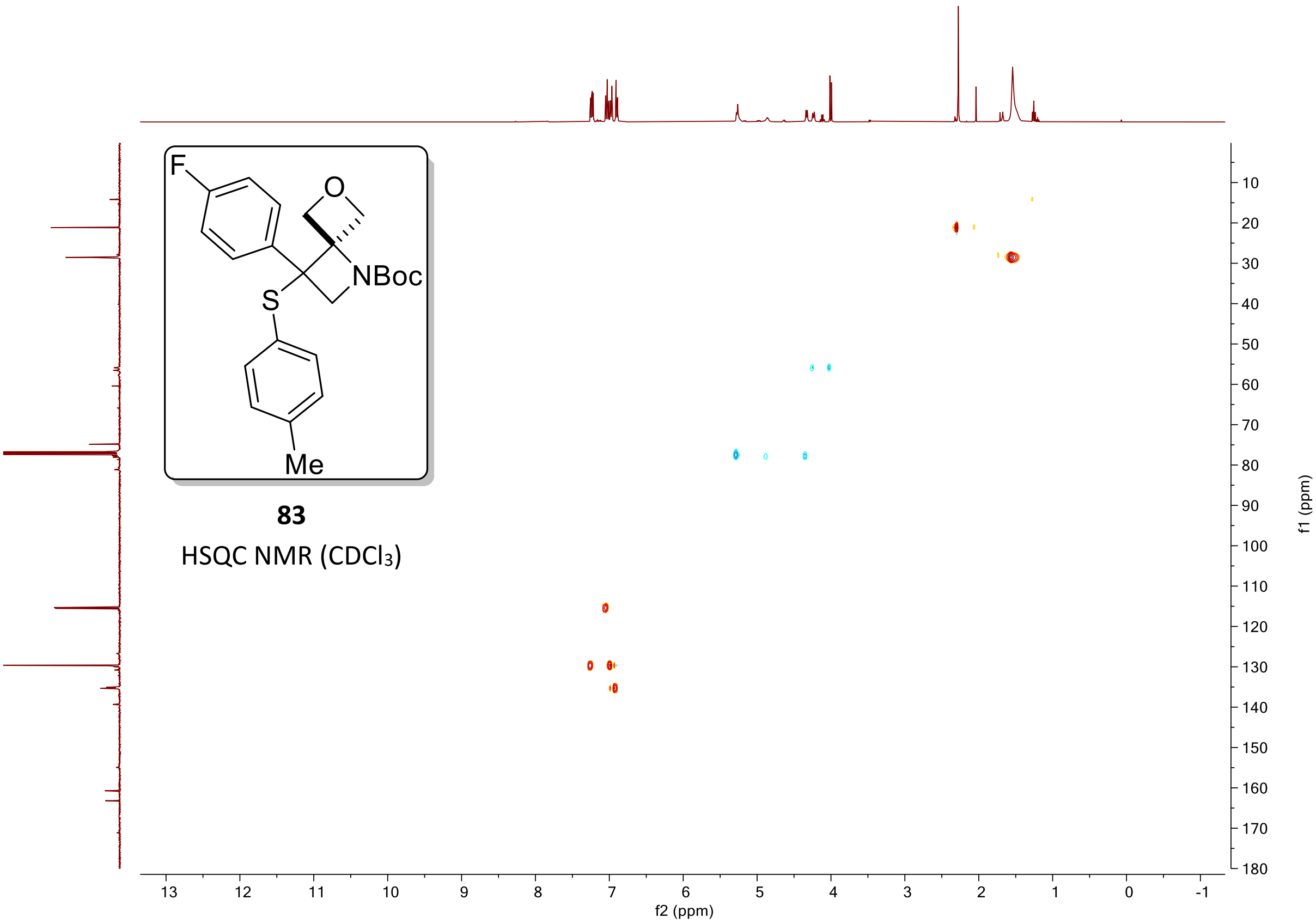


**83**

HSQC NMR ($CDCl_3$)

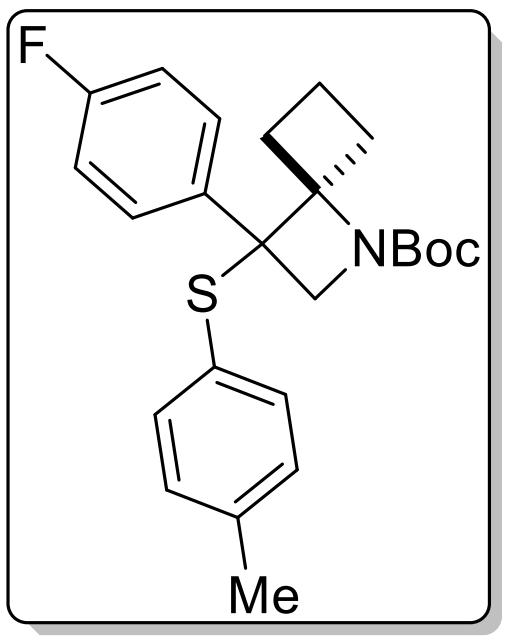


**84**

$^{1}$H NMR (400 MHz, $CDCl_3$)

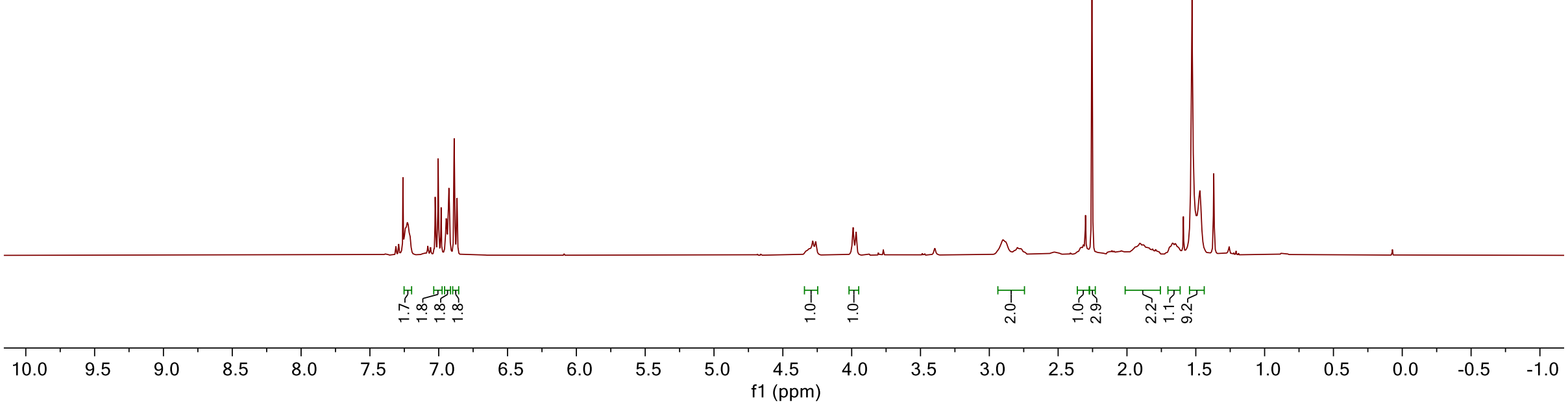


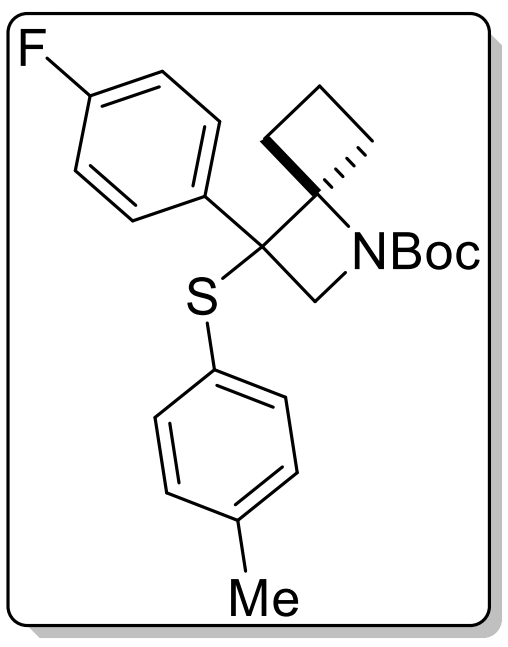


**84**

$^{19}$F NMR (377 MHz, $CDCl_3$)

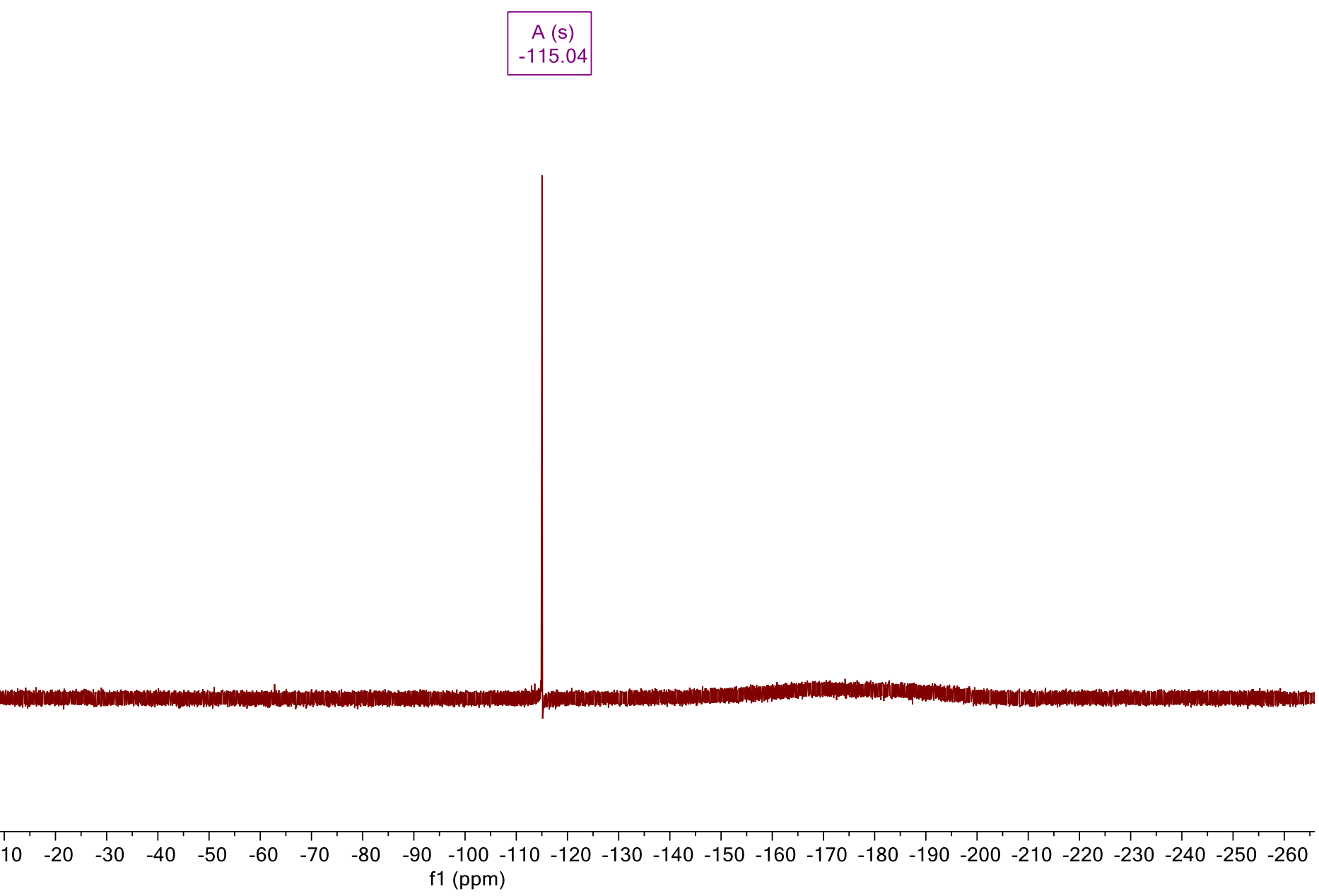

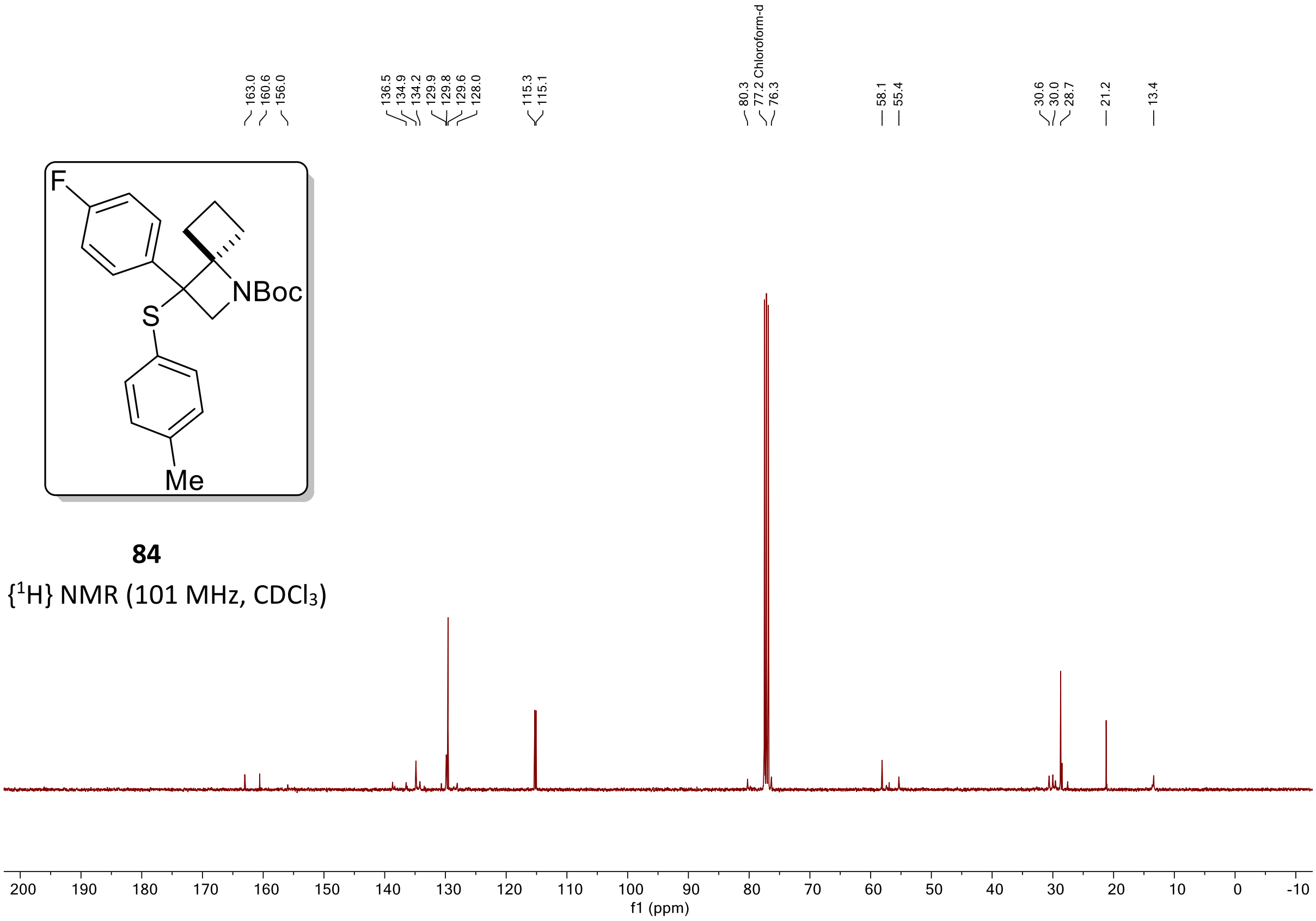


**84**

$^{13}C\ \{^{1}H\}$ NMR (101 MHz, $CDCl_3$)

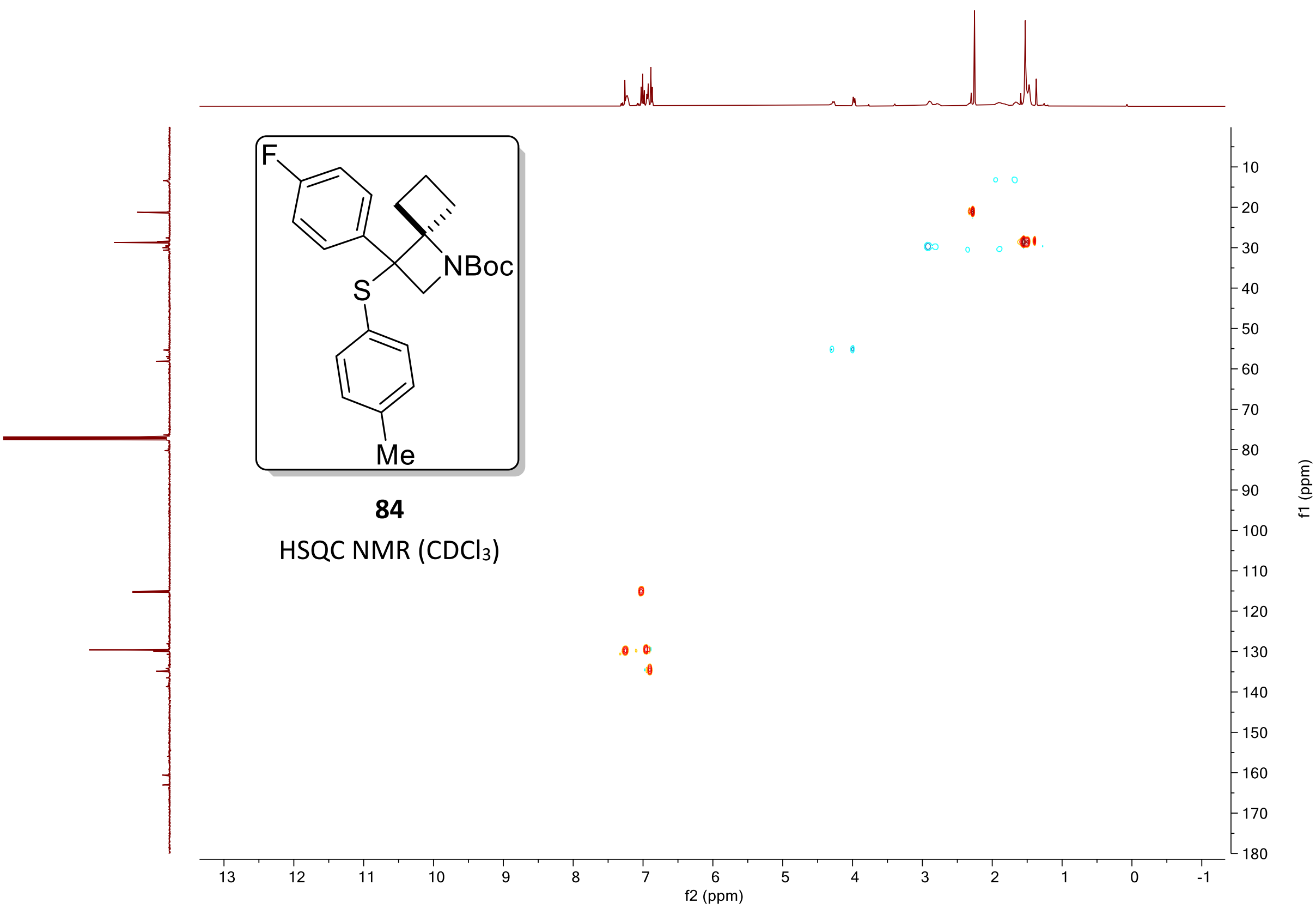


**84**

HSQC NMR ($CDCl_3$)

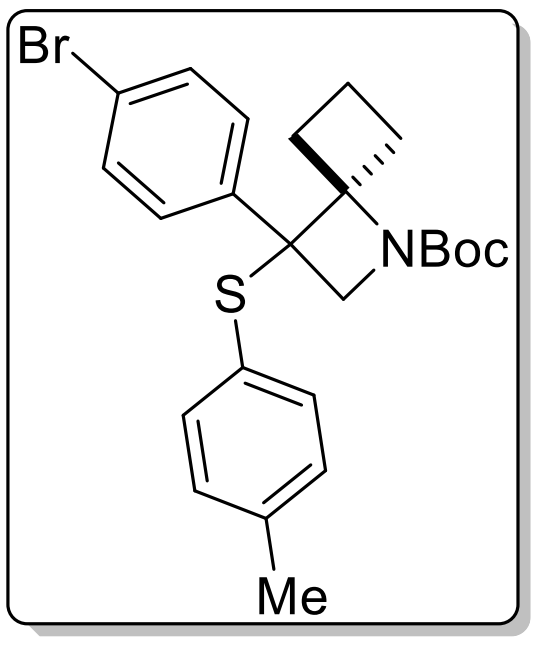


**85**

$^{1}$H NMR (400 MHz, $CDCl_3$)

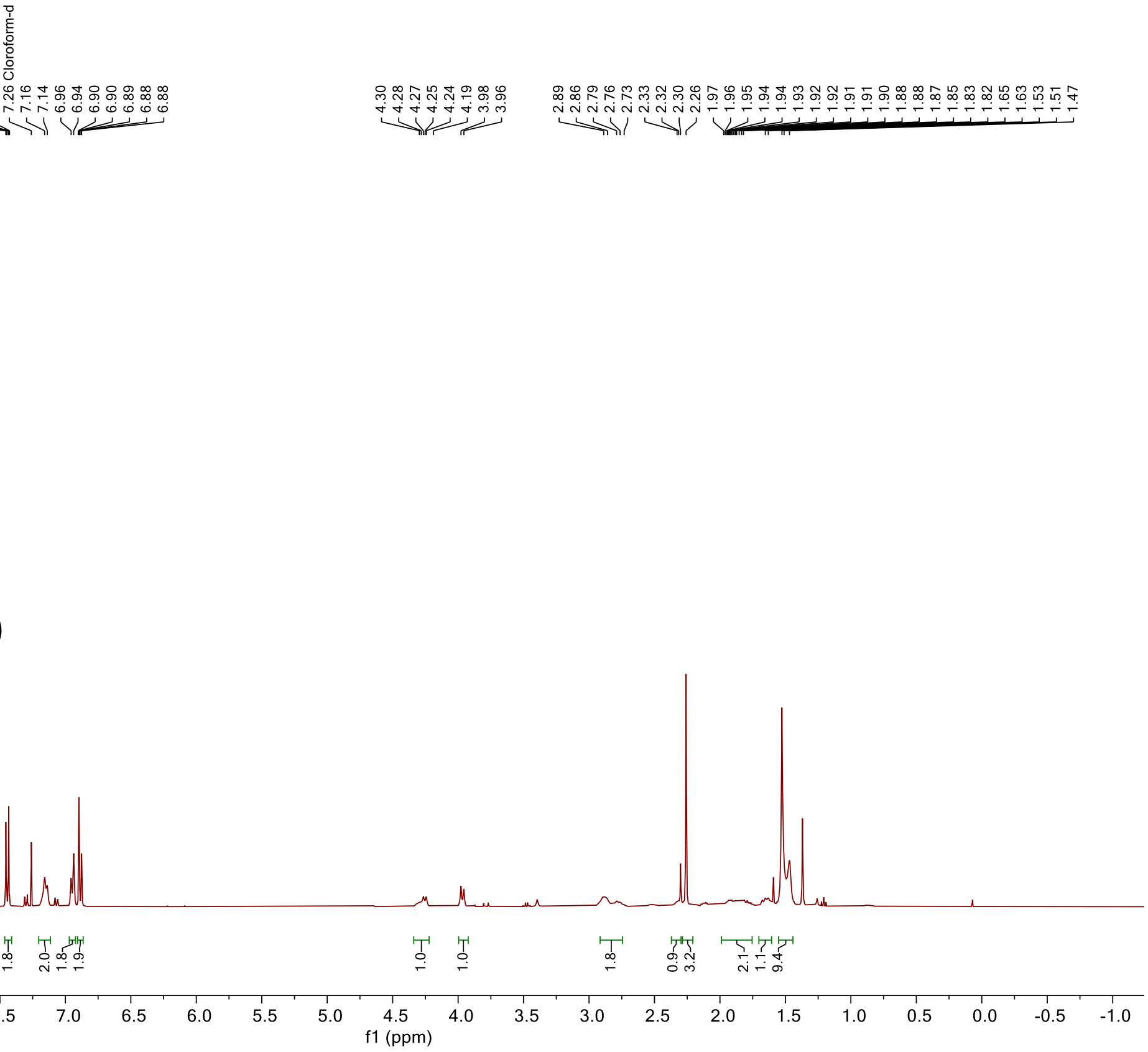


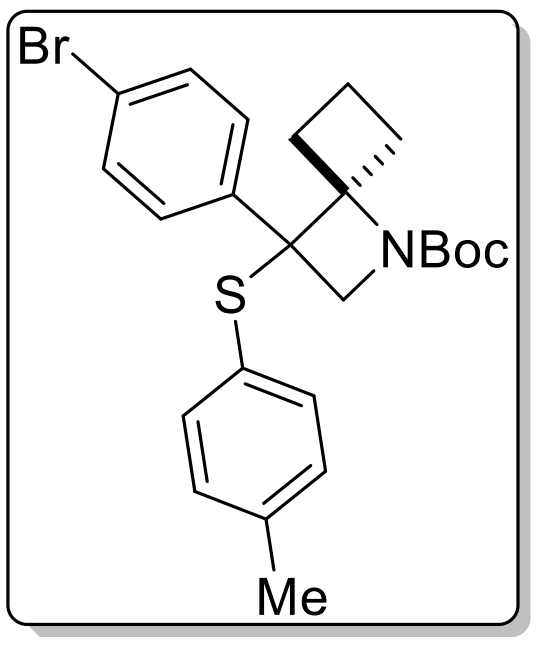


**85**

$^{13}$C {$^{1}$H} NMR (101 MHz, $CDCl_3$)

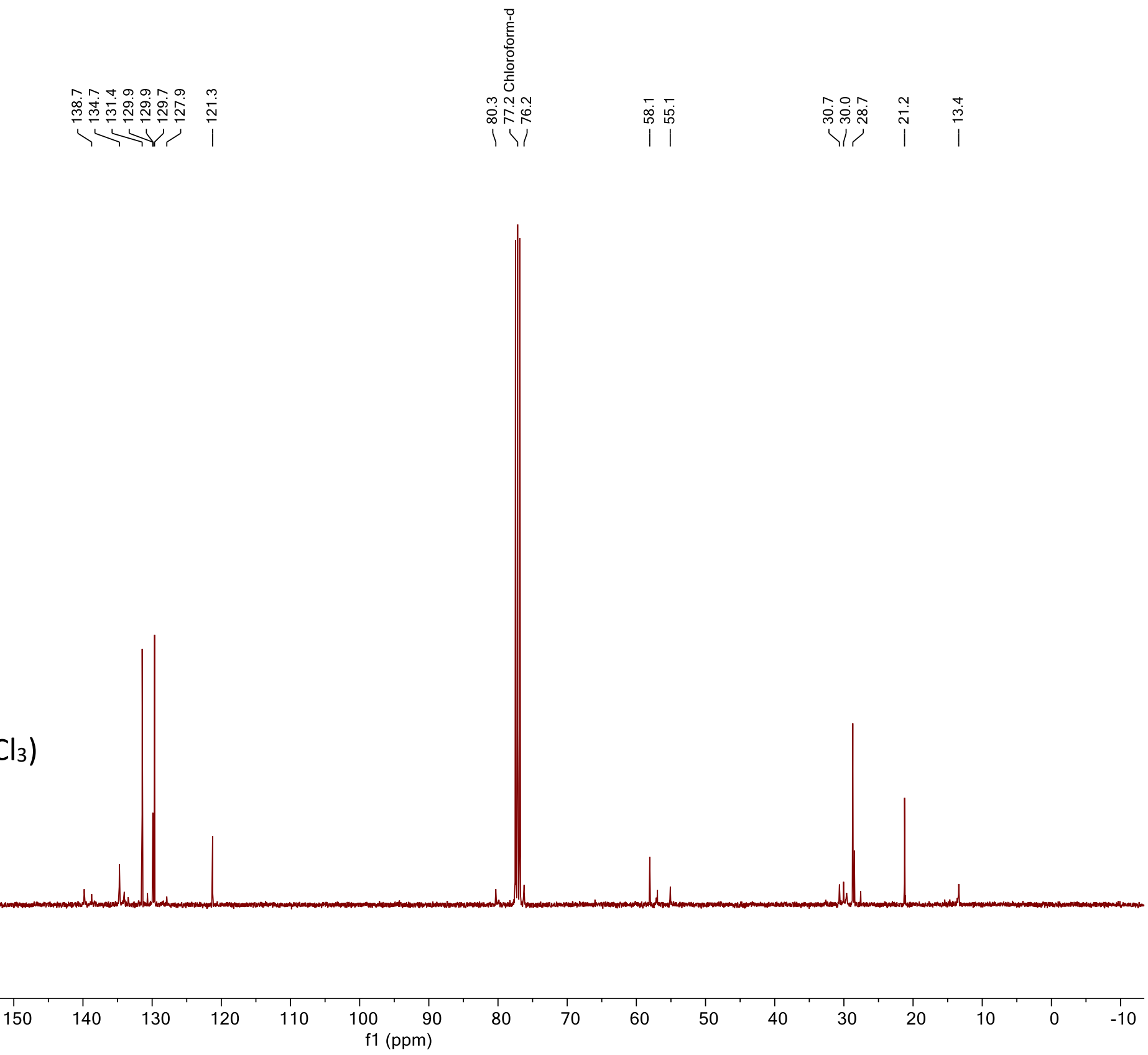

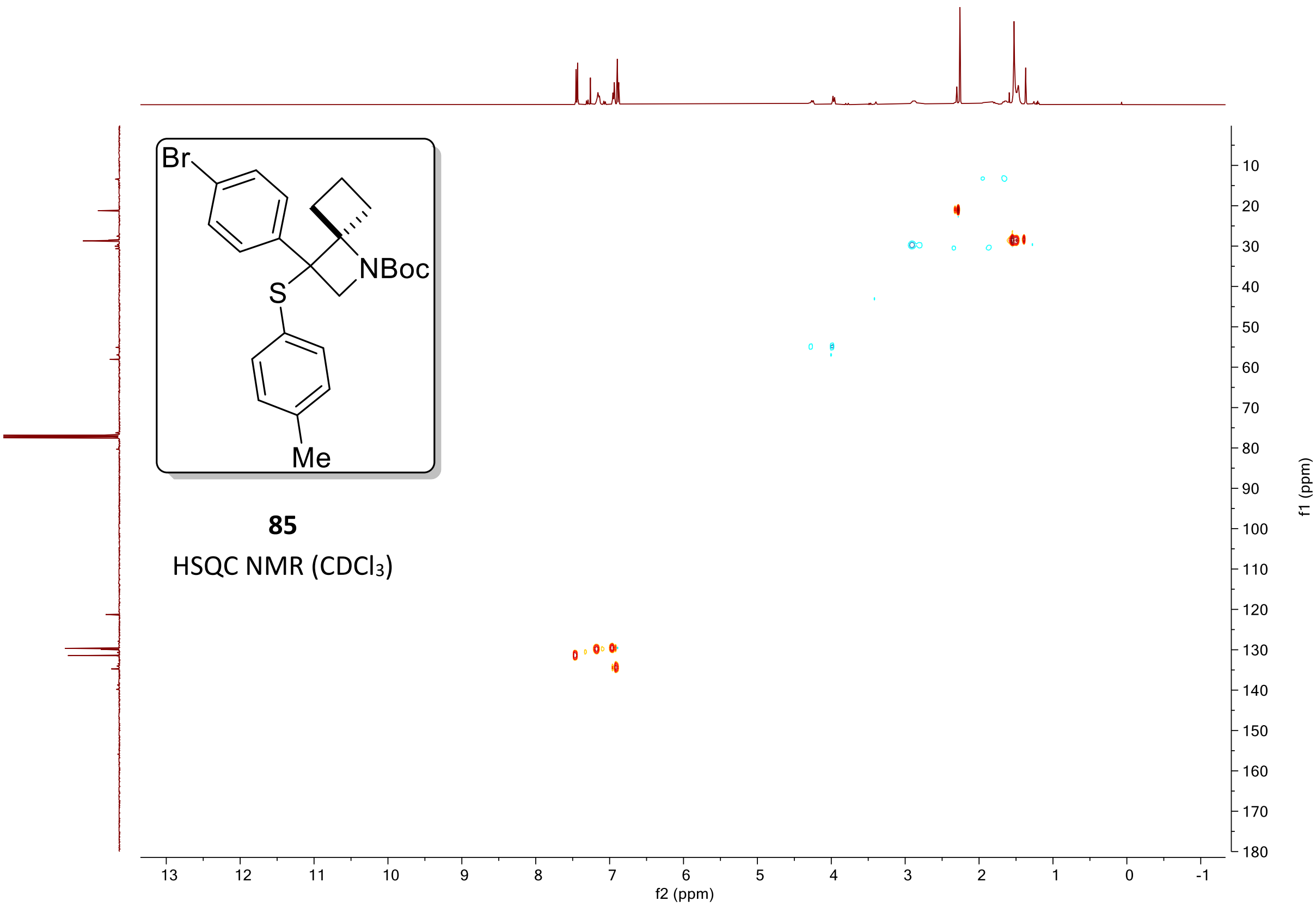
Br
NBoc
S
Me
85
HSQC NMR ($CDCl_3$)
f2 (ppm)
f1 (ppm)

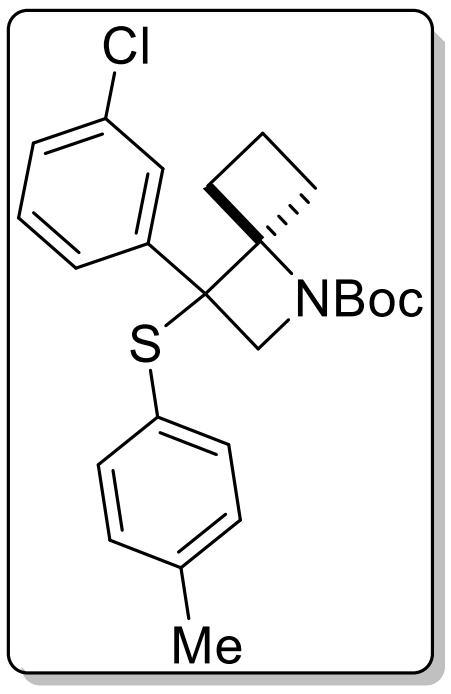


**86**

$^1$H NMR (400 MHz, $CDCl_3$)

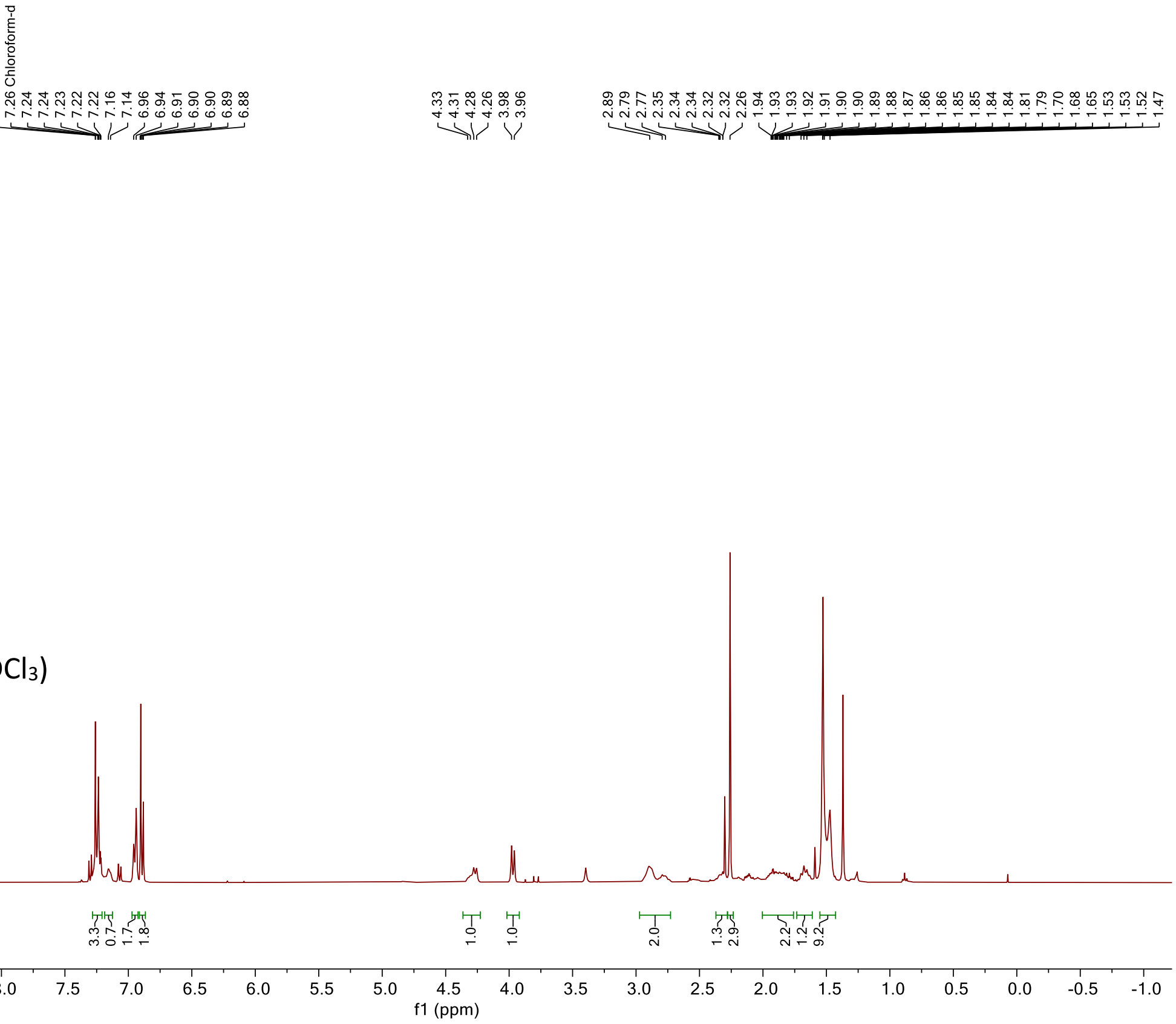


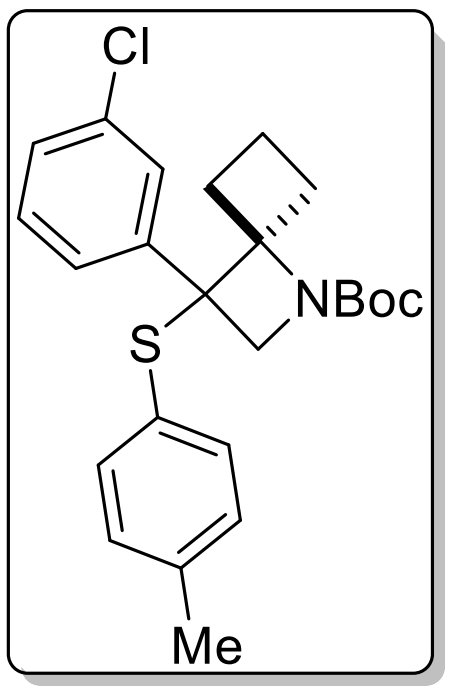


**86**

$^{13}$C {$^1$H} NMR (101 MHz, $CDCl_3$)

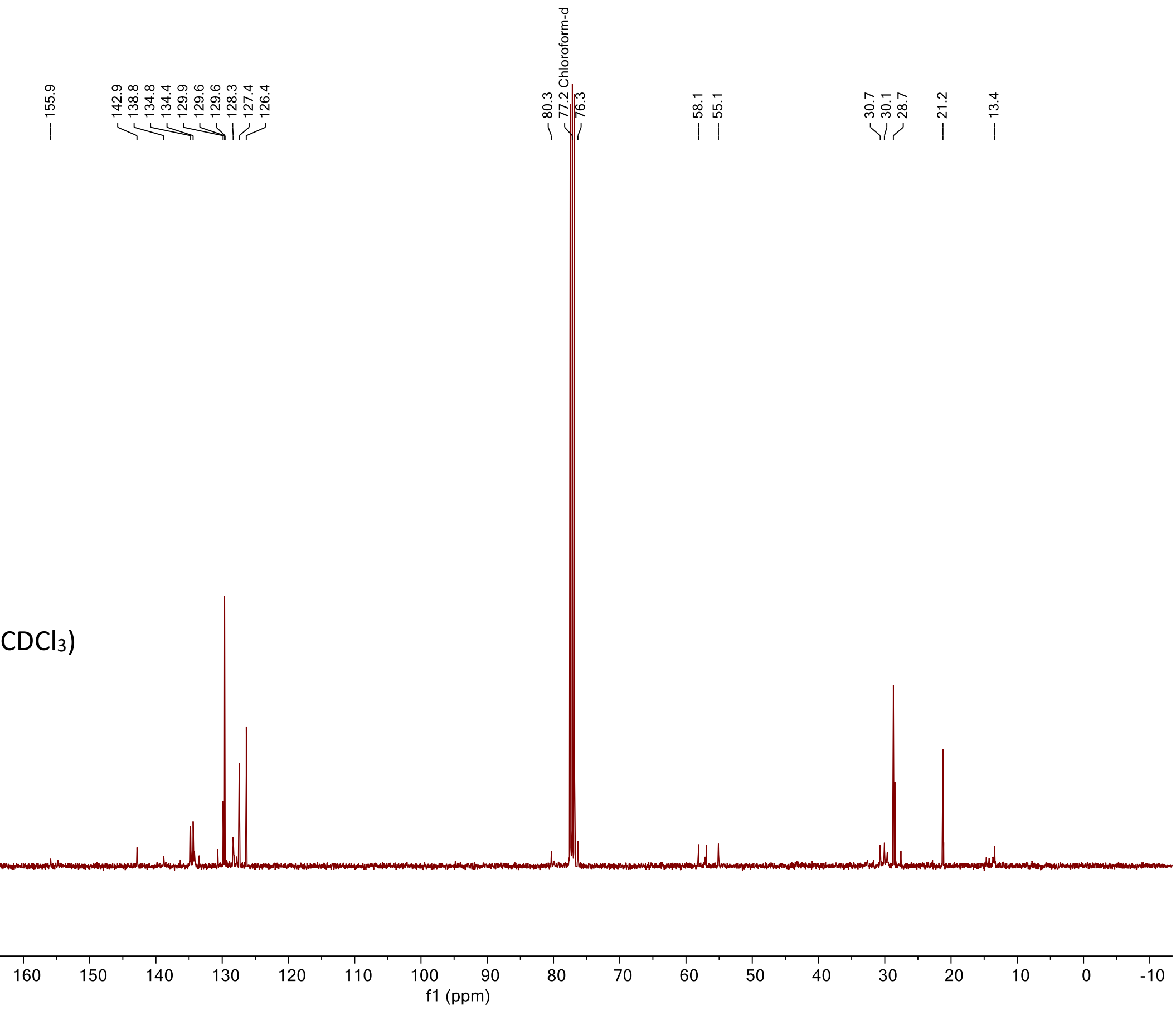

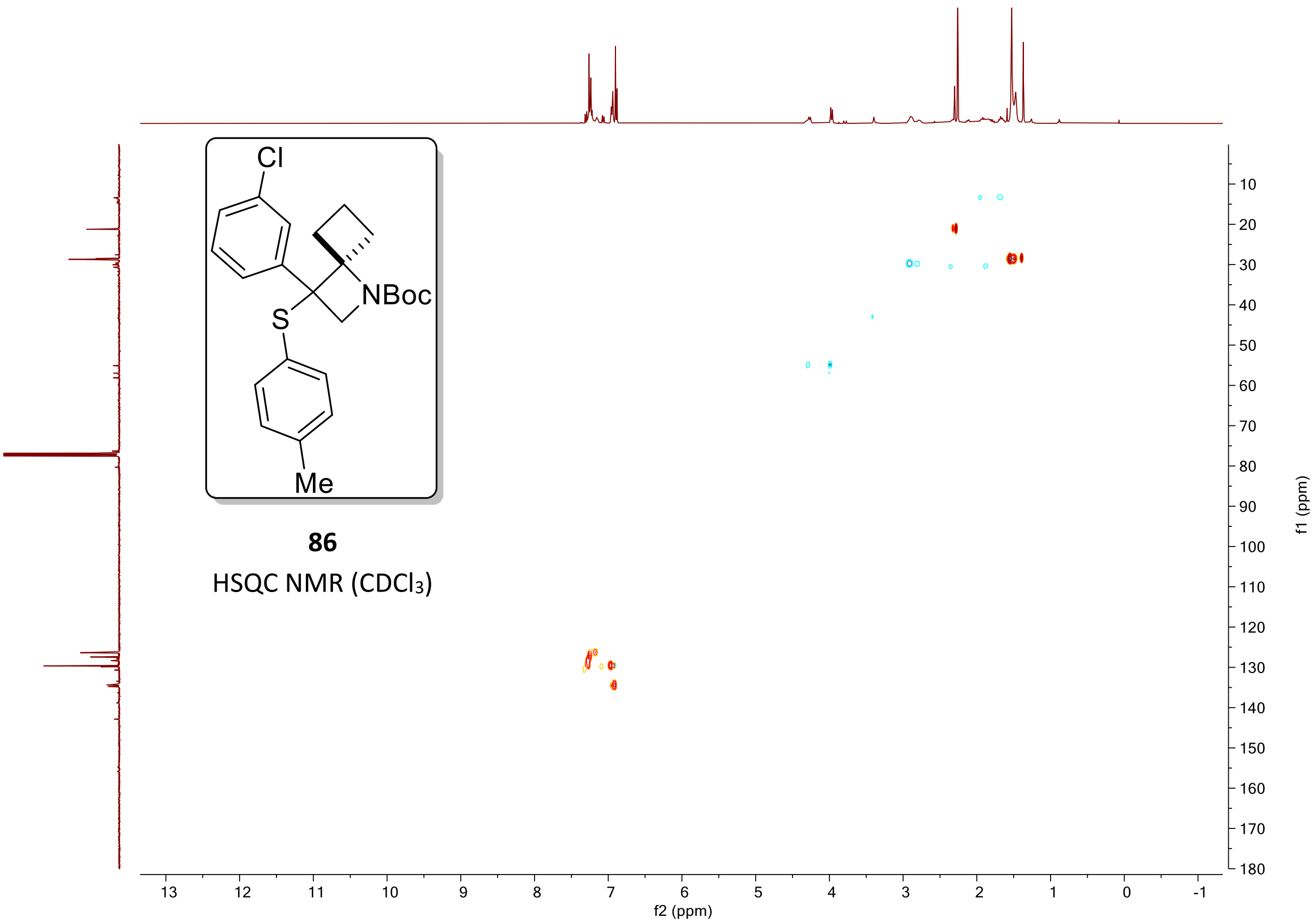


**86**
HSQC NMR ($CDCl_3$)

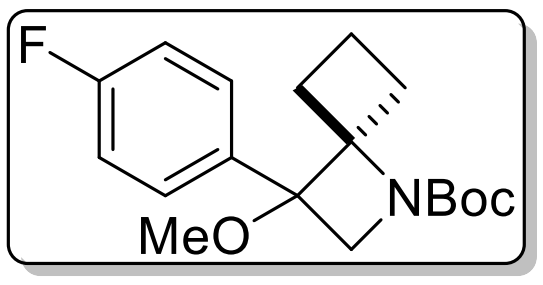

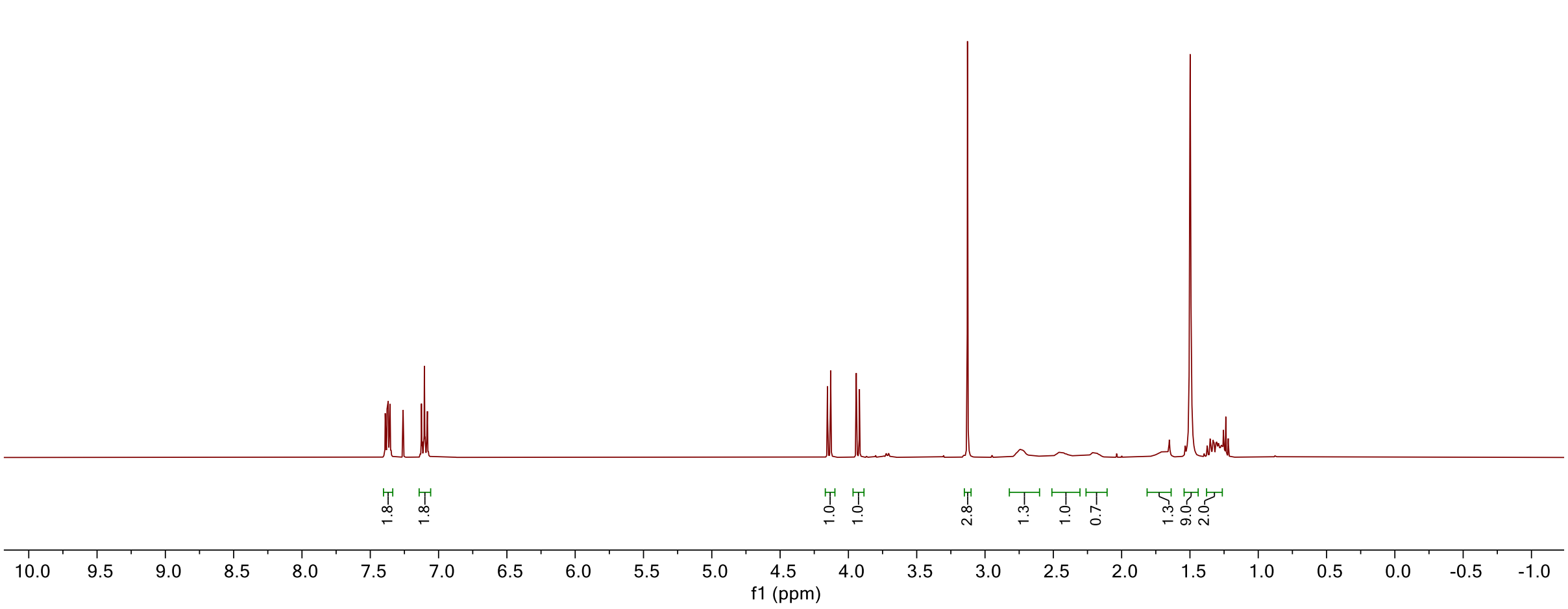


**87**

$^{1}$H NMR (400 MHz, $CDCl_3$)

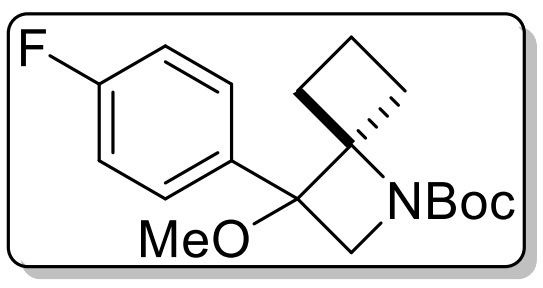

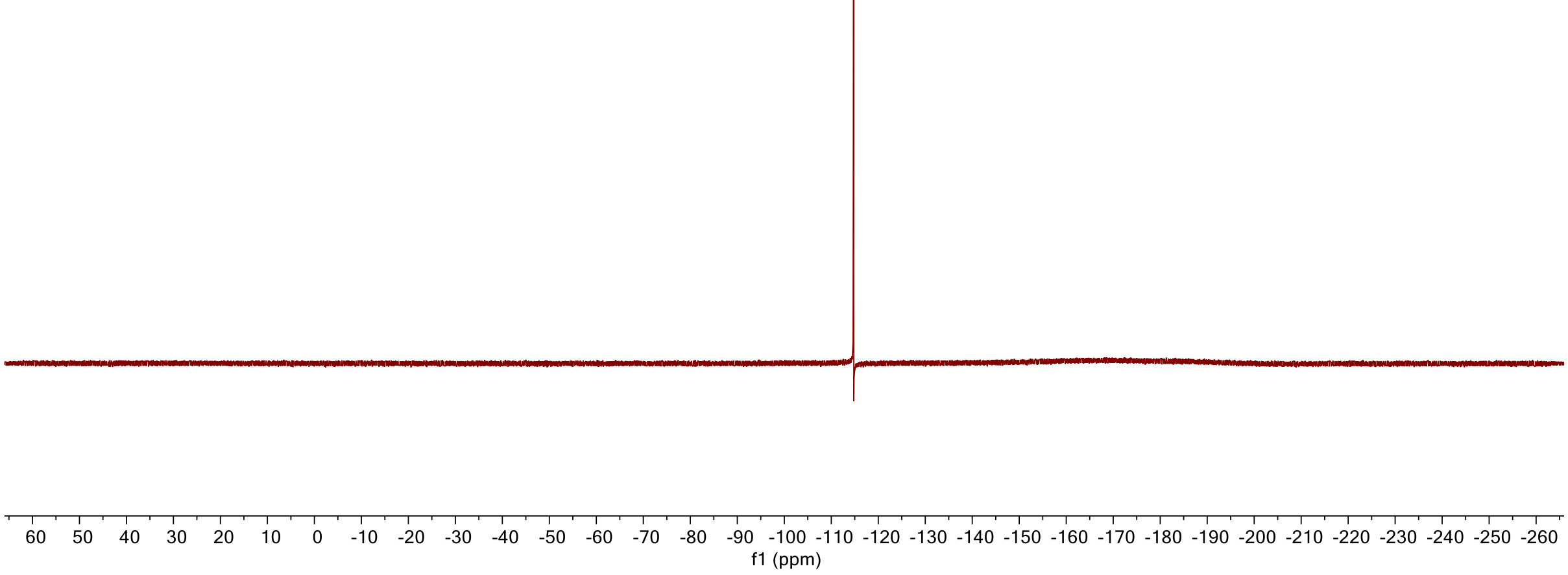


**87**

$^{19}$F NMR (377 MHz, $CDCl_3$)

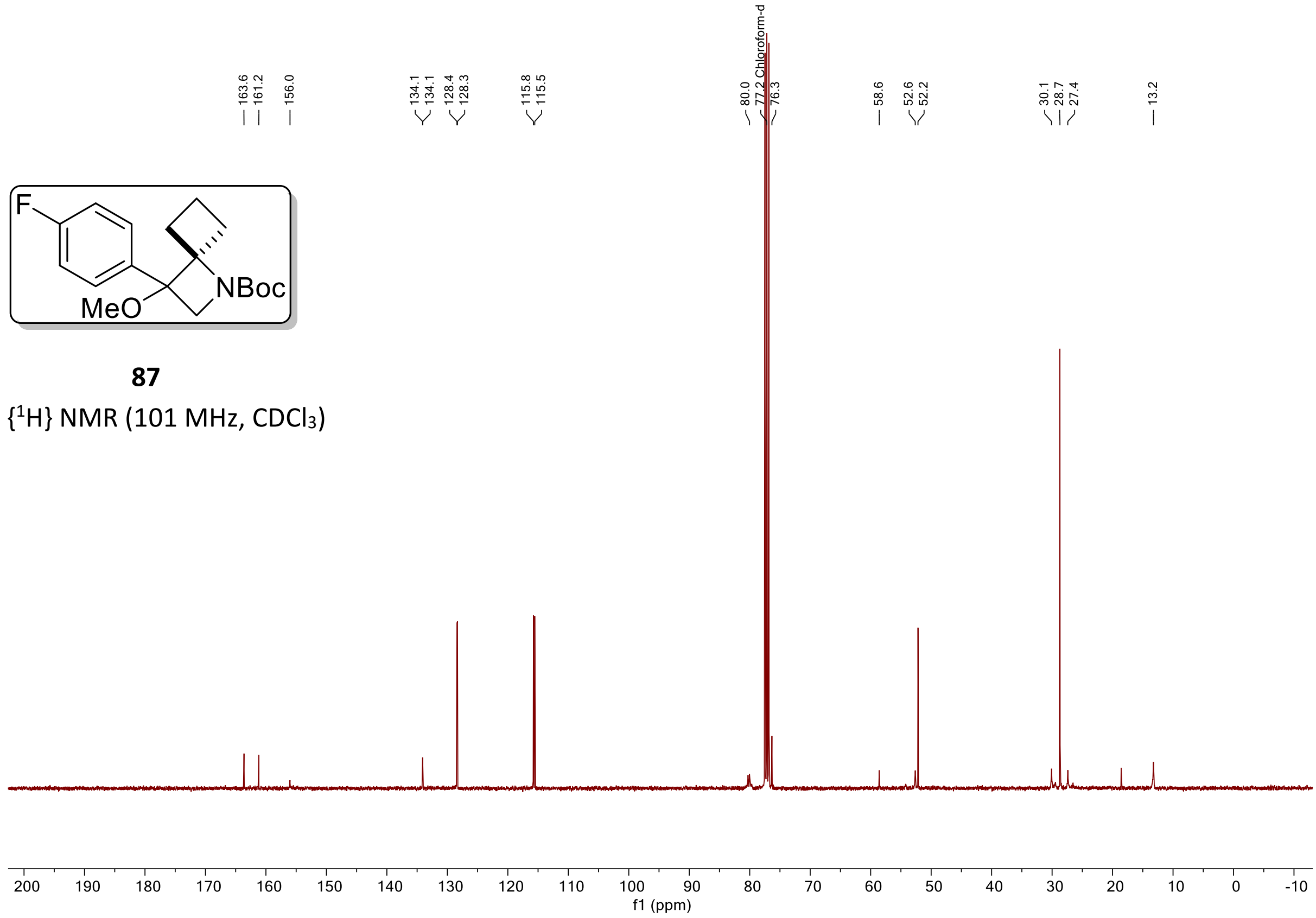


**87**

$^{13}C\{^{1}H\}$ NMR (101 MHz, $CDCl_3$)

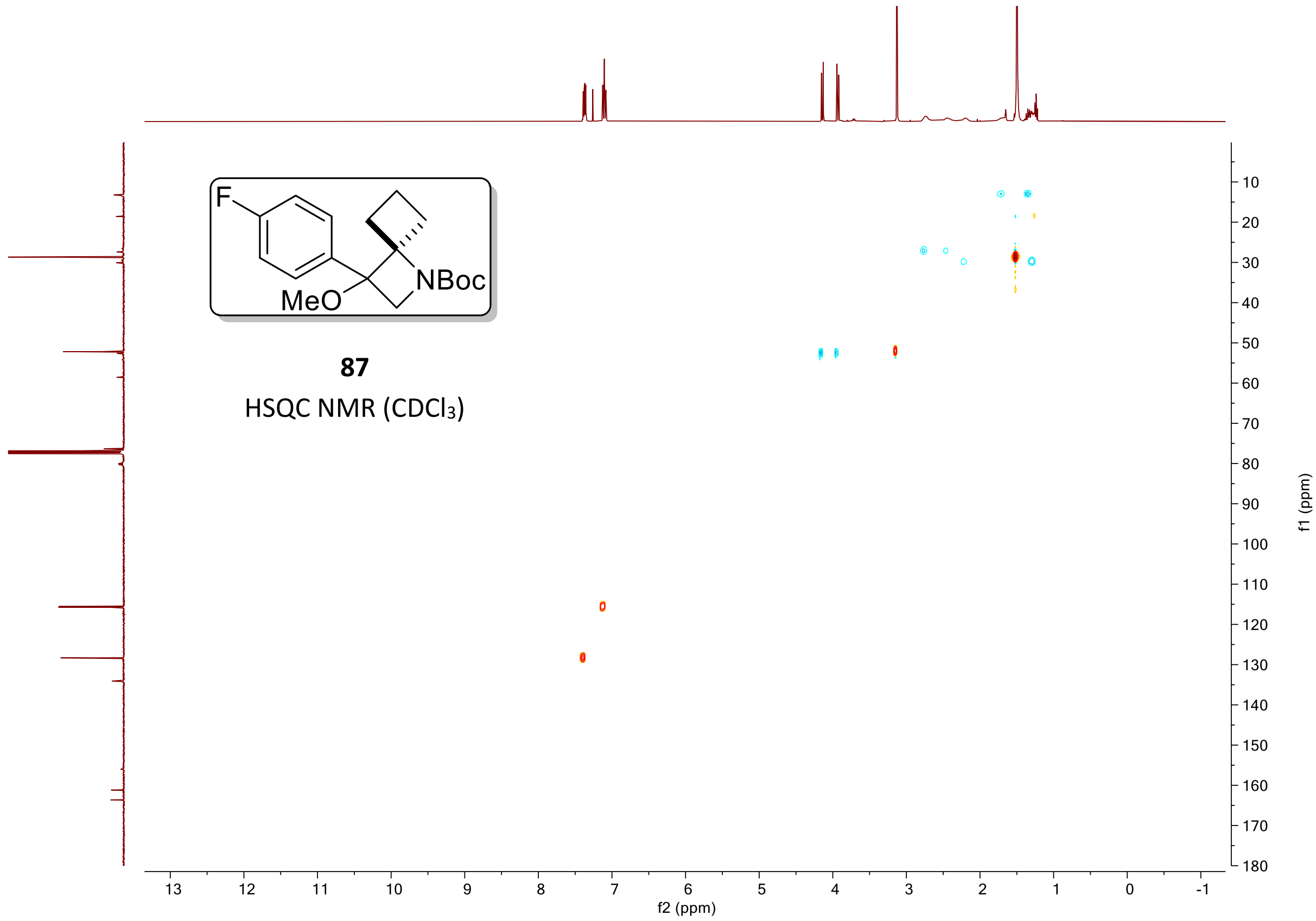


**87**

HSQC NMR ($CDCl_3$)

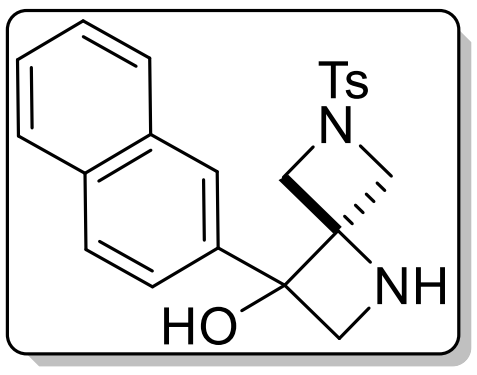

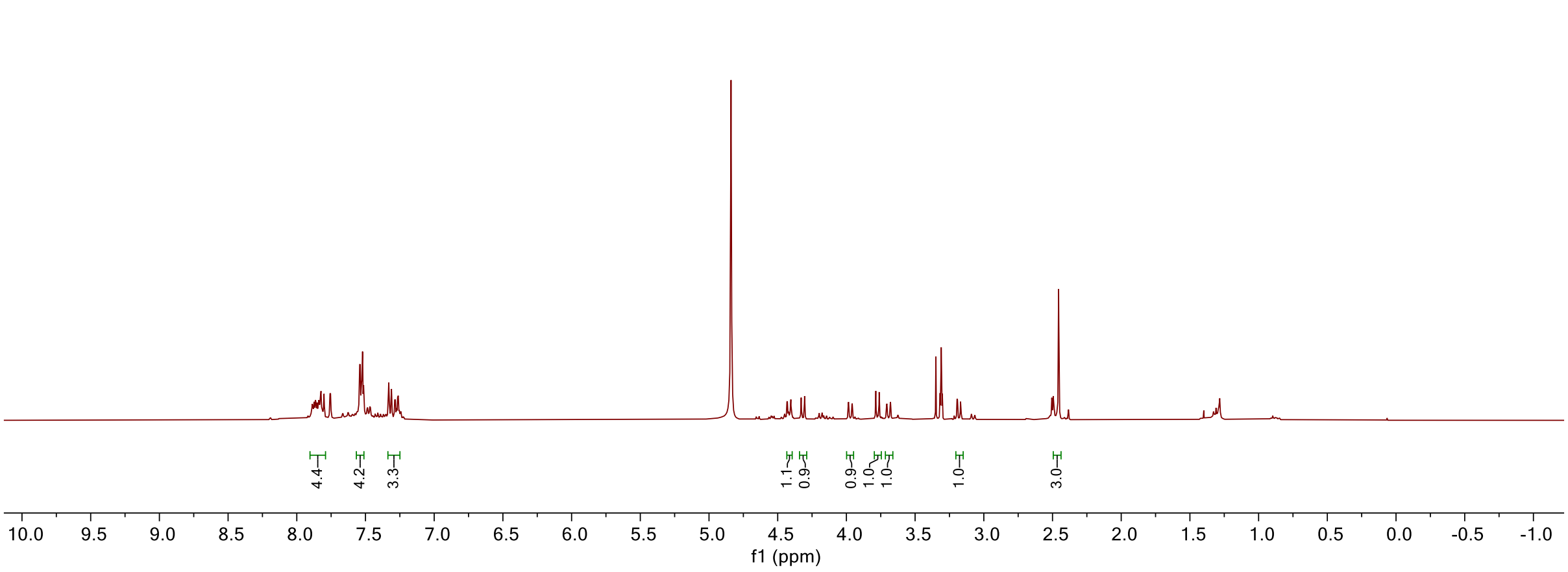


**88**

$^{1}H$ NMR (400 MHz, $CD_3OD$)

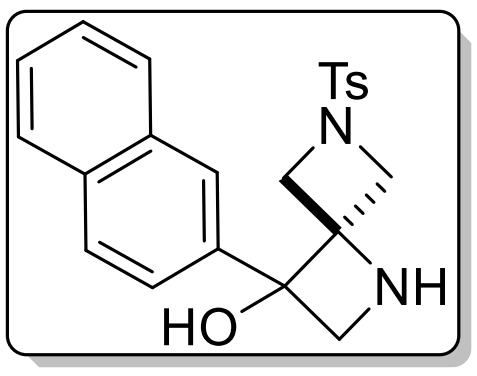

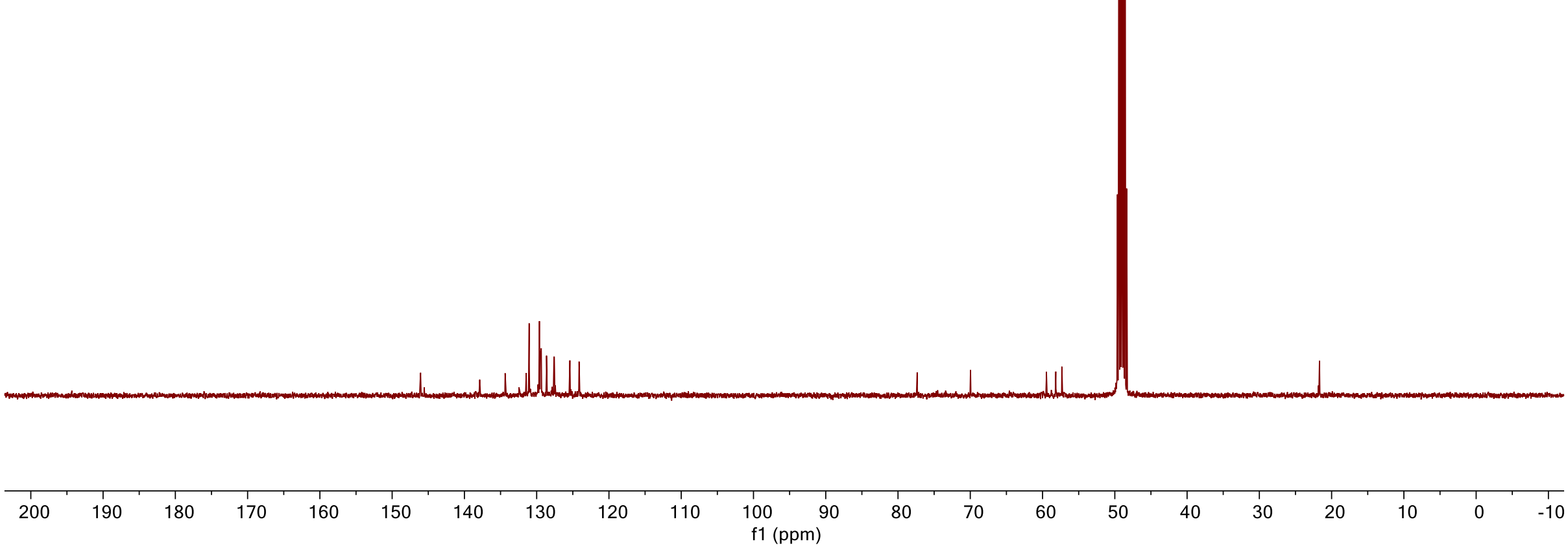


**88**

$^{13}C$ {$^{1}H$} NMR (101 MHz, $CD_3OD$)

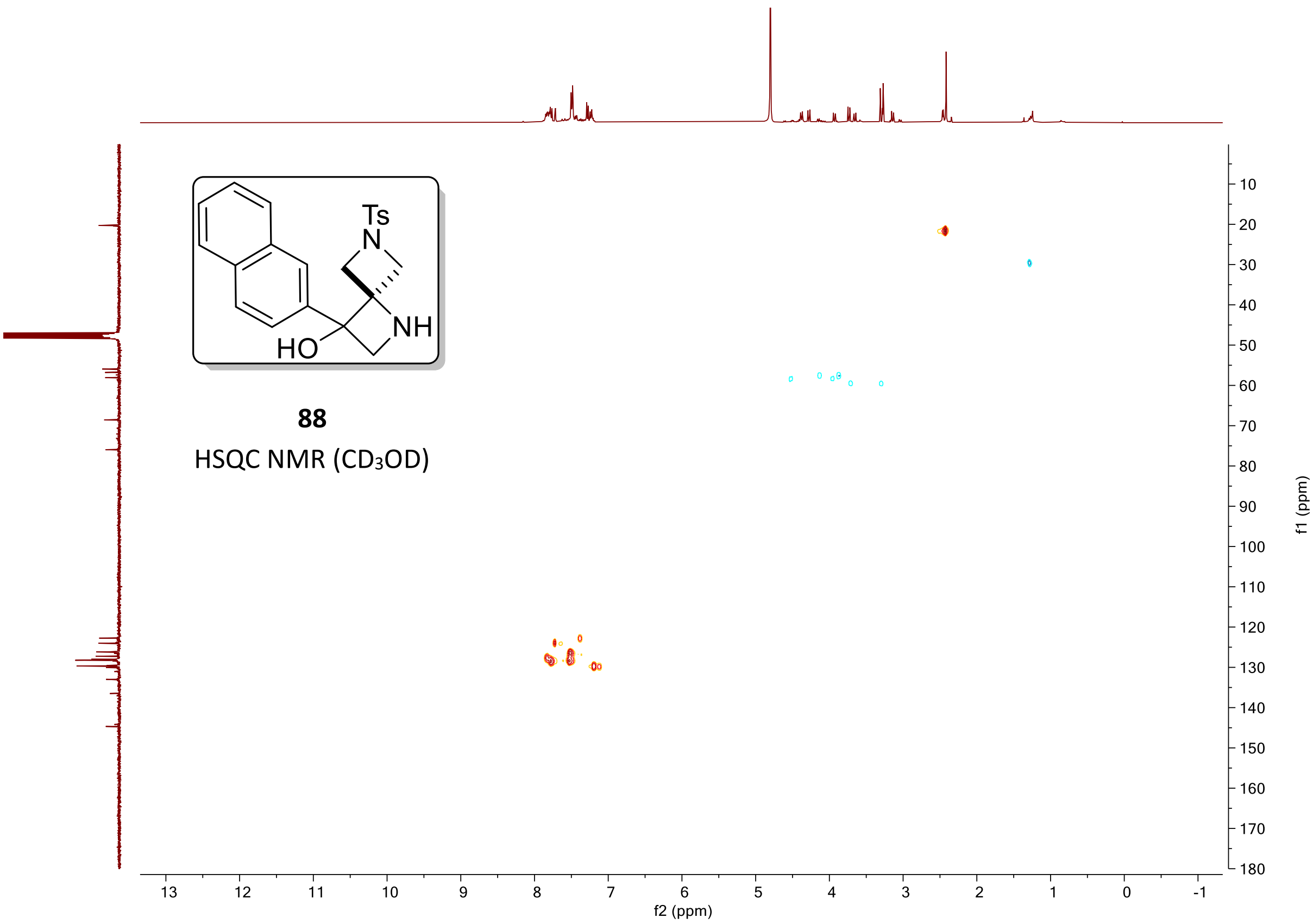
Ts
N
NH
HO
88
HSQC NMR (CD3OD)
f2 (ppm)
f1 (ppm)

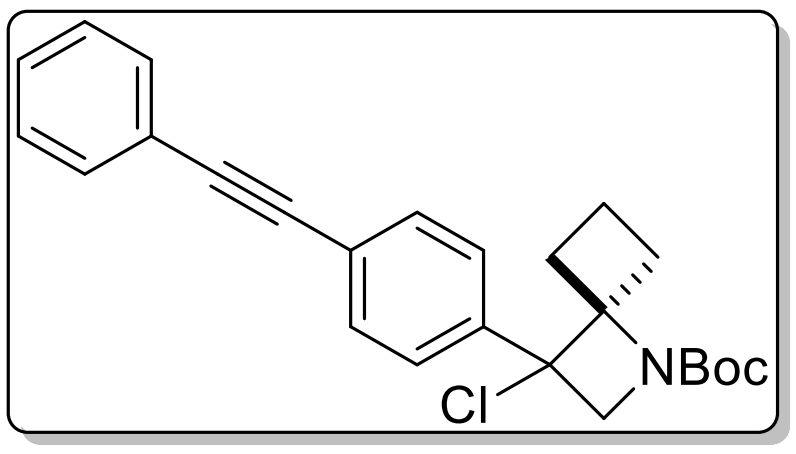


**89**

$^{1}$H NMR (400 MHz, $CDCl_3$)

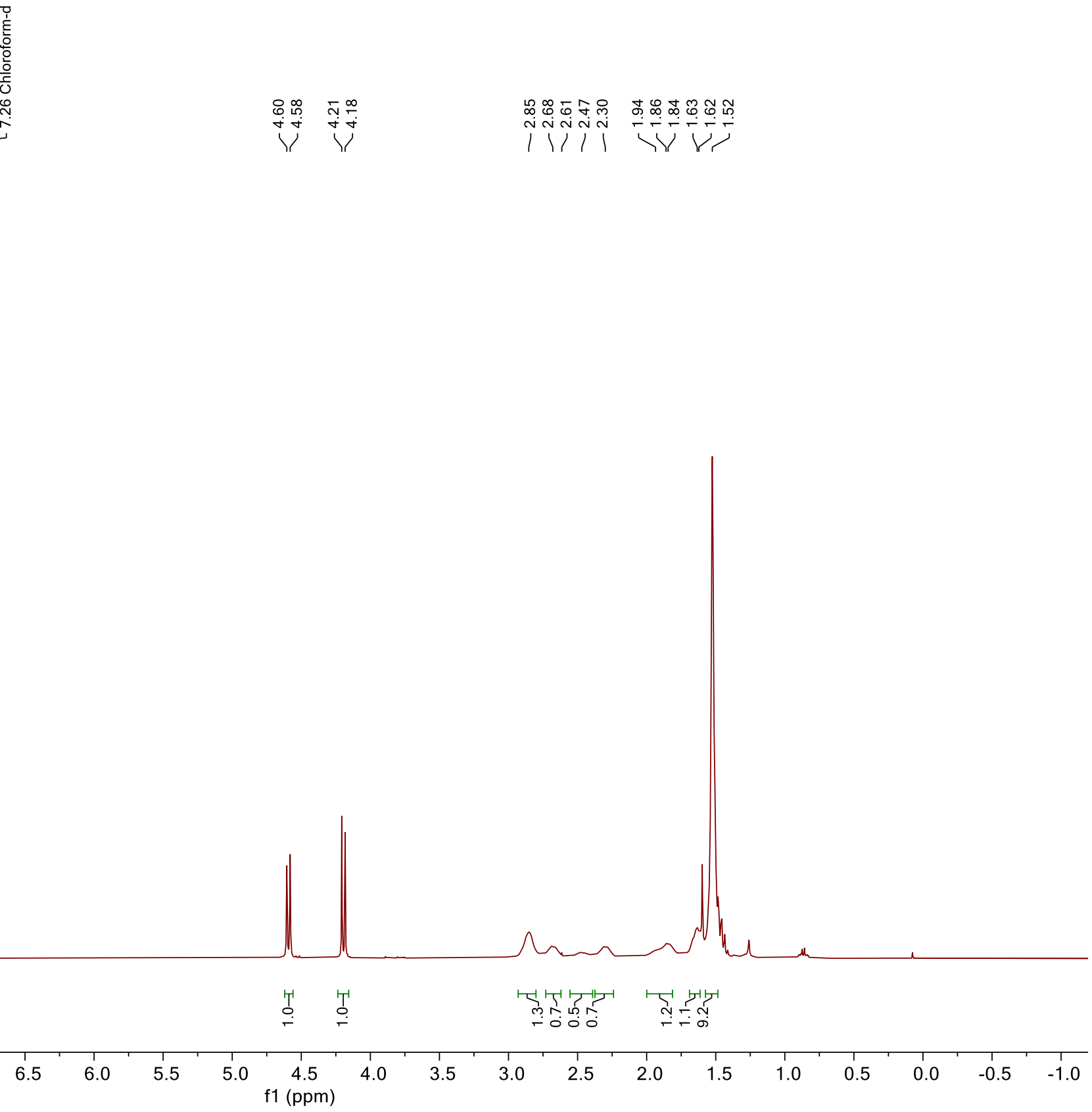


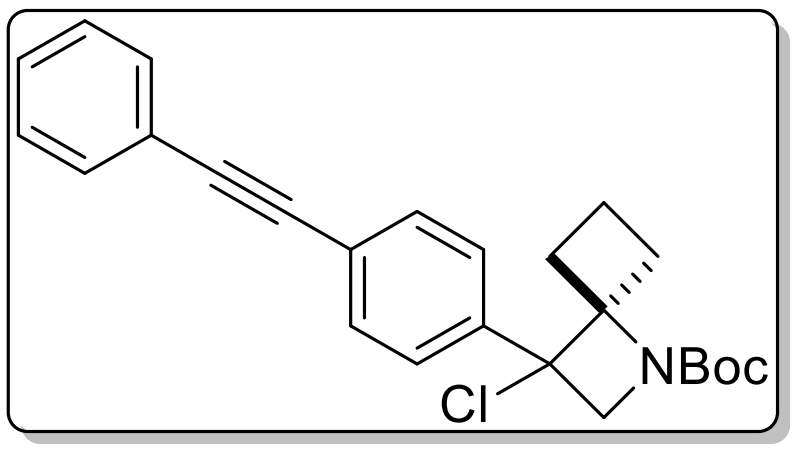


**89**

$^{13}C\ \{^{1}H\}$ NMR (101 MHz, $CDCl_3$)

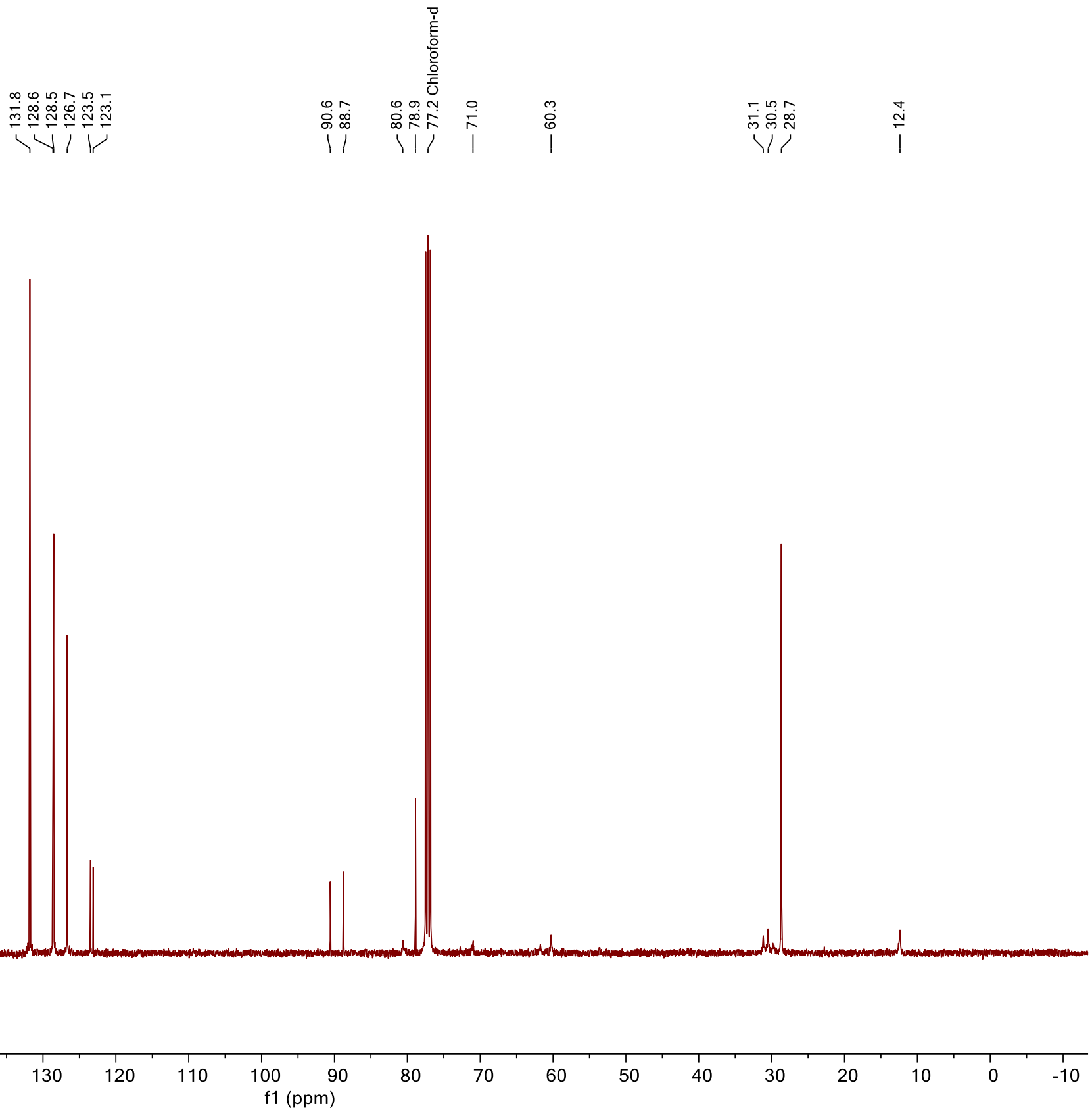

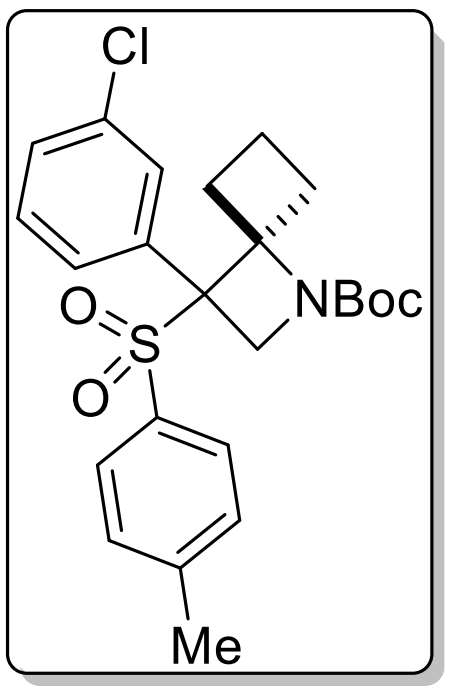


**90**

$^1$H NMR (400 MHz, $CDCl_3$)

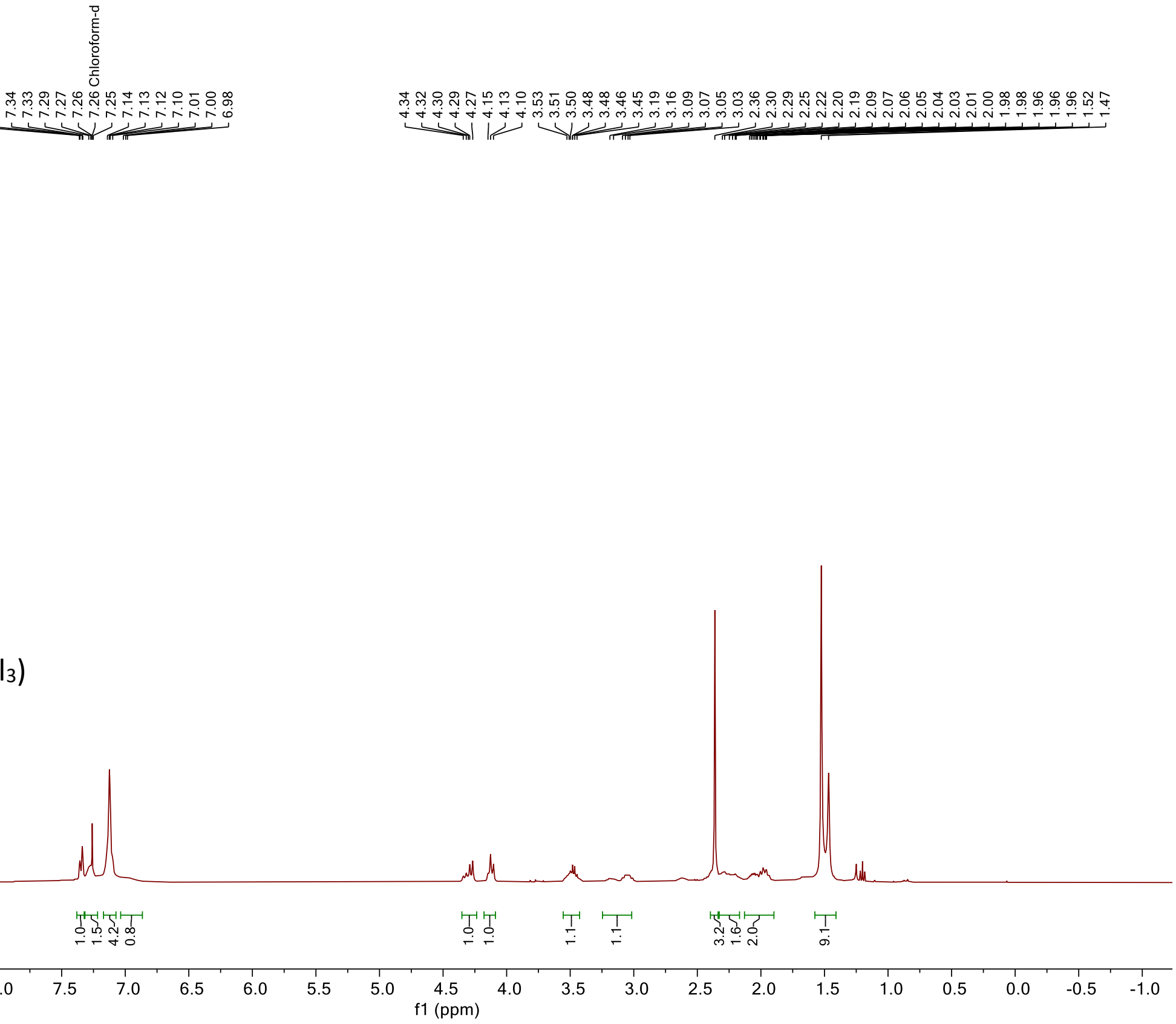


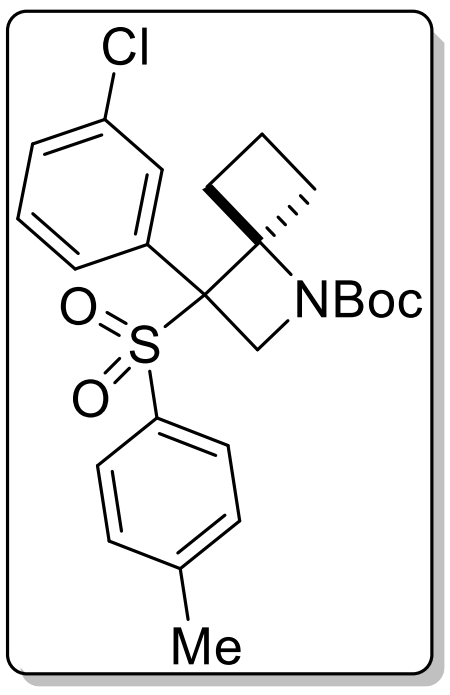


**90**

$^{13}$C {$^1$H} NMR (101 MHz, $CDCl_3$)

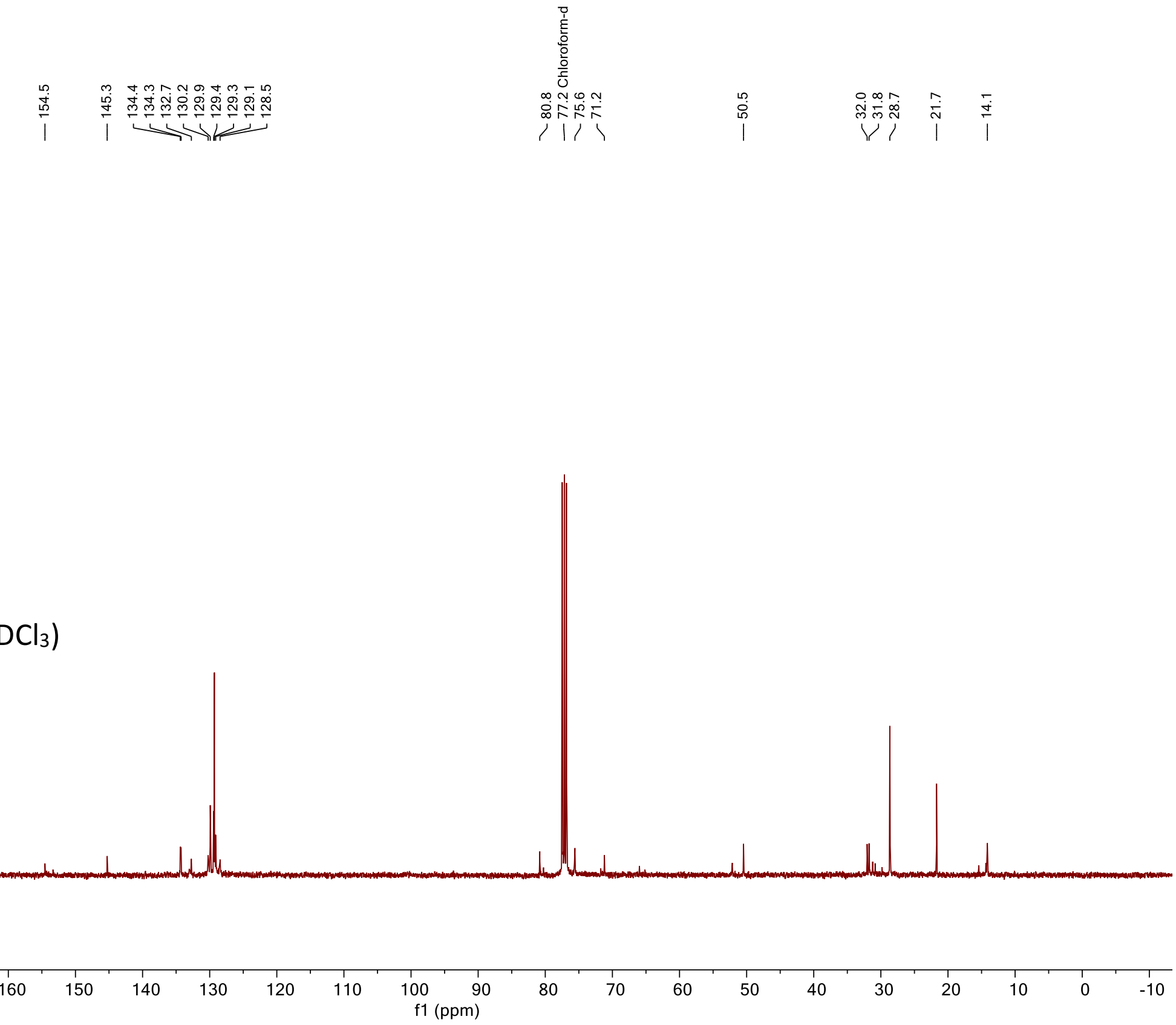

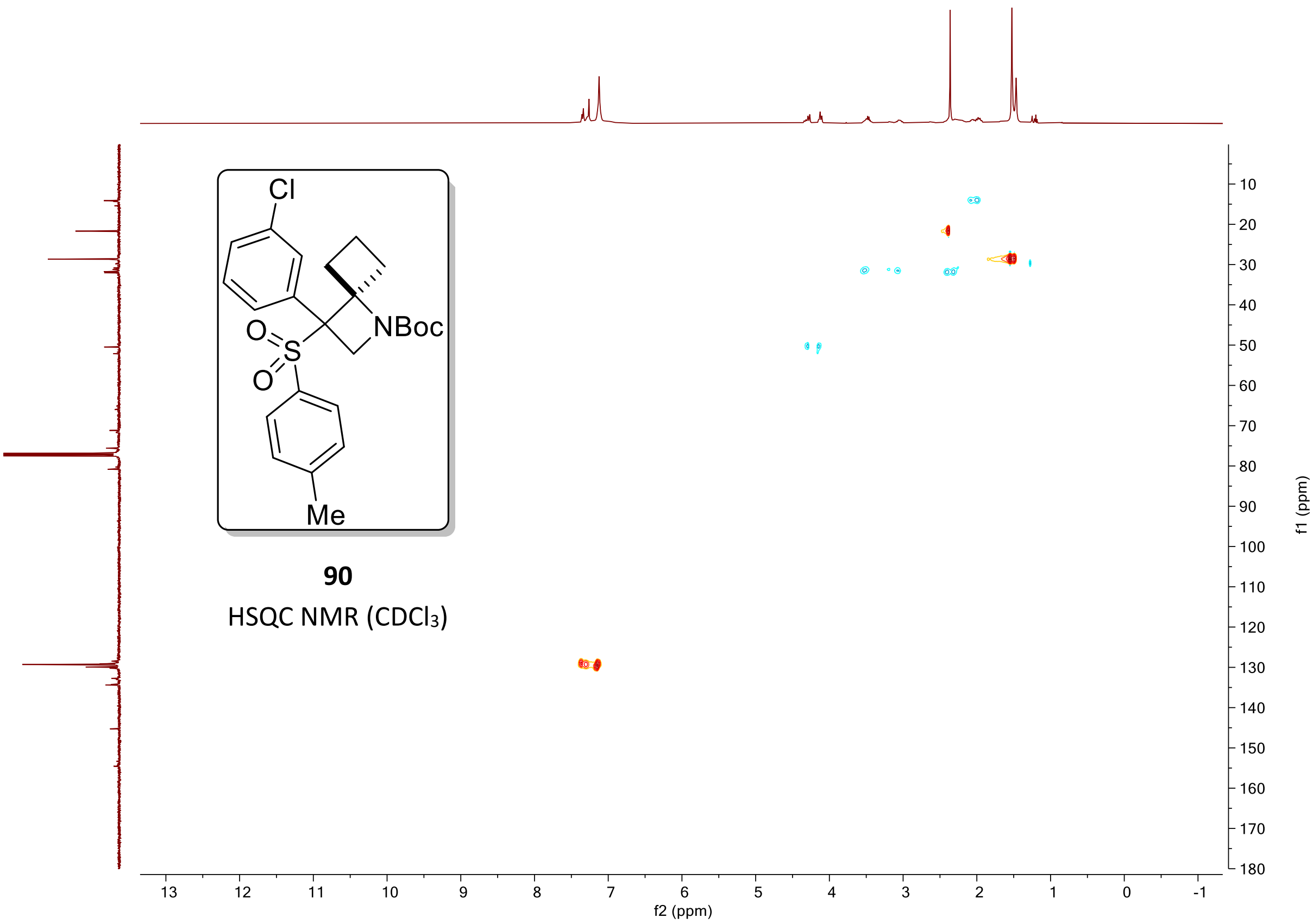


**90**
HSQC NMR ($CDCl_3$)

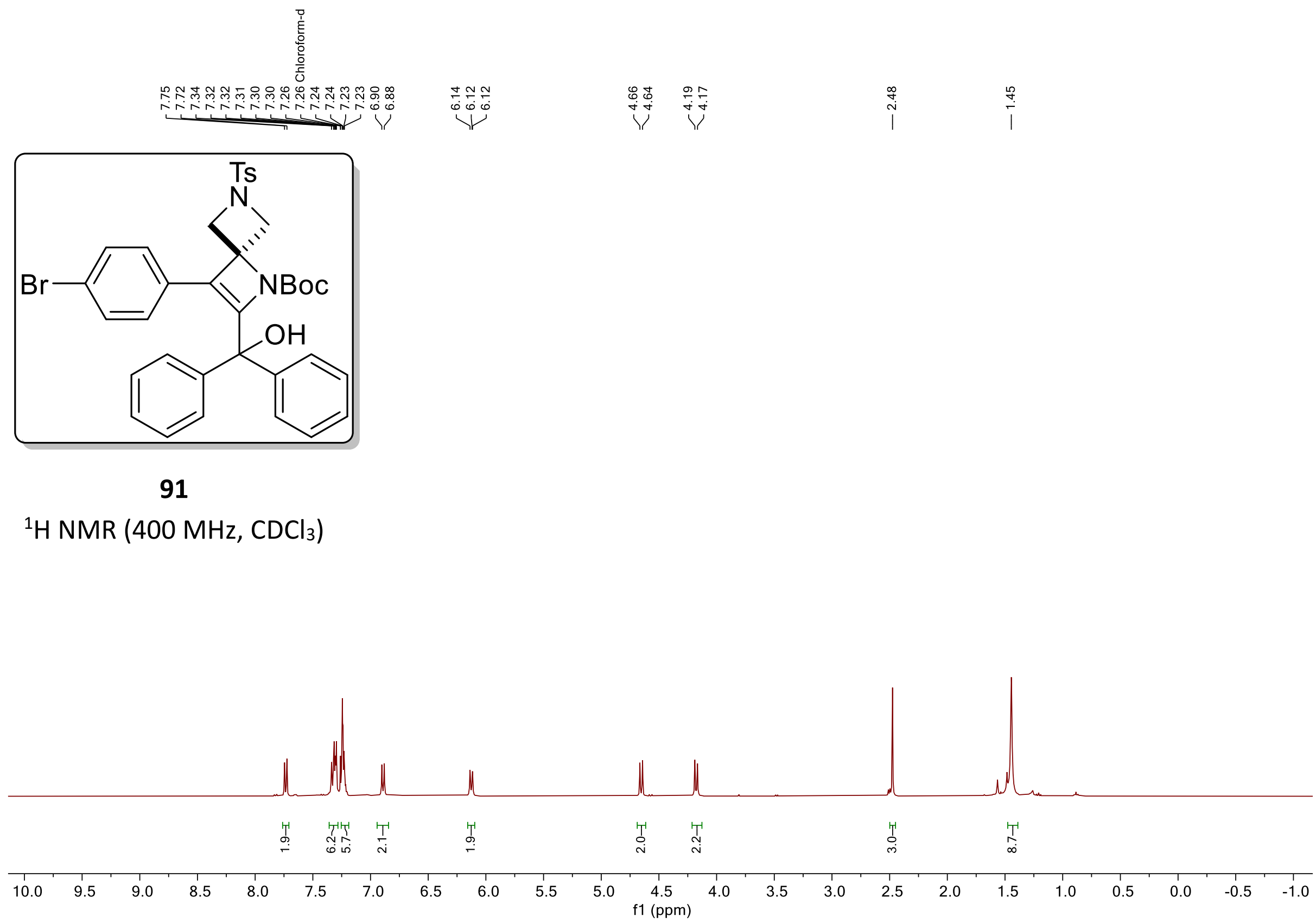


**91**

$^{1}$H NMR (400 MHz, $CDCl_3$)

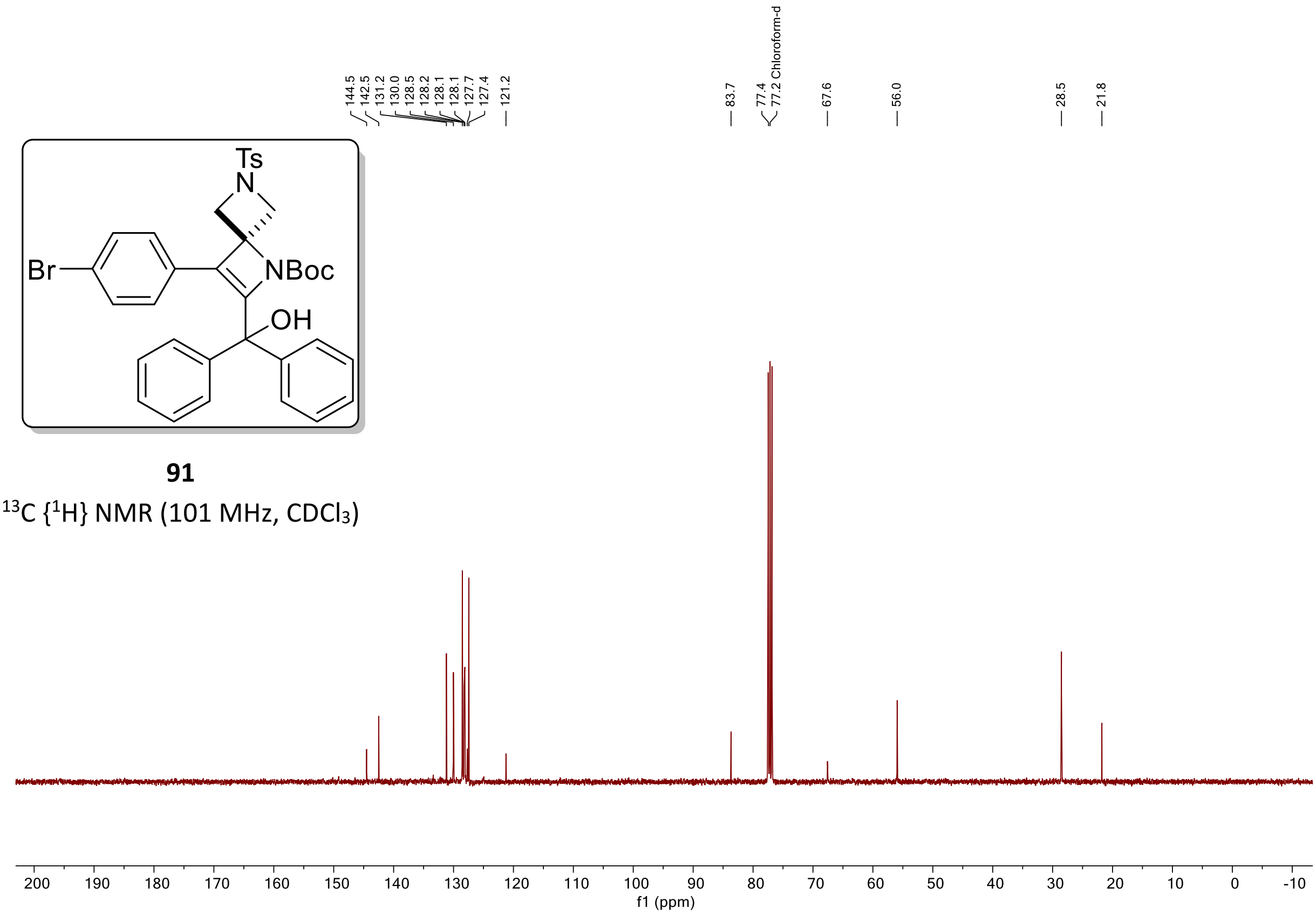


**91**

$^{13}$C {$^{1}$H} NMR (101 MHz, $CDCl_3$)

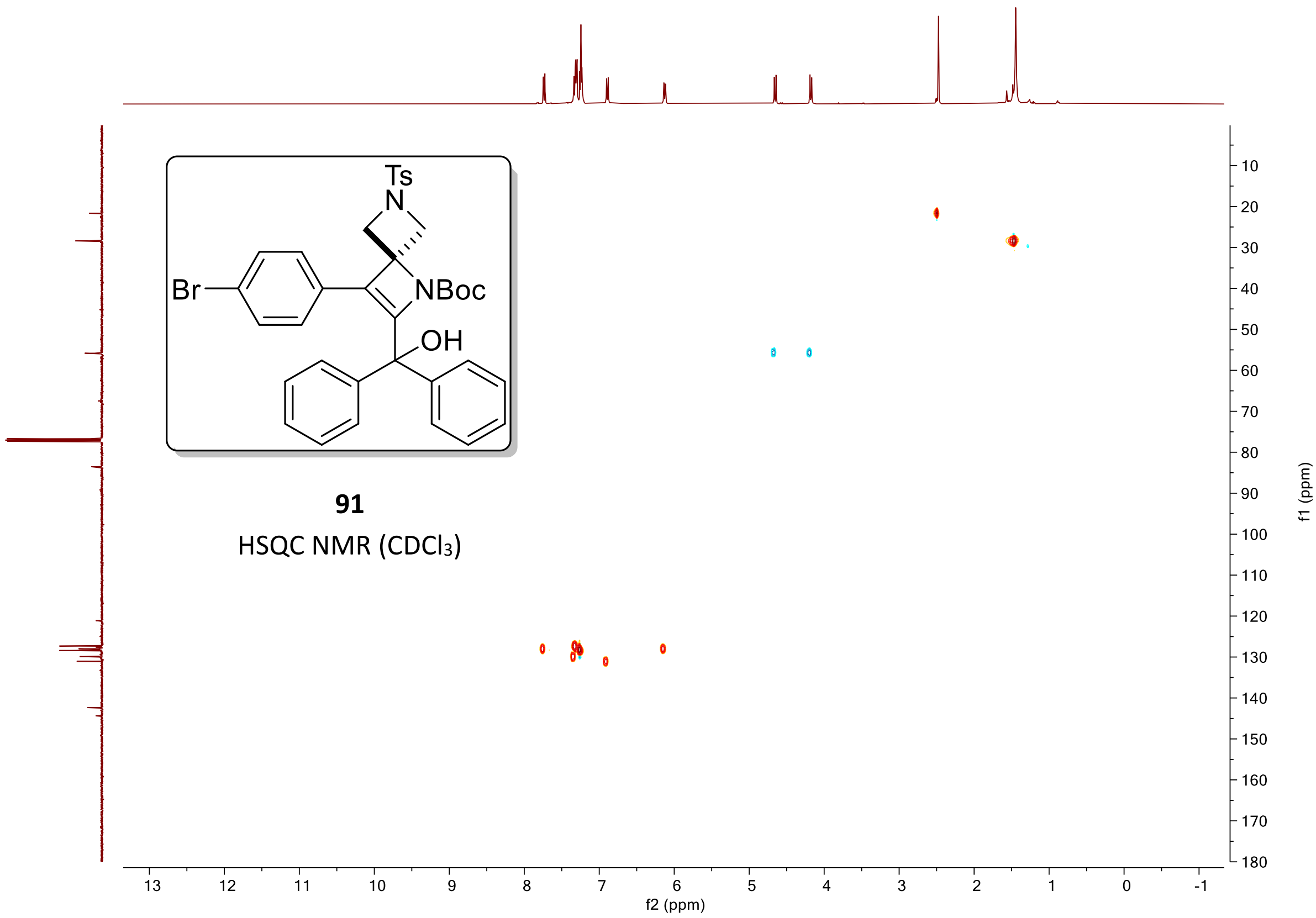


**91**

HSQC NMR ($CDCl_3$)

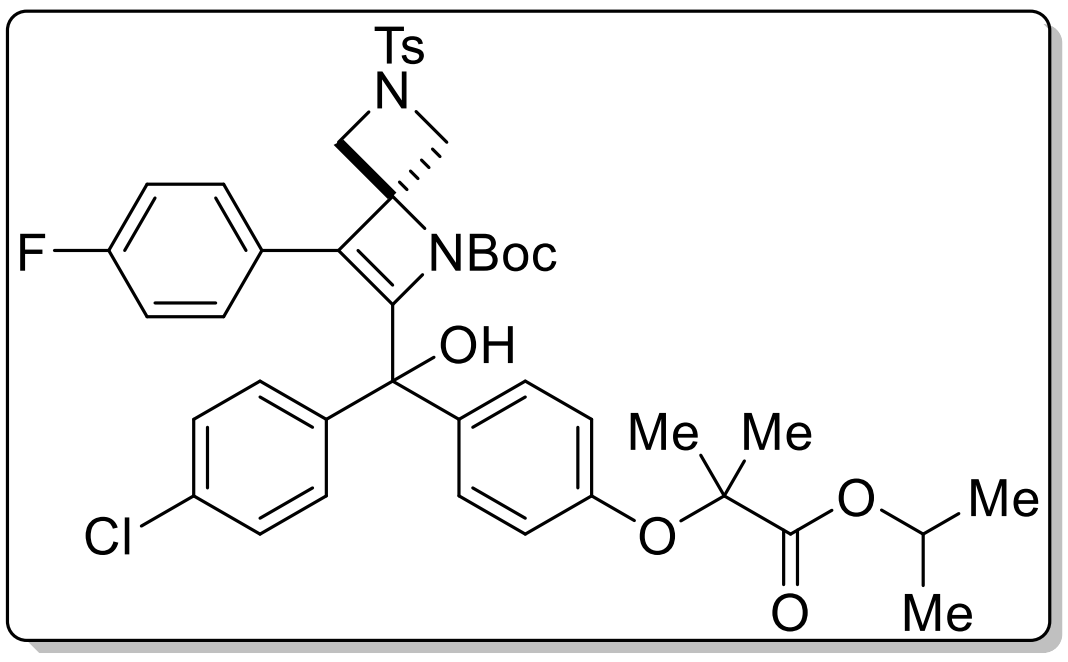

**92**

$^{1}$H NMR (400 MHz, $CDCl_3$)

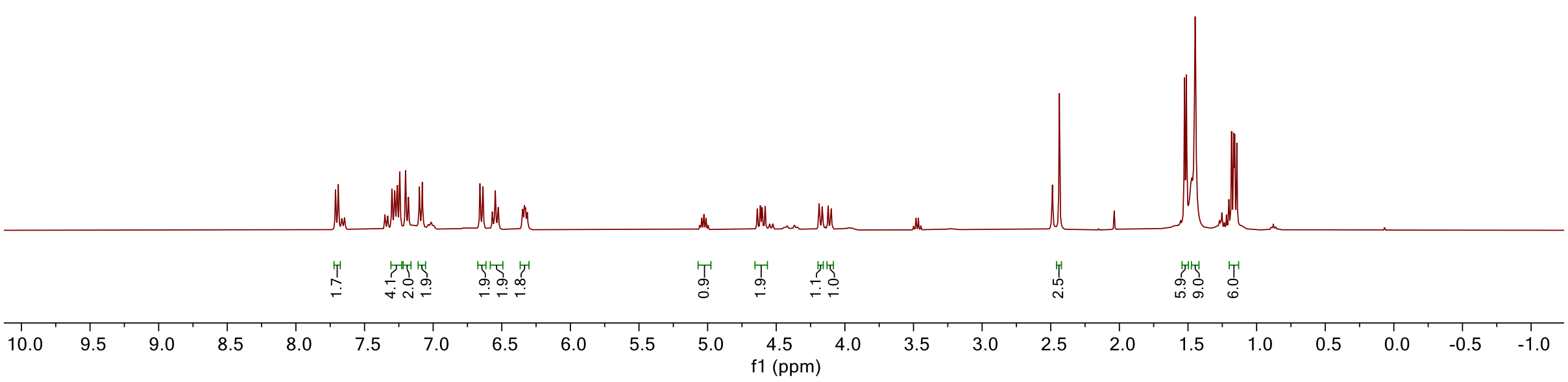


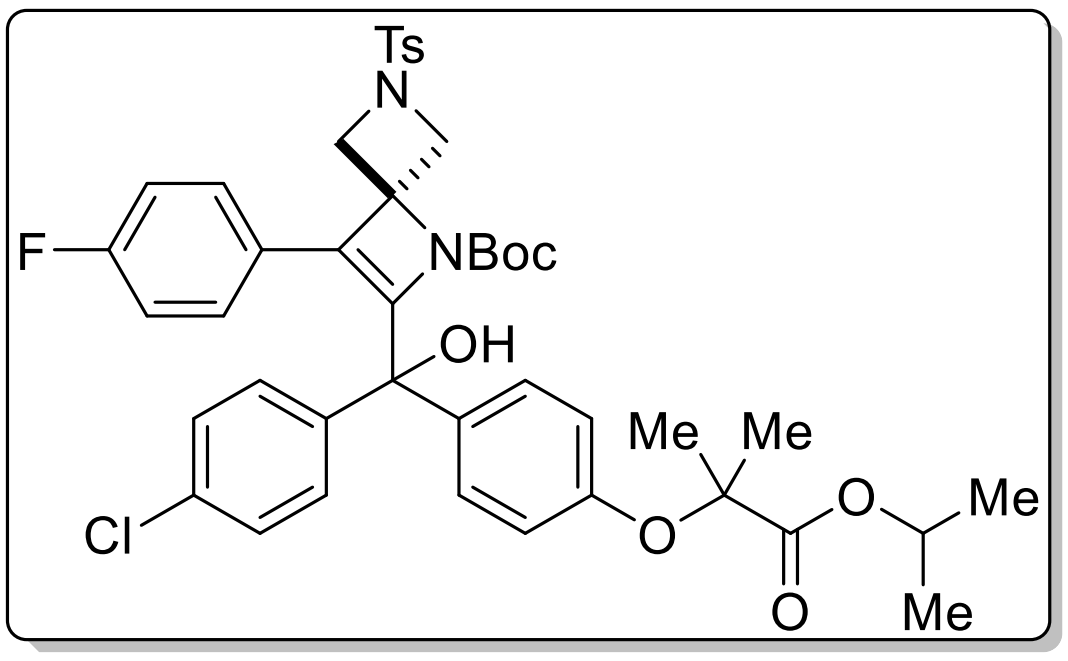

**92**

$^{19}$F NMR (377 MHz, $CDCl_3$)

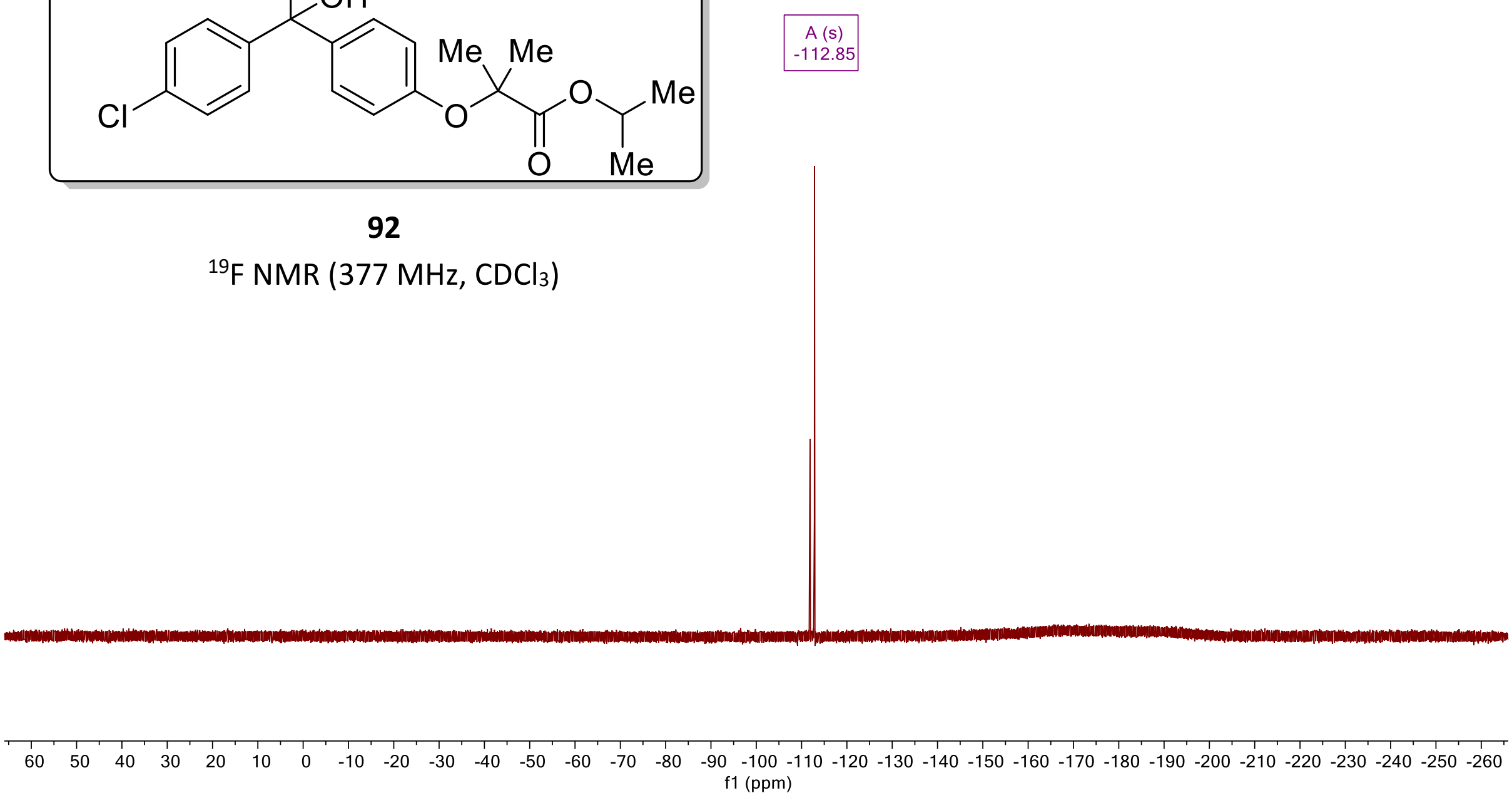

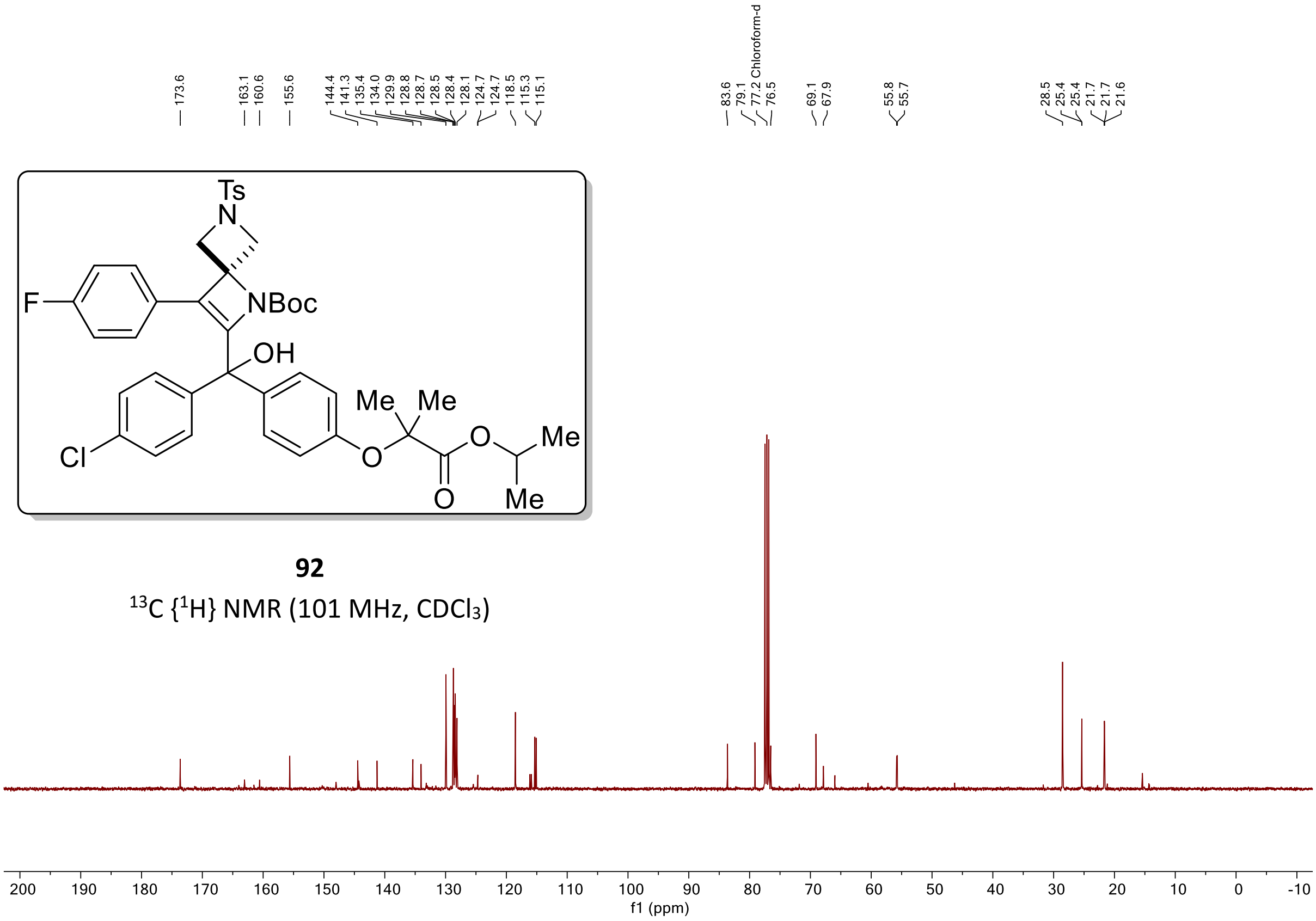


**92**

$^{13}C$ {$^{1}H$} NMR (101 MHz, $CDCl_3$)

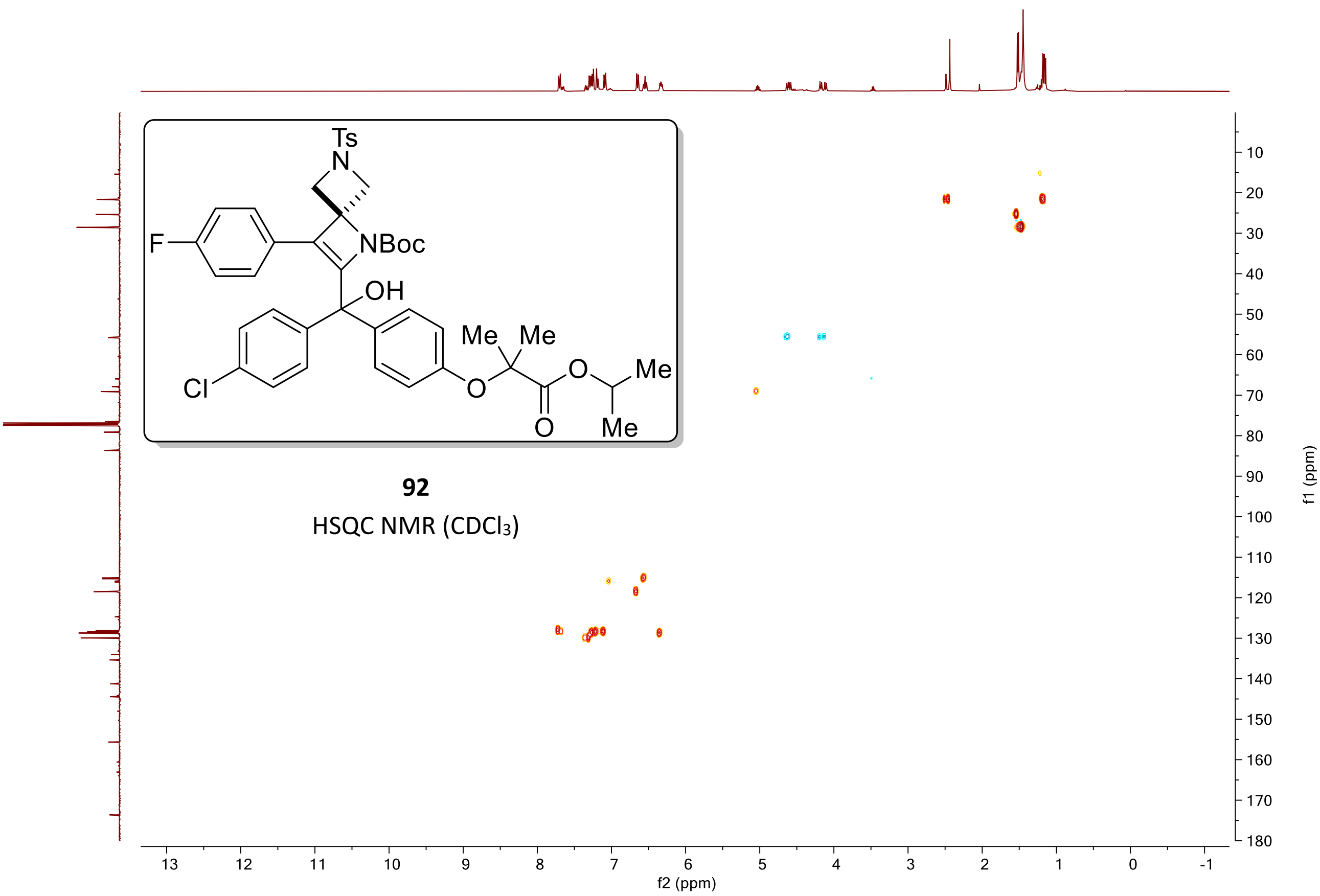


**92**

HSQC NMR ($CDCl_3$)

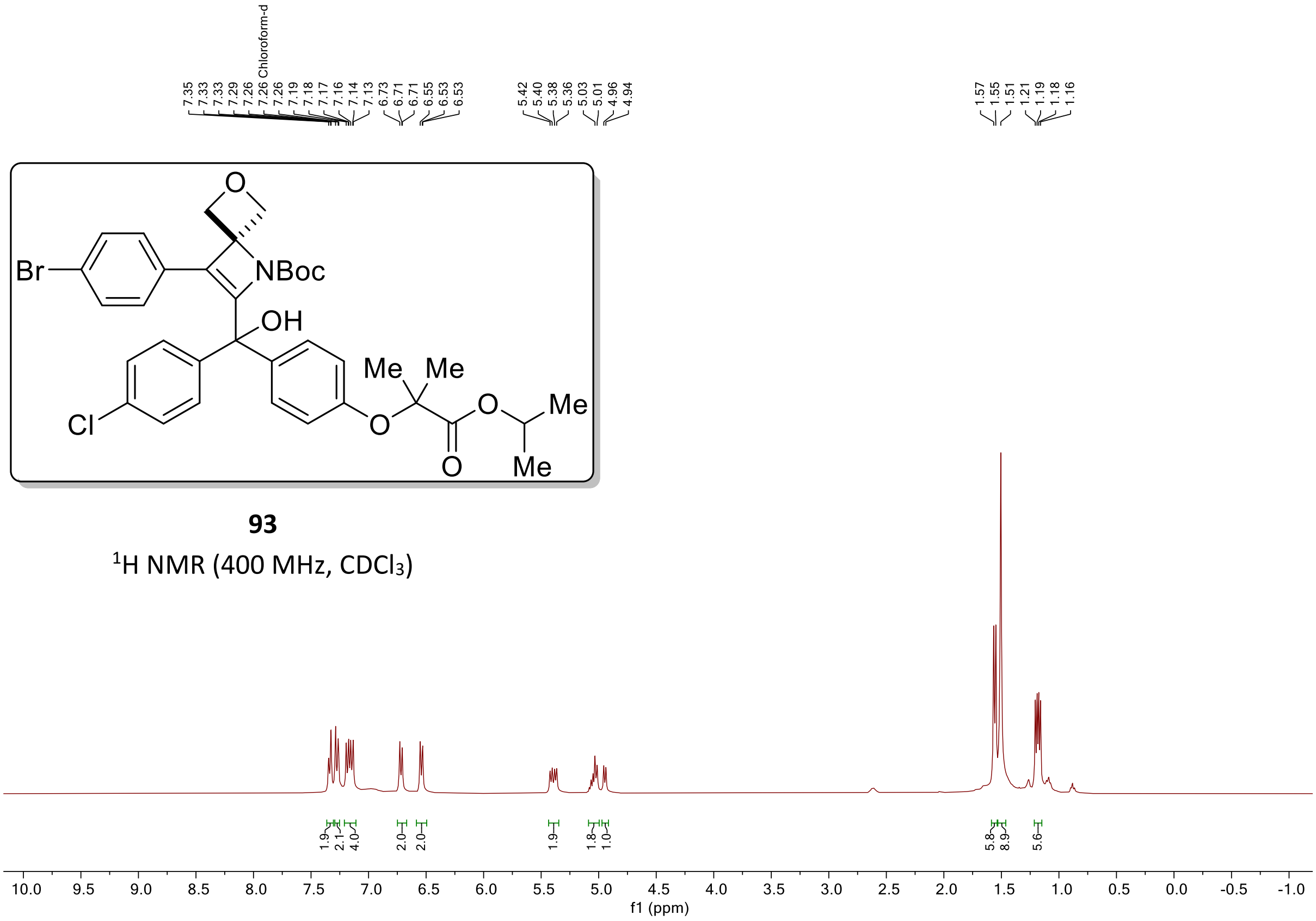


**93**

$^{1}$H NMR (400 MHz, CDCl$_3$)

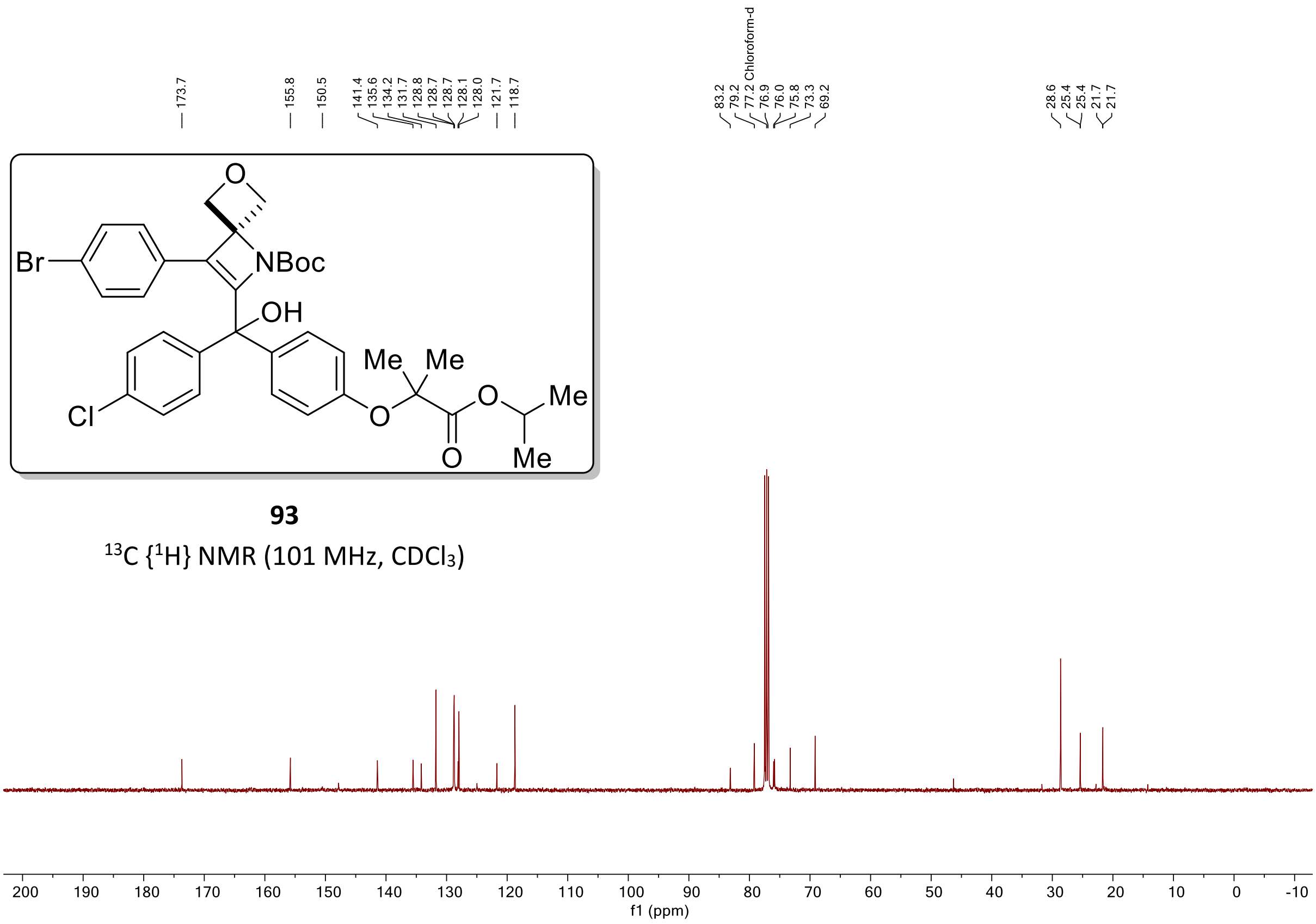


**93**

$^{13}$C {$^{1}$H} NMR (101 MHz, CDCl$_3$)

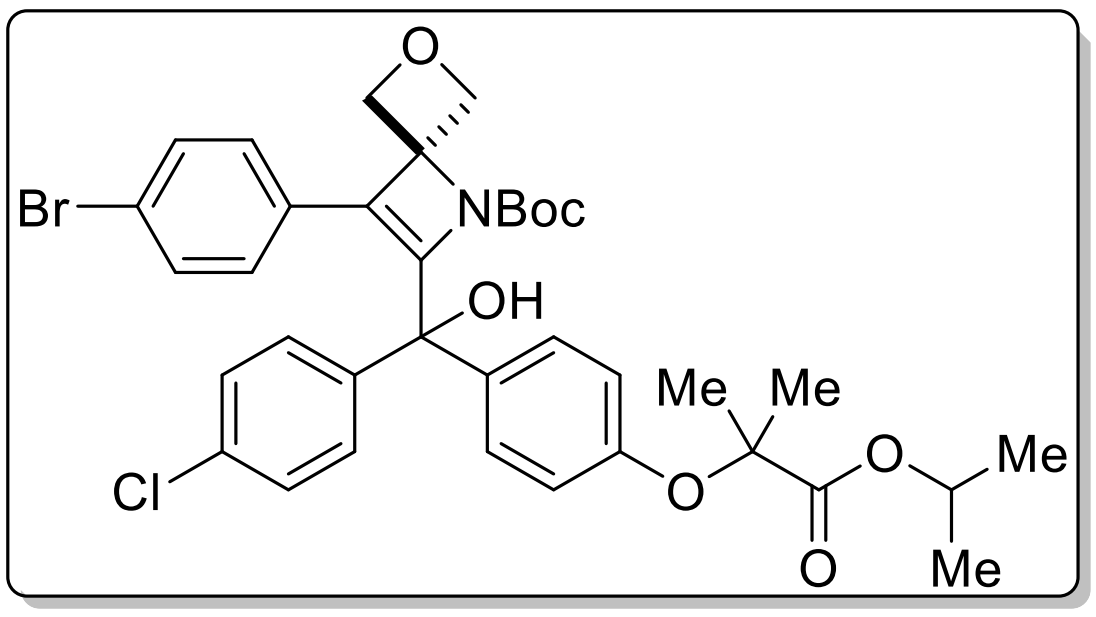


**93**

HSQC NMR ($CDCl_3$)

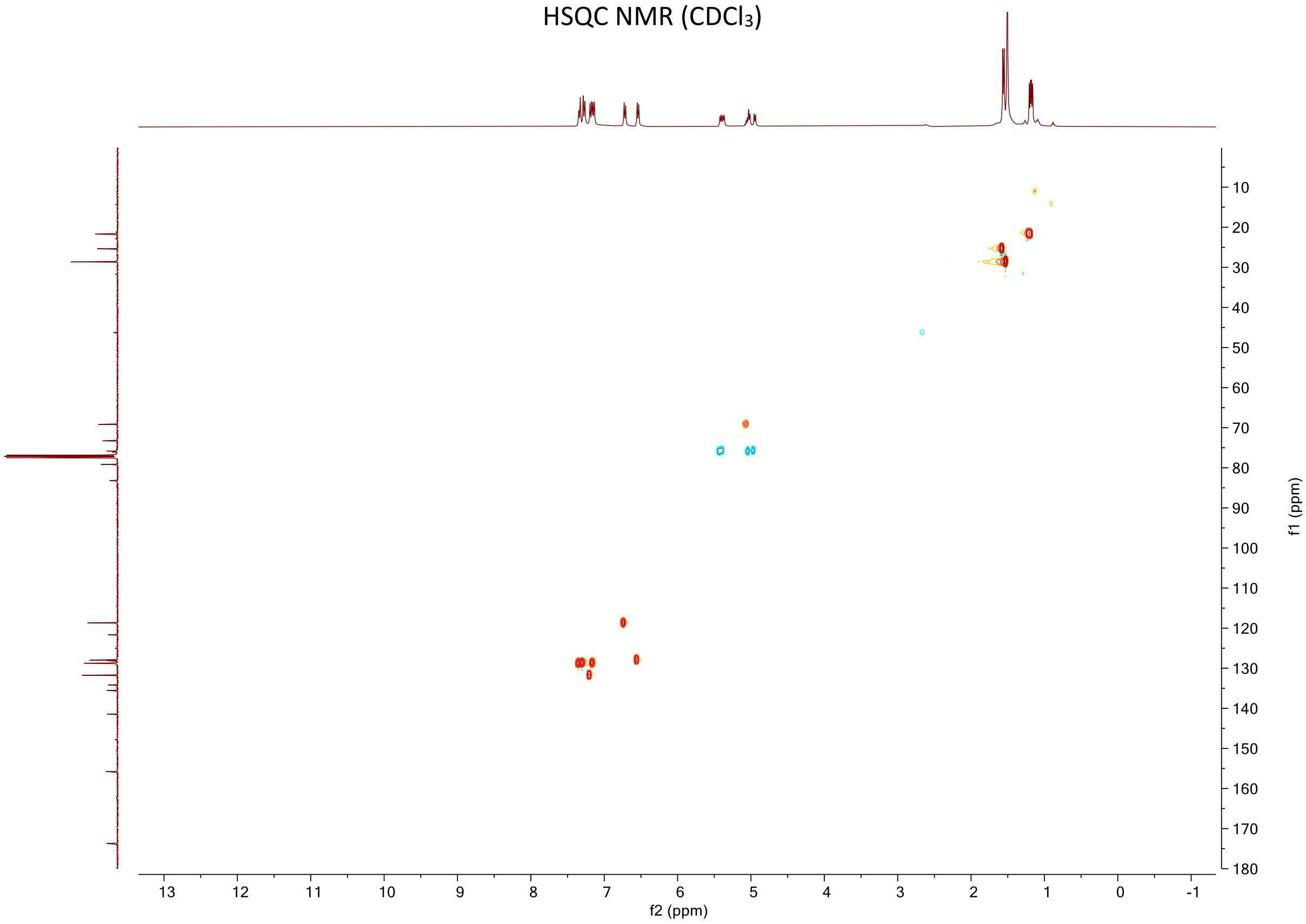

Supplement: Supplementary file 1 [file ANIE-65-e3488479-s003.pdf]
